# Decoding the Mechanism of CheReCunJin Formula in Treating Sjögren's Syndrome Based on Network Pharmacology and Molecular Docking

**DOI:** 10.1155/2022/1193846

**Published:** 2022-09-20

**Authors:** Xiaoyu Xu, Linshuang Wang, Qian Chen, Zikang Wang, Xun Pan, Xike Peng, Miao Wang, Dongfeng Wei, Yanping Li, Bin Wu

**Affiliations:** ^1^College of Traditional Chinese Medicine, Chongqing Medical University, Chongqing, China; ^2^Department of Rheumatology, Chongqing Hospital of Traditional Chinese Medicine, Chongqing, China; ^3^Institute of Basic Research in Clinical Medicine, China Academy of Chinese Medical Sciences, Beijing, China

## Abstract

**Background:**

Sjögren's syndrome (SS) is a chronic autoimmune disease characterized by progressive oral and ocular dryness that correlates poorly with autoimmune damage to the glands. CheReCunJin (CRCJ) formula is a prescription formulated according to the Chinese medicine theory for SS treatment.

**Objective:**

This study aimed to explore the underlying mechanisms of CRCJ against SS.

**Methods:**

The databases, including Traditional Chinese Medicine System Pharmacology, Encyclopedia of Traditional Chinese Medicine, Bioinformatics Analysis Tool for the molecular mechanism of Traditional Chinese Medicine, and Traditional Chinese Medicine Integrated Databases, obtained the active ingredients and predicted targets of CRCJ. Then, DrugBank, Therapeutic Target Database, Genecards, Comparative Toxicogenomics Database, and DisGeNET disease databases were used to screen the predicted targets of SS. Intersected targets of CRCJ and SS were visualized by using Venn diagrams. The overlapping targets were uploaded to the protein–protein interaction network analysis search tool. Cytoscape 3.8.2 software constructed a “compound-targets-disease” network. Gene Ontology and the Kyoto Encyclopedia of Genes and Genomes analyses characterized potential targets' biological functions and pathways. AutoDock Vina 1.1.2 software was used to research and verify chemical effective drug components and critical targets.

**Results:**

From the database, we identified 878 active components and 2578 targets of CRCJ, and 827 SS-related targets. 246 SS-related genes in CRCJ were identified by intersection analysis, and then ten hub genes were identified as crucial potential targets from PPI, including ALB, IL-6, TNF, INS, AKT1, IL1B, VEGFA, TP53, JUN, and TLR4. The process of CRCJ action against SS was mainly involved in human cytomegalovirus infection and Th17 cell differentiation, as well as the toll-like receptor signaling and p53 signaling pathways. Molecular docking showed that the bioactive compounds of CRCJ had a good binding affinity with hub targets.

**Conclusions:**

The results showed that CRCJ could activate multiple pathways and treat SS through multiple compounds and targets. This study lays a foundation for better elucidation of the molecular mechanism of CRCJ in the treatment of SS, and also provides basic guidance for future research on Chinese herbal compounds.

## 1. Introduction

Sjögren's syndrome (SS) is a global systemic autoimmune disease with a prevalence rate second only to rheumatoid arthritis [1, 2]. The condition is characterized by peri-ductal infiltration of the salivary glands (sialadenitis) and lacrimal glands that destroy the secretory function leading to xerostomia/dry mouth and keratoconjunctivitis sicca/dry eyes [3]. It profoundly affects patients' quality of survival and life [4]. However, its aetiology and pathogenic mechanisms have not been elucidated [5]. Therapy for SS in humans is currently focused on symptomatic treatment, often by hormone treatment and means of immunosuppression [6, 7]. However, long-term use of glucocorticoids and immunosuppression can lead to severe toxic side effects, reduced immunity, increased risk of infection, and aggravation of the condition [8]. Traditional Chinese medicine (TCM) has been suggested to be more effective than western medicine in treating SS and preventing adverse reactions [9]. According to TCM theory [10], SS is mainly caused by Yin deficiency and dryness-heat. “Dry” is an essential manifestation of SS disease. TCM believes that the spleen opens at the mouth and the liver opens at the eye. Our previous studies found that the clinical efficacy of the CRCJ formula is significant and can significantly reduce the EULAR SS Patient-Reported Index and EULAR Sjögren's syndrome disease activity index scores of SS patients [11, 12]. The CRCJ formula comprises 12 herbs, including raw Gypsum, Anemarrhena, Rhizoma Phragmites, Prunella, Flos Chrysanthemi, Folium Mori, Astragalus mongholicus Bunge, Glehnia littoralis, Rehmannia glutinosa, Ophiopogon japonicus, Radix Puerariae, and Radix Achyranthis Bidentatae. However, the precise mechanism by which TCM exerts immune regulation effects on SS remains unclear, thereby limiting the clinical application of CRCJ [13].

Network pharmacology is a new strategy based on system biology, bioinformatics, and multidisciplines, which can systematically and comprehensively observe the intervention of drugs on disease networks [14–20]. It is especially suitable for TCM research in treating complex diseases [21, 22]. Molecular docking is a computational method for predicting the optimal interaction of two molecules [23]. Therefore, this study intends to elucidate the underlying mechanism of CRCJ in treating SS based on network pharmacology and molecular docking approach from a holistic viewpoint.

## 2. Material and Methods

The chemical compounds in the CRCJ formula were obtained from the Traditional Chinese Medicine System Pharmacology (TCMSP), Traditional Chinese Medicine Integrated Database (TCMID), Encyclopedia of Traditional Chinese Medicine (ETCM), and Bioinformatics Analysis Tool for Molecular mechANism of Traditional Chinese Medicine (BATMAN-TCM) databases. Subsequently, a herbs-compounds network was constructed using Cytoscape 3.8.2 software. Potential targets were selected according to four drug-target databases: the TCMSP [24], ETCM [25], BATMAN-TCM [26], and TCMID databases [27]. Then, the genes were gathered from five databases (DrugBank, Therapeutic Target Database (TTD), Genecards, Comparative Toxicogenomics Database (CTD), and DisGeNET) and recognized as SS-related targets. Next, the shared genes of the compound and SS-related targets were selected for the hub gene set. In addition, a compound-targets-disease (C-T-D) network was constructed using hub genes in Cytoscape 3.8.2. Gene ontology (GO) and Kyoto Encyclopedia of Genes and Genomes (KEGG) pathway enrichment analyses were conducted via Sangerbox. The binding affinity of a molecular docking simulation verified the relationship between potential active compounds and potential targets, and the bound complexes were visualized using PyMOL 2.3.0 [28]. All the databases, software, and tools used are listed in Table 1.

### 2.1. Collection and Screening of Active Ingredients of CRCJ

Prediction of possible chemical compounds for CRCJ was performed by searching TCMSP, ETCM, BATMAN-TCM, and TCMID databases, using Chinese herbs as keywords. When the same drug possesses various components in multiple databases, we would take the union to gather complete information about the chemical's contents. The parameters for the selection of the active ingredients were set as follows: oral absorption availability (OB) ≥ 30% and drug-likeness (DL) ≥ 0.18 [29]. The active components of the CRCJ formula were screened. Then the Cytoscape 3.8.2 network visualization software was used to visualize the output of the Chinese herbal compounds [30].

### 2.2. Prediction and Screening of Targets of CRCJ Active Components

Based on the selected active ingredients, component-related protein targets were searched and predicted from the TCMSP, ETCM, and BATMAN-TCM databases, and the finding gene targets were standardized using the UniProt KB database [31].

### 2.3. Screening of Targets Related to SS

We constructed reference targets related to SS from the extensive databases DrugBank, TTD, CTD, Genecards, and DisGeNET. Similarly, a reference protein set was established by merging all the disease databases [32–35]. Finally, the collected genes were transferred to potential protein targets.

### 2.4. Construction of an Intersection Target Network

The intersection of the CRCJ-related targets and SS-related targets was selected to construct the protein–protein interactions (PPI) network [36, 37]. Only interactions with a score of ≥0.4 were chosen for the PPI network construction [38], and the PPI network was visualized using the Cytoscape 3.8.2 software. The C-T-D network was established by mapping the possible maps with the active compounds and SS made by Cytoscape 3.8.2. In these networks, compounds, targets, diseases, and herbs were labeled nodes, while edges represented interactions. Cytoscape 3.8.2 plug-in was used to analyze the topology parameters of each node in the network. The cluster analysis was then conducted using MCODE. This plug-in in Cytoscape 3.8.2 was used to identify overlapping proteins and selected vital proteins to construct subnetworks [39].

### 2.5. GO and KEGG Enrichment Analysis

To understand the center of the functional genes and the signaling pathways, the ImageGP was used to carry out functional enrichment to explore GO terms, including “biological processes” (BP), “molecular functions” (MF), “cellular component” (CC), and the KEGG pathway (*p* ≤ 0.05) [40]. Finally, the result file was exported for use with other widely used software tools in the path, including Graph Pad Prism 7 (http://www.graphpad.com) [41] and Sangerbox (http://sangerbox.com/) for data visualization and statistical analysis.

### 2.6. Molecular Docking Analysis

We utilized the AutoDock Vina 1.1.2 software to simulate molecular docking and examine the potential molecular interactions between compounds of the CRCJ formula and hub genes. Firstly, based on the node degree in the PPI, we identified hub genes with nodes larger than 120 degrees. Proteins with three-dimensional structures in the protein database (PDB) and AlphaFold were selected as targets for molecular docking. Then, the compounds to be docked were chosen based on the ordering of compound degree values. According to the target information of the CRCJ, TCMSP, ID, and PubChem CID were matched with the corresponding candidate target components [42]. The structure of compounds was downloaded from the TCMSP database in MOL2 format, or other formats were converted to MOL2 format using OpenBabel. While the 3D structure of the target protein (PDB format) was downloaded from PDB Database (https://www.rcsb.org/). PyMOL 2.3.0 software was employed to identify the active binding sites and visualize software-generated protein structure diagrams. Based on the affinity between the active compound and the target, heatmaps were plotted using Graph Pad Prism 7.

## 3. Results

### 3.1. CRCJ Effective Constituent

**Figure 1:**
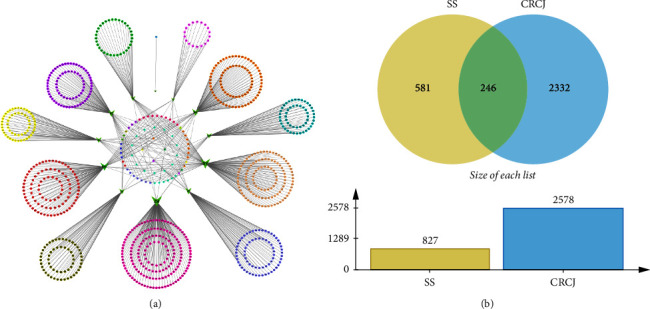
(a) Herb-compound network of 12 herbs and 878 bioactive compounds in CRCJ. Green triangles represent the herbs present in CRCJ. The different colors represent different classes of compounds. The central round portrays the common ingredients in these herbs. (b) Venn diagram of overlapping targets of CRCJ and SS-related targets. There are 246 overlapping targets between them.

The CRCJ formula is composed of 12 types of Chinese herbs. To reveal the potential treatment mechanisms of the CRCJ in SS, we integrated the ingredient information from the TCMID, ETCM, TCMSP, and BATMAN databases. 642, 353, 100, and 279 active ingredients were obtained from the four databases, respectively (Table 2). After the elimination of duplicates, a total of 878 types of active ingredients were retrieved (Supplementary Table 1). We also constructed a network to demonstrate the relationship among Chinese herbs and active compounds with Cytoscape 3.8.2 (Figure 1(a)). Node degree refers to the number of edges associated with the node, also known as correlation degree. The network contained 904 nodes and 986 edges (Supplementary Table 2). The results show that stigmasterol, beta-sitosterol, kaempferol, adenosine, adenine, nucleoside, oleanolic acid, uridine, choline, quercetin, caffeic acid, chlorogenic acid, rutin, and adenine were the 12 compounds with the highest average ranking.

### 3.2. Potential Targets of the CRCJ

To ensure the potential target of the CRCJ in SS, we uploaded 878 compounds to the TCMSP, BATMAN-TCM, and ETCM databases. Finally, 1558, 856, and 306 proteins were found in the BATMAN-TCM, ETCM databases, and TCMSP database, respectively. Each UniProt ID for the targets was converted to the corresponding gene name. As a result, a total of 2578 genes were obtained after removing the repetition (Supplementary Table 3).

### 3.3. Screening of Targets Associated with SS

There were 47, 10, 728, 28, and 132 protein targets in DrugBank, TTD, Genecards, CTD, and DisGeNET databases. 827 SS-related target genes were identified by retrieving public databases after removing the repeat targets (Supplementary Table 4).

### 3.4. Construction of a C-T-D Network and the Protein–Protein Interaction (PPI) Network

**Figure 2:**
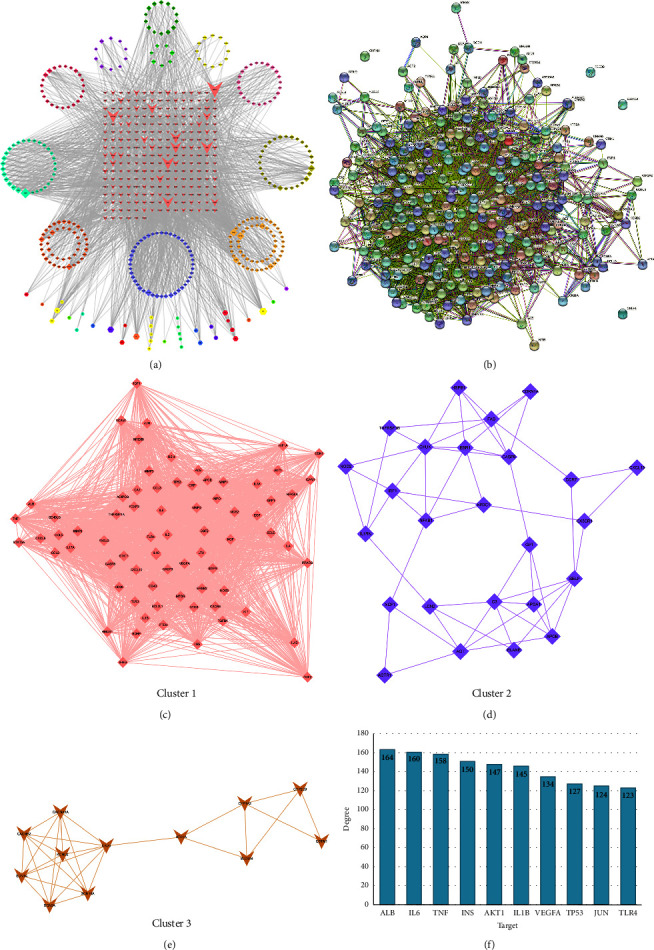
(a) This is the C-T-D network of CRCJ, consisting of 246 targets and 317 active components, among which the red triangle symbol in the middle represents the target and the surrounding rhombus and hexagon represent the active component. (b) The common target PPI network consists of 246 nodes and 5342 edges and the average node degree of 43.4. The 246 rounds represent the potential protein targets of compound CRCJ against SS, and the black lines denote the interaction relationship between the protein targets. (c) The screening process of the PPI network by cytoscape. Module 1 and its core target. (d) Module 2 and its core target. (e) Module 3 and its core target. (f) PPI network of the top ten hub targets. The *y*-axis represents the number of neighboring proteins of the target protein. The *x*-axis represents the target protein. The key potential targets were chosen according to the index of degree value.

When the CRCJ targets were combined with the SS-related targets, it was found that 246 targets of CRCJ were generally SS-related targets (Figure 1(b)). The results reveal that these common targets could be potential therapeutic targets for alleviating the symptoms of SS. Meanwhile, the C-T-D network was constructed using active ingredients, common targets, and diseases (Figure 2(a)), which consist of 246 targets and 317 active ingredients. We used STRING 11.0 to build a PPI network to explore the correlation between the CRCJ and SS-related targets (Figure 2(b)). This network consisted of 246 nodes and 5342 edges in PPI and an average node degree of 43.4. Edge tables saved as CSV files can be imported into Cytoscape 3.8.2 for analysis, visualization, integration, and annotation of complex networks such as molecular interaction networks. In the web, the node represents the target gene. The size and color of the node represent the degree of nodes. The darker the color, the larger the size, and the greater the degree. Three significant modules module 1 (MCODE score = 58.417), module 2 (MCODE score = 5.273), and module 3 (MCODE score = 4.583) were constructed by clustering analysis of the PPI network in Cytotype MCODE (Figures 2(c)–2(e)) (Supplementary Table 5). The size of the nodes and different node colors represent the size of the degree value. The top ten hub targets, namely, ALB, IL-6, TNF, INS, AKT1, ILB1, VEGFA, TP53, JUN, and TLR4, are shown in Figure 2(f).

### 3.5. GO and KEGG Enrichment Analysis

**Figure 3:**
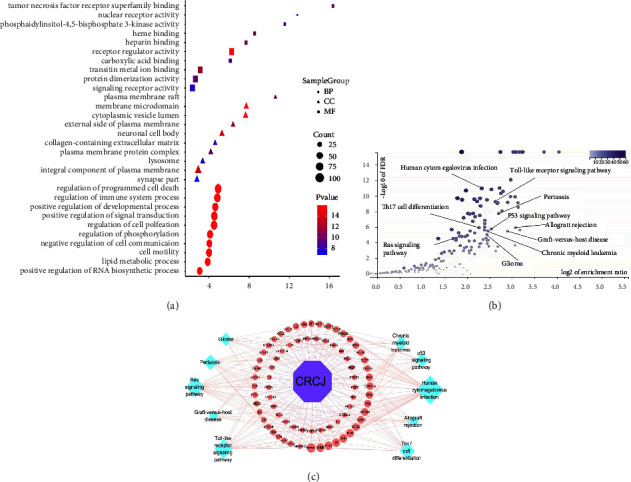
(a) GO enrichment analysis of the common targets. The top ten GO functional terms in BP, CC, and MF were selected (*p* < 0.05), respectively. The color represents the *p*-value, and the size of the spot represents the gene count. (b) KEGG signaling pathway enrichment analysis. The *y*-axis represents the FDR. The *x*-axis represents the enrichment ratio. (c) CRCJ-target-pathway network. The purple hexagon shape, pink circles, and blue circles represent the CRCJ, targets, and KEGG pathway, respectively.

We performed a GO and KEGG enrichment analysis for 246 targets. GO enrichment analyses included BP, CC, and MF. The bubble map of GO terms provided a graphic representation of the ten pathways with the highest BP, CC, and MF concentrations, respectively. Lower *p* values with a red color and higher count with more extensive graphics indicated greater enrichment of GO terms (Figure 3(a)). GO analysis showed that CC terms were widely distributed, mainly located in the exosomes, cytoplasm, mitochondria, endoplasmic reticulum, and perinuclear regions of the cytoplasm. MF terms included binding to zinc, iron, heme iron ions ,and enzymes. BP terms included transcriptional regulation apoptosis, drug reactions, and signal transduction which played a significant role in therapeutic processes. KEGG pathway enrichment analysis (*p* < 0.05) of the 246 proteins identified 19 statistically significant signaling pathways. The volcanic maps were constructed based on the differential *p*-value analysis (Figure 3(b)). KEGG enrichment analysis revealed the top ten enriched pathways were the human cytomegalovirus infection, toll-like receptor signaling, pertussis, allograft rejection, Th17 cell differentiation, p53 signaling, chronic myeloid leukemia, glioma, graft vs host disease, and Ras signaling (Table 3). In addition, the interactions among CRCJ, 73 potential targets, and the top ten pathways were visualized by a CRCJ-target-pathway network (Figure 3(c)).

### 3.6. Binding Affinities between Candidate Targets and Compounds in the CRCJ

**Figure 4:**
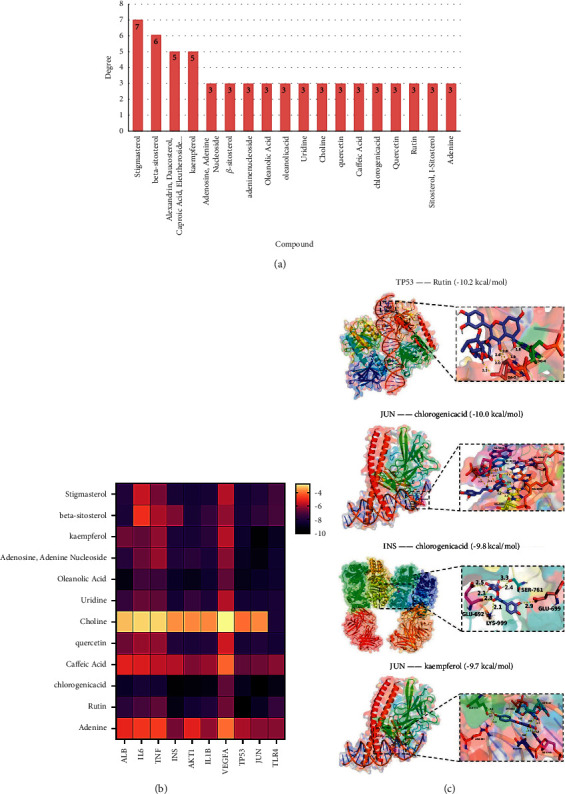
(a) The degree value of candidate compounds. (b) Heat map of molecular docking scores (kcal/mol). (c) Schematic diagram of the docking results. The top four molecular docking diagrams with high binding energies are shown.

To analyze the feasibility of the CRCJ in treating SS, we chose the top 12 compounds based on their degree value, which included stigmasterol, beta-sitosterol, kaempferol, adenosine, adenine, nucleoside, oleanolic acid, uridine, choline, quercetin, caffeic acid, chlorogenic acid, rutin, and adenine. We also conducted molecule docking studies for the top degree ranked compounds in the CRCJ with hub targets (Figure 4(a)). The two-dimensional structures of 12 components and the crystal structures and ligands of 10 targets were obtained. ALB, IL-6, TNF, INS, AKT1, IL1B, VEGFA, TP53, JUN, and TLR4 proteins were selected as putative docking targets. The information is presented in Table 4, including details such as the PDB ID and AlphaFold ID (Table 4). We performed 120 molecular dockings based on the selected chemicals and proteins. The affinity of combinations less than −7 kcal/mol^−1^ indicated good docking activities of hub targets and main compounds, which may contribute to SS treatment. All pharmacodynamic components and marks in sequence, and the results are displayed in the heat map (Figure 4(b)). The complexes with high scores were TP53 and rutin (−10.2 kcal/mol), JUN and chlorogenic acid (−10.0 kcal/mol); INS and chlorogenic acid (−9.8 kcal/mol); JUN and kaempferol (−9.7 kcal/mol); AKT1 and chlorogenic acid (−9.5 kcal/mol); JUN and adenosine and adenine nucleoside (−9.4 kcal/mol); IL1B and chlorogenic acid (9.3 kcal/mol), ALB and oleanolic acid (−9.3 kcal/mol); TLR4 and chlorogenic acid (−9.2 kcal/mol); and INS and rutin (9.2 kcal/mol).  Figure 4(c) shows the top 4 molecular docking diagrams with high binding energies. These findings provided valuable information for the development of SS drugs. The above data collectively suggested that the above active ingredients which have a good affinity with disease targets possibly played a central role in CRCJ for SS treatment.

## 4. Discussion

Currently, the pathogenesis of SS remains unclear. The pathogenesis is multifactorial, related to the innate immune system, autoantibodies, cytokines, epithelial cells, endothelial cells, metabolic disorders, and environmental factors [43, 44]. Previous studies have found that CRCJ can significantly improve patients in tear secretion and salivary secretion function, ESSDAI, and ESSPRI score, as well as lowering of erythrocyte sedimentation rate (ESR) and C-reactive protein (CRP) levels [12]. However, the potential mechanism of SS improvement by CRCJ's active ingredients is rarely studied. In this study, we used a systems pharmacology approach to analyze the underlying pharmacological mechanisms of the CRCJ on SS. Network pharmacology starts with multitarget research, providing a new strategy to study TCM [45].

A total of 878 active components and 246 potential targets were associated with SS in the CRCJ. In a comprehensive analysis of the compound and SS network, the 5 key compounds were found by network pharmacology and molecular docking analysis, including stigmasterol, *β*-sitosterol, kaempferol, and quercetin. Kaempferol is a flavonoid in many plants, and it has a wide range of pharmacological properties, such as anti-inflammatory and antioxidation effects [46–49]. A study demonstrated that SS patients with oxidative stress markers were significantly higher than the healthy control group [50]. Kaempferol is also a natural immunosuppressive agent. It is reported that kaempferol significantly inhibits concanavalin A (ConA) stimulation in early activation of mice T lymphocytes, arrests cell cycle, and inhibits ConA stimulation T cell proliferation. Thus, kaempferol can reduce autoimmune disease and organ transplant rejection caused by the excessive activation and proliferation of T lymphocytes [20, 51, 52]. Some studies reported that kaempferol could promote activating the Ampk/Nrf2/HO-1 signaling pathway and relieve the ox-LDL-induced endothelial injury [53–55]. Other findings showed that kaempferol significantly promoted cell proliferation of regulatory T cells and expressed lower levels of Foxp3. Further, kaempferol could effectively improve the symptoms of arthritis in mice and decrease the PIM1-mediated phosphorylation of S422 at Foxp3. These results suggested that kaempferol can improve autoimmune disorders and inflammatory responses in SS. *β*-sitosterol has many pharmacological activities, including anti-inflammatory, antioxidant, and immunoregulation [56–58].

Quercetin is a natural flavonoid, and research indicates that it exhibits antioxidant, anti-inflammatory, immunoregulation, and many antiallergic bioactive compound activities [59, 60]. Numerous studies have reported that quercetin exerts an anti-inflammatory effect by inhibiting the production of proinflammatory cytokines and decreasing the expression of cyclooxygenase and lipoxygenases [61, 62]. These results show that quercetin can reduce the expression of p38 mitogen-activated protein kinase (MAPK), extracellular signal-related kinase-1/2, and nuclear factor-kappa B (NF-*κ*B) to alleviate the inflammatory response caused by streptococcus suis [63]. Evidence suggests that the NF-*κ*B signal can cause chronic inflammation and ultimately lead to SS [64]. Moreover, it also can reduce proinflammatory cytokine production, such as necrosis factor alpha and interleukin 6 [65]. Quercetin is not only able to inhibit the production of proinflammatory cytokines but also able to increase the secretion of anti-inflammatory cytokines, such as IL-10 [66]. Overall, the interaction of active compounds in CRCJ can systematically synergistically promote different biological responses in the human body, such as anti-inflammatory effects, antioxidative stress, and immunomodulatory activities.

This is consistent with the pathological mechanism of autoimmune diseases, especially SS, and shows that CRCJ has a potential therapeutic effect on SS. We can identify ten interacting target genes by analyzing the PPI network according to the degree value and show that the CRCJ components act on multiple target genes and synergistic interactions among different genes. The top targets obtained according to the rank value were ALB, IL-6, TNF, INS, AKT1, IL1B, VEGFA, TP53, JUN, and TLR4. The results indicate that they may be the key targets of the CRCJ therapy for SS. The results showed that the active components of CRCJ acted on multiple target genes and synergistic interactions among individual genes. Among TNF, IL-6, AKT1, and VEGFA are associated with the development of SS. TNF is mainly produced by innate immune cells and is a critical regulatory cytokine of autoimmunity [67]. One study involved 58 patients with primary Sjögren's syndrome (pSS) with elevated levels of YKL-40, IL-6, and TNF-*α*. The elevated levels are linked to ESSDAI, ESR, and CRP. Simultaneously, SS patients with ANA, anti-SSA antibodies, and anti-SSB antibodies positive have higher TNF-*α* levels [68]. These studies identify that the level of TNF-*α* in the salivary glands of SS patients increases when compared with the normal population [69]. Moreover, the results show that TNF-*α* may promote the development and progression of SS by inducing the oral mucosal epithelium and ocular mucosal epithelial cell apoptosis [70, 71]. Therefore, we speculate that the CRCJ may inhibit apoptosis in salivary gland epithelial cells via blocking TNF-*α*, which in turn affects the target control of glandular cells. IL-6 is a B cell growth and differentiation factor and can promote immunoglobulin synthesis.

IL-6 plays a role in inflammation and B cell maturation. Sun et al. measured the IL-6 levels in serum and salivary glands of 21 pSS postmenopausal patients and 21 healthy postmenopausal women based on enzyme-linked immunosorbent assay [65]. In addition, the patients' immunoglobulins and sedimentation rate of serum [72]. Finally, results indicated that the IL-6 levels in serum and salivary glands of the patient group are higher than the normal group and are positively correlated with the IgG and ESR levels. This study suggests that IL-6 may influence the pathogenesis and development of pSS by affecting the synthesis and secretion of IgG. Combined with the literature analysis, we speculate that CRCJ may inhibit abnormal activation of B cells, thus reducing hyperglobulinemia, which is dominated by increased immunoglobulin G, and the improving SS condition. AKT1 is an essential regulator in AKT1/mTOR signaling pathways, and this pathway in autoimmune diseases such as SS can regulate the IL-17 effect [73]. In SS, salivary gland epithelial cells can produce proangiogenic factors by activating the VEGFA/TACE/VEGFR2/NF-kB axis [74–76], but VEGFA-regulated TACE is responsible for the soluble-TNF-*α* release, and the production of TNF-*α* is closely associated with SS [77]. We speculate that one of the mechanisms of the CRCJ treatment in SS represses VEGFA and AKT1 expression, which in turn reduces inflammatory release. We can clearly see that the multiple active ingredients in the soup interact with various targets with specific functions and treat the disease.

KEGG pathway enrichment analysis results showed that the top ten signaling pathways of CRCJ for SS treatment are as follows: human cytomegalovirus infection, toll-like receptor signaling pathway, pertussis, allograft rejection, Th17 cell differentiation, p53 signaling pathway, chronic myeloid leukemia, glioma, graft vs host disease, and the Ras signaling pathway. Toll-like receptor (TLR) could play an essential role in the pathogenesis of autoimmune disorders. Recent studies have found TLR1, TLR2, and TLR4 mRNA levels are upregulated in the epithelial cells of salivary glands of pSS patients compared with the healthy control group [78]. TLR7 and TLR9 mRNA levels are upregulated in peripheral blood mononuclear cells of pSS patients [79]. Other studies have shown that peptidoglycans can activate TLR2 and induce peripheral blood mononuclear cells in SS patients to produce IL-17 and IL-23 via the IL-6, STAT3, and NF–kB signaling pathways [80]. Therefore, we hypothesize that the CRCJ, by interfering with the signaling pathway of TLR4, can protect salivary gland tissue cells [81]. Additionally, viral infections have long been considered to be highly associated with SS and may be related to human cytomegalovirus (HCMV)-induced stimulation of TLR7 and TLR9. HCMV promotes plasmacytoid dendritic cells to the product I IFN. There are also research findings that I IFN expression is increased in the salivary glands, serum, and peripheral blood of patients with pSS [82]. Thus, we speculate that the CRCJ may improve SS by inhibiting HCMV infection. CRCJ may inhibit Th17 cells, which can secrete IL-17 and IL-22, and which play an essential role in autoimmune diseases and body defense responses. IL-17 is a highly versatile proinflammatory cytokine and can stimulate IL-6, IL-8, TNF-*α*, IL-1*β* and other inflammatory cytokines, which induce the body to produce more inflammatory factors. One study showed that the inflammatory cytokines in small salivary glands of pSS patients are mainly IL17. Moreover, Th17-centric cytokines IL-17, IL-6, IL-23, and IL-12 are significantly increased in the plasma of SS patients [83]. Thus, our study speculated that CRCJ may have participated in the Th17 immune response, and we speculate that CRCJ may have participated in the Th17 immune response to alleviate inflammatory injury in the pSS immune system.

This study demonstrates the synergistic effect of the active compounds on CRCJ. The regulation of the immune and anti-infection and inflammation may contribute to SS treatment. Taken together, the results indicate that CRCJ may promote a reduction in the SS pathological immune network and provide new strategies for immunomodulatory therapies for SS.

## 5. Conclusion

A total of 878 active compounds and 2578 predicted targets in CRCJ and 827 SS-related targets were identified. 246 key targets of CRCJ for SS treatment were obtained including ALB, IL-6, TNF, INS, AKT1, IL1B, VEGFA, TP53, JUN, and TLR4. GO and KEGG enrichment analyses suggested that these key targets are mainly involved in multiple pathways, including the human cytomegalovirus infection, toll-like receptor signaling, Th17 cell differentiation, p53 signaling, pertussis, allograft rejection, and Ras signaling. Besides, the molecular docking study also showed that the main active CRCJ components have a good binding affinity with key protein targets, which provided an important basis for further investigation. This study combines network pharmacology and molecular docking, underlying a foundation for further research on the mechanism of CRCJ.

## Figures and Tables

**Table 1:** The tools and databases used in this study.

Name	Full name	The url
TCMSP	Traditional Chinese medicine systems pharmacology	https://tcmspw.com/tcmsp.php
BATMAN-TCM	A bioinformatics analysis tool for molecular mechanism of traditional Chinese medicine	http://bionet.ncpsb.org.cn/batman-tcm/
ETCM	The encyclopedia of traditional Chinese medicine	http://www.tcmip.cn/ETCM/index.php/Home/
TCMID	Traditional Chinese medicine integrated database	http://www.megabionet.org/tcmid/
PubChem		https://pubchem.ncbi.nlm.nih.gov/
Cytoscape		https://cytoscape.org/
TTD	Therapeutic target database	http://db.idrblab.net/ttd/
CTD	Comparative toxicogenomics database	http://ctdbase.org/
DrugBank		https://go.drugbank.com/
Genecard		https://www.genecards.org/
DisGeNET		https://www.disgenet.org/
STRING		https://www.string-db.org/
UNIPORT		https://www.uniprot.org/
AutoDock vina		https://vina.scripps.edu/index.heml
Pymol		https://pymol.en.softonic.com/download

**Table 2:** Compounds of Chinese herbs in CRCJ.

Herbs	TCMSP	BATMAN	ETCM	TCMID	UNION
Gypsum	0	0	0	1	1
Rehmannia glutinosa	0	9	12	49	61
Ophiopogon japonicus	0	22	52	55	79
Anemarrhena	15	32	41	166	175
Radix Achyranthis Bidentatae	20	10	15	22	47
Glehnia littoralis	8	17	59	82	118
Rhizoma Phragmites	1	9	20	10	25
Radix Puerariae	4	31	33	51	64
Prunella	11	33	39	68	97
Folium Mori	29	73	72	95	156
Flos Chrysanthemi	20	38	16	46	68
Astragalus mongholicus Bunge	20	35	27	70	95
UNION	100	279	353	642	878

**Table 3:** The top 10 enriched KEGG pathway.

KEGG ID and description	Gene set size	Enrichment ratio	*P* value	FDR
hsa05163 : human cytomegalovirus infection	225	4.556	2.87*e*^−13^	9.17*e*^−12^
hsa04620 : toll-like receptor signaling pathway	104	6.160	4.20*e*^−11^	5.48*e*^−10^
hsa05133 : pertussis	76	6.322	8.61*e*^−09^	6.84*e*^−08^
hsa05330 : allograft rejection	38	8.429	1.59*e*^−07^	1.12*e*^−06^
hsa04659 : Th17 cell differentiation	107	4.790	1.67*e*^−07^	1.16*e*^−06^
hsa04115 : p53 signaling pathway	72	5.783	2.63*e*^−07^	1.75*e*^−06^
hsa05520 : chronic myeloid leukemia	76	5.479	5.07*e*^−07^	3.06*e*^−06^
hsa05214 : glioma	71	5.414	1.61*e*^−06^	9.05*e*^−06^
hsa05332 : graft-versus-host disease	41	7.031	3.53*e*^−06^	1.80*e*^−05^
hsa04014 : ras signaling pathway	232	2.899	1.02*e*^−05^	4.66*e*^−05^

**Table 4:** Candidate targets for molecular docking.

Target	Protein	PDB ID	AlphaFold
INS	Insulin	6B70	
ALB	Albumin	1YSX	
IL6	Interleukin-6		P05231
TNF	Tumor necrosis factor		P01375
TP53	Cellular tumor antigen p53	3Q05	
IL1B	Interleukin-1 beta	4DEP	
VEGFA	Vascular endothelial growth factor A		P15692
AKT1	Threonine-protein kinase	4GV1	
JUN	Transcription factor AP-1	1S9K	
TLR4	Toll-like receptor 4	2Z66	

## Data Availability

The data used to support the findings of this study are available from the corresponding author upon request..

## Supplementary Materials

Supplementary Materials Supplementary Materials Table S1: the active ingredients of CRCJ. Table S2: the nodes and edges of the network. Table S3: summary table of drug targets. Table S4: summary table of disease targets. Table S5: network cluster results.
