# Supplementary material for: Decoding the Mechanism of CheReCunJin Formula in Treating Sjögren's Syndrome Based on Network Pharmacology and Molecular Docking
Source: Evid Based Complement Alternat Med. 2022 Sep 20;2022:1193846. doi: 10.1155/2022/1193846 (PMC9553462; doi:10.1155/2022/1193846)
Supplement: Supplementary Materials — Table S1: the active ingredients of CRCJ. Table S2: the nodes and edges of the network. Table S3: summary table of drug targets. Table S4: summary table of disease targets. Table S5: network cluster results. [file 1193846.f1.zip › 1193846.f1/Supplementary Table 1.docx]

Supplementary Table 1

Rehmaglutin D

Campesterol

Glutinoside

Rehmaglutin B

Rehmaglutin A

Gamma-Aminobutyric Acid

Catalpol

Rehmaglutin C

Acteoside

Ophiopogonone B

Ophiopogonin A

Uridine

6-Aldehydo-Isoophipogonone A

Ophiopogonone A

6-Aldehydo-Isoophipogonone B

Ophiopogonanone A

Methyl Ophiopogonanone B

Diosgenin

Guanosine

Ruscogenin

Ophiopogonin C

Methyl Ophiopogonanone A

Ophiopogonanone C

Ophiopogonanone E

N-Trans-Feruloyltyramine

Ophiopogonin D

Ophiopogonin B

Stigmasterol

Ophiopogon B

Ophiopogon A

Orchinol

12-O-Nicotinoylisolineolone

Choline

Neogitogenin

Anemarsaponin B

Anemarrhenasaponin-I

Degalactotigonin

Marmesin

Chrysanthemaxanthin

Monomethyl Lithospermate

Timosaponin A-Iii

Xilingsaponin B

Isomangiferin

Anemarsaponin G

Neoglucobrassicin

Tingenone

Spongesterol

Ginkgetin

Hippeastrine

4-O-Beta-D-Glucopyranosyl Methyl Gallate

Mangiferin

Ginkgolic Acid

Macrostemonoside F

Cis-Hinokiresinol

Isomangiferolic Acid

Mangiferolic Acid

Monomethyl-Cis-Hinokiresinol

Nicotine

Nicotinamide

Smilagenin

Anemarrhenasaponin-Ia

Anemarsaponin E

Sarsasapogenin

Chrysophanol-8-O-Beta-D-(6'-O-Galloyl)-Glucopyranoside

Ecdysterone-3-O-Beta-D-Glucopyranoside

Rubrosterone

Dibutyl Phthalate

Chrysophanol

Spinoside A

Rubschisantherin

Ecdysterone

Alpha-Spinasterol-Beta-D-Glucoside

Inokosterone

Bergamotin

Quercetin

Psoralen

Crenulatin

Isoquercitrin

Isoimperatorin

Imperatorin

Marmesinin

Rutin

Erythritol

Xanthotoxol

Psoralenol

Scopoletin

Caffeicacid

Skimmin

Colchamine

Coixol

Riboflavine

Vitamin B2

Vitamin B1

Collettiside Iii

Thiarubrine A

Tricin

Ribose

Scopine

Kalopanax Saponin C

Sophoradiol

Coumingidine

5-Methylhydantoin

Allantoin

Puerarin-Xyloside

3-Methoxy Pyridine

Scoparone

Genistein

Robinin

Genistein 7-Glucoside

Gamma-Sitosterol

Dauricine

Tuberosin

Daidzein

Formononetin

Puerarin

Genistein 8-C-Glucoside

"7,9-Diacetyltaxayuntin"

Robustadial A

Daidzin

"4',6''-Diacetyl Puerarin"

Kaikasaponin Iii

Puerarol

Arachidic Acid

3'-Methoxypuerarin

Coumestrol

Dalbergenone

Daucosterol

4'-Methoxypuerarin

Methyl Oleanolate

Homoorientin

Niga-Ichigoside F1

Alpha-Phellandrene

Vitamin K

Linalyl Acetate

Fenchone

Hyperoside

Oleanolic Acid-28-O-Beta-D-Glucopyranoside

Methyl Maslinate

Sericoside

Cyanidin

Luteolin

Vulgarsaponin B

Fenchyl Alcohol

Beta-Amyrin

Ursolic Acid

Arjunglucoside I

Caffeic Acid

Cinaroside

Delphinidin

Vulgaxanthin I

Peonin

Alpha-Spinasterol

Hyperin

Beta-Pinene

Methyl Betulinate

Oleanolic Acid

Linalool

Ursolicacid

Myrcene

Moracin G

Mulberrofuran B

Albafuran A

Moracin E

"3,4-Dihydroxydihydgaroaroiuran"

Beta-Sitosterol-3-O-Beta-D-Xylopyranoside

Lupeol

Scopolin

Moracin F

Myoinositol

P-Cresol

Eugenol Methyl Ether

Kuwanone H

Isoramanone

Oxysanguinarine

Albanol A

Maclurin

Moracin H

Kwangsine

"7alpha,21s,25-Trihydroxy-3beta-Acetoxy-21s,23r-Epoxy-9(11)-En-Dammarane"

"26-Hydroxy-Dammara-20,24-Dien-3-One"

Kuwanon H

Isovaleroxy-Hydroxy Dihydrovaltrate

3'-O-Methyl Sappanol

Moscatin

1'-Methoxy-2'-Hydroxydihydromollugin

Mulberrofuran A

Morindin

Moracin B

Chlorogenic Acid

Kuwanon C

Insularine

4-Hydroxycoumarin

Morellic Acid

Isobutyric Acid

Methyl Salicylate

Kuwanon G

Foliosidine

Morin

Moracetin

Paeonolide

"(6s,9r)-Roseoside"

Umbelliferone

Macranthoidin A

Astragalin

Morusin

Moracin C

Paeonol

Guaiacol

Fumaric Acid

4-Prenylresveratrol

Adenine

M-Cresol

3-Hydroxycoumarin

Lupeol Acetate

Eugenol

"5,7-Dihydroxychromone"

5-Hydroxycoumarin

Moracin D

Amygdalin

6-Hydroxycoumarin

Trigonelline

Heptacosane

"2,4-Dihydroxyacetophenone"

Chrysanthenone

Borneol

Chlorochrymorin

Tetradecanal

Chrysanthetriol

Chamazulene

Caryophyllene

Alpha-Pinene

Tricosane

Camphene

P-Cymene

Stachydrine

Chrysanthemol

Camphor

Lonicerin

Alpha-Terpinolene

Alpha-Humulene

Alpha-Terpinene

Chrysanthemin

Aminozide

Sabinene

Tilianin

"1,8-Cineole"

Farnesol

26-Chloro-26-Deoxycryptogenin

Nerolidol

Heneicosane

"3',4'-Dihydroxyacetophenone"

Alpha-Terpineol

Acacetin

Thymol

Astragaloside Vi

2-Hydroxy-3-Methoxystrychnine

Astragaloside Ii

Soyasaponin 1

Cycloastragenol

20-Hexadecanoylingenol

Isorhamnetin

Astramembrannin I

Beta-Sitosterol

Astragaloside Iv

Rhamnocitrin

Suffruticoside A

Betaine

Soyasapogenol B

Kaempferol

Kumatakenin

Astragaloside V

Astragaloside Iii

Astragaloside I

"3,5-Dimethoxystilbene"

Calycosin

Sucrose

Acetic Acid

Canavanine

Astragaloside Vii

Stachyose

"D-Mannitol,Cordycepic Acid"

"Alexandrin,Daucosterol,Caproic Acid,Eleutheroside A,Sitogluside,Strumaroside,Î’-Sitosterol-Î’-D-Glucoside"

8-Epiloganic Acid

Fructose

Geniposide

"Aucuboside,Aucubin"

Ajugol

"D-Glucose,Glucose"

Raffinose

"Adenosine,Adenine Nucleoside"

"Alpha-Humulene,Humulene,Î‘-Humulene"

Î‘-Cyperene

"1-Isopropyl-4-Methylcyclohex-3-Enol,4-Terpineol"

"3,7-Dimethylocta-1,6-Dien-3-Ol,Linalol,Linalool"

Guaiol

Î’-Patchoulene

6-Aldehydo-Isoophiopogone A

6-Aldehydo-Isoophiopogone B

Borneol-2-O-Î’-D-Apiofuranosyl(1â†’6)-Î’-D-Glucopyranoside

Borneol-2-O-Î‘-L-Arabinofuranosyl(1â†’6)-Î’-D-Glucopyranoside

Borneol-2-O-Î’-D-Glucopyranoside

N-( Trans-P-Coumaroyl) Tyramine

"5,7-Dihydroxy-6,8-Dimethyl-3-(4'-Hydroxy-3'-Methoxybenzyl)Chroman-4-One"

"5-Hydroxy-7,8-Dimethoxy-6-Methyl-3-(3',4'-Dihydroxybenzyl) Chroman-4-One"

N-[Î’-Hydroxy-Î’-(4-Hydroxyphenyl)]Ethyl-4-Hydroxy Cinnamide

2'-Hydroxymethylophiopogonone A

Isoophiopogonone A

Jasmololone

Ophiogenin-3-O-Î‘-L-Rhamnopyranosyl(1â†’2)-Î’-D-Glucopyranoside

Ophiopogonanone B

Ophiopogonanone D

Ophiopogonanone F

Ophiopogonin C'

Ophiopogonin D'

Ophiopogonone C

Ophiopogonoside A

Ruscogenin 1-O-Sulfate

"2,5,7-Trihydroxy-6,8-Dimethyl-3-(4'-Methoxybenzyl)Chroman-4-One"

"2,5,7-Trihydroxy-6,8-Dimethyl-3-(3',4'-Methylenedioxybenzyl)-Chroman-4-One"

"5,7,2'-Trihydroxy-6-Methyl-3-(3',4'-Methylenedioxybenzyl) Chromone"

Cyperene

Glycerol

Longifolene

"Niacin,Nicotinic Acid"

Anemarrhenasaponin I

Anemarrhenasaponin Ia

Anemarsaponin C

Anemarsaponin F

Cthd0233276-21

"(25S)-26-O-Î’-D-Glucopyranosyl-22-Hydroxy-5Î’-Furostane-3Î’,26-Diol 3-O-Î’-D-Glucopyranosyl-(1â†’2)-O-Î’-D-Galactopyranoside"

P-Hydroxyphenyl Crotonic Acid

Markogenin

Markogenin3-O-Î’-D-Glucopyranosyl-(1â†’2)-Î’-D-Galactopyranoside

"Nicotinamide,Vitamin Pp"

Pentacosyl Vinyl Ester

Smilagenin-3-O-[Î’-D-Glucopyranosyl-(1â†’2)]-Î’-D-Mannopyranoside

"(5Î’,25S)-Spirostan-3Î’,15Î‘,23Î‘-Diol-3-O-D-Glucopyranosyl-(1â†’2)-Î’-D-Galactopyranoside"

"(5Î’,25S)-Spirostan-3Î’,15Î‘,23Î‘-Triol-3-O-D-Glucopyranosyl-(1â†’2)-Î’-D-Galactopyranoside"

Timobiose

Timosaponin A1

Timosaponin A3

Anemaran A

Anemaran B

Timosaponin A- I

Smilageninoside

Timosaponin A- Iii

Timosaponin B- Ii

Desgalactotigonin

Pantothenate

"Dbp,Dibutyl Phthalate"

Î‘-Spinasterol

"Ecdysterone,Folic Acid"

Î‘-Spinasterol-Î’-D-Glucoside

Achyranthoside I

Achyranthoside Ii

Bidentatoside I

Bidentatoside Ii

Chikusetsu Saponin V Methyl Ester

"Inokosterone,Isoquercitrin"

(25R)-Inokosterone

(25S)-Inokosterone

3-O-trans ferulylquinic acid

"Pavilion,Scopoletin,Scopoletol,Trigonelline"

"Guercetol,Quercetin,Quercetin,Sophoretin,Meletin,Xanthaurine"

"Rutin,Rutoside,Vitamin P"

"3'-Caffeoylquinic Acid,5'-Caffeoylquinic Acid,Chlorogenic Acid,Heriguard"

"Isoquercitrin,Isoquercetrin,Kuwanon H"

"Aspartate,Asparagic Acid,Asparaginic Acid,Aspartic Acid,Skimmin"

Bergapten

Lecithin

Bergaptin

Cephalin

4-[Î’-D-Apiofuranosyl-(1â†’6)-Î’-D-Glucopyranosyloxy]-3-Methoxypropiophenone

Baihuaqianhuoside

"Benzyl Alcohol O-Î’-D-Glucopyranoside,Campesterol"

Bergaptol-O-Î’-D-Glucopyranoside

"Butane-2,3-Diol 2-O-Î’-D-Glucopyranoside"

Citrusin A

Corchoionoside A

1-Deoxy-D-Lyxitol

2-Deoxy-D-Ribitol1

"3,7-Dimethyloct-3(10)-Ene-1,2,6,7-Tetrol"

"(2S,6Î–)-3,7-Dimethyloct-3(10)-Ene-1,2,6,7-Tetrol 1-O-Î’-D-Glucopyranoside"

Ethyl Î’-D-Glucopyranoside

Glehlinoside A

Glehlinoside B

Glehlinoside C

4'-Hydroxyimperatorin 4'-O-Î’-D-Glucopyranoside

(3R')-Hydroxymarmesin 4'-O-Î’-D-Glucopyranoside

"(3R)-2-Hydroxymethylbutane-1,2,3,4-Tetrol"

Icariside F2

Isobutyl Î’-D-Glucopyranoside

Isopropyl Î’-D-Apiofuranosyl-(1â†’6)-Î’-D-Glucopyranoside

Isopropyl Î’-D-Glucopyranoside

Junipediol A 29-O-Î’-D-Glucopyranoside

"Trans-P-Menthane-1Î‘,2Î’,8-Triol"

"(4R)-P-Menth-1-Ene-7,8-Diol 8-O-Î’-D-Glucopyranoside"

"Cis-P-Menth-2-Ene-1Î‘,7,8-Triol"

"Trans-P-Menth-2-Ene-1Î‘,7,8-Triol"

Osthenol-7-O-Î’-Gentiobioside

Oxymarmesinin 5'-O-Î’-D-Glucopyranoside

Phenethyl Î’-D-Glucopyranoside

Picraquassioside A

(X{2212})-Secoisolariciresinol

(X{2212})-Secoisolariciresinol 4-O-Î’-D-Glucopyranoside

(D)-Threitol

Vanillic Acid

Xanthotoxol 8-O-Î’-D-Glucopyranoside

Î’-Amyrin

"Cinnamic Acid,Hydroxycinnamic Acid,P-Coumaric Acid,P-Hydroxy Cinnamic Acid"

P-Hydroxybenzaldehyde

Xylose

Asparagine

"Coniferaldehyde,Coniferyl Aldehyde,Trans-Coniferyl Aldehye"

Syringaldehyde

Gentisic Acid

"Î‘-Tocopher,Tocopherol"

"2,5-Dimethoxy-P-Benzoquinone"

Arabinose

"Sitosterol,Î’-Sitosterol"

"Dimethylesculetin,Scoparone"

"Arachic Acid , Eicosanoic Acid , Arachidic Acid"

Formononetin-7-Glucoside

"(R)-Allantoin,Allantoin"

"Daidzein 4',7-Diglucoside"

"4',6'-Diacetyl Puerarin"

Kudzusapogenol B Methyl Ester

Kudzusapogenol C

7-(6-O-Malonyl-Î’-D-Glucopyransyloxy)-3-(4-Hydroxyphenyl)-4H-1-Benzopyran-4-One

5-Methyl Hydantoin

4'-O-Methylpuerarin

Puerarin-4'-O-D-Glucoside

Puerarin-Xyloside I

Puerarin Xyloside Ii

Pueroside A

Pueroside B

"4',6,7-Trihydroxyisoflavone-6-Methylether-7-O-Î’-D-Xylopyranosyl-(1â†’6)-Î’-D-Glucopyranoside"

"4',5,7-Trihydroxy-6-Methoxyisoflavone7-O-Î’-D-Xylopyranosyl-(1â†’6)-Î’-D-Glucopyranoside"

"Hyperin,Hyperoside,Hyperoside,Quercetin-3-O-Galactoside"

Methyl 2Î‘-Hydroxyursa-28-Oate

3Î’-Hydroxy-Olean-12-En-28-Al

3Î’-Hydroxy-Urs-12-En-28-Al

Methylbetulinate

"Methyl 2Î‘,3Î‘-Dihydroxyursa-12,20(30)-Dien-28-Oate"

"Methyl 2Î‘,3Î‘-Dihydroxyursa-12-En-28-Oate"

Methyl 3-Epimaslinate

"Methyl (13S,14R)2Î‘,3Î‘,24-Trihydroxy-13,14-Cyclo-Oleana-11-En-28-Oate"

"Methyl (12R,13S)2Î‘,3Î‘,24-Trihydroxy-12,13-Cyclo-Taraxer-14-En-28-Oate"

"Methyl 2Î‘,3Î‘,24-Trihydroxyoleana-11,13(18)-Dien-28-Oate"

"Methyl 2Î‘,3Î‘,23-Trihydroxyolean-12-En-28-Oate"

"Methyl 2Î‘,3Î‘,24-Trihydroxyolean-12-En-28-Oate"

"Methyl 2Î‘,3Î‘,24-Trihydroxyursa-12,20(30)-Dien-28-Oate"

Methyl Ursolate

Niga-Ichigoside F2

"Olean-12-En-3Î’,28-Diol"

Pruvuloside A

Pruvuloside B

"(22E,20S,24S)-Stigmasta-7,22-Dien-3-One"

5Î‘-Stigmast-7-En-3Î’-Ol

"2Î‘,3Î‘,24-Trihydroxyursa-12-En-28-Oate"

"Urs-12-En-3Î’,28-Diol"

Vulgarsaponin

Hativene A

Hativene B

Hativene C

"7-Hydroxycoumarin,Skimmetin,Umbelliferone"

Ethyl Palmitate

"Inositol C,Isovaleric Acid"

"4-Hydroxycoumarin,Folinic Acid"

"Ascorbic Acid,Vitamin C"

"Paeonol,Scopoletin"

"Campesterol,M-Cresol"

"Asparagine,Trigonelline"

"Acetic Acid,Propionic Acid"

"Eugenol,Guaiacol"

"5-Hydroxycoumarin,Guaiacol"

"Pentanic Acid,Scopolin"

"3-Hydroxycoumarin,Folic Acid"

"Moran A,Morusin,Paeonol,Quercetin"

"Roseoside,Skimmin"

O-Cresol

"Acetic Acid,Scopolin"

"Methyl Salicylate,Moracin C"

"Isovaleric Acid,Lupeol"

"Lupeol,Moracetin"

"Cudranin,M-Cresol"

Cudranin

"2',4'-Dihydroxy-7-Methoxy-8-Prenylflavan,5,7-Dihydroxychromone"

"2',7-Dihydroxy-4'-Methoxy-8-Prenylflavan"

Inositol C

"Moracin B,N-Methyl-1-Deoxynojirimycin"

"Moracetin,Moracin D"

"Mulberrin,Mulberrochromene,Pentanic Acid"

"(6S,9R)-Roseoside,Mulberrofuran A"

Pipecolic Acid

"Quercetin-3-O-Glucoside,Quercetin-3-O-Glucoside"

Glutamine

"Kuwanon G,Albanin F,Moracenin B,Kuwanon I"

"Butanoic Acid,Butyric Acid"

Hemolysin

Folinic Acid

Apigenin-7-O-Î’-D-(4''-Caffeoyl)Glucuronide

Cacticin

"N-Isobutyl-2E,4E,10E,12Z-Tetradecatetraen-8-Ynamide"

"N-Isobutyl-2E,4E,12E-Tetradecatrien-8,10-Diynamide"

"N-Isobutyl-2E,4E,12Z-Tetradecatrien-8,10-Diynamide"

"Cetylic Acid,Hexadecanoic Acid,Palmitic Acid"

Î’etaine

Medicarpin

Acetyl Astragaloside I

Astragaloside Viii

Astramembrannin Ii

"9,10-Dimethoxy-Pterocarpane-3-O-Î’-D-Glucoside"

Glucuronic Acid

"2'-Hydroxy-3',4'-Dimethoxy-Isoflavane-7-O-Î’-D-Glucoside"

Isoastragaloside I

Isoastragaloside Ii

Astrasieversianin Ix

caso4·2h2o

10-de-oxyeucommiol

3-indolecarboxylic acid

3-methoxy-4-hydroxyl cinnamic aldehyde

4-［2-formyl-5-(hydroxymethyl)-1H-pyrrolyl］ butanoic acid

5-hydroxy-2-pyridinemethanol

"5α,6β-dihydroxy dau-costerol"

6-methyl-3-pyr-idinol

6-O-sec-hydroxyaeginetoyl ajugol

7-isoquinoli-nol

adenosine

cerbrosides

coniferin

diincarvilone A

echinacoside

ferulic acid methyl ester

frehmaglutin B

glutinosalactone A

isoacteoside

jiofuraldehyde dimethyl aceta

leonoside F

martynoside

pterolactam

rehmaionoside a

rehmaionoside c

rehmamegastigmane

rehmannin

rehmannio-side D

rehmapicraside

rehmapicrogenin

rehmapicrogenin monomethyl ester

rehmapiono side a

rehmapiono side b

rehmapiono side c

salidroside

trans-p-hydroxy cinnamic acid methyl ester

β-sitosterol

"2,5,7-trihydroxy-6,8-dimethyl-3-(3',4'-methyl-enedioxybenzyl)-chroman-4-one"

"2,5,7-trihydroxy-6,8-dimethyl-3-(4'-methoxy-benzyl)chroman-4-one"

25(s)-ruscogenin 1-o-alpha-l-rhamnopy-ranosyl-(1-2)-beta-d-xylopyranoside

25(s)-ruscogenin 1-o-[α-l-rhamnopyranosyl-(1→2)][β-d-xylopyra-nosyl-(1→3)]-β-d-fucopyranoside

"5,7,2'-trihydroxy-6-methyl-3-(3',4'-methylene-dioxybenzyl)chromone"

"5,7-dihydroxy-6,8-dime thyl-3-(4'-hydroxy-3'-methoxybenzyl)chroman-4-one"

"5-hydroxy-7,8-dimethoxy-6-methyl-3-(3',4'-dihydroxybenzyl)chroman-4-one"

adeninenucleoside

borneol-2-o-α-l-arabinofuranosyl(1→6)-β-d-glucopyranoside

borneol-2-o-β-d-apiofuranosyl(1→6)-β-d-glucopyranoside

borneol-2-o-β-d-glucopyranoside

methy-lophiopogonone a

methy-lophiopogonone b

methyl beta-orcinol caroxylate

n-(trans-p-coumaroyl)tyramine

n-[β-hydroxy-β-(4-hydroxyphenyl)]ethyl-4-hydroxy cinnamide

oleanolicacid

ophiogenin-3-o-α-l-rhamnopyranosyl(1→2)-β-d-glucopyranoside

stigmasterol-beta-d-glucoside

β-patchoulene

(25 S)一Karatavioside C

(25 S)一Officinalisnin-I

"(25r,s)-5alpha-spirostane-2alpha,3beta-diol 3-o-[o-beta-d-glucopyranosyl-(1-2)-o-beta-d-glucopyranosyl-(1-4)-beta-d-galactopyranoside]"

"(25s)-26-o-β-d-glucopyranosyl-22-hydroxy-5β-furostane-3β,26-diol 3-o-β-d-glucopy-ranosyl-(1→2)-o-β-d-galactopyranoside"

"(3 β, 5 β, 25 s)一Spirostan-3-of"

"(5β,25s)-spirostan-3β,15α,23α-diol-3-o-d-glu-copyranosyl-(1→2)-β-d-galactopyranoside"

"(5β,25s)-spirostan-3β,15α,23α-triol-3-o-d-glu-copyranosyl-(1→2)-β-d-galactopyranoside"

(E)-4'-demethyl-6-methyleucomin

"(E)-5,7-dihydroxy-3一(4'-hydroxybenzylidene)chroman-4-one"

"(Z)-4',4一(3-nethenyl-1-propene-l,3-diyl)bisphenol"

"2',4',4-trihydroxychalcone"

3-pyridylcarbinol

"4,4'-dihydroxychalcone"

4-hydroxybenzoic acid

7-hydroxy-3一(4-hydroxybenzyl)chroman

anemaran

"anemarans a, b, c, d"

anemarchalconyn

anemarcoumarinA

Anemarnoside A

Anemarnoside B

AnemarrhenasapininIII

Anemarrhenasaponin II

aurantiamide

aurantiamide acetate

B TT

B V(C57)

baohuoside

benzoic acid

BI

broussonin a

broussonin b

cishinokiresinol

coumaroyltyramine

cyclo(Tyr-Leu)

desglucolanatigonin ii

dosgenin

emodin

foliamangiferosideA

icariside ii

iriflophenone

ismangiferin

Isoarsasapogenin

isosakuranetin

makrkogenin-3-o-beta-d-glucopyranosyl-(1->2)-beta-d-galactopyranoside

manguferin

markogein

markogenin-3-o-beta-d-glucopyranosyl-(1-2)-beta-d-galactopyranoside

markogenin3-o-β-d-glucopyranosyl-(1→2)-β-d-galactopyranoside

Markosapogenin

monomethy-cis-hinokiresinol

n-cis-feruloyltyramine

neogitogein

neomangiferin

nicotinicacid

oxy- hinokiresinol

oxy-cis-hinokiresinol

p-hydroxyphenyl crotonicacid

palmitic acid

pantothenic acid

Purpureagitoside

sarsasapogenin 3-o-4-rhamnosyl-sophoroside

sarsasapogenin-3-o-beta-d-glucopyranosyl(1->2)-beta-galactopyranoside

smilagenin-3-o-[β-d-glucopyranosyl-(1→2)]-β-d-mannopyranoside

smilagenin-3-o[beta-d-glucopyranosyl-(1-2)]-beta-d-mannopyranoside

smilagenone

stearic acid

Timosaponin

Timosaponin A I

Timosaponin A II

"timosaponin a- i, a- ii, a- iii, a- iv, b- i & b- ii"

timosaponin a-1

timosaponin AIII

Timosaponin AIV

Timosaponin B III

Timosaponin B IV(C51)

Timosaponin B IV(C57)

Timosaponin B V(C46)

Timosaponin B VI

timosaponin BI / anemarsaponin E

timosaponin BII

timosaponin BIII / anemarsaponin B

Timosaponin D 1

Timosaponin D2

timosaponin E1

Timosaponin F(C39)

Timosaponin G(C39)

Timosaponin G(C50)

Timosaponin J

Timosaponin K

Timosaponin L

Timosaponin0

TimosaponinC

TimosaponinC 1

TimosaponinC2

TimosaponinD

TimosaponinE 1

TimosaponinE2

TimosaponinF(C50)

TimosaponinH 1

TimosaponinN

xuelianlacton e

zimoside A

di-m-butyl uralsaponin a esters

inophyllolide

α-spinasterol

α-spinasterol-β-d-glucoside

"(2s,6 ζ)-3,7-dimethyloct-3(10)-ene-1,2,6,7-tetrol1-o-β-d-glucopyranoside"

(3r ')-hydroxymarmesin 4'-o-β-d-gluco-pyranoside

"(4r)-p-menth-1-ene-7,8-diol 8-o-β-d-glucopyra-noside"

"(8E)heptadecyl-1,8-diene-4,6-diene-3,10-diol"

(?)-secoisolariciresinol

(?)-secoisolariciresinol4-o-β-d-glucopyranoside

4''-hydroxyimperatorin 4''-o-β-d-glucopyra-noside

4-[β-d-apiofuranosyl-(1→6)-β-d-glucopyra-nosyloxy]-3-methoxypropiophenone

benzyl alcoholo-β-d-glucopyranoside

bergaptol-o-β-d-glucopyranoside

"butane-2,3-diol 2-o-β-d-glucopyranoside"

Butanol α-D- fructofuranoside

chlorogenicacid

cis-falcarindiol

"cis-p-menth-2-ene-1α,7,8-triol"

Cool in the bergamot

Do isoimperatorin lactone

ethylβ-d-glucopyranoside

Fa Kalin diol

ferulic acid

ferulicacid

isobutyl β-d-glucopyranoside

Isopropyl apioglucoside

isopropylβ-d-glucopyranoside

junipediol a 29-o-β-d-glucopyranoside

linolenic acid

osthenol-7-o-beta-gentiobioside

osthenol-7-o-β-gentiobioside

oxymarmesinin 5'-o-β-d-glucopyranoside

panaxynol

pehllopterin

phenethylβ-d-glucopyranoside

salicylic acid

Stigmasterol 3-O-β-D- glucopyranoside

syringin

"Trans, trans-2,4-decadienal"

Trans-2-octene-1-ol

"trans-p-menth-2-ene-1α,7,8-triol"

"trans-p-menthane-1α,2β,8-triol"

vanillicacid

Vanilloid

Vanilloid 4-O-β-D- pyranoside

xanthotoxin

xanthotoxol 8-o-β-d-glucopyranoside

tricin-7-o-beta-d-glucopyranoside

1-formyl-beta-carboline

"4',5,7-trihydroxy-6-methoxyisoflavone7-o-β-d-xylopyranosyl-(1→6)-β-d-glucopyrano-side"

"4',6,7-trihydroxyisoflavone-6-methylether-7-o-β-d-xylopyranosyl-(1→6)-β-d-glucopyranoside"

6-malonylgypenoside v

7-(6-o-malonyl-β-d-glucopyransyloxy)-3-(4-hydroxyphenyl)-4h-1-benzopyran-4-one

arachidicacid

methyl-p-hydroxycinnamate

pueroside

pukeenside

"1, 8-cineole"

"2α,3α,24-trihydroxyursa-12-en-28-oate"

3-oxoalanine

3alpha-dihydroxyursen-12-en-28-oate

3β-hydroxy-olean-12-en-28-al

3β-hydroxy-urs-12-en-28-al

5α-stigmast-7-en-3β-ol

deucosterol

homoovientin

"methyl 2α,3α,23-trihydroxyolean-12-en-28-oate"

"methyl 2α,3α,24-trihydroxyolean-12-en-28-oate"

"methyl 2α,3α,24-trihydroxyoleana-11,13(18)-dien-28-oate"

"methyl 2α,3α,24-trihydroxyursa-12,20(30)-dien-28-oate"

"methyl 2α,3α-dihydroxyursa-12,20(30)-dien-28-oate"

"methyl 2α,3α-dihydroxyursa-12-en-28-oate"

methyl 2α-hydroxyursa-28-oate

"methyl(12r,13s)2α,3α,24-trihydroxy-12,13-cyclo-taraxer-14-en-28-oate"

"methyl(13s,14r)2α,3α,24-trihydroxy-13,14-cyclo-oleana-11-en-28-oate"

methyl-2alpha

"methyl-2alpha, 3alpha, 28-trihydroxyuran-12-en-28-oate"

methyl-2alpha-hydroxyursoate

"olean-12-en-3β,28-diol"

p. asiatic naki

p. hsipida benth.

prunellin

"urs-12-en-3β,28-diol"

"(-)-guaia-1(10),11-dien-15-al"

"2',4'-dihydroxy-7-methoxy-8-prenylflavan"

"2',7-dihydroxy-4'-methoxy-8-prenylflavan2',7-di-o-尾-d-glucopyranoside"

albanol

"alpha, beta-hexenal"

benzyl alcoholo-尾-d-glucopyranoside

campesteryl ferulate

folicacid

folinicacid

isobutyricacid

isobutyrylmaeelotochromene

isovalericacid

iupeol

pentanicacid

scopeletin

尾-sitosterol-尾-d-glucoside

apigenin-7-o-glucoside

apigenin-7-o-β-d-(4''-caffeoyl)glucuronide

decahydro naphthalene

"n-isobutyl-2e,4 e,10 e,12z-tetradecatetraen-8-ynamide"

"n-isobutyl-2e,4 e,12e-tetradecatrien-8,10-di-ynamide"

"n-isobutyl-2e,4 e,12z-tetradecatrien-8,10-di-ynamide"

"(3r)-2', 3'-dihydroxy-7, 4'-dimethoxyisoflavone"

"(6ar, 11ar)-10-hydroxy-3, 9-dimethoxypterocarpane"

(?)-medicarpin

"2', 4'-dihydroxy-5"

2'-hydroxy-3

"2'-hydroxy-3',4'-dime thoxy-isoflavane-7-o-β-d-glucoside"

"20(r)-21,24-cyclo-3beta,25-dihydroxyl-dammar-23(24)-en-21-one"

3'-hydroxy-4--methoxyisoflavone-7-o-beta-d-glucoside

3-o-beta-d-glucuronopyranosyl gypsogenin

4'-dimethoxyisoflavane-7-o-beta-d-glucoside

"4-hydroxy-2,6-dimethyl-6-(3,7-dimethyl-2,6-octadienyl)-8-(3-methyl-2-butenyl)-2h-1-benzopyran-5,7(3h,6h)-dione"

6-dimethoxy-isoflavane

"9, 10-dimethoxypterocarpane-3-o-beta-d-glucoside"

"9,10-dimethoxy-pterocarpane-3-o-β-d-gluco-side"

acetylastragaloside

astragaloside 1~8

astramembrannin

cyclosieversigenin

formononentin

glucuronicacid

hexadecanoicacid

"isoastragaloside1, 3"

kumugansine a

n-candicine

βetaine

asperglaucide

Anhydroicaritin

Anemarsaponin F_qt

Timosaponin B III_qt

Icariin I

Anemarsaponin C_qt

Anemarsaponin E_qt

(Z)-3-(4-hydroxy-3-methoxy-phenyl)-N-[2-(4-hydroxyphenyl)ethyl]acrylamide

"poriferasta-7,22E-dien-3beta-ol"

28-norolean-17-en-3-ol

"bidentatoside,ii_qt"

β-ecdysterone

berberine

coptisine

wogonin

delta 7-stigmastenol

baicalein

Baicalin

epiberberine

Inophyllum E

Spinasterol

palmatine

beta-daucosterol_qt

Alloisoimperatorin

Ammidin

Cnidilin

3'-Methoxydaidzein

"Daidzein-4,7-diglucoside"

Vulgaxanthin-I

poriferasterol monoglucoside_qt

stigmast-7-enol

poriferast-5-en-3beta-ol

beta-carotene

FA

arachidonic acid

Supraene

Iristectorigenin A

"icosa-11,14,17-trienoic acid methyl ester"

Norartocarpetin

Linolenic acid ethyl ester

Tetramethoxyluteolin

Skimmin (8CI)

(24r)-saringosterol

"[(1S,5S,7S)-7-acetoxy-5-isopropenyl-2,8-dimethylene-cyclodecyl] acetate"

Linarin

Chryseriol

"5,7-dihydroxy-2-(3-hydroxy-4-methoxyphenyl)chroman-4-one"

EUPATORIN

24-Ethylcholest-4-en-3-one

Diosmetin

naringenin

Artemetin

Cynarin(e)

Truflex OBP

Mairin

Jaranol

hederagenin

"(3S,8S,9S,10R,13R,14S,17R)-10,13-dimethyl-17-[(2R,5S)-5-propan-2-yloctan-2-yl]-2,3,4,7,8,9,11,12,14,15,16,17-dodecahydro-1H-cyclopenta[a]phenanthren-3-ol"

"icosa-1,14,17-trienoic acid methyl ester"

"3,9-di-O-methylnissolin"

"5'-hydroxyiso-muronulatol-2',5'-di-O-glucoside"

7-O-methylisomucronulatol

"9,10-dimethoxypterocarpan-3-O-β-D-glucoside"

"(6aR,11aR)-9,10-dimethoxy-6a,11a-dihydro-6H-benzofurano[3,2-c]chromen-3-ol"

Bifendate

isoflavanone

"(3R)-3-(2-hydroxy-3,4-dimethoxyphenyl)chroman-7-ol"

"isomucronulatol-7,2'-di-O-glucosiole"

"1,7-Dihydroxy-3,9-dimethoxy pterocarpene"
