# Supplementary material for: Decoding the Mechanism of CheReCunJin Formula in Treating Sjögren's Syndrome Based on Network Pharmacology and Molecular Docking
Source: Evid Based Complement Alternat Med. 2022 Sep 20;2022:1193846. doi: 10.1155/2022/1193846 (PMC9553462; doi:10.1155/2022/1193846)
Supplement: Supplementary Materials — Table S1: the active ingredients of CRCJ. Table S2: the nodes and edges of the network. Table S3: summary table of drug targets. Table S4: summary table of disease targets. Table S5: network cluster results. [file 1193846.f1.zip › 1193846.f1/Supplementary Table 2.docx]

Supplementary Table 2

#node1 node2 node1_string_id node2_string_id neighborhood_on_chromosome gene_fusion phylogenetic_cooccurrence homology coexpression experimentally_determined_interaction database_annotated automated_textmining combined_score

ABCA1 CETP 9606.ENSP00000363868 9606.ENSP00000200676 0 0 0 0 0 0 0 0.847 0.847

ABCA1 PON1 9606.ENSP00000363868 9606.ENSP00000222381 0 0 0 0 0 0 0 0.616 0.616

ABCA1 MPO 9606.ENSP00000363868 9606.ENSP00000225275 0 0 0 0 0 0 0 0.475 0.475

ABCA1 CCL2 9606.ENSP00000363868 9606.ENSP00000225831 0 0 0 0 0.085 0 0 0.529 0.55

ABCA1 APOB 9606.ENSP00000363868 9606.ENSP00000233242 0 0 0 0 0.06 0 0 0.821 0.824

ABCA1 APOA1 9606.ENSP00000363868 9606.ENSP00000236850 0 0 0 0 0.062 0.686 0.8 0.991 0.999

ABCA1 APOE 9606.ENSP00000363868 9606.ENSP00000252486 0 0 0 0 0.063 0 0 0.983 0.983

ABCA1 NR1H2 9606.ENSP00000363868 9606.ENSP00000253727 0 0 0 0 0.062 0.681 0.8 0.838 0.989

ABCA1 CRP 9606.ENSP00000363868 9606.ENSP00000255030 0 0 0 0 0.062 0 0 0.47 0.481

ABCA1 IL1B 9606.ENSP00000363868 9606.ENSP00000263341 0 0 0 0 0.066 0 0 0.56 0.572

ABCA1 ICAM1 9606.ENSP00000363868 9606.ENSP00000264832 0 0 0 0 0 0 0 0.462 0.462

ABCA1 PPARG 9606.ENSP00000363868 9606.ENSP00000287820 0 0 0 0 0 0.06 0 0.725 0.73

ABCA1 HMGCR 9606.ENSP00000363868 9606.ENSP00000287936 0 0 0 0 0.062 0 0 0.784 0.79

ABCA1 VCAM1 9606.ENSP00000363868 9606.ENSP00000294728 0 0 0 0 0.092 0 0 0.546 0.57

ABCA1 ALB 9606.ENSP00000363868 9606.ENSP00000295897 0 0 0 0 0.062 0 0 0.56 0.57

ABCA1 NOS3 9606.ENSP00000363868 9606.ENSP00000297494 0 0 0 0 0.062 0.169 0 0.471 0.552

ABCA1 LPL 9606.ENSP00000363868 9606.ENSP00000309757 0 0 0 0 0.058 0 0 0.683 0.688

ABCA1 PPARD 9606.ENSP00000363868 9606.ENSP00000310928 0 0 0 0 0.06 0.06 0 0.428 0.45

ABCA1 LEP 9606.ENSP00000363868 9606.ENSP00000312652 0 0 0 0 0 0 0 0.463 0.463

ABCA1 SNCA 9606.ENSP00000363868 9606.ENSP00000338345 0 0 0 0 0 0 0 0.448 0.448

ABCA1 SREBF1 9606.ENSP00000363868 9606.ENSP00000348069 0 0 0 0 0.062 0.068 0 0.778 0.789

ABCA1 MMP9 9606.ENSP00000363868 9606.ENSP00000361405 0 0 0 0 0.062 0 0 0.392 0.405

ABCA1 TLR4 9606.ENSP00000363868 9606.ENSP00000363089 0 0 0 0 0.107 0.056 0 0.521 0.561

ABCA1 IL10 9606.ENSP00000363868 9606.ENSP00000412237 0 0 0 0 0 0 0 0.45 0.45

ABCA1 ADIPOQ 9606.ENSP00000363868 9606.ENSP00000389814 0 0 0 0 0.062 0 0 0.467 0.478

ABCA1 AKT1 9606.ENSP00000363868 9606.ENSP00000451828 0 0 0 0 0.062 0.057 0 0.471 0.491

ABCA1 MTTP 9606.ENSP00000363868 9606.ENSP00000427679 0 0 0 0 0.063 0 0 0.571 0.581

ABCA1 IL6 9606.ENSP00000363868 9606.ENSP00000385675 0 0 0 0 0.058 0 0 0.594 0.601

ABCA1 INS 9606.ENSP00000363868 9606.ENSP00000380432 0 0 0 0 0 0 0 0.602 0.602

ABCA1 TNF 9606.ENSP00000363868 9606.ENSP00000398698 0 0 0 0 0 0 0 0.607 0.607

ABCB1 PON1 9606.ENSP00000478255 9606.ENSP00000222381 0 0 0 0 0.063 0 0 0.51 0.521

ABCB1 NR3C1 9606.ENSP00000478255 9606.ENSP00000231509 0 0 0 0 0 0.056 0 0.391 0.4

ABCB1 APOE 9606.ENSP00000478255 9606.ENSP00000252486 0 0 0 0 0.062 0 0 0.391 0.404

ABCB1 CYP2C9 9606.ENSP00000478255 9606.ENSP00000260682 0 0 0 0 0.062 0 0 0.713 0.719

ABCB1 SLC6A4 9606.ENSP00000478255 9606.ENSP00000261707 0 0 0 0 0 0 0 0.457 0.457

ABCB1 EGF 9606.ENSP00000478255 9606.ENSP00000265171 0 0 0 0 0 0 0 0.549 0.55

ABCB1 TP53 9606.ENSP00000478255 9606.ENSP00000269305 0 0 0 0 0 0 0.9 0.616 0.96

ABCB1 ERBB2 9606.ENSP00000478255 9606.ENSP00000269571 0 0 0 0 0.062 0 0 0.558 0.568

ABCB1 EGFR 9606.ENSP00000478255 9606.ENSP00000275493 0 0 0 0 0.062 0 0 0.687 0.694

ABCB1 HMGCR 9606.ENSP00000478255 9606.ENSP00000287936 0 0 0 0 0 0.126 0 0.51 0.554

ABCB1 KIT 9606.ENSP00000478255 9606.ENSP00000288135 0 0 0 0 0.062 0 0 0.457 0.468

ABCB1 ALB 9606.ENSP00000478255 9606.ENSP00000295897 0 0 0 0 0.062 0 0 0.587 0.596

ABCB1 BCL2L1 9606.ENSP00000478255 9606.ENSP00000302564 0 0 0 0 0 0 0 0.501 0.501

ABCB1 CASP3 9606.ENSP00000478255 9606.ENSP00000311032 0 0 0 0 0 0 0 0.566 0.566

ABCB1 GSTM1 9606.ENSP00000478255 9606.ENSP00000311469 0 0 0 0 0 0 0 0.505 0.505

ABCB1 CASP9 9606.ENSP00000478255 9606.ENSP00000330237 0 0 0 0 0 0 0 0.414 0.414

ABCB1 CYP3A4 9606.ENSP00000478255 9606.ENSP00000337915 0 0 0 0 0.083 0 0 0.962 0.964

ABCB1 CYP1A2 9606.ENSP00000478255 9606.ENSP00000342007 0 0 0 0 0 0 0 0.64 0.64

ABCB1 OCLN 9606.ENSP00000478255 9606.ENSP00000347379 0 0 0 0 0.062 0 0 0.73 0.736

ABCB1 TOP1 9606.ENSP00000478255 9606.ENSP00000354522 0 0 0 0 0.062 0.059 0 0.518 0.537

ABCB1 MTOR 9606.ENSP00000478255 9606.ENSP00000354558 0 0 0 0 0.062 0.056 0 0.45 0.471

ABCB1 NR1I3 9606.ENSP00000478255 9606.ENSP00000356959 0 0 0 0 0.062 0.056 0 0.607 0.621

ABCB1 JUN 9606.ENSP00000478255 9606.ENSP00000360266 0 0 0 0 0 0.058 0 0.409 0.419

ABCB1 PTEN 9606.ENSP00000478255 9606.ENSP00000361021 0 0 0 0 0.062 0.061 0 0.467 0.49

ABCB1 IL6 9606.ENSP00000478255 9606.ENSP00000385675 0 0 0 0 0 0 0 0.419 0.419

ABCB1 TNF 9606.ENSP00000478255 9606.ENSP00000398698 0 0 0 0 0 0 0 0.455 0.455

ABCB1 ESR1 9606.ENSP00000478255 9606.ENSP00000405330 0 0 0 0 0 0.056 0 0.47 0.478

ABCB1 BRCA1 9606.ENSP00000478255 9606.ENSP00000418960 0 0 0 0 0 0 0 0.424 0.424

ABCB1 HTR2A 9606.ENSP00000478255 9606.ENSP00000437737 0 0 0 0 0 0 0 0.402 0.402

ABCB1 HIF1A 9606.ENSP00000478255 9606.ENSP00000437955 0 0 0 0 0.054 0 0 0.598 0.603

ABCB1 AKT1 9606.ENSP00000478255 9606.ENSP00000451828 0 0 0 0 0.062 0.06 0 0.62 0.636

ABCB1 CYP1B1 9606.ENSP00000478255 9606.ENSP00000478561 0 0 0 0 0.056 0 0 0.473 0.481

ABCB1 VEGFA 9606.ENSP00000478255 9606.ENSP00000478570 0 0 0 0 0.062 0 0 0.505 0.515

ABHD5 PPARG 9606.ENSP00000390849 9606.ENSP00000287820 0 0 0 0 0.062 0.058 0 0.518 0.537

ABHD5 LPL 9606.ENSP00000390849 9606.ENSP00000309757 0 0 0 0 0 0 0 0.471 0.471

ABHD5 SREBF1 9606.ENSP00000390849 9606.ENSP00000348069 0 0 0 0 0 0 0 0.467 0.467

ABHD5 INS 9606.ENSP00000390849 9606.ENSP00000380432 0 0 0 0 0 0 0 0.52 0.52

ACE AQP2 9606.ENSP00000290866 9606.ENSP00000199280 0 0 0 0 0 0 0 0.418 0.418

ACE CETP 9606.ENSP00000290866 9606.ENSP00000200676 0 0 0 0 0.062 0 0 0.5 0.51

ACE HMOX1 9606.ENSP00000290866 9606.ENSP00000216117 0 0 0 0 0 0 0 0.457 0.457

ACE CTSG 9606.ENSP00000290866 9606.ENSP00000216336 0 0 0 0 0 0 0 0.52 0.52

ACE MMP2 9606.ENSP00000290866 9606.ENSP00000219070 0 0 0 0 0.062 0 0 0.545 0.554

ACE TGFB1 9606.ENSP00000290866 9606.ENSP00000221930 0 0 0 0 0 0 0 0.635 0.635

ACE PON1 9606.ENSP00000290866 9606.ENSP00000222381 0 0 0 0 0.057 0 0 0.518 0.526

ACE MPO 9606.ENSP00000290866 9606.ENSP00000225275 0 0 0 0 0.063 0 0 0.523 0.534

ACE CCL2 9606.ENSP00000290866 9606.ENSP00000225831 0 0 0 0 0 0 0 0.609 0.609

ACE IFNG 9606.ENSP00000290866 9606.ENSP00000229135 0 0 0 0 0 0 0 0.467 0.467

ACE APOB 9606.ENSP00000290866 9606.ENSP00000233242 0 0 0 0 0.063 0 0 0.609 0.617

ACE CTSD 9606.ENSP00000290866 9606.ENSP00000236671 0 0 0 0 0 0 0 0.483 0.483

ACE APOA1 9606.ENSP00000290866 9606.ENSP00000236850 0 0 0 0 0.062 0 0 0.519 0.529

ACE FGF23 9606.ENSP00000290866 9606.ENSP00000237837 0 0 0 0 0 0 0 0.518 0.518

ACE CAT 9606.ENSP00000290866 9606.ENSP00000241052 0 0 0 0 0 0 0 0.601 0.601

ACE C3 9606.ENSP00000290866 9606.ENSP00000245907 0 0 0 0 0.071 0 0 0.393 0.412

ACE APOE 9606.ENSP00000290866 9606.ENSP00000252486 0 0 0 0 0.064 0 0 0.72 0.726

ACE CRP 9606.ENSP00000290866 9606.ENSP00000255030 0 0 0 0 0 0 0 0.855 0.855

ACE CYP2C9 9606.ENSP00000290866 9606.ENSP00000260682 0 0 0 0 0 0.056 0 0.414 0.423

ACE SLC6A4 9606.ENSP00000290866 9606.ENSP00000261707 0 0 0 0 0 0 0 0.423 0.422

ACE IL1B 9606.ENSP00000290866 9606.ENSP00000263341 0 0 0 0 0 0 0 0.602 0.602

ACE SELP 9606.ENSP00000290866 9606.ENSP00000263686 0 0 0 0 0 0 0 0.476 0.476

ACE FGF2 9606.ENSP00000290866 9606.ENSP00000264498 0 0 0 0 0 0 0 0.425 0.425

ACE ICAM1 9606.ENSP00000290866 9606.ENSP00000264832 0 0 0 0 0 0 0 0.61 0.61

ACE EGF 9606.ENSP00000290866 9606.ENSP00000265171 0 0 0 0 0.073 0 0 0.67 0.681

ACE TP53 9606.ENSP00000290866 9606.ENSP00000269305 0 0 0 0 0.063 0 0 0.393 0.407

ACE ERBB2 9606.ENSP00000290866 9606.ENSP00000269571 0 0 0 0 0.062 0 0 0.409 0.422

ACE REN 9606.ENSP00000290866 9606.ENSP00000272190 0 0 0 0 0 0 0 0.967 0.967

ACE EGFR 9606.ENSP00000290866 9606.ENSP00000275493 0 0 0 0 0 0 0 0.429 0.429

ACE CRH 9606.ENSP00000290866 9606.ENSP00000276571 0 0 0 0 0 0 0 0.544 0.544

ACE SST 9606.ENSP00000290866 9606.ENSP00000287641 0 0 0 0 0 0 0 0.419 0.418

ACE PPARG 9606.ENSP00000290866 9606.ENSP00000287820 0 0 0 0 0.062 0 0 0.78 0.785

ACE HMGCR 9606.ENSP00000290866 9606.ENSP00000287936 0 0 0 0 0 0 0 0.565 0.565

ACE NCF1 9606.ENSP00000290866 9606.ENSP00000289473 0 0 0 0 0 0 0 0.505 0.505

ACE MTOR 9606.ENSP00000290866 9606.ENSP00000354558 0 0 0 0 0 0 0 0.405 0.405

ACE LPL 9606.ENSP00000290866 9606.ENSP00000309757 0 0 0 0 0 0 0 0.406 0.406

ACE HIF1A 9606.ENSP00000290866 9606.ENSP00000437955 0 0 0 0 0.049 0 0 0.407 0.411

ACE COLQ 9606.ENSP00000290866 9606.ENSP00000373298 0 0 0 0 0.049 0 0 0.414 0.419

ACE TLR4 9606.ENSP00000290866 9606.ENSP00000363089 0 0 0 0 0 0 0 0.43 0.43

ACE CASP3 9606.ENSP00000290866 9606.ENSP00000311032 0 0 0 0 0 0 0 0.442 0.442

ACE MMP1 9606.ENSP00000290866 9606.ENSP00000322788 0 0 0 0 0.062 0 0 0.432 0.445

ACE JUN 9606.ENSP00000290866 9606.ENSP00000360266 0 0 0 0 0 0 0 0.447 0.447

ACE ESR1 9606.ENSP00000290866 9606.ENSP00000405330 0 0 0 0 0.062 0 0 0.456 0.467

ACE THBD 9606.ENSP00000290866 9606.ENSP00000366307 0 0 0 0 0.062 0 0 0.457 0.469

ACE CXCL8 9606.ENSP00000290866 9606.ENSP00000306512 0 0 0 0 0 0 0 0.47 0.47

ACE VDR 9606.ENSP00000290866 9606.ENSP00000447173 0 0 0 0 0 0 0 0.471 0.471

ACE CALCA 9606.ENSP00000290866 9606.ENSP00000331746 0 0 0 0 0 0 0 0.475 0.475

ACE SPP1 9606.ENSP00000290866 9606.ENSP00000378517 0 0 0 0 0 0 0 0.479 0.479

ACE MMP3 9606.ENSP00000290866 9606.ENSP00000299855 0 0 0 0 0.062 0 0 0.473 0.484

ACE NOS1 9606.ENSP00000290866 9606.ENSP00000477999 0 0 0 0 0.062 0 0 0.499 0.51

ACE CYP3A4 9606.ENSP00000290866 9606.ENSP00000337915 0 0 0 0 0.064 0.056 0 0.505 0.524

ACE SELE 9606.ENSP00000290866 9606.ENSP00000331736 0 0 0 0 0 0 0 0.528 0.528

ACE LCN2 9606.ENSP00000290866 9606.ENSP00000362108 0 0 0 0 0.062 0 0 0.517 0.528

ACE IL10 9606.ENSP00000290866 9606.ENSP00000412237 0 0 0 0 0 0 0 0.53 0.53

ACE IGF1 9606.ENSP00000290866 9606.ENSP00000302665 0 0 0 0 0 0 0 0.566 0.566

ACE AKT1 9606.ENSP00000290866 9606.ENSP00000451828 0 0 0 0 0 0 0 0.577 0.577

ACE EDNRA 9606.ENSP00000290866 9606.ENSP00000315011 0 0 0 0 0.062 0 0 0.573 0.583

ACE VCAM1 9606.ENSP00000290866 9606.ENSP00000294728 0 0 0 0 0.062 0 0 0.593 0.602

ACE F2 9606.ENSP00000290866 9606.ENSP00000308541 0 0 0 0 0.054 0 0 0.608 0.613

ACE GPT 9606.ENSP00000290866 9606.ENSP00000378408 0 0 0 0 0 0 0 0.617 0.617

ACE MMP9 9606.ENSP00000290866 9606.ENSP00000361405 0 0 0 0 0.084 0 0 0.602 0.621

ACE ADIPOQ 9606.ENSP00000290866 9606.ENSP00000389814 0 0 0 0 0.066 0 0 0.621 0.631

ACE LEP 9606.ENSP00000290866 9606.ENSP00000312652 0 0 0 0 0.062 0 0 0.625 0.634

ACE ADAM17 9606.ENSP00000290866 9606.ENSP00000309968 0 0 0 0 0 0 0 0.666 0.666

ACE VEGFA 9606.ENSP00000290866 9606.ENSP00000478570 0 0 0 0 0.065 0 0 0.681 0.688

ACE IL6 9606.ENSP00000290866 9606.ENSP00000385675 0 0 0 0 0 0 0 0.706 0.706

ACE TNF 9606.ENSP00000290866 9606.ENSP00000398698 0 0 0 0 0 0 0 0.707 0.707

ACE NOS3 9606.ENSP00000290866 9606.ENSP00000297494 0 0 0 0 0.063 0 0 0.8 0.804

ACE EDN1 9606.ENSP00000290866 9606.ENSP00000368683 0 0 0 0 0 0 0 0.804 0.804

ACE INS 9606.ENSP00000290866 9606.ENSP00000380432 0 0 0 0 0 0 0 0.858 0.858

ACE ALB 9606.ENSP00000290866 9606.ENSP00000295897 0 0 0 0 0 0 0 0.859 0.859

ACE NR3C2 9606.ENSP00000290866 9606.ENSP00000350815 0 0 0 0 0.062 0 0 0.86 0.863

ACE AGTR1 9606.ENSP00000290866 9606.ENSP00000419422 0 0 0 0 0.062 0 0 0.922 0.924

ACE AGT 9606.ENSP00000290866 9606.ENSP00000355627 0 0 0 0 0.062 0.822 0.9 0.94 0.998

ACE2 CCL2 9606.ENSP00000389326 9606.ENSP00000225831 0 0 0 0 0 0 0 0.435 0.435

ACE2 CAT 9606.ENSP00000389326 9606.ENSP00000241052 0 0 0 0 0 0.272 0 0.306 0.473

ACE2 APOE 9606.ENSP00000389326 9606.ENSP00000252486 0 0 0 0 0.082 0 0 0.408 0.434

ACE2 CRP 9606.ENSP00000389326 9606.ENSP00000255030 0 0 0 0 0 0 0 0.48 0.48

ACE2 IL1B 9606.ENSP00000389326 9606.ENSP00000263341 0 0 0 0 0 0 0 0.432 0.432

ACE2 REN 9606.ENSP00000389326 9606.ENSP00000272190 0 0 0 0 0.062 0 0 0.965 0.966

ACE2 VCAM1 9606.ENSP00000389326 9606.ENSP00000294728 0 0 0 0 0.052 0 0 0.403 0.41

ACE2 ALB 9606.ENSP00000389326 9606.ENSP00000295897 0 0 0 0 0 0.27 0 0.557 0.663

ACE2 NOS3 9606.ENSP00000389326 9606.ENSP00000297494 0 0 0 0 0.062 0 0 0.581 0.59

ACE2 ADAM17 9606.ENSP00000389326 9606.ENSP00000309968 0 0 0 0 0 0 0 0.606 0.606

ACE2 NR3C2 9606.ENSP00000389326 9606.ENSP00000350815 0 0 0 0 0.062 0 0 0.435 0.447

ACE2 AGT 9606.ENSP00000389326 9606.ENSP00000355627 0 0 0 0 0.063 0.845 0.9 0.878 0.998

ACE2 MMP9 9606.ENSP00000389326 9606.ENSP00000361405 0 0 0 0 0.062 0 0 0.423 0.436

ACE2 EDN1 9606.ENSP00000389326 9606.ENSP00000368683 0 0 0 0 0 0 0 0.671 0.671

ACE2 INS 9606.ENSP00000389326 9606.ENSP00000380432 0 0 0 0 0 0 0 0.579 0.579

ACE2 IL6 9606.ENSP00000389326 9606.ENSP00000385675 0 0 0 0 0 0 0 0.516 0.516

ACE2 NOS1 9606.ENSP00000389326 9606.ENSP00000477999 0 0 0 0 0 0 0 0.427 0.427

ACE2 AKT1 9606.ENSP00000389326 9606.ENSP00000451828 0 0 0 0 0 0 0 0.443 0.443

ACE2 TNF 9606.ENSP00000389326 9606.ENSP00000398698 0 0 0 0 0 0 0 0.471 0.471

ACE2 FURIN 9606.ENSP00000389326 9606.ENSP00000483552 0 0 0 0 0 0 0 0.525 0.525

ACE2 AGTR1 9606.ENSP00000389326 9606.ENSP00000419422 0 0 0 0 0.062 0 0 0.865 0.868

ACP5 NFKBIA 9606.ENSP00000468767 9606.ENSP00000216797 0 0 0 0 0.092 0.056 0 0.502 0.536

ACP5 COL1A1 9606.ENSP00000468767 9606.ENSP00000225964 0 0 0 0 0 0 0 0.51 0.51

ACP5 FGF23 9606.ENSP00000468767 9606.ENSP00000237837 0 0 0 0 0 0 0 0.556 0.556

ACP5 IL1A 9606.ENSP00000468767 9606.ENSP00000263339 0 0 0 0 0.062 0 0 0.391 0.404

ACP5 IL1B 9606.ENSP00000468767 9606.ENSP00000263341 0 0 0 0 0.105 0 0 0.559 0.588

ACP5 CA2 9606.ENSP00000468767 9606.ENSP00000285379 0 0 0 0 0.062 0 0 0.654 0.661

ACP5 ALB 9606.ENSP00000468767 9606.ENSP00000295897 0 0 0 0 0.064 0 0 0.59 0.6

ACP5 IGF1 9606.ENSP00000468767 9606.ENSP00000302665 0 0 0 0 0.062 0 0 0.505 0.515

ACP5 CALCA 9606.ENSP00000468767 9606.ENSP00000331746 0 0 0 0 0 0 0 0.662 0.662

ACP5 IL17A 9606.ENSP00000468767 9606.ENSP00000344192 0 0 0 0 0 0 0 0.459 0.459

ACP5 BGLAP 9606.ENSP00000468767 9606.ENSP00000357255 0 0 0 0 0 0 0 0.871 0.871

ACP5 JUN 9606.ENSP00000468767 9606.ENSP00000360266 0 0 0 0 0 0 0 0.503 0.503

ACP5 MMP9 9606.ENSP00000468767 9606.ENSP00000361405 0 0 0 0 0.214 0 0 0.681 0.738

ACP5 SPP1 9606.ENSP00000468767 9606.ENSP00000378517 0 0 0 0 0.062 0.296 0 0.683 0.772

ACP5 INS 9606.ENSP00000468767 9606.ENSP00000380432 0 0 0 0 0 0 0 0.404 0.404

ACP5 IL6 9606.ENSP00000468767 9606.ENSP00000385675 0 0 0 0 0 0 0 0.603 0.603

ACP5 TNF 9606.ENSP00000468767 9606.ENSP00000398698 0 0 0 0 0.109 0 0 0.594 0.622

ACP5 ESR1 9606.ENSP00000468767 9606.ENSP00000405330 0 0 0 0 0.065 0 0 0.428 0.442

ACP5 AKT1 9606.ENSP00000468767 9606.ENSP00000451828 0 0 0 0 0.059 0.061 0 0.467 0.488

ACP5 VEGFA 9606.ENSP00000468767 9606.ENSP00000478570 0 0 0 0 0 0 0 0.452 0.452

ACSL4 APOB 9606.ENSP00000339787 9606.ENSP00000233242 0 0 0 0 0.053 0.292 0 0.196 0.413

ACSL4 HMGCR 9606.ENSP00000339787 9606.ENSP00000287936 0 0 0 0 0.082 0.105 0 0.365 0.433

ACSL4 LPL 9606.ENSP00000339787 9606.ENSP00000309757 0 0 0 0 0.062 0 0.8 0.222 0.841

ADA IL2 9606.ENSP00000361965 9606.ENSP00000226730 0 0 0 0 0 0 0 0.414 0.414

ADA IFNG 9606.ENSP00000361965 9606.ENSP00000229135 0 0 0 0 0 0 0 0.46 0.459

ADA CRP 9606.ENSP00000361965 9606.ENSP00000255030 0 0 0 0 0 0 0 0.52 0.52

ADA ALB 9606.ENSP00000361965 9606.ENSP00000295897 0 0 0 0 0.077 0.145 0 0.556 0.619

ADA HPRT1 9606.ENSP00000361965 9606.ENSP00000298556 0.08 0 0 0 0.146 0 0 0.61 0.667

ADA TOP1 9606.ENSP00000361965 9606.ENSP00000354522 0 0 0 0 0 0 0 0.642 0.642

ADA IL6 9606.ENSP00000361965 9606.ENSP00000385675 0 0 0 0 0 0 0 0.414 0.414

ADA GPT 9606.ENSP00000361965 9606.ENSP00000378408 0.045 0 0 0 0 0 0 0.415 0.418

ADA TNF 9606.ENSP00000361965 9606.ENSP00000398698 0 0 0 0 0 0 0 0.468 0.468

ADA INS 9606.ENSP00000361965 9606.ENSP00000380432 0 0 0 0 0 0 0 0.47 0.47

ADA XDH 9606.ENSP00000361965 9606.ENSP00000368727 0.081 0 0 0 0.062 0 0 0.44 0.475

ADAM17 TNFRSF1A 9606.ENSP00000309968 9606.ENSP00000162749 0 0 0 0 0 0.27 0 0.768 0.824

ADAM17 MMP2 9606.ENSP00000309968 9606.ENSP00000219070 0 0 0 0 0 0 0 0.518 0.518

ADAM17 CCL2 9606.ENSP00000309968 9606.ENSP00000225831 0 0 0 0 0 0 0 0.426 0.426

ADAM17 NOTCH2 9606.ENSP00000309968 9606.ENSP00000256646 0 0 0 0 0.062 0.148 0.8 0.481 0.906

ADAM17 IL1B 9606.ENSP00000309968 9606.ENSP00000263341 0 0 0 0 0.06 0 0 0.519 0.528

ADAM17 ICAM1 9606.ENSP00000309968 9606.ENSP00000264832 0 0 0 0 0 0 0 0.46 0.459

ADAM17 EGF 9606.ENSP00000309968 9606.ENSP00000265171 0 0 0 0 0 0 0.9 0.659 0.964

ADAM17 ERBB3 9606.ENSP00000309968 9606.ENSP00000267101 0 0 0 0 0 0 0 0.482 0.482

ADAM17 ERBB2 9606.ENSP00000309968 9606.ENSP00000269571 0 0 0 0 0.049 0 0.9 0.506 0.948

ADAM17 REN 9606.ENSP00000309968 9606.ENSP00000272190 0 0 0 0 0.047 0 0 0.539 0.542

ADAM17 EGFR 9606.ENSP00000309968 9606.ENSP00000275493 0 0 0 0 0.062 0 0 0.948 0.949

ADAM17 VCAM1 9606.ENSP00000309968 9606.ENSP00000294728 0 0 0 0 0.049 0 0 0.462 0.466

ADAM17 ALB 9606.ENSP00000309968 9606.ENSP00000295897 0 0 0 0 0 0 0 0.437 0.437

ADAM17 MMP3 9606.ENSP00000309968 9606.ENSP00000299855 0 0 0 0 0 0 0 0.535 0.535

ADAM17 PRKCB 9606.ENSP00000309968 9606.ENSP00000305355 0 0 0 0 0 0 0.9 0.063 0.902

ADAM17 CXCL8 9606.ENSP00000309968 9606.ENSP00000306512 0 0 0 0 0 0 0 0.469 0.469

ADAM17 MMP1 9606.ENSP00000309968 9606.ENSP00000322788 0 0 0 0 0 0 0 0.428 0.428

ADAM17 TLR4 9606.ENSP00000309968 9606.ENSP00000363089 0 0 0 0 0.062 0.057 0 0.407 0.429

ADAM17 IL10 9606.ENSP00000309968 9606.ENSP00000412237 0 0 0 0 0 0 0 0.452 0.452

ADAM17 VEGFA 9606.ENSP00000309968 9606.ENSP00000478570 0 0 0 0 0 0 0 0.459 0.459

ADAM17 P4HB 9606.ENSP00000309968 9606.ENSP00000327801 0 0 0 0 0.062 0.056 0 0.45 0.47

ADAM17 HIF1A 9606.ENSP00000309968 9606.ENSP00000437955 0 0 0 0 0.062 0 0 0.486 0.497

ADAM17 AKT1 9606.ENSP00000309968 9606.ENSP00000451828 0 0 0 0 0.052 0 0 0.543 0.548

ADAM17 CX3CR1 9606.ENSP00000309968 9606.ENSP00000351059 0 0 0 0 0 0 0 0.607 0.608

ADAM17 MMP9 9606.ENSP00000309968 9606.ENSP00000361405 0 0 0 0 0 0 0 0.618 0.618

ADAM17 IL6 9606.ENSP00000309968 9606.ENSP00000385675 0 0 0 0 0 0 0 0.724 0.724

ADAM17 BDNF 9606.ENSP00000309968 9606.ENSP00000414303 0 0 0 0 0 0 0.9 0.276 0.924

ADAM17 TNFRSF1B 9606.ENSP00000309968 9606.ENSP00000365435 0 0 0 0 0.062 0.27 0.9 0.544 0.964

ADAM17 FURIN 9606.ENSP00000309968 9606.ENSP00000483552 0 0 0 0 0.064 0 0.9 0.688 0.968

ADAM17 TNF 9606.ENSP00000309968 9606.ENSP00000398698 0 0 0 0 0.062 0.213 0.9 0.847 0.987

ADIPOQ CETP 9606.ENSP00000389814 9606.ENSP00000200676 0 0 0 0 0 0 0 0.468 0.468

ADIPOQ MIF 9606.ENSP00000389814 9606.ENSP00000215754 0 0 0 0 0 0 0 0.425 0.424

ADIPOQ HMOX1 9606.ENSP00000389814 9606.ENSP00000216117 0 0 0 0 0 0 0 0.55 0.55

ADIPOQ MMP2 9606.ENSP00000389814 9606.ENSP00000219070 0 0 0 0 0.062 0 0 0.413 0.425

ADIPOQ TGFB1 9606.ENSP00000389814 9606.ENSP00000221930 0 0 0 0 0 0 0 0.441 0.441

ADIPOQ PON1 9606.ENSP00000389814 9606.ENSP00000222381 0 0 0 0 0 0 0 0.499 0.499

ADIPOQ MPO 9606.ENSP00000389814 9606.ENSP00000225275 0 0 0 0 0 0 0 0.464 0.463

ADIPOQ CCL2 9606.ENSP00000389814 9606.ENSP00000225831 0 0 0 0 0 0 0 0.77 0.77

ADIPOQ IL2 9606.ENSP00000389814 9606.ENSP00000226730 0 0 0 0 0 0 0 0.414 0.414

ADIPOQ IFNG 9606.ENSP00000389814 9606.ENSP00000229135 0 0 0 0 0 0 0 0.505 0.505

ADIPOQ IL4 9606.ENSP00000389814 9606.ENSP00000231449 0 0 0 0 0 0 0 0.582 0.582

ADIPOQ NR3C1 9606.ENSP00000389814 9606.ENSP00000231509 0 0 0 0 0 0 0.9 0.418 0.939

ADIPOQ APOB 9606.ENSP00000389814 9606.ENSP00000233242 0 0 0 0 0 0 0 0.704 0.704

ADIPOQ APOA1 9606.ENSP00000389814 9606.ENSP00000236850 0 0 0 0 0 0 0 0.681 0.681

ADIPOQ FGF23 9606.ENSP00000389814 9606.ENSP00000237837 0 0 0 0 0 0 0 0.444 0.444

ADIPOQ CAT 9606.ENSP00000389814 9606.ENSP00000241052 0 0 0 0 0 0 0 0.521 0.521

ADIPOQ NR1D1 9606.ENSP00000389814 9606.ENSP00000246672 0 0 0 0 0 0 0 0.436 0.436

ADIPOQ APOE 9606.ENSP00000389814 9606.ENSP00000252486 0 0 0 0 0 0 0 0.658 0.659

ADIPOQ CRP 9606.ENSP00000389814 9606.ENSP00000255030 0 0 0 0 0.062 0 0 0.88 0.882

ADIPOQ IL1RN 9606.ENSP00000389814 9606.ENSP00000259206 0 0 0 0 0 0 0 0.425 0.424

ADIPOQ IL1A 9606.ENSP00000389814 9606.ENSP00000263339 0 0 0 0 0 0 0 0.42 0.42

ADIPOQ IL1B 9606.ENSP00000389814 9606.ENSP00000263341 0 0 0 0 0 0 0 0.715 0.715

ADIPOQ SELP 9606.ENSP00000389814 9606.ENSP00000263686 0 0 0 0 0 0 0 0.455 0.455

ADIPOQ FGF2 9606.ENSP00000389814 9606.ENSP00000264498 0 0 0 0 0.062 0 0 0.78 0.785

ADIPOQ ICAM1 9606.ENSP00000389814 9606.ENSP00000264832 0 0 0 0 0 0 0 0.608 0.608

ADIPOQ EGF 9606.ENSP00000389814 9606.ENSP00000265171 0 0 0 0 0 0 0 0.402 0.402

ADIPOQ TP53 9606.ENSP00000389814 9606.ENSP00000269305 0 0 0 0 0 0 0 0.424 0.424

ADIPOQ REN 9606.ENSP00000389814 9606.ENSP00000272190 0 0 0 0 0 0 0 0.663 0.663

ADIPOQ PPARG 9606.ENSP00000389814 9606.ENSP00000287820 0 0 0 0 0.063 0 0.9 0.894 0.989

ADIPOQ HMGCR 9606.ENSP00000389814 9606.ENSP00000287936 0 0 0 0 0 0 0 0.451 0.451

ADIPOQ VCAM1 9606.ENSP00000389814 9606.ENSP00000294728 0 0 0 0 0.112 0 0 0.629 0.656

ADIPOQ ALB 9606.ENSP00000389814 9606.ENSP00000295897 0 0 0 0 0 0 0 0.717 0.717

ADIPOQ NOS3 9606.ENSP00000389814 9606.ENSP00000297494 0 0 0 0 0.062 0 0 0.681 0.687

ADIPOQ MMP3 9606.ENSP00000389814 9606.ENSP00000299855 0 0 0 0 0.062 0 0 0.406 0.419

ADIPOQ IGF1 9606.ENSP00000389814 9606.ENSP00000302665 0 0 0 0 0.075 0 0 0.747 0.756

ADIPOQ INSR 9606.ENSP00000389814 9606.ENSP00000303830 0 0 0 0 0 0 0 0.52 0.52

ADIPOQ IL13 9606.ENSP00000389814 9606.ENSP00000304915 0 0 0 0 0 0 0 0.43 0.43

ADIPOQ CXCL8 9606.ENSP00000389814 9606.ENSP00000306512 0 0 0 0 0 0 0 0.681 0.681

ADIPOQ LPL 9606.ENSP00000389814 9606.ENSP00000309757 0 0 0 0 0.063 0 0 0.779 0.784

ADIPOQ PPARD 9606.ENSP00000389814 9606.ENSP00000310928 0 0 0 0 0 0 0 0.515 0.515

ADIPOQ CASP3 9606.ENSP00000389814 9606.ENSP00000311032 0 0 0 0 0 0 0 0.459 0.459

ADIPOQ LEP 9606.ENSP00000389814 9606.ENSP00000312652 0 0 0 0 0.145 0 0 0.924 0.932

ADIPOQ SELE 9606.ENSP00000389814 9606.ENSP00000331736 0 0 0 0 0 0 0 0.603 0.603

ADIPOQ IL17A 9606.ENSP00000389814 9606.ENSP00000344192 0 0 0 0 0 0 0 0.46 0.459

ADIPOQ SREBF1 9606.ENSP00000389814 9606.ENSP00000348069 0 0 0 0 0 0 0 0.749 0.749

ADIPOQ MTOR 9606.ENSP00000389814 9606.ENSP00000354558 0 0 0 0 0 0 0 0.536 0.536

ADIPOQ AGT 9606.ENSP00000389814 9606.ENSP00000355627 0 0 0 0 0 0 0 0.652 0.652

ADIPOQ HSD11B1 9606.ENSP00000389814 9606.ENSP00000355995 0 0 0 0 0 0 0 0.61 0.611

ADIPOQ BGLAP 9606.ENSP00000389814 9606.ENSP00000357255 0 0 0 0 0 0 0 0.681 0.681

ADIPOQ CD40LG 9606.ENSP00000389814 9606.ENSP00000359663 0 0 0 0 0 0 0 0.82 0.82

ADIPOQ JUN 9606.ENSP00000389814 9606.ENSP00000360266 0 0 0 0 0 0 0 0.516 0.516

ADIPOQ MMP9 9606.ENSP00000389814 9606.ENSP00000361405 0 0 0 0 0 0 0 0.529 0.529

ADIPOQ LCN2 9606.ENSP00000389814 9606.ENSP00000362108 0 0 0 0 0 0 0 0.56 0.56

ADIPOQ TLR4 9606.ENSP00000389814 9606.ENSP00000363089 0 0 0 0 0.066 0 0 0.573 0.584

ADIPOQ THBD 9606.ENSP00000389814 9606.ENSP00000366307 0 0 0 0 0.062 0 0 0.392 0.406

ADIPOQ EDN1 9606.ENSP00000389814 9606.ENSP00000368683 0 0 0 0 0 0 0 0.558 0.558

ADIPOQ SHBG 9606.ENSP00000389814 9606.ENSP00000369816 0 0 0 0 0.065 0 0 0.717 0.724

ADIPOQ GPT 9606.ENSP00000389814 9606.ENSP00000378408 0 0 0 0 0 0 0 0.713 0.713

ADIPOQ SPP1 9606.ENSP00000389814 9606.ENSP00000378517 0 0 0 0 0 0 0 0.526 0.526

ADIPOQ CYP19A1 9606.ENSP00000389814 9606.ENSP00000379683 0 0 0 0 0 0 0 0.502 0.502

ADIPOQ INS 9606.ENSP00000389814 9606.ENSP00000380432 0 0 0 0 0 0 0 0.922 0.922

ADIPOQ IL6 9606.ENSP00000389814 9606.ENSP00000385675 0 0 0 0 0 0 0 0.872 0.872

ADIPOQ BDNF 9606.ENSP00000389814 9606.ENSP00000414303 0 0 0 0 0 0 0 0.425 0.424

ADIPOQ HIF1A 9606.ENSP00000389814 9606.ENSP00000437955 0 0 0 0 0 0 0 0.468 0.468

ADIPOQ LTA 9606.ENSP00000389814 9606.ENSP00000403495 0 0 0 0 0 0 0 0.473 0.473

ADIPOQ ESR1 9606.ENSP00000389814 9606.ENSP00000405330 0 0 0 0 0 0 0 0.505 0.505

ADIPOQ CCL5 9606.ENSP00000389814 9606.ENSP00000474412 0 0 0 0 0 0 0 0.533 0.533

ADIPOQ AGTR1 9606.ENSP00000389814 9606.ENSP00000419422 0 0 0 0 0 0 0 0.578 0.578

ADIPOQ VEGFA 9606.ENSP00000389814 9606.ENSP00000478570 0 0 0 0 0 0 0 0.612 0.612

ADIPOQ IL10 9606.ENSP00000389814 9606.ENSP00000412237 0 0 0 0 0 0 0 0.689 0.689

ADIPOQ AKT1 9606.ENSP00000389814 9606.ENSP00000451828 0 0 0 0 0 0 0 0.692 0.692

ADIPOQ TNF 9606.ENSP00000389814 9606.ENSP00000398698 0 0 0 0 0 0 0 0.872 0.872

AGT CFTR 9606.ENSP00000355627 9606.ENSP00000003084 0 0 0 0 0 0 0.9 0.147 0.911

AGT APOH 9606.ENSP00000355627 9606.ENSP00000205948 0 0 0 0 0.349 0.057 0 0.277 0.517

AGT CTSG 9606.ENSP00000355627 9606.ENSP00000216336 0 0 0 0 0 0.277 0.9 0.419 0.954

AGT MMP2 9606.ENSP00000355627 9606.ENSP00000219070 0 0 0 0 0.069 0.056 0 0.432 0.457

AGT TGFB1 9606.ENSP00000355627 9606.ENSP00000221930 0 0 0 0 0 0 0 0.601 0.601

AGT CCL2 9606.ENSP00000355627 9606.ENSP00000225831 0 0 0 0 0 0 0 0.498 0.498

AGT APOB 9606.ENSP00000355627 9606.ENSP00000233242 0 0 0 0 0.325 0 0 0.325 0.525

AGT CTSD 9606.ENSP00000355627 9606.ENSP00000236671 0 0 0 0 0 0 0.9 0.384 0.935

AGT APOA1 9606.ENSP00000355627 9606.ENSP00000236850 0 0 0 0 0.34 0.141 0 0.336 0.591

AGT CAT 9606.ENSP00000355627 9606.ENSP00000241052 0 0 0 0 0.066 0 0 0.498 0.512

AGT C3 9606.ENSP00000355627 9606.ENSP00000245907 0 0 0 0 0.179 0 0 0.322 0.419

AGT APOE 9606.ENSP00000355627 9606.ENSP00000252486 0 0 0 0 0.152 0 0 0.613 0.658

AGT CRP 9606.ENSP00000355627 9606.ENSP00000255030 0 0 0 0 0.103 0 0 0.522 0.554

AGT IL1B 9606.ENSP00000355627 9606.ENSP00000263341 0 0 0 0 0 0 0 0.539 0.539

AGT ICAM1 9606.ENSP00000355627 9606.ENSP00000264832 0 0 0 0 0 0 0 0.511 0.511

AGT TP53 9606.ENSP00000355627 9606.ENSP00000269305 0 0 0 0 0 0.192 0 0.343 0.447

AGT REN 9606.ENSP00000355627 9606.ENSP00000272190 0 0 0 0 0.062 0.936 0.9 0.992 0.999

AGT PPARG 9606.ENSP00000355627 9606.ENSP00000287820 0 0 0 0 0.06 0.058 0 0.525 0.542

AGT NCF1 9606.ENSP00000355627 9606.ENSP00000289473 0 0 0 0 0 0 0 0.621 0.621

AGT VCAM1 9606.ENSP00000355627 9606.ENSP00000294728 0 0 0 0 0.088 0 0 0.483 0.508

AGT ALB 9606.ENSP00000355627 9606.ENSP00000295897 0 0 0 0 0.258 0.104 0 0.638 0.738

AGT NOS3 9606.ENSP00000355627 9606.ENSP00000297494 0 0 0 0 0.062 0 0 0.651 0.658

AGT MMP3 9606.ENSP00000355627 9606.ENSP00000299855 0 0 0 0 0 0.056 0 0.42 0.429

AGT IGF1 9606.ENSP00000355627 9606.ENSP00000302665 0 0 0 0 0.062 0 0 0.508 0.519

AGT CHRM1 9606.ENSP00000355627 9606.ENSP00000306490 0 0 0 0 0.127 0 0.9 0 0.908

AGT F2 9606.ENSP00000355627 9606.ENSP00000308541 0 0 0 0 0.375 0.12 0.9 0.405 0.962

AGT LPL 9606.ENSP00000355627 9606.ENSP00000309757 0 0 0 0 0.076 0 0 0.39 0.412

AGT CASP3 9606.ENSP00000355627 9606.ENSP00000311032 0 0 0 0 0.063 0 0 0.397 0.411

AGT LEP 9606.ENSP00000355627 9606.ENSP00000312652 0 0 0 0 0 0 0 0.683 0.683

AGT EDNRA 9606.ENSP00000355627 9606.ENSP00000315011 0 0 0 0 0.061 0.056 0.8 0.542 0.907

AGT SELE 9606.ENSP00000355627 9606.ENSP00000331736 0 0 0 0 0 0.057 0 0.403 0.413

AGT PLCB1 9606.ENSP00000355627 9606.ENSP00000338185 0 0 0 0 0.047 0.062 0.9 0.117 0.91

AGT NR3C2 9606.ENSP00000355627 9606.ENSP00000350815 0 0 0 0 0.063 0.058 0 0.68 0.693

AGT JUN 9606.ENSP00000355627 9606.ENSP00000360266 0 0 0 0 0 0 0 0.413 0.413

AGT XDH 9606.ENSP00000355627 9606.ENSP00000368727 0 0 0 0 0.062 0 0 0.402 0.415

AGT SPP1 9606.ENSP00000355627 9606.ENSP00000378517 0 0 0 0 0.142 0 0 0.372 0.438

AGT HSD11B1 9606.ENSP00000355627 9606.ENSP00000355995 0 0 0 0 0.119 0 0 0.391 0.44

AGT SHBG 9606.ENSP00000355627 9606.ENSP00000369816 0 0 0 0 0.167 0 0 0.364 0.447

AGT NOS1 9606.ENSP00000355627 9606.ENSP00000477999 0 0 0 0 0.052 0 0 0.455 0.462

AGT LCN2 9606.ENSP00000355627 9606.ENSP00000362108 0 0 0 0 0.062 0 0 0.459 0.47

AGT VEGFA 9606.ENSP00000355627 9606.ENSP00000478570 0 0 0 0 0.065 0 0 0.489 0.501

AGT MMP9 9606.ENSP00000355627 9606.ENSP00000361405 0 0 0 0 0 0.056 0 0.499 0.507

AGT AKT1 9606.ENSP00000355627 9606.ENSP00000451828 0 0 0 0 0 0 0 0.555 0.555

AGT TNF 9606.ENSP00000355627 9606.ENSP00000398698 0 0 0 0 0 0 0 0.602 0.602

AGT INS 9606.ENSP00000355627 9606.ENSP00000380432 0 0 0 0 0 0 0 0.602 0.602

AGT IL6 9606.ENSP00000355627 9606.ENSP00000385675 0 0 0 0 0 0 0 0.651 0.651

AGT CXCR4 9606.ENSP00000355627 9606.ENSP00000386884 0 0 0 0 0 0.056 0.9 0.138 0.911

AGT EDN1 9606.ENSP00000355627 9606.ENSP00000368683 0 0 0 0 0 0 0.9 0.82 0.981

AGT AGTR1 9606.ENSP00000355627 9606.ENSP00000419422 0 0 0 0 0.083 0.501 0.9 0.872 0.993

AGTR1 MMP2 9606.ENSP00000419422 9606.ENSP00000219070 0 0 0 0 0.098 0.057 0 0.414 0.458

AGTR1 TGFB1 9606.ENSP00000419422 9606.ENSP00000221930 0 0 0 0 0 0 0 0.529 0.529

AGTR1 CCL2 9606.ENSP00000419422 9606.ENSP00000225831 0 0 0 0 0.07 0.057 0 0.556 0.576

AGTR1 CAT 9606.ENSP00000419422 9606.ENSP00000241052 0 0 0 0 0.062 0 0 0.391 0.404

AGTR1 APOE 9606.ENSP00000419422 9606.ENSP00000252486 0 0 0 0 0.062 0 0 0.519 0.529

AGTR1 CRP 9606.ENSP00000419422 9606.ENSP00000255030 0 0 0 0 0.062 0 0 0.456 0.467

AGTR1 IL1B 9606.ENSP00000419422 9606.ENSP00000263341 0 0 0 0 0.056 0 0 0.587 0.594

AGTR1 ICAM1 9606.ENSP00000419422 9606.ENSP00000264832 0 0 0 0 0.055 0 0 0.422 0.43

AGTR1 REN 9606.ENSP00000419422 9606.ENSP00000272190 0 0 0 0 0.055 0 0 0.942 0.943

AGTR1 EGFR 9606.ENSP00000419422 9606.ENSP00000275493 0 0 0 0 0.076 0.229 0 0.505 0.617

AGTR1 PPARG 9606.ENSP00000419422 9606.ENSP00000287820 0 0 0 0 0.062 0.056 0 0.761 0.769

AGTR1 NCF1 9606.ENSP00000419422 9606.ENSP00000289473 0 0 0 0 0 0 0 0.519 0.519

AGTR1 VCAM1 9606.ENSP00000419422 9606.ENSP00000294728 0 0 0 0 0.076 0 0 0.46 0.479

AGTR1 ALB 9606.ENSP00000419422 9606.ENSP00000295897 0 0 0 0 0.065 0 0 0.556 0.567

AGTR1 NOS3 9606.ENSP00000419422 9606.ENSP00000297494 0 0 0 0 0.062 0.213 0 0.883 0.906

AGTR1 IGF1 9606.ENSP00000419422 9606.ENSP00000302665 0 0 0 0 0.062 0 0 0.391 0.404

AGTR1 CXCL8 9606.ENSP00000419422 9606.ENSP00000306512 0 0 0 0 0 0 0.6 0.305 0.71

AGTR1 F2 9606.ENSP00000419422 9606.ENSP00000308541 0 0 0 0 0.064 0.097 0.8 0.152 0.837

AGTR1 CASP3 9606.ENSP00000419422 9606.ENSP00000311032 0 0 0 0 0 0.056 0 0.391 0.4

AGTR1 LEP 9606.ENSP00000419422 9606.ENSP00000312652 0 0 0 0 0 0 0 0.451 0.451

AGTR1 CAV3 9606.ENSP00000419422 9606.ENSP00000341940 0 0 0 0 0.062 0.057 0 0.468 0.488

AGTR1 NR3C2 9606.ENSP00000419422 9606.ENSP00000350815 0 0 0 0 0 0.056 0 0.651 0.656

AGTR1 JUN 9606.ENSP00000419422 9606.ENSP00000360266 0 0 0 0 0 0 0 0.515 0.515

AGTR1 MMP9 9606.ENSP00000419422 9606.ENSP00000361405 0 0 0 0 0 0.057 0 0.459 0.468

AGTR1 EDN1 9606.ENSP00000419422 9606.ENSP00000368683 0 0 0 0 0 0 0 0.681 0.681

AGTR1 SPP1 9606.ENSP00000419422 9606.ENSP00000378517 0 0 0 0 0 0 0 0.403 0.403

AGTR1 INS 9606.ENSP00000419422 9606.ENSP00000380432 0 0 0 0 0 0 0 0.603 0.603

AGTR1 IL6 9606.ENSP00000419422 9606.ENSP00000385675 0 0 0 0 0.063 0 0 0.556 0.566

AGTR1 TNF 9606.ENSP00000419422 9606.ENSP00000398698 0 0 0 0 0 0 0 0.519 0.519

AGTR1 NOS1 9606.ENSP00000419422 9606.ENSP00000477999 0 0 0 0 0.049 0 0 0.425 0.429

AGTR1 AKT1 9606.ENSP00000419422 9606.ENSP00000451828 0 0 0 0 0 0.056 0 0.518 0.525

AGTR1 VEGFA 9606.ENSP00000419422 9606.ENSP00000478570 0 0 0 0 0.062 0 0 0.561 0.57

AKT1 TNFRSF1A 9606.ENSP00000451828 9606.ENSP00000162749 0 0 0 0 0.062 0.225 0 0.604 0.687

AKT1 MIF 9606.ENSP00000451828 9606.ENSP00000215754 0 0 0 0 0 0.058 0 0.468 0.477

AKT1 HMOX1 9606.ENSP00000451828 9606.ENSP00000216117 0 0 0 0 0 0.23 0 0.749 0.799

AKT1 NFKBIA 9606.ENSP00000451828 9606.ENSP00000216797 0 0 0 0 0.062 0.33 0 0.768 0.841

AKT1 MMP2 9606.ENSP00000451828 9606.ENSP00000219070 0 0 0 0 0.062 0 0 0.767 0.772

AKT1 TGFB1 9606.ENSP00000451828 9606.ENSP00000221930 0 0 0 0 0.076 0.15 0 0.634 0.688

AKT1 MPO 9606.ENSP00000451828 9606.ENSP00000225275 0 0 0 0 0 0.077 0 0.526 0.544

AKT1 CCL2 9606.ENSP00000451828 9606.ENSP00000225831 0 0 0 0 0 0 0 0.685 0.685

AKT1 COL1A1 9606.ENSP00000451828 9606.ENSP00000225964 0 0 0 0 0.062 0.056 0 0.502 0.521

AKT1 NFKB1 9606.ENSP00000451828 9606.ENSP00000226574 0 0 0 0 0.066 0.176 0.9 0.526 0.958

AKT1 IL2 9606.ENSP00000451828 9606.ENSP00000226730 0 0 0 0 0 0 0.9 0.682 0.966

AKT1 KITLG 9606.ENSP00000451828 9606.ENSP00000228280 0 0 0 0 0 0 0 0.606 0.606

AKT1 IFNG 9606.ENSP00000451828 9606.ENSP00000229135 0 0 0 0 0 0 0 0.605 0.605

AKT1 IL4 9606.ENSP00000451828 9606.ENSP00000231449 0 0 0 0 0.063 0 0 0.68 0.687

AKT1 NR3C1 9606.ENSP00000451828 9606.ENSP00000231509 0 0 0 0 0 0.297 0 0.558 0.676

AKT1 APOB 9606.ENSP00000451828 9606.ENSP00000233242 0 0 0 0 0 0 0 0.448 0.448

AKT1 CTSD 9606.ENSP00000451828 9606.ENSP00000236671 0 0 0 0 0.096 0.077 0 0.46 0.51

AKT1 APOA1 9606.ENSP00000451828 9606.ENSP00000236850 0 0 0 0 0 0 0 0.448 0.448

AKT1 CAT 9606.ENSP00000451828 9606.ENSP00000241052 0.044 0 0 0 0 0.058 0.9 0.729 0.972

AKT1 IRF1 9606.ENSP00000451828 9606.ENSP00000245414 0 0 0 0 0 0.056 0 0.415 0.424

AKT1 CCR7 9606.ENSP00000451828 9606.ENSP00000246657 0 0 0 0 0 0 0 0.471 0.471

AKT1 HSPB1 9606.ENSP00000451828 9606.ENSP00000248553 0 0 0 0 0 0.76 0 0.982 0.995

AKT1 APOE 9606.ENSP00000451828 9606.ENSP00000252486 0 0 0 0 0 0 0 0.665 0.665

AKT1 CRP 9606.ENSP00000451828 9606.ENSP00000255030 0 0 0 0 0 0 0 0.568 0.568

AKT1 NOTCH2 9606.ENSP00000451828 9606.ENSP00000256646 0 0 0 0 0 0.1 0 0.556 0.583

AKT1 MDM2 9606.ENSP00000451828 9606.ENSP00000258149 0 0 0 0 0 0.898 0.9 0.852 0.998

AKT1 SMAD7 9606.ENSP00000451828 9606.ENSP00000262158 0 0 0 0 0.062 0.334 0 0.556 0.698

AKT1 IL1A 9606.ENSP00000451828 9606.ENSP00000263339 0 0 0 0 0 0 0 0.521 0.521

AKT1 IL1B 9606.ENSP00000451828 9606.ENSP00000263341 0 0 0 0 0 0.056 0 0.774 0.777

AKT1 SELP 9606.ENSP00000451828 9606.ENSP00000263686 0 0 0 0 0 0.058 0 0.513 0.521

AKT1 FGF2 9606.ENSP00000451828 9606.ENSP00000264498 0 0 0 0 0.062 0 0 0.75 0.755

AKT1 FGF10 9606.ENSP00000451828 9606.ENSP00000264664 0 0 0 0 0 0 0 0.401 0.401

AKT1 ICAM1 9606.ENSP00000451828 9606.ENSP00000264832 0 0 0 0 0 0 0 0.678 0.678

AKT1 LEF1 9606.ENSP00000451828 9606.ENSP00000265165 0 0 0 0 0 0.057 0 0.535 0.543

AKT1 EGF 9606.ENSP00000451828 9606.ENSP00000265171 0 0 0 0 0 0.056 0 0.859 0.861

AKT1 ERBB3 9606.ENSP00000451828 9606.ENSP00000267101 0 0 0 0 0 0.32 0.9 0.845 0.988

AKT1 TP53 9606.ENSP00000451828 9606.ENSP00000269305 0 0 0 0 0.053 0.201 0.9 0.877 0.989

AKT1 ERBB2 9606.ENSP00000451828 9606.ENSP00000269571 0 0 0 0.556 0.053 0.188 0 0.898 0.516

AKT1 SOD1 9606.ENSP00000451828 9606.ENSP00000270142 0 0 0 0 0 0.09 0.9 0.569 0.957

AKT1 REN 9606.ENSP00000451828 9606.ENSP00000272190 0 0 0 0 0 0.077 0 0.607 0.621

AKT1 EGFR 9606.ENSP00000451828 9606.ENSP00000275493 0 0 0 0.554 0 0.65 0 0.926 0.794

AKT1 BMP6 9606.ENSP00000451828 9606.ENSP00000283147 0 0 0 0 0 0.15 0 0.335 0.41

AKT1 SST 9606.ENSP00000451828 9606.ENSP00000287641 0 0 0 0 0 0 0 0.477 0.477

AKT1 PPARG 9606.ENSP00000451828 9606.ENSP00000287820 0 0 0 0 0.062 0.077 0 0.836 0.846

AKT1 HMGCR 9606.ENSP00000451828 9606.ENSP00000287936 0 0 0 0 0 0.058 0 0.56 0.568

AKT1 KIT 9606.ENSP00000451828 9606.ENSP00000288135 0 0 0 0 0 0.122 0 0.736 0.758

AKT1 NCF1 9606.ENSP00000451828 9606.ENSP00000289473 0 0 0 0 0 0.213 0 0.984 0.987

AKT1 BAX 9606.ENSP00000451828 9606.ENSP00000293288 0 0 0 0 0.061 0.27 0 0.682 0.763

AKT1 VCAM1 9606.ENSP00000451828 9606.ENSP00000294728 0 0 0 0 0 0 0 0.659 0.659

AKT1 ALB 9606.ENSP00000451828 9606.ENSP00000295897 0 0 0 0 0 0 0 0.792 0.792

AKT1 TLR3 9606.ENSP00000451828 9606.ENSP00000296795 0 0 0 0 0 0.168 0 0.47 0.54

AKT1 CSF2 9606.ENSP00000451828 9606.ENSP00000296871 0 0 0 0 0 0 0 0.603 0.603

AKT1 NOS3 9606.ENSP00000451828 9606.ENSP00000297494 0 0 0 0 0.049 0.879 0.9 0.988 0.999

AKT1 HPRT1 9606.ENSP00000451828 9606.ENSP00000298556 0.046 0 0 0 0 0 0 0.459 0.461

AKT1 MMP3 9606.ENSP00000451828 9606.ENSP00000299855 0 0 0 0 0 0 0 0.602 0.602

AKT1 FADD 9606.ENSP00000451828 9606.ENSP00000301838 0 0 0 0 0.073 0.069 0 0.564 0.591

AKT1 BCL2L1 9606.ENSP00000451828 9606.ENSP00000302564 0 0 0 0 0.065 0.27 0.9 0.982 0.998

AKT1 IGF1 9606.ENSP00000451828 9606.ENSP00000302665 0 0 0 0 0 0 0 0.878 0.878

AKT1 INSR 9606.ENSP00000451828 9606.ENSP00000303830 0 0 0 0.559 0.052 0.182 0.9 0.73 0.942

AKT1 IL13 9606.ENSP00000451828 9606.ENSP00000304915 0 0 0 0 0 0 0 0.536 0.536

AKT1 PRKCB 9606.ENSP00000451828 9606.ENSP00000305355 0 0 0 0.868 0 0.456 0 0.56 0.495

AKT1 CXCL10 9606.ENSP00000451828 9606.ENSP00000305651 0 0 0 0 0 0 0 0.502 0.502

AKT1 ITPR1 9606.ENSP00000451828 9606.ENSP00000306253 0 0 0 0 0 0.493 0 0.818 0.904

AKT1 CXCL8 9606.ENSP00000451828 9606.ENSP00000306512 0 0 0 0 0 0 0 0.714 0.714

AKT1 CYCS 9606.ENSP00000451828 9606.ENSP00000307786 0 0 0 0 0 0 0 0.806 0.806

AKT1 F2 9606.ENSP00000451828 9606.ENSP00000308541 0 0 0 0 0 0.056 0 0.521 0.529

AKT1 LPL 9606.ENSP00000451828 9606.ENSP00000309757 0 0 0 0 0 0 0 0.472 0.472

AKT1 PPARD 9606.ENSP00000451828 9606.ENSP00000310928 0 0 0 0 0.062 0.077 0 0.419 0.453

AKT1 CASP3 9606.ENSP00000451828 9606.ENSP00000311032 0 0 0 0 0 0.475 0 0.882 0.935

AKT1 LEP 9606.ENSP00000451828 9606.ENSP00000312652 0 0 0 0 0 0 0 0.739 0.739

AKT1 EDNRA 9606.ENSP00000451828 9606.ENSP00000315011 0 0 0 0 0 0.056 0.9 0.391 0.937

AKT1 MMP1 9606.ENSP00000451828 9606.ENSP00000322788 0 0 0 0 0 0 0 0.56 0.56

AKT1 HSPA5 9606.ENSP00000451828 9606.ENSP00000324173 0 0 0 0 0 0.531 0 0.983 0.992

AKT1 CD28 9606.ENSP00000451828 9606.ENSP00000324890 0 0 0 0 0.063 0.056 0.65 0.621 0.867

AKT1 NOS2 9606.ENSP00000451828 9606.ENSP00000327251 0 0 0 0 0.063 0.077 0.9 0.646 0.965

AKT1 P4HB 9606.ENSP00000451828 9606.ENSP00000327801 0 0 0 0 0.095 0.078 0 0.406 0.461

AKT1 SOCS1 9606.ENSP00000451828 9606.ENSP00000329418 0 0 0 0 0 0.103 0 0.52 0.551

AKT1 CASP9 9606.ENSP00000451828 9606.ENSP00000330237 0 0 0 0 0 0.231 0.9 0.814 0.984

AKT1 SELE 9606.ENSP00000451828 9606.ENSP00000331736 0 0 0 0 0 0.058 0 0.527 0.535

AKT1 CALCA 9606.ENSP00000451828 9606.ENSP00000331746 0 0 0 0 0 0 0 0.408 0.408

AKT1 CYP3A4 9606.ENSP00000451828 9606.ENSP00000337915 0 0 0 0 0 0.056 0 0.392 0.401

AKT1 SNCA 9606.ENSP00000451828 9606.ENSP00000338345 0 0 0 0 0 0.307 0 0.513 0.649

AKT1 STAT5A 9606.ENSP00000451828 9606.ENSP00000341208 0 0 0 0 0.06 0.14 0 0.786 0.811

AKT1 CAV3 9606.ENSP00000451828 9606.ENSP00000341940 0 0 0 0 0 0.076 0 0.49 0.509

AKT1 USP7 9606.ENSP00000451828 9606.ENSP00000343535 0 0 0 0 0.062 0.058 0 0.416 0.439

AKT1 IL17A 9606.ENSP00000451828 9606.ENSP00000344192 0 0 0 0 0 0 0 0.601 0.601

AKT1 E2F1 9606.ENSP00000451828 9606.ENSP00000345571 0 0 0 0 0.092 0.075 0.9 0.486 0.951

AKT1 OCLN 9606.ENSP00000451828 9606.ENSP00000347379 0 0 0 0 0 0.057 0 0.556 0.564

AKT1 FAS 9606.ENSP00000451828 9606.ENSP00000347979 0 0 0 0 0 0 0 0.548 0.548

AKT1 SREBF1 9606.ENSP00000451828 9606.ENSP00000348069 0 0 0 0 0.085 0.062 0 0.718 0.737

AKT1 CASP8 9606.ENSP00000451828 9606.ENSP00000351273 0 0 0 0 0 0.063 0 0.76 0.766

AKT1 VCP 9606.ENSP00000451828 9606.ENSP00000351777 0 0 0 0 0.073 0.676 0 0.427 0.813

AKT1 DNMT1 9606.ENSP00000451828 9606.ENSP00000352516 0 0 0 0 0 0.27 0 0.559 0.664

AKT1 STAT1 9606.ENSP00000451828 9606.ENSP00000354394 0 0 0 0 0 0.294 0.9 0.702 0.977

AKT1 TOP1 9606.ENSP00000451828 9606.ENSP00000354522 0 0 0 0 0 0 0 0.489 0.489

AKT1 MTOR 9606.ENSP00000451828 9606.ENSP00000354558 0 0 0 0 0.081 0.904 0.9 0.991 0.999

AKT1 PARP1 9606.ENSP00000451828 9606.ENSP00000355759 0 0 0 0 0.062 0 0 0.659 0.666

AKT1 FASLG 9606.ENSP00000451828 9606.ENSP00000356694 0 0 0 0 0 0 0 0.648 0.648

AKT1 BGLAP 9606.ENSP00000451828 9606.ENSP00000357255 0 0 0 0 0 0 0 0.517 0.517

AKT1 NGF 9606.ENSP00000451828 9606.ENSP00000358525 0 0 0 0 0 0.056 0 0.729 0.734

AKT1 CHUK 9606.ENSP00000451828 9606.ENSP00000359424 0 0 0 0.593 0 0.872 0.9 0.898 0.991

AKT1 CD40LG 9606.ENSP00000451828 9606.ENSP00000359663 0 0 0 0 0 0.056 0.9 0.493 0.947

AKT1 JUN 9606.ENSP00000451828 9606.ENSP00000360266 0 0 0 0 0 0.057 0.8 0.877 0.974

AKT1 PTEN 9606.ENSP00000451828 9606.ENSP00000361021 0 0 0 0 0.062 0.852 0 0.952 0.992

AKT1 CD40 9606.ENSP00000451828 9606.ENSP00000361359 0 0 0 0 0 0.078 0.9 0.561 0.956

AKT1 MMP9 9606.ENSP00000451828 9606.ENSP00000361405 0 0 0 0 0.062 0 0 0.811 0.815

AKT1 HDAC1 9606.ENSP00000451828 9606.ENSP00000362649 0 0 0 0 0 0.078 0 0.598 0.614

AKT1 TLR4 9606.ENSP00000451828 9606.ENSP00000363089 0 0 0 0 0.063 0.168 0 0.696 0.742

AKT1 ITPR3 9606.ENSP00000451828 9606.ENSP00000363435 0 0 0 0 0.07 0.282 0 0.972 0.98

AKT1 ALOX5 9606.ENSP00000451828 9606.ENSP00000363512 0 0 0 0 0 0 0 0.456 0.456

AKT1 FOXP3 9606.ENSP00000451828 9606.ENSP00000365380 0 0 0 0 0 0.118 0 0.607 0.639

AKT1 EDN1 9606.ENSP00000451828 9606.ENSP00000368683 0 0 0 0 0.062 0 0.9 0.673 0.966

AKT1 TH 9606.ENSP00000451828 9606.ENSP00000370571 0 0 0 0 0 0 0 0.536 0.536

AKT1 GPT 9606.ENSP00000451828 9606.ENSP00000378408 0 0 0 0 0.098 0.057 0 0.556 0.589

AKT1 SPP1 9606.ENSP00000451828 9606.ENSP00000378517 0 0 0 0 0 0.06 0 0.61 0.618

AKT1 CYP19A1 9606.ENSP00000451828 9606.ENSP00000379683 0 0 0 0 0 0.056 0 0.609 0.615

AKT1 INS 9606.ENSP00000451828 9606.ENSP00000380432 0 0 0 0 0 0 0.9 0.908 0.99

AKT1 ITGB2 9606.ENSP00000451828 9606.ENSP00000380948 0 0 0 0 0.076 0.07 0 0.414 0.452

AKT1 BCL2 9606.ENSP00000451828 9606.ENSP00000381185 0 0 0 0 0.062 0 0.9 0.457 0.944

AKT1 CDKN1A 9606.ENSP00000451828 9606.ENSP00000384849 0 0 0 0 0 0.789 0.9 0.71 0.993

AKT1 IL6 9606.ENSP00000451828 9606.ENSP00000385675 0 0 0 0 0.056 0 0 0.829 0.832

AKT1 CXCR4 9606.ENSP00000451828 9606.ENSP00000386884 0 0 0 0 0.062 0.056 0 0.71 0.721

AKT1 TNF 9606.ENSP00000451828 9606.ENSP00000398698 0 0 0 0 0 0.056 0 0.826 0.829

AKT1 MYD88 9606.ENSP00000451828 9606.ENSP00000401399 0 0 0 0 0 0.149 0 0.648 0.688

AKT1 ESR1 9606.ENSP00000451828 9606.ENSP00000405330 0 0 0 0 0.055 0.297 0.9 0.805 0.985

AKT1 IL10 9606.ENSP00000451828 9606.ENSP00000412237 0 0 0 0 0 0.06 0 0.69 0.697

AKT1 BDNF 9606.ENSP00000451828 9606.ENSP00000414303 0 0 0 0 0 0.056 0 0.774 0.778

AKT1 BRCA1 9606.ENSP00000451828 9606.ENSP00000418960 0 0 0 0 0 0.835 0.8 0.657 0.987

AKT1 HIF1A 9606.ENSP00000451828 9606.ENSP00000437955 0 0 0 0 0 0.261 0.9 0.887 0.99

AKT1 VDR 9606.ENSP00000451828 9606.ENSP00000447173 0 0 0 0 0 0.077 0 0.511 0.53

AKT1 ELANE 9606.ENSP00000451828 9606.ENSP00000466090 0 0 0 0 0 0.056 0 0.392 0.401

AKT1 STIM1 9606.ENSP00000451828 9606.ENSP00000478059 0 0 0 0 0 0 0 0.418 0.418

AKT1 FURIN 9606.ENSP00000451828 9606.ENSP00000483552 0 0 0 0 0.106 0.056 0 0.429 0.476

AKT1 CCL3 9606.ENSP00000451828 9606.ENSP00000477908 0 0 0 0 0 0 0 0.478 0.478

AKT1 GRIN2B 9606.ENSP00000451828 9606.ENSP00000477455 0 0 0 0 0 0.07 0 0.504 0.518

AKT1 CCL5 9606.ENSP00000451828 9606.ENSP00000474412 0 0 0 0 0 0 0 0.561 0.561

AKT1 VEGFA 9606.ENSP00000451828 9606.ENSP00000478570 0 0 0 0 0.063 0.213 0 0.871 0.896

AKT1 NOS1 9606.ENSP00000451828 9606.ENSP00000477999 0 0 0 0 0.049 0.077 0.8 0.589 0.918

ALB CFTR 9606.ENSP00000295897 9606.ENSP00000003084 0 0 0 0 0.062 0 0 0.568 0.578

ALB TNFRSF1A 9606.ENSP00000295897 9606.ENSP00000162749 0 0 0 0 0.062 0 0 0.509 0.519

ALB AQP2 9606.ENSP00000295897 9606.ENSP00000199280 0 0 0 0 0.052 0 0 0.483 0.489

ALB CETP 9606.ENSP00000295897 9606.ENSP00000200676 0 0 0 0 0.062 0 0 0.451 0.463

ALB APOH 9606.ENSP00000295897 9606.ENSP00000205948 0 0 0 0 0.797 0 0 0.52 0.898

ALB MIF 9606.ENSP00000295897 9606.ENSP00000215754 0 0 0 0 0 0 0 0.447 0.447

ALB HMOX1 9606.ENSP00000295897 9606.ENSP00000216117 0 0 0 0 0 0 0 0.693 0.693

ALB CTSG 9606.ENSP00000295897 9606.ENSP00000216336 0 0 0 0 0.053 0.078 0 0.454 0.481

ALB NFKBIA 9606.ENSP00000295897 9606.ENSP00000216797 0 0 0 0 0 0 0 0.646 0.646

ALB MMP2 9606.ENSP00000295897 9606.ENSP00000219070 0 0 0 0 0.081 0.141 0 0.634 0.686

ALB TGFB1 9606.ENSP00000295897 9606.ENSP00000221930 0 0 0 0 0 0 0 0.658 0.658

ALB PON1 9606.ENSP00000295897 9606.ENSP00000222381 0 0 0 0 0.072 0.213 0 0.557 0.648

ALB MPO 9606.ENSP00000295897 9606.ENSP00000225275 0 0 0 0 0 0.128 0 0.876 0.887

ALB CCL2 9606.ENSP00000295897 9606.ENSP00000225831 0 0 0 0 0 0 0 0.74 0.74

ALB COL1A1 9606.ENSP00000295897 9606.ENSP00000225964 0 0 0 0 0 0 0 0.566 0.566

ALB IL2 9606.ENSP00000295897 9606.ENSP00000226730 0 0 0 0 0 0 0 0.775 0.775

ALB KITLG 9606.ENSP00000295897 9606.ENSP00000228280 0 0 0 0 0 0 0 0.606 0.606

ALB IFNG 9606.ENSP00000295897 9606.ENSP00000229135 0 0 0 0 0 0 0 0.74 0.74

ALB IL4 9606.ENSP00000295897 9606.ENSP00000231449 0 0 0 0 0 0 0 0.737 0.737

ALB NR3C1 9606.ENSP00000295897 9606.ENSP00000231509 0 0 0 0 0.052 0.176 0 0.582 0.645

ALB LTF 9606.ENSP00000295897 9606.ENSP00000231751 0 0 0 0 0.344 0 0 0.516 0.669

ALB TFPI 9606.ENSP00000295897 9606.ENSP00000233156 0 0 0 0 0.1 0.13 0 0.403 0.491

ALB APOB 9606.ENSP00000295897 9606.ENSP00000233242 0 0 0 0 0.564 0 0 0.855 0.934

ALB CTSD 9606.ENSP00000295897 9606.ENSP00000236671 0 0 0 0 0.062 0 0 0.59 0.599

ALB APOA1 9606.ENSP00000295897 9606.ENSP00000236850 0 0 0 0 0.698 0.35 0.65 0.843 0.987

ALB FGF23 9606.ENSP00000295897 9606.ENSP00000237837 0 0 0 0 0 0 0 0.662 0.662

ALB CAT 9606.ENSP00000295897 9606.ENSP00000241052 0 0 0 0 0.064 0 0 0.834 0.838

ALB C3 9606.ENSP00000295897 9606.ENSP00000245907 0 0 0 0 0.144 0.223 0 0.93 0.949

ALB CCR7 9606.ENSP00000295897 9606.ENSP00000246657 0 0 0 0 0 0 0 0.542 0.542

ALB HSPB1 9606.ENSP00000295897 9606.ENSP00000248553 0 0 0 0 0 0.128 0 0.543 0.585

ALB HBA2 9606.ENSP00000295897 9606.ENSP00000251595 0 0 0 0 0.062 0 0 0.607 0.615

ALB APOE 9606.ENSP00000295897 9606.ENSP00000252486 0 0 0 0 0.096 0.262 0 0.954 0.967

ALB CRP 9606.ENSP00000295897 9606.ENSP00000255030 0 0 0 0 0.111 0 0 0.915 0.921

ALB MDM2 9606.ENSP00000295897 9606.ENSP00000258149 0 0 0 0 0 0.27 0 0.482 0.606

ALB ORM1 9606.ENSP00000295897 9606.ENSP00000259396 0 0 0 0 0.557 0.267 0 0.786 0.924

ALB CYP2C9 9606.ENSP00000295897 9606.ENSP00000260682 0 0 0 0 0.084 0 0 0.63 0.646

ALB LYZ 9606.ENSP00000295897 9606.ENSP00000261267 0 0 0 0 0.093 0 0 0.599 0.621

ALB SLC6A4 9606.ENSP00000295897 9606.ENSP00000261707 0 0 0 0 0.052 0 0 0.485 0.491

ALB SMAD7 9606.ENSP00000295897 9606.ENSP00000262158 0 0 0 0 0 0 0 0.456 0.456

ALB IL1A 9606.ENSP00000295897 9606.ENSP00000263339 0 0 0 0 0.062 0 0 0.594 0.602

ALB IL1B 9606.ENSP00000295897 9606.ENSP00000263341 0 0 0 0 0 0 0 0.822 0.822

ALB SELP 9606.ENSP00000295897 9606.ENSP00000263686 0 0 0 0 0.062 0 0 0.649 0.656

ALB FGF2 9606.ENSP00000295897 9606.ENSP00000264498 0 0 0 0 0 0 0 0.821 0.821

ALB FGF10 9606.ENSP00000295897 9606.ENSP00000264664 0 0 0 0 0 0 0 0.506 0.506

ALB ICAM1 9606.ENSP00000295897 9606.ENSP00000264832 0 0 0 0 0 0 0 0.711 0.711

ALB EGF 9606.ENSP00000295897 9606.ENSP00000265171 0 0 0 0 0 0 0 0.817 0.817

ALB ERBB3 9606.ENSP00000295897 9606.ENSP00000267101 0 0 0 0 0.09 0 0 0.891 0.897

ALB TP53 9606.ENSP00000295897 9606.ENSP00000269305 0 0 0 0 0 0 0 0.715 0.715

ALB ERBB2 9606.ENSP00000295897 9606.ENSP00000269571 0 0 0 0 0 0 0 0.923 0.923

ALB SOD1 9606.ENSP00000295897 9606.ENSP00000270142 0 0 0 0 0 0.151 0 0.538 0.591

ALB REN 9606.ENSP00000295897 9606.ENSP00000272190 0 0 0 0 0.096 0 0 0.832 0.841

ALB EGFR 9606.ENSP00000295897 9606.ENSP00000275493 0 0 0 0 0.062 0 0 0.784 0.789

ALB CRH 9606.ENSP00000295897 9606.ENSP00000276571 0 0 0 0 0 0 0 0.543 0.543

ALB CA2 9606.ENSP00000295897 9606.ENSP00000285379 0 0 0 0 0 0 0 0.461 0.461

ALB CXCL13 9606.ENSP00000295897 9606.ENSP00000286758 0 0 0 0 0.062 0 0 0.392 0.405

ALB SST 9606.ENSP00000295897 9606.ENSP00000287641 0 0 0 0 0.062 0 0 0.634 0.642

ALB PPARG 9606.ENSP00000295897 9606.ENSP00000287820 0 0 0 0 0.058 0.058 0 0.727 0.737

ALB HMGCR 9606.ENSP00000295897 9606.ENSP00000287936 0 0 0 0 0 0 0 0.483 0.483

ALB KIT 9606.ENSP00000295897 9606.ENSP00000288135 0 0 0 0 0 0 0 0.687 0.687

ALB NCF1 9606.ENSP00000295897 9606.ENSP00000289473 0 0 0 0 0 0 0 0.519 0.519

ALB BAX 9606.ENSP00000295897 9606.ENSP00000293288 0 0 0 0 0.049 0 0 0.458 0.462

ALB VCAM1 9606.ENSP00000295897 9606.ENSP00000294728 0 0 0 0 0 0 0 0.668 0.668

ALB RET 9606.ENSP00000295897 9606.ENSP00000347942 0 0 0 0 0 0 0 0.406 0.406

ALB FASLG 9606.ENSP00000295897 9606.ENSP00000356694 0 0 0 0 0 0 0 0.407 0.407

ALB EDNRA 9606.ENSP00000295897 9606.ENSP00000315011 0 0 0 0 0 0 0 0.41 0.41

ALB HNF1B 9606.ENSP00000295897 9606.ENSP00000480291 0 0 0 0 0.064 0 0 0.408 0.422

ALB XDH 9606.ENSP00000295897 9606.ENSP00000368727 0 0 0 0 0.062 0 0 0.411 0.424

ALB KCNJ10 9606.ENSP00000295897 9606.ENSP00000357068 0 0 0 0 0 0 0 0.43 0.43

ALB ITGAL 9606.ENSP00000295897 9606.ENSP00000349252 0 0 0 0 0 0 0 0.433 0.433

ALB CHUK 9606.ENSP00000295897 9606.ENSP00000359424 0 0 0 0 0 0 0 0.447 0.447

ALB S100A8 9606.ENSP00000295897 9606.ENSP00000357722 0 0 0 0 0 0.128 0 0.4 0.454

ALB DNMT1 9606.ENSP00000295897 9606.ENSP00000352516 0 0 0 0 0 0 0 0.456 0.456

ALB CYP19A1 9606.ENSP00000295897 9606.ENSP00000379683 0 0 0 0 0.049 0 0 0.452 0.457

ALB MTTP 9606.ENSP00000295897 9606.ENSP00000427679 0 0 0 0 0.17 0 0 0.38 0.464

ALB HTR2A 9606.ENSP00000295897 9606.ENSP00000437737 0 0 0 0 0 0 0 0.465 0.466

ALB TPO 9606.ENSP00000295897 9606.ENSP00000318820 0 0 0 0 0 0 0 0.468 0.468

ALB GRIN2B 9606.ENSP00000295897 9606.ENSP00000477455 0 0 0 0 0 0 0 0.47 0.47

ALB STAT5A 9606.ENSP00000295897 9606.ENSP00000341208 0 0 0 0 0.069 0 0 0.468 0.484

ALB PF4 9606.ENSP00000295897 9606.ENSP00000296029 0 0 0 0 0 0 0 0.487 0.487

ALB GSTM1 9606.ENSP00000295897 9606.ENSP00000311469 0 0 0 0 0.062 0 0 0.483 0.494

ALB HDAC1 9606.ENSP00000295897 9606.ENSP00000362649 0 0 0 0 0 0 0 0.495 0.495

ALB CX3CR1 9606.ENSP00000295897 9606.ENSP00000351059 0 0 0 0 0 0 0 0.496 0.496

ALB CDKN1A 9606.ENSP00000295897 9606.ENSP00000384849 0 0 0 0 0 0 0 0.496 0.496

ALB NR3C2 9606.ENSP00000295897 9606.ENSP00000350815 0 0 0 0 0.052 0.058 0 0.492 0.506

ALB BRCA1 9606.ENSP00000295897 9606.ENSP00000418960 0 0 0 0 0 0 0 0.512 0.512

ALB THBD 9606.ENSP00000295897 9606.ENSP00000366307 0 0 0 0 0 0 0 0.515 0.515

ALB FAS 9606.ENSP00000295897 9606.ENSP00000347979 0 0 0 0 0.062 0 0 0.51 0.521

ALB TLR3 9606.ENSP00000295897 9606.ENSP00000296795 0 0 0 0 0 0 0 0.524 0.524

ALB CD40LG 9606.ENSP00000295897 9606.ENSP00000359663 0 0 0 0 0 0 0 0.526 0.526

ALB VDR 9606.ENSP00000295897 9606.ENSP00000447173 0 0 0 0 0.052 0.058 0 0.519 0.532

ALB P4HB 9606.ENSP00000295897 9606.ENSP00000327801 0 0 0 0 0 0 0 0.539 0.539

ALB SREBF1 9606.ENSP00000295897 9606.ENSP00000348069 0 0 0 0 0.062 0 0 0.532 0.542

ALB NOS1 9606.ENSP00000295897 9606.ENSP00000477999 0 0 0 0 0 0 0 0.548 0.548

ALB NOS2 9606.ENSP00000295897 9606.ENSP00000327251 0 0 0 0 0 0 0 0.55 0.55

ALB TOP1 9606.ENSP00000295897 9606.ENSP00000354522 0 0 0 0 0 0 0 0.553 0.553

ALB CCL3 9606.ENSP00000295897 9606.ENSP00000477908 0 0 0 0 0 0 0 0.555 0.555

ALB PARP1 9606.ENSP00000295897 9606.ENSP00000355759 0 0 0 0 0 0 0 0.556 0.556

ALB FURIN 9606.ENSP00000295897 9606.ENSP00000483552 0 0 0 0 0.062 0 0 0.556 0.565

ALB HPRT1 9606.ENSP00000295897 9606.ENSP00000298556 0 0 0 0 0 0 0 0.572 0.572

ALB CXCL10 9606.ENSP00000295897 9606.ENSP00000305651 0 0 0 0 0 0 0 0.577 0.577

ALB MYD88 9606.ENSP00000295897 9606.ENSP00000401399 0 0 0 0 0 0 0 0.589 0.589

ALB STAT1 9606.ENSP00000295897 9606.ENSP00000354394 0 0 0 0 0 0 0 0.589 0.589

ALB CYP1A2 9606.ENSP00000295897 9606.ENSP00000342007 0 0 0 0 0.062 0 0 0.582 0.591

ALB MMP1 9606.ENSP00000295897 9606.ENSP00000322788 0 0 0 0 0.058 0.141 0 0.541 0.596

ALB CD40 9606.ENSP00000295897 9606.ENSP00000361359 0 0 0 0 0 0 0 0.601 0.601

ALB CALCA 9606.ENSP00000295897 9606.ENSP00000331746 0 0 0 0 0 0 0 0.619 0.619

ALB CCL5 9606.ENSP00000295897 9606.ENSP00000474412 0 0 0 0 0 0 0 0.627 0.627

ALB HBA1 9606.ENSP00000295897 9606.ENSP00000322421 0 0 0 0 0.096 0 0 0.607 0.629

ALB MTOR 9606.ENSP00000295897 9606.ENSP00000354558 0 0 0 0 0 0 0 0.629 0.629

ALB CD28 9606.ENSP00000295897 9606.ENSP00000324890 0 0 0 0 0 0 0 0.634 0.634

ALB HIF1A 9606.ENSP00000295897 9606.ENSP00000437955 0 0 0 0 0 0 0 0.635 0.635

ALB CASP8 9606.ENSP00000295897 9606.ENSP00000351273 0 0 0 0 0 0 0 0.638 0.638

ALB PTEN 9606.ENSP00000295897 9606.ENSP00000361021 0 0 0 0 0 0 0 0.655 0.655

ALB HSPA5 9606.ENSP00000295897 9606.ENSP00000324173 0 0 0 0 0 0 0 0.657 0.657

ALB CXCR4 9606.ENSP00000295897 9606.ENSP00000386884 0 0 0 0 0 0 0 0.659 0.659

ALB IL13 9606.ENSP00000295897 9606.ENSP00000304915 0 0 0 0 0 0 0 0.662 0.662

ALB SELE 9606.ENSP00000295897 9606.ENSP00000331736 0 0 0 0 0 0 0 0.661 0.662

ALB BCL2L1 9606.ENSP00000295897 9606.ENSP00000302564 0 0 0 0 0.049 0 0 0.668 0.671

ALB CASP9 9606.ENSP00000295897 9606.ENSP00000330237 0 0 0 0 0 0 0 0.672 0.672

ALB TH 9606.ENSP00000295897 9606.ENSP00000370571 0 0 0 0 0.066 0 0 0.665 0.673

ALB MMP3 9606.ENSP00000295897 9606.ENSP00000299855 0 0 0 0 0.058 0.141 0 0.633 0.677

ALB IL17A 9606.ENSP00000295897 9606.ENSP00000344192 0 0 0 0 0 0 0 0.689 0.689

ALB NOS3 9606.ENSP00000295897 9606.ENSP00000297494 0 0 0 0 0.062 0 0 0.683 0.69

ALB ESR1 9606.ENSP00000295897 9606.ENSP00000405330 0 0 0 0 0.062 0.058 0 0.681 0.693

ALB SNCA 9606.ENSP00000295897 9606.ENSP00000338345 0 0 0 0 0 0 0 0.693 0.694

ALB BGLAP 9606.ENSP00000295897 9606.ENSP00000357255 0 0 0 0 0 0 0 0.693 0.694

ALB JUN 9606.ENSP00000295897 9606.ENSP00000360266 0 0 0 0 0 0 0 0.698 0.698

ALB ELANE 9606.ENSP00000295897 9606.ENSP00000466090 0 0 0 0 0.053 0.276 0 0.603 0.704

ALB SPP1 9606.ENSP00000295897 9606.ENSP00000378517 0 0 0 0 0.076 0 0 0.695 0.706

ALB LCN2 9606.ENSP00000295897 9606.ENSP00000362108 0 0 0 0 0.062 0 0 0.703 0.709

ALB OCLN 9606.ENSP00000295897 9606.ENSP00000347379 0 0 0 0 0.062 0 0 0.703 0.709

ALB CSF2 9606.ENSP00000295897 9606.ENSP00000296871 0 0 0 0 0 0 0 0.725 0.725

ALB BDNF 9606.ENSP00000295897 9606.ENSP00000414303 0 0 0 0 0 0 0 0.73 0.73

ALB FOXP3 9606.ENSP00000295897 9606.ENSP00000365380 0 0 0 0 0 0.257 0 0.669 0.743

ALB CXCL8 9606.ENSP00000295897 9606.ENSP00000306512 0 0 0 0 0 0 0 0.743 0.743

ALB LEP 9606.ENSP00000295897 9606.ENSP00000312652 0 0 0 0 0 0 0 0.745 0.746

ALB CYP3A4 9606.ENSP00000295897 9606.ENSP00000337915 0 0 0 0 0.064 0 0 0.748 0.754

ALB IGF1 9606.ENSP00000295897 9606.ENSP00000302665 0 0 0 0 0.081 0 0 0.76 0.77

ALB MMP9 9606.ENSP00000295897 9606.ENSP00000361405 0 0 0 0 0.062 0.141 0 0.741 0.773

ALB EDN1 9606.ENSP00000295897 9606.ENSP00000368683 0 0 0 0 0 0 0 0.774 0.774

ALB VEGFA 9606.ENSP00000295897 9606.ENSP00000478570 0 0 0 0 0.062 0 0 0.78 0.785

ALB TLR4 9606.ENSP00000295897 9606.ENSP00000363089 0 0 0 0 0 0 0 0.794 0.794

ALB LPL 9606.ENSP00000295897 9606.ENSP00000309757 0 0 0 0 0 0 0 0.796 0.796

ALB IL10 9606.ENSP00000295897 9606.ENSP00000412237 0 0 0 0 0 0 0 0.808 0.808

ALB NGF 9606.ENSP00000295897 9606.ENSP00000358525 0 0 0 0 0 0 0 0.843 0.844

ALB CASP3 9606.ENSP00000295897 9606.ENSP00000311032 0 0 0 0 0 0 0 0.852 0.852

ALB IL6 9606.ENSP00000295897 9606.ENSP00000385675 0 0 0 0 0 0 0 0.86 0.86

ALB CYCS 9606.ENSP00000295897 9606.ENSP00000307786 0 0 0 0 0 0 0 0.86 0.86

ALB TNF 9606.ENSP00000295897 9606.ENSP00000398698 0 0 0 0 0 0 0 0.866 0.866

ALB GPT 9606.ENSP00000295897 9606.ENSP00000378408 0 0 0 0 0.079 0 0 0.915 0.918

ALB F2 9606.ENSP00000295897 9606.ENSP00000308541 0 0 0 0 0.624 0.073 0 0.908 0.965

ALB SHBG 9606.ENSP00000295897 9606.ENSP00000369816 0 0 0 0 0.062 0 0 0.989 0.989

ALB INS 9606.ENSP00000295897 9606.ENSP00000380432 0 0 0 0 0 0 0 0.99 0.99

ALDH3A1 CAT 9606.ENSP00000411821 9606.ENSP00000241052 0 0 0 0 0.079 0.172 0 0.375 0.482

ALDH3A1 EGF 9606.ENSP00000411821 9606.ENSP00000265171 0 0 0 0 0 0 0 0.405 0.405

ALDH3A1 TP53 9606.ENSP00000411821 9606.ENSP00000269305 0 0 0 0 0 0 0 0.428 0.428

ALDH3A1 ERBB2 9606.ENSP00000411821 9606.ENSP00000269571 0 0 0 0 0.062 0 0 0.405 0.418

ALDH3A1 GSTM1 9606.ENSP00000411821 9606.ENSP00000311469 0 0 0 0 0 0.104 0.65 0.294 0.759

ALDH3A1 CYP1B1 9606.ENSP00000411821 9606.ENSP00000478561 0 0 0 0 0.062 0.061 0 0.62 0.636

ALOX5 HMOX1 9606.ENSP00000363512 9606.ENSP00000216117 0 0 0 0 0.06 0 0 0.404 0.415

ALOX5 MMP2 9606.ENSP00000363512 9606.ENSP00000219070 0 0 0 0 0 0 0 0.503 0.503

ALOX5 MPO 9606.ENSP00000363512 9606.ENSP00000225275 0 0 0 0 0.121 0 0 0.478 0.522

ALOX5 CCL2 9606.ENSP00000363512 9606.ENSP00000225831 0 0 0 0 0 0 0 0.466 0.465

ALOX5 IL4 9606.ENSP00000363512 9606.ENSP00000231449 0 0 0 0 0 0 0.9 0.469 0.944

ALOX5 CAT 9606.ENSP00000363512 9606.ENSP00000241052 0 0 0 0 0 0 0 0.411 0.411

ALOX5 APOE 9606.ENSP00000363512 9606.ENSP00000252486 0 0 0 0 0 0 0 0.413 0.412

ALOX5 CYP2C9 9606.ENSP00000363512 9606.ENSP00000260682 0 0 0 0 0 0 0.9 0.335 0.93

ALOX5 IL1B 9606.ENSP00000363512 9606.ENSP00000263341 0 0 0 0 0.098 0 0 0.606 0.629

ALOX5 SELP 9606.ENSP00000363512 9606.ENSP00000263686 0 0 0 0 0.14 0 0 0.335 0.403

ALOX5 ICAM1 9606.ENSP00000363512 9606.ENSP00000264832 0 0 0 0 0.06 0 0 0.408 0.419

ALOX5 TP53 9606.ENSP00000363512 9606.ENSP00000269305 0 0 0 0 0 0 0 0.475 0.475

ALOX5 PPARG 9606.ENSP00000363512 9606.ENSP00000287820 0 0 0 0 0 0 0 0.676 0.676

ALOX5 VCAM1 9606.ENSP00000363512 9606.ENSP00000294728 0 0 0 0 0.062 0 0 0.392 0.405

ALOX5 IL13 9606.ENSP00000363512 9606.ENSP00000304915 0 0 0 0 0 0 0.9 0.469 0.944

ALOX5 CXCL8 9606.ENSP00000363512 9606.ENSP00000306512 0 0 0 0 0 0 0 0.506 0.506

ALOX5 MMP9 9606.ENSP00000363512 9606.ENSP00000361405 0 0 0 0 0.159 0 0 0.459 0.526

ALOX5 TLR4 9606.ENSP00000363512 9606.ENSP00000363089 0 0 0 0 0.142 0 0 0.431 0.491

ALOX5 VEGFA 9606.ENSP00000363512 9606.ENSP00000478570 0 0 0 0 0 0 0 0.454 0.454

ALOX5 IL10 9606.ENSP00000363512 9606.ENSP00000412237 0 0 0 0 0 0 0 0.517 0.517

ALOX5 CCL3 9606.ENSP00000363512 9606.ENSP00000477908 0 0 0 0 0.111 0 0 0.485 0.522

ALOX5 IL6 9606.ENSP00000363512 9606.ENSP00000385675 0 0 0 0 0 0 0 0.601 0.601

ALOX5 ITGB2 9606.ENSP00000363512 9606.ENSP00000380948 0 0 0 0 0.548 0 0 0.207 0.626

ALOX5 TNF 9606.ENSP00000363512 9606.ENSP00000398698 0 0 0 0 0.121 0 0 0.62 0.652

APOA1 CETP 9606.ENSP00000236850 9606.ENSP00000200676 0 0 0 0 0.065 0.213 0.72 0.877 0.971

APOA1 APOH 9606.ENSP00000236850 9606.ENSP00000205948 0 0 0 0 0.845 0.386 0.72 0.663 0.989

APOA1 PON1 9606.ENSP00000236850 9606.ENSP00000222381 0 0 0 0 0.097 0.213 0.72 0.987 0.997

APOA1 MPO 9606.ENSP00000236850 9606.ENSP00000225275 0 0 0 0 0 0 0 0.959 0.959

APOA1 CCL2 9606.ENSP00000236850 9606.ENSP00000225831 0 0 0 0 0 0 0 0.519 0.519

APOA1 LTF 9606.ENSP00000236850 9606.ENSP00000231751 0 0 0 0 0.145 0 0.5 0.281 0.665

APOA1 APOB 9606.ENSP00000236850 9606.ENSP00000233242 0 0 0 0 0.793 0.698 0.72 0.928 0.998

APOA1 CAT 9606.ENSP00000236850 9606.ENSP00000241052 0 0 0 0 0.062 0 0 0.391 0.404

APOA1 IGF1 9606.ENSP00000236850 9606.ENSP00000302665 0 0 0 0 0.063 0 0 0.392 0.405

APOA1 MMP9 9606.ENSP00000236850 9606.ENSP00000361405 0 0 0 0 0.062 0 0 0.393 0.406

APOA1 IL10 9606.ENSP00000236850 9606.ENSP00000412237 0 0 0 0 0 0 0 0.408 0.408

APOA1 SELP 9606.ENSP00000236850 9606.ENSP00000263686 0 0 0 0 0.062 0.135 0 0.338 0.415

APOA1 REN 9606.ENSP00000236850 9606.ENSP00000272190 0 0 0 0 0.085 0 0 0.391 0.419

APOA1 CXCL8 9606.ENSP00000236850 9606.ENSP00000306512 0 0 0 0 0 0 0 0.42 0.42

APOA1 CD40LG 9606.ENSP00000236850 9606.ENSP00000359663 0 0 0 0 0 0.27 0 0.244 0.424

APOA1 HBA2 9606.ENSP00000236850 9606.ENSP00000251595 0 0 0 0 0.051 0 0 0.465 0.47

APOA1 SELE 9606.ENSP00000236850 9606.ENSP00000331736 0 0 0 0 0.049 0.135 0 0.42 0.481

APOA1 HBA1 9606.ENSP00000236850 9606.ENSP00000322421 0 0 0 0 0.079 0 0 0.467 0.488

APOA1 IL1B 9606.ENSP00000236850 9606.ENSP00000263341 0 0 0 0 0 0 0 0.517 0.517

APOA1 ICAM1 9606.ENSP00000236850 9606.ENSP00000264832 0 0 0 0 0 0 0 0.518 0.518

APOA1 CALCA 9606.ENSP00000236850 9606.ENSP00000331746 0 0 0 0 0 0 0.5 0.138 0.55

APOA1 SHBG 9606.ENSP00000236850 9606.ENSP00000369816 0 0 0 0 0.07 0 0 0.557 0.57

APOA1 NR1H2 9606.ENSP00000236850 9606.ENSP00000253727 0 0 0 0 0.062 0 0 0.57 0.579

APOA1 TLR4 9606.ENSP00000236850 9606.ENSP00000363089 0 0 0 0 0.062 0 0 0.589 0.598

APOA1 LYZ 9606.ENSP00000236850 9606.ENSP00000261267 0 0 0 0 0.076 0 0.3 0.432 0.6

APOA1 TNF 9606.ENSP00000236850 9606.ENSP00000398698 0 0 0 0 0 0 0 0.606 0.606

APOA1 LEP 9606.ENSP00000236850 9606.ENSP00000312652 0 0 0 0 0 0 0 0.609 0.609

APOA1 IL6 9606.ENSP00000236850 9606.ENSP00000385675 0 0 0 0 0 0 0 0.612 0.612

APOA1 VCAM1 9606.ENSP00000236850 9606.ENSP00000294728 0 0 0 0 0.062 0.13 0 0.565 0.614

APOA1 GPT 9606.ENSP00000236850 9606.ENSP00000378408 0 0 0 0 0.073 0 0 0.604 0.617

APOA1 SREBF1 9606.ENSP00000236850 9606.ENSP00000348069 0 0 0 0 0.062 0 0 0.619 0.628

APOA1 HMGCR 9606.ENSP00000236850 9606.ENSP00000287936 0 0 0 0 0 0 0 0.63 0.63

APOA1 PPARG 9606.ENSP00000236850 9606.ENSP00000287820 0 0 0 0 0.062 0 0 0.668 0.676

APOA1 C3 9606.ENSP00000236850 9606.ENSP00000245907 0 0 0 0 0.111 0 0 0.655 0.681

APOA1 NOS3 9606.ENSP00000236850 9606.ENSP00000297494 0 0 0 0 0 0 0 0.736 0.736

APOA1 ORM1 9606.ENSP00000236850 9606.ENSP00000259396 0 0 0 0 0.572 0 0 0.577 0.811

APOA1 CRP 9606.ENSP00000236850 9606.ENSP00000255030 0 0 0 0 0.088 0 0 0.804 0.813

APOA1 SNCA 9606.ENSP00000236850 9606.ENSP00000338345 0 0 0 0 0 0.486 0.5 0.342 0.816

APOA1 INS 9606.ENSP00000236850 9606.ENSP00000380432 0 0 0 0 0.062 0 0.5 0.759 0.877

APOA1 F2 9606.ENSP00000236850 9606.ENSP00000308541 0 0 0 0 0.801 0 0 0.519 0.9

APOA1 JUN 9606.ENSP00000236850 9606.ENSP00000360266 0 0 0 0 0 0 0.9 0.265 0.923

APOA1 P4HB 9606.ENSP00000236850 9606.ENSP00000327801 0 0 0 0 0.062 0 0.9 0.293 0.927

APOA1 LPL 9606.ENSP00000236850 9606.ENSP00000309757 0 0 0 0 0 0 0.9 0.809 0.98

APOA1 MTTP 9606.ENSP00000236850 9606.ENSP00000427679 0 0 0 0 0.548 0 0.9 0.635 0.982

APOA1 APOE 9606.ENSP00000236850 9606.ENSP00000252486 0 0 0 0 0.205 0.371 0.8 0.981 0.997

APOB CETP 9606.ENSP00000233242 9606.ENSP00000200676 0 0 0 0 0.066 0 0.9 0.892 0.989

APOB APOH 9606.ENSP00000233242 9606.ENSP00000205948 0 0 0 0 0.812 0 0.72 0.608 0.977

APOB PON1 9606.ENSP00000233242 9606.ENSP00000222381 0 0 0 0 0.08 0 0.72 0.73 0.924

APOB MPO 9606.ENSP00000233242 9606.ENSP00000225275 0 0 0 0 0.062 0 0 0.525 0.535

APOB CCL2 9606.ENSP00000233242 9606.ENSP00000225831 0 0 0 0 0 0 0 0.517 0.517

APOB CXCL8 9606.ENSP00000233242 9606.ENSP00000306512 0 0 0 0 0 0 0 0.401 0.4

APOB BRCA1 9606.ENSP00000233242 9606.ENSP00000418960 0 0 0 0 0.088 0 0 0.37 0.401

APOB VEGFA 9606.ENSP00000233242 9606.ENSP00000478570 0 0 0 0 0.061 0 0 0.391 0.403

APOB CTSD 9606.ENSP00000233242 9606.ENSP00000236671 0 0 0 0 0.062 0.178 0 0.295 0.409

APOB HBA1 9606.ENSP00000233242 9606.ENSP00000322421 0 0 0 0 0.07 0 0 0.391 0.409

APOB CAT 9606.ENSP00000233242 9606.ENSP00000241052 0 0 0 0 0.062 0 0 0.405 0.418

APOB IL10 9606.ENSP00000233242 9606.ENSP00000412237 0 0 0 0 0 0 0 0.42 0.42

APOB IGF1 9606.ENSP00000233242 9606.ENSP00000302665 0 0 0 0 0.063 0 0 0.409 0.422

APOB SELP 9606.ENSP00000233242 9606.ENSP00000263686 0 0 0 0 0.062 0 0 0.414 0.426

APOB FURIN 9606.ENSP00000233242 9606.ENSP00000483552 0 0 0 0 0.062 0 0 0.422 0.435

APOB NR1H2 9606.ENSP00000233242 9606.ENSP00000253727 0 0 0 0 0.062 0 0 0.448 0.46

APOB IL1B 9606.ENSP00000233242 9606.ENSP00000263341 0 0 0 0 0 0 0 0.47 0.47

APOB SELE 9606.ENSP00000233242 9606.ENSP00000331736 0 0 0 0 0.049 0 0 0.469 0.473

APOB NOS3 9606.ENSP00000233242 9606.ENSP00000297494 0 0 0 0 0 0 0 0.507 0.507

APOB ICAM1 9606.ENSP00000233242 9606.ENSP00000264832 0 0 0 0 0 0 0 0.518 0.518

APOB C3 9606.ENSP00000233242 9606.ENSP00000245907 0 0 0 0 0.166 0 0 0.47 0.539

APOB REN 9606.ENSP00000233242 9606.ENSP00000272190 0 0 0 0 0.118 0.178 0 0.452 0.568

APOB VCAM1 9606.ENSP00000233242 9606.ENSP00000294728 0 0 0 0 0.061 0 0 0.565 0.574

APOB TNF 9606.ENSP00000233242 9606.ENSP00000398698 0 0 0 0 0 0 0 0.574 0.574

APOB ORM1 9606.ENSP00000233242 9606.ENSP00000259396 0 0 0 0 0.412 0 0 0.334 0.591

APOB CYP3A4 9606.ENSP00000233242 9606.ENSP00000337915 0 0 0 0 0.19 0 0 0.517 0.592

APOB SHBG 9606.ENSP00000233242 9606.ENSP00000369816 0 0 0 0 0 0 0 0.595 0.595

APOB PPARG 9606.ENSP00000233242 9606.ENSP00000287820 0 0 0 0 0.062 0 0 0.61 0.618

APOB IL6 9606.ENSP00000233242 9606.ENSP00000385675 0 0 0 0 0 0 0 0.619 0.619

APOB HSPA5 9606.ENSP00000233242 9606.ENSP00000324173 0 0 0 0 0 0.494 0 0.322 0.642

APOB GPT 9606.ENSP00000233242 9606.ENSP00000378408 0 0 0 0 0.065 0 0 0.668 0.676

APOB LEP 9606.ENSP00000233242 9606.ENSP00000312652 0 0 0 0 0 0 0 0.681 0.681

APOB HMGCR 9606.ENSP00000233242 9606.ENSP00000287936 0 0 0 0 0 0 0 0.715 0.715

APOB SREBF1 9606.ENSP00000233242 9606.ENSP00000348069 0 0 0 0 0.062 0 0 0.72 0.726

APOB VCP 9606.ENSP00000233242 9606.ENSP00000351777 0 0 0 0 0 0.686 0 0.207 0.74

APOB TLR4 9606.ENSP00000233242 9606.ENSP00000363089 0 0 0 0 0 0.057 0.6 0.453 0.775

APOB F2 9606.ENSP00000233242 9606.ENSP00000308541 0 0 0 0 0.704 0.056 0 0.466 0.837

APOB INS 9606.ENSP00000233242 9606.ENSP00000380432 0 0 0 0 0 0 0 0.856 0.856

APOB ITGB2 9606.ENSP00000233242 9606.ENSP00000380948 0 0 0 0 0 0 0.9 0.064 0.902

APOB CRP 9606.ENSP00000233242 9606.ENSP00000255030 0 0 0 0 0.082 0 0 0.902 0.906

APOB P4HB 9606.ENSP00000233242 9606.ENSP00000327801 0 0 0 0 0.062 0 0.9 0.843 0.983

APOB LPL 9606.ENSP00000233242 9606.ENSP00000309757 0 0 0 0 0 0 0.9 0.931 0.992

APOB APOE 9606.ENSP00000233242 9606.ENSP00000252486 0 0 0 0 0.321 0.385 0.72 0.991 0.998

APOB MTTP 9606.ENSP00000233242 9606.ENSP00000427679 0 0 0 0 0.546 0.735 0.9 0.99 0.999

APOE CETP 9606.ENSP00000252486 9606.ENSP00000200676 0 0 0 0 0.062 0 0.72 0.827 0.95

APOE APOH 9606.ENSP00000252486 9606.ENSP00000205948 0 0 0 0 0.062 0.369 0.72 0.595 0.923

APOE HMOX1 9606.ENSP00000252486 9606.ENSP00000216117 0 0 0 0 0.062 0 0 0.518 0.528

APOE NFKBIA 9606.ENSP00000252486 9606.ENSP00000216797 0 0 0 0 0 0.146 0 0.39 0.456

APOE MMP2 9606.ENSP00000252486 9606.ENSP00000219070 0 0 0 0 0.076 0.075 0 0.466 0.503

APOE TGFB1 9606.ENSP00000252486 9606.ENSP00000221930 0 0 0 0 0 0 0 0.402 0.402

APOE PON1 9606.ENSP00000252486 9606.ENSP00000222381 0 0 0 0 0.062 0 0.72 0.743 0.926

APOE MPO 9606.ENSP00000252486 9606.ENSP00000225275 0 0 0 0 0 0 0 0.472 0.472

APOE CCL2 9606.ENSP00000252486 9606.ENSP00000225831 0 0 0 0 0.065 0 0 0.69 0.697

APOE IFNG 9606.ENSP00000252486 9606.ENSP00000229135 0 0 0 0 0 0 0 0.511 0.511

APOE IL4 9606.ENSP00000252486 9606.ENSP00000231449 0 0 0 0 0 0 0 0.468 0.468

APOE CTSD 9606.ENSP00000252486 9606.ENSP00000236671 0 0 0 0 0.085 0 0 0.584 0.604

APOE CAT 9606.ENSP00000252486 9606.ENSP00000241052 0 0 0 0 0.062 0 0 0.515 0.525

APOE C3 9606.ENSP00000252486 9606.ENSP00000245907 0 0 0 0 0.138 0 0 0.563 0.607

APOE FGF2 9606.ENSP00000252486 9606.ENSP00000264498 0 0 0 0 0 0 0 0.402 0.402

APOE CSF2 9606.ENSP00000252486 9606.ENSP00000296871 0 0 0 0 0 0 0 0.403 0.403

APOE HIF1A 9606.ENSP00000252486 9606.ENSP00000437955 0 0 0 0 0.062 0 0 0.391 0.404

APOE CCL3 9606.ENSP00000252486 9606.ENSP00000477908 0 0 0 0 0 0 0 0.405 0.405

APOE NOS2 9606.ENSP00000252486 9606.ENSP00000327251 0 0 0 0 0.069 0 0 0.39 0.408

APOE VDR 9606.ENSP00000252486 9606.ENSP00000447173 0 0 0 0 0 0 0 0.417 0.416

APOE LCN2 9606.ENSP00000252486 9606.ENSP00000362108 0 0 0 0 0 0 0 0.418 0.418

APOE VCP 9606.ENSP00000252486 9606.ENSP00000351777 0 0 0 0 0 0.128 0 0.363 0.421

APOE IL17A 9606.ENSP00000252486 9606.ENSP00000344192 0 0 0 0 0 0 0 0.424 0.424

APOE GRIN2B 9606.ENSP00000252486 9606.ENSP00000477455 0 0 0 0 0 0.081 0 0.416 0.44

APOE GPT 9606.ENSP00000252486 9606.ENSP00000378408 0 0 0 0 0 0 0 0.455 0.455

APOE TP53 9606.ENSP00000252486 9606.ENSP00000269305 0 0 0 0 0 0 0 0.459 0.459

APOE NGF 9606.ENSP00000252486 9606.ENSP00000358525 0 0 0 0 0 0 0 0.464 0.463

APOE ESR1 9606.ENSP00000252486 9606.ENSP00000405330 0 0 0 0 0 0 0 0.464 0.463

APOE CYP2C9 9606.ENSP00000252486 9606.ENSP00000260682 0 0 0 0 0.052 0 0 0.458 0.464

APOE CCL5 9606.ENSP00000252486 9606.ENSP00000474412 0 0 0 0 0 0 0 0.478 0.478

APOE EGF 9606.ENSP00000252486 9606.ENSP00000265171 0 0 0 0 0 0.056 0 0.471 0.479

APOE OCLN 9606.ENSP00000252486 9606.ENSP00000347379 0 0 0 0 0.062 0 0 0.474 0.486

APOE IL1A 9606.ENSP00000252486 9606.ENSP00000263339 0 0 0 0 0 0 0 0.502 0.502

APOE NCF1 9606.ENSP00000252486 9606.ENSP00000289473 0 0 0 0 0.062 0 0 0.491 0.503

APOE SOD1 9606.ENSP00000252486 9606.ENSP00000270142 0 0 0 0 0 0 0 0.507 0.507

APOE SLC6A4 9606.ENSP00000252486 9606.ENSP00000261707 0 0 0 0 0.062 0 0 0.504 0.514

APOE JUN 9606.ENSP00000252486 9606.ENSP00000360266 0 0 0 0 0 0 0 0.515 0.515

APOE CX3CR1 9606.ENSP00000252486 9606.ENSP00000351059 0 0 0 0 0 0 0 0.522 0.522

APOE SPP1 9606.ENSP00000252486 9606.ENSP00000378517 0 0 0 0 0.153 0 0 0.463 0.526

APOE EDN1 9606.ENSP00000252486 9606.ENSP00000368683 0 0 0 0 0 0 0 0.539 0.539

APOE F2 9606.ENSP00000252486 9606.ENSP00000308541 0 0 0 0 0.085 0 0 0.519 0.541

APOE CXCL8 9606.ENSP00000252486 9606.ENSP00000306512 0 0 0 0 0 0 0 0.548 0.548

APOE IGF1 9606.ENSP00000252486 9606.ENSP00000302665 0 0 0 0 0.062 0 0 0.562 0.571

APOE VEGFA 9606.ENSP00000252486 9606.ENSP00000478570 0 0 0 0 0 0 0 0.594 0.594

APOE SELP 9606.ENSP00000252486 9606.ENSP00000263686 0 0 0 0 0 0.125 0 0.567 0.605

APOE REN 9606.ENSP00000252486 9606.ENSP00000272190 0 0 0 0 0 0 0 0.606 0.606

APOE SELE 9606.ENSP00000252486 9606.ENSP00000331736 0 0 0 0 0 0.125 0 0.57 0.608

APOE CASP3 9606.ENSP00000252486 9606.ENSP00000311032 0 0 0 0 0 0 0 0.607 0.608

APOE SREBF1 9606.ENSP00000252486 9606.ENSP00000348069 0 0 0 0 0.062 0 0 0.615 0.623

APOE LEP 9606.ENSP00000252486 9606.ENSP00000312652 0 0 0 0 0 0 0 0.628 0.628

APOE IL10 9606.ENSP00000252486 9606.ENSP00000412237 0 0 0 0 0 0 0 0.646 0.646

APOE MTTP 9606.ENSP00000252486 9606.ENSP00000427679 0 0 0 0 0.064 0 0 0.643 0.652

APOE HMGCR 9606.ENSP00000252486 9606.ENSP00000287936 0 0 0 0 0 0 0 0.662 0.662

APOE MMP9 9606.ENSP00000252486 9606.ENSP00000361405 0 0 0 0 0 0.075 0 0.665 0.677

APOE ICAM1 9606.ENSP00000252486 9606.ENSP00000264832 0 0 0 0 0.062 0 0 0.673 0.68

APOE IL1B 9606.ENSP00000252486 9606.ENSP00000263341 0 0 0 0 0 0 0 0.688 0.688

APOE NOS1 9606.ENSP00000252486 9606.ENSP00000477999 0 0 0 0 0.052 0 0 0.697 0.7

APOE TNF 9606.ENSP00000252486 9606.ENSP00000398698 0 0 0 0 0 0 0 0.713 0.713

APOE IL6 9606.ENSP00000252486 9606.ENSP00000385675 0 0 0 0 0 0 0 0.714 0.714

APOE NOS3 9606.ENSP00000252486 9606.ENSP00000297494 0 0 0 0 0.062 0 0 0.71 0.716

APOE PPARG 9606.ENSP00000252486 9606.ENSP00000287820 0 0 0 0 0 0 0 0.724 0.724

APOE CRP 9606.ENSP00000252486 9606.ENSP00000255030 0 0 0 0 0 0 0 0.728 0.728

APOE INS 9606.ENSP00000252486 9606.ENSP00000380432 0 0 0 0 0 0 0 0.739 0.739

APOE TLR4 9606.ENSP00000252486 9606.ENSP00000363089 0 0 0 0 0 0 0 0.756 0.756

APOE VCAM1 9606.ENSP00000252486 9606.ENSP00000294728 0 0 0 0 0.069 0.043 0 0.756 0.764

APOE BDNF 9606.ENSP00000252486 9606.ENSP00000414303 0 0 0 0 0 0 0 0.815 0.815

APOE MMP3 9606.ENSP00000252486 9606.ENSP00000299855 0 0 0 0 0 0.689 0 0.445 0.82

APOE NR1H2 9606.ENSP00000252486 9606.ENSP00000253727 0 0 0 0 0.053 0 0.9 0.562 0.954

APOE SNCA 9606.ENSP00000252486 9606.ENSP00000338345 0 0 0 0 0.062 0.878 0 0.761 0.97

APOE LPL 9606.ENSP00000252486 9606.ENSP00000309757 0 0 0 0 0.098 0 0.9 0.869 0.987

APOH CETP 9606.ENSP00000205948 9606.ENSP00000200676 0 0 0 0 0 0 0.72 0.323 0.802

APOH MPO 9606.ENSP00000205948 9606.ENSP00000225275 0 0 0 0 0 0.056 0 0.391 0.4

APOH THBD 9606.ENSP00000205948 9606.ENSP00000366307 0 0 0 0 0 0 0 0.492 0.492

APOH CRP 9606.ENSP00000205948 9606.ENSP00000255030 0 0 0 0 0.13 0 0 0.576 0.616

APOH PF4 9606.ENSP00000205948 9606.ENSP00000296029 0 0 0 0 0 0.297 0 0.519 0.648

APOH C3 9606.ENSP00000205948 9606.ENSP00000245907 0 0 0 0 0.111 0 0 0.635 0.662

APOH TLR4 9606.ENSP00000205948 9606.ENSP00000363089 0 0 0 0 0 0.056 0 0.746 0.75

APOH ORM1 9606.ENSP00000205948 9606.ENSP00000259396 0 0 0 0 0.565 0 0 0.452 0.751

APOH LPL 9606.ENSP00000205948 9606.ENSP00000309757 0 0 0 0 0 0 0.8 0.282 0.85

APOH PON1 9606.ENSP00000205948 9606.ENSP00000222381 0 0 0 0 0.161 0 0.72 0.425 0.853

APOH F2 9606.ENSP00000205948 9606.ENSP00000308541 0 0 0 0 0.847 0 0 0.845 0.975

AQP2 CFTR 9606.ENSP00000199280 9606.ENSP00000003084 0 0 0 0 0 0.059 0 0.483 0.492

AQP2 AQP8 9606.ENSP00000199280 9606.ENSP00000219660 0 0 0.447 0.764 0 0 0.6 0.852 0.711

AQP2 REN 9606.ENSP00000199280 9606.ENSP00000272190 0 0 0 0 0.085 0 0 0.71 0.724

AQP8 EDNRA 9606.ENSP00000219660 9606.ENSP00000315011 0 0 0 0 0 0 0 0.404 0.404

ATP13A2 CTSD 9606.ENSP00000327214 9606.ENSP00000236671 0 0 0 0 0.106 0 0 0.552 0.582

ATP13A2 SOD1 9606.ENSP00000327214 9606.ENSP00000270142 0 0 0 0 0.062 0.149 0 0.339 0.426

ATP13A2 VCP 9606.ENSP00000327214 9606.ENSP00000351777 0 0 0 0 0 0.102 0 0.373 0.413

ATP13A2 TH 9606.ENSP00000327214 9606.ENSP00000370571 0 0 0 0 0 0 0 0.414 0.414

ATP13A2 CLN3 9606.ENSP00000327214 9606.ENSP00000454229 0 0 0 0 0 0 0 0.64 0.64

ATP13A2 SNCA 9606.ENSP00000327214 9606.ENSP00000338345 0 0 0 0 0 0 0 0.847 0.847

ATP1A2 SCN1A 9606.ENSP00000354490 9606.ENSP00000303540 0 0 0 0 0.207 0.173 0 0.807 0.862

ATP1A2 ITPR1 9606.ENSP00000354490 9606.ENSP00000306253 0 0 0 0 0.065 0.23 0 0.236 0.402

ATP1A2 RYR1 9606.ENSP00000354490 9606.ENSP00000352608 0 0 0 0 0.144 0.165 0 0.235 0.405

ATP1A2 CACNA1A 9606.ENSP00000354490 9606.ENSP00000353362 0 0 0 0 0.211 0.16 0 0.846 0.889

ATP1A2 KCNJ10 9606.ENSP00000354490 9606.ENSP00000357068 0 0 0 0 0.219 0 0 0.476 0.573

BAX HMOX1 9606.ENSP00000293288 9606.ENSP00000216117 0 0 0 0 0.063 0 0 0.503 0.514

BAX CAT 9606.ENSP00000293288 9606.ENSP00000241052 0 0 0 0 0 0 0 0.518 0.518

BAX MDM2 9606.ENSP00000293288 9606.ENSP00000258149 0 0 0 0 0 0.056 0 0.455 0.463

BAX IL1B 9606.ENSP00000293288 9606.ENSP00000263341 0 0 0 0 0.049 0 0 0.465 0.469

BAX TP53 9606.ENSP00000293288 9606.ENSP00000269305 0 0 0 0 0.051 0.738 0.9 0.681 0.991

BAX HIF1A 9606.ENSP00000293288 9606.ENSP00000437955 0 0 0 0 0 0.063 0 0.391 0.404

BAX PARP1 9606.ENSP00000293288 9606.ENSP00000355759 0 0 0 0 0 0 0 0.413 0.412

BAX PTEN 9606.ENSP00000293288 9606.ENSP00000361021 0 0 0 0 0 0.076 0 0.401 0.422

BAX MMP9 9606.ENSP00000293288 9606.ENSP00000361405 0 0 0 0 0 0 0 0.451 0.451

BAX FAS 9606.ENSP00000293288 9606.ENSP00000347979 0 0 0 0 0 0 0 0.452 0.452

BAX IL6 9606.ENSP00000293288 9606.ENSP00000385675 0 0 0 0 0 0 0 0.459 0.459

BAX HSPA5 9606.ENSP00000293288 9606.ENSP00000324173 0 0 0 0 0 0 0 0.459 0.459

BAX VEGFA 9606.ENSP00000293288 9606.ENSP00000478570 0 0 0 0 0 0 0 0.467 0.467

BAX TNF 9606.ENSP00000293288 9606.ENSP00000398698 0 0 0 0 0 0.059 0 0.457 0.467

BAX FASLG 9606.ENSP00000293288 9606.ENSP00000356694 0 0 0 0 0 0 0 0.468 0.468

BAX FADD 9606.ENSP00000293288 9606.ENSP00000301838 0 0 0 0 0.086 0 0 0.47 0.494

BAX SNCA 9606.ENSP00000293288 9606.ENSP00000338345 0 0 0 0 0 0.43 0 0.165 0.503

BAX CDKN1A 9606.ENSP00000293288 9606.ENSP00000384849 0 0 0 0 0.069 0 0 0.501 0.515

BAX MTOR 9606.ENSP00000293288 9606.ENSP00000354558 0 0 0 0 0 0.058 0 0.509 0.517

BAX JUN 9606.ENSP00000293288 9606.ENSP00000360266 0 0 0 0 0 0 0 0.558 0.558

BAX CASP8 9606.ENSP00000293288 9606.ENSP00000351273 0 0 0 0 0 0.374 0 0.688 0.796

BAX CASP9 9606.ENSP00000293288 9606.ENSP00000330237 0 0 0 0 0 0.374 0 0.746 0.834

BAX CASP3 9606.ENSP00000293288 9606.ENSP00000311032 0 0 0 0 0 0.374 0 0.845 0.899

BAX CYCS 9606.ENSP00000293288 9606.ENSP00000307786 0 0 0 0 0.042 0.281 0.9 0.767 0.981

BAX BCL2L1 9606.ENSP00000293288 9606.ENSP00000302564 0 0 0 0.643 0.047 0.913 0.9 0.709 0.993

BAX BCL2 9606.ENSP00000293288 9606.ENSP00000381185 0 0 0 0.645 0 0.981 0.9 0.758 0.998

BCL2 CTSD 9606.ENSP00000381185 9606.ENSP00000236671 0 0 0 0 0 0 0.6 0.061 0.608

BCL2 TP53 9606.ENSP00000381185 9606.ENSP00000269305 0 0 0 0 0.051 0.884 0.9 0.628 0.995

BCL2 SOD1 9606.ENSP00000381185 9606.ENSP00000270142 0 0 0 0 0 0.297 0.8 0.15 0.87

BCL2 BCL2L1 9606.ENSP00000381185 9606.ENSP00000302564 0 0 0 0.898 0 0.87 0.9 0.627 0.987

BCL2 IGF1 9606.ENSP00000381185 9606.ENSP00000302665 0 0 0 0 0 0 0 0.413 0.413

BCL2 ITPR1 9606.ENSP00000381185 9606.ENSP00000306253 0 0 0 0 0.077 0.486 0 0.138 0.555

BCL2 CYCS 9606.ENSP00000381185 9606.ENSP00000307786 0 0 0 0 0 0.225 0.9 0.619 0.967

BCL2 CASP3 9606.ENSP00000381185 9606.ENSP00000311032 0 0 0 0 0 0.539 0 0.622 0.818

BCL2 CASP9 9606.ENSP00000381185 9606.ENSP00000330237 0 0 0 0 0 0.178 0 0.511 0.581

BCL2 CASP8 9606.ENSP00000381185 9606.ENSP00000351273 0 0 0 0 0 0.726 0 0.432 0.838

BCL2 PARP1 9606.ENSP00000381185 9606.ENSP00000355759 0 0 0 0 0 0.27 0 0.268 0.443

BCL2 HIF1A 9606.ENSP00000381185 9606.ENSP00000437955 0 0 0 0 0 0.538 0 0.324 0.674

BCL2 ESR1 9606.ENSP00000381185 9606.ENSP00000405330 0 0 0 0 0.069 0.057 0.9 0.244 0.924

BCL2L1 TNFRSF1A 9606.ENSP00000302564 9606.ENSP00000162749 0 0 0 0 0 0 0 0.651 0.651

BCL2L1 HMOX1 9606.ENSP00000302564 9606.ENSP00000216117 0 0 0 0 0 0 0 0.566 0.566

BCL2L1 NFKBIA 9606.ENSP00000302564 9606.ENSP00000216797 0 0 0 0 0.062 0.056 0 0.686 0.698

BCL2L1 MMP2 9606.ENSP00000302564 9606.ENSP00000219070 0 0 0 0 0 0 0 0.556 0.556

BCL2L1 CCL2 9606.ENSP00000302564 9606.ENSP00000225831 0 0 0 0 0 0 0 0.457 0.457

BCL2L1 NFKB1 9606.ENSP00000302564 9606.ENSP00000226574 0 0 0 0 0 0.056 0 0.449 0.457

BCL2L1 IL2 9606.ENSP00000302564 9606.ENSP00000226730 0 0 0 0 0 0 0 0.621 0.621

BCL2L1 KITLG 9606.ENSP00000302564 9606.ENSP00000228280 0 0 0 0 0 0 0 0.426 0.426

BCL2L1 IFNG 9606.ENSP00000302564 9606.ENSP00000229135 0 0 0 0 0.052 0 0 0.484 0.49

BCL2L1 IL4 9606.ENSP00000302564 9606.ENSP00000231449 0 0 0 0 0 0 0 0.505 0.505

BCL2L1 NR3C1 9606.ENSP00000302564 9606.ENSP00000231509 0 0 0 0 0 0.149 0 0.341 0.415

BCL2L1 CTSD 9606.ENSP00000302564 9606.ENSP00000236671 0 0 0 0 0.076 0 0.6 0.325 0.728

BCL2L1 CAT 9606.ENSP00000302564 9606.ENSP00000241052 0 0 0 0 0 0 0 0.564 0.564

BCL2L1 HSPB1 9606.ENSP00000302564 9606.ENSP00000248553 0 0 0 0 0.062 0.099 0 0.493 0.534

BCL2L1 MDM2 9606.ENSP00000302564 9606.ENSP00000258149 0 0 0 0 0.054 0.176 0 0.712 0.756

BCL2L1 IL1B 9606.ENSP00000302564 9606.ENSP00000263341 0 0 0 0 0.049 0 0 0.601 0.604

BCL2L1 FGF2 9606.ENSP00000302564 9606.ENSP00000264498 0 0 0 0 0.053 0 0 0.504 0.51

BCL2L1 ICAM1 9606.ENSP00000302564 9606.ENSP00000264832 0 0 0 0 0 0 0 0.504 0.504

BCL2L1 EGF 9606.ENSP00000302564 9606.ENSP00000265171 0 0 0 0 0 0 0 0.754 0.754

BCL2L1 TP53 9606.ENSP00000302564 9606.ENSP00000269305 0 0 0 0 0.051 0.977 0.9 0.99 0.999

BCL2L1 ERBB2 9606.ENSP00000302564 9606.ENSP00000269571 0 0 0 0 0 0 0 0.683 0.683

BCL2L1 EGFR 9606.ENSP00000302564 9606.ENSP00000275493 0 0 0 0 0 0 0 0.686 0.686

BCL2L1 PPARG 9606.ENSP00000302564 9606.ENSP00000287820 0 0 0 0 0 0.057 0 0.456 0.465

BCL2L1 KIT 9606.ENSP00000302564 9606.ENSP00000288135 0 0 0 0 0 0 0 0.511 0.511

BCL2L1 CSF2 9606.ENSP00000302564 9606.ENSP00000296871 0 0 0 0 0 0 0 0.51 0.51

BCL2L1 NOS3 9606.ENSP00000302564 9606.ENSP00000297494 0 0 0 0 0 0 0 0.408 0.408

BCL2L1 FADD 9606.ENSP00000302564 9606.ENSP00000301838 0 0 0 0 0.065 0 0 0.739 0.746

BCL2L1 SOCS1 9606.ENSP00000302564 9606.ENSP00000329418 0 0 0 0 0 0 0 0.412 0.412

BCL2L1 IL17A 9606.ENSP00000302564 9606.ENSP00000344192 0 0 0 0 0 0 0 0.415 0.415

BCL2L1 LTA 9606.ENSP00000302564 9606.ENSP00000403495 0 0 0 0 0 0.059 0 0.426 0.436

BCL2L1 BRCA1 9606.ENSP00000302564 9606.ENSP00000418960 0 0 0 0 0 0 0 0.451 0.451

BCL2L1 TOP1 9606.ENSP00000302564 9606.ENSP00000354522 0 0 0 0 0 0.105 0 0.421 0.459

BCL2L1 BDNF 9606.ENSP00000302564 9606.ENSP00000414303 0 0 0 0 0 0 0 0.46 0.459

BCL2L1 TLR4 9606.ENSP00000302564 9606.ENSP00000363089 0 0 0 0 0.062 0 0 0.459 0.47

BCL2L1 MYD88 9606.ENSP00000302564 9606.ENSP00000401399 0 0 0 0 0 0.153 0 0.402 0.472

BCL2L1 NGF 9606.ENSP00000302564 9606.ENSP00000358525 0 0 0 0 0 0 0 0.475 0.475

BCL2L1 FOXP3 9606.ENSP00000302564 9606.ENSP00000365380 0 0 0 0 0 0.076 0 0.457 0.476

BCL2L1 PRKCD 9606.ENSP00000302564 9606.ENSP00000378217 0 0 0 0 0 0 0 0.499 0.499

BCL2L1 CXCR4 9606.ENSP00000302564 9606.ENSP00000386884 0 0 0 0 0 0 0 0.503 0.503

BCL2L1 CD40LG 9606.ENSP00000302564 9606.ENSP00000359663 0 0 0 0 0 0 0 0.52 0.52

BCL2L1 CXCL8 9606.ENSP00000302564 9606.ENSP00000306512 0 0 0 0 0 0 0 0.53 0.53

BCL2L1 CHUK 9606.ENSP00000302564 9606.ENSP00000359424 0 0 0 0 0 0 0 0.557 0.557

BCL2L1 CD40 9606.ENSP00000302564 9606.ENSP00000361359 0 0 0 0 0.049 0 0 0.559 0.562

BCL2L1 HSPA5 9606.ENSP00000302564 9606.ENSP00000324173 0 0 0 0 0 0 0 0.562 0.562

BCL2L1 CD28 9606.ENSP00000302564 9606.ENSP00000324890 0 0 0 0 0 0 0 0.563 0.563

BCL2L1 ESR1 9606.ENSP00000302564 9606.ENSP00000405330 0 0 0 0 0 0.057 0 0.559 0.566

BCL2L1 IGF1 9606.ENSP00000302564 9606.ENSP00000302665 0 0 0 0 0 0 0 0.566 0.566

BCL2L1 IL10 9606.ENSP00000302564 9606.ENSP00000412237 0 0 0 0 0 0 0 0.568 0.568

BCL2L1 INS 9606.ENSP00000302564 9606.ENSP00000380432 0 0 0 0 0 0 0 0.575 0.575

BCL2L1 HDAC1 9606.ENSP00000302564 9606.ENSP00000362649 0 0 0 0 0 0 0 0.582 0.582

BCL2L1 STAT1 9606.ENSP00000302564 9606.ENSP00000354394 0 0 0 0 0 0.069 0 0.608 0.62

BCL2L1 PARP1 9606.ENSP00000302564 9606.ENSP00000355759 0 0 0 0 0 0 0 0.651 0.651

BCL2L1 MMP9 9606.ENSP00000302564 9606.ENSP00000361405 0 0 0 0 0 0 0 0.658 0.658

BCL2L1 HIF1A 9606.ENSP00000302564 9606.ENSP00000437955 0 0 0 0 0 0.139 0 0.637 0.674

BCL2L1 CDKN1A 9606.ENSP00000302564 9606.ENSP00000384849 0 0 0 0 0.076 0 0 0.662 0.674

BCL2L1 IL6 9606.ENSP00000302564 9606.ENSP00000385675 0 0 0 0 0 0 0 0.687 0.687

BCL2L1 FAS 9606.ENSP00000302564 9606.ENSP00000347979 0 0 0 0 0 0 0 0.691 0.692

BCL2L1 ITPR3 9606.ENSP00000302564 9606.ENSP00000363435 0 0 0 0 0.076 0 0 0.689 0.701

BCL2L1 VEGFA 9606.ENSP00000302564 9606.ENSP00000478570 0 0 0 0 0 0 0 0.701 0.701

BCL2L1 JUN 9606.ENSP00000302564 9606.ENSP00000360266 0 0 0 0 0.062 0 0 0.697 0.704

BCL2L1 TNF 9606.ENSP00000302564 9606.ENSP00000398698 0 0 0 0 0 0.059 0 0.711 0.716

BCL2L1 PTEN 9606.ENSP00000302564 9606.ENSP00000361021 0 0 0 0 0 0.076 0 0.712 0.723

BCL2L1 FASLG 9606.ENSP00000302564 9606.ENSP00000356694 0 0 0 0 0 0 0 0.744 0.744

BCL2L1 SNCA 9606.ENSP00000302564 9606.ENSP00000338345 0 0 0 0 0.12 0.193 0 0.693 0.763

BCL2L1 MTOR 9606.ENSP00000302564 9606.ENSP00000354558 0 0 0 0 0.053 0.495 0 0.721 0.855

BCL2L1 CASP3 9606.ENSP00000302564 9606.ENSP00000311032 0 0 0 0 0 0.178 0 0.9 0.915

BCL2L1 STAT5A 9606.ENSP00000302564 9606.ENSP00000341208 0 0 0 0 0 0.069 0.9 0.689 0.968

BCL2L1 CASP9 9606.ENSP00000302564 9606.ENSP00000330237 0 0 0 0 0.056 0.724 0 0.905 0.973

BCL2L1 CASP8 9606.ENSP00000302564 9606.ENSP00000351273 0 0 0 0 0.052 0.727 0 0.916 0.976

BCL2L1 ITPR1 9606.ENSP00000302564 9606.ENSP00000306253 0 0 0 0 0.074 0 0 0.98 0.981

BCL2L1 CYCS 9606.ENSP00000302564 9606.ENSP00000307786 0 0 0 0 0 0.681 0.9 0.906 0.996

BDNF TNFRSF1A 9606.ENSP00000414303 9606.ENSP00000162749 0 0 0 0 0 0 0 0.403 0.403

BDNF HMOX1 9606.ENSP00000414303 9606.ENSP00000216117 0 0 0 0 0 0 0 0.587 0.587

BDNF MMP2 9606.ENSP00000414303 9606.ENSP00000219070 0 0 0 0 0.06 0 0 0.391 0.403

BDNF TGFB1 9606.ENSP00000414303 9606.ENSP00000221930 0 0 0 0 0 0 0 0.47 0.47

BDNF CCL2 9606.ENSP00000414303 9606.ENSP00000225831 0 0 0 0 0 0 0 0.564 0.564

BDNF IL2 9606.ENSP00000414303 9606.ENSP00000226730 0 0 0 0 0 0 0 0.461 0.461

BDNF KITLG 9606.ENSP00000414303 9606.ENSP00000228280 0 0 0 0 0.088 0 0 0.419 0.448

BDNF IFNG 9606.ENSP00000414303 9606.ENSP00000229135 0 0 0 0 0 0 0 0.481 0.481

BDNF IL4 9606.ENSP00000414303 9606.ENSP00000231449 0 0 0 0 0 0 0 0.551 0.551

BDNF NR3C1 9606.ENSP00000414303 9606.ENSP00000231509 0 0 0 0 0 0 0 0.687 0.688

BDNF CAT 9606.ENSP00000414303 9606.ENSP00000241052 0 0 0 0 0 0 0 0.53 0.53

BDNF CRP 9606.ENSP00000414303 9606.ENSP00000255030 0 0 0 0 0 0 0 0.556 0.556

BDNF SLC6A4 9606.ENSP00000414303 9606.ENSP00000261707 0 0 0 0 0.049 0 0 0.91 0.911

BDNF IL1A 9606.ENSP00000414303 9606.ENSP00000263339 0 0 0 0 0 0 0 0.461 0.461

BDNF IL1B 9606.ENSP00000414303 9606.ENSP00000263341 0 0 0 0 0 0 0 0.734 0.734

BDNF FGF2 9606.ENSP00000414303 9606.ENSP00000264498 0 0 0 0 0.11 0 0 0.857 0.867

BDNF FGF10 9606.ENSP00000414303 9606.ENSP00000264664 0 0 0 0 0 0 0 0.474 0.474

BDNF ICAM1 9606.ENSP00000414303 9606.ENSP00000264832 0 0 0 0 0 0 0 0.416 0.416

BDNF EGF 9606.ENSP00000414303 9606.ENSP00000265171 0 0 0 0 0.072 0 0 0.73 0.739

BDNF ERBB3 9606.ENSP00000414303 9606.ENSP00000267101 0 0 0 0 0 0 0.6 0.275 0.697

BDNF TP53 9606.ENSP00000414303 9606.ENSP00000269305 0 0 0 0 0 0 0.9 0.51 0.948

BDNF ERBB2 9606.ENSP00000414303 9606.ENSP00000269571 0 0 0 0 0 0 0.6 0.48 0.783

BDNF SOD1 9606.ENSP00000414303 9606.ENSP00000270142 0 0 0 0 0 0 0 0.565 0.565

BDNF EGFR 9606.ENSP00000414303 9606.ENSP00000275493 0 0 0 0 0.132 0 0.6 0.46 0.796

BDNF CRH 9606.ENSP00000414303 9606.ENSP00000276571 0 0 0 0 0.094 0 0 0.73 0.745

BDNF SST 9606.ENSP00000414303 9606.ENSP00000287641 0 0 0 0 0.065 0 0 0.573 0.584

BDNF PPARG 9606.ENSP00000414303 9606.ENSP00000287820 0 0 0 0 0 0 0 0.502 0.502

BDNF KIT 9606.ENSP00000414303 9606.ENSP00000288135 0 0 0 0 0 0 0.6 0.29 0.703

BDNF CSF2 9606.ENSP00000414303 9606.ENSP00000296871 0 0 0 0 0 0 0 0.407 0.407

BDNF NOS3 9606.ENSP00000414303 9606.ENSP00000297494 0 0 0 0 0 0.213 0 0.472 0.567

BDNF MMP3 9606.ENSP00000414303 9606.ENSP00000299855 0 0 0 0 0 0 0.9 0.351 0.932

BDNF IGF1 9606.ENSP00000414303 9606.ENSP00000302665 0 0 0 0 0 0 0 0.904 0.904

BDNF INSR 9606.ENSP00000414303 9606.ENSP00000303830 0 0 0 0 0 0.056 0.6 0.217 0.678

BDNF IL13 9606.ENSP00000414303 9606.ENSP00000304915 0 0 0 0 0 0 0 0.429 0.429

BDNF CXCL8 9606.ENSP00000414303 9606.ENSP00000306512 0 0 0 0 0 0 0 0.518 0.518

BDNF CYCS 9606.ENSP00000414303 9606.ENSP00000307786 0 0 0 0 0 0 0 0.507 0.507

BDNF CASP3 9606.ENSP00000414303 9606.ENSP00000311032 0 0 0 0 0 0 0 0.715 0.715

BDNF LEP 9606.ENSP00000414303 9606.ENSP00000312652 0 0 0 0 0 0 0 0.7 0.701

BDNF HSPA5 9606.ENSP00000414303 9606.ENSP00000324173 0 0 0 0 0 0 0 0.429 0.429

BDNF CASP9 9606.ENSP00000414303 9606.ENSP00000330237 0 0 0 0 0 0 0 0.418 0.418

BDNF CALCA 9606.ENSP00000414303 9606.ENSP00000331746 0 0 0 0 0.083 0 0 0.675 0.689

BDNF SNCA 9606.ENSP00000414303 9606.ENSP00000338345 0 0 0 0 0.083 0 0 0.667 0.681

BDNF IL17A 9606.ENSP00000414303 9606.ENSP00000344192 0 0 0 0 0 0 0 0.423 0.422

BDNF RET 9606.ENSP00000414303 9606.ENSP00000347942 0 0 0 0 0 0 0 0.438 0.438

BDNF NR3C2 9606.ENSP00000414303 9606.ENSP00000350815 0 0 0 0 0 0 0 0.427 0.427

BDNF CX3CR1 9606.ENSP00000414303 9606.ENSP00000351059 0 0 0 0 0 0 0 0.495 0.495

BDNF DNMT1 9606.ENSP00000414303 9606.ENSP00000352516 0 0 0 0 0 0 0 0.558 0.558

BDNF MTOR 9606.ENSP00000414303 9606.ENSP00000354558 0 0 0 0 0 0 0 0.702 0.702

BDNF NGF 9606.ENSP00000414303 9606.ENSP00000358525 0 0 0 0.841 0.1 0 0.9 0.958 0.92

BDNF JUN 9606.ENSP00000414303 9606.ENSP00000360266 0 0 0 0 0 0 0 0.61 0.61

BDNF PTEN 9606.ENSP00000414303 9606.ENSP00000361021 0 0 0 0 0 0 0 0.505 0.505

BDNF MMP9 9606.ENSP00000414303 9606.ENSP00000361405 0 0 0 0 0 0 0 0.563 0.563

BDNF HDAC1 9606.ENSP00000414303 9606.ENSP00000362649 0 0 0 0 0 0 0.9 0.48 0.945

BDNF TLR4 9606.ENSP00000414303 9606.ENSP00000363089 0 0 0 0 0 0.076 0 0.531 0.548

BDNF TH 9606.ENSP00000414303 9606.ENSP00000370571 0 0 0 0 0.062 0 0 0.773 0.778

BDNF INS 9606.ENSP00000414303 9606.ENSP00000380432 0 0 0 0 0 0 0 0.72 0.72

BDNF IL6 9606.ENSP00000414303 9606.ENSP00000385675 0 0 0 0 0 0 0 0.794 0.794

BDNF CXCR4 9606.ENSP00000414303 9606.ENSP00000386884 0 0 0 0 0 0 0 0.517 0.518

BDNF SCN10A 9606.ENSP00000414303 9606.ENSP00000390600 0 0 0 0 0 0 0 0.434 0.434

BDNF TNF 9606.ENSP00000414303 9606.ENSP00000398698 0 0 0 0 0 0 0 0.747 0.747

BDNF LTA 9606.ENSP00000414303 9606.ENSP00000403495 0 0 0 0 0 0 0 0.455 0.455

BDNF ESR1 9606.ENSP00000414303 9606.ENSP00000405330 0 0 0 0 0 0.213 0 0.608 0.678

BDNF FGFR2 9606.ENSP00000414303 9606.ENSP00000410294 0 0 0 0 0 0 0.6 0.239 0.682

BDNF IL10 9606.ENSP00000414303 9606.ENSP00000412237 0 0 0 0 0 0 0 0.675 0.675

BDNF CCL3 9606.ENSP00000414303 9606.ENSP00000477908 0 0 0 0 0 0 0 0.403 0.403

BDNF CCL5 9606.ENSP00000414303 9606.ENSP00000474412 0 0 0 0 0 0 0 0.452 0.452

BDNF HIF1A 9606.ENSP00000414303 9606.ENSP00000437955 0 0 0 0 0 0 0 0.465 0.465

BDNF FURIN 9606.ENSP00000414303 9606.ENSP00000483552 0 0 0 0 0 0.057 0 0.477 0.486

BDNF NOS1 9606.ENSP00000414303 9606.ENSP00000477999 0 0 0 0 0 0 0 0.527 0.527

BDNF HTR2A 9606.ENSP00000414303 9606.ENSP00000437737 0 0 0 0 0.062 0 0 0.736 0.742

BDNF VEGFA 9606.ENSP00000414303 9606.ENSP00000478570 0 0 0 0 0 0 0 0.798 0.799

BDNF GRIN2B 9606.ENSP00000414303 9606.ENSP00000477455 0 0 0 0 0.062 0 0.6 0.767 0.905

BGLAP MMP2 9606.ENSP00000357255 9606.ENSP00000219070 0 0 0 0 0 0 0 0.406 0.406

BGLAP TGFB1 9606.ENSP00000357255 9606.ENSP00000221930 0 0 0 0 0 0 0 0.56 0.56

BGLAP COL1A1 9606.ENSP00000357255 9606.ENSP00000225964 0 0 0 0 0 0 0 0.827 0.827

BGLAP FGF23 9606.ENSP00000357255 9606.ENSP00000237837 0 0 0 0 0 0 0 0.733 0.733

BGLAP CRP 9606.ENSP00000357255 9606.ENSP00000255030 0 0 0 0 0 0 0 0.561 0.561

BGLAP IL1B 9606.ENSP00000357255 9606.ENSP00000263341 0 0 0 0 0 0 0 0.557 0.557

BGLAP FGF2 9606.ENSP00000357255 9606.ENSP00000264498 0 0 0 0 0 0 0 0.644 0.644

BGLAP LEF1 9606.ENSP00000357255 9606.ENSP00000265165 0 0 0 0 0 0 0 0.405 0.405

BGLAP EGF 9606.ENSP00000357255 9606.ENSP00000265171 0 0 0 0 0 0 0 0.402 0.402

BGLAP BMP6 9606.ENSP00000357255 9606.ENSP00000283147 0 0 0 0 0 0 0 0.603 0.603

BGLAP PPARG 9606.ENSP00000357255 9606.ENSP00000287820 0 0 0 0 0 0 0 0.701 0.701

BGLAP VCAM1 9606.ENSP00000357255 9606.ENSP00000294728 0 0 0 0 0 0 0 0.404 0.404

BGLAP MMP3 9606.ENSP00000357255 9606.ENSP00000299855 0 0 0 0 0 0 0 0.459 0.459

BGLAP IGF1 9606.ENSP00000357255 9606.ENSP00000302665 0 0 0 0 0 0 0 0.759 0.759

BGLAP CXCL8 9606.ENSP00000357255 9606.ENSP00000306512 0 0 0 0 0 0 0 0.417 0.416

BGLAP F2 9606.ENSP00000357255 9606.ENSP00000308541 0 0 0 0 0 0 0 0.459 0.459

BGLAP LPL 9606.ENSP00000357255 9606.ENSP00000309757 0 0 0 0 0 0 0 0.594 0.594

BGLAP LEP 9606.ENSP00000357255 9606.ENSP00000312652 0 0 0 0 0 0 0 0.683 0.683

BGLAP CALCA 9606.ENSP00000357255 9606.ENSP00000331746 0 0 0 0 0 0 0 0.711 0.711

BGLAP HIF1A 9606.ENSP00000357255 9606.ENSP00000437955 0 0 0 0 0 0 0 0.404 0.404

BGLAP GDF5 9606.ENSP00000357255 9606.ENSP00000363492 0 0 0 0 0 0 0 0.46 0.459

BGLAP JUN 9606.ENSP00000357255 9606.ENSP00000360266 0 0 0 0 0 0 0 0.469 0.469

BGLAP MMP9 9606.ENSP00000357255 9606.ENSP00000361405 0 0 0 0 0.065 0 0 0.518 0.53

BGLAP ESR1 9606.ENSP00000357255 9606.ENSP00000405330 0 0 0 0 0 0 0 0.571 0.571

BGLAP TNF 9606.ENSP00000357255 9606.ENSP00000398698 0 0 0 0 0 0 0 0.612 0.612

BGLAP SHBG 9606.ENSP00000357255 9606.ENSP00000369816 0 0 0 0 0 0 0 0.659 0.659

BGLAP IL6 9606.ENSP00000357255 9606.ENSP00000385675 0 0 0 0 0 0 0 0.661 0.661

BGLAP VEGFA 9606.ENSP00000357255 9606.ENSP00000478570 0 0 0 0 0 0 0 0.666 0.666

BGLAP INS 9606.ENSP00000357255 9606.ENSP00000380432 0 0 0 0 0 0 0 0.707 0.707

BGLAP VDR 9606.ENSP00000357255 9606.ENSP00000447173 0 0 0 0 0 0 0 0.709 0.709

BGLAP FURIN 9606.ENSP00000357255 9606.ENSP00000483552 0 0 0 0 0 0 0.9 0.15 0.911

BGLAP SPP1 9606.ENSP00000357255 9606.ENSP00000378517 0 0 0 0 0.062 0 0 0.916 0.918

BMP6 COL1A1 9606.ENSP00000283147 9606.ENSP00000225964 0 0 0 0 0.062 0.115 0 0.43 0.486

BMP6 SMAD7 9606.ENSP00000283147 9606.ENSP00000262158 0 0 0 0 0 0.148 0 0.588 0.634

BMP6 FGF2 9606.ENSP00000283147 9606.ENSP00000264498 0 0 0 0 0 0 0 0.598 0.599

BMP6 EGF 9606.ENSP00000283147 9606.ENSP00000265171 0 0 0 0 0 0 0 0.407 0.407

BMP6 INS 9606.ENSP00000283147 9606.ENSP00000380432 0 0 0 0 0 0 0 0.415 0.415

BMP6 MMP9 9606.ENSP00000283147 9606.ENSP00000361405 0 0 0 0 0 0 0 0.464 0.464

BMP6 SPP1 9606.ENSP00000283147 9606.ENSP00000378517 0 0 0 0 0 0 0 0.473 0.473

BMP6 IL6 9606.ENSP00000283147 9606.ENSP00000385675 0 0 0 0 0.063 0 0 0.468 0.48

BMP6 VEGFA 9606.ENSP00000283147 9606.ENSP00000478570 0 0 0 0 0.062 0 0 0.52 0.53

BMP6 IGF1 9606.ENSP00000283147 9606.ENSP00000302665 0 0 0 0 0.05 0 0 0.589 0.593

BRCA1 CFTR 9606.ENSP00000418960 9606.ENSP00000003084 0 0 0 0 0 0 0 0.42 0.42

BRCA1 HMOX1 9606.ENSP00000418960 9606.ENSP00000216117 0 0 0 0 0 0 0 0.45 0.45

BRCA1 NR3C1 9606.ENSP00000418960 9606.ENSP00000231509 0 0 0 0 0 0.058 0 0.66 0.666

BRCA1 MDM2 9606.ENSP00000418960 9606.ENSP00000258149 0 0 0 0 0 0 0 0.675 0.675

BRCA1 EGF 9606.ENSP00000418960 9606.ENSP00000265171 0 0 0 0 0 0 0 0.522 0.522

BRCA1 ERBB3 9606.ENSP00000418960 9606.ENSP00000267101 0 0 0 0 0 0 0 0.466 0.465

BRCA1 TP53 9606.ENSP00000418960 9606.ENSP00000269305 0 0 0 0 0.076 0.9 0.9 0.989 0.999

BRCA1 ERBB2 9606.ENSP00000418960 9606.ENSP00000269571 0 0 0 0 0 0 0 0.856 0.856

BRCA1 EGFR 9606.ENSP00000418960 9606.ENSP00000275493 0 0 0 0 0 0 0 0.837 0.837

BRCA1 PPARG 9606.ENSP00000418960 9606.ENSP00000287820 0 0 0 0 0 0.058 0 0.512 0.52

BRCA1 KIT 9606.ENSP00000418960 9606.ENSP00000288135 0 0 0 0 0 0 0 0.503 0.503

BRCA1 VCAM1 9606.ENSP00000418960 9606.ENSP00000294728 0 0 0 0 0 0.459 0 0.134 0.511

BRCA1 IGF1 9606.ENSP00000418960 9606.ENSP00000302665 0 0 0 0 0 0 0 0.456 0.456

BRCA1 ITPR1 9606.ENSP00000418960 9606.ENSP00000306253 0 0 0 0 0.064 0.312 0 0.472 0.63

BRCA1 CASP3 9606.ENSP00000418960 9606.ENSP00000311032 0 0 0 0 0.062 0.213 0 0.594 0.674

BRCA1 CASP9 9606.ENSP00000418960 9606.ENSP00000330237 0 0 0 0 0 0.27 0 0.353 0.507

BRCA1 STAT5A 9606.ENSP00000418960 9606.ENSP00000341208 0 0 0 0 0 0.486 0 0.915 0.954

BRCA1 USP7 9606.ENSP00000418960 9606.ENSP00000343535 0 0 0 0 0.062 0.128 0 0.408 0.473

BRCA1 E2F1 9606.ENSP00000418960 9606.ENSP00000345571 0 0 0 0 0.13 0.617 0 0.483 0.813

BRCA1 RET 9606.ENSP00000418960 9606.ENSP00000347942 0 0 0 0 0 0 0 0.47 0.47

BRCA1 CASP8 9606.ENSP00000418960 9606.ENSP00000351273 0 0 0 0 0.062 0 0 0.46 0.471

BRCA1 VCP 9606.ENSP00000418960 9606.ENSP00000351777 0 0 0 0 0.062 0.486 0 0.955 0.976

BRCA1 DNMT1 9606.ENSP00000418960 9606.ENSP00000352516 0 0 0 0 0.163 0.128 0 0.656 0.727

BRCA1 STAT1 9606.ENSP00000418960 9606.ENSP00000354394 0 0 0 0 0 0.27 0 0.876 0.906

BRCA1 TOP1 9606.ENSP00000418960 9606.ENSP00000354522 0 0 0 0 0.062 0.429 0 0.609 0.772

BRCA1 MTOR 9606.ENSP00000418960 9606.ENSP00000354558 0 0 0 0 0.051 0.076 0 0.469 0.494

BRCA1 PARP1 9606.ENSP00000418960 9606.ENSP00000355759 0 0 0 0 0.098 0.479 0 0.886 0.942

BRCA1 JUN 9606.ENSP00000418960 9606.ENSP00000360266 0 0 0 0 0 0.64 0 0.712 0.892

BRCA1 PTEN 9606.ENSP00000418960 9606.ENSP00000361021 0 0 0 0 0.064 0 0 0.807 0.812

BRCA1 HDAC1 9606.ENSP00000418960 9606.ENSP00000362649 0 0 0 0 0.062 0.27 0 0.725 0.795

BRCA1 PCNA 9606.ENSP00000418960 9606.ENSP00000368458 0 0 0 0 0.279 0.128 0.9 0.172 0.941

BRCA1 HELLS 9606.ENSP00000418960 9606.ENSP00000377601 0 0 0 0 0.367 0.056 0 0.148 0.447

BRCA1 CYP19A1 9606.ENSP00000418960 9606.ENSP00000379683 0 0 0 0 0 0 0 0.605 0.605

BRCA1 INS 9606.ENSP00000418960 9606.ENSP00000380432 0 0 0 0 0 0 0 0.501 0.501

BRCA1 CDKN1A 9606.ENSP00000418960 9606.ENSP00000384849 0 0 0 0 0 0.27 0 0.617 0.709

BRCA1 ESR1 9606.ENSP00000418960 9606.ENSP00000405330 0 0 0 0 0 0.9 0.9 0.988 0.999

BRCA1 POLD1 9606.ENSP00000418960 9606.ENSP00000406046 0 0 0 0 0.198 0.391 0.9 0.613 0.978

BRCA1 FGFR2 9606.ENSP00000418960 9606.ENSP00000410294 0 0 0 0 0 0 0 0.557 0.557

BRCA1 CYP1B1 9606.ENSP00000418960 9606.ENSP00000478561 0 0 0 0 0 0 0 0.5 0.5

BRCA1 VEGFA 9606.ENSP00000418960 9606.ENSP00000478570 0 0 0 0 0 0.128 0 0.518 0.561

BRCA1 VDR 9606.ENSP00000418960 9606.ENSP00000447173 0 0 0 0 0 0.282 0 0.458 0.594

BRCA1 HIF1A 9606.ENSP00000418960 9606.ENSP00000437955 0 0 0 0 0.062 0.27 0 0.634 0.728

C3 CTSG 9606.ENSP00000245907 9606.ENSP00000216336 0 0 0 0 0 0.514 0 0.358 0.674

C3 MPO 9606.ENSP00000245907 9606.ENSP00000225275 0 0 0 0 0 0.297 0 0.527 0.653

C3 CCL2 9606.ENSP00000245907 9606.ENSP00000225831 0 0 0 0 0.088 0 0 0.505 0.529

C3 IL2 9606.ENSP00000245907 9606.ENSP00000226730 0 0 0 0 0 0 0 0.465 0.465

C3 IFNG 9606.ENSP00000245907 9606.ENSP00000229135 0 0 0 0 0 0 0 0.412 0.412

C3 IL4 9606.ENSP00000245907 9606.ENSP00000231449 0 0 0 0 0 0 0 0.459 0.459

C3 LTF 9606.ENSP00000245907 9606.ENSP00000231751 0 0 0 0 0.109 0 0 0.392 0.436

C3 CAT 9606.ENSP00000245907 9606.ENSP00000241052 0.078 0 0 0 0.062 0 0 0.402 0.437

C3 CXCL10 9606.ENSP00000245907 9606.ENSP00000305651 0 0 0 0 0.088 0 0 0.37 0.401

C3 CCL5 9606.ENSP00000245907 9606.ENSP00000474412 0 0 0 0 0.063 0 0 0.391 0.404

C3 CD40 9606.ENSP00000245907 9606.ENSP00000361359 0 0 0 0 0.062 0 0 0.391 0.404

C3 HBA2 9606.ENSP00000245907 9606.ENSP00000251595 0 0 0 0 0.062 0 0 0.391 0.404

C3 CSF2 9606.ENSP00000245907 9606.ENSP00000296871 0 0 0 0 0 0 0 0.404 0.404

C3 MYD88 9606.ENSP00000245907 9606.ENSP00000401399 0 0 0 0 0.062 0 0 0.392 0.405

C3 IL1A 9606.ENSP00000245907 9606.ENSP00000263339 0 0 0 0 0.064 0 0 0.391 0.405

C3 MMP9 9606.ENSP00000245907 9606.ENSP00000361405 0 0 0 0 0.062 0 0 0.394 0.407

C3 LCN2 9606.ENSP00000245907 9606.ENSP00000362108 0 0 0 0 0.09 0 0 0.38 0.411

C3 TLR3 9606.ENSP00000245907 9606.ENSP00000296795 0 0 0 0 0.062 0.087 0 0.369 0.412

C3 GPT 9606.ENSP00000245907 9606.ENSP00000378408 0 0 0 0 0.063 0 0 0.399 0.413

C3 VCAM1 9606.ENSP00000245907 9606.ENSP00000294728 0 0 0 0 0.088 0 0 0.391 0.42

C3 LYZ 9606.ENSP00000245907 9606.ENSP00000261267 0 0 0 0 0.097 0 0 0.385 0.421

C3 INS 9606.ENSP00000245907 9606.ENSP00000380432 0 0 0 0 0 0 0 0.422 0.422

C3 VCP 9606.ENSP00000245907 9606.ENSP00000351777 0 0 0 0 0 0 0 0.428 0.428

C3 MTOR 9606.ENSP00000245907 9606.ENSP00000354558 0 0 0 0 0 0 0 0.454 0.454

C3 IL17A 9606.ENSP00000245907 9606.ENSP00000344192 0 0 0 0 0 0 0 0.457 0.457

C3 VEGFA 9606.ENSP00000245907 9606.ENSP00000478570 0 0 0 0 0.073 0 0 0.459 0.477

C3 ICAM1 9606.ENSP00000245907 9606.ENSP00000264832 0 0 0 0 0.098 0 0 0.468 0.499

C3 CD40LG 9606.ENSP00000245907 9606.ENSP00000359663 0 0 0 0 0 0.312 0 0.325 0.515

C3 ELANE 9606.ENSP00000245907 9606.ENSP00000466090 0 0 0 0 0.062 0.134 0 0.46 0.523

C3 ORM1 9606.ENSP00000245907 9606.ENSP00000259396 0 0 0 0 0.112 0 0 0.505 0.542

C3 IL10 9606.ENSP00000245907 9606.ENSP00000412237 0 0 0 0 0 0 0 0.565 0.565

C3 CA2 9606.ENSP00000245907 9606.ENSP00000285379 0 0 0 0 0 0 0 0.576 0.576

C3 F2 9606.ENSP00000245907 9606.ENSP00000308541 0 0 0 0 0.109 0.134 0 0.508 0.588

C3 CXCL8 9606.ENSP00000245907 9606.ENSP00000306512 0 0 0 0 0.07 0 0 0.577 0.59

C3 TLR4 9606.ENSP00000245907 9606.ENSP00000363089 0 0 0 0 0.062 0.087 0 0.583 0.611

C3 IL1B 9606.ENSP00000245907 9606.ENSP00000263341 0 0 0 0 0.064 0 0 0.606 0.615

C3 TNF 9606.ENSP00000245907 9606.ENSP00000398698 0 0 0 0 0.062 0 0 0.611 0.62

C3 IL6 9606.ENSP00000245907 9606.ENSP00000385675 0 0 0 0 0.071 0 0 0.616 0.628

C3 THBD 9606.ENSP00000245907 9606.ENSP00000366307 0 0 0 0 0.073 0 0 0.784 0.791

C3 CRP 9606.ENSP00000245907 9606.ENSP00000255030 0 0 0 0 0.08 0.486 0 0.897 0.947

C3 ITGB2 9606.ENSP00000245907 9606.ENSP00000380948 0 0 0 0 0.064 0.27 0.9 0.587 0.968

C3 SELP 9606.ENSP00000245907 9606.ENSP00000263686 0 0 0 0 0.062 0 0 0.988 0.989

CA2 CFTR 9606.ENSP00000285379 9606.ENSP00000003084 0 0 0 0 0 0 0 0.581 0.581

CA2 HBA2 9606.ENSP00000285379 9606.ENSP00000251595 0 0 0 0 0.09 0 0 0.387 0.419

CA2 HBA1 9606.ENSP00000285379 9606.ENSP00000322421 0 0 0 0 0.083 0 0 0.404 0.43

CA2 JUN 9606.ENSP00000285379 9606.ENSP00000360266 0 0 0 0 0 0.129 0 0.413 0.467

CA2 MMP9 9606.ENSP00000285379 9606.ENSP00000361405 0 0 0 0 0 0 0 0.469 0.469

CACNA1A POLG 9606.ENSP00000353362 9606.ENSP00000268124 0 0 0 0 0 0 0 0.459 0.459

CACNA1A SCN1A 9606.ENSP00000353362 9606.ENSP00000303540 0 0 0 0.612 0.215 0.4 0 0.769 0.653

CACNA1A PRKCB 9606.ENSP00000353362 9606.ENSP00000305355 0 0 0 0 0.09 0 0.8 0.075 0.817

CACNA1A ITPR1 9606.ENSP00000353362 9606.ENSP00000306253 0 0 0 0 0.064 0.102 0 0.627 0.659

CACNA1A SCN11A 9606.ENSP00000353362 9606.ENSP00000307599 0 0 0 0.632 0 0.398 0 0.427 0.487

CACNA1A CACNB2 9606.ENSP00000353362 9606.ENSP00000320025 0 0 0 0 0.109 0.548 0.6 0.518 0.912

CACNA1A KCND2 9606.ENSP00000353362 9606.ENSP00000333496 0 0 0 0 0.17 0.074 0 0.407 0.504

CACNA1A PLCB1 9606.ENSP00000353362 9606.ENSP00000338185 0 0 0 0 0.089 0.082 0 0.369 0.426

CACNA1A CAV3 9606.ENSP00000353362 9606.ENSP00000341940 0 0 0 0 0.062 0.163 0 0.689 0.734

CACNA1A RYR1 9606.ENSP00000353362 9606.ENSP00000352608 0 0 0 0 0.102 0.166 0.8 0.425 0.902

CACNA1A ITPR3 9606.ENSP00000353362 9606.ENSP00000363435 0 0 0 0 0.064 0.102 0 0.349 0.405

CACNA1A SYT2 9606.ENSP00000353362 9606.ENSP00000356236 0 0 0 0 0.173 0.07 0 0.37 0.473

CACNA1A SCN10A 9606.ENSP00000353362 9606.ENSP00000390600 0 0 0 0.621 0 0.398 0 0.404 0.484

CACNA1A GRIN2B 9606.ENSP00000353362 9606.ENSP00000477455 0 0 0 0 0.231 0.057 0 0.381 0.512

CACNA1A SCN9A 9606.ENSP00000353362 9606.ENSP00000386306 0 0 0 0.621 0.065 0.397 0 0.531 0.525

CACNA1A KCNC3 9606.ENSP00000353362 9606.ENSP00000434241 0 0 0 0 0.066 0.074 0 0.7 0.718

CACNA1A INS 9606.ENSP00000353362 9606.ENSP00000380432 0 0 0 0 0 0 0.8 0.26 0.845

CACNB2 SCN1A 9606.ENSP00000320025 9606.ENSP00000303540 0 0 0 0 0.108 0.346 0 0.442 0.646

CACNB2 KCND2 9606.ENSP00000320025 9606.ENSP00000333496 0 0 0 0 0.086 0 0 0.397 0.425

CACNB2 CAV3 9606.ENSP00000320025 9606.ENSP00000341940 0 0 0 0 0 0 0 0.452 0.452

CACNB2 SCN9A 9606.ENSP00000320025 9606.ENSP00000386306 0 0 0 0 0.062 0.346 0 0.286 0.523

CACNB2 SCN10A 9606.ENSP00000320025 9606.ENSP00000390600 0 0 0 0 0.062 0.278 0 0.475 0.613

CACNB2 RYR1 9606.ENSP00000320025 9606.ENSP00000352608 0 0 0 0 0.062 0 0.72 0.127 0.75

CACNB2 INS 9606.ENSP00000320025 9606.ENSP00000380432 0 0 0 0 0 0 0.9 0.094 0.905

CALCA CCL2 9606.ENSP00000331746 9606.ENSP00000225831 0 0 0 0 0 0 0 0.4 0.4

CALCA FGF23 9606.ENSP00000331746 9606.ENSP00000237837 0 0 0 0 0 0 0 0.519 0.519

CALCA CRP 9606.ENSP00000331746 9606.ENSP00000255030 0 0 0 0 0 0 0 0.611 0.611

CALCA LYZ 9606.ENSP00000331746 9606.ENSP00000261267 0 0 0 0 0 0 0.5 0.071 0.516

CALCA IL1B 9606.ENSP00000331746 9606.ENSP00000263341 0 0 0 0 0 0 0 0.566 0.566

CALCA REN 9606.ENSP00000331746 9606.ENSP00000272190 0 0 0 0 0.054 0 0 0.52 0.526

CALCA CRH 9606.ENSP00000331746 9606.ENSP00000276571 0 0 0 0 0.09 0 0.3 0.683 0.78

CALCA SST 9606.ENSP00000331746 9606.ENSP00000287641 0 0 0 0 0.066 0 0 0.866 0.87

CALCA F2RL1 9606.ENSP00000331746 9606.ENSP00000296677 0 0 0 0 0 0 0 0.464 0.464

CALCA NOS3 9606.ENSP00000331746 9606.ENSP00000297494 0 0 0 0 0.062 0 0 0.46 0.472

CALCA IGF1 9606.ENSP00000331746 9606.ENSP00000302665 0 0 0 0 0.064 0 0 0.518 0.529

CALCA CXCL8 9606.ENSP00000331746 9606.ENSP00000306512 0 0 0 0 0.055 0 0 0.459 0.466

CALCA SCN11A 9606.ENSP00000331746 9606.ENSP00000307599 0 0 0 0 0.06 0 0 0.424 0.435

CALCA LEP 9606.ENSP00000331746 9606.ENSP00000312652 0 0 0 0 0 0 0 0.471 0.471

CALCA IL10 9606.ENSP00000331746 9606.ENSP00000412237 0 0 0 0 0 0 0 0.46 0.46

CALCA VEGFA 9606.ENSP00000331746 9606.ENSP00000478570 0 0 0 0 0 0 0 0.467 0.467

CALCA NOS1 9606.ENSP00000331746 9606.ENSP00000477999 0 0 0 0 0.062 0 0 0.475 0.486

CALCA SNCA 9606.ENSP00000331746 9606.ENSP00000338345 0 0 0 0 0.062 0 0.3 0.287 0.49

CALCA SCN9A 9606.ENSP00000331746 9606.ENSP00000386306 0 0 0 0 0.062 0 0 0.484 0.495

CALCA SCN10A 9606.ENSP00000331746 9606.ENSP00000390600 0 0 0 0 0 0 0 0.553 0.553

CALCA NKX2-1 9606.ENSP00000331746 9606.ENSP00000346879 0 0 0 0 0 0 0 0.595 0.595

CALCA TNF 9606.ENSP00000331746 9606.ENSP00000398698 0 0 0 0 0 0 0 0.609 0.609

CALCA EDN1 9606.ENSP00000331746 9606.ENSP00000368683 0 0 0 0 0 0 0 0.609 0.609

CALCA ESR1 9606.ENSP00000331746 9606.ENSP00000405330 0 0 0 0 0 0 0 0.612 0.612

CALCA IL6 9606.ENSP00000331746 9606.ENSP00000385675 0 0 0 0 0 0 0 0.682 0.682

CALCA TH 9606.ENSP00000331746 9606.ENSP00000370571 0 0 0 0 0.066 0 0 0.753 0.76

CALCA RET 9606.ENSP00000331746 9606.ENSP00000347942 0 0 0 0 0.062 0 0 0.76 0.765

CALCA NGF 9606.ENSP00000331746 9606.ENSP00000358525 0 0 0 0 0.062 0 0 0.784 0.789

CALCA INS 9606.ENSP00000331746 9606.ENSP00000380432 0 0 0 0 0 0 0.5 0.691 0.839

CASP3 TNFRSF1A 9606.ENSP00000311032 9606.ENSP00000162749 0 0 0 0 0 0 0 0.705 0.705

CASP3 HMOX1 9606.ENSP00000311032 9606.ENSP00000216117 0 0 0 0 0 0 0 0.762 0.762

CASP3 NFKBIA 9606.ENSP00000311032 9606.ENSP00000216797 0 0 0 0 0 0.09 0 0.734 0.748

CASP3 MMP2 9606.ENSP00000311032 9606.ENSP00000219070 0 0 0 0 0 0 0 0.681 0.681

CASP3 TGFB1 9606.ENSP00000311032 9606.ENSP00000221930 0 0 0 0 0 0 0 0.578 0.578

CASP3 MPO 9606.ENSP00000311032 9606.ENSP00000225275 0 0 0 0 0 0 0 0.66 0.66

CASP3 CCL2 9606.ENSP00000311032 9606.ENSP00000225831 0 0 0 0 0.049 0 0 0.636 0.639

CASP3 COL1A1 9606.ENSP00000311032 9606.ENSP00000225964 0 0 0 0 0 0 0 0.404 0.404

CASP3 NFKB1 9606.ENSP00000311032 9606.ENSP00000226574 0 0 0 0 0 0.09 0 0.461 0.489

CASP3 IL2 9606.ENSP00000311032 9606.ENSP00000226730 0 0 0 0 0 0 0 0.736 0.736

CASP3 IFNG 9606.ENSP00000311032 9606.ENSP00000229135 0 0 0 0 0 0 0 0.608 0.608

CASP3 IL4 9606.ENSP00000311032 9606.ENSP00000231449 0 0 0 0 0 0 0 0.566 0.566

CASP3 NR3C1 9606.ENSP00000311032 9606.ENSP00000231509 0 0 0 0 0 0.06 0 0.391 0.403

CASP3 CTSD 9606.ENSP00000311032 9606.ENSP00000236671 0 0 0 0 0 0.058 0 0.567 0.575

CASP3 CAT 9606.ENSP00000311032 9606.ENSP00000241052 0 0 0 0 0 0 0 0.779 0.779

CASP3 HSPB1 9606.ENSP00000311032 9606.ENSP00000248553 0 0 0 0 0 0.303 0 0.699 0.782

CASP3 CRP 9606.ENSP00000311032 9606.ENSP00000255030 0 0 0 0 0 0 0 0.494 0.494

CASP3 MDM2 9606.ENSP00000311032 9606.ENSP00000258149 0 0 0 0 0.063 0.875 0 0.73 0.965

CASP3 IL1A 9606.ENSP00000311032 9606.ENSP00000263339 0 0 0 0 0 0 0 0.528 0.528

CASP3 IL1B 9606.ENSP00000311032 9606.ENSP00000263341 0 0 0 0 0 0.056 0 0.825 0.827

CASP3 SELP 9606.ENSP00000311032 9606.ENSP00000263686 0 0 0 0 0 0 0 0.408 0.408

CASP3 FGF2 9606.ENSP00000311032 9606.ENSP00000264498 0 0 0 0 0 0 0 0.689 0.689

CASP3 ICAM1 9606.ENSP00000311032 9606.ENSP00000264832 0 0 0 0 0 0 0 0.643 0.643

CASP3 EGF 9606.ENSP00000311032 9606.ENSP00000265171 0 0 0 0 0 0 0 0.71 0.71

CASP3 ERBB3 9606.ENSP00000311032 9606.ENSP00000267101 0 0 0 0 0 0.06 0 0.466 0.477

CASP3 TP53 9606.ENSP00000311032 9606.ENSP00000269305 0 0 0 0 0.062 0.27 0 0.9 0.925

CASP3 ERBB2 9606.ENSP00000311032 9606.ENSP00000269571 0 0 0 0 0 0.06 0 0.69 0.696

CASP3 SOD1 9606.ENSP00000311032 9606.ENSP00000270142 0 0 0 0 0 0.102 0 0.604 0.629

CASP3 REN 9606.ENSP00000311032 9606.ENSP00000272190 0 0 0 0 0 0.058 0 0.486 0.495

CASP3 EGFR 9606.ENSP00000311032 9606.ENSP00000275493 0 0 0 0 0 0.06 0 0.754 0.759

CASP3 PPARG 9606.ENSP00000311032 9606.ENSP00000287820 0 0 0 0 0 0.06 0 0.637 0.644

CASP3 KIT 9606.ENSP00000311032 9606.ENSP00000288135 0 0 0 0 0 0 0 0.582 0.582

CASP3 NCF1 9606.ENSP00000311032 9606.ENSP00000289473 0 0 0 0 0.052 0 0 0.471 0.477

CASP3 VCAM1 9606.ENSP00000311032 9606.ENSP00000294728 0 0 0 0 0.053 0 0 0.549 0.554

CASP3 TLR3 9606.ENSP00000311032 9606.ENSP00000296795 0 0 0 0 0 0.07 0 0.459 0.475

CASP3 CSF2 9606.ENSP00000311032 9606.ENSP00000296871 0 0 0 0 0 0 0 0.51 0.51

CASP3 NOS3 9606.ENSP00000311032 9606.ENSP00000297494 0 0 0 0 0 0 0 0.682 0.682

CASP3 HPRT1 9606.ENSP00000311032 9606.ENSP00000298556 0 0 0 0 0 0 0 0.456 0.456

CASP3 MMP3 9606.ENSP00000311032 9606.ENSP00000299855 0 0 0 0 0 0 0 0.633 0.633

CASP3 FADD 9606.ENSP00000311032 9606.ENSP00000301838 0 0 0 0 0 0.123 0.9 0.858 0.986

CASP3 IGF1 9606.ENSP00000311032 9606.ENSP00000302665 0 0 0 0 0 0 0 0.702 0.702

CASP3 IL13 9606.ENSP00000311032 9606.ENSP00000304915 0 0 0 0 0 0 0 0.424 0.424

CASP3 PRKCB 9606.ENSP00000311032 9606.ENSP00000305355 0 0 0 0 0 0 0.8 0.307 0.855

CASP3 CXCL10 9606.ENSP00000311032 9606.ENSP00000305651 0 0 0 0 0 0 0 0.469 0.469

CASP3 ITPR1 9606.ENSP00000311032 9606.ENSP00000306253 0 0 0 0 0 0 0 0.563 0.563

CASP3 CXCL8 9606.ENSP00000311032 9606.ENSP00000306512 0 0 0 0 0 0 0 0.668 0.668

CASP3 CYCS 9606.ENSP00000311032 9606.ENSP00000307786 0 0 0 0 0.063 0 0.9 0.952 0.995

CASP3 CD40LG 9606.ENSP00000311032 9606.ENSP00000359663 0 0 0 0 0 0.056 0 0.394 0.403

CASP3 TNFRSF1B 9606.ENSP00000311032 9606.ENSP00000365435 0 0 0 0 0 0 0 0.404 0.404

CASP3 E2F1 9606.ENSP00000311032 9606.ENSP00000345571 0 0 0 0 0 0 0 0.414 0.414

CASP3 CCL3 9606.ENSP00000311032 9606.ENSP00000477908 0 0 0 0 0 0 0 0.416 0.416

CASP3 GRIN2B 9606.ENSP00000311032 9606.ENSP00000477455 0 0 0 0 0 0 0 0.42 0.42

CASP3 SELE 9606.ENSP00000311032 9606.ENSP00000331736 0 0 0 0 0 0 0 0.422 0.422

CASP3 CD28 9606.ENSP00000311032 9606.ENSP00000324890 0 0 0 0 0 0 0 0.424 0.424

CASP3 CYP19A1 9606.ENSP00000311032 9606.ENSP00000379683 0 0 0 0 0 0 0 0.426 0.426

CASP3 LTA 9606.ENSP00000311032 9606.ENSP00000403495 0 0 0 0 0 0.078 0 0.418 0.44

CASP3 ELANE 9606.ENSP00000311032 9606.ENSP00000466090 0 0 0 0 0 0 0 0.452 0.452

CASP3 ITPR3 9606.ENSP00000311032 9606.ENSP00000363435 0 0 0 0 0 0 0 0.452 0.452

CASP3 CYP1B1 9606.ENSP00000311032 9606.ENSP00000478561 0 0 0 0 0 0 0 0.464 0.464

CASP3 CD40 9606.ENSP00000311032 9606.ENSP00000361359 0 0 0 0 0.048 0 0 0.467 0.47

CASP3 MMP1 9606.ENSP00000311032 9606.ENSP00000322788 0 0 0 0 0 0 0 0.472 0.472

CASP3 CCL5 9606.ENSP00000311032 9606.ENSP00000474412 0 0 0 0 0 0 0 0.476 0.476

CASP3 DNMT1 9606.ENSP00000311032 9606.ENSP00000352516 0 0 0 0 0.062 0 0 0.48 0.492

CASP3 LCN2 9606.ENSP00000311032 9606.ENSP00000362108 0 0 0 0 0 0 0 0.494 0.494

CASP3 LEP 9606.ENSP00000311032 9606.ENSP00000312652 0 0 0 0 0 0 0 0.496 0.496

CASP3 FOXP3 9606.ENSP00000311032 9606.ENSP00000365380 0 0 0 0 0 0 0 0.504 0.504

CASP3 IL17A 9606.ENSP00000311032 9606.ENSP00000344192 0 0 0 0 0 0 0 0.504 0.504

CASP3 NOS1 9606.ENSP00000311032 9606.ENSP00000477999 0 0 0 0 0 0 0 0.523 0.523

CASP3 P4HB 9606.ENSP00000311032 9606.ENSP00000327801 0 0 0 0 0.062 0.149 0 0.455 0.527

CASP3 NOS2 9606.ENSP00000311032 9606.ENSP00000327251 0 0 0 0 0 0 0 0.529 0.529

CASP3 EDN1 9606.ENSP00000311032 9606.ENSP00000368683 0 0 0 0 0 0 0 0.54 0.54

CASP3 CXCR4 9606.ENSP00000311032 9606.ENSP00000386884 0 0 0 0 0 0.056 0 0.55 0.557

CASP3 HDAC1 9606.ENSP00000311032 9606.ENSP00000362649 0 0 0 0 0.062 0 0 0.549 0.558

CASP3 MYD88 9606.ENSP00000311032 9606.ENSP00000401399 0 0 0 0 0 0.115 0 0.535 0.571

CASP3 STAT5A 9606.ENSP00000311032 9606.ENSP00000341208 0 0 0 0 0 0 0 0.574 0.574

CASP3 SNCA 9606.ENSP00000311032 9606.ENSP00000338345 0 0 0 0 0 0 0 0.575 0.575

CASP3 TH 9606.ENSP00000311032 9606.ENSP00000370571 0 0 0 0 0 0 0 0.576 0.576

CASP3 SPP1 9606.ENSP00000311032 9606.ENSP00000378517 0 0 0 0 0 0.27 0 0.459 0.588

CASP3 CHUK 9606.ENSP00000311032 9606.ENSP00000359424 0 0 0 0 0.077 0.103 0 0.572 0.615

CASP3 RET 9606.ENSP00000311032 9606.ENSP00000347942 0 0 0 0 0 0.27 0 0.499 0.619

CASP3 ESR1 9606.ENSP00000311032 9606.ENSP00000405330 0 0 0 0 0 0.06 0 0.674 0.68

CASP3 GPT 9606.ENSP00000311032 9606.ENSP00000378408 0 0 0 0 0 0 0 0.681 0.681

CASP3 TLR4 9606.ENSP00000311032 9606.ENSP00000363089 0 0 0 0 0 0.07 0 0.681 0.69

CASP3 IL10 9606.ENSP00000311032 9606.ENSP00000412237 0 0 0 0 0 0 0 0.7 0.7

CASP3 STAT1 9606.ENSP00000311032 9606.ENSP00000354394 0 0 0 0 0.063 0.271 0 0.598 0.701

CASP3 MTOR 9606.ENSP00000311032 9606.ENSP00000354558 0 0 0 0 0 0.057 0 0.71 0.714

CASP3 HIF1A 9606.ENSP00000311032 9606.ENSP00000437955 0 0 0 0 0.049 0 0 0.718 0.72

CASP3 VEGFA 9606.ENSP00000311032 9606.ENSP00000478570 0 0 0 0 0 0 0 0.76 0.76

CASP3 PTEN 9606.ENSP00000311032 9606.ENSP00000361021 0 0 0 0 0.062 0 0 0.762 0.767

CASP3 MMP9 9606.ENSP00000311032 9606.ENSP00000361405 0 0 0 0 0.063 0 0 0.766 0.772

CASP3 INS 9606.ENSP00000311032 9606.ENSP00000380432 0 0 0 0 0 0 0 0.792 0.792

CASP3 FASLG 9606.ENSP00000311032 9606.ENSP00000356694 0 0 0 0 0 0 0 0.803 0.803

CASP3 HSPA5 9606.ENSP00000311032 9606.ENSP00000324173 0 0 0 0 0 0.153 0 0.8 0.824

CASP3 IL6 9606.ENSP00000311032 9606.ENSP00000385675 0 0 0 0 0 0 0 0.836 0.836

CASP3 JUN 9606.ENSP00000311032 9606.ENSP00000360266 0 0 0 0 0 0.057 0 0.834 0.837

CASP3 FAS 9606.ENSP00000311032 9606.ENSP00000347979 0 0 0 0 0 0.059 0.36 0.764 0.845

CASP3 TNF 9606.ENSP00000311032 9606.ENSP00000398698 0 0 0 0 0 0.102 0 0.889 0.896

CASP3 SREBF1 9606.ENSP00000311032 9606.ENSP00000348069 0 0 0 0 0 0 0.9 0.422 0.939

CASP3 OCLN 9606.ENSP00000311032 9606.ENSP00000347379 0 0 0 0 0 0 0.9 0.533 0.951

CASP3 TOP1 9606.ENSP00000311032 9606.ENSP00000354522 0 0 0 0 0 0.263 0.9 0.559 0.964

CASP3 NGF 9606.ENSP00000311032 9606.ENSP00000358525 0 0 0 0 0 0 0.9 0.676 0.966

CASP3 CASP8 9606.ENSP00000311032 9606.ENSP00000351273 0 0 0 0.835 0.062 0.768 0.9 0.96 0.98

CASP3 PRKCD 9606.ENSP00000311032 9606.ENSP00000378217 0 0 0 0 0 0 0.9 0.854 0.984

CASP3 CASP9 9606.ENSP00000311032 9606.ENSP00000330237 0 0 0 0.833 0 0.87 0.9 0.968 0.988

CASP3 CDKN1A 9606.ENSP00000311032 9606.ENSP00000384849 0 0 0 0 0 0.77 0.8 0.883 0.994

CASP3 PARP1 9606.ENSP00000311032 9606.ENSP00000355759 0 0 0 0 0.062 0.88 0.9 0.851 0.998

CASP8 TNFRSF1A 9606.ENSP00000351273 9606.ENSP00000162749 0 0 0 0 0.063 0.476 0.9 0.99 0.999

CASP8 HMOX1 9606.ENSP00000351273 9606.ENSP00000216117 0 0 0 0 0 0 0 0.459 0.459

CASP8 NFKBIA 9606.ENSP00000351273 9606.ENSP00000216797 0 0 0 0 0.062 0.09 0 0.737 0.756

CASP8 MMP2 9606.ENSP00000351273 9606.ENSP00000219070 0 0 0 0 0.062 0 0 0.463 0.474

CASP8 CCL2 9606.ENSP00000351273 9606.ENSP00000225831 0 0 0 0 0.062 0 0 0.459 0.47

CASP8 COL1A1 9606.ENSP00000351273 9606.ENSP00000225964 0 0 0 0 0 0 0.9 0.18 0.914

CASP8 NFKB1 9606.ENSP00000351273 9606.ENSP00000226574 0 0 0 0 0.062 0.09 0.9 0.443 0.946

CASP8 IL2 9606.ENSP00000351273 9606.ENSP00000226730 0 0 0 0 0 0 0 0.515 0.515

CASP8 IFNG 9606.ENSP00000351273 9606.ENSP00000229135 0 0 0 0 0.062 0 0 0.508 0.519

CASP8 IL4 9606.ENSP00000351273 9606.ENSP00000231449 0 0 0 0 0 0 0 0.404 0.404

CASP8 CTSD 9606.ENSP00000351273 9606.ENSP00000236671 0 0 0 0 0.052 0.058 0 0.415 0.432

CASP8 CAT 9606.ENSP00000351273 9606.ENSP00000241052 0 0 0 0 0.062 0 0 0.575 0.584

CASP8 IRF1 9606.ENSP00000351273 9606.ENSP00000245414 0 0 0 0 0.13 0 0 0.394 0.451

CASP8 HSPB1 9606.ENSP00000351273 9606.ENSP00000248553 0 0 0 0 0 0.058 0 0.467 0.477

CASP8 MDM2 9606.ENSP00000351273 9606.ENSP00000258149 0 0 0 0 0.466 0 0 0.588 0.77

CASP8 IL1RN 9606.ENSP00000351273 9606.ENSP00000259206 0 0 0 0 0.085 0.056 0 0.461 0.494

CASP8 IL1A 9606.ENSP00000351273 9606.ENSP00000263339 0 0 0 0 0.062 0 0 0.461 0.472

CASP8 IL1B 9606.ENSP00000351273 9606.ENSP00000263341 0 0 0 0 0.063 0.182 0.9 0.715 0.975

CASP8 ICAM1 9606.ENSP00000351273 9606.ENSP00000264832 0 0 0 0 0.064 0 0 0.426 0.44

CASP8 EGF 9606.ENSP00000351273 9606.ENSP00000265171 0 0 0 0 0 0 0 0.511 0.511

CASP8 TP53 9606.ENSP00000351273 9606.ENSP00000269305 0 0 0 0 0.143 0.27 0.8 0.839 0.977

CASP8 ERBB2 9606.ENSP00000351273 9606.ENSP00000269571 0 0 0 0 0 0.06 0 0.522 0.531

CASP8 SOD1 9606.ENSP00000351273 9606.ENSP00000270142 0 0 0 0 0 0.102 0 0.415 0.452

CASP8 EGFR 9606.ENSP00000351273 9606.ENSP00000275493 0 0 0 0 0 0.06 0 0.597 0.605

CASP8 PPARG 9606.ENSP00000351273 9606.ENSP00000287820 0 0 0 0 0.062 0.06 0 0.403 0.427

CASP8 TLR3 9606.ENSP00000351273 9606.ENSP00000296795 0 0 0 0 0.062 0.292 0.6 0.959 0.987

CASP8 CSF2 9606.ENSP00000351273 9606.ENSP00000296871 0 0 0 0 0 0 0 0.423 0.423

CASP8 NOD2 9606.ENSP00000351273 9606.ENSP00000300589 0 0 0 0 0.083 0.056 0 0.395 0.43

CASP8 FADD 9606.ENSP00000351273 9606.ENSP00000301838 0 0 0 0 0.062 0.999 0.9 0.996 0.999

CASP8 IGF1 9606.ENSP00000351273 9606.ENSP00000302665 0 0 0 0 0 0 0 0.469 0.469

CASP8 COL3A1 9606.ENSP00000351273 9606.ENSP00000304408 0 0 0 0 0 0 0.9 0.086 0.904

CASP8 CXCL8 9606.ENSP00000351273 9606.ENSP00000306512 0 0 0 0 0.067 0 0 0.525 0.538

CASP8 CYCS 9606.ENSP00000351273 9606.ENSP00000307786 0 0 0 0 0.063 0 0 0.905 0.907

CASP8 LEP 9606.ENSP00000351273 9606.ENSP00000312652 0 0 0 0 0 0 0 0.458 0.459

CASP8 HSPA5 9606.ENSP00000351273 9606.ENSP00000324173 0 0 0 0 0 0.258 0 0.578 0.673

CASP8 CD28 9606.ENSP00000351273 9606.ENSP00000324890 0 0 0 0 0.096 0 0 0.398 0.433

CASP8 CASP9 9606.ENSP00000351273 9606.ENSP00000330237 0 0 0 0.707 0.053 0.488 0 0.961 0.636

CASP8 FAS 9606.ENSP00000351273 9606.ENSP00000347979 0 0 0 0 0.063 0.924 0.9 0.993 0.999

CASP8 GPT 9606.ENSP00000351273 9606.ENSP00000378408 0 0 0 0 0 0 0 0.403 0.403

CASP8 CCL5 9606.ENSP00000351273 9606.ENSP00000474412 0 0 0 0 0.107 0 0 0.376 0.42

CASP8 CD40LG 9606.ENSP00000351273 9606.ENSP00000359663 0 0 0 0 0.085 0.056 0 0.384 0.421

CASP8 CYP1B1 9606.ENSP00000351273 9606.ENSP00000478561 0 0 0 0 0 0 0 0.435 0.435

CASP8 SPP1 9606.ENSP00000351273 9606.ENSP00000378517 0 0 0 0 0 0.27 0 0.265 0.44

CASP8 LTA 9606.ENSP00000351273 9606.ENSP00000403495 0 0 0 0 0.062 0.058 0 0.424 0.446

CASP8 TOP1 9606.ENSP00000351273 9606.ENSP00000354522 0 0 0 0 0 0.103 0 0.408 0.446

CASP8 PRKCD 9606.ENSP00000351273 9606.ENSP00000378217 0 0 0 0 0.062 0 0 0.458 0.469

CASP8 CD40 9606.ENSP00000351273 9606.ENSP00000361359 0 0 0 0 0.062 0 0 0.471 0.482

CASP8 NOS1 9606.ENSP00000351273 9606.ENSP00000477999 0 0 0 0 0 0 0 0.486 0.485

CASP8 INS 9606.ENSP00000351273 9606.ENSP00000380432 0 0 0 0 0 0 0 0.489 0.489

CASP8 TNFRSF1B 9606.ENSP00000351273 9606.ENSP00000365435 0 0 0 0 0.086 0 0 0.469 0.494

CASP8 STAT1 9606.ENSP00000351273 9606.ENSP00000354394 0 0 0 0 0.062 0 0 0.484 0.495

CASP8 HIF1A 9606.ENSP00000351273 9606.ENSP00000437955 0 0 0 0 0.062 0 0 0.507 0.517

CASP8 ESR1 9606.ENSP00000351273 9606.ENSP00000405330 0 0 0 0 0 0.06 0 0.517 0.526

CASP8 IL10 9606.ENSP00000351273 9606.ENSP00000412237 0 0 0 0 0 0 0 0.54 0.54

CASP8 MMP9 9606.ENSP00000351273 9606.ENSP00000361405 0 0 0 0 0 0 0 0.544 0.544

CASP8 VEGFA 9606.ENSP00000351273 9606.ENSP00000478570 0 0 0 0 0 0 0 0.562 0.562

CASP8 CDKN1A 9606.ENSP00000351273 9606.ENSP00000384849 0 0 0 0 0 0 0 0.569 0.569

CASP8 MTOR 9606.ENSP00000351273 9606.ENSP00000354558 0 0 0 0 0 0.145 0 0.527 0.579

CASP8 MYD88 9606.ENSP00000351273 9606.ENSP00000401399 0 0 0 0 0.064 0.115 0 0.581 0.624

CASP8 PTEN 9606.ENSP00000351273 9606.ENSP00000361021 0 0 0 0 0.048 0.27 0 0.568 0.674

CASP8 IL6 9606.ENSP00000351273 9606.ENSP00000385675 0 0 0 0 0.062 0 0 0.699 0.706

CASP8 JUN 9606.ENSP00000351273 9606.ENSP00000360266 0 0 0 0 0 0.057 0 0.704 0.709

CASP8 PARP1 9606.ENSP00000351273 9606.ENSP00000355759 0 0 0 0 0 0.312 0 0.733 0.809

CASP8 TNFAIP3 9606.ENSP00000351273 9606.ENSP00000481570 0 0 0 0 0.092 0.68 0 0.403 0.811

CASP8 TLR4 9606.ENSP00000351273 9606.ENSP00000363089 0 0 0 0 0.062 0.07 0.6 0.612 0.846

CASP8 CHUK 9606.ENSP00000351273 9606.ENSP00000359424 0 0 0 0 0.053 0.317 0.9 0.83 0.987

CASP8 TNF 9606.ENSP00000351273 9606.ENSP00000398698 0 0 0 0 0.062 0.282 0.9 0.868 0.989

CASP8 FASLG 9606.ENSP00000351273 9606.ENSP00000356694 0 0 0 0 0 0.871 0.9 0.948 0.999

CASP9 TNFRSF1A 9606.ENSP00000330237 9606.ENSP00000162749 0 0 0 0 0 0 0 0.654 0.654

CASP9 HMOX1 9606.ENSP00000330237 9606.ENSP00000216117 0 0 0 0 0 0 0 0.561 0.561

CASP9 NFKBIA 9606.ENSP00000330237 9606.ENSP00000216797 0 0 0 0 0.062 0.09 0 0.596 0.625

CASP9 MMP2 9606.ENSP00000330237 9606.ENSP00000219070 0 0 0 0 0 0 0 0.519 0.519

CASP9 IL2 9606.ENSP00000330237 9606.ENSP00000226730 0 0 0 0 0 0 0 0.413 0.412

CASP9 CAT 9606.ENSP00000330237 9606.ENSP00000241052 0 0 0 0 0 0 0 0.665 0.665

CASP9 HSPB1 9606.ENSP00000330237 9606.ENSP00000248553 0 0 0 0 0 0.132 0 0.533 0.577

CASP9 MDM2 9606.ENSP00000330237 9606.ENSP00000258149 0 0 0 0 0.062 0 0 0.631 0.639

CASP9 IL1B 9606.ENSP00000330237 9606.ENSP00000263341 0 0 0 0 0 0.056 0 0.612 0.618

CASP9 EGF 9606.ENSP00000330237 9606.ENSP00000265171 0 0 0 0 0 0 0 0.504 0.504

CASP9 TP53 9606.ENSP00000330237 9606.ENSP00000269305 0 0 0 0 0.062 0 0 0.859 0.862

CASP9 ERBB2 9606.ENSP00000330237 9606.ENSP00000269571 0 0 0 0 0 0.06 0 0.527 0.536

CASP9 SOD1 9606.ENSP00000330237 9606.ENSP00000270142 0 0 0 0 0 0.102 0 0.418 0.455

CASP9 EGFR 9606.ENSP00000330237 9606.ENSP00000275493 0 0 0 0 0 0.06 0 0.615 0.623

CASP9 PPARG 9606.ENSP00000330237 9606.ENSP00000287820 0 0 0 0 0 0.06 0 0.421 0.432

CASP9 NOS3 9606.ENSP00000330237 9606.ENSP00000297494 0 0 0 0 0 0 0 0.459 0.459

CASP9 FADD 9606.ENSP00000330237 9606.ENSP00000301838 0 0 0 0 0.053 0.123 0 0.855 0.869

CASP9 IGF1 9606.ENSP00000330237 9606.ENSP00000302665 0 0 0 0 0 0 0 0.517 0.517

CASP9 CXCL8 9606.ENSP00000330237 9606.ENSP00000306512 0 0 0 0 0 0 0 0.467 0.467

CASP9 CYCS 9606.ENSP00000330237 9606.ENSP00000307786 0 0 0 0 0.063 0.68 0.9 0.991 0.999

CASP9 HSPA5 9606.ENSP00000330237 9606.ENSP00000324173 0 0 0 0 0 0.153 0 0.669 0.708

CASP9 STAT1 9606.ENSP00000330237 9606.ENSP00000354394 0 0 0 0 0 0 0 0.401 0.401

CASP9 LTA 9606.ENSP00000330237 9606.ENSP00000403495 0 0 0 0 0 0.058 0 0.399 0.41

CASP9 NGF 9606.ENSP00000330237 9606.ENSP00000358525 0 0 0 0 0 0 0 0.431 0.431

CASP9 MYD88 9606.ENSP00000330237 9606.ENSP00000401399 0 0 0 0 0.062 0.115 0 0.377 0.438

CASP9 TOP1 9606.ENSP00000330237 9606.ENSP00000354522 0 0 0 0 0 0.103 0 0.404 0.442

CASP9 GPT 9606.ENSP00000330237 9606.ENSP00000378408 0 0 0 0 0 0 0 0.45 0.45

CASP9 IL10 9606.ENSP00000330237 9606.ENSP00000412237 0 0 0 0 0 0 0 0.462 0.462

CASP9 PRKCD 9606.ENSP00000330237 9606.ENSP00000378217 0 0 0 0 0.062 0 0 0.459 0.47

CASP9 TLR4 9606.ENSP00000330237 9606.ENSP00000363089 0 0 0 0 0 0.07 0 0.462 0.478

CASP9 CHUK 9606.ENSP00000330237 9606.ENSP00000359424 0 0 0 0 0 0.103 0 0.459 0.493

CASP9 ESR1 9606.ENSP00000330237 9606.ENSP00000405330 0 0 0 0 0 0.06 0 0.504 0.513

CASP9 HIF1A 9606.ENSP00000330237 9606.ENSP00000437955 0 0 0 0 0 0 0 0.523 0.523

CASP9 INS 9606.ENSP00000330237 9606.ENSP00000380432 0 0 0 0 0 0 0 0.528 0.528

CASP9 MTOR 9606.ENSP00000330237 9606.ENSP00000354558 0 0 0 0 0 0.057 0 0.595 0.601

CASP9 VEGFA 9606.ENSP00000330237 9606.ENSP00000478570 0 0 0 0 0 0 0 0.602 0.602

CASP9 CDKN1A 9606.ENSP00000330237 9606.ENSP00000384849 0 0 0 0 0 0 0 0.618 0.618

CASP9 MMP9 9606.ENSP00000330237 9606.ENSP00000361405 0 0 0 0 0 0 0 0.619 0.619

CASP9 IL6 9606.ENSP00000330237 9606.ENSP00000385675 0 0 0 0 0 0 0 0.624 0.624

CASP9 PTEN 9606.ENSP00000330237 9606.ENSP00000361021 0 0 0 0 0.048 0 0 0.628 0.63

CASP9 FAS 9606.ENSP00000330237 9606.ENSP00000347979 0 0 0 0 0 0.059 0 0.716 0.721

CASP9 TNF 9606.ENSP00000330237 9606.ENSP00000398698 0 0 0 0 0 0.058 0 0.716 0.721

CASP9 FASLG 9606.ENSP00000330237 9606.ENSP00000356694 0 0 0 0 0 0 0 0.739 0.739

CASP9 JUN 9606.ENSP00000330237 9606.ENSP00000360266 0 0 0 0 0 0.329 0 0.688 0.782

CASP9 PARP1 9606.ENSP00000330237 9606.ENSP00000355759 0 0 0 0 0 0.097 0.8 0.742 0.949

CAT HMOX1 9606.ENSP00000241052 9606.ENSP00000216117 0 0 0 0 0.062 0.101 0 0.882 0.892

CAT NFKBIA 9606.ENSP00000241052 9606.ENSP00000216797 0.049 0 0 0 0.065 0.063 0 0.538 0.564

CAT MMP2 9606.ENSP00000241052 9606.ENSP00000219070 0 0 0 0 0 0 0 0.46 0.459

CAT TGFB1 9606.ENSP00000241052 9606.ENSP00000221930 0 0 0 0 0 0 0 0.436 0.436

CAT PON1 9606.ENSP00000241052 9606.ENSP00000222381 0 0 0 0 0.062 0 0 0.596 0.604

CAT MPO 9606.ENSP00000241052 9606.ENSP00000225275 0 0 0 0 0.061 0.241 0 0.702 0.769

CAT CCL2 9606.ENSP00000241052 9606.ENSP00000225831 0 0 0 0 0 0 0 0.564 0.564

CAT IL2 9606.ENSP00000241052 9606.ENSP00000226730 0 0 0 0 0 0 0 0.467 0.467

CAT IFNG 9606.ENSP00000241052 9606.ENSP00000229135 0 0 0 0 0 0 0 0.514 0.514

CAT IL4 9606.ENSP00000241052 9606.ENSP00000231449 0 0 0 0 0 0 0 0.468 0.468

CAT CTSD 9606.ENSP00000241052 9606.ENSP00000236671 0 0 0 0 0.086 0.138 0 0.381 0.47

CAT FASLG 9606.ENSP00000241052 9606.ENSP00000356694 0 0 0 0 0 0 0 0.4 0.4

CAT CYP1A2 9606.ENSP00000241052 9606.ENSP00000342007 0.047 0 0 0 0 0 0 0.398 0.401

CAT IL17A 9606.ENSP00000241052 9606.ENSP00000344192 0 0 0 0 0 0 0 0.408 0.408

CAT SELE 9606.ENSP00000241052 9606.ENSP00000331736 0 0 0 0 0.049 0 0 0.41 0.415

CAT CYP3A4 9606.ENSP00000241052 9606.ENSP00000337915 0.047 0 0 0 0.049 0 0 0.43 0.438

CAT EGFR 9606.ENSP00000241052 9606.ENSP00000275493 0 0 0 0 0.062 0 0 0.431 0.443

CAT IL1A 9606.ENSP00000241052 9606.ENSP00000263339 0 0 0 0 0 0 0 0.45 0.45

CAT NGF 9606.ENSP00000241052 9606.ENSP00000358525 0 0 0 0 0 0 0 0.455 0.455

CAT TH 9606.ENSP00000241052 9606.ENSP00000370571 0 0 0 0 0.062 0 0 0.447 0.459

CAT OCLN 9606.ENSP00000241052 9606.ENSP00000347379 0 0 0 0 0.062 0 0 0.451 0.463

CAT EGF 9606.ENSP00000241052 9606.ENSP00000265171 0 0 0 0 0 0 0 0.465 0.465

CAT SREBF1 9606.ENSP00000241052 9606.ENSP00000348069 0 0 0 0 0.062 0 0 0.474 0.486

CAT PARP1 9606.ENSP00000241052 9606.ENSP00000355759 0 0 0 0 0 0 0 0.487 0.487

CAT SNCA 9606.ENSP00000241052 9606.ENSP00000338345 0 0 0 0 0 0 0 0.508 0.508

CAT HSPB1 9606.ENSP00000241052 9606.ENSP00000248553 0 0 0 0 0 0 0 0.508 0.508

CAT P4HB 9606.ENSP00000241052 9606.ENSP00000327801 0 0 0 0 0.066 0.132 0 0.454 0.519

CAT VCAM1 9606.ENSP00000241052 9606.ENSP00000294728 0 0 0 0 0 0 0 0.522 0.522

CAT LEP 9606.ENSP00000241052 9606.ENSP00000312652 0 0 0 0 0 0 0 0.529 0.529

CAT IGF1 9606.ENSP00000241052 9606.ENSP00000302665 0 0 0 0 0.062 0 0 0.529 0.539

CAT MTOR 9606.ENSP00000241052 9606.ENSP00000354558 0 0 0 0 0 0 0 0.545 0.545

CAT ESR1 9606.ENSP00000241052 9606.ENSP00000405330 0 0 0 0 0.062 0.27 0 0.4 0.553

CAT EDN1 9606.ENSP00000241052 9606.ENSP00000368683 0 0 0 0 0 0 0 0.56 0.56

CAT MMP9 9606.ENSP00000241052 9606.ENSP00000361405 0 0 0 0 0 0 0 0.566 0.566

CAT ICAM1 9606.ENSP00000241052 9606.ENSP00000264832 0 0 0 0 0 0 0 0.574 0.574

CAT TLR4 9606.ENSP00000241052 9606.ENSP00000363089 0 0 0 0 0.06 0.056 0 0.562 0.577

CAT VEGFA 9606.ENSP00000241052 9606.ENSP00000478570 0 0 0 0 0 0 0 0.601 0.601

CAT REN 9606.ENSP00000241052 9606.ENSP00000272190 0 0 0 0 0.062 0.138 0 0.547 0.602

CAT LTA 9606.ENSP00000241052 9606.ENSP00000403495 0 0 0 0 0 0 0 0.602 0.602

CAT NOS1 9606.ENSP00000241052 9606.ENSP00000477999 0 0 0 0 0.049 0 0 0.598 0.602

CAT CXCL8 9606.ENSP00000241052 9606.ENSP00000306512 0 0 0 0 0 0 0 0.603 0.603

CAT CRP 9606.ENSP00000241052 9606.ENSP00000255030 0 0 0 0 0.062 0 0 0.605 0.614

CAT GSTM1 9606.ENSP00000241052 9606.ENSP00000311469 0 0 0 0 0.056 0.178 0 0.556 0.625

CAT HSPA5 9606.ENSP00000241052 9606.ENSP00000324173 0 0 0 0 0.062 0 0 0.618 0.626

CAT IL10 9606.ENSP00000241052 9606.ENSP00000412237 0 0 0 0 0 0 0 0.633 0.633

CAT PTEN 9606.ENSP00000241052 9606.ENSP00000361021 0 0 0 0 0.053 0.27 0 0.514 0.635

CAT HIF1A 9606.ENSP00000241052 9606.ENSP00000437955 0 0 0 0 0 0 0 0.648 0.648

CAT PPARG 9606.ENSP00000241052 9606.ENSP00000287820 0 0 0 0 0 0 0 0.655 0.655

CAT JUN 9606.ENSP00000241052 9606.ENSP00000360266 0 0 0 0 0 0 0 0.66 0.66

CAT NCF1 9606.ENSP00000241052 9606.ENSP00000289473 0 0 0 0 0 0 0 0.675 0.675

CAT TP53 9606.ENSP00000241052 9606.ENSP00000269305 0 0 0 0 0 0 0 0.689 0.689

CAT XDH 9606.ENSP00000241052 9606.ENSP00000368727 0 0 0 0 0.062 0 0 0.697 0.703

CAT IL1B 9606.ENSP00000241052 9606.ENSP00000263341 0 0 0 0 0 0 0 0.734 0.734

CAT NOS3 9606.ENSP00000241052 9606.ENSP00000297494 0 0 0 0 0.049 0 0 0.744 0.746

CAT IL6 9606.ENSP00000241052 9606.ENSP00000385675 0 0 0 0 0 0 0 0.756 0.756

CAT TNF 9606.ENSP00000241052 9606.ENSP00000398698 0 0 0 0 0 0 0 0.779 0.779

CAT GPT 9606.ENSP00000241052 9606.ENSP00000378408 0 0 0 0 0.064 0.349 0 0.727 0.819

CAT INS 9606.ENSP00000241052 9606.ENSP00000380432 0 0 0 0 0 0 0 0.84 0.84

CAT CYCS 9606.ENSP00000241052 9606.ENSP00000307786 0 0 0 0 0.156 0.272 0 0.873 0.915

CAT NOS2 9606.ENSP00000241052 9606.ENSP00000327251 0 0 0 0 0.049 0 0.9 0.558 0.954

CAT SOD1 9606.ENSP00000241052 9606.ENSP00000270142 0.111 0 0 0 0.124 0.52 0.9 0.923 0.996

CAV3 POLG 9606.ENSP00000341940 9606.ENSP00000268124 0 0 0 0 0 0 0 0.427 0.427

CAV3 EGFR 9606.ENSP00000341940 9606.ENSP00000275493 0 0 0 0 0 0.27 0.8 0.199 0.872

CAV3 NOS3 9606.ENSP00000341940 9606.ENSP00000297494 0 0 0 0 0.062 0.176 0.8 0.988 0.998

CAV3 SCN1A 9606.ENSP00000341940 9606.ENSP00000303540 0 0 0 0 0.062 0.136 0 0.404 0.474

CAV3 CYCS 9606.ENSP00000341940 9606.ENSP00000307786 0 0 0 0 0 0 0 0.424 0.424

CAV3 NOS2 9606.ENSP00000341940 9606.ENSP00000327251 0 0 0 0 0.063 0.098 0 0.386 0.435

CAV3 KCND2 9606.ENSP00000341940 9606.ENSP00000333496 0 0 0 0 0.062 0.076 0 0.889 0.895

CAV3 SCN9A 9606.ENSP00000341940 9606.ENSP00000386306 0 0 0 0 0.062 0.136 0 0.375 0.449

CAV3 SCN10A 9606.ENSP00000341940 9606.ENSP00000390600 0 0 0 0 0 0.136 0 0.391 0.451

CAV3 KCNJ10 9606.ENSP00000341940 9606.ENSP00000357068 0 0 0 0 0.051 0 0 0.476 0.482

CAV3 HTR2A 9606.ENSP00000341940 9606.ENSP00000437737 0 0 0 0 0 0.057 0 0.475 0.484

CAV3 VDR 9606.ENSP00000341940 9606.ENSP00000447173 0 0 0 0 0 0 0 0.633 0.633

CAV3 ESR1 9606.ENSP00000341940 9606.ENSP00000405330 0 0 0 0 0 0 0 0.702 0.702

CAV3 RYR1 9606.ENSP00000341940 9606.ENSP00000352608 0 0 0 0 0.064 0.27 0 0.691 0.771

CAV3 NOS1 9606.ENSP00000341940 9606.ENSP00000477999 0 0 0 0 0.054 0.296 0 0.937 0.954

CCL2 TNFRSF1A 9606.ENSP00000225831 9606.ENSP00000162749 0 0 0 0 0.086 0 0 0.612 0.63

CCL2 MIF 9606.ENSP00000225831 9606.ENSP00000215754 0 0 0 0 0 0 0 0.7 0.7

CCL2 HMOX1 9606.ENSP00000225831 9606.ENSP00000216117 0 0 0 0 0.095 0 0 0.668 0.686

CCL2 NFKBIA 9606.ENSP00000225831 9606.ENSP00000216797 0 0 0 0 0.063 0 0 0.702 0.709

CCL2 MMP2 9606.ENSP00000225831 9606.ENSP00000219070 0 0 0 0 0.155 0 0 0.667 0.706

CCL2 TGFB1 9606.ENSP00000225831 9606.ENSP00000221930 0 0 0 0 0.049 0 0 0.653 0.655

CCL2 PON1 9606.ENSP00000225831 9606.ENSP00000222381 0 0 0 0 0 0 0 0.452 0.452

CCL2 MPO 9606.ENSP00000225831 9606.ENSP00000225275 0 0 0 0 0 0 0 0.682 0.682

CCL2 FAS 9606.ENSP00000225831 9606.ENSP00000347979 0 0 0 0 0.051 0 0 0.396 0.402

CCL2 S100A9 9606.ENSP00000225831 9606.ENSP00000357727 0 0 0 0 0.064 0 0 0.39 0.404

CCL2 ERBB2 9606.ENSP00000225831 9606.ENSP00000269571 0 0 0 0 0 0 0 0.409 0.408

CCL2 STAT5A 9606.ENSP00000225831 9606.ENSP00000341208 0 0 0 0 0 0 0 0.413 0.412

CCL2 NR3C1 9606.ENSP00000225831 9606.ENSP00000231509 0 0 0 0 0.055 0 0 0.406 0.414

CCL2 F2 9606.ENSP00000225831 9606.ENSP00000308541 0 0 0 0 0 0 0 0.416 0.416

CCL2 SOD1 9606.ENSP00000225831 9606.ENSP00000270142 0 0 0 0 0 0 0 0.427 0.426

CCL2 CYCS 9606.ENSP00000225831 9606.ENSP00000307786 0 0 0 0 0 0 0 0.454 0.454

CCL2 COL3A1 9606.ENSP00000225831 9606.ENSP00000304408 0 0 0 0 0.111 0 0 0.417 0.459

CCL2 MTOR 9606.ENSP00000225831 9606.ENSP00000354558 0 0 0 0 0 0 0 0.459 0.459

CCL2 PTEN 9606.ENSP00000225831 9606.ENSP00000361021 0 0 0 0 0.062 0 0 0.455 0.466

CCL2 ESR1 9606.ENSP00000225831 9606.ENSP00000405330 0 0 0 0 0.055 0 0 0.46 0.467

CCL2 KIT 9606.ENSP00000225831 9606.ENSP00000288135 0 0 0 0 0 0 0 0.468 0.468

CCL2 ITGB2 9606.ENSP00000225831 9606.ENSP00000380948 0 0 0 0 0.062 0 0 0.458 0.469

CCL2 SOCS1 9606.ENSP00000225831 9606.ENSP00000329418 0 0 0 0 0.062 0 0 0.467 0.478

CCL2 LPL 9606.ENSP00000225831 9606.ENSP00000309757 0 0 0 0 0.065 0 0 0.468 0.481

CCL2 CHUK 9606.ENSP00000225831 9606.ENSP00000359424 0 0 0 0 0 0 0 0.495 0.495

CCL2 TNFAIP3 9606.ENSP00000225831 9606.ENSP00000481570 0 0 0 0 0.097 0 0 0.467 0.498

CCL2 IL17RA 9606.ENSP00000225831 9606.ENSP00000320936 0 0 0 0 0 0 0 0.499 0.499

CCL2 ELANE 9606.ENSP00000225831 9606.ENSP00000466090 0 0 0 0 0 0 0 0.502 0.502

CCL2 NCF1 9606.ENSP00000225831 9606.ENSP00000289473 0 0 0 0 0.062 0 0 0.504 0.514

CCL2 HPRT1 9606.ENSP00000225831 9606.ENSP00000298556 0 0 0 0 0 0 0 0.515 0.515

CCL2 OCLN 9606.ENSP00000225831 9606.ENSP00000347379 0 0 0 0 0 0 0 0.517 0.517

CCL2 SREBF1 9606.ENSP00000225831 9606.ENSP00000348069 0 0 0 0 0 0 0 0.519 0.519

CCL2 ITGAL 9606.ENSP00000225831 9606.ENSP00000349252 0 0 0 0 0 0 0 0.522 0.522

CCL2 EGFR 9606.ENSP00000225831 9606.ENSP00000275493 0 0 0 0 0.062 0 0 0.517 0.527

CCL2 NOD2 9606.ENSP00000225831 9606.ENSP00000300589 0 0 0 0 0.066 0 0 0.518 0.53

CCL2 KITLG 9606.ENSP00000225831 9606.ENSP00000228280 0 0 0 0 0.07 0 0 0.518 0.532

CCL2 THBD 9606.ENSP00000225831 9606.ENSP00000366307 0 0 0 0 0.095 0 0 0.504 0.532

CCL2 CD28 9606.ENSP00000225831 9606.ENSP00000324890 0 0 0 0 0.086 0 0 0.521 0.543

CCL2 FASLG 9606.ENSP00000225831 9606.ENSP00000356694 0 0 0 0 0 0 0 0.543 0.543

CCL2 COL1A1 9606.ENSP00000225831 9606.ENSP00000225964 0 0 0 0 0.098 0 0 0.519 0.547

CCL2 IDO1 9606.ENSP00000225831 9606.ENSP00000430950 0 0 0 0 0.09 0 0 0.526 0.55

CCL2 TNFRSF1B 9606.ENSP00000225831 9606.ENSP00000365435 0 0 0 0 0.053 0 0 0.558 0.563

CCL2 GPT 9606.ENSP00000225831 9606.ENSP00000378408 0 0 0 0 0 0 0 0.601 0.601

CCL2 IGF1 9606.ENSP00000225831 9606.ENSP00000302665 0 0 0 0 0 0 0 0.611 0.611

CCL2 NGF 9606.ENSP00000225831 9606.ENSP00000358525 0 0 0 0 0.076 0 0 0.601 0.615

CCL2 NOS2 9606.ENSP00000225831 9606.ENSP00000327251 0 0 0 0 0 0 0 0.624 0.624

CCL2 HIF1A 9606.ENSP00000225831 9606.ENSP00000437955 0 0 0 0 0.062 0 0 0.618 0.626

CCL2 REN 9606.ENSP00000225831 9606.ENSP00000272190 0 0 0 0 0 0 0 0.636 0.636

CCL2 TP53 9606.ENSP00000225831 9606.ENSP00000269305 0 0 0 0 0 0 0 0.644 0.644

CCL2 LCN2 9606.ENSP00000225831 9606.ENSP00000362108 0 0 0 0 0.06 0 0 0.653 0.659

CCL2 FOXP3 9606.ENSP00000225831 9606.ENSP00000365380 0 0 0 0 0 0 0 0.663 0.663

CCL2 STAT1 9606.ENSP00000225831 9606.ENSP00000354394 0 0 0 0 0.063 0 0 0.664 0.672

CCL2 IL1RN 9606.ENSP00000225831 9606.ENSP00000259206 0 0 0 0 0.099 0 0 0.652 0.673

CCL2 EDN1 9606.ENSP00000225831 9606.ENSP00000368683 0 0 0 0 0.088 0 0 0.661 0.678

CCL2 EGF 9606.ENSP00000225831 9606.ENSP00000265171 0 0 0 0 0 0 0 0.681 0.681

CCL2 NOS3 9606.ENSP00000225831 9606.ENSP00000297494 0 0 0 0 0 0 0 0.681 0.681

CCL2 CD40LG 9606.ENSP00000225831 9606.ENSP00000359663 0 0 0 0 0 0.057 0 0.681 0.686

CCL2 FGF2 9606.ENSP00000225831 9606.ENSP00000264498 0 0 0 0 0 0 0 0.687 0.687

CCL2 TLR3 9606.ENSP00000225831 9606.ENSP00000296795 0 0 0 0 0.056 0 0 0.683 0.688

CCL2 SPP1 9606.ENSP00000225831 9606.ENSP00000378517 0 0 0 0 0.119 0 0 0.663 0.69

CCL2 SELP 9606.ENSP00000225831 9606.ENSP00000263686 0 0 0 0 0.056 0 0 0.693 0.698

CCL2 MYD88 9606.ENSP00000225831 9606.ENSP00000401399 0 0 0 0 0.062 0 0 0.692 0.699

CCL2 MMP1 9606.ENSP00000225831 9606.ENSP00000322788 0 0 0 0 0.109 0.213 0 0.614 0.705

CCL2 PF4 9606.ENSP00000225831 9606.ENSP00000296029 0 0 0 0 0.062 0.237 0 0.622 0.706

CCL2 PPARG 9606.ENSP00000225831 9606.ENSP00000287820 0 0 0 0 0.055 0 0 0.705 0.709

CCL2 LTA 9606.ENSP00000225831 9606.ENSP00000403495 0 0 0 0 0.062 0 0 0.711 0.717

CCL2 IRF1 9606.ENSP00000225831 9606.ENSP00000245414 0 0 0 0 0.072 0.27 0 0.62 0.72

CCL2 INS 9606.ENSP00000225831 9606.ENSP00000380432 0 0 0 0 0 0 0 0.741 0.741

CCL2 LEP 9606.ENSP00000225831 9606.ENSP00000312652 0 0 0 0 0 0 0 0.754 0.754

CCL2 CXCL13 9606.ENSP00000225831 9606.ENSP00000286758 0 0 0 0 0 0.28 0 0.7 0.775

CCL2 SELE 9606.ENSP00000225831 9606.ENSP00000331736 0 0 0 0 0.109 0 0 0.767 0.784

CCL2 MMP9 9606.ENSP00000225831 9606.ENSP00000361405 0 0 0 0 0.076 0 0 0.779 0.787

CCL2 MMP3 9606.ENSP00000225831 9606.ENSP00000299855 0 0 0 0 0.107 0.213 0 0.731 0.795

CCL2 CD40 9606.ENSP00000225831 9606.ENSP00000361359 0 0 0 0 0.062 0 0 0.792 0.796

CCL2 CRP 9606.ENSP00000225831 9606.ENSP00000255030 0 0 0 0 0 0 0 0.805 0.805

CCL2 IL2 9606.ENSP00000225831 9606.ENSP00000226730 0 0 0 0 0 0 0 0.81 0.81

CCL2 TLR4 9606.ENSP00000225831 9606.ENSP00000363089 0 0 0 0 0.097 0 0 0.818 0.829

CCL2 IFNG 9606.ENSP00000225831 9606.ENSP00000229135 0 0 0 0 0.063 0 0 0.835 0.839

CCL2 IL17A 9606.ENSP00000225831 9606.ENSP00000344192 0 0 0 0 0 0 0 0.856 0.856

CCL2 VEGFA 9606.ENSP00000225831 9606.ENSP00000478570 0 0 0 0 0 0 0 0.856 0.856

CCL2 CCR7 9606.ENSP00000225831 9606.ENSP00000246657 0 0 0 0 0.062 0 0.6 0.66 0.861

CCL2 ICAM1 9606.ENSP00000225831 9606.ENSP00000264832 0 0 0 0 0.148 0 0 0.859 0.874

CCL2 NFKB1 9606.ENSP00000225831 9606.ENSP00000226574 0 0 0 0 0.063 0 0.8 0.471 0.892

CCL2 VCAM1 9606.ENSP00000225831 9606.ENSP00000294728 0 0 0 0 0.202 0 0 0.879 0.899

CCL2 CXCR4 9606.ENSP00000225831 9606.ENSP00000386884 0 0 0 0 0.062 0.057 0.6 0.804 0.921

CCL2 CCL3 9606.ENSP00000225831 9606.ENSP00000477908 0 0 0 0.847 0.117 0 0.9 0.933 0.921

CCL2 CX3CR1 9606.ENSP00000225831 9606.ENSP00000351059 0 0 0 0 0 0 0.6 0.86 0.941

CCL2 CCL5 9606.ENSP00000225831 9606.ENSP00000474412 0 0 0 0.831 0.066 0.45 0.9 0.931 0.952

CCL2 JUN 9606.ENSP00000225831 9606.ENSP00000360266 0 0 0 0 0.062 0 0.9 0.686 0.968

CCL2 IL1A 9606.ENSP00000225831 9606.ENSP00000263339 0 0 0 0 0.092 0 0.9 0.837 0.983

CCL2 IL13 9606.ENSP00000225831 9606.ENSP00000304915 0 0 0 0 0 0 0.9 0.841 0.983

CCL2 IL4 9606.ENSP00000225831 9606.ENSP00000231449 0 0 0 0 0 0 0.9 0.86 0.985

CCL2 CSF2 9606.ENSP00000225831 9606.ENSP00000296871 0 0 0 0 0.063 0 0.9 0.882 0.988

CCL2 IL10 9606.ENSP00000225831 9606.ENSP00000412237 0 0 0 0 0 0 0.9 0.901 0.989

CCL2 CXCL10 9606.ENSP00000225831 9606.ENSP00000305651 0 0 0 0 0.16 0 0.9 0.91 0.991

CCL2 TNF 9606.ENSP00000225831 9606.ENSP00000398698 0 0 0 0 0.062 0 0.9 0.93 0.992

CCL2 IL1B 9606.ENSP00000225831 9606.ENSP00000263341 0 0 0 0 0.127 0 0.9 0.927 0.993

CCL2 IL6 9606.ENSP00000225831 9606.ENSP00000385675 0 0 0 0 0.267 0 0.9 0.93 0.994

CCL2 CXCL8 9606.ENSP00000225831 9606.ENSP00000306512 0 0 0 0 0.271 0.28 0.9 0.923 0.995

CCL3 TNFRSF1A 9606.ENSP00000477908 9606.ENSP00000162749 0 0 0 0 0.058 0 0 0.501 0.509

CCL3 MIF 9606.ENSP00000477908 9606.ENSP00000215754 0 0 0 0 0 0 0 0.527 0.527

CCL3 HMOX1 9606.ENSP00000477908 9606.ENSP00000216117 0 0 0 0 0.08 0 0 0.38 0.405

CCL3 NFKBIA 9606.ENSP00000477908 9606.ENSP00000216797 0 0 0 0 0.152 0 0 0.515 0.571

CCL3 MMP2 9606.ENSP00000477908 9606.ENSP00000219070 0 0 0 0 0 0 0 0.421 0.42

CCL3 TGFB1 9606.ENSP00000477908 9606.ENSP00000221930 0 0 0 0 0.082 0.213 0 0.457 0.574

CCL3 MPO 9606.ENSP00000477908 9606.ENSP00000225275 0 0 0 0 0.077 0 0 0.577 0.593

CCL3 IL2 9606.ENSP00000477908 9606.ENSP00000226730 0 0 0 0 0 0 0 0.832 0.832

CCL3 KITLG 9606.ENSP00000477908 9606.ENSP00000228280 0 0 0 0 0 0 0 0.52 0.52

CCL3 IFNG 9606.ENSP00000477908 9606.ENSP00000229135 0 0 0 0 0.261 0 0 0.773 0.825

CCL3 IL4 9606.ENSP00000477908 9606.ENSP00000231449 0 0 0 0 0 0 0 0.83 0.83

CCL3 IRF1 9606.ENSP00000477908 9606.ENSP00000245414 0 0 0 0 0.108 0 0 0.414 0.455

CCL3 CCR7 9606.ENSP00000477908 9606.ENSP00000246657 0 0 0 0 0.129 0 0.6 0.675 0.877

CCL3 CRP 9606.ENSP00000477908 9606.ENSP00000255030 0 0 0 0 0 0 0 0.568 0.568

CCL3 IL1RN 9606.ENSP00000477908 9606.ENSP00000259206 0 0 0 0 0.234 0 0 0.581 0.665

CCL3 IL1A 9606.ENSP00000477908 9606.ENSP00000263339 0 0 0 0 0.124 0 0.9 0.837 0.984

CCL3 IL1B 9606.ENSP00000477908 9606.ENSP00000263341 0 0 0 0 0.685 0 0.9 0.885 0.996

CCL3 SELP 9606.ENSP00000477908 9606.ENSP00000263686 0 0 0 0 0.089 0 0 0.481 0.507

CCL3 FGF2 9606.ENSP00000477908 9606.ENSP00000264498 0 0 0 0 0 0 0 0.609 0.609

CCL3 ICAM1 9606.ENSP00000477908 9606.ENSP00000264832 0 0 0 0 0.118 0 0 0.692 0.717

CCL3 EGF 9606.ENSP00000477908 9606.ENSP00000265171 0 0 0 0 0 0 0 0.571 0.571

CCL3 CXCL13 9606.ENSP00000477908 9606.ENSP00000286758 0 0 0 0 0.085 0 0 0.687 0.701

CCL3 PPARG 9606.ENSP00000477908 9606.ENSP00000287820 0 0 0 0 0 0 0 0.406 0.406

CCL3 VCAM1 9606.ENSP00000477908 9606.ENSP00000294728 0 0 0 0 0.062 0 0 0.644 0.652

CCL3 PF4 9606.ENSP00000477908 9606.ENSP00000296029 0 0 0 0 0.081 0 0 0.626 0.642

CCL3 TLR3 9606.ENSP00000477908 9606.ENSP00000296795 0 0 0 0 0 0 0 0.617 0.617

CCL3 CSF2 9606.ENSP00000477908 9606.ENSP00000296871 0 0 0 0 0.12 0 0.9 0.876 0.988

CCL3 MMP3 9606.ENSP00000477908 9606.ENSP00000299855 0 0 0 0 0.063 0 0 0.487 0.499

CCL3 NOD2 9606.ENSP00000477908 9606.ENSP00000300589 0 0 0 0 0.185 0 0 0.383 0.476

CCL3 IGF1 9606.ENSP00000477908 9606.ENSP00000302665 0 0 0 0 0 0 0 0.423 0.423

CCL3 IL13 9606.ENSP00000477908 9606.ENSP00000304915 0 0 0 0 0 0 0 0.844 0.844

CCL3 CXCL10 9606.ENSP00000477908 9606.ENSP00000305651 0 0 0 0 0.237 0 0.9 0.906 0.992

CCL3 CXCL8 9606.ENSP00000477908 9606.ENSP00000306512 0 0 0 0 0.187 0 0.9 0.876 0.989

CCL3 LEP 9606.ENSP00000477908 9606.ENSP00000312652 0 0 0 0 0 0 0 0.514 0.514

CCL3 IL17RA 9606.ENSP00000477908 9606.ENSP00000320936 0 0 0 0 0.085 0 0 0.413 0.441

CCL3 MMP1 9606.ENSP00000477908 9606.ENSP00000322788 0 0 0 0 0.062 0 0 0.392 0.405

CCL3 CD28 9606.ENSP00000477908 9606.ENSP00000324890 0 0 0 0 0.062 0 0 0.535 0.545

CCL3 NOS2 9606.ENSP00000477908 9606.ENSP00000327251 0 0 0 0 0.062 0 0 0.491 0.503

CCL3 SOCS1 9606.ENSP00000477908 9606.ENSP00000329418 0 0 0 0 0.093 0 0 0.38 0.413

CCL3 SELE 9606.ENSP00000477908 9606.ENSP00000331736 0 0 0 0 0.054 0 0 0.538 0.545

CCL3 IL17A 9606.ENSP00000477908 9606.ENSP00000344192 0 0 0 0 0 0 0 0.84 0.84

CCL3 ITGAL 9606.ENSP00000477908 9606.ENSP00000349252 0 0 0 0 0.154 0 0 0.406 0.477

CCL3 CX3CR1 9606.ENSP00000477908 9606.ENSP00000351059 0 0 0 0 0 0 0.6 0.803 0.918

CCL3 STAT1 9606.ENSP00000477908 9606.ENSP00000354394 0 0 0 0 0.062 0 0 0.539 0.549

CCL3 FASLG 9606.ENSP00000477908 9606.ENSP00000356694 0 0 0 0 0.077 0 0 0.468 0.487

CCL3 NGF 9606.ENSP00000477908 9606.ENSP00000358525 0 0 0 0 0 0 0 0.47 0.47

CCL3 CD40LG 9606.ENSP00000477908 9606.ENSP00000359663 0 0 0 0 0.069 0 0 0.619 0.63

CCL3 JUN 9606.ENSP00000477908 9606.ENSP00000360266 0 0 0 0 0.049 0 0 0.479 0.483

CCL3 CD40 9606.ENSP00000477908 9606.ENSP00000361359 0 0 0 0 0.107 0 0 0.689 0.711

CCL3 MMP9 9606.ENSP00000477908 9606.ENSP00000361405 0 0 0 0 0.14 0 0 0.623 0.662

CCL3 LCN2 9606.ENSP00000477908 9606.ENSP00000362108 0 0 0 0 0.062 0 0 0.396 0.41

CCL3 TLR4 9606.ENSP00000477908 9606.ENSP00000363089 0 0 0 0 0.108 0 0 0.707 0.727

CCL3 FOXP3 9606.ENSP00000477908 9606.ENSP00000365380 0 0 0 0 0 0 0 0.577 0.577

CCL3 TNFRSF1B 9606.ENSP00000477908 9606.ENSP00000365435 0 0 0 0 0.222 0 0 0.459 0.561

CCL3 SPP1 9606.ENSP00000477908 9606.ENSP00000378517 0 0 0 0 0.065 0 0 0.473 0.486

CCL3 INS 9606.ENSP00000477908 9606.ENSP00000380432 0 0 0 0 0 0 0 0.452 0.452

CCL3 ITGB2 9606.ENSP00000477908 9606.ENSP00000380948 0 0 0 0 0.231 0 0 0.368 0.493

CCL3 IL6 9606.ENSP00000477908 9606.ENSP00000385675 0 0 0 0 0.139 0 0.9 0.885 0.989

CCL3 CXCR4 9606.ENSP00000477908 9606.ENSP00000386884 0 0 0 0 0.085 0 0.6 0.746 0.899

CCL3 TNF 9606.ENSP00000477908 9606.ENSP00000398698 0 0 0 0 0.407 0 0.9 0.903 0.993

CCL3 MYD88 9606.ENSP00000477908 9606.ENSP00000401399 0 0 0 0 0.081 0 0 0.624 0.64

CCL3 LTA 9606.ENSP00000477908 9606.ENSP00000403495 0 0 0 0 0.223 0 0 0.745 0.794

CCL3 IL10 9606.ENSP00000477908 9606.ENSP00000412237 0 0 0 0 0.099 0 0.9 0.879 0.988

CCL3 ELANE 9606.ENSP00000477908 9606.ENSP00000466090 0 0 0 0 0.065 0 0 0.419 0.434

CCL3 CCL5 9606.ENSP00000477908 9606.ENSP00000474412 0 0 0 0.94 0.163 0 0.9 0.937 0.917

CCL3 TNFAIP3 9606.ENSP00000477908 9606.ENSP00000481570 0 0 0 0 0.16 0 0 0.324 0.408

CCL3 VEGFA 9606.ENSP00000477908 9606.ENSP00000478570 0 0 0 0 0 0 0 0.738 0.738

CCL5 TNFRSF1A 9606.ENSP00000474412 9606.ENSP00000162749 0 0 0 0 0.062 0 0 0.557 0.566

CCL5 MIF 9606.ENSP00000474412 9606.ENSP00000215754 0 0 0 0 0 0 0 0.53 0.53

CCL5 HMOX1 9606.ENSP00000474412 9606.ENSP00000216117 0 0 0 0 0 0 0 0.401 0.4

CCL5 CTSG 9606.ENSP00000474412 9606.ENSP00000216336 0 0 0 0 0.069 0 0 0.408 0.425

CCL5 NFKBIA 9606.ENSP00000474412 9606.ENSP00000216797 0 0 0 0 0.066 0 0 0.608 0.618

CCL5 MMP2 9606.ENSP00000474412 9606.ENSP00000219070 0 0 0 0 0 0 0 0.506 0.506

CCL5 TGFB1 9606.ENSP00000474412 9606.ENSP00000221930 0 0 0 0 0.062 0.059 0 0.55 0.568

CCL5 MPO 9606.ENSP00000474412 9606.ENSP00000225275 0 0 0 0 0 0 0 0.556 0.556

CCL5 NFKB1 9606.ENSP00000474412 9606.ENSP00000226574 0 0 0 0 0.063 0 0.8 0.415 0.88

CCL5 IL2 9606.ENSP00000474412 9606.ENSP00000226730 0 0 0 0 0 0 0 0.826 0.826

CCL5 KITLG 9606.ENSP00000474412 9606.ENSP00000228280 0 0 0 0 0 0 0 0.503 0.503

CCL5 IFNG 9606.ENSP00000474412 9606.ENSP00000229135 0 0 0 0 0.149 0 0 0.796 0.819

CCL5 IL4 9606.ENSP00000474412 9606.ENSP00000231449 0 0 0 0 0 0 0 0.837 0.837

CCL5 IRF1 9606.ENSP00000474412 9606.ENSP00000245414 0 0 0 0 0.249 0 0 0.597 0.684

CCL5 CCR7 9606.ENSP00000474412 9606.ENSP00000246657 0 0 0 0 0.185 0 0.6 0.697 0.892

CCL5 CRP 9606.ENSP00000474412 9606.ENSP00000255030 0 0 0 0 0 0 0 0.566 0.566

CCL5 IL1RN 9606.ENSP00000474412 9606.ENSP00000259206 0 0 0 0 0.118 0 0 0.58 0.614

CCL5 IL1A 9606.ENSP00000474412 9606.ENSP00000263339 0 0 0 0 0.063 0 0.9 0.813 0.98

CCL5 IL1B 9606.ENSP00000474412 9606.ENSP00000263341 0 0 0 0 0.129 0 0.9 0.876 0.988

CCL5 SELP 9606.ENSP00000474412 9606.ENSP00000263686 0 0 0 0 0.096 0 0 0.687 0.705

CCL5 FGF2 9606.ENSP00000474412 9606.ENSP00000264498 0 0 0 0 0 0 0 0.624 0.624

CCL5 ICAM1 9606.ENSP00000474412 9606.ENSP00000264832 0 0 0 0 0.078 0 0 0.742 0.752

CCL5 EGF 9606.ENSP00000474412 9606.ENSP00000265171 0 0 0 0 0 0 0 0.606 0.606

CCL5 TP53 9606.ENSP00000474412 9606.ENSP00000269305 0 0 0 0 0 0 0 0.47 0.47

CCL5 EGFR 9606.ENSP00000474412 9606.ENSP00000275493 0 0 0 0 0 0 0 0.43 0.43

CCL5 CXCL13 9606.ENSP00000474412 9606.ENSP00000286758 0 0 0 0 0.088 0 0 0.681 0.697

CCL5 PPARG 9606.ENSP00000474412 9606.ENSP00000287820 0 0 0 0 0.062 0 0 0.471 0.482

CCL5 VCAM1 9606.ENSP00000474412 9606.ENSP00000294728 0 0 0 0 0.082 0 0 0.722 0.734

CCL5 PF4 9606.ENSP00000474412 9606.ENSP00000296029 0 0 0 0 0.096 0.467 0 0.995 0.997

CCL5 TLR3 9606.ENSP00000474412 9606.ENSP00000296795 0 0 0 0 0 0 0 0.711 0.711

CCL5 CSF2 9606.ENSP00000474412 9606.ENSP00000296871 0 0 0 0 0.062 0 0.9 0.871 0.986

CCL5 HPRT1 9606.ENSP00000474412 9606.ENSP00000298556 0 0 0 0 0 0 0 0.416 0.416

CCL5 MMP3 9606.ENSP00000474412 9606.ENSP00000299855 0 0 0 0 0.063 0 0 0.537 0.548

CCL5 NOD2 9606.ENSP00000474412 9606.ENSP00000300589 0 0 0 0 0.124 0 0 0.461 0.508

CCL5 IGF1 9606.ENSP00000474412 9606.ENSP00000302665 0 0 0 0 0 0 0 0.507 0.507

CCL5 IL13 9606.ENSP00000474412 9606.ENSP00000304915 0 0 0 0 0 0 0 0.844 0.844

CCL5 CXCL10 9606.ENSP00000474412 9606.ENSP00000305651 0 0 0 0 0.306 0.248 0.6 0.908 0.978

CCL5 CXCL8 9606.ENSP00000474412 9606.ENSP00000306512 0 0 0 0 0.065 0.281 0.6 0.888 0.966

CCL5 LEP 9606.ENSP00000474412 9606.ENSP00000312652 0 0 0 0 0 0 0 0.559 0.559

CCL5 IL17RA 9606.ENSP00000474412 9606.ENSP00000320936 0 0 0 0 0.071 0 0 0.393 0.411

CCL5 MMP1 9606.ENSP00000474412 9606.ENSP00000322788 0 0 0 0 0.049 0 0 0.505 0.509

CCL5 CD28 9606.ENSP00000474412 9606.ENSP00000324890 0 0 0 0 0.088 0 0 0.546 0.568

CCL5 NOS2 9606.ENSP00000474412 9606.ENSP00000327251 0 0 0 0 0.064 0 0 0.533 0.544

CCL5 SOCS1 9606.ENSP00000474412 9606.ENSP00000329418 0 0 0 0 0.064 0 0 0.458 0.47

CCL5 SELE 9606.ENSP00000474412 9606.ENSP00000331736 0 0 0 0 0.054 0 0 0.606 0.611

CCL5 IL17A 9606.ENSP00000474412 9606.ENSP00000344192 0 0 0 0 0 0 0 0.839 0.839

CCL5 ITGAL 9606.ENSP00000474412 9606.ENSP00000349252 0 0 0 0 0.236 0 0 0.452 0.563

CCL5 CX3CR1 9606.ENSP00000474412 9606.ENSP00000351059 0 0 0 0 0 0 0.6 0.671 0.862

CCL5 STAT1 9606.ENSP00000474412 9606.ENSP00000354394 0 0 0 0 0.14 0 0 0.669 0.704

CCL5 FASLG 9606.ENSP00000474412 9606.ENSP00000356694 0 0 0 0 0.136 0 0 0.509 0.557

CCL5 NGF 9606.ENSP00000474412 9606.ENSP00000358525 0 0 0 0 0 0 0 0.47 0.47

CCL5 CHUK 9606.ENSP00000474412 9606.ENSP00000359424 0 0 0 0 0.049 0 0 0.422 0.426

CCL5 CD40LG 9606.ENSP00000474412 9606.ENSP00000359663 0 0 0 0 0.111 0.056 0 0.664 0.693

CCL5 JUN 9606.ENSP00000474412 9606.ENSP00000360266 0 0 0 0 0 0 0.8 0.523 0.9

CCL5 CD40 9606.ENSP00000474412 9606.ENSP00000361359 0 0 0 0 0.093 0 0 0.668 0.686

CCL5 MMP9 9606.ENSP00000474412 9606.ENSP00000361405 0 0 0 0 0.076 0 0 0.666 0.679

CCL5 LCN2 9606.ENSP00000474412 9606.ENSP00000362108 0 0 0 0 0.062 0 0 0.423 0.435

CCL5 TLR4 9606.ENSP00000474412 9606.ENSP00000363089 0 0 0 0 0.062 0 0 0.728 0.734

CCL5 FOXP3 9606.ENSP00000474412 9606.ENSP00000365380 0 0 0 0 0 0 0 0.637 0.637

CCL5 TNFRSF1B 9606.ENSP00000474412 9606.ENSP00000365435 0 0 0 0 0.152 0 0 0.506 0.563

CCL5 EDN1 9606.ENSP00000474412 9606.ENSP00000368683 0 0 0 0 0 0 0 0.458 0.458

CCL5 SPP1 9606.ENSP00000474412 9606.ENSP00000378517 0 0 0 0 0 0 0 0.505 0.505

CCL5 INS 9606.ENSP00000474412 9606.ENSP00000380432 0 0 0 0 0 0 0 0.523 0.523

CCL5 ITGB2 9606.ENSP00000474412 9606.ENSP00000380948 0 0 0 0 0.177 0 0 0.396 0.481

CCL5 IL6 9606.ENSP00000474412 9606.ENSP00000385675 0 0 0 0 0.065 0 0.9 0.883 0.988

CCL5 CXCR4 9606.ENSP00000474412 9606.ENSP00000386884 0 0 0 0 0.062 0.056 0.6 0.896 0.958

CCL5 TNF 9606.ENSP00000474412 9606.ENSP00000398698 0 0 0 0 0.157 0 0.9 0.893 0.99

CCL5 MYD88 9606.ENSP00000474412 9606.ENSP00000401399 0 0 0 0 0.062 0 0 0.666 0.673

CCL5 LTA 9606.ENSP00000474412 9606.ENSP00000403495 0 0 0 0 0.109 0 0 0.687 0.71

CCL5 IL10 9606.ENSP00000474412 9606.ENSP00000412237 0 0 0 0 0.098 0 0.9 0.876 0.987

CCL5 IDO1 9606.ENSP00000474412 9606.ENSP00000430950 0 0 0 0 0.108 0 0 0.502 0.537

CCL5 HIF1A 9606.ENSP00000474412 9606.ENSP00000437955 0 0 0 0 0 0 0 0.465 0.465

CCL5 ELANE 9606.ENSP00000474412 9606.ENSP00000466090 0 0 0 0 0.049 0 0 0.414 0.418

CCL5 TNFAIP3 9606.ENSP00000474412 9606.ENSP00000481570 0 0 0 0 0.109 0 0 0.371 0.416

CCL5 VEGFA 9606.ENSP00000474412 9606.ENSP00000478570 0 0 0 0 0 0 0 0.755 0.755

CCR7 IL2 9606.ENSP00000246657 9606.ENSP00000226730 0 0 0 0 0 0 0 0.773 0.773

CCR7 IFNG 9606.ENSP00000246657 9606.ENSP00000229135 0 0 0 0 0.108 0 0 0.734 0.752

CCR7 IL4 9606.ENSP00000246657 9606.ENSP00000231449 0 0 0 0 0 0 0 0.719 0.719

CCR7 KIT 9606.ENSP00000246657 9606.ENSP00000288135 0 0 0 0 0 0 0 0.407 0.407

CCR7 LEF1 9606.ENSP00000246657 9606.ENSP00000265165 0 0 0 0 0.114 0 0 0.39 0.436

CCR7 VEGFA 9606.ENSP00000246657 9606.ENSP00000478570 0 0 0 0 0 0 0 0.46 0.459

CCR7 IL1A 9606.ENSP00000246657 9606.ENSP00000263339 0 0 0 0 0 0 0 0.46 0.459

CCR7 THBD 9606.ENSP00000246657 9606.ENSP00000366307 0 0 0 0 0 0 0 0.465 0.465

CCR7 IDO1 9606.ENSP00000246657 9606.ENSP00000430950 0 0 0 0 0.069 0 0 0.459 0.474

CCR7 MYD88 9606.ENSP00000246657 9606.ENSP00000401399 0 0 0 0 0.076 0 0 0.46 0.479

CCR7 MMP9 9606.ENSP00000246657 9606.ENSP00000361405 0 0 0 0 0.097 0 0 0.45 0.482

CCR7 STAT1 9606.ENSP00000246657 9606.ENSP00000354394 0 0 0 0 0.074 0 0 0.465 0.483

CCR7 FASLG 9606.ENSP00000246657 9606.ENSP00000356694 0 0 0 0 0.069 0 0 0.47 0.485

CCR7 TLR3 9606.ENSP00000246657 9606.ENSP00000296795 0 0 0 0 0 0 0 0.508 0.508

CCR7 STAT5A 9606.ENSP00000246657 9606.ENSP00000341208 0 0 0 0 0.118 0 0 0.471 0.513

CCR7 LTA 9606.ENSP00000246657 9606.ENSP00000403495 0 0 0 0 0.23 0 0 0.403 0.521

CCR7 ITGB2 9606.ENSP00000246657 9606.ENSP00000380948 0 0 0 0 0.267 0 0 0.391 0.534

CCR7 VCAM1 9606.ENSP00000246657 9606.ENSP00000294728 0 0 0 0 0.053 0 0 0.552 0.557

CCR7 SELE 9606.ENSP00000246657 9606.ENSP00000331736 0 0 0 0 0 0 0 0.556 0.557

CCR7 TLR4 9606.ENSP00000246657 9606.ENSP00000363089 0 0 0 0 0.069 0 0 0.564 0.576

CCR7 SELP 9606.ENSP00000246657 9606.ENSP00000263686 0 0 0 0 0.088 0 0 0.579 0.6

CCR7 IL13 9606.ENSP00000246657 9606.ENSP00000304915 0 0 0 0 0.06 0 0 0.601 0.608

CCR7 ITGAL 9606.ENSP00000246657 9606.ENSP00000349252 0 0 0 0 0.173 0 0 0.601 0.656

CCR7 ICAM1 9606.ENSP00000246657 9606.ENSP00000264832 0 0 0 0 0.076 0 0 0.648 0.661

CCR7 IL1B 9606.ENSP00000246657 9606.ENSP00000263341 0 0 0 0 0.113 0 0 0.652 0.678

CCR7 IL17A 9606.ENSP00000246657 9606.ENSP00000344192 0 0 0 0 0 0 0 0.689 0.689

CCR7 CSF2 9606.ENSP00000246657 9606.ENSP00000296871 0 0 0 0 0 0 0 0.695 0.695

CCR7 IL6 9606.ENSP00000246657 9606.ENSP00000385675 0 0 0 0 0.071 0 0 0.686 0.696

CCR7 CD40LG 9606.ENSP00000246657 9606.ENSP00000359663 0 0 0 0 0.13 0 0 0.69 0.719

CCR7 PF4 9606.ENSP00000246657 9606.ENSP00000296029 0 0 0 0 0.09 0 0.6 0.325 0.733

CCR7 IL10 9606.ENSP00000246657 9606.ENSP00000412237 0 0 0 0 0.065 0 0 0.743 0.75

CCR7 FOXP3 9606.ENSP00000246657 9606.ENSP00000365380 0 0 0 0 0.096 0 0 0.758 0.772

CCR7 CD40 9606.ENSP00000246657 9606.ENSP00000361359 0 0 0 0 0.107 0 0 0.766 0.782

CCR7 TNF 9606.ENSP00000246657 9606.ENSP00000398698 0 0 0 0 0.194 0 0 0.746 0.787

CCR7 FAS 9606.ENSP00000246657 9606.ENSP00000347979 0 0 0 0 0.088 0 0 0.82 0.829

CCR7 CXCL8 9606.ENSP00000246657 9606.ENSP00000306512 0 0 0 0 0.074 0 0.6 0.602 0.839

CCR7 CD28 9606.ENSP00000246657 9606.ENSP00000324890 0 0 0 0 0.107 0 0 0.855 0.865

CCR7 CXCL10 9606.ENSP00000246657 9606.ENSP00000305651 0 0 0 0 0.11 0 0.6 0.681 0.876

CCR7 CXCL13 9606.ENSP00000246657 9606.ENSP00000286758 0 0 0 0 0.121 0 0.6 0.809 0.927

CD28 TNFRSF1A 9606.ENSP00000324890 9606.ENSP00000162749 0 0 0 0 0.062 0 0 0.561 0.57

CD28 NFKBIA 9606.ENSP00000324890 9606.ENSP00000216797 0 0 0 0 0 0 0 0.504 0.504

CD28 TGFB1 9606.ENSP00000324890 9606.ENSP00000221930 0 0 0 0 0.062 0 0 0.525 0.536

CD28 IL2 9606.ENSP00000324890 9606.ENSP00000226730 0 0 0 0 0.063 0 0 0.912 0.914

CD28 IFNG 9606.ENSP00000324890 9606.ENSP00000229135 0 0 0 0 0.062 0 0 0.851 0.854

CD28 IL4 9606.ENSP00000324890 9606.ENSP00000231449 0 0 0 0 0.065 0 0 0.863 0.866

CD28 CRP 9606.ENSP00000324890 9606.ENSP00000255030 0 0 0 0 0 0 0 0.455 0.455

CD28 IL1A 9606.ENSP00000324890 9606.ENSP00000263339 0 0 0 0 0 0 0 0.455 0.455

CD28 IL1B 9606.ENSP00000324890 9606.ENSP00000263341 0 0 0 0 0 0 0 0.686 0.686

CD28 SELP 9606.ENSP00000324890 9606.ENSP00000263686 0 0 0 0 0.062 0 0 0.39 0.403

CD28 ICAM1 9606.ENSP00000324890 9606.ENSP00000264832 0 0 0 0 0.062 0 0 0.683 0.69

CD28 TP53 9606.ENSP00000324890 9606.ENSP00000269305 0 0 0 0 0 0 0 0.442 0.442

CD28 ERBB2 9606.ENSP00000324890 9606.ENSP00000269571 0 0 0 0 0 0 0 0.502 0.502

CD28 KIT 9606.ENSP00000324890 9606.ENSP00000288135 0 0 0 0 0 0 0 0.455 0.455

CD28 VCAM1 9606.ENSP00000324890 9606.ENSP00000294728 0 0 0 0 0.065 0 0 0.421 0.435

CD28 TLR3 9606.ENSP00000324890 9606.ENSP00000296795 0 0 0 0 0.062 0 0 0.459 0.47

CD28 CSF2 9606.ENSP00000324890 9606.ENSP00000296871 0 0 0 0 0 0 0 0.72 0.72

CD28 HPRT1 9606.ENSP00000324890 9606.ENSP00000298556 0 0 0 0 0 0 0 0.42 0.42

CD28 IL13 9606.ENSP00000324890 9606.ENSP00000304915 0 0 0 0 0 0 0 0.7 0.7

CD28 CXCL10 9606.ENSP00000324890 9606.ENSP00000305651 0 0 0 0 0 0 0 0.523 0.523

CD28 CXCL8 9606.ENSP00000324890 9606.ENSP00000306512 0 0 0 0 0 0 0 0.568 0.568

CD28 TNFRSF1B 9606.ENSP00000324890 9606.ENSP00000365435 0 0 0 0 0.083 0 0 0.392 0.419

CD28 VEGFA 9606.ENSP00000324890 9606.ENSP00000478570 0 0 0 0 0 0 0 0.426 0.426

CD28 INS 9606.ENSP00000324890 9606.ENSP00000380432 0 0 0 0 0 0 0 0.428 0.428

CD28 CX3CR1 9606.ENSP00000324890 9606.ENSP00000351059 0 0 0 0 0.088 0 0 0.42 0.448

CD28 MYD88 9606.ENSP00000324890 9606.ENSP00000401399 0 0 0 0 0.062 0 0 0.475 0.487

CD28 LTA 9606.ENSP00000324890 9606.ENSP00000403495 0 0 0 0 0.079 0 0 0.484 0.505

CD28 JUN 9606.ENSP00000324890 9606.ENSP00000360266 0 0 0 0 0 0 0 0.511 0.511

CD28 MTOR 9606.ENSP00000324890 9606.ENSP00000354558 0 0 0 0 0 0 0 0.517 0.517

CD28 IDO1 9606.ENSP00000324890 9606.ENSP00000430950 0 0 0 0 0 0 0 0.538 0.538

CD28 TLR4 9606.ENSP00000324890 9606.ENSP00000363089 0 0 0 0 0.066 0 0 0.563 0.575

CD28 STAT1 9606.ENSP00000324890 9606.ENSP00000354394 0 0 0 0 0.062 0 0 0.568 0.578

CD28 CXCR4 9606.ENSP00000324890 9606.ENSP00000386884 0 0 0 0 0.099 0 0 0.572 0.598

CD28 FASLG 9606.ENSP00000324890 9606.ENSP00000356694 0 0 0 0 0 0 0 0.617 0.617

CD28 STAT5A 9606.ENSP00000324890 9606.ENSP00000341208 0 0 0 0 0.076 0 0 0.622 0.636

CD28 IL6 9606.ENSP00000324890 9606.ENSP00000385675 0 0 0 0 0 0 0 0.792 0.792

CD28 TNF 9606.ENSP00000324890 9606.ENSP00000398698 0 0 0 0 0.062 0 0 0.833 0.837

CD28 IL17A 9606.ENSP00000324890 9606.ENSP00000344192 0 0 0 0 0 0 0 0.847 0.847

CD28 IL10 9606.ENSP00000324890 9606.ENSP00000412237 0 0 0 0 0.062 0 0 0.844 0.847

CD28 FAS 9606.ENSP00000324890 9606.ENSP00000347979 0 0 0 0 0.062 0 0 0.853 0.856

CD28 FOXP3 9606.ENSP00000324890 9606.ENSP00000365380 0 0 0 0 0 0.063 0 0.878 0.881

CD28 CD40LG 9606.ENSP00000324890 9606.ENSP00000359663 0 0 0 0 0.129 0 0 0.889 0.899

CD28 ITGB2 9606.ENSP00000324890 9606.ENSP00000380948 0 0 0 0 0.118 0 0.9 0.391 0.941

CD28 CD40 9606.ENSP00000324890 9606.ENSP00000361359 0 0 0 0 0.062 0 0 0.95 0.951

CD28 ITGAL 9606.ENSP00000324890 9606.ENSP00000349252 0 0 0 0 0.15 0 0.9 0.635 0.966

CD40 TNFRSF1A 9606.ENSP00000361359 9606.ENSP00000162749 0 0 0 0 0.062 0 0 0.852 0.855

CD40 NFKBIA 9606.ENSP00000361359 9606.ENSP00000216797 0 0 0 0 0.083 0 0.9 0.761 0.976

CD40 TGFB1 9606.ENSP00000361359 9606.ENSP00000221930 0 0 0 0 0.062 0 0 0.429 0.441

CD40 NFKB1 9606.ENSP00000361359 9606.ENSP00000226574 0 0 0 0 0.064 0 0.9 0.465 0.945

CD40 IL2 9606.ENSP00000361359 9606.ENSP00000226730 0 0 0 0 0 0 0 0.802 0.802

CD40 IFNG 9606.ENSP00000361359 9606.ENSP00000229135 0 0 0 0 0 0 0 0.804 0.804

CD40 IL4 9606.ENSP00000361359 9606.ENSP00000231449 0 0 0 0 0 0 0 0.969 0.969

CD40 IRF1 9606.ENSP00000361359 9606.ENSP00000245414 0 0 0 0 0.125 0 0 0.451 0.499

CD40 CRP 9606.ENSP00000361359 9606.ENSP00000255030 0 0 0 0 0 0 0 0.478 0.478

CD40 LYZ 9606.ENSP00000361359 9606.ENSP00000261267 0 0 0 0 0.062 0 0 0.46 0.472

CD40 IL1A 9606.ENSP00000361359 9606.ENSP00000263339 0 0 0 0 0.156 0 0 0.594 0.643

CD40 IL1B 9606.ENSP00000361359 9606.ENSP00000263341 0 0 0 0 0.164 0 0 0.758 0.789

CD40 SELP 9606.ENSP00000361359 9606.ENSP00000263686 0 0 0 0 0.085 0 0 0.566 0.586

CD40 ICAM1 9606.ENSP00000361359 9606.ENSP00000264832 0 0 0 0 0.107 0 0 0.763 0.779

CD40 TP53 9606.ENSP00000361359 9606.ENSP00000269305 0 0 0 0 0.062 0 0 0.464 0.475

CD40 ERBB2 9606.ENSP00000361359 9606.ENSP00000269571 0 0 0 0 0 0 0 0.413 0.412

CD40 CXCL13 9606.ENSP00000361359 9606.ENSP00000286758 0 0 0 0 0.062 0 0 0.528 0.538

CD40 PPARG 9606.ENSP00000361359 9606.ENSP00000287820 0 0 0 0 0.062 0 0 0.391 0.404

CD40 KIT 9606.ENSP00000361359 9606.ENSP00000288135 0 0 0 0 0 0 0 0.454 0.454

CD40 VCAM1 9606.ENSP00000361359 9606.ENSP00000294728 0 0 0 0 0.084 0 0 0.617 0.634

CD40 TLR3 9606.ENSP00000361359 9606.ENSP00000296795 0 0 0 0 0.062 0 0 0.84 0.844

CD40 CSF2 9606.ENSP00000361359 9606.ENSP00000296871 0 0 0 0 0.084 0 0 0.841 0.848

CD40 NOD2 9606.ENSP00000361359 9606.ENSP00000300589 0 0 0 0 0.098 0 0 0.476 0.507

CD40 IL13 9606.ENSP00000361359 9606.ENSP00000304915 0 0 0 0 0 0 0 0.681 0.681

CD40 CXCL10 9606.ENSP00000361359 9606.ENSP00000305651 0 0 0 0 0.121 0 0 0.642 0.672

CD40 CXCL8 9606.ENSP00000361359 9606.ENSP00000306512 0 0 0 0 0.089 0 0 0.689 0.705

CD40 IL17RA 9606.ENSP00000361359 9606.ENSP00000320936 0 0 0 0 0 0 0 0.401 0.401

CD40 NOS2 9606.ENSP00000361359 9606.ENSP00000327251 0 0 0 0 0.062 0 0 0.467 0.479

CD40 SOCS1 9606.ENSP00000361359 9606.ENSP00000329418 0 0 0 0 0.085 0 0 0.456 0.481

CD40 SELE 9606.ENSP00000361359 9606.ENSP00000331736 0 0 0 0 0.062 0 0 0.5 0.51

CD40 STAT5A 9606.ENSP00000361359 9606.ENSP00000341208 0 0 0 0 0.089 0 0.9 0.464 0.946

CD40 IL17A 9606.ENSP00000361359 9606.ENSP00000344192 0 0 0 0 0 0 0 0.729 0.729

CD40 FAS 9606.ENSP00000361359 9606.ENSP00000347979 0 0 0 0 0.081 0 0 0.841 0.848

CD40 ITGAL 9606.ENSP00000361359 9606.ENSP00000349252 0 0 0 0 0.085 0 0 0.586 0.605

CD40 PAX5 9606.ENSP00000361359 9606.ENSP00000350844 0 0 0 0 0.062 0 0 0.462 0.474

CD40 CX3CR1 9606.ENSP00000361359 9606.ENSP00000351059 0 0 0 0 0 0 0 0.519 0.519

CD40 STAT1 9606.ENSP00000361359 9606.ENSP00000354394 0 0 0 0 0.064 0 0 0.616 0.625

CD40 FASLG 9606.ENSP00000361359 9606.ENSP00000356694 0 0 0 0 0 0 0 0.616 0.616

CD40 CHUK 9606.ENSP00000361359 9606.ENSP00000359424 0 0 0 0 0.063 0.519 0.54 0.609 0.908

CD40 CD40LG 9606.ENSP00000361359 9606.ENSP00000359663 0 0 0 0 0.062 0.932 0.9 0.995 0.999

CD40 JUN 9606.ENSP00000361359 9606.ENSP00000360266 0 0 0 0 0.054 0 0 0.556 0.563

CD40 ITGB2 9606.ENSP00000361359 9606.ENSP00000380948 0 0 0 0 0.1 0 0 0.394 0.431

CD40 INS 9606.ENSP00000361359 9606.ENSP00000380432 0 0 0 0 0 0 0 0.466 0.465

CD40 HDAC1 9606.ENSP00000361359 9606.ENSP00000362649 0 0 0 0 0 0 0 0.48 0.48

CD40 MMP9 9606.ENSP00000361359 9606.ENSP00000361405 0 0 0 0 0.093 0 0 0.473 0.502

CD40 VEGFA 9606.ENSP00000361359 9606.ENSP00000478570 0 0 0 0 0 0 0 0.56 0.56

CD40 THBD 9606.ENSP00000361359 9606.ENSP00000366307 0 0 0 0 0.062 0 0 0.559 0.568

CD40 IDO1 9606.ENSP00000361359 9606.ENSP00000430950 0 0 0 0 0.096 0 0 0.563 0.588

CD40 CXCR4 9606.ENSP00000361359 9606.ENSP00000386884 0 0 0 0 0.062 0 0 0.597 0.606

CD40 TNFAIP3 9606.ENSP00000361359 9606.ENSP00000481570 0 0 0 0 0.108 0.132 0 0.556 0.626

CD40 TNFRSF1B 9606.ENSP00000361359 9606.ENSP00000365435 0 0 0 0.714 0.101 0 0.6 0.518 0.678

CD40 FOXP3 9606.ENSP00000361359 9606.ENSP00000365380 0 0 0 0 0 0 0 0.773 0.773

CD40 MYD88 9606.ENSP00000361359 9606.ENSP00000401399 0 0 0 0 0.062 0 0 0.798 0.803

CD40 TLR4 9606.ENSP00000361359 9606.ENSP00000363089 0 0 0 0 0.085 0 0 0.832 0.84

CD40 IL6 9606.ENSP00000361359 9606.ENSP00000385675 0 0 0 0 0.11 0 0 0.834 0.846

CD40 LTA 9606.ENSP00000361359 9606.ENSP00000403495 0 0 0 0 0.147 0 0.6 0.658 0.873

CD40 IL10 9606.ENSP00000361359 9606.ENSP00000412237 0 0 0 0 0.062 0 0 0.885 0.888

CD40 TNF 9606.ENSP00000361359 9606.ENSP00000398698 0 0 0 0 0.161 0 0.6 0.864 0.95

CD40LG TNFRSF1A 9606.ENSP00000359663 9606.ENSP00000162749 0 0 0 0 0 0 0 0.824 0.824

CD40LG NFKBIA 9606.ENSP00000359663 9606.ENSP00000216797 0 0 0 0 0 0.062 0.9 0.506 0.949

CD40LG MPO 9606.ENSP00000359663 9606.ENSP00000225275 0 0 0 0 0 0.056 0 0.506 0.513

CD40LG NFKB1 9606.ENSP00000359663 9606.ENSP00000226574 0 0 0 0 0 0.062 0.9 0.366 0.935

CD40LG IL2 9606.ENSP00000359663 9606.ENSP00000226730 0 0 0 0 0.076 0 0 0.806 0.813

CD40LG IFNG 9606.ENSP00000359663 9606.ENSP00000229135 0 0 0 0 0.117 0 0 0.863 0.874

CD40LG IL4 9606.ENSP00000359663 9606.ENSP00000231449 0 0 0 0 0 0 0 0.934 0.934

CD40LG CRP 9606.ENSP00000359663 9606.ENSP00000255030 0 0 0 0 0 0 0 0.661 0.661

CD40LG IL1RN 9606.ENSP00000359663 9606.ENSP00000259206 0 0 0 0 0.064 0 0 0.391 0.405

CD40LG IL1A 9606.ENSP00000359663 9606.ENSP00000263339 0 0 0 0 0 0 0 0.649 0.649

CD40LG IL1B 9606.ENSP00000359663 9606.ENSP00000263341 0 0 0 0 0 0 0 0.692 0.692

CD40LG SELP 9606.ENSP00000359663 9606.ENSP00000263686 0 0 0 0 0.139 0 0 0.759 0.783

CD40LG ICAM1 9606.ENSP00000359663 9606.ENSP00000264832 0 0 0 0 0 0 0 0.709 0.709

CD40LG EGF 9606.ENSP00000359663 9606.ENSP00000265171 0 0 0 0 0 0.056 0 0.402 0.411

CD40LG TP53 9606.ENSP00000359663 9606.ENSP00000269305 0 0 0 0 0 0.213 0 0.662 0.722

CD40LG CXCL13 9606.ENSP00000359663 9606.ENSP00000286758 0 0 0 0 0 0 0 0.603 0.603

CD40LG VCAM1 9606.ENSP00000359663 9606.ENSP00000294728 0 0 0 0 0.085 0 0 0.612 0.629

CD40LG PF4 9606.ENSP00000359663 9606.ENSP00000296029 0 0 0 0 0.088 0 0 0.605 0.624

CD40LG TLR3 9606.ENSP00000359663 9606.ENSP00000296795 0 0 0 0 0.062 0 0 0.563 0.572

CD40LG CSF2 9606.ENSP00000359663 9606.ENSP00000296871 0 0 0 0 0 0 0 0.748 0.748

CD40LG IL13 9606.ENSP00000359663 9606.ENSP00000304915 0 0 0 0 0.062 0 0 0.692 0.699

CD40LG PRKCB 9606.ENSP00000359663 9606.ENSP00000305355 0 0 0 0 0.064 0.056 0 0.451 0.473

CD40LG CXCL10 9606.ENSP00000359663 9606.ENSP00000305651 0 0 0 0 0 0 0 0.605 0.605

CD40LG CXCL8 9606.ENSP00000359663 9606.ENSP00000306512 0 0 0 0 0.062 0 0 0.681 0.688

CD40LG F2 9606.ENSP00000359663 9606.ENSP00000308541 0 0 0 0 0 0.097 0 0.411 0.446

CD40LG SELE 9606.ENSP00000359663 9606.ENSP00000331736 0 0 0 0 0.056 0 0 0.56 0.566

CD40LG STAT5A 9606.ENSP00000359663 9606.ENSP00000341208 0 0 0 0 0 0.056 0.9 0.454 0.943

CD40LG IL17A 9606.ENSP00000359663 9606.ENSP00000344192 0 0 0 0 0.062 0 0 0.76 0.766

CD40LG FAS 9606.ENSP00000359663 9606.ENSP00000347979 0 0 0 0 0 0 0 0.646 0.646

CD40LG ITGAL 9606.ENSP00000359663 9606.ENSP00000349252 0 0 0 0 0.123 0 0 0.563 0.6

CD40LG PAX5 9606.ENSP00000359663 9606.ENSP00000350844 0 0 0 0 0.089 0.056 0 0.417 0.455

CD40LG CX3CR1 9606.ENSP00000359663 9606.ENSP00000351059 0 0 0 0 0.076 0 0 0.391 0.413

CD40LG STAT1 9606.ENSP00000359663 9606.ENSP00000354394 0 0 0 0 0 0.056 0 0.505 0.512

CD40LG FASLG 9606.ENSP00000359663 9606.ENSP00000356694 0 0 0 0 0.105 0 0 0.662 0.684

CD40LG CHUK 9606.ENSP00000359663 9606.ENSP00000359424 0 0 0 0 0 0.056 0 0.503 0.51

CD40LG JUN 9606.ENSP00000359663 9606.ENSP00000360266 0 0 0 0 0 0 0 0.422 0.422

CD40LG IDO1 9606.ENSP00000359663 9606.ENSP00000430950 0 0 0 0 0 0 0 0.457 0.457

CD40LG INS 9606.ENSP00000359663 9606.ENSP00000380432 0 0 0 0 0 0 0 0.468 0.468

CD40LG MMP9 9606.ENSP00000359663 9606.ENSP00000361405 0 0 0 0 0 0.057 0 0.506 0.514

CD40LG THBD 9606.ENSP00000359663 9606.ENSP00000366307 0 0 0 0 0 0 0 0.516 0.516

CD40LG VEGFA 9606.ENSP00000359663 9606.ENSP00000478570 0 0 0 0 0 0 0 0.558 0.558

CD40LG MYD88 9606.ENSP00000359663 9606.ENSP00000401399 0 0 0 0 0 0 0 0.56 0.56

CD40LG CXCR4 9606.ENSP00000359663 9606.ENSP00000386884 0 0 0 0 0.068 0 0 0.558 0.57

CD40LG FOXP3 9606.ENSP00000359663 9606.ENSP00000365380 0 0 0 0 0.1 0.057 0 0.726 0.747

CD40LG IL6 9606.ENSP00000359663 9606.ENSP00000385675 0 0 0 0 0 0 0 0.761 0.761

CD40LG TLR4 9606.ENSP00000359663 9606.ENSP00000363089 0 0 0 0 0.062 0 0 0.773 0.778

CD40LG TNFRSF1B 9606.ENSP00000359663 9606.ENSP00000365435 0 0 0 0 0.088 0 0.6 0.459 0.785

CD40LG LTA 9606.ENSP00000359663 9606.ENSP00000403495 0 0 0 0 0.098 0 0.6 0.576 0.833

CD40LG IL10 9606.ENSP00000359663 9606.ENSP00000412237 0 0 0 0 0 0 0 0.862 0.863

CD40LG TNF 9606.ENSP00000359663 9606.ENSP00000398698 0 0 0 0 0.076 0 0.6 0.822 0.928

CD40LG ITGB2 9606.ENSP00000359663 9606.ENSP00000380948 0 0 0 0 0.122 0 0.9 0.707 0.972

CDKN1A HMOX1 9606.ENSP00000384849 9606.ENSP00000216117 0 0 0 0 0.076 0 0 0.419 0.44

CDKN1A NFKBIA 9606.ENSP00000384849 9606.ENSP00000216797 0 0 0 0 0.097 0 0 0.458 0.489

CDKN1A MMP2 9606.ENSP00000384849 9606.ENSP00000219070 0 0 0 0 0.061 0 0 0.422 0.434

CDKN1A TGFB1 9606.ENSP00000384849 9606.ENSP00000221930 0 0 0 0 0.077 0 0 0.44 0.461

CDKN1A MDM2 9606.ENSP00000384849 9606.ENSP00000258149 0 0 0 0 0.062 0.872 0 0.823 0.976

CDKN1A SMAD7 9606.ENSP00000384849 9606.ENSP00000262158 0 0 0 0 0.065 0 0 0.433 0.447

CDKN1A IL1B 9606.ENSP00000384849 9606.ENSP00000263341 0 0 0 0 0.069 0 0 0.39 0.407

CDKN1A FGF2 9606.ENSP00000384849 9606.ENSP00000264498 0 0 0 0 0 0 0 0.452 0.452

CDKN1A EGF 9606.ENSP00000384849 9606.ENSP00000265171 0 0 0 0 0 0 0 0.559 0.559

CDKN1A TP53 9606.ENSP00000384849 9606.ENSP00000269305 0 0 0 0 0 0.968 0.9 0.903 0.999

CDKN1A ERBB2 9606.ENSP00000384849 9606.ENSP00000269571 0 0 0 0 0 0 0 0.559 0.559

CDKN1A EGFR 9606.ENSP00000384849 9606.ENSP00000275493 0 0 0 0 0.064 0 0 0.569 0.579

CDKN1A PPARG 9606.ENSP00000384849 9606.ENSP00000287820 0 0 0 0 0 0 0 0.49 0.49

CDKN1A IGF1 9606.ENSP00000384849 9606.ENSP00000302665 0 0 0 0 0.051 0 0 0.504 0.509

CDKN1A CXCL8 9606.ENSP00000384849 9606.ENSP00000306512 0 0 0 0 0.106 0 0 0.426 0.465

CDKN1A CYCS 9606.ENSP00000384849 9606.ENSP00000307786 0 0 0 0 0 0 0 0.6 0.6

CDKN1A NOS2 9606.ENSP00000384849 9606.ENSP00000327251 0 0 0 0 0 0.105 0.9 0.15 0.917

CDKN1A STAT5A 9606.ENSP00000384849 9606.ENSP00000341208 0 0 0 0 0 0 0.9 0.39 0.936

CDKN1A E2F1 9606.ENSP00000384849 9606.ENSP00000345571 0 0 0 0 0.052 0.273 0.9 0.672 0.974

CDKN1A FAS 9606.ENSP00000384849 9606.ENSP00000347979 0 0 0 0 0.101 0 0 0.465 0.498

CDKN1A DNMT1 9606.ENSP00000384849 9606.ENSP00000352516 0 0 0 0 0 0 0 0.504 0.504

CDKN1A STAT1 9606.ENSP00000384849 9606.ENSP00000354394 0 0 0 0 0.058 0 0.9 0.526 0.951

CDKN1A TOP1 9606.ENSP00000384849 9606.ENSP00000354522 0 0 0 0 0 0.105 0 0.4 0.44

CDKN1A MTOR 9606.ENSP00000384849 9606.ENSP00000354558 0 0 0 0 0 0 0 0.521 0.521

CDKN1A PARP1 9606.ENSP00000384849 9606.ENSP00000355759 0 0 0 0 0.049 0.675 0 0.489 0.828

CDKN1A FASLG 9606.ENSP00000384849 9606.ENSP00000356694 0 0 0 0 0 0 0 0.414 0.414

CDKN1A JUN 9606.ENSP00000384849 9606.ENSP00000360266 0 0 0 0 0.079 0.27 0.9 0.616 0.97

CDKN1A PTEN 9606.ENSP00000384849 9606.ENSP00000361021 0 0 0 0 0 0 0 0.665 0.665

CDKN1A MMP9 9606.ENSP00000384849 9606.ENSP00000361405 0 0 0 0 0 0 0 0.468 0.468

CDKN1A HDAC1 9606.ENSP00000384849 9606.ENSP00000362649 0 0 0 0 0 0.731 0 0.633 0.897

CDKN1A PCNA 9606.ENSP00000384849 9606.ENSP00000368458 0 0 0 0 0.063 0.998 0.9 0.265 0.999

CDKN1A INS 9606.ENSP00000384849 9606.ENSP00000380432 0 0 0 0 0 0 0 0.508 0.508

CDKN1A VDR 9606.ENSP00000384849 9606.ENSP00000447173 0 0 0 0 0.065 0.27 0 0.349 0.516

CDKN1A IL6 9606.ENSP00000384849 9606.ENSP00000385675 0 0 0 0 0.076 0 0 0.506 0.524

CDKN1A TNF 9606.ENSP00000384849 9606.ENSP00000398698 0 0 0 0 0.062 0 0 0.552 0.562

CDKN1A VEGFA 9606.ENSP00000384849 9606.ENSP00000478570 0 0 0 0 0.069 0 0 0.556 0.568

CDKN1A ESR1 9606.ENSP00000384849 9606.ENSP00000405330 0 0 0 0 0 0.675 0 0.597 0.863

CDKN1A HIF1A 9606.ENSP00000384849 9606.ENSP00000437955 0 0 0 0 0 0 0.9 0.522 0.95

CETP IL6 9606.ENSP00000200676 9606.ENSP00000385675 0 0 0 0 0.062 0 0 0.391 0.404

CETP VCAM1 9606.ENSP00000200676 9606.ENSP00000294728 0 0 0 0 0.083 0 0 0.401 0.428

CETP SREBF1 9606.ENSP00000200676 9606.ENSP00000348069 0 0 0 0 0 0 0 0.451 0.451

CETP PPARG 9606.ENSP00000200676 9606.ENSP00000287820 0 0 0 0 0 0 0 0.456 0.456

CETP CRP 9606.ENSP00000200676 9606.ENSP00000255030 0 0 0 0 0.065 0 0 0.567 0.578

CETP INS 9606.ENSP00000200676 9606.ENSP00000380432 0 0 0 0 0 0 0 0.603 0.603

CETP MTTP 9606.ENSP00000200676 9606.ENSP00000427679 0 0 0 0 0 0 0 0.611 0.611

CETP HMGCR 9606.ENSP00000200676 9606.ENSP00000287936 0 0 0 0 0 0 0 0.635 0.635

CETP PON1 9606.ENSP00000200676 9606.ENSP00000222381 0 0 0 0 0 0 0.72 0.738 0.923

CETP LPL 9606.ENSP00000200676 9606.ENSP00000309757 0 0 0 0 0.065 0.213 0.54 0.802 0.924

CETP NR1H2 9606.ENSP00000200676 9606.ENSP00000253727 0 0 0 0 0 0 0.9 0.332 0.93

CFTR OCLN 9606.ENSP00000003084 9606.ENSP00000347379 0 0 0 0 0.062 0 0 0.394 0.407

CFTR TP53 9606.ENSP00000003084 9606.ENSP00000269305 0 0 0 0 0 0.064 0 0.403 0.417

CFTR IL6 9606.ENSP00000003084 9606.ENSP00000385675 0 0 0 0 0 0 0 0.457 0.457

CFTR RYR1 9606.ENSP00000003084 9606.ENSP00000352608 0 0 0 0 0 0.248 0 0.319 0.466

CFTR IL4 9606.ENSP00000003084 9606.ENSP00000231449 0 0 0 0 0 0 0 0.481 0.481

CFTR IL1B 9606.ENSP00000003084 9606.ENSP00000263341 0 0 0 0 0 0 0 0.494 0.493

CFTR NR3C2 9606.ENSP00000003084 9606.ENSP00000350815 0 0 0 0 0 0.056 0 0.508 0.515

CFTR INS 9606.ENSP00000003084 9606.ENSP00000380432 0 0 0 0 0 0 0 0.517 0.517

CFTR TLR4 9606.ENSP00000003084 9606.ENSP00000363089 0 0 0 0 0 0 0 0.529 0.529

CFTR TNF 9606.ENSP00000003084 9606.ENSP00000398698 0 0 0 0 0 0 0 0.531 0.532

CFTR ELANE 9606.ENSP00000003084 9606.ENSP00000466090 0 0 0 0 0 0 0 0.55 0.55

CFTR HSPA5 9606.ENSP00000003084 9606.ENSP00000324173 0 0 0 0 0.062 0.406 0 0.323 0.591

CFTR PTEN 9606.ENSP00000003084 9606.ENSP00000361021 0 0 0 0 0.055 0.061 0 0.576 0.592

CFTR CXCL8 9606.ENSP00000003084 9606.ENSP00000306512 0 0 0 0 0.062 0 0 0.601 0.609

CFTR CLDN4 9606.ENSP00000003084 9606.ENSP00000409544 0 0 0 0 0.068 0 0.54 0.29 0.669

CFTR HSPB1 9606.ENSP00000003084 9606.ENSP00000248553 0 0 0 0 0 0.634 0 0.305 0.735

CFTR F2 9606.ENSP00000003084 9606.ENSP00000308541 0 0 0 0 0 0 0.9 0.166 0.913

CFTR EDN1 9606.ENSP00000003084 9606.ENSP00000368683 0 0 0 0 0 0 0.9 0.212 0.917

CFTR VCP 9606.ENSP00000003084 9606.ENSP00000351777 0 0 0 0 0.058 0.89 0.6 0.773 0.989

CHRM1 CHRM3 9606.ENSP00000306490 9606.ENSP00000255380 0 0 0 0.95 0.119 0 0.8 0.812 0.823

CHRM1 SST 9606.ENSP00000306490 9606.ENSP00000287641 0 0 0 0 0.171 0 0.6 0.167 0.699

CHRM1 GRIN2B 9606.ENSP00000306490 9606.ENSP00000477455 0 0 0 0 0.188 0 0 0.347 0.447

CHRM1 PLCB1 9606.ENSP00000306490 9606.ENSP00000338185 0 0 0 0 0.096 0 0 0.426 0.459

CHRM1 CHRM2 9606.ENSP00000306490 9606.ENSP00000399745 0 0 0 0.923 0.059 0 0.8 0.805 0.815

CHRM1 F2 9606.ENSP00000306490 9606.ENSP00000308541 0 0 0 0 0 0 0.9 0 0.9

CHRM1 JUN 9606.ENSP00000306490 9606.ENSP00000360266 0 0 0 0 0 0 0.9 0.062 0.902

CHRM1 CXCR4 9606.ENSP00000306490 9606.ENSP00000386884 0 0 0 0 0 0 0.9 0.11 0.907

CHRM1 EDN1 9606.ENSP00000306490 9606.ENSP00000368683 0 0 0 0 0 0 0.9 0.141 0.91

CHRM2 SLC6A4 9606.ENSP00000399745 9606.ENSP00000261707 0 0 0 0 0.062 0 0 0.471 0.483

CHRM2 SST 9606.ENSP00000399745 9606.ENSP00000287641 0 0 0 0 0.063 0 0.6 0.159 0.657

CHRM2 EDN1 9606.ENSP00000399745 9606.ENSP00000368683 0 0 0 0 0 0 0.6 0.083 0.617

CHRM3 IL6 9606.ENSP00000255380 9606.ENSP00000385675 0 0 0 0 0 0 0 0.411 0.411

CHRM3 PLCB1 9606.ENSP00000255380 9606.ENSP00000338185 0 0 0 0 0.085 0 0.65 0.268 0.745

CHUK TNFRSF1A 9606.ENSP00000359424 9606.ENSP00000162749 0 0 0 0 0.062 0.884 0.9 0.761 0.997

CHUK NFKBIA 9606.ENSP00000359424 9606.ENSP00000216797 0 0 0 0 0.049 0.994 0.9 0.935 0.999

CHUK NFKB1 9606.ENSP00000359424 9606.ENSP00000226574 0 0 0 0 0.062 0.965 0.9 0.815 0.999

CHUK IRF1 9606.ENSP00000359424 9606.ENSP00000245414 0 0 0 0 0.062 0.127 0 0.373 0.441

CHUK HSPB1 9606.ENSP00000359424 9606.ENSP00000248553 0 0 0 0 0 0.289 0 0.309 0.488

CHUK MDM2 9606.ENSP00000359424 9606.ENSP00000258149 0 0 0 0 0.063 0.056 0 0.379 0.402

CHUK IL1A 9606.ENSP00000359424 9606.ENSP00000263339 0 0 0 0 0.062 0 0.9 0.392 0.937

CHUK IL1B 9606.ENSP00000359424 9606.ENSP00000263341 0 0 0 0 0 0.056 0 0.772 0.776

CHUK ICAM1 9606.ENSP00000359424 9606.ENSP00000264832 0 0 0 0 0 0 0 0.469 0.469

CHUK EGF 9606.ENSP00000359424 9606.ENSP00000265171 0 0 0 0 0 0.056 0 0.403 0.412

CHUK TP53 9606.ENSP00000359424 9606.ENSP00000269305 0 0 0 0 0.054 0.47 0 0.793 0.887

CHUK EGFR 9606.ENSP00000359424 9606.ENSP00000275493 0 0 0 0 0 0.107 0 0.465 0.502

CHUK VCAM1 9606.ENSP00000359424 9606.ENSP00000294728 0 0 0 0 0.062 0 0 0.405 0.418

CHUK TLR3 9606.ENSP00000359424 9606.ENSP00000296795 0 0 0 0 0 0.089 0.6 0.611 0.845

CHUK NOD2 9606.ENSP00000359424 9606.ENSP00000300589 0 0 0 0 0 0 0.9 0.466 0.944

CHUK FADD 9606.ENSP00000359424 9606.ENSP00000301838 0 0 0 0 0 0.215 0.9 0.72 0.976

CHUK PRKCB 9606.ENSP00000359424 9606.ENSP00000305355 0 0 0 0.565 0 0.686 0.9 0.261 0.97

CHUK CXCL8 9606.ENSP00000359424 9606.ENSP00000306512 0 0 0 0 0 0 0 0.601 0.601

CHUK IL17A 9606.ENSP00000359424 9606.ENSP00000344192 0 0 0 0 0 0 0 0.418 0.418

CHUK FAS 9606.ENSP00000359424 9606.ENSP00000347979 0 0 0 0 0.053 0 0.9 0.293 0.927

CHUK STAT1 9606.ENSP00000359424 9606.ENSP00000354394 0 0 0 0 0 0.14 0 0.509 0.559

CHUK MTOR 9606.ENSP00000359424 9606.ENSP00000354558 0 0 0 0 0.062 0.519 0.8 0.59 0.958

CHUK FASLG 9606.ENSP00000359424 9606.ENSP00000356694 0 0 0 0 0 0 0.9 0.34 0.931

CHUK NGF 9606.ENSP00000359424 9606.ENSP00000358525 0 0 0 0 0 0.056 0.9 0.2 0.917

CHUK PRKCD 9606.ENSP00000359424 9606.ENSP00000378217 0 0 0 0.56 0.063 0.327 0 0.319 0.427

CHUK EDA 9606.ENSP00000359424 9606.ENSP00000363680 0 0 0 0 0 0.148 0 0.372 0.442

CHUK VEGFA 9606.ENSP00000359424 9606.ENSP00000478570 0 0 0 0 0.062 0 0 0.456 0.467

CHUK HIF1A 9606.ENSP00000359424 9606.ENSP00000437955 0 0 0 0 0.064 0.07 0 0.438 0.468

CHUK TNFRSF1B 9606.ENSP00000359424 9606.ENSP00000365435 0 0 0 0 0.062 0.282 0 0.322 0.503

CHUK MMP9 9606.ENSP00000359424 9606.ENSP00000361405 0 0 0 0 0 0 0 0.503 0.503

CHUK IL10 9606.ENSP00000359424 9606.ENSP00000412237 0 0 0 0 0 0.06 0 0.509 0.518

CHUK ESR1 9606.ENSP00000359424 9606.ENSP00000405330 0 0 0 0 0 0.3 0 0.517 0.647

CHUK IL6 9606.ENSP00000359424 9606.ENSP00000385675 0 0 0 0 0.049 0 0 0.686 0.689

CHUK JUN 9606.ENSP00000359424 9606.ENSP00000360266 0 0 0 0 0 0.057 0 0.735 0.74

CHUK PTEN 9606.ENSP00000359424 9606.ENSP00000361021 0 0 0 0 0.097 0.323 0.8 0.513 0.932

CHUK TNFAIP3 9606.ENSP00000359424 9606.ENSP00000481570 0 0 0 0 0 0.298 0.9 0.469 0.959

CHUK TLR4 9606.ENSP00000359424 9606.ENSP00000363089 0 0 0 0 0 0.089 0.9 0.66 0.966

CHUK TNF 9606.ENSP00000359424 9606.ENSP00000398698 0 0 0 0 0.062 0.68 0.9 0.777 0.992

CHUK MYD88 9606.ENSP00000359424 9606.ENSP00000401399 0 0 0 0 0.06 0.149 0.9 0.936 0.994

CLDN4 ERBB2 9606.ENSP00000409544 9606.ENSP00000269571 0 0 0 0 0.146 0 0 0.36 0.43

CLDN4 EGFR 9606.ENSP00000409544 9606.ENSP00000275493 0 0 0 0 0.091 0 0 0.396 0.427

CLDN4 OCLN 9606.ENSP00000409544 9606.ENSP00000347379 0 0 0 0 0.107 0 0.6 0.915 0.967

CLDN4 SHBG 9606.ENSP00000409544 9606.ENSP00000369816 0 0 0 0 0 0.192 0 0.429 0.519

CLN3 CTSD 9606.ENSP00000454229 9606.ENSP00000236671 0 0 0 0 0.107 0.083 0 0.702 0.735

COL1A1 PLOD1 9606.ENSP00000225964 9606.ENSP00000196061 0 0 0 0 0.13 0.223 0.6 0.496 0.845

COL1A1 MMP2 9606.ENSP00000225964 9606.ENSP00000219070 0 0 0 0 0.879 0.355 0 0.612 0.967

COL1A1 TGFB1 9606.ENSP00000225964 9606.ENSP00000221930 0 0 0 0 0.062 0.182 0 0.664 0.72

COL1A1 FGF23 9606.ENSP00000225964 9606.ENSP00000237837 0 0 0 0 0 0.056 0 0.397 0.407

COL1A1 VCAM1 9606.ENSP00000225964 9606.ENSP00000294728 0 0 0 0 0.112 0 0 0.379 0.424

COL1A1 ESR1 9606.ENSP00000225964 9606.ENSP00000405330 0 0 0 0 0 0.056 0 0.421 0.43

COL1A1 HIF1A 9606.ENSP00000225964 9606.ENSP00000437955 0 0 0 0 0 0 0 0.438 0.438

COL1A1 JUN 9606.ENSP00000225964 9606.ENSP00000360266 0 0 0 0 0 0 0 0.442 0.442

COL1A1 TP53 9606.ENSP00000225964 9606.ENSP00000269305 0 0 0 0 0 0 0 0.452 0.452

COL1A1 HPRT1 9606.ENSP00000225964 9606.ENSP00000298556 0 0 0 0 0 0 0 0.454 0.454

COL1A1 GPT 9606.ENSP00000225964 9606.ENSP00000378408 0 0 0 0 0.062 0 0 0.458 0.469

COL1A1 EGFR 9606.ENSP00000225964 9606.ENSP00000275493 0 0 0 0 0.173 0.103 0 0.369 0.491

COL1A1 INS 9606.ENSP00000225964 9606.ENSP00000380432 0 0 0 0 0 0 0 0.501 0.501

COL1A1 GDF5 9606.ENSP00000225964 9606.ENSP00000363492 0 0 0 0 0.089 0.08 0 0.46 0.508

COL1A1 IL1B 9606.ENSP00000225964 9606.ENSP00000263341 0 0 0 0 0 0 0 0.522 0.522

COL1A1 VEGFA 9606.ENSP00000225964 9606.ENSP00000478570 0 0 0 0 0.076 0 0 0.505 0.523

COL1A1 IGF1 9606.ENSP00000225964 9606.ENSP00000302665 0 0 0 0 0.063 0 0 0.52 0.531

COL1A1 TNF 9606.ENSP00000225964 9606.ENSP00000398698 0 0 0 0 0 0.066 0 0.526 0.539

COL1A1 PPARG 9606.ENSP00000225964 9606.ENSP00000287820 0 0 0 0 0 0.056 0 0.533 0.54

COL1A1 FGF2 9606.ENSP00000225964 9606.ENSP00000264498 0 0 0 0 0.076 0.056 0 0.533 0.557

COL1A1 IL6 9606.ENSP00000225964 9606.ENSP00000385675 0 0 0 0 0.065 0 0 0.564 0.575

COL1A1 SMAD7 9606.ENSP00000225964 9606.ENSP00000262158 0 0 0 0 0 0.182 0 0.528 0.597

COL1A1 MMP1 9606.ENSP00000225964 9606.ENSP00000322788 0 0 0 0 0.128 0.059 0 0.611 0.653

COL1A1 VDR 9606.ENSP00000225964 9606.ENSP00000447173 0 0 0 0 0.107 0.345 0 0.456 0.654

COL1A1 MMP3 9606.ENSP00000225964 9606.ENSP00000299855 0 0 0 0 0.159 0.059 0 0.617 0.671

COL1A1 SPP1 9606.ENSP00000225964 9606.ENSP00000378517 0 0 0 0 0.063 0 0 0.714 0.72

COL1A1 MMP9 9606.ENSP00000225964 9606.ENSP00000361405 0 0 0 0 0.077 0.227 0 0.639 0.72

COL1A1 P4HB 9606.ENSP00000225964 9606.ENSP00000327801 0 0 0 0 0.069 0.217 0.6 0.339 0.781

COL1A1 PLOD2 9606.ENSP00000225964 9606.ENSP00000282903 0 0 0 0 0.146 0.145 0.6 0.6 0.867

COL1A1 COL3A1 9606.ENSP00000225964 9606.ENSP00000304408 0 0 0.439 0.951 0.944 0 0.8 0.892 0.989

COL3A1 PLOD1 9606.ENSP00000304408 9606.ENSP00000196061 0 0 0 0 0.096 0.144 0.6 0.543 0.839

COL3A1 MMP2 9606.ENSP00000304408 9606.ENSP00000219070 0 0 0 0 0.478 0.153 0 0.558 0.787

COL3A1 TGFB1 9606.ENSP00000304408 9606.ENSP00000221930 0 0 0 0 0 0.08 0 0.559 0.577

COL3A1 SMAD7 9606.ENSP00000304408 9606.ENSP00000262158 0 0 0 0 0 0.182 0 0.326 0.425

COL3A1 IL1B 9606.ENSP00000304408 9606.ENSP00000263341 0 0 0 0 0 0 0 0.414 0.414

COL3A1 FGF2 9606.ENSP00000304408 9606.ENSP00000264498 0 0 0 0 0.089 0.056 0 0.37 0.411

COL3A1 PLOD2 9606.ENSP00000304408 9606.ENSP00000282903 0 0 0 0 0.115 0.144 0.6 0.404 0.795

COL3A1 VCAM1 9606.ENSP00000304408 9606.ENSP00000294728 0 0 0 0 0.222 0 0 0.325 0.452

COL3A1 MMP3 9606.ENSP00000304408 9606.ENSP00000299855 0 0 0 0 0.135 0.059 0 0.558 0.609

COL3A1 IGF1 9606.ENSP00000304408 9606.ENSP00000302665 0 0 0 0 0.114 0 0 0.391 0.437

COL3A1 SPP1 9606.ENSP00000304408 9606.ENSP00000378517 0 0 0 0 0.062 0 0 0.414 0.426

COL3A1 TNF 9606.ENSP00000304408 9606.ENSP00000398698 0 0 0 0 0 0.102 0 0.395 0.433

COL3A1 IL6 9606.ENSP00000304408 9606.ENSP00000385675 0 0 0 0 0.063 0 0 0.456 0.468

COL3A1 EDNRA 9606.ENSP00000304408 9606.ENSP00000315011 0 0 0 0 0.351 0 0 0.226 0.476

COL3A1 MMP1 9606.ENSP00000304408 9606.ENSP00000322788 0 0 0 0 0.088 0.059 0 0.505 0.538

COL3A1 MMP9 9606.ENSP00000304408 9606.ENSP00000361405 0 0 0 0 0.078 0.059 0 0.556 0.582

COL3A1 P4HB 9606.ENSP00000304408 9606.ENSP00000327801 0 0 0 0 0.062 0.058 0.6 0.235 0.693

COLQ PLOD1 9606.ENSP00000373298 9606.ENSP00000196061 0 0 0 0 0 0.402 0 0.048 0.407

COLQ PLOD2 9606.ENSP00000373298 9606.ENSP00000282903 0 0 0 0 0 0.695 0 0.048 0.697

CRH IL2 9606.ENSP00000276571 9606.ENSP00000226730 0 0 0 0 0 0 0 0.503 0.503

CRH NR3C1 9606.ENSP00000276571 9606.ENSP00000231509 0 0 0 0 0 0 0 0.838 0.838

CRH CRP 9606.ENSP00000276571 9606.ENSP00000255030 0 0 0 0 0 0 0 0.423 0.422

CRH SLC6A4 9606.ENSP00000276571 9606.ENSP00000261707 0 0 0 0 0.064 0 0 0.681 0.688

CRH IL1B 9606.ENSP00000276571 9606.ENSP00000263341 0 0 0 0 0 0 0 0.609 0.609

CRH REN 9606.ENSP00000276571 9606.ENSP00000272190 0 0 0 0 0 0 0 0.503 0.503

CRH IL10 9606.ENSP00000276571 9606.ENSP00000412237 0 0 0 0 0 0 0 0.433 0.433

CRH GRIN2B 9606.ENSP00000276571 9606.ENSP00000477455 0 0 0 0 0.096 0 0 0.455 0.486

CRH ESR1 9606.ENSP00000276571 9606.ENSP00000405330 0 0 0 0 0 0 0 0.497 0.497

CRH IGF1 9606.ENSP00000276571 9606.ENSP00000302665 0 0 0 0 0.062 0 0 0.505 0.515

CRH HSD11B1 9606.ENSP00000276571 9606.ENSP00000355995 0 0 0 0 0 0 0 0.53 0.53

CRH TNF 9606.ENSP00000276571 9606.ENSP00000398698 0 0 0 0 0 0 0 0.539 0.539

CRH NGF 9606.ENSP00000276571 9606.ENSP00000358525 0 0 0 0 0.063 0 0 0.531 0.542

CRH HTR2A 9606.ENSP00000276571 9606.ENSP00000437737 0 0 0 0 0.083 0 0 0.575 0.594

CRH IL6 9606.ENSP00000276571 9606.ENSP00000385675 0 0 0 0 0 0 0 0.634 0.634

CRH INS 9606.ENSP00000276571 9606.ENSP00000380432 0 0 0 0 0 0 0 0.681 0.681

CRH TH 9606.ENSP00000276571 9606.ENSP00000370571 0 0 0 0 0.063 0 0 0.689 0.696

CRH NR3C2 9606.ENSP00000276571 9606.ENSP00000350815 0 0 0 0 0 0 0 0.7 0.7

CRH SST 9606.ENSP00000276571 9606.ENSP00000287641 0 0 0 0 0.161 0 0 0.777 0.805

CRH LEP 9606.ENSP00000276571 9606.ENSP00000312652 0 0 0 0 0 0 0 0.859 0.859

CRP TNFRSF1A 9606.ENSP00000255030 9606.ENSP00000162749 0 0 0 0 0 0 0 0.601 0.601

CRP MIF 9606.ENSP00000255030 9606.ENSP00000215754 0 0 0 0 0 0 0 0.558 0.558

CRP HMOX1 9606.ENSP00000255030 9606.ENSP00000216117 0 0 0 0 0 0 0 0.478 0.478

CRP NFKBIA 9606.ENSP00000255030 9606.ENSP00000216797 0 0 0 0 0 0 0 0.43 0.43

CRP MMP2 9606.ENSP00000255030 9606.ENSP00000219070 0 0 0 0 0 0 0 0.519 0.519

CRP TGFB1 9606.ENSP00000255030 9606.ENSP00000221930 0 0 0 0 0 0 0 0.509 0.509

CRP PON1 9606.ENSP00000255030 9606.ENSP00000222381 0 0 0 0 0.099 0 0 0.663 0.683

CRP MPO 9606.ENSP00000255030 9606.ENSP00000225275 0 0 0 0 0 0 0 0.805 0.805

CRP IL2 9606.ENSP00000255030 9606.ENSP00000226730 0 0 0 0 0 0 0 0.703 0.703

CRP IFNG 9606.ENSP00000255030 9606.ENSP00000229135 0 0 0 0 0 0 0 0.747 0.747

CRP IL4 9606.ENSP00000255030 9606.ENSP00000231449 0 0 0 0 0 0 0 0.683 0.683

CRP NR3C1 9606.ENSP00000255030 9606.ENSP00000231509 0 0 0 0 0 0 0 0.455 0.455

CRP FGF23 9606.ENSP00000255030 9606.ENSP00000237837 0 0 0 0 0 0 0 0.637 0.637

CRP EGFR 9606.ENSP00000255030 9606.ENSP00000275493 0 0 0 0 0.062 0 0 0.39 0.403

CRP ESR1 9606.ENSP00000255030 9606.ENSP00000405330 0 0 0 0 0.062 0 0 0.391 0.404

CRP EGF 9606.ENSP00000255030 9606.ENSP00000265171 0 0 0 0 0 0 0 0.405 0.405

CRP NOD2 9606.ENSP00000255030 9606.ENSP00000300589 0 0 0 0 0 0 0 0.407 0.407

CRP MYD88 9606.ENSP00000255030 9606.ENSP00000401399 0 0 0 0 0 0 0 0.415 0.415

CRP S100A9 9606.ENSP00000255030 9606.ENSP00000357727 0 0 0 0 0 0 0 0.416 0.416

CRP FGF2 9606.ENSP00000255030 9606.ENSP00000264498 0 0 0 0 0.062 0 0 0.405 0.418

CRP CYP3A4 9606.ENSP00000255030 9606.ENSP00000337915 0 0 0 0 0.085 0 0 0.392 0.42

CRP TP53 9606.ENSP00000255030 9606.ENSP00000269305 0 0 0 0 0 0 0 0.42 0.42

CRP PF4 9606.ENSP00000255030 9606.ENSP00000296029 0 0 0 0 0 0 0 0.457 0.457

CRP JUN 9606.ENSP00000255030 9606.ENSP00000360266 0 0 0 0 0 0 0 0.461 0.461

CRP FOXP3 9606.ENSP00000255030 9606.ENSP00000365380 0 0 0 0 0 0 0 0.468 0.468

CRP LTA 9606.ENSP00000255030 9606.ENSP00000403495 0 0 0 0 0 0 0 0.48 0.48

CRP TPO 9606.ENSP00000255030 9606.ENSP00000318820 0 0 0 0 0 0 0 0.493 0.493

CRP VDR 9606.ENSP00000255030 9606.ENSP00000447173 0 0 0 0 0 0 0 0.502 0.502

CRP HMGCR 9606.ENSP00000255030 9606.ENSP00000287936 0 0 0 0 0 0 0 0.505 0.505

CRP SPP1 9606.ENSP00000255030 9606.ENSP00000378517 0 0 0 0 0 0 0 0.52 0.52

CRP MMP1 9606.ENSP00000255030 9606.ENSP00000322788 0 0 0 0 0.052 0 0 0.518 0.523

CRP LPL 9606.ENSP00000255030 9606.ENSP00000309757 0 0 0 0 0 0 0 0.556 0.556

CRP ORM1 9606.ENSP00000255030 9606.ENSP00000259396 0 0 0 0 0.113 0 0 0.539 0.574

CRP IL13 9606.ENSP00000255030 9606.ENSP00000304915 0 0 0 0 0 0 0 0.595 0.595

CRP CSF2 9606.ENSP00000255030 9606.ENSP00000296871 0 0 0 0 0 0 0 0.597 0.597

CRP ELANE 9606.ENSP00000255030 9606.ENSP00000466090 0 0 0 0 0 0 0 0.608 0.608

CRP PPARG 9606.ENSP00000255030 9606.ENSP00000287820 0 0 0 0 0 0 0 0.61 0.61

CRP THBD 9606.ENSP00000255030 9606.ENSP00000366307 0 0 0 0 0 0 0 0.636 0.636

CRP CXCL10 9606.ENSP00000255030 9606.ENSP00000305651 0 0 0 0 0 0 0 0.637 0.637

CRP TNFRSF1B 9606.ENSP00000255030 9606.ENSP00000365435 0 0 0 0 0.062 0 0 0.636 0.643

CRP MMP3 9606.ENSP00000255030 9606.ENSP00000299855 0 0 0 0 0 0 0 0.654 0.654

CRP IL1A 9606.ENSP00000255030 9606.ENSP00000263339 0 0 0 0 0.063 0 0 0.65 0.658

CRP LCN2 9606.ENSP00000255030 9606.ENSP00000362108 0 0 0 0 0 0 0 0.667 0.667

CRP NOS3 9606.ENSP00000255030 9606.ENSP00000297494 0 0 0 0 0 0 0 0.681 0.681

CRP IGF1 9606.ENSP00000255030 9606.ENSP00000302665 0 0 0 0 0.062 0 0 0.681 0.687

CRP IL1RN 9606.ENSP00000255030 9606.ENSP00000259206 0 0 0 0 0.062 0 0 0.681 0.687

CRP TLR4 9606.ENSP00000255030 9606.ENSP00000363089 0 0 0 0 0 0 0 0.694 0.694

CRP IL17A 9606.ENSP00000255030 9606.ENSP00000344192 0 0 0 0 0 0 0 0.702 0.702

CRP EDN1 9606.ENSP00000255030 9606.ENSP00000368683 0 0 0 0 0 0 0 0.704 0.704

CRP MMP9 9606.ENSP00000255030 9606.ENSP00000361405 0 0 0 0 0 0 0 0.706 0.706

CRP VEGFA 9606.ENSP00000255030 9606.ENSP00000478570 0 0 0 0 0 0 0 0.707 0.707

CRP REN 9606.ENSP00000255030 9606.ENSP00000272190 0 0 0 0 0 0 0 0.74 0.74

CRP SHBG 9606.ENSP00000255030 9606.ENSP00000369816 0 0 0 0 0.168 0 0 0.732 0.767

CRP VCAM1 9606.ENSP00000255030 9606.ENSP00000294728 0 0 0 0 0 0 0 0.802 0.802

CRP ICAM1 9606.ENSP00000255030 9606.ENSP00000264832 0 0 0 0 0 0 0 0.805 0.805

CRP SELE 9606.ENSP00000255030 9606.ENSP00000331736 0 0 0 0 0.063 0 0 0.806 0.811

CRP SELP 9606.ENSP00000255030 9606.ENSP00000263686 0 0 0 0 0.065 0 0 0.818 0.823

CRP IL10 9606.ENSP00000255030 9606.ENSP00000412237 0 0 0 0 0 0 0 0.838 0.838

CRP CXCL8 9606.ENSP00000255030 9606.ENSP00000306512 0 0 0 0 0 0 0 0.857 0.857

CRP IL1B 9606.ENSP00000255030 9606.ENSP00000263341 0 0 0 0 0.062 0 0 0.859 0.862

CRP F2 9606.ENSP00000255030 9606.ENSP00000308541 0 0 0 0 0.153 0 0 0.856 0.872

CRP GPT 9606.ENSP00000255030 9606.ENSP00000378408 0 0 0 0 0.062 0 0 0.88 0.882

CRP TNF 9606.ENSP00000255030 9606.ENSP00000398698 0 0 0 0 0 0 0 0.911 0.911

CRP INS 9606.ENSP00000255030 9606.ENSP00000380432 0 0 0 0 0.063 0 0 0.91 0.912

CRP IL6 9606.ENSP00000255030 9606.ENSP00000385675 0 0 0 0 0 0 0 0.916 0.916

CRP LEP 9606.ENSP00000255030 9606.ENSP00000312652 0 0 0 0 0 0.213 0 0.946 0.956

CSF2 TNFRSF1A 9606.ENSP00000296871 9606.ENSP00000162749 0 0 0 0 0 0 0 0.56 0.56

CSF2 MIF 9606.ENSP00000296871 9606.ENSP00000215754 0 0 0 0 0 0 0 0.476 0.476

CSF2 HMOX1 9606.ENSP00000296871 9606.ENSP00000216117 0 0 0 0 0 0 0 0.401 0.4

CSF2 CTSG 9606.ENSP00000296871 9606.ENSP00000216336 0 0 0 0 0 0 0 0.416 0.416

CSF2 NFKBIA 9606.ENSP00000296871 9606.ENSP00000216797 0 0 0 0 0.074 0 0 0.562 0.577

CSF2 MMP2 9606.ENSP00000296871 9606.ENSP00000219070 0 0 0 0 0 0 0 0.459 0.459

CSF2 TGFB1 9606.ENSP00000296871 9606.ENSP00000221930 0 0 0 0 0 0 0 0.626 0.626

CSF2 MPO 9606.ENSP00000296871 9606.ENSP00000225275 0 0 0 0 0 0 0 0.608 0.608

CSF2 IL2 9606.ENSP00000296871 9606.ENSP00000226730 0 0 0 0 0 0 0.5 0.884 0.939

CSF2 KITLG 9606.ENSP00000296871 9606.ENSP00000228280 0 0 0 0 0 0.213 0 0.98 0.983

CSF2 IFNG 9606.ENSP00000296871 9606.ENSP00000229135 0 0 0 0 0.076 0 0 0.856 0.862

CSF2 IL4 9606.ENSP00000296871 9606.ENSP00000231449 0 0 0 0 0 0 0 0.914 0.914

CSF2 IRF1 9606.ENSP00000296871 9606.ENSP00000245414 0 0 0 0 0 0 0 0.501 0.501

CSF2 IL1RN 9606.ENSP00000296871 9606.ENSP00000259206 0 0 0 0 0.08 0 0 0.537 0.556

CSF2 IL1A 9606.ENSP00000296871 9606.ENSP00000263339 0 0 0 0 0.202 0 0.9 0.857 0.987

CSF2 IL1B 9606.ENSP00000296871 9606.ENSP00000263341 0 0 0 0 0.134 0 0.9 0.874 0.988

CSF2 SELP 9606.ENSP00000296871 9606.ENSP00000263686 0 0 0 0 0 0 0 0.467 0.467

CSF2 FGF2 9606.ENSP00000296871 9606.ENSP00000264498 0 0 0 0 0.062 0 0 0.681 0.687

CSF2 ICAM1 9606.ENSP00000296871 9606.ENSP00000264832 0 0 0 0 0.088 0 0 0.732 0.745

CSF2 EGF 9606.ENSP00000296871 9606.ENSP00000265171 0 0 0 0 0 0 0 0.658 0.658

CSF2 TP53 9606.ENSP00000296871 9606.ENSP00000269305 0 0 0 0 0 0 0 0.646 0.646

CSF2 ERBB2 9606.ENSP00000296871 9606.ENSP00000269571 0 0 0 0 0 0 0 0.566 0.566

CSF2 EGFR 9606.ENSP00000296871 9606.ENSP00000275493 0 0 0 0 0 0 0.6 0.521 0.8

CSF2 CXCL13 9606.ENSP00000296871 9606.ENSP00000286758 0 0 0 0 0 0 0 0.519 0.519

CSF2 PPARG 9606.ENSP00000296871 9606.ENSP00000287820 0 0 0 0 0 0 0 0.469 0.469

CSF2 KIT 9606.ENSP00000296871 9606.ENSP00000288135 0 0 0 0 0 0 0 0.782 0.783

CSF2 VCAM1 9606.ENSP00000296871 9606.ENSP00000294728 0 0 0 0 0 0 0 0.676 0.676

CSF2 PF4 9606.ENSP00000296871 9606.ENSP00000296029 0 0 0 0 0.049 0 0 0.47 0.475

CSF2 TLR3 9606.ENSP00000296871 9606.ENSP00000296795 0 0 0 0 0 0 0 0.67 0.67

CSF2 LCN2 9606.ENSP00000296871 9606.ENSP00000362108 0 0 0 0 0 0 0 0.402 0.402

CSF2 EDN1 9606.ENSP00000296871 9606.ENSP00000368683 0 0 0 0 0.062 0 0 0.402 0.415

CSF2 HPRT1 9606.ENSP00000296871 9606.ENSP00000298556 0 0 0 0 0 0 0 0.429 0.429

CSF2 SPP1 9606.ENSP00000296871 9606.ENSP00000378517 0 0 0 0 0 0 0 0.46 0.459

CSF2 TNFRSF1B 9606.ENSP00000296871 9606.ENSP00000365435 0 0 0 0 0.062 0 0 0.454 0.465

CSF2 SOCS1 9606.ENSP00000296871 9606.ENSP00000329418 0 0 0 0 0.068 0 0 0.467 0.482

CSF2 FAS 9606.ENSP00000296871 9606.ENSP00000347979 0 0 0 0 0.062 0 0 0.481 0.492

CSF2 MMP1 9606.ENSP00000296871 9606.ENSP00000322788 0 0 0 0 0.098 0 0 0.464 0.496

CSF2 HIF1A 9606.ENSP00000296871 9606.ENSP00000437955 0 0 0 0 0 0 0 0.496 0.496

CSF2 ITGAL 9606.ENSP00000296871 9606.ENSP00000349252 0 0 0 0 0 0 0 0.504 0.504

CSF2 THBD 9606.ENSP00000296871 9606.ENSP00000366307 0 0 0 0 0 0 0 0.505 0.505

CSF2 NOD2 9606.ENSP00000296871 9606.ENSP00000300589 0 0 0 0 0.062 0 0 0.504 0.514

CSF2 MMP3 9606.ENSP00000296871 9606.ENSP00000299855 0 0 0 0 0.066 0 0 0.502 0.515

CSF2 ELANE 9606.ENSP00000296871 9606.ENSP00000466090 0 0 0 0 0 0 0 0.517 0.517

CSF2 SELE 9606.ENSP00000296871 9606.ENSP00000331736 0 0 0 0 0 0 0 0.518 0.518

CSF2 LEP 9606.ENSP00000296871 9606.ENSP00000312652 0 0 0 0 0 0 0 0.523 0.523

CSF2 IGF1 9606.ENSP00000296871 9606.ENSP00000302665 0 0 0 0 0 0 0 0.528 0.528

CSF2 NGF 9606.ENSP00000296871 9606.ENSP00000358525 0 0 0 0 0 0 0 0.528 0.528

CSF2 NOS2 9606.ENSP00000296871 9606.ENSP00000327251 0 0 0 0 0.063 0 0 0.533 0.543

CSF2 CX3CR1 9606.ENSP00000296871 9606.ENSP00000351059 0 0 0 0 0 0 0 0.558 0.558

CSF2 JUN 9606.ENSP00000296871 9606.ENSP00000360266 0 0 0 0 0 0 0 0.559 0.559

CSF2 FASLG 9606.ENSP00000296871 9606.ENSP00000356694 0 0 0 0 0 0 0 0.561 0.561

CSF2 IDO1 9606.ENSP00000296871 9606.ENSP00000430950 0 0 0 0 0 0 0 0.565 0.565

CSF2 INS 9606.ENSP00000296871 9606.ENSP00000380432 0 0 0 0 0 0 0 0.574 0.574

CSF2 CXCR4 9606.ENSP00000296871 9606.ENSP00000386884 0 0 0 0 0 0 0 0.606 0.606

CSF2 MMP9 9606.ENSP00000296871 9606.ENSP00000361405 0 0 0 0 0 0 0 0.625 0.625

CSF2 IL17RA 9606.ENSP00000296871 9606.ENSP00000320936 0 0 0 0 0 0 0 0.628 0.628

CSF2 MYD88 9606.ENSP00000296871 9606.ENSP00000401399 0 0 0 0 0 0 0 0.667 0.667

CSF2 FOXP3 9606.ENSP00000296871 9606.ENSP00000365380 0 0 0 0 0 0 0 0.737 0.737

CSF2 TLR4 9606.ENSP00000296871 9606.ENSP00000363089 0 0 0 0 0 0 0 0.764 0.765

CSF2 LTA 9606.ENSP00000296871 9606.ENSP00000403495 0 0 0 0 0.09 0 0 0.756 0.769

CSF2 VEGFA 9606.ENSP00000296871 9606.ENSP00000478570 0 0 0 0 0 0 0 0.802 0.802

CSF2 IL17A 9606.ENSP00000296871 9606.ENSP00000344192 0 0 0 0 0 0 0 0.878 0.878

CSF2 IL13 9606.ENSP00000296871 9606.ENSP00000304915 0 0 0 0 0.062 0 0 0.878 0.881

CSF2 STAT1 9606.ENSP00000296871 9606.ENSP00000354394 0 0 0 0 0 0 0.9 0.64 0.962

CSF2 STAT5A 9606.ENSP00000296871 9606.ENSP00000341208 0 0 0 0 0 0 0.9 0.695 0.968

CSF2 CXCL10 9606.ENSP00000296871 9606.ENSP00000305651 0 0 0 0 0.083 0 0.9 0.822 0.982

CSF2 CXCL8 9606.ENSP00000296871 9606.ENSP00000306512 0 0 0 0 0.148 0 0.9 0.876 0.988

CSF2 IL10 9606.ENSP00000296871 9606.ENSP00000412237 0 0 0 0 0.062 0 0.9 0.884 0.988

CSF2 TNF 9606.ENSP00000296871 9606.ENSP00000398698 0 0 0 0 0.131 0 0.9 0.902 0.99

CSF2 IL6 9606.ENSP00000296871 9606.ENSP00000385675 0 0 0 0 0.173 0 0.9 0.91 0.991

CTSD CTSG 9606.ENSP00000236671 9606.ENSP00000216336 0 0 0 0 0 0 0 0.566 0.566

CTSD MMP2 9606.ENSP00000236671 9606.ENSP00000219070 0 0 0 0 0.052 0 0 0.476 0.482

CTSD EGF 9606.ENSP00000236671 9606.ENSP00000265171 0 0 0 0 0 0 0 0.401 0.4

CTSD VEGFA 9606.ENSP00000236671 9606.ENSP00000478570 0 0 0 0 0 0 0 0.405 0.405

CTSD IL1B 9606.ENSP00000236671 9606.ENSP00000263341 0 0 0 0 0.062 0 0 0.395 0.408

CTSD MMP3 9606.ENSP00000236671 9606.ENSP00000299855 0 0 0 0 0.052 0 0 0.419 0.426

CTSD MTOR 9606.ENSP00000236671 9606.ENSP00000354558 0 0 0 0 0 0.106 0 0.427 0.466

CTSD MMP9 9606.ENSP00000236671 9606.ENSP00000361405 0 0 0 0 0.081 0 0 0.473 0.495

CTSD HSPB1 9606.ENSP00000236671 9606.ENSP00000248553 0 0 0 0 0.098 0 0 0.465 0.496

CTSD INS 9606.ENSP00000236671 9606.ENSP00000380432 0 0 0 0 0 0.213 0 0.406 0.512

CTSD CYCS 9606.ENSP00000236671 9606.ENSP00000307786 0 0 0 0 0.062 0 0 0.504 0.514

CTSD TP53 9606.ENSP00000236671 9606.ENSP00000269305 0 0 0 0 0 0 0 0.518 0.518

CTSD TNF 9606.ENSP00000236671 9606.ENSP00000398698 0 0 0 0 0.062 0 0 0.536 0.547

CTSD ERBB2 9606.ENSP00000236671 9606.ENSP00000269571 0 0 0 0 0 0 0 0.559 0.559

CTSD FURIN 9606.ENSP00000236671 9606.ENSP00000483552 0 0 0 0 0.148 0.297 0 0.342 0.572

CTSD EGFR 9606.ENSP00000236671 9606.ENSP00000275493 0 0 0 0 0 0.103 0 0.567 0.595

CTSD P4HB 9606.ENSP00000236671 9606.ENSP00000327801 0 0 0 0 0.324 0.06 0 0.424 0.602

CTSD HSPA5 9606.ENSP00000236671 9606.ENSP00000324173 0 0 0 0 0.062 0.153 0 0.565 0.624

CTSD SNCA 9606.ENSP00000236671 9606.ENSP00000338345 0 0 0 0 0 0.27 0 0.593 0.69

CTSD TPO 9606.ENSP00000236671 9606.ENSP00000318820 0 0 0 0 0.089 0 0.9 0.047 0.905

CTSD ESR1 9606.ENSP00000236671 9606.ENSP00000405330 0 0 0 0 0.088 0.056 0.9 0.606 0.961

CTSG MMP3 9606.ENSP00000216336 9606.ENSP00000299855 0 0 0 0 0 0 0 0.401 0.401

CTSG ITGB2 9606.ENSP00000216336 9606.ENSP00000380948 0 0 0 0 0.152 0 0 0.323 0.401

CTSG TLR4 9606.ENSP00000216336 9606.ENSP00000363089 0 0 0 0 0.076 0.056 0 0.403 0.433

CTSG TFPI 9606.ENSP00000216336 9606.ENSP00000233156 0 0 0 0 0 0.143 0 0.367 0.434

CTSG S100A9 9606.ENSP00000216336 9606.ENSP00000357727 0 0 0 0 0.172 0 0 0.359 0.446

CTSG LCN2 9606.ENSP00000216336 9606.ENSP00000362108 0 0 0 0 0.088 0 0 0.425 0.453

CTSG MMP2 9606.ENSP00000216336 9606.ENSP00000219070 0 0 0 0 0 0 0 0.455 0.455

CTSG REN 9606.ENSP00000216336 9606.ENSP00000272190 0 0 0 0 0 0 0 0.473 0.473

CTSG LYZ 9606.ENSP00000216336 9606.ENSP00000261267 0 0 0 0 0.213 0 0 0.393 0.501

CTSG SELP 9606.ENSP00000216336 9606.ENSP00000263686 0 0 0 0 0.118 0 0 0.46 0.503

CTSG IL6 9606.ENSP00000216336 9606.ENSP00000385675 0 0 0 0 0 0 0 0.505 0.505

CTSG VCAM1 9606.ENSP00000216336 9606.ENSP00000294728 0 0 0 0 0 0.213 0 0.417 0.521

CTSG TNF 9606.ENSP00000216336 9606.ENSP00000398698 0 0 0 0 0.07 0 0 0.521 0.535

CTSG CXCR4 9606.ENSP00000216336 9606.ENSP00000386884 0 0 0 0 0.08 0.263 0 0.397 0.555

CTSG IL1B 9606.ENSP00000216336 9606.ENSP00000263341 0 0 0 0 0.076 0 0 0.556 0.572

CTSG LTF 9606.ENSP00000216336 9606.ENSP00000231751 0 0 0 0 0.152 0.169 0 0.45 0.578

CTSG CXCL8 9606.ENSP00000216336 9606.ENSP00000306512 0 0 0 0 0.069 0 0 0.57 0.582

CTSG F2RL1 9606.ENSP00000216336 9606.ENSP00000296677 0 0 0 0 0 0.213 0.6 0.216 0.731

CTSG ELANE 9606.ENSP00000216336 9606.ENSP00000466090 0 0 0.437 0.821 0.784 0 0.3 0.911 0.877

CTSG IGF1 9606.ENSP00000216336 9606.ENSP00000302665 0 0 0 0 0 0 0.9 0.156 0.911

CTSG MPO 9606.ENSP00000216336 9606.ENSP00000225275 0 0 0 0 0.611 0.056 0 0.816 0.927

CTSG MMP1 9606.ENSP00000216336 9606.ENSP00000322788 0 0 0 0 0 0 0.9 0.391 0.936

CTSG MMP9 9606.ENSP00000216336 9606.ENSP00000361405 0 0 0 0 0.089 0 0.9 0.602 0.96

CX3CR1 IL2 9606.ENSP00000351059 9606.ENSP00000226730 0 0 0 0 0 0 0 0.501 0.501

CX3CR1 IFNG 9606.ENSP00000351059 9606.ENSP00000229135 0 0 0 0 0 0 0 0.545 0.545

CX3CR1 IL4 9606.ENSP00000351059 9606.ENSP00000231449 0 0 0 0 0 0 0 0.602 0.602

CX3CR1 NR3C1 9606.ENSP00000351059 9606.ENSP00000231509 0 0 0 0 0 0 0 0.448 0.448

CX3CR1 LYZ 9606.ENSP00000351059 9606.ENSP00000261267 0 0 0 0 0.141 0 0 0.339 0.408

CX3CR1 IL1A 9606.ENSP00000351059 9606.ENSP00000263339 0 0 0 0 0 0 0 0.47 0.47

CX3CR1 IL1B 9606.ENSP00000351059 9606.ENSP00000263341 0 0 0 0 0.065 0 0 0.696 0.703

CX3CR1 SELP 9606.ENSP00000351059 9606.ENSP00000263686 0 0 0 0 0.098 0 0 0.406 0.441

CX3CR1 ICAM1 9606.ENSP00000351059 9606.ENSP00000264832 0 0 0 0 0 0 0 0.529 0.529

CX3CR1 CXCL13 9606.ENSP00000351059 9606.ENSP00000286758 0 0 0 0 0 0 0.6 0.508 0.795

CX3CR1 KIT 9606.ENSP00000351059 9606.ENSP00000288135 0 0 0 0 0 0 0 0.429 0.429

CX3CR1 VCAM1 9606.ENSP00000351059 9606.ENSP00000294728 0 0 0 0 0 0 0 0.508 0.508

CX3CR1 PF4 9606.ENSP00000351059 9606.ENSP00000296029 0 0 0 0 0.076 0 0.6 0.414 0.764

CX3CR1 TLR3 9606.ENSP00000351059 9606.ENSP00000296795 0 0 0 0 0 0 0 0.421 0.42

CX3CR1 IGF1 9606.ENSP00000351059 9606.ENSP00000302665 0 0 0 0 0 0 0 0.407 0.407

CX3CR1 IL13 9606.ENSP00000351059 9606.ENSP00000304915 0 0 0 0 0 0 0 0.53 0.53

CX3CR1 CXCL10 9606.ENSP00000351059 9606.ENSP00000305651 0 0 0 0 0 0 0.6 0.604 0.835

CX3CR1 CXCL8 9606.ENSP00000351059 9606.ENSP00000306512 0 0 0 0 0 0 0.6 0.558 0.815

CX3CR1 NOS2 9606.ENSP00000351059 9606.ENSP00000327251 0 0 0 0 0 0 0 0.415 0.415

CX3CR1 IL17A 9606.ENSP00000351059 9606.ENSP00000344192 0 0 0 0 0 0 0 0.531 0.531

CX3CR1 ITGAL 9606.ENSP00000351059 9606.ENSP00000349252 0 0 0 0 0.139 0 0 0.458 0.513

CX3CR1 MMP9 9606.ENSP00000351059 9606.ENSP00000361405 0 0 0 0 0 0 0 0.462 0.462

CX3CR1 MYD88 9606.ENSP00000351059 9606.ENSP00000401399 0 0 0 0 0 0 0 0.469 0.469

CX3CR1 ITGB2 9606.ENSP00000351059 9606.ENSP00000380948 0 0 0 0 0.213 0 0 0.372 0.484

CX3CR1 HIF1A 9606.ENSP00000351059 9606.ENSP00000437955 0 0 0 0 0 0.273 0 0.329 0.491

CX3CR1 VEGFA 9606.ENSP00000351059 9606.ENSP00000478570 0 0 0 0 0 0 0 0.502 0.502

CX3CR1 FOXP3 9606.ENSP00000351059 9606.ENSP00000365380 0 0 0 0 0 0 0 0.556 0.556

CX3CR1 TLR4 9606.ENSP00000351059 9606.ENSP00000363089 0 0 0 0 0.099 0 0 0.619 0.642

CX3CR1 IL6 9606.ENSP00000351059 9606.ENSP00000385675 0 0 0 0 0 0 0 0.674 0.674

CX3CR1 IL10 9606.ENSP00000351059 9606.ENSP00000412237 0 0 0 0 0 0 0 0.676 0.676

CX3CR1 TNF 9606.ENSP00000351059 9606.ENSP00000398698 0 0 0 0 0 0 0 0.699 0.699

CXCL10 TNFRSF1A 9606.ENSP00000305651 9606.ENSP00000162749 0 0 0 0 0.062 0 0 0.518 0.528

CXCL10 MIF 9606.ENSP00000305651 9606.ENSP00000215754 0 0 0 0 0 0 0 0.494 0.494

CXCL10 HMOX1 9606.ENSP00000305651 9606.ENSP00000216117 0 0 0 0 0.065 0 0 0.474 0.487

CXCL10 NFKBIA 9606.ENSP00000305651 9606.ENSP00000216797 0 0 0 0 0.098 0 0 0.567 0.593

CXCL10 MMP2 9606.ENSP00000305651 9606.ENSP00000219070 0 0 0 0 0 0 0 0.424 0.424

CXCL10 TGFB1 9606.ENSP00000305651 9606.ENSP00000221930 0 0 0 0 0 0 0 0.458 0.458

CXCL10 MPO 9606.ENSP00000305651 9606.ENSP00000225275 0 0 0 0 0 0 0 0.467 0.467

CXCL10 NFKB1 9606.ENSP00000305651 9606.ENSP00000226574 0 0 0 0 0.106 0 0 0.383 0.425

CXCL10 IL2 9606.ENSP00000305651 9606.ENSP00000226730 0 0 0 0 0 0 0 0.805 0.805

CXCL10 IFNG 9606.ENSP00000305651 9606.ENSP00000229135 0 0 0 0 0.118 0 0 0.861 0.872

CXCL10 IL4 9606.ENSP00000305651 9606.ENSP00000231449 0 0 0 0 0 0 0 0.817 0.817

CXCL10 IRF1 9606.ENSP00000305651 9606.ENSP00000245414 0 0 0 0 0.378 0 0 0.689 0.798

CXCL10 IL1RN 9606.ENSP00000305651 9606.ENSP00000259206 0 0 0 0 0.231 0 0 0.582 0.665

CXCL10 IL1A 9606.ENSP00000305651 9606.ENSP00000263339 0 0 0 0 0.121 0 0.9 0.802 0.981

CXCL10 IL1B 9606.ENSP00000305651 9606.ENSP00000263341 0 0 0 0 0.242 0 0.9 0.863 0.988

CXCL10 SELP 9606.ENSP00000305651 9606.ENSP00000263686 0 0 0 0 0.076 0 0 0.451 0.471

CXCL10 FGF2 9606.ENSP00000305651 9606.ENSP00000264498 0 0 0 0 0 0 0 0.604 0.604

CXCL10 ICAM1 9606.ENSP00000305651 9606.ENSP00000264832 0 0 0 0 0.145 0 0 0.708 0.739

CXCL10 EGF 9606.ENSP00000305651 9606.ENSP00000265171 0 0 0 0 0 0 0 0.601 0.601

CXCL10 TP53 9606.ENSP00000305651 9606.ENSP00000269305 0 0 0 0 0.062 0 0 0.395 0.408

CXCL10 CXCL13 9606.ENSP00000305651 9606.ENSP00000286758 0 0 0 0 0.153 0 0.9 0.712 0.973

CXCL10 PPARG 9606.ENSP00000305651 9606.ENSP00000287820 0 0 0 0 0 0 0 0.456 0.456

CXCL10 VCAM1 9606.ENSP00000305651 9606.ENSP00000294728 0 0 0 0 0.098 0 0 0.723 0.74

CXCL10 PF4 9606.ENSP00000305651 9606.ENSP00000296029 0 0 0 0 0.054 0.237 0.9 0.766 0.98

CXCL10 TLR3 9606.ENSP00000305651 9606.ENSP00000296795 0 0 0 0 0.063 0 0 0.726 0.732

CXCL10 HPRT1 9606.ENSP00000305651 9606.ENSP00000298556 0 0 0 0 0 0 0 0.475 0.475

CXCL10 MMP3 9606.ENSP00000305651 9606.ENSP00000299855 0 0 0 0 0.062 0 0 0.51 0.52

CXCL10 NOD2 9606.ENSP00000305651 9606.ENSP00000300589 0 0 0 0 0.152 0 0 0.456 0.518

CXCL10 IL13 9606.ENSP00000305651 9606.ENSP00000304915 0 0 0 0 0 0 0 0.834 0.834

CXCL10 THBD 9606.ENSP00000305651 9606.ENSP00000366307 0 0 0 0 0.076 0 0 0.391 0.413

CXCL10 FAS 9606.ENSP00000305651 9606.ENSP00000347979 0 0 0 0 0.09 0 0 0.386 0.417

CXCL10 NGF 9606.ENSP00000305651 9606.ENSP00000358525 0 0 0 0 0 0 0 0.419 0.418

CXCL10 IL17RA 9606.ENSP00000305651 9606.ENSP00000320936 0 0 0 0 0.062 0 0 0.426 0.438

CXCL10 HDAC1 9606.ENSP00000305651 9606.ENSP00000362649 0 0 0 0 0 0 0 0.448 0.448

CXCL10 GPT 9606.ENSP00000305651 9606.ENSP00000378408 0 0 0 0 0 0 0 0.466 0.465

CXCL10 INS 9606.ENSP00000305651 9606.ENSP00000380432 0 0 0 0 0 0 0 0.469 0.469

CXCL10 SPP1 9606.ENSP00000305651 9606.ENSP00000378517 0 0 0 0 0.062 0 0 0.458 0.469

CXCL10 MMP1 9606.ENSP00000305651 9606.ENSP00000322788 0 0 0 0 0.065 0 0 0.456 0.469

CXCL10 LEP 9606.ENSP00000305651 9606.ENSP00000312652 0 0 0 0 0 0 0 0.476 0.476

CXCL10 FASLG 9606.ENSP00000305651 9606.ENSP00000356694 0 0 0 0 0 0 0 0.502 0.502

CXCL10 TNFAIP3 9606.ENSP00000305651 9606.ENSP00000481570 0 0 0 0 0.192 0 0 0.416 0.508

CXCL10 TNFRSF1B 9606.ENSP00000305651 9606.ENSP00000365435 0 0 0 0 0.116 0 0 0.466 0.508

CXCL10 JUN 9606.ENSP00000305651 9606.ENSP00000360266 0 0 0 0 0.049 0 0 0.51 0.514

CXCL10 LCN2 9606.ENSP00000305651 9606.ENSP00000362108 0 0 0 0 0.085 0 0 0.504 0.527

CXCL10 NOS2 9606.ENSP00000305651 9606.ENSP00000327251 0 0 0 0 0.064 0 0 0.526 0.537

CXCL10 SELE 9606.ENSP00000305651 9606.ENSP00000331736 0 0 0 0 0.076 0 0 0.55 0.566

CXCL10 SOCS1 9606.ENSP00000305651 9606.ENSP00000329418 0 0 0 0 0.137 0 0 0.519 0.567

CXCL10 MMP9 9606.ENSP00000305651 9606.ENSP00000361405 0 0 0 0 0.14 0 0 0.611 0.651

CXCL10 FOXP3 9606.ENSP00000305651 9606.ENSP00000365380 0 0 0 0 0 0 0 0.657 0.657

CXCL10 MYD88 9606.ENSP00000305651 9606.ENSP00000401399 0 0 0 0 0.098 0 0 0.689 0.708

CXCL10 IDO1 9606.ENSP00000305651 9606.ENSP00000430950 0 0 0 0 0.411 0 0 0.56 0.73

CXCL10 LTA 9606.ENSP00000305651 9606.ENSP00000403495 0 0 0 0 0.15 0 0 0.702 0.736

CXCL10 VEGFA 9606.ENSP00000305651 9606.ENSP00000478570 0 0 0 0 0 0 0 0.739 0.739

CXCL10 IL17A 9606.ENSP00000305651 9606.ENSP00000344192 0 0 0 0 0.045 0 0 0.824 0.825

CXCL10 CXCR4 9606.ENSP00000305651 9606.ENSP00000386884 0 0 0 0 0 0 0.6 0.702 0.875

CXCL10 STAT1 9606.ENSP00000305651 9606.ENSP00000354394 0 0 0 0 0.707 0 0 0.745 0.922

CXCL10 CXCL8 9606.ENSP00000305651 9606.ENSP00000306512 0 0 0 0 0.145 0 0.6 0.879 0.955

CXCL10 TLR4 9606.ENSP00000305651 9606.ENSP00000363089 0 0 0 0 0.078 0 0 0.959 0.961

CXCL10 IL10 9606.ENSP00000305651 9606.ENSP00000412237 0 0 0 0 0.084 0 0.9 0.866 0.986

CXCL10 TNF 9606.ENSP00000305651 9606.ENSP00000398698 0 0 0 0 0.216 0 0.9 0.881 0.989

CXCL10 IL6 9606.ENSP00000305651 9606.ENSP00000385675 0 0 0 0 0.175 0 0.9 0.878 0.989

CXCL13 TNFRSF1A 9606.ENSP00000286758 9606.ENSP00000162749 0 0 0 0 0 0 0 0.4 0.4

CXCL13 IL2 9606.ENSP00000286758 9606.ENSP00000226730 0 0 0 0 0 0 0 0.556 0.556

CXCL13 IFNG 9606.ENSP00000286758 9606.ENSP00000229135 0 0 0 0 0.09 0 0 0.543 0.566

CXCL13 IL4 9606.ENSP00000286758 9606.ENSP00000231449 0 0 0 0 0 0 0 0.604 0.604

CXCL13 IL1A 9606.ENSP00000286758 9606.ENSP00000263339 0 0 0 0 0 0 0 0.504 0.504

CXCL13 IL1B 9606.ENSP00000286758 9606.ENSP00000263341 0 0 0 0 0.062 0 0 0.601 0.61

CXCL13 FGF2 9606.ENSP00000286758 9606.ENSP00000264498 0 0 0 0 0 0.213 0 0.294 0.42

CXCL13 ICAM1 9606.ENSP00000286758 9606.ENSP00000264832 0 0 0 0 0.062 0 0 0.545 0.555

CXCL13 VEGFA 9606.ENSP00000286758 9606.ENSP00000478570 0 0 0 0 0 0 0 0.421 0.42

CXCL13 TLR4 9606.ENSP00000286758 9606.ENSP00000363089 0 0 0 0 0.062 0 0 0.413 0.425

CXCL13 LEP 9606.ENSP00000286758 9606.ENSP00000312652 0 0 0 0 0 0 0 0.442 0.442

CXCL13 MMP9 9606.ENSP00000286758 9606.ENSP00000361405 0 0 0 0 0.1 0 0 0.414 0.45

CXCL13 LTA 9606.ENSP00000286758 9606.ENSP00000403495 0 0 0 0 0.085 0 0 0.489 0.513

CXCL13 IL13 9606.ENSP00000286758 9606.ENSP00000304915 0 0 0 0 0 0 0 0.556 0.556

CXCL13 FOXP3 9606.ENSP00000286758 9606.ENSP00000365380 0 0 0 0 0.064 0 0 0.564 0.574

CXCL13 VCAM1 9606.ENSP00000286758 9606.ENSP00000294728 0 0 0 0 0.09 0 0 0.564 0.587

CXCL13 IL17A 9606.ENSP00000286758 9606.ENSP00000344192 0 0 0 0 0.076 0 0 0.617 0.63

CXCL13 IL6 9606.ENSP00000286758 9606.ENSP00000385675 0 0 0 0 0 0 0 0.652 0.652

CXCL13 TNF 9606.ENSP00000286758 9606.ENSP00000398698 0 0 0 0 0.062 0 0 0.649 0.657

CXCL13 IL10 9606.ENSP00000286758 9606.ENSP00000412237 0 0 0 0 0.062 0 0 0.651 0.658

CXCL13 PF4 9606.ENSP00000286758 9606.ENSP00000296029 0 0 0 0 0 0 0.8 0.504 0.896

CXCL13 CXCR4 9606.ENSP00000286758 9606.ENSP00000386884 0 0 0 0 0.062 0 0.6 0.872 0.948

CXCL8 TNFRSF1A 9606.ENSP00000306512 9606.ENSP00000162749 0 0 0 0 0.069 0 0 0.72 0.728

CXCL8 MIF 9606.ENSP00000306512 9606.ENSP00000215754 0 0 0 0 0 0 0 0.73 0.73

CXCL8 HMOX1 9606.ENSP00000306512 9606.ENSP00000216117 0 0 0 0 0.077 0 0 0.66 0.673

CXCL8 NFKBIA 9606.ENSP00000306512 9606.ENSP00000216797 0 0 0 0 0.269 0 0 0.794 0.843

CXCL8 MMP2 9606.ENSP00000306512 9606.ENSP00000219070 0 0 0 0 0 0 0 0.688 0.688

CXCL8 TGFB1 9606.ENSP00000306512 9606.ENSP00000221930 0 0 0 0 0.096 0 0 0.652 0.671

CXCL8 MPO 9606.ENSP00000306512 9606.ENSP00000225275 0 0 0 0 0.065 0 0 0.766 0.771

CXCL8 NFKB1 9606.ENSP00000306512 9606.ENSP00000226574 0 0 0 0 0.118 0 0.9 0.569 0.958

CXCL8 IL2 9606.ENSP00000306512 9606.ENSP00000226730 0 0 0 0 0 0 0 0.873 0.873

CXCL8 KITLG 9606.ENSP00000306512 9606.ENSP00000228280 0 0 0 0 0 0 0 0.561 0.561

CXCL8 IFNG 9606.ENSP00000306512 9606.ENSP00000229135 0 0 0 0 0.062 0 0 0.859 0.862

CXCL8 IL4 9606.ENSP00000306512 9606.ENSP00000231449 0 0 0 0 0 0 0.9 0.873 0.986

CXCL8 NR3C1 9606.ENSP00000306512 9606.ENSP00000231509 0 0 0 0 0.062 0 0 0.506 0.516

CXCL8 IRF1 9606.ENSP00000306512 9606.ENSP00000245414 0 0 0 0 0.12 0 0 0.529 0.568

CXCL8 HSPB1 9606.ENSP00000306512 9606.ENSP00000248553 0 0 0 0 0 0 0 0.45 0.45

CXCL8 IL1RN 9606.ENSP00000306512 9606.ENSP00000259206 0 0 0 0 0.324 0 0 0.717 0.8

CXCL8 LYZ 9606.ENSP00000306512 9606.ENSP00000261267 0 0 0 0 0.117 0 0 0.37 0.42

CXCL8 IL1A 9606.ENSP00000306512 9606.ENSP00000263339 0 0 0 0 0.234 0 0.9 0.872 0.989

CXCL8 IL1B 9606.ENSP00000306512 9606.ENSP00000263341 0 0 0 0 0.679 0 0.9 0.948 0.998

CXCL8 SELP 9606.ENSP00000306512 9606.ENSP00000263686 0 0 0 0 0.049 0 0 0.719 0.722

CXCL8 FGF2 9606.ENSP00000306512 9606.ENSP00000264498 0 0 0 0 0 0 0 0.775 0.775

CXCL8 ICAM1 9606.ENSP00000306512 9606.ENSP00000264832 0 0 0 0 0.214 0 0 0.858 0.883

CXCL8 EGF 9606.ENSP00000306512 9606.ENSP00000265171 0 0 0 0 0 0 0 0.725 0.725

CXCL8 TP53 9606.ENSP00000306512 9606.ENSP00000269305 0 0 0 0 0 0.128 0 0.663 0.693

CXCL8 ERBB2 9606.ENSP00000306512 9606.ENSP00000269571 0 0 0 0 0 0.27 0 0.627 0.716

CXCL8 REN 9606.ENSP00000306512 9606.ENSP00000272190 0 0 0 0 0 0 0 0.425 0.424

CXCL8 EGFR 9606.ENSP00000306512 9606.ENSP00000275493 0 0 0 0 0 0 0 0.654 0.654

CXCL8 PPARG 9606.ENSP00000306512 9606.ENSP00000287820 0 0 0 0 0.056 0 0 0.631 0.636

CXCL8 KIT 9606.ENSP00000306512 9606.ENSP00000288135 0 0 0 0 0 0 0 0.504 0.504

CXCL8 NCF1 9606.ENSP00000306512 9606.ENSP00000289473 0 0 0 0 0.08 0 0 0.395 0.419

CXCL8 VCAM1 9606.ENSP00000306512 9606.ENSP00000294728 0 0 0 0 0.062 0 0 0.817 0.821

CXCL8 PF4 9606.ENSP00000306512 9606.ENSP00000296029 0 0 0 0.818 0.062 0.498 0.5 0.951 0.788

CXCL8 TLR3 9606.ENSP00000306512 9606.ENSP00000296795 0 0 0 0 0.056 0 0 0.78 0.784

CXCL8 NOS3 9606.ENSP00000306512 9606.ENSP00000297494 0 0 0 0 0.053 0 0 0.622 0.627

CXCL8 HPRT1 9606.ENSP00000306512 9606.ENSP00000298556 0 0 0 0 0 0 0 0.434 0.434

CXCL8 MMP3 9606.ENSP00000306512 9606.ENSP00000299855 0 0 0 0 0.107 0 0 0.714 0.734

CXCL8 NOD2 9606.ENSP00000306512 9606.ENSP00000300589 0 0 0 0 0.096 0 0 0.661 0.681

CXCL8 IGF1 9606.ENSP00000306512 9606.ENSP00000302665 0 0 0 0 0 0 0 0.656 0.656

CXCL8 IL13 9606.ENSP00000306512 9606.ENSP00000304915 0 0 0 0 0 0 0.9 0.861 0.985

CXCL8 FAS 9606.ENSP00000306512 9606.ENSP00000347979 0 0 0 0 0.066 0 0 0.456 0.47

CXCL8 VDR 9606.ENSP00000306512 9606.ENSP00000447173 0 0 0 0 0.076 0 0 0.451 0.471

CXCL8 CYCS 9606.ENSP00000306512 9606.ENSP00000307786 0 0 0 0 0 0 0 0.501 0.501

CXCL8 MTOR 9606.ENSP00000306512 9606.ENSP00000354558 0 0 0 0 0 0 0 0.505 0.505

CXCL8 ITGAL 9606.ENSP00000306512 9606.ENSP00000349252 0 0 0 0 0 0 0 0.51 0.51

CXCL8 STAT5A 9606.ENSP00000306512 9606.ENSP00000341208 0 0 0 0 0.108 0 0 0.48 0.516

CXCL8 ESR1 9606.ENSP00000306512 9606.ENSP00000405330 0 0 0 0 0 0 0 0.518 0.518

CXCL8 PTEN 9606.ENSP00000306512 9606.ENSP00000361021 0 0 0 0 0 0 0 0.519 0.519

CXCL8 SOCS1 9606.ENSP00000306512 9606.ENSP00000329418 0 0 0 0 0.084 0 0 0.501 0.523

CXCL8 GPT 9606.ENSP00000306512 9606.ENSP00000378408 0 0 0 0 0 0 0 0.524 0.524

CXCL8 IDO1 9606.ENSP00000306512 9606.ENSP00000430950 0 0 0 0 0.083 0 0 0.503 0.525

CXCL8 S100A9 9606.ENSP00000306512 9606.ENSP00000357727 0 0 0 0 0.164 0 0 0.463 0.532

CXCL8 ITGB2 9606.ENSP00000306512 9606.ENSP00000380948 0 0 0 0 0.099 0 0 0.505 0.535

CXCL8 F2 9606.ENSP00000306512 9606.ENSP00000308541 0 0 0 0 0 0 0 0.535 0.535

CXCL8 FASLG 9606.ENSP00000306512 9606.ENSP00000356694 0 0 0 0 0 0 0 0.562 0.562

CXCL8 TNFRSF1B 9606.ENSP00000306512 9606.ENSP00000365435 0 0 0 0 0.074 0 0 0.562 0.577

CXCL8 NOS2 9606.ENSP00000306512 9606.ENSP00000327251 0 0 0 0 0.053 0 0 0.583 0.589

CXCL8 NGF 9606.ENSP00000306512 9606.ENSP00000358525 0 0 0 0 0.054 0 0 0.594 0.599

CXCL8 THBD 9606.ENSP00000306512 9606.ENSP00000366307 0 0 0 0 0.139 0 0 0.556 0.601

CXCL8 IL17RA 9606.ENSP00000306512 9606.ENSP00000320936 0 0 0 0 0 0 0 0.604 0.604

CXCL8 OCLN 9606.ENSP00000306512 9606.ENSP00000347379 0 0 0 0 0 0 0 0.612 0.612

CXCL8 SPP1 9606.ENSP00000306512 9606.ENSP00000378517 0 0 0 0 0.082 0 0 0.605 0.622

CXCL8 LCN2 9606.ENSP00000306512 9606.ENSP00000362108 0 0 0 0 0.107 0 0 0.603 0.631

CXCL8 FOXP3 9606.ENSP00000306512 9606.ENSP00000365380 0 0 0 0 0 0 0 0.633 0.633

CXCL8 EDN1 9606.ENSP00000306512 9606.ENSP00000368683 0 0 0 0 0.072 0 0 0.634 0.645

CXCL8 STAT1 9606.ENSP00000306512 9606.ENSP00000354394 0 0 0 0 0.053 0 0 0.675 0.679

CXCL8 LEP 9606.ENSP00000306512 9606.ENSP00000312652 0 0 0 0 0 0 0 0.682 0.682

CXCL8 HIF1A 9606.ENSP00000306512 9606.ENSP00000437955 0 0 0 0 0.07 0 0 0.681 0.69

CXCL8 INS 9606.ENSP00000306512 9606.ENSP00000380432 0 0 0 0 0 0 0 0.692 0.692

CXCL8 S100A8 9606.ENSP00000306512 9606.ENSP00000357722 0 0 0 0 0.189 0 0 0.664 0.716

CXCL8 LTA 9606.ENSP00000306512 9606.ENSP00000403495 0 0 0 0 0.061 0 0 0.732 0.737

CXCL8 ELANE 9606.ENSP00000306512 9606.ENSP00000466090 0 0 0 0 0.065 0 0 0.744 0.75

CXCL8 MMP1 9606.ENSP00000306512 9606.ENSP00000322788 0 0 0 0 0.19 0 0 0.715 0.759

CXCL8 MYD88 9606.ENSP00000306512 9606.ENSP00000401399 0 0 0 0 0.076 0 0 0.753 0.762

CXCL8 TNFAIP3 9606.ENSP00000306512 9606.ENSP00000481570 0 0 0 0 0.537 0 0 0.528 0.772

CXCL8 SELE 9606.ENSP00000306512 9606.ENSP00000331736 0 0 0 0 0.049 0 0 0.773 0.775

CXCL8 MMP9 9606.ENSP00000306512 9606.ENSP00000361405 0 0 0 0 0.134 0.213 0 0.825 0.871

CXCL8 VEGFA 9606.ENSP00000306512 9606.ENSP00000478570 0 0 0 0 0.065 0 0 0.875 0.879

CXCL8 IL17A 9606.ENSP00000306512 9606.ENSP00000344192 0 0 0 0 0 0 0 0.891 0.891

CXCL8 TLR4 9606.ENSP00000306512 9606.ENSP00000363089 0 0 0 0 0.117 0 0 0.881 0.891

CXCL8 CXCR4 9606.ENSP00000306512 9606.ENSP00000386884 0 0 0 0 0 0 0.6 0.792 0.913

CXCL8 JUN 9606.ENSP00000306512 9606.ENSP00000360266 0 0 0 0 0.095 0.27 0.8 0.715 0.957

CXCL8 TNF 9606.ENSP00000306512 9606.ENSP00000398698 0 0 0 0 0.158 0 0.9 0.921 0.992

CXCL8 IL10 9606.ENSP00000306512 9606.ENSP00000412237 0 0 0 0 0.062 0 0.9 0.947 0.994

CXCL8 IL6 9606.ENSP00000306512 9606.ENSP00000385675 0 0 0 0 0.581 0 0.9 0.947 0.997

CXCR4 TNFRSF1A 9606.ENSP00000386884 9606.ENSP00000162749 0 0 0 0 0.062 0 0 0.42 0.432

CXCR4 MIF 9606.ENSP00000386884 9606.ENSP00000215754 0 0 0 0 0 0 0 0.98 0.98

CXCR4 NFKBIA 9606.ENSP00000386884 9606.ENSP00000216797 0 0 0 0 0.082 0.062 0 0.423 0.46

CXCR4 MMP2 9606.ENSP00000386884 9606.ENSP00000219070 0 0 0 0 0.062 0.057 0 0.624 0.638

CXCR4 TGFB1 9606.ENSP00000386884 9606.ENSP00000221930 0 0 0 0 0.073 0 0 0.496 0.514

CXCR4 IL2 9606.ENSP00000386884 9606.ENSP00000226730 0 0 0 0 0 0 0 0.658 0.658

CXCR4 KITLG 9606.ENSP00000386884 9606.ENSP00000228280 0 0 0 0 0 0 0 0.646 0.646

CXCR4 IFNG 9606.ENSP00000386884 9606.ENSP00000229135 0 0 0 0 0 0 0 0.567 0.567

CXCR4 IL4 9606.ENSP00000386884 9606.ENSP00000231449 0 0 0 0 0.063 0 0 0.604 0.613

CXCR4 IL1A 9606.ENSP00000386884 9606.ENSP00000263339 0 0 0 0 0 0 0 0.469 0.469

CXCR4 IL1B 9606.ENSP00000386884 9606.ENSP00000263341 0 0 0 0 0.062 0 0 0.654 0.661

CXCR4 SELP 9606.ENSP00000386884 9606.ENSP00000263686 0 0 0 0 0.076 0 0 0.561 0.577

CXCR4 FGF2 9606.ENSP00000386884 9606.ENSP00000264498 0 0 0 0 0 0 0 0.677 0.677

CXCR4 ICAM1 9606.ENSP00000386884 9606.ENSP00000264832 0 0 0 0 0.062 0 0 0.665 0.672

CXCR4 EGF 9606.ENSP00000386884 9606.ENSP00000265171 0 0 0 0 0 0.056 0 0.686 0.692

CXCR4 TP53 9606.ENSP00000386884 9606.ENSP00000269305 0 0 0 0 0 0 0 0.606 0.606

CXCR4 ERBB2 9606.ENSP00000386884 9606.ENSP00000269571 0 0 0 0 0 0.061 0 0.565 0.574

CXCR4 SOD1 9606.ENSP00000386884 9606.ENSP00000270142 0 0 0 0 0 0 0 0.447 0.447

CXCR4 EGFR 9606.ENSP00000386884 9606.ENSP00000275493 0 0 0 0 0 0.061 0 0.912 0.914

CXCR4 PPARG 9606.ENSP00000386884 9606.ENSP00000287820 0 0 0 0 0.062 0.056 0 0.391 0.413

CXCR4 KIT 9606.ENSP00000386884 9606.ENSP00000288135 0 0 0 0 0 0 0 0.733 0.733

CXCR4 VCAM1 9606.ENSP00000386884 9606.ENSP00000294728 0 0 0 0 0.053 0 0 0.747 0.751

CXCR4 PF4 9606.ENSP00000386884 9606.ENSP00000296029 0 0 0 0 0.062 0 0.6 0.517 0.803

CXCR4 TLR3 9606.ENSP00000386884 9606.ENSP00000296795 0 0 0 0 0 0 0 0.424 0.424

CXCR4 NOS3 9606.ENSP00000386884 9606.ENSP00000297494 0 0 0 0 0.055 0 0 0.504 0.511

CXCR4 MMP3 9606.ENSP00000386884 9606.ENSP00000299855 0 0 0 0 0 0.057 0 0.459 0.468

CXCR4 IGF1 9606.ENSP00000386884 9606.ENSP00000302665 0 0 0 0 0 0 0 0.729 0.729

CXCR4 IL13 9606.ENSP00000386884 9606.ENSP00000304915 0 0 0 0 0 0 0 0.47 0.47

CXCR4 F2 9606.ENSP00000386884 9606.ENSP00000308541 0 0 0 0 0 0.097 0.9 0.253 0.926

CXCR4 MMP1 9606.ENSP00000386884 9606.ENSP00000322788 0 0 0 0 0 0.057 0 0.473 0.482

CXCR4 SOCS1 9606.ENSP00000386884 9606.ENSP00000329418 0 0 0 0 0.062 0.417 0 0.485 0.693

CXCR4 SELE 9606.ENSP00000386884 9606.ENSP00000331736 0 0 0 0 0 0 0 0.593 0.593

CXCR4 STAT5A 9606.ENSP00000386884 9606.ENSP00000341208 0 0 0 0 0.088 0.056 0.9 0.415 0.942

CXCR4 USP7 9606.ENSP00000386884 9606.ENSP00000343535 0 0 0 0 0 0 0 0.428 0.428

CXCR4 IL17A 9606.ENSP00000386884 9606.ENSP00000344192 0 0 0 0 0 0 0 0.561 0.561

CXCR4 FAS 9606.ENSP00000386884 9606.ENSP00000347979 0 0 0 0 0.062 0 0 0.503 0.514

CXCR4 ITGAL 9606.ENSP00000386884 9606.ENSP00000349252 0 0 0 0 0.144 0 0 0.524 0.575

CXCR4 VCP 9606.ENSP00000386884 9606.ENSP00000351777 0 0 0 0 0 0.058 0 0.572 0.58

CXCR4 STAT1 9606.ENSP00000386884 9606.ENSP00000354394 0 0 0 0 0 0.225 0.9 0.562 0.963

CXCR4 MTOR 9606.ENSP00000386884 9606.ENSP00000354558 0 0 0 0 0 0.056 0 0.466 0.475

CXCR4 FASLG 9606.ENSP00000386884 9606.ENSP00000356694 0 0 0 0 0 0 0 0.419 0.418

CXCR4 NGF 9606.ENSP00000386884 9606.ENSP00000358525 0 0 0 0 0 0 0 0.403 0.403

CXCR4 JUN 9606.ENSP00000386884 9606.ENSP00000360266 0 0 0 0 0 0 0 0.501 0.501

CXCR4 PTEN 9606.ENSP00000386884 9606.ENSP00000361021 0 0 0 0 0 0.058 0 0.525 0.533

CXCR4 MMP9 9606.ENSP00000386884 9606.ENSP00000361405 0 0 0 0 0.076 0.057 0 0.708 0.723

CXCR4 TLR4 9606.ENSP00000386884 9606.ENSP00000363089 0 0 0 0 0.085 0 0 0.763 0.774

CXCR4 GDF5 9606.ENSP00000386884 9606.ENSP00000363492 0 0 0 0 0 0 0 0.851 0.851

CXCR4 FOXP3 9606.ENSP00000386884 9606.ENSP00000365380 0 0 0 0 0 0.057 0 0.594 0.6

CXCR4 EDN1 9606.ENSP00000386884 9606.ENSP00000368683 0 0 0 0 0 0 0.9 0.419 0.939

CXCR4 SPP1 9606.ENSP00000386884 9606.ENSP00000378517 0 0 0 0 0 0 0 0.715 0.715

CXCR4 INS 9606.ENSP00000386884 9606.ENSP00000380432 0 0 0 0 0 0 0 0.527 0.527

CXCR4 ITGB2 9606.ENSP00000386884 9606.ENSP00000380948 0 0 0 0 0.162 0 0 0.482 0.547

CXCR4 IL6 9606.ENSP00000386884 9606.ENSP00000385675 0 0 0 0 0 0 0 0.698 0.698

CXCR4 LTA 9606.ENSP00000386884 9606.ENSP00000403495 0 0 0 0 0.074 0 0 0.458 0.477

CXCR4 ESR1 9606.ENSP00000386884 9606.ENSP00000405330 0 0 0 0 0 0.056 0 0.47 0.478

CXCR4 MYD88 9606.ENSP00000386884 9606.ENSP00000401399 0 0 0 0 0 0 0 0.504 0.504

CXCR4 ELANE 9606.ENSP00000386884 9606.ENSP00000466090 0 0 0 0 0.067 0.263 0 0.452 0.59

CXCR4 IL10 9606.ENSP00000386884 9606.ENSP00000412237 0 0 0 0 0 0 0 0.666 0.666

CXCR4 TNF 9606.ENSP00000386884 9606.ENSP00000398698 0 0 0 0 0.079 0 0 0.749 0.76

CXCR4 HIF1A 9606.ENSP00000386884 9606.ENSP00000437955 0 0 0 0 0 0.273 0 0.71 0.78

CXCR4 VEGFA 9606.ENSP00000386884 9606.ENSP00000478570 0 0 0 0 0 0 0 0.917 0.917

CYCS TNFRSF1A 9606.ENSP00000307786 9606.ENSP00000162749 0 0 0 0 0 0 0 0.66 0.66

CYCS HMOX1 9606.ENSP00000307786 9606.ENSP00000216117 0 0 0 0 0.064 0 0 0.708 0.716

CYCS NFKBIA 9606.ENSP00000307786 9606.ENSP00000216797 0 0 0 0 0.062 0 0 0.566 0.575

CYCS MMP2 9606.ENSP00000307786 9606.ENSP00000219070 0 0 0 0 0 0 0 0.504 0.504

CYCS DHODH 9606.ENSP00000307786 9606.ENSP00000219240 0 0 0 0 0.065 0 0 0.672 0.681

CYCS MPO 9606.ENSP00000307786 9606.ENSP00000225275 0 0 0 0 0 0 0 0.425 0.425

CYCS IL2 9606.ENSP00000307786 9606.ENSP00000226730 0 0 0 0 0 0 0 0.47 0.47

CYCS HSPB1 9606.ENSP00000307786 9606.ENSP00000248553 0 0 0 0 0 0.462 0.9 0.893 0.993

CYCS MDM2 9606.ENSP00000307786 9606.ENSP00000258149 0 0 0 0 0 0 0 0.6 0.6

CYCS IL1B 9606.ENSP00000307786 9606.ENSP00000263341 0 0 0 0 0 0 0 0.636 0.636

CYCS FGF2 9606.ENSP00000307786 9606.ENSP00000264498 0 0 0 0 0 0 0 0.454 0.454

CYCS ICAM1 9606.ENSP00000307786 9606.ENSP00000264832 0 0 0 0 0 0 0 0.458 0.457

CYCS EGF 9606.ENSP00000307786 9606.ENSP00000265171 0 0 0 0 0 0 0 0.517 0.517

CYCS TP53 9606.ENSP00000307786 9606.ENSP00000269305 0 0 0 0 0 0 0 0.859 0.859

CYCS ERBB2 9606.ENSP00000307786 9606.ENSP00000269571 0 0 0 0 0 0 0 0.468 0.468

CYCS SOD1 9606.ENSP00000307786 9606.ENSP00000270142 0 0 0 0 0.126 0 0 0.588 0.625

CYCS EGFR 9606.ENSP00000307786 9606.ENSP00000275493 0 0 0 0 0 0 0 0.556 0.556

CYCS PPARG 9606.ENSP00000307786 9606.ENSP00000287820 0 0 0 0 0 0 0 0.56 0.56

CYCS NCF1 9606.ENSP00000307786 9606.ENSP00000289473 0 0 0 0 0 0 0 0.478 0.478

CYCS NOS3 9606.ENSP00000307786 9606.ENSP00000297494 0 0 0 0 0.064 0 0 0.629 0.638

CYCS HPRT1 9606.ENSP00000307786 9606.ENSP00000298556 0 0 0 0 0.126 0 0 0.35 0.407

CYCS FADD 9606.ENSP00000307786 9606.ENSP00000301838 0 0 0 0 0.045 0 0 0.759 0.76

CYCS IGF1 9606.ENSP00000307786 9606.ENSP00000302665 0 0 0 0 0 0 0 0.51 0.51

CYCS ITPR1 9606.ENSP00000307786 9606.ENSP00000306253 0 0 0 0 0 0.116 0.36 0.987 0.992

CYCS TOP1 9606.ENSP00000307786 9606.ENSP00000354522 0 0 0 0 0.062 0 0 0.395 0.408

CYCS CYP19A1 9606.ENSP00000307786 9606.ENSP00000379683 0 0 0 0 0.062 0.142 0 0.325 0.41

CYCS TH 9606.ENSP00000307786 9606.ENSP00000370571 0 0 0 0 0 0 0 0.417 0.416

CYCS NOS2 9606.ENSP00000307786 9606.ENSP00000327251 0 0 0 0 0.064 0 0 0.422 0.436

CYCS CYP1A2 9606.ENSP00000307786 9606.ENSP00000342007 0 0 0 0 0 0.142 0 0.376 0.442

CYCS CYP3A4 9606.ENSP00000307786 9606.ENSP00000337915 0 0 0 0 0 0.247 0 0.317 0.464

CYCS IL10 9606.ENSP00000307786 9606.ENSP00000412237 0 0 0 0 0 0 0 0.468 0.468

CYCS GPT 9606.ENSP00000307786 9606.ENSP00000378408 0 0 0 0 0 0 0 0.469 0.469

CYCS TLR4 9606.ENSP00000307786 9606.ENSP00000363089 0 0 0 0 0.062 0 0 0.469 0.48

CYCS P4HB 9606.ENSP00000307786 9606.ENSP00000327801 0 0 0 0 0.062 0.157 0 0.434 0.513

CYCS LTA 9606.ENSP00000307786 9606.ENSP00000403495 0 0 0 0 0 0 0 0.515 0.515

CYCS ESR1 9606.ENSP00000307786 9606.ENSP00000405330 0 0 0 0 0 0 0 0.517 0.517

CYCS MMP9 9606.ENSP00000307786 9606.ENSP00000361405 0 0 0 0 0 0 0 0.57 0.57

CYCS PTEN 9606.ENSP00000307786 9606.ENSP00000361021 0 0 0 0 0.062 0 0 0.593 0.602

CYCS PRKCD 9606.ENSP00000307786 9606.ENSP00000378217 0 0 0 0 0 0 0 0.607 0.607

CYCS IL6 9606.ENSP00000307786 9606.ENSP00000385675 0 0 0 0 0 0 0 0.61 0.61

CYCS HIF1A 9606.ENSP00000307786 9606.ENSP00000437955 0 0 0 0 0 0 0 0.612 0.612

CYCS MTOR 9606.ENSP00000307786 9606.ENSP00000354558 0 0 0 0 0 0 0 0.615 0.615

CYCS VEGFA 9606.ENSP00000307786 9606.ENSP00000478570 0 0 0 0 0 0 0 0.634 0.634

CYCS NOS1 9606.ENSP00000307786 9606.ENSP00000477999 0 0 0 0 0.064 0 0 0.64 0.649

CYCS INS 9606.ENSP00000307786 9606.ENSP00000380432 0 0 0 0 0.062 0 0 0.663 0.671

CYCS FAS 9606.ENSP00000307786 9606.ENSP00000347979 0 0 0 0 0 0 0 0.712 0.712

CYCS HSPA5 9606.ENSP00000307786 9606.ENSP00000324173 0 0 0 0 0.088 0 0 0.7 0.715

CYCS JUN 9606.ENSP00000307786 9606.ENSP00000360266 0 0 0 0 0 0 0 0.73 0.73

CYCS PARP1 9606.ENSP00000307786 9606.ENSP00000355759 0 0 0 0 0.062 0 0 0.73 0.735

CYCS FASLG 9606.ENSP00000307786 9606.ENSP00000356694 0 0 0 0 0 0 0 0.739 0.739

CYCS TNF 9606.ENSP00000307786 9606.ENSP00000398698 0 0 0 0 0 0 0 0.739 0.739

CYCS ITPR3 9606.ENSP00000307786 9606.ENSP00000363435 0 0 0 0 0 0.116 0 0.756 0.775

CYCS SNCA 9606.ENSP00000307786 9606.ENSP00000338345 0 0 0 0 0 0.203 0 0.763 0.803

CYCS NGF 9606.ENSP00000307786 9606.ENSP00000358525 0 0 0 0 0 0 0.9 0.563 0.954

CYP19A1 NR3C1 9606.ENSP00000379683 9606.ENSP00000231509 0 0 0 0 0 0.073 0 0.415 0.434

CYP19A1 EGF 9606.ENSP00000379683 9606.ENSP00000265171 0 0 0 0 0.064 0 0 0.506 0.517

CYP19A1 ERBB3 9606.ENSP00000379683 9606.ENSP00000267101 0 0 0 0 0 0 0 0.406 0.406

CYP19A1 TP53 9606.ENSP00000379683 9606.ENSP00000269305 0 0 0 0 0 0.058 0 0.557 0.564

CYP19A1 ERBB2 9606.ENSP00000379683 9606.ENSP00000269571 0 0 0 0 0 0 0 0.802 0.802

CYP19A1 SRD5A1 9606.ENSP00000379683 9606.ENSP00000274192 0 0 0 0 0 0 0.9 0.709 0.969

CYP19A1 EGFR 9606.ENSP00000379683 9606.ENSP00000275493 0 0 0 0 0 0 0 0.618 0.618

CYP19A1 PPARG 9606.ENSP00000379683 9606.ENSP00000287820 0 0 0 0 0 0.073 0 0.504 0.52

CYP19A1 HMGCR 9606.ENSP00000379683 9606.ENSP00000287936 0 0 0 0 0.065 0 0 0.565 0.576

CYP19A1 IGF1 9606.ENSP00000379683 9606.ENSP00000302665 0 0 0 0 0.049 0 0 0.667 0.67

CYP19A1 LEP 9606.ENSP00000379683 9606.ENSP00000312652 0 0 0 0 0 0 0 0.667 0.667

CYP19A1 CYP3A4 9606.ENSP00000379683 9606.ENSP00000337915 0 0 0.419 0.577 0 0.139 0.9 0.626 0.944

CYP19A1 CYP1A2 9606.ENSP00000379683 9606.ENSP00000342007 0 0 0.411 0.56 0 0.09 0 0.566 0.426

CYP19A1 MTOR 9606.ENSP00000379683 9606.ENSP00000354558 0 0 0 0 0 0 0 0.519 0.519

CYP19A1 HSD11B1 9606.ENSP00000379683 9606.ENSP00000355995 0 0 0 0 0 0.056 0 0.469 0.477

CYP19A1 JUN 9606.ENSP00000379683 9606.ENSP00000360266 0 0 0 0 0 0.061 0 0.391 0.403

CYP19A1 PTEN 9606.ENSP00000379683 9606.ENSP00000361021 0 0 0 0 0.062 0 0 0.503 0.513

CYP19A1 SHBG 9606.ENSP00000379683 9606.ENSP00000369816 0 0 0 0 0 0 0 0.733 0.733

CYP19A1 IL10 9606.ENSP00000379683 9606.ENSP00000412237 0 0 0 0 0 0 0 0.428 0.428

CYP19A1 CYP1B1 9606.ENSP00000379683 9606.ENSP00000478561 0 0 0.365 0.555 0 0.09 0 0.694 0.461

CYP19A1 TNF 9606.ENSP00000379683 9606.ENSP00000398698 0 0 0 0 0 0 0 0.49 0.49

CYP19A1 IL6 9606.ENSP00000379683 9606.ENSP00000385675 0 0 0 0 0 0 0 0.505 0.505

CYP19A1 VEGFA 9606.ENSP00000379683 9606.ENSP00000478570 0 0 0 0 0 0 0 0.518 0.518

CYP19A1 INS 9606.ENSP00000379683 9606.ENSP00000380432 0 0 0 0 0.062 0 0 0.627 0.635

CYP19A1 ESR1 9606.ENSP00000379683 9606.ENSP00000405330 0 0 0 0 0 0.073 0 0.919 0.922

CYP1A2 PON1 9606.ENSP00000342007 9606.ENSP00000222381 0 0 0 0 0 0 0 0.459 0.459

CYP1A2 CYP2C9 9606.ENSP00000342007 9606.ENSP00000260682 0 0 0.444 0.772 0.077 0 0.9 0.916 0.931

CYP1A2 SLC6A4 9606.ENSP00000342007 9606.ENSP00000261707 0 0 0 0 0 0 0 0.46 0.459

CYP1A2 GSTM1 9606.ENSP00000342007 9606.ENSP00000311469 0 0 0 0 0 0.057 0.65 0.635 0.869

CYP1A2 CYP3A4 9606.ENSP00000342007 9606.ENSP00000337915 0 0 0.442 0.604 0.139 0.077 0.9 0.927 0.954

CYP1A2 ESR1 9606.ENSP00000342007 9606.ENSP00000405330 0 0 0 0 0 0.073 0 0.391 0.411

CYP1A2 HTR2A 9606.ENSP00000342007 9606.ENSP00000437737 0 0 0 0 0 0 0 0.494 0.493

CYP1A2 GPT 9606.ENSP00000342007 9606.ENSP00000378408 0 0 0 0 0.063 0 0 0.531 0.542

CYP1A2 CYP1B1 9606.ENSP00000342007 9606.ENSP00000478561 0 0 0.418 0.882 0 0 0.8 0.858 0.828

CYP1A2 IDO1 9606.ENSP00000342007 9606.ENSP00000430950 0 0 0 0 0 0 0.9 0.073 0.903

CYP1A2 XDH 9606.ENSP00000342007 9606.ENSP00000368727 0 0 0 0 0.051 0 0.9 0.134 0.91

CYP1A2 NR1I3 9606.ENSP00000342007 9606.ENSP00000356959 0 0 0 0 0.076 0.073 0.9 0.66 0.967

CYP1B1 HMOX1 9606.ENSP00000478561 9606.ENSP00000216117 0 0 0 0 0.101 0 0 0.415 0.452

CYP1B1 TP53 9606.ENSP00000478561 9606.ENSP00000269305 0 0 0 0 0 0.058 0 0.468 0.477

CYP1B1 GSTM1 9606.ENSP00000478561 9606.ENSP00000311469 0 0 0 0 0.053 0.057 0.65 0.681 0.886

CYP1B1 CYP3A4 9606.ENSP00000478561 9606.ENSP00000337915 0 0 0.403 0.597 0.085 0.104 0 0.766 0.496

CYP1B1 NR1I3 9606.ENSP00000478561 9606.ENSP00000356959 0 0 0 0 0.062 0.073 0 0.46 0.49

CYP1B1 SHBG 9606.ENSP00000478561 9606.ENSP00000369816 0 0 0 0 0.062 0 0 0.392 0.405

CYP1B1 IL6 9606.ENSP00000478561 9606.ENSP00000385675 0 0 0 0 0.086 0 0 0.373 0.402

CYP1B1 ESR1 9606.ENSP00000478561 9606.ENSP00000405330 0 0 0 0 0.073 0.073 0 0.655 0.678

CYP1B1 IDO1 9606.ENSP00000478561 9606.ENSP00000430950 0 0 0 0 0 0 0.9 0.3 0.927

CYP2C9 PON1 9606.ENSP00000260682 9606.ENSP00000222381 0 0 0 0 0.081 0 0 0.393 0.418

CYP2C9 MPO 9606.ENSP00000260682 9606.ENSP00000225275 0 0 0 0 0.053 0 0 0.408 0.415

CYP2C9 INS 9606.ENSP00000260682 9606.ENSP00000380432 0 0 0 0 0 0 0 0.401 0.4

CYP2C9 SLC6A4 9606.ENSP00000260682 9606.ENSP00000261707 0 0 0 0 0 0 0 0.402 0.402

CYP2C9 HMGCR 9606.ENSP00000260682 9606.ENSP00000287936 0 0 0 0 0.064 0 0 0.396 0.411

CYP2C9 GPT 9606.ENSP00000260682 9606.ENSP00000378408 0 0 0 0 0.063 0 0 0.43 0.443

CYP2C9 ESR1 9606.ENSP00000260682 9606.ENSP00000405330 0 0 0 0 0.053 0.073 0 0.513 0.535

CYP2C9 F2 9606.ENSP00000260682 9606.ENSP00000308541 0 0 0 0 0.087 0.061 0 0.596 0.623

CYP2C9 NR1I3 9606.ENSP00000260682 9606.ENSP00000356959 0 0 0 0 0.062 0.073 0 0.702 0.718

CYP2C9 GSTM1 9606.ENSP00000260682 9606.ENSP00000311469 0 0 0 0 0 0.057 0.65 0.518 0.827

CYP2C9 CYP3A4 9606.ENSP00000260682 9606.ENSP00000337915 0 0 0.444 0.648 0.247 0.346 0.9 0.927 0.969

CYP3A4 PON1 9606.ENSP00000337915 9606.ENSP00000222381 0 0 0 0 0.098 0 0 0.451 0.483

CYP3A4 NR3C1 9606.ENSP00000337915 9606.ENSP00000231509 0 0 0 0 0 0.073 0 0.582 0.596

CYP3A4 TP53 9606.ENSP00000337915 9606.ENSP00000269305 0 0 0 0 0 0.058 0 0.417 0.427

CYP3A4 REN 9606.ENSP00000337915 9606.ENSP00000272190 0 0 0 0 0.064 0 0 0.391 0.405

CYP3A4 EGFR 9606.ENSP00000337915 9606.ENSP00000275493 0 0 0 0 0 0 0 0.545 0.545

CYP3A4 PPARG 9606.ENSP00000337915 9606.ENSP00000287820 0 0 0 0 0.053 0.073 0 0.413 0.439

CYP3A4 HMGCR 9606.ENSP00000337915 9606.ENSP00000287936 0 0 0 0 0.064 0 0 0.502 0.514

CYP3A4 F2 9606.ENSP00000337915 9606.ENSP00000308541 0 0 0 0 0.087 0.061 0 0.502 0.535

CYP3A4 GSTM1 9606.ENSP00000337915 9606.ENSP00000311469 0 0 0 0 0 0.056 0.65 0.561 0.842

CYP3A4 VEGFA 9606.ENSP00000337915 9606.ENSP00000478570 0 0 0 0 0 0 0 0.404 0.404

CYP3A4 MTOR 9606.ENSP00000337915 9606.ENSP00000354558 0 0 0 0 0 0 0 0.406 0.406

CYP3A4 IL6 9606.ENSP00000337915 9606.ENSP00000385675 0 0 0 0 0 0 0 0.457 0.457

CYP3A4 HTR2A 9606.ENSP00000337915 9606.ENSP00000437737 0 0 0 0 0 0 0 0.503 0.503

CYP3A4 INS 9606.ENSP00000337915 9606.ENSP00000380432 0 0 0 0 0 0 0 0.519 0.519

CYP3A4 VDR 9606.ENSP00000337915 9606.ENSP00000447173 0 0 0 0 0 0.073 0 0.603 0.616

CYP3A4 ESR1 9606.ENSP00000337915 9606.ENSP00000405330 0 0 0 0 0.049 0.073 0 0.621 0.637

CYP3A4 GPT 9606.ENSP00000337915 9606.ENSP00000378408 0 0 0 0 0.09 0 0 0.656 0.674

CYP3A4 NR1I3 9606.ENSP00000337915 9606.ENSP00000356959 0 0 0 0 0.062 0.073 0 0.826 0.836

CYP3A4 HSD11B1 9606.ENSP00000337915 9606.ENSP00000355995 0 0 0 0 0.111 0.056 0.9 0.326 0.935

DHODH OGDH 9606.ENSP00000219240 9606.ENSP00000222673 0 0 0 0 0.066 0.271 0 0.235 0.434

DNAJC3 HSPA5 9606.ENSP00000473631 9606.ENSP00000324173 0.042 0 0 0 0.841 0.378 0 0.861 0.985

DNAJC3 P4HB 9606.ENSP00000473631 9606.ENSP00000327801 0 0 0 0 0.52 0.33 0 0.539 0.839

DNAJC3 FURIN 9606.ENSP00000473631 9606.ENSP00000483552 0 0 0 0 0.414 0 0 0.048 0.418

DNMT1 NR3C1 9606.ENSP00000352516 9606.ENSP00000231509 0 0 0 0 0 0.056 0 0.478 0.487

DNMT1 MDM2 9606.ENSP00000352516 9606.ENSP00000258149 0 0 0 0 0 0 0 0.408 0.408

DNMT1 TP53 9606.ENSP00000352516 9606.ENSP00000269305 0 0 0 0 0.072 0.486 0 0.986 0.993

DNMT1 ERBB2 9606.ENSP00000352516 9606.ENSP00000269571 0 0 0 0 0 0 0 0.467 0.467

DNMT1 EGFR 9606.ENSP00000352516 9606.ENSP00000275493 0 0 0 0 0 0 0 0.476 0.476

DNMT1 PPARG 9606.ENSP00000352516 9606.ENSP00000287820 0 0 0 0 0 0.056 0 0.82 0.823

DNMT1 SNCA 9606.ENSP00000352516 9606.ENSP00000338345 0 0 0 0 0 0 0 0.605 0.605

DNMT1 USP7 9606.ENSP00000352516 9606.ENSP00000343535 0 0 0 0 0.063 0.977 0 0.988 0.999

DNMT1 E2F1 9606.ENSP00000352516 9606.ENSP00000345571 0 0 0 0 0.127 0.27 0.9 0.345 0.952

DNMT1 TNF 9606.ENSP00000352516 9606.ENSP00000398698 0 0 0 0 0 0 0 0.449 0.449

DNMT1 VEGFA 9606.ENSP00000352516 9606.ENSP00000478570 0 0 0 0 0 0 0 0.452 0.452

DNMT1 INS 9606.ENSP00000352516 9606.ENSP00000380432 0 0 0 0 0 0 0 0.454 0.454

DNMT1 POLD1 9606.ENSP00000352516 9606.ENSP00000406046 0.043 0 0 0 0.38 0 0 0.216 0.495

DNMT1 JUN 9606.ENSP00000352516 9606.ENSP00000360266 0 0 0 0 0 0.129 0 0.452 0.502

DNMT1 IL6 9606.ENSP00000352516 9606.ENSP00000385675 0 0 0 0 0 0 0 0.504 0.504

DNMT1 HIF1A 9606.ENSP00000352516 9606.ENSP00000437955 0 0 0 0 0 0 0 0.538 0.538

DNMT1 PTEN 9606.ENSP00000352516 9606.ENSP00000361021 0 0 0 0 0.062 0 0 0.564 0.573

DNMT1 FOXP3 9606.ENSP00000352516 9606.ENSP00000365380 0 0 0 0 0 0.373 0 0.427 0.625

DNMT1 ESR1 9606.ENSP00000352516 9606.ENSP00000405330 0 0 0 0 0 0.056 0 0.648 0.654

DNMT1 PARP1 9606.ENSP00000352516 9606.ENSP00000355759 0 0 0 0 0.231 0.501 0 0.815 0.923

DNMT1 HELLS 9606.ENSP00000352516 9606.ENSP00000377601 0.053 0 0 0 0.208 0.27 0 0.901 0.938

DNMT1 PCNA 9606.ENSP00000352516 9606.ENSP00000368458 0.198 0 0 0 0.355 0.975 0 0 0.986

DNMT1 HDAC1 9606.ENSP00000352516 9606.ENSP00000362649 0 0 0 0 0.188 0.903 0.9 0.993 0.999

E2F1 NFKB1 9606.ENSP00000345571 9606.ENSP00000226574 0 0 0 0 0.054 0.395 0 0.193 0.498

E2F1 MDM2 9606.ENSP00000345571 9606.ENSP00000258149 0 0 0 0 0 0.486 0.9 0.719 0.984

E2F1 LEF1 9606.ENSP00000345571 9606.ENSP00000265165 0 0 0 0 0 0.297 0 0.46 0.605

E2F1 TP53 9606.ENSP00000345571 9606.ENSP00000269305 0 0 0 0 0 0.678 0 0.925 0.975

E2F1 ERBB2 9606.ENSP00000345571 9606.ENSP00000269571 0 0 0 0 0.062 0 0 0.454 0.466

E2F1 EGFR 9606.ENSP00000345571 9606.ENSP00000275493 0 0 0 0 0.062 0 0 0.498 0.509

E2F1 JUN 9606.ENSP00000345571 9606.ENSP00000360266 0 0 0 0 0 0 0 0.403 0.403

E2F1 HELLS 9606.ENSP00000345571 9606.ENSP00000377601 0 0 0 0 0.188 0.115 0 0.261 0.422

E2F1 MTOR 9606.ENSP00000345571 9606.ENSP00000354558 0 0 0 0 0.063 0.122 0 0.407 0.469

E2F1 PTEN 9606.ENSP00000345571 9606.ENSP00000361021 0 0 0 0 0.062 0 0 0.514 0.525

E2F1 ESR1 9606.ENSP00000345571 9606.ENSP00000405330 0 0 0 0 0.062 0.281 0 0.406 0.564

E2F1 HIF1A 9606.ENSP00000345571 9606.ENSP00000437955 0 0 0 0 0 0.057 0 0.627 0.634

E2F1 PARP1 9606.ENSP00000345571 9606.ENSP00000355759 0 0 0 0 0.097 0.501 0 0.445 0.728

E2F1 NGF 9606.ENSP00000345571 9606.ENSP00000358525 0 0 0 0 0 0 0.9 0.134 0.909

E2F1 PCNA 9606.ENSP00000345571 9606.ENSP00000368458 0 0 0 0 0.173 0 0.9 0.396 0.945

E2F1 HDAC1 9606.ENSP00000345571 9606.ENSP00000362649 0 0 0 0 0.112 0.773 0.9 0.67 0.992

EDA LEF1 9606.ENSP00000363680 9606.ENSP00000265165 0 0 0 0 0 0.227 0 0.366 0.489

EDA TLR4 9606.ENSP00000363680 9606.ENSP00000363089 0 0 0 0 0 0.105 0 0.421 0.459

EDA TNF 9606.ENSP00000363680 9606.ENSP00000398698 0 0 0 0 0 0 0 0.409 0.409

EDA FURIN 9606.ENSP00000363680 9606.ENSP00000483552 0 0 0 0 0 0.213 0 0.51 0.598

EDN1 HMOX1 9606.ENSP00000368683 9606.ENSP00000216117 0 0 0 0 0 0 0 0.603 0.603

EDN1 NFKBIA 9606.ENSP00000368683 9606.ENSP00000216797 0 0 0 0 0.062 0 0 0.403 0.416

EDN1 MMP2 9606.ENSP00000368683 9606.ENSP00000219070 0 0 0 0 0.062 0 0.8 0.604 0.919

EDN1 TGFB1 9606.ENSP00000368683 9606.ENSP00000221930 0 0 0 0 0 0 0 0.74 0.74

EDN1 MPO 9606.ENSP00000368683 9606.ENSP00000225275 0 0 0 0 0 0 0 0.472 0.472

EDN1 KITLG 9606.ENSP00000368683 9606.ENSP00000228280 0 0 0 0 0.069 0 0 0.574 0.587

EDN1 IFNG 9606.ENSP00000368683 9606.ENSP00000229135 0 0 0 0 0 0 0 0.459 0.459

EDN1 IL4 9606.ENSP00000368683 9606.ENSP00000231449 0 0 0 0 0.063 0 0 0.427 0.441

EDN1 IL1A 9606.ENSP00000368683 9606.ENSP00000263339 0 0 0 0 0.086 0 0 0.46 0.485

EDN1 IL1B 9606.ENSP00000368683 9606.ENSP00000263341 0 0 0 0 0.062 0 0 0.693 0.7

EDN1 SELP 9606.ENSP00000368683 9606.ENSP00000263686 0 0 0 0 0 0 0 0.601 0.601

EDN1 FGF2 9606.ENSP00000368683 9606.ENSP00000264498 0 0 0 0 0 0 0 0.67 0.671

EDN1 ICAM1 9606.ENSP00000368683 9606.ENSP00000264832 0 0 0 0 0.065 0 0 0.689 0.697

EDN1 EGF 9606.ENSP00000368683 9606.ENSP00000265171 0 0 0 0 0 0 0.9 0.645 0.963

EDN1 TP53 9606.ENSP00000368683 9606.ENSP00000269305 0 0 0 0 0 0 0 0.457 0.457

EDN1 REN 9606.ENSP00000368683 9606.ENSP00000272190 0 0 0 0 0 0 0 0.895 0.895

EDN1 EGFR 9606.ENSP00000368683 9606.ENSP00000275493 0 0 0 0 0.098 0 0.9 0.468 0.947

EDN1 PPARG 9606.ENSP00000368683 9606.ENSP00000287820 0 0 0 0 0 0 0 0.652 0.652

EDN1 NCF1 9606.ENSP00000368683 9606.ENSP00000289473 0 0 0 0 0 0 0 0.499 0.499

EDN1 VCAM1 9606.ENSP00000368683 9606.ENSP00000294728 0 0 0 0 0 0 0 0.713 0.713

EDN1 NOS3 9606.ENSP00000368683 9606.ENSP00000297494 0 0 0 0 0 0 0 0.863 0.863

EDN1 MMP3 9606.ENSP00000368683 9606.ENSP00000299855 0 0 0 0 0 0 0 0.474 0.474

EDN1 IGF1 9606.ENSP00000368683 9606.ENSP00000302665 0 0 0 0 0 0 0 0.644 0.644

EDN1 ITPR1 9606.ENSP00000368683 9606.ENSP00000306253 0 0 0 0 0 0 0 0.418 0.418

EDN1 F2 9606.ENSP00000368683 9606.ENSP00000308541 0 0 0 0 0 0 0.9 0.531 0.951

EDN1 LEP 9606.ENSP00000368683 9606.ENSP00000312652 0 0 0 0 0 0 0 0.571 0.571

EDN1 EDNRA 9606.ENSP00000368683 9606.ENSP00000315011 0 0 0 0 0 0.678 0.9 0.992 0.999

EDN1 MMP1 9606.ENSP00000368683 9606.ENSP00000322788 0 0 0 0 0.069 0 0 0.47 0.486

EDN1 NOS2 9606.ENSP00000368683 9606.ENSP00000327251 0 0 0 0 0.049 0 0 0.476 0.48

EDN1 SOCS1 9606.ENSP00000368683 9606.ENSP00000329418 0 0 0 0 0 0 0 0.435 0.435

EDN1 SELE 9606.ENSP00000368683 9606.ENSP00000331736 0 0 0 0 0 0 0 0.681 0.681

EDN1 PLCB1 9606.ENSP00000368683 9606.ENSP00000338185 0 0 0 0 0 0 0.9 0.333 0.93

EDN1 NR3C2 9606.ENSP00000368683 9606.ENSP00000350815 0 0 0 0 0 0 0 0.469 0.469

EDN1 NGF 9606.ENSP00000368683 9606.ENSP00000358525 0 0 0 0 0 0 0 0.478 0.478

EDN1 JUN 9606.ENSP00000368683 9606.ENSP00000360266 0 0 0 0 0.074 0 0.9 0.594 0.959

EDN1 MMP9 9606.ENSP00000368683 9606.ENSP00000361405 0 0 0 0 0 0 0.8 0.652 0.927

EDN1 TLR4 9606.ENSP00000368683 9606.ENSP00000363089 0 0 0 0 0.081 0 0 0.471 0.493

EDN1 ITPR3 9606.ENSP00000368683 9606.ENSP00000363435 0 0 0 0 0.062 0 0 0.41 0.423

EDN1 THBD 9606.ENSP00000368683 9606.ENSP00000366307 0 0 0 0 0.077 0 0 0.556 0.572

EDN1 SPP1 9606.ENSP00000368683 9606.ENSP00000378517 0 0 0 0 0 0 0 0.495 0.495

EDN1 IL10 9606.ENSP00000368683 9606.ENSP00000412237 0 0 0 0 0 0 0 0.522 0.522

EDN1 NOS1 9606.ENSP00000368683 9606.ENSP00000477999 0 0 0 0 0 0 0 0.569 0.569

EDN1 IL6 9606.ENSP00000368683 9606.ENSP00000385675 0 0 0 0 0.088 0 0 0.731 0.744

EDN1 TNF 9606.ENSP00000368683 9606.ENSP00000398698 0 0 0 0 0.063 0 0 0.739 0.745

EDN1 INS 9606.ENSP00000368683 9606.ENSP00000380432 0 0 0 0 0 0 0 0.855 0.855

EDN1 VEGFA 9606.ENSP00000368683 9606.ENSP00000478570 0 0 0 0 0 0 0 0.923 0.923

EDN1 HIF1A 9606.ENSP00000368683 9606.ENSP00000437955 0 0 0 0 0 0 0.9 0.641 0.962

EDNRA MMP2 9606.ENSP00000315011 9606.ENSP00000219070 0 0 0 0 0.161 0.057 0 0.323 0.418

EDNRA EGF 9606.ENSP00000315011 9606.ENSP00000265171 0 0 0 0 0 0.056 0.9 0.266 0.924

EDNRA ERBB2 9606.ENSP00000315011 9606.ENSP00000269571 0 0 0 0 0.062 0.061 0 0.411 0.436

EDNRA REN 9606.ENSP00000315011 9606.ENSP00000272190 0 0 0 0 0 0 0 0.624 0.624

EDNRA EGFR 9606.ENSP00000315011 9606.ENSP00000275493 0 0 0 0 0.161 0.061 0.9 0.293 0.936

EDNRA SST 9606.ENSP00000315011 9606.ENSP00000287641 0 0 0 0 0 0 0.6 0.194 0.663

EDNRA NOS3 9606.ENSP00000315011 9606.ENSP00000297494 0 0 0 0 0.062 0 0 0.652 0.659

EDNRA F2 9606.ENSP00000315011 9606.ENSP00000308541 0 0 0 0 0 0.097 0.8 0.081 0.819

EDNRA LTA 9606.ENSP00000315011 9606.ENSP00000403495 0 0 0 0 0 0 0 0.433 0.434

EDNRA TNF 9606.ENSP00000315011 9606.ENSP00000398698 0 0 0 0 0 0 0 0.459 0.459

EDNRA VEGFA 9606.ENSP00000315011 9606.ENSP00000478570 0 0 0 0 0.063 0 0 0.502 0.513

EDNRA PLCB1 9606.ENSP00000315011 9606.ENSP00000338185 0 0 0 0 0 0.076 0.9 0.255 0.925

EGF TNFRSF1A 9606.ENSP00000265171 9606.ENSP00000162749 0 0 0 0 0 0 0 0.468 0.468

EGF HMOX1 9606.ENSP00000265171 9606.ENSP00000216117 0 0 0 0 0 0 0 0.4 0.4

EGF NFKBIA 9606.ENSP00000265171 9606.ENSP00000216797 0 0 0 0 0 0 0 0.504 0.504

EGF MMP2 9606.ENSP00000265171 9606.ENSP00000219070 0 0 0 0 0 0 0 0.671 0.671

EGF TGFB1 9606.ENSP00000265171 9606.ENSP00000221930 0 0 0 0 0 0 0 0.881 0.881

EGF MPO 9606.ENSP00000265171 9606.ENSP00000225275 0 0 0 0 0.062 0.069 0 0.386 0.417

EGF IL2 9606.ENSP00000265171 9606.ENSP00000226730 0 0 0 0 0 0 0 0.615 0.615

EGF KITLG 9606.ENSP00000265171 9606.ENSP00000228280 0 0 0 0 0 0 0 0.572 0.572

EGF IFNG 9606.ENSP00000265171 9606.ENSP00000229135 0 0 0 0 0 0 0 0.607 0.607

EGF IL4 9606.ENSP00000265171 9606.ENSP00000231449 0 0 0 0 0 0 0 0.635 0.635

EGF FGF23 9606.ENSP00000265171 9606.ENSP00000237837 0 0 0 0 0 0.058 0 0.679 0.685

EGF HSPB1 9606.ENSP00000265171 9606.ENSP00000248553 0 0 0 0 0.066 0 0 0.393 0.408

EGF MDM2 9606.ENSP00000265171 9606.ENSP00000258149 0 0 0 0 0 0 0 0.506 0.506

EGF SMAD7 9606.ENSP00000265171 9606.ENSP00000262158 0 0 0 0 0 0 0 0.586 0.586

EGF IL1A 9606.ENSP00000265171 9606.ENSP00000263339 0 0 0 0 0 0 0 0.7 0.7

EGF IL1B 9606.ENSP00000265171 9606.ENSP00000263341 0 0 0 0 0 0 0 0.71 0.71

EGF SELP 9606.ENSP00000265171 9606.ENSP00000263686 0 0 0 0 0 0 0 0.61 0.61

EGF FGF2 9606.ENSP00000265171 9606.ENSP00000264498 0 0 0 0 0 0.058 0 0.958 0.959

EGF FGF10 9606.ENSP00000265171 9606.ENSP00000264664 0 0 0 0 0 0.058 0 0.833 0.836

EGF ICAM1 9606.ENSP00000265171 9606.ENSP00000264832 0 0 0 0 0 0 0 0.56 0.56

EGF LEF1 9606.ENSP00000265171 9606.ENSP00000265165 0 0 0 0 0 0 0 0.463 0.463

EGF HDAC1 9606.ENSP00000265171 9606.ENSP00000362649 0 0 0 0 0 0 0 0.402 0.402

EGF PF4 9606.ENSP00000265171 9606.ENSP00000296029 0 0 0 0 0 0 0 0.406 0.406

EGF ITGB2 9606.ENSP00000265171 9606.ENSP00000380948 0 0 0 0 0 0.064 0 0.396 0.411

EGF REN 9606.ENSP00000265171 9606.ENSP00000272190 0 0 0 0 0 0 0 0.413 0.412

EGF FASLG 9606.ENSP00000265171 9606.ENSP00000356694 0 0 0 0 0 0 0 0.423 0.422

EGF HPRT1 9606.ENSP00000265171 9606.ENSP00000298556 0 0 0 0 0 0 0 0.429 0.429

EGF TH 9606.ENSP00000265171 9606.ENSP00000370571 0 0 0 0 0.054 0 0 0.457 0.464

EGF PRKCD 9606.ENSP00000265171 9606.ENSP00000378217 0 0 0 0 0.063 0 0 0.466 0.478

EGF SREBF1 9606.ENSP00000265171 9606.ENSP00000348069 0 0 0 0 0 0.27 0 0.325 0.486

EGF HSPA5 9606.ENSP00000265171 9606.ENSP00000324173 0 0 0 0 0 0 0 0.497 0.497

EGF RET 9606.ENSP00000265171 9606.ENSP00000347942 0 0 0 0 0 0.057 0 0.495 0.503

EGF SELE 9606.ENSP00000265171 9606.ENSP00000331736 0 0 0 0 0 0 0 0.505 0.505

EGF TLR4 9606.ENSP00000265171 9606.ENSP00000363089 0 0 0 0 0 0.056 0 0.507 0.514

EGF SST 9606.ENSP00000265171 9606.ENSP00000287641 0 0 0 0 0.063 0 0 0.509 0.52

EGF OCLN 9606.ENSP00000265171 9606.ENSP00000347379 0 0 0 0 0 0 0 0.526 0.526

EGF NOS3 9606.ENSP00000265171 9606.ENSP00000297494 0 0 0 0 0.062 0 0 0.517 0.527

EGF PPARG 9606.ENSP00000265171 9606.ENSP00000287820 0 0 0 0 0 0.056 0 0.522 0.529

EGF FURIN 9606.ENSP00000265171 9606.ENSP00000483552 0 0 0 0 0 0 0 0.539 0.539

EGF MMP3 9606.ENSP00000265171 9606.ENSP00000299855 0 0 0 0 0 0 0 0.548 0.548

EGF STAT5A 9606.ENSP00000265171 9606.ENSP00000341208 0 0 0 0 0 0 0 0.549 0.549

EGF VCAM1 9606.ENSP00000265171 9606.ENSP00000294728 0 0 0 0 0 0 0 0.557 0.557

EGF LEP 9606.ENSP00000265171 9606.ENSP00000312652 0 0 0 0 0 0 0 0.557 0.557

EGF IL17A 9606.ENSP00000265171 9606.ENSP00000344192 0 0 0 0 0 0 0 0.566 0.566

EGF SPP1 9606.ENSP00000265171 9606.ENSP00000378517 0 0 0 0 0 0 0 0.566 0.566

EGF LTA 9606.ENSP00000265171 9606.ENSP00000403495 0 0 0 0 0 0.059 0 0.587 0.595

EGF MMP1 9606.ENSP00000265171 9606.ENSP00000322788 0 0 0 0 0 0 0 0.626 0.626

EGF IL10 9606.ENSP00000265171 9606.ENSP00000412237 0 0 0 0 0 0 0 0.676 0.676

EGF HIF1A 9606.ENSP00000265171 9606.ENSP00000437955 0 0 0 0 0 0 0 0.683 0.683

EGF JUN 9606.ENSP00000265171 9606.ENSP00000360266 0 0 0 0 0 0 0 0.701 0.701

EGF IL13 9606.ENSP00000265171 9606.ENSP00000304915 0 0 0 0 0 0 0 0.707 0.707

EGF MMP9 9606.ENSP00000265171 9606.ENSP00000361405 0 0 0 0 0 0 0 0.728 0.728

EGF PTEN 9606.ENSP00000265171 9606.ENSP00000361021 0 0 0 0 0.068 0.062 0 0.714 0.728

EGF F2 9606.ENSP00000265171 9606.ENSP00000308541 0 0 0 0 0 0.056 0 0.747 0.751

EGF IL6 9606.ENSP00000265171 9606.ENSP00000385675 0 0 0 0 0 0 0 0.763 0.763

EGF TP53 9606.ENSP00000265171 9606.ENSP00000269305 0 0 0 0 0 0 0 0.766 0.766

EGF ESR1 9606.ENSP00000265171 9606.ENSP00000405330 0 0 0 0 0 0.056 0 0.782 0.785

EGF NGF 9606.ENSP00000265171 9606.ENSP00000358525 0 0 0 0 0.062 0 0 0.78 0.785

EGF TNF 9606.ENSP00000265171 9606.ENSP00000398698 0 0 0 0 0 0.059 0 0.783 0.787

EGF FGFR2 9606.ENSP00000265171 9606.ENSP00000410294 0 0 0 0 0 0.057 0.6 0.567 0.822

EGF INSR 9606.ENSP00000265171 9606.ENSP00000303830 0 0 0 0 0 0.056 0.8 0.323 0.861

EGF VEGFA 9606.ENSP00000265171 9606.ENSP00000478570 0 0 0 0 0 0 0 0.878 0.878

EGF PRKCB 9606.ENSP00000265171 9606.ENSP00000305355 0 0 0 0 0 0 0.9 0.243 0.921

EGF KIT 9606.ENSP00000265171 9606.ENSP00000288135 0 0 0 0 0 0.057 0.8 0.63 0.924

EGF STAT1 9606.ENSP00000265171 9606.ENSP00000354394 0 0 0 0 0 0 0.9 0.585 0.956

EGF MTOR 9606.ENSP00000265171 9606.ENSP00000354558 0 0 0 0 0 0 0.9 0.653 0.963

EGF IGF1 9606.ENSP00000265171 9606.ENSP00000302665 0 0 0 0 0 0 0.8 0.873 0.973

EGF INS 9606.ENSP00000265171 9606.ENSP00000380432 0 0 0 0 0 0 0.8 0.902 0.979

EGF P4HB 9606.ENSP00000265171 9606.ENSP00000327801 0 0 0 0 0 0.056 0 0.989 0.989

EGF ERBB2 9606.ENSP00000265171 9606.ENSP00000269571 0 0 0 0 0.16 0.321 0.9 0.989 0.999

EGF ERBB3 9606.ENSP00000265171 9606.ENSP00000267101 0 0 0 0 0.177 0.301 0.9 0.987 0.999

EGF EGFR 9606.ENSP00000265171 9606.ENSP00000275493 0 0 0 0 0.16 0.982 0.9 0.991 0.999

EGFR TNFRSF1A 9606.ENSP00000275493 9606.ENSP00000162749 0 0 0 0 0.062 0.642 0 0.582 0.847

EGFR MIF 9606.ENSP00000275493 9606.ENSP00000215754 0 0 0 0 0 0.27 0 0.289 0.458

EGFR NFKBIA 9606.ENSP00000275493 9606.ENSP00000216797 0 0 0 0 0 0.063 0 0.517 0.528

EGFR MMP2 9606.ENSP00000275493 9606.ENSP00000219070 0 0 0 0 0.11 0 0 0.684 0.707

EGFR TGFB1 9606.ENSP00000275493 9606.ENSP00000221930 0 0 0 0 0 0.183 0 0.683 0.73

EGFR NFKB1 9606.ENSP00000275493 9606.ENSP00000226574 0 0 0 0 0 0.063 0 0.399 0.413

EGFR IL2 9606.ENSP00000275493 9606.ENSP00000226730 0 0 0 0 0 0 0.6 0.556 0.815

EGFR KITLG 9606.ENSP00000275493 9606.ENSP00000228280 0 0 0 0 0.197 0 0.8 0.457 0.905

EGFR IFNG 9606.ENSP00000275493 9606.ENSP00000229135 0 0 0 0 0 0 0.6 0.515 0.798

EGFR IL4 9606.ENSP00000275493 9606.ENSP00000231449 0 0 0 0 0.062 0 0.6 0.468 0.783

EGFR NR3C1 9606.ENSP00000275493 9606.ENSP00000231509 0 0 0 0 0 0.664 0 0.394 0.788

EGFR FGF23 9606.ENSP00000275493 9606.ENSP00000237837 0 0 0 0 0 0 0.6 0.19 0.662

EGFR HSPB1 9606.ENSP00000275493 9606.ENSP00000248553 0 0 0 0 0.089 0.68 0 0.468 0.831

EGFR NOTCH2 9606.ENSP00000275493 9606.ENSP00000256646 0 0 0 0 0.089 0.229 0 0.551 0.657

EGFR MDM2 9606.ENSP00000275493 9606.ENSP00000258149 0 0 0 0 0 0 0.6 0.69 0.87

EGFR IL1A 9606.ENSP00000275493 9606.ENSP00000263339 0 0 0 0 0 0 0 0.533 0.533

EGFR IL1B 9606.ENSP00000275493 9606.ENSP00000263341 0 0 0 0 0 0 0 0.561 0.561

EGFR SELP 9606.ENSP00000275493 9606.ENSP00000263686 0 0 0 0 0 0 0 0.412 0.412

EGFR FGF2 9606.ENSP00000275493 9606.ENSP00000264498 0 0 0 0 0.098 0 0.6 0.719 0.889

EGFR FGF10 9606.ENSP00000275493 9606.ENSP00000264664 0 0 0 0 0 0 0.6 0.457 0.773

EGFR ICAM1 9606.ENSP00000275493 9606.ENSP00000264832 0 0 0 0 0 0.27 0 0.51 0.627

EGFR LEF1 9606.ENSP00000275493 9606.ENSP00000265165 0 0 0 0 0.049 0 0 0.44 0.445

EGFR ERBB3 9606.ENSP00000275493 9606.ENSP00000267101 0 0 0 0.913 0.062 0.972 0.9 0.991 0.997

EGFR TP53 9606.ENSP00000275493 9606.ENSP00000269305 0 0 0 0 0 0.279 0 0.877 0.908

EGFR ERBB2 9606.ENSP00000275493 9606.ENSP00000269571 0 0 0 0.941 0.077 0.982 0.9 0.99 0.998

EGFR MTTP 9606.ENSP00000275493 9606.ENSP00000427679 0 0 0 0 0 0.397 0 0.05 0.402

EGFR IL17A 9606.ENSP00000275493 9606.ENSP00000344192 0 0 0 0 0 0 0 0.409 0.408

EGFR ITPR1 9606.ENSP00000275493 9606.ENSP00000306253 0 0 0 0 0.065 0.056 0 0.388 0.412

EGFR GPT 9606.ENSP00000275493 9606.ENSP00000378408 0 0 0 0 0 0 0 0.425 0.424

EGFR SELE 9606.ENSP00000275493 9606.ENSP00000331736 0 0 0 0 0 0 0 0.425 0.425

EGFR TPO 9606.ENSP00000275493 9606.ENSP00000318820 0 0 0 0 0.062 0 0 0.413 0.426

EGFR TLR3 9606.ENSP00000275493 9606.ENSP00000296795 0 0 0 0 0 0.103 0 0.388 0.427

EGFR VDR 9606.ENSP00000275493 9606.ENSP00000447173 0 0 0 0 0 0.102 0 0.391 0.429

EGFR ITPR3 9606.ENSP00000275493 9606.ENSP00000363435 0 0 0 0 0.099 0.056 0 0.385 0.431

EGFR NOS3 9606.ENSP00000275493 9606.ENSP00000297494 0 0 0 0 0 0 0 0.459 0.459

EGFR FOXP3 9606.ENSP00000275493 9606.ENSP00000365380 0 0 0 0 0 0 0 0.46 0.46

EGFR IL17RA 9606.ENSP00000275493 9606.ENSP00000320936 0 0 0 0 0 0 0 0.463 0.463

EGFR FASLG 9606.ENSP00000275493 9606.ENSP00000356694 0 0 0 0 0 0 0 0.469 0.469

EGFR VCAM1 9606.ENSP00000275493 9606.ENSP00000294728 0 0 0 0 0.076 0 0 0.453 0.472

EGFR FURIN 9606.ENSP00000275493 9606.ENSP00000483552 0 0 0 0 0.062 0 0 0.461 0.473

EGFR FGFR2 9606.ENSP00000275493 9606.ENSP00000410294 0 0 0 0.61 0.063 0.209 0 0.838 0.477

EGFR MYD88 9606.ENSP00000275493 9606.ENSP00000401399 0 0 0 0 0 0 0 0.488 0.488

EGFR KIT 9606.ENSP00000275493 9606.ENSP00000288135 0 0 0 0.568 0.054 0.243 0 0.765 0.496

EGFR OCLN 9606.ENSP00000275493 9606.ENSP00000347379 0 0 0 0 0.062 0 0 0.523 0.534

EGFR MMP1 9606.ENSP00000275493 9606.ENSP00000322788 0 0 0 0 0.078 0 0 0.516 0.535

EGFR LCN2 9606.ENSP00000275493 9606.ENSP00000362108 0 0 0 0 0 0 0 0.535 0.535

EGFR HDAC1 9606.ENSP00000275493 9606.ENSP00000362649 0 0 0 0 0 0.103 0 0.532 0.562

EGFR P4HB 9606.ENSP00000275493 9606.ENSP00000327801 0 0 0 0 0.081 0.183 0 0.463 0.562

EGFR SPP1 9606.ENSP00000275493 9606.ENSP00000378517 0 0 0 0 0 0 0 0.6 0.6

EGFR NKX2-1 9606.ENSP00000275493 9606.ENSP00000346879 0 0 0 0 0 0.058 0 0.636 0.642

EGFR PARP1 9606.ENSP00000275493 9606.ENSP00000355759 0 0 0 0 0 0.31 0 0.506 0.644

EGFR INSR 9606.ENSP00000275493 9606.ENSP00000303830 0 0 0 0.574 0.062 0.107 0.5 0.567 0.651

EGFR PPARG 9606.ENSP00000275493 9606.ENSP00000287820 0 0 0 0 0.062 0.316 0 0.517 0.663

EGFR TOP1 9606.ENSP00000275493 9606.ENSP00000354522 0 0 0 0 0 0.27 0 0.602 0.697

EGFR PLCB1 9606.ENSP00000275493 9606.ENSP00000338185 0 0 0 0 0 0.139 0.6 0.226 0.71

EGFR TNF 9606.ENSP00000275493 9606.ENSP00000398698 0 0 0 0 0.063 0 0 0.704 0.712

EGFR SNCA 9606.ENSP00000275493 9606.ENSP00000338345 0 0 0 0 0 0.622 0 0.281 0.716

EGFR ITGB2 9606.ENSP00000275493 9606.ENSP00000380948 0 0 0 0 0.051 0.649 0 0.255 0.731

EGFR MMP9 9606.ENSP00000275493 9606.ENSP00000361405 0 0 0 0 0 0 0 0.733 0.733

EGFR JUN 9606.ENSP00000275493 9606.ENSP00000360266 0 0 0 0 0.06 0.139 0 0.732 0.764

EGFR SOCS1 9606.ENSP00000275493 9606.ENSP00000329418 0 0 0 0 0 0.667 0 0.39 0.788

EGFR IL10 9606.ENSP00000275493 9606.ENSP00000412237 0 0 0 0 0 0 0.6 0.521 0.8

EGFR LEP 9606.ENSP00000275493 9606.ENSP00000312652 0 0 0 0 0 0 0.6 0.53 0.804

EGFR NGF 9606.ENSP00000275493 9606.ENSP00000358525 0 0 0 0 0.098 0 0.6 0.505 0.805

EGFR IL13 9606.ENSP00000275493 9606.ENSP00000304915 0 0 0 0 0 0 0.6 0.567 0.819

EGFR RET 9606.ENSP00000275493 9606.ENSP00000347942 0 0 0 0.593 0.063 0.723 0 0.865 0.824

EGFR MMP3 9606.ENSP00000275493 9606.ENSP00000299855 0 0 0 0 0.076 0 0.65 0.541 0.838

EGFR TLR4 9606.ENSP00000275493 9606.ENSP00000363089 0 0 0 0 0 0.317 0 0.773 0.838

EGFR IL6 9606.ENSP00000275493 9606.ENSP00000385675 0 0 0 0 0 0 0.6 0.683 0.867

EGFR IGF1 9606.ENSP00000275493 9606.ENSP00000302665 0 0 0 0 0.062 0 0.6 0.783 0.912

EGFR PTEN 9606.ENSP00000275493 9606.ENSP00000361021 0 0 0 0 0 0.342 0 0.899 0.931

EGFR PRKCB 9606.ENSP00000275493 9606.ENSP00000305355 0 0 0 0.553 0.049 0.282 0.9 0.325 0.935

EGFR INS 9606.ENSP00000275493 9606.ENSP00000380432 0 0 0 0 0 0 0.8 0.724 0.942

EGFR VEGFA 9606.ENSP00000275493 9606.ENSP00000478570 0 0 0 0 0.089 0 0.6 0.861 0.944

EGFR FAS 9606.ENSP00000275493 9606.ENSP00000347979 0 0 0 0 0 0.213 0 0.935 0.946

EGFR HIF1A 9606.ENSP00000275493 9606.ENSP00000437955 0 0 0 0 0 0 0.9 0.776 0.976

EGFR MTOR 9606.ENSP00000275493 9606.ENSP00000354558 0 0 0 0 0.062 0.197 0.9 0.751 0.978

EGFR HSPA5 9606.ENSP00000275493 9606.ENSP00000324173 0 0 0 0 0 0.682 0 0.955 0.985

EGFR ESR1 9606.ENSP00000275493 9606.ENSP00000405330 0 0 0 0 0 0.696 0 0.988 0.996

EGFR STAT1 9606.ENSP00000275493 9606.ENSP00000354394 0 0 0 0 0.062 0.875 0.9 0.709 0.996

EGFR STAT5A 9606.ENSP00000275493 9606.ENSP00000341208 0 0 0 0 0.049 0.881 0.8 0.938 0.998

ELANE MMP2 9606.ENSP00000466090 9606.ENSP00000219070 0 0 0 0 0 0 0 0.519 0.519

ELANE MPO 9606.ENSP00000466090 9606.ENSP00000225275 0 0 0 0 0.784 0.141 0 0.859 0.971

ELANE IFNG 9606.ENSP00000466090 9606.ENSP00000229135 0 0 0 0 0 0 0 0.415 0.415

ELANE IL4 9606.ENSP00000466090 9606.ENSP00000231449 0 0 0 0 0 0 0 0.452 0.452

ELANE LTF 9606.ENSP00000466090 9606.ENSP00000231751 0 0 0 0 0.155 0.169 0 0.597 0.692

ELANE TFPI 9606.ENSP00000466090 9606.ENSP00000233156 0 0 0 0 0 0.296 0 0.413 0.569

ELANE LYZ 9606.ENSP00000466090 9606.ENSP00000261267 0 0 0 0 0.112 0 0 0.373 0.419

ELANE IL1A 9606.ENSP00000466090 9606.ENSP00000263339 0 0 0 0 0 0 0 0.427 0.426

ELANE IL1B 9606.ENSP00000466090 9606.ENSP00000263341 0 0 0 0 0.078 0 0 0.662 0.675

ELANE SELP 9606.ENSP00000466090 9606.ENSP00000263686 0 0 0 0 0.07 0 0 0.505 0.519

ELANE ICAM1 9606.ENSP00000466090 9606.ENSP00000264832 0 0 0 0 0 0 0 0.519 0.519

ELANE VCAM1 9606.ENSP00000466090 9606.ENSP00000294728 0 0 0 0 0 0.213 0 0.591 0.664

ELANE PF4 9606.ENSP00000466090 9606.ENSP00000296029 0 0 0 0 0.097 0 0 0.391 0.426

ELANE F2RL1 9606.ENSP00000466090 9606.ENSP00000296677 0 0 0 0 0 0.213 0 0.309 0.433

ELANE MMP3 9606.ENSP00000466090 9606.ENSP00000299855 0 0 0 0 0.052 0 0 0.414 0.42

ELANE IL13 9606.ENSP00000466090 9606.ENSP00000304915 0 0 0 0 0 0 0 0.458 0.457

ELANE F2 9606.ENSP00000466090 9606.ENSP00000308541 0 0 0.352 0.617 0.068 0 0.5 0.525 0.656

ELANE MMP1 9606.ENSP00000466090 9606.ENSP00000322788 0 0 0 0 0.052 0 0.9 0.459 0.944

ELANE SELE 9606.ENSP00000466090 9606.ENSP00000331736 0 0 0 0 0 0 0 0.427 0.426

ELANE IL17A 9606.ENSP00000466090 9606.ENSP00000344192 0 0 0 0 0 0 0 0.522 0.522

ELANE S100A9 9606.ENSP00000466090 9606.ENSP00000357727 0 0 0 0 0.158 0 0 0.37 0.447

ELANE MMP9 9606.ENSP00000466090 9606.ENSP00000361405 0 0 0 0 0.095 0 0 0.693 0.71

ELANE LCN2 9606.ENSP00000466090 9606.ENSP00000362108 0 0 0 0 0.12 0 0 0.507 0.547

ELANE TLR4 9606.ENSP00000466090 9606.ENSP00000363089 0 0 0 0 0.083 0.056 0 0.564 0.59

ELANE THBD 9606.ENSP00000466090 9606.ENSP00000366307 0 0 0 0 0.06 0 0 0.468 0.478

ELANE IL6 9606.ENSP00000466090 9606.ENSP00000385675 0 0 0 0 0 0 0 0.662 0.662

ELANE TNF 9606.ENSP00000466090 9606.ENSP00000398698 0 0 0 0 0.062 0 0 0.667 0.675

ELANE IL10 9606.ENSP00000466090 9606.ENSP00000412237 0 0 0 0 0 0 0 0.528 0.528

ELANE VEGFA 9606.ENSP00000466090 9606.ENSP00000478570 0 0 0 0 0 0 0 0.459 0.459

ERBB2 NFKBIA 9606.ENSP00000269571 9606.ENSP00000216797 0 0 0 0 0 0.063 0 0.392 0.405

ERBB2 MMP2 9606.ENSP00000269571 9606.ENSP00000219070 0 0 0 0 0.063 0 0 0.606 0.615

ERBB2 TGFB1 9606.ENSP00000269571 9606.ENSP00000221930 0 0 0 0 0 0.179 0 0.495 0.568

ERBB2 IL2 9606.ENSP00000269571 9606.ENSP00000226730 0 0 0 0 0 0 0 0.604 0.604

ERBB2 KITLG 9606.ENSP00000269571 9606.ENSP00000228280 0 0 0 0 0.062 0 0.6 0.322 0.723

ERBB2 IFNG 9606.ENSP00000269571 9606.ENSP00000229135 0 0 0 0 0 0 0 0.459 0.459

ERBB2 IL4 9606.ENSP00000269571 9606.ENSP00000231449 0 0 0 0 0.062 0 0 0.458 0.469

ERBB2 NR3C1 9606.ENSP00000269571 9606.ENSP00000231509 0 0 0 0 0 0.102 0 0.381 0.42

ERBB2 FGF23 9606.ENSP00000269571 9606.ENSP00000237837 0 0 0 0 0 0 0.6 0.121 0.633

ERBB2 HSPB1 9606.ENSP00000269571 9606.ENSP00000248553 0 0 0 0 0.062 0 0 0.467 0.478

ERBB2 NOTCH2 9606.ENSP00000269571 9606.ENSP00000256646 0 0 0 0 0.059 0.229 0 0.511 0.614

ERBB2 MDM2 9606.ENSP00000269571 9606.ENSP00000258149 0 0 0 0 0 0 0 0.662 0.662

ERBB2 IL1B 9606.ENSP00000269571 9606.ENSP00000263341 0 0 0 0 0 0 0 0.575 0.575

ERBB2 FGF2 9606.ENSP00000269571 9606.ENSP00000264498 0 0 0 0 0.062 0 0.6 0.57 0.824

ERBB2 FGF10 9606.ENSP00000269571 9606.ENSP00000264664 0 0 0 0 0 0 0.6 0.315 0.714

ERBB2 ICAM1 9606.ENSP00000269571 9606.ENSP00000264832 0 0 0 0 0 0 0 0.425 0.424

ERBB2 ERBB3 9606.ENSP00000269571 9606.ENSP00000267101 0 0 0 0.897 0.117 0.915 0.9 0.991 0.992

ERBB2 TP53 9606.ENSP00000269571 9606.ENSP00000269305 0 0 0 0 0 0.129 0 0.878 0.889

ERBB2 LEP 9606.ENSP00000269571 9606.ENSP00000312652 0 0 0 0 0 0 0 0.404 0.404

ERBB2 GPT 9606.ENSP00000269571 9606.ENSP00000378408 0 0 0 0 0 0 0 0.414 0.414

ERBB2 INSR 9606.ENSP00000269571 9606.ENSP00000303830 0 0 0 0.567 0.062 0.246 0 0.512 0.42

ERBB2 FURIN 9606.ENSP00000269571 9606.ENSP00000483552 0 0 0 0 0.062 0 0 0.416 0.428

ERBB2 PPARD 9606.ENSP00000269571 9606.ENSP00000310928 0 0 0 0 0.062 0.102 0 0.382 0.434

ERBB2 HDAC1 9606.ENSP00000269571 9606.ENSP00000362649 0 0 0 0 0 0 0 0.44 0.44

ERBB2 FASLG 9606.ENSP00000269571 9606.ENSP00000356694 0 0 0 0 0 0 0 0.459 0.459

ERBB2 MMP1 9606.ENSP00000269571 9606.ENSP00000322788 0 0 0 0 0 0 0 0.464 0.464

ERBB2 HSPA5 9606.ENSP00000269571 9606.ENSP00000324173 0 0 0 0 0 0.163 0 0.39 0.467

ERBB2 TLR3 9606.ENSP00000269571 9606.ENSP00000296795 0 0 0 0 0 0.103 0 0.432 0.468

ERBB2 IL10 9606.ENSP00000269571 9606.ENSP00000412237 0 0 0 0 0 0 0 0.469 0.469

ERBB2 NKX2-1 9606.ENSP00000269571 9606.ENSP00000346879 0 0 0 0 0 0.058 0 0.469 0.478

ERBB2 KIT 9606.ENSP00000269571 9606.ENSP00000288135 0 0 0 0.567 0.054 0.243 0 0.722 0.482

ERBB2 IL13 9606.ENSP00000269571 9606.ENSP00000304915 0 0 0 0 0 0 0 0.488 0.488

ERBB2 PPARG 9606.ENSP00000269571 9606.ENSP00000287820 0 0 0 0 0 0.102 0 0.457 0.492

ERBB2 SPP1 9606.ENSP00000269571 9606.ENSP00000378517 0 0 0 0 0 0.117 0 0.45 0.493

ERBB2 RET 9606.ENSP00000269571 9606.ENSP00000347942 0 0 0 0.593 0.063 0.277 0 0.762 0.51

ERBB2 FOXP3 9606.ENSP00000269571 9606.ENSP00000365380 0 0 0 0 0 0 0 0.52 0.52

ERBB2 SOCS1 9606.ENSP00000269571 9606.ENSP00000329418 0 0 0 0 0 0.424 0 0.234 0.54

ERBB2 FGFR2 9606.ENSP00000269571 9606.ENSP00000410294 0 0 0 0.617 0.083 0.332 0 0.768 0.547

ERBB2 TOP1 9606.ENSP00000269571 9606.ENSP00000354522 0 0 0 0 0 0.13 0 0.518 0.562

ERBB2 STAT5A 9606.ENSP00000269571 9606.ENSP00000341208 0 0 0 0 0.049 0.116 0 0.542 0.581

ERBB2 TNF 9606.ENSP00000269571 9606.ENSP00000398698 0 0 0 0 0.062 0 0 0.61 0.618

ERBB2 PARP1 9606.ENSP00000269571 9606.ENSP00000355759 0 0 0 0 0 0 0 0.624 0.624

ERBB2 JUN 9606.ENSP00000269571 9606.ENSP00000360266 0 0 0 0 0.062 0.139 0 0.644 0.687

ERBB2 HIF1A 9606.ENSP00000269571 9606.ENSP00000437955 0 0 0 0 0 0 0 0.708 0.708

ERBB2 MTOR 9606.ENSP00000269571 9606.ENSP00000354558 0 0 0 0 0.049 0.058 0 0.71 0.717

ERBB2 MMP9 9606.ENSP00000269571 9606.ENSP00000361405 0 0 0 0 0 0 0 0.723 0.724

ERBB2 NGF 9606.ENSP00000269571 9606.ENSP00000358525 0 0 0 0 0.062 0 0.6 0.369 0.742

ERBB2 STAT1 9606.ENSP00000269571 9606.ENSP00000354394 0 0 0 0 0.062 0.624 0 0.479 0.8

ERBB2 INS 9606.ENSP00000269571 9606.ENSP00000380432 0 0 0 0 0 0 0.6 0.702 0.875

ERBB2 PTEN 9606.ENSP00000269571 9606.ENSP00000361021 0 0 0 0 0 0.136 0 0.887 0.898

ERBB2 VEGFA 9606.ENSP00000269571 9606.ENSP00000478570 0 0 0 0 0 0 0.6 0.805 0.918

ERBB2 IGF1 9606.ENSP00000269571 9606.ENSP00000302665 0 0 0 0 0.049 0 0.6 0.84 0.934

ERBB2 ESR1 9606.ENSP00000269571 9606.ENSP00000405330 0 0 0 0 0 0.316 0 0.918 0.941

ERBB2 IL6 9606.ENSP00000269571 9606.ENSP00000385675 0 0 0 0 0 0 0.9 0.673 0.965

ERBB3 KITLG 9606.ENSP00000267101 9606.ENSP00000228280 0 0 0 0 0 0 0.6 0.23 0.678

ERBB3 FGF23 9606.ENSP00000267101 9606.ENSP00000237837 0 0 0 0 0 0 0.6 0.085 0.618

ERBB3 NOTCH2 9606.ENSP00000267101 9606.ENSP00000256646 0 0 0 0 0 0.229 0 0.338 0.468

ERBB3 MDM2 9606.ENSP00000267101 9606.ENSP00000258149 0 0 0 0 0 0.27 0 0.427 0.563

ERBB3 FGF2 9606.ENSP00000267101 9606.ENSP00000264498 0 0 0 0 0 0 0.6 0.404 0.751

ERBB3 FGF10 9606.ENSP00000267101 9606.ENSP00000264664 0 0 0 0 0 0 0.6 0.197 0.665

ERBB3 MMP9 9606.ENSP00000267101 9606.ENSP00000361405 0 0 0 0 0 0 0 0.406 0.406

ERBB3 HIF1A 9606.ENSP00000267101 9606.ENSP00000437955 0 0 0 0 0 0 0 0.449 0.449

ERBB3 STAT5A 9606.ENSP00000267101 9606.ENSP00000341208 0 0 0 0 0.049 0.116 0 0.437 0.485

ERBB3 KIT 9606.ENSP00000267101 9606.ENSP00000288135 0 0 0 0.566 0.054 0.395 0 0.525 0.533

ERBB3 MTOR 9606.ENSP00000267101 9606.ENSP00000354558 0 0 0 0 0 0.058 0 0.53 0.538

ERBB3 RET 9606.ENSP00000267101 9606.ENSP00000347942 0 0 0 0.574 0.063 0.391 0 0.595 0.551

ERBB3 JUN 9606.ENSP00000267101 9606.ENSP00000360266 0 0 0 0 0 0.139 0 0.505 0.556

ERBB3 FGFR2 9606.ENSP00000267101 9606.ENSP00000410294 0 0 0 0.598 0.118 0.39 0 0.725 0.6

ERBB3 TP53 9606.ENSP00000267101 9606.ENSP00000269305 0 0 0 0 0 0.13 0 0.633 0.667

ERBB3 NGF 9606.ENSP00000267101 9606.ENSP00000358525 0 0 0 0 0 0 0.6 0.345 0.727

ERBB3 ESR1 9606.ENSP00000267101 9606.ENSP00000405330 0 0 0 0 0 0.102 0 0.725 0.743

ERBB3 INS 9606.ENSP00000267101 9606.ENSP00000380432 0 0 0 0 0 0 0.6 0.475 0.781

ERBB3 PTEN 9606.ENSP00000267101 9606.ENSP00000361021 0 0 0 0 0 0.191 0 0.768 0.804

ERBB3 IGF1 9606.ENSP00000267101 9606.ENSP00000302665 0 0 0 0 0 0 0.6 0.561 0.817

ERBB3 VEGFA 9606.ENSP00000267101 9606.ENSP00000478570 0 0 0 0 0 0 0.6 0.563 0.818

ESR1 NFKBIA 9606.ENSP00000405330 9606.ENSP00000216797 0 0 0 0 0 0.057 0 0.499 0.508

ESR1 MMP2 9606.ENSP00000405330 9606.ENSP00000219070 0 0 0 0 0 0 0 0.56 0.56

ESR1 TGFB1 9606.ENSP00000405330 9606.ENSP00000221930 0 0 0 0 0 0 0 0.488 0.488

ESR1 NFKB1 9606.ENSP00000405330 9606.ENSP00000226574 0 0 0 0 0 0.494 0 0.696 0.839

ESR1 IFNG 9606.ENSP00000405330 9606.ENSP00000229135 0 0 0 0 0 0 0 0.415 0.415

ESR1 IL4 9606.ENSP00000405330 9606.ENSP00000231449 0 0 0 0 0.062 0 0 0.407 0.419

ESR1 NR3C1 9606.ENSP00000405330 9606.ENSP00000231509 0 0 0 0.66 0 0 0.9 0.89 0.93

ESR1 HSPB1 9606.ENSP00000405330 9606.ENSP00000248553 0 0 0 0 0 0.346 0.9 0.422 0.958

ESR1 MDM2 9606.ENSP00000405330 9606.ENSP00000258149 0 0 0 0 0 0.757 0 0.81 0.952

ESR1 SLC6A4 9606.ENSP00000405330 9606.ENSP00000261707 0 0 0 0 0.062 0.057 0 0.391 0.414

ESR1 IL1B 9606.ENSP00000405330 9606.ENSP00000263341 0 0 0 0 0 0 0 0.729 0.729

ESR1 FGF2 9606.ENSP00000405330 9606.ENSP00000264498 0 0 0 0 0 0 0 0.51 0.51

ESR1 LEF1 9606.ENSP00000405330 9606.ENSP00000265165 0 0 0 0 0 0.182 0 0.54 0.607

ESR1 TP53 9606.ENSP00000405330 9606.ENSP00000269305 0 0 0 0 0 0.835 0 0.968 0.994

ESR1 SOD1 9606.ENSP00000405330 9606.ENSP00000270142 0 0 0 0 0.062 0 0 0.703 0.71

ESR1 SRD5A1 9606.ENSP00000405330 9606.ENSP00000274192 0 0 0 0 0 0 0 0.404 0.404

ESR1 KIT 9606.ENSP00000405330 9606.ENSP00000288135 0 0 0 0 0 0.057 0 0.628 0.634

ESR1 NOS3 9606.ENSP00000405330 9606.ENSP00000297494 0 0 0 0 0 0.345 0.9 0.985 0.998

ESR1 MMP3 9606.ENSP00000405330 9606.ENSP00000299855 0 0 0 0 0 0 0 0.435 0.435

ESR1 IGF1 9606.ENSP00000405330 9606.ENSP00000302665 0 0 0 0 0.062 0 0.9 0.988 0.998

ESR1 INSR 9606.ENSP00000405330 9606.ENSP00000303830 0 0 0 0 0 0.077 0 0.391 0.413

ESR1 GSTM1 9606.ENSP00000405330 9606.ENSP00000311469 0 0 0 0 0 0.062 0 0.406 0.419

ESR1 LEP 9606.ENSP00000405330 9606.ENSP00000312652 0 0 0 0 0 0 0 0.62 0.62

ESR1 MMP1 9606.ENSP00000405330 9606.ENSP00000322788 0 0 0 0 0 0 0 0.418 0.418

ESR1 HSPA5 9606.ENSP00000405330 9606.ENSP00000324173 0 0 0 0 0 0.309 0 0.404 0.571

ESR1 P4HB 9606.ENSP00000405330 9606.ENSP00000327801 0 0 0 0 0 0.128 0 0.88 0.891

ESR1 STAT5A 9606.ENSP00000405330 9606.ENSP00000341208 0 0 0 0 0.065 0.501 0.9 0.561 0.976

ESR1 USP7 9606.ENSP00000405330 9606.ENSP00000343535 0 0 0 0 0 0.328 0 0.184 0.428

ESR1 NKX2-1 9606.ENSP00000405330 9606.ENSP00000346879 0 0 0 0 0 0.07 0 0.505 0.519

ESR1 OCLN 9606.ENSP00000405330 9606.ENSP00000347379 0 0 0 0 0 0 0 0.496 0.496

ESR1 RET 9606.ENSP00000405330 9606.ENSP00000347942 0 0 0 0 0 0.057 0 0.467 0.475

ESR1 SREBF1 9606.ENSP00000405330 9606.ENSP00000348069 0 0 0 0 0 0.213 0 0.451 0.549

ESR1 STAT1 9606.ENSP00000405330 9606.ENSP00000354394 0 0 0 0 0 0.142 0.65 0.487 0.832

ESR1 MTOR 9606.ENSP00000405330 9606.ENSP00000354558 0 0 0 0 0 0.285 0 0.712 0.785

ESR1 PARP1 9606.ENSP00000405330 9606.ENSP00000355759 0 0 0 0 0 0.292 0 0.932 0.95

ESR1 NGF 9606.ENSP00000405330 9606.ENSP00000358525 0 0 0 0 0 0 0 0.459 0.459

ESR1 JUN 9606.ENSP00000405330 9606.ENSP00000360266 0 0 0 0 0 0.684 0.9 0.988 0.999

ESR1 PTEN 9606.ENSP00000405330 9606.ENSP00000361021 0 0 0 0 0 0.282 0 0.715 0.787

ESR1 MMP9 9606.ENSP00000405330 9606.ENSP00000361405 0 0 0 0 0 0 0 0.615 0.615

ESR1 HDAC1 9606.ENSP00000405330 9606.ENSP00000362649 0 0 0 0 0 0.876 0.9 0.987 0.999

ESR1 FOXP3 9606.ENSP00000405330 9606.ENSP00000365380 0 0 0 0 0 0.062 0 0.547 0.557

ESR1 PCNA 9606.ENSP00000405330 9606.ENSP00000368458 0 0 0 0 0.049 0.079 0.9 0.075 0.908

ESR1 SHBG 9606.ENSP00000405330 9606.ENSP00000369816 0 0 0 0 0 0 0 0.835 0.835

ESR1 NFIB 9606.ENSP00000405330 9606.ENSP00000370340 0 0 0 0 0 0.306 0 0.266 0.469

ESR1 TH 9606.ENSP00000405330 9606.ENSP00000370571 0 0 0 0 0.052 0 0 0.451 0.457

ESR1 PRKCD 9606.ENSP00000405330 9606.ENSP00000378217 0 0 0 0 0 0.056 0 0.436 0.445

ESR1 SPP1 9606.ENSP00000405330 9606.ENSP00000378517 0 0 0 0 0 0 0 0.599 0.599

ESR1 INS 9606.ENSP00000405330 9606.ENSP00000380432 0 0 0 0 0 0 0 0.771 0.771

ESR1 IL6 9606.ENSP00000405330 9606.ENSP00000385675 0 0 0 0 0 0 0 0.677 0.677

ESR1 TNF 9606.ENSP00000405330 9606.ENSP00000398698 0 0 0 0 0 0 0 0.622 0.622

ESR1 IL10 9606.ENSP00000405330 9606.ENSP00000412237 0 0 0 0 0 0 0 0.46 0.459

ESR1 FGFR2 9606.ENSP00000405330 9606.ENSP00000410294 0 0 0 0 0 0.057 0 0.561 0.568

ESR1 VEGFA 9606.ENSP00000405330 9606.ENSP00000478570 0 0 0 0 0.062 0 0 0.685 0.691

ESR1 HIF1A 9606.ENSP00000405330 9606.ENSP00000437955 0 0 0 0 0 0.587 0 0.835 0.929

F2 TGFB1 9606.ENSP00000308541 9606.ENSP00000221930 0 0 0 0 0 0 0 0.425 0.425

F2 MPO 9606.ENSP00000308541 9606.ENSP00000225275 0 0 0 0 0 0.056 0 0.503 0.51

F2 IFNG 9606.ENSP00000308541 9606.ENSP00000229135 0 0 0 0 0 0 0 0.402 0.402

F2 TFPI 9606.ENSP00000308541 9606.ENSP00000233156 0 0 0 0 0 0.143 0 0.708 0.739

F2 ORM1 9606.ENSP00000308541 9606.ENSP00000259396 0 0 0 0 0.566 0 0 0.346 0.704

F2 IL1B 9606.ENSP00000308541 9606.ENSP00000263341 0 0 0 0 0 0 0 0.556 0.556

F2 SELP 9606.ENSP00000308541 9606.ENSP00000263686 0 0 0 0 0.062 0 0 0.768 0.773

F2 FGF2 9606.ENSP00000308541 9606.ENSP00000264498 0 0 0 0 0 0 0 0.498 0.498

F2 REN 9606.ENSP00000308541 9606.ENSP00000272190 0 0 0 0 0.087 0 0 0.435 0.463

F2 VCAM1 9606.ENSP00000308541 9606.ENSP00000294728 0 0 0 0 0 0 0 0.508 0.508

F2 PF4 9606.ENSP00000308541 9606.ENSP00000296029 0 0 0 0 0 0 0 0.673 0.673

F2 F2RL1 9606.ENSP00000308541 9606.ENSP00000296677 0 0 0 0 0.062 0.213 0.6 0.384 0.794

F2 NOS3 9606.ENSP00000308541 9606.ENSP00000297494 0 0 0 0 0 0 0 0.405 0.405

F2 IGF1 9606.ENSP00000308541 9606.ENSP00000302665 0 0 0 0 0.105 0 0.9 0.38 0.939

F2 TLR4 9606.ENSP00000308541 9606.ENSP00000363089 0 0 0 0 0 0.056 0 0.409 0.418

F2 IL10 9606.ENSP00000308541 9606.ENSP00000412237 0 0 0 0 0 0 0 0.446 0.446

F2 SELE 9606.ENSP00000308541 9606.ENSP00000331736 0 0 0 0 0.049 0 0 0.553 0.556

F2 MMP9 9606.ENSP00000308541 9606.ENSP00000361405 0 0 0 0 0 0 0 0.573 0.573

F2 INS 9606.ENSP00000308541 9606.ENSP00000380432 0 0 0 0 0 0 0 0.578 0.578

F2 VEGFA 9606.ENSP00000308541 9606.ENSP00000478570 0 0 0 0 0 0 0 0.583 0.583

F2 TNF 9606.ENSP00000308541 9606.ENSP00000398698 0 0 0 0 0 0 0 0.627 0.627

F2 IL6 9606.ENSP00000308541 9606.ENSP00000385675 0 0 0 0 0 0 0 0.633 0.633

F2 HDAC1 9606.ENSP00000308541 9606.ENSP00000362649 0 0 0 0 0 0 0 0.642 0.642

F2 SPP1 9606.ENSP00000308541 9606.ENSP00000378517 0 0 0 0 0.062 0.608 0 0.514 0.806

F2 PLCB1 9606.ENSP00000308541 9606.ENSP00000338185 0 0 0 0 0 0 0.9 0 0.9

F2 GPT 9606.ENSP00000308541 9606.ENSP00000378408 0 0 0 0 0.062 0 0 0.912 0.913

F2 FURIN 9606.ENSP00000308541 9606.ENSP00000483552 0 0 0 0 0.062 0 0.9 0.274 0.926

F2 THBD 9606.ENSP00000308541 9606.ENSP00000366307 0 0 0 0 0 0.962 0.8 0.925 0.999

F2RL1 TLR4 9606.ENSP00000296677 9606.ENSP00000363089 0 0 0 0 0 0.27 0 0.633 0.721

FADD TNFRSF1A 9606.ENSP00000301838 9606.ENSP00000162749 0 0 0 0 0.062 0.758 0.9 0.989 0.999

FADD NFKBIA 9606.ENSP00000301838 9606.ENSP00000216797 0 0 0 0 0 0.056 0 0.56 0.567

FADD NFKB1 9606.ENSP00000301838 9606.ENSP00000226574 0 0 0 0 0.099 0.056 0 0.398 0.443

FADD IL1B 9606.ENSP00000301838 9606.ENSP00000263341 0 0 0 0 0 0 0 0.566 0.566

FADD TP53 9606.ENSP00000301838 9606.ENSP00000269305 0 0 0 0 0.063 0 0 0.638 0.646

FADD SOD1 9606.ENSP00000301838 9606.ENSP00000270142 0 0 0 0 0.068 0 0 0.486 0.5

FADD PPARG 9606.ENSP00000301838 9606.ENSP00000287820 0 0 0 0 0 0 0 0.433 0.433

FADD TLR3 9606.ENSP00000301838 9606.ENSP00000296795 0 0 0 0 0 0.281 0.6 0.586 0.87

FADD TNFRSF1B 9606.ENSP00000301838 9606.ENSP00000365435 0 0 0 0 0 0 0 0.451 0.451

FADD IL6 9606.ENSP00000301838 9606.ENSP00000385675 0 0 0 0 0 0 0 0.459 0.459

FADD PTEN 9606.ENSP00000301838 9606.ENSP00000361021 0 0 0 0 0 0 0 0.486 0.486

FADD JUN 9606.ENSP00000301838 9606.ENSP00000360266 0 0 0 0 0 0 0 0.556 0.556

FADD LTA 9606.ENSP00000301838 9606.ENSP00000403495 0 0 0 0 0 0.124 0 0.529 0.57

FADD NOS1 9606.ENSP00000301838 9606.ENSP00000477999 0 0 0 0 0 0 0 0.626 0.626

FADD OCLN 9606.ENSP00000301838 9606.ENSP00000347379 0 0 0 0 0 0 0 0.721 0.721

FADD TLR4 9606.ENSP00000301838 9606.ENSP00000363089 0 0 0 0 0 0.056 0.6 0.504 0.796

FADD STAT1 9606.ENSP00000301838 9606.ENSP00000354394 0 0 0 0 0 0.213 0.9 0.527 0.959

FADD TNFAIP3 9606.ENSP00000301838 9606.ENSP00000481570 0 0 0 0 0 0.686 0.9 0.32 0.976

FADD TNF 9606.ENSP00000301838 9606.ENSP00000398698 0 0 0 0 0 0.345 0.9 0.952 0.996

FADD MYD88 9606.ENSP00000301838 9606.ENSP00000401399 0 0 0 0 0.096 0.631 0.8 0.974 0.998

FADD FASLG 9606.ENSP00000301838 9606.ENSP00000356694 0 0 0 0 0 0.87 0.9 0.988 0.999

FADD FAS 9606.ENSP00000301838 9606.ENSP00000347979 0 0 0 0 0 0.983 0.9 0.99 0.999

FAS TNFRSF1A 9606.ENSP00000347979 9606.ENSP00000162749 0 0 0 0 0.062 0.287 0.8 0.841 0.975

FAS NFKBIA 9606.ENSP00000347979 9606.ENSP00000216797 0 0 0 0 0.063 0 0 0.415 0.428

FAS IL2 9606.ENSP00000347979 9606.ENSP00000226730 0 0 0 0 0 0 0 0.7 0.7

FAS IFNG 9606.ENSP00000347979 9606.ENSP00000229135 0 0 0 0 0.06 0 0 0.627 0.634

FAS IL4 9606.ENSP00000347979 9606.ENSP00000231449 0 0 0 0 0 0 0 0.59 0.59

FAS HSPB1 9606.ENSP00000347979 9606.ENSP00000248553 0 0 0 0 0 0 0.9 0.271 0.924

FAS MDM2 9606.ENSP00000347979 9606.ENSP00000258149 0 0 0 0 0 0 0 0.401 0.4

FAS IL1B 9606.ENSP00000347979 9606.ENSP00000263341 0 0 0 0 0.097 0 0 0.535 0.562

FAS ICAM1 9606.ENSP00000347979 9606.ENSP00000264832 0 0 0 0 0.091 0 0 0.557 0.58

FAS TP53 9606.ENSP00000347979 9606.ENSP00000269305 0 0 0 0 0.063 0 0.9 0.682 0.967

FAS HSPA5 9606.ENSP00000347979 9606.ENSP00000324173 0 0 0 0 0 0.27 0 0.285 0.455

FAS STAT5A 9606.ENSP00000347979 9606.ENSP00000341208 0 0 0 0 0.062 0 0.9 0.333 0.932

FAS IL17A 9606.ENSP00000347979 9606.ENSP00000344192 0 0 0 0 0 0 0 0.55 0.55

FAS MYD88 9606.ENSP00000347979 9606.ENSP00000401399 0 0 0 0 0.081 0 0 0.378 0.404

FAS PAX5 9606.ENSP00000347979 9606.ENSP00000350844 0 0 0 0 0 0 0 0.415 0.415

FAS VEGFA 9606.ENSP00000347979 9606.ENSP00000478570 0 0 0 0 0 0 0 0.421 0.421

FAS LTA 9606.ENSP00000347979 9606.ENSP00000403495 0 0 0 0 0.062 0.059 0 0.402 0.426

FAS STAT1 9606.ENSP00000347979 9606.ENSP00000354394 0 0 0 0 0.087 0 0 0.404 0.433

FAS TLR4 9606.ENSP00000347979 9606.ENSP00000363089 0 0 0 0 0.079 0 0 0.446 0.468

FAS JUN 9606.ENSP00000347979 9606.ENSP00000360266 0 0 0 0 0 0 0 0.504 0.504

FAS TNFRSF1B 9606.ENSP00000347979 9606.ENSP00000365435 0 0 0 0 0.064 0 0 0.502 0.513

FAS ITGAL 9606.ENSP00000347979 9606.ENSP00000349252 0 0 0 0 0.062 0 0 0.509 0.519

FAS IL10 9606.ENSP00000347979 9606.ENSP00000412237 0 0 0 0 0 0 0 0.606 0.606

FAS IL6 9606.ENSP00000347979 9606.ENSP00000385675 0 0 0 0 0.083 0 0 0.602 0.619

FAS FOXP3 9606.ENSP00000347979 9606.ENSP00000365380 0 0 0 0 0 0 0 0.645 0.646

FAS TNF 9606.ENSP00000347979 9606.ENSP00000398698 0 0 0 0 0.089 0.283 0 0.753 0.825

FAS FASLG 9606.ENSP00000347979 9606.ENSP00000356694 0 0 0 0 0 0.87 0.9 0.989 0.999

FASLG TNFRSF1A 9606.ENSP00000356694 9606.ENSP00000162749 0 0 0 0 0 0.579 0 0.979 0.991

FASLG NFKBIA 9606.ENSP00000356694 9606.ENSP00000216797 0 0 0 0 0 0 0 0.459 0.459

FASLG MMP2 9606.ENSP00000356694 9606.ENSP00000219070 0 0 0 0 0 0 0 0.454 0.454

FASLG NFKB1 9606.ENSP00000356694 9606.ENSP00000226574 0 0 0 0 0 0.27 0 0.283 0.454

FASLG IL2 9606.ENSP00000356694 9606.ENSP00000226730 0 0 0 0 0.065 0 0 0.707 0.714

FASLG IFNG 9606.ENSP00000356694 9606.ENSP00000229135 0 0 0 0 0.261 0 0 0.594 0.687

FASLG IL4 9606.ENSP00000356694 9606.ENSP00000231449 0 0 0 0 0 0 0 0.611 0.611

FASLG HSPB1 9606.ENSP00000356694 9606.ENSP00000248553 0 0 0 0 0 0 0.9 0.27 0.923

FASLG IL1A 9606.ENSP00000356694 9606.ENSP00000263339 0 0 0 0 0 0 0 0.454 0.454

FASLG IL1B 9606.ENSP00000356694 9606.ENSP00000263341 0 0 0 0 0 0 0 0.632 0.632

FASLG ICAM1 9606.ENSP00000356694 9606.ENSP00000264832 0 0 0 0 0 0 0 0.625 0.625

FASLG TP53 9606.ENSP00000356694 9606.ENSP00000269305 0 0 0 0 0 0 0 0.681 0.681

FASLG NCF1 9606.ENSP00000356694 9606.ENSP00000289473 0 0 0 0 0.065 0.279 0 0.194 0.409

FASLG VCAM1 9606.ENSP00000356694 9606.ENSP00000294728 0 0 0 0 0 0 0 0.405 0.405

FASLG TLR3 9606.ENSP00000356694 9606.ENSP00000296795 0 0 0 0 0 0 0 0.43 0.43

FASLG IL13 9606.ENSP00000356694 9606.ENSP00000304915 0 0 0 0 0 0 0 0.503 0.503

FASLG STAT5A 9606.ENSP00000356694 9606.ENSP00000341208 0 0 0 0 0 0 0.9 0.375 0.934

FASLG IL17A 9606.ENSP00000356694 9606.ENSP00000344192 0 0 0 0 0 0 0 0.56 0.56

FASLG ITGAL 9606.ENSP00000356694 9606.ENSP00000349252 0 0 0 0 0.076 0 0 0.412 0.433

FASLG STAT1 9606.ENSP00000356694 9606.ENSP00000354394 0 0 0 0 0 0 0 0.464 0.463

FASLG HIF1A 9606.ENSP00000356694 9606.ENSP00000437955 0 0 0 0 0 0 0 0.407 0.407

FASLG IDO1 9606.ENSP00000356694 9606.ENSP00000430950 0 0 0 0 0 0 0 0.422 0.422

FASLG INS 9606.ENSP00000356694 9606.ENSP00000380432 0 0 0 0 0 0 0 0.427 0.426

FASLG PTEN 9606.ENSP00000356694 9606.ENSP00000361021 0 0 0 0 0 0 0 0.468 0.468

FASLG MMP9 9606.ENSP00000356694 9606.ENSP00000361405 0 0 0 0 0 0 0 0.469 0.469

FASLG TLR4 9606.ENSP00000356694 9606.ENSP00000363089 0 0 0 0 0.062 0 0 0.469 0.48

FASLG TNFRSF1B 9606.ENSP00000356694 9606.ENSP00000365435 0 0 0 0 0.076 0 0 0.519 0.536

FASLG VEGFA 9606.ENSP00000356694 9606.ENSP00000478570 0 0 0 0 0 0 0 0.594 0.594

FASLG JUN 9606.ENSP00000356694 9606.ENSP00000360266 0 0 0 0 0 0 0 0.601 0.601

FASLG FOXP3 9606.ENSP00000356694 9606.ENSP00000365380 0 0 0 0 0 0 0 0.62 0.62

FASLG IL6 9606.ENSP00000356694 9606.ENSP00000385675 0 0 0 0 0 0 0 0.656 0.656

FASLG IL10 9606.ENSP00000356694 9606.ENSP00000412237 0 0 0 0 0.062 0 0 0.684 0.69

FGF10 FGF23 9606.ENSP00000264664 9606.ENSP00000237837 0 0 0 0.66 0 0 0.5 0.514 0.583

FGF10 FGF2 9606.ENSP00000264664 9606.ENSP00000264498 0 0 0 0.723 0 0 0.5 0.794 0.608

FGF10 TNF 9606.ENSP00000264664 9606.ENSP00000398698 0 0 0 0 0 0 0 0.404 0.404

FGF10 IL6 9606.ENSP00000264664 9606.ENSP00000385675 0 0 0 0 0 0 0 0.428 0.428

FGF10 NGF 9606.ENSP00000264664 9606.ENSP00000358525 0 0 0 0 0 0 0 0.484 0.484

FGF10 RET 9606.ENSP00000264664 9606.ENSP00000347942 0 0 0 0 0.062 0.183 0 0.383 0.486

FGF10 LEF1 9606.ENSP00000264664 9606.ENSP00000265165 0 0 0 0 0 0.057 0 0.484 0.493

FGF10 VEGFA 9606.ENSP00000264664 9606.ENSP00000478570 0 0 0 0 0 0 0 0.537 0.537

FGF10 INSR 9606.ENSP00000264664 9606.ENSP00000303830 0 0 0 0 0 0 0.6 0.099 0.624

FGF10 INS 9606.ENSP00000264664 9606.ENSP00000380432 0 0 0 0 0 0 0 0.651 0.651

FGF10 NKX2-1 9606.ENSP00000264664 9606.ENSP00000346879 0 0 0 0 0 0 0 0.666 0.666

FGF10 IGF1 9606.ENSP00000264664 9606.ENSP00000302665 0 0 0 0 0 0 0 0.741 0.741

FGF10 KIT 9606.ENSP00000264664 9606.ENSP00000288135 0 0 0 0 0.065 0.183 0.6 0.311 0.761

FGF10 FGFR2 9606.ENSP00000264664 9606.ENSP00000410294 0 0 0 0 0 0.977 0.6 0.99 0.999

FGF2 TNFRSF1A 9606.ENSP00000264498 9606.ENSP00000162749 0 0 0 0 0.062 0 0 0.401 0.414

FGF2 HMOX1 9606.ENSP00000264498 9606.ENSP00000216117 0 0 0 0 0 0 0 0.45 0.45

FGF2 MMP2 9606.ENSP00000264498 9606.ENSP00000219070 0 0 0 0 0.088 0 0.9 0.691 0.969

FGF2 TGFB1 9606.ENSP00000264498 9606.ENSP00000221930 0 0 0 0 0 0 0.9 0.798 0.978

FGF2 IL2 9606.ENSP00000264498 9606.ENSP00000226730 0 0 0 0 0 0 0 0.601 0.601

FGF2 KITLG 9606.ENSP00000264498 9606.ENSP00000228280 0 0 0 0 0.078 0 0 0.707 0.718

FGF2 IFNG 9606.ENSP00000264498 9606.ENSP00000229135 0 0 0 0 0 0 0 0.735 0.735

FGF2 IL4 9606.ENSP00000264498 9606.ENSP00000231449 0 0 0 0 0.063 0 0 0.607 0.616

FGF2 FGF23 9606.ENSP00000264498 9606.ENSP00000237837 0 0 0 0.597 0 0 0.3 0.742 0.506

FGF2 IL1A 9606.ENSP00000264498 9606.ENSP00000263339 0 0 0 0 0.065 0 0 0.723 0.73

FGF2 IL1B 9606.ENSP00000264498 9606.ENSP00000263341 0 0 0 0 0 0 0 0.785 0.785

FGF2 SELP 9606.ENSP00000264498 9606.ENSP00000263686 0 0 0 0 0.062 0 0 0.567 0.577

FGF2 REN 9606.ENSP00000264498 9606.ENSP00000272190 0 0 0 0 0 0.059 0 0.391 0.403

FGF2 HPRT1 9606.ENSP00000264498 9606.ENSP00000298556 0 0 0 0 0 0 0 0.408 0.408

FGF2 LEF1 9606.ENSP00000264498 9606.ENSP00000265165 0 0 0 0 0 0.057 0 0.403 0.413

FGF2 SST 9606.ENSP00000264498 9606.ENSP00000287641 0 0 0 0 0 0 0 0.414 0.414

FGF2 HSPA5 9606.ENSP00000264498 9606.ENSP00000324173 0 0 0 0 0 0.062 0 0.411 0.424

FGF2 OCLN 9606.ENSP00000264498 9606.ENSP00000347379 0 0 0 0 0 0 0 0.43 0.43

FGF2 NKX2-1 9606.ENSP00000264498 9606.ENSP00000346879 0 0 0 0 0 0 0 0.432 0.432

FGF2 TLR4 9606.ENSP00000264498 9606.ENSP00000363089 0 0 0 0 0.065 0 0 0.431 0.445

FGF2 STAT1 9606.ENSP00000264498 9606.ENSP00000354394 0 0 0 0 0 0 0 0.494 0.494

FGF2 MTOR 9606.ENSP00000264498 9606.ENSP00000354558 0 0 0 0 0 0 0 0.506 0.506

FGF2 GDF5 9606.ENSP00000264498 9606.ENSP00000363492 0 0 0 0 0 0 0 0.536 0.536

FGF2 LTA 9606.ENSP00000264498 9606.ENSP00000403495 0 0 0 0 0 0 0 0.554 0.554

FGF2 PTEN 9606.ENSP00000264498 9606.ENSP00000361021 0 0 0 0 0 0 0 0.577 0.577

FGF2 IL13 9606.ENSP00000264498 9606.ENSP00000304915 0 0 0 0 0 0 0 0.6 0.6

FGF2 TH 9606.ENSP00000264498 9606.ENSP00000370571 0 0 0 0 0 0 0 0.602 0.602

FGF2 ICAM1 9606.ENSP00000264498 9606.ENSP00000264832 0 0 0 0 0 0 0 0.604 0.604

FGF2 SELE 9606.ENSP00000264498 9606.ENSP00000331736 0 0 0 0 0.062 0 0 0.595 0.604

FGF2 JUN 9606.ENSP00000264498 9606.ENSP00000360266 0 0 0 0 0 0 0 0.605 0.605

FGF2 LEP 9606.ENSP00000264498 9606.ENSP00000312652 0 0 0 0 0 0 0 0.607 0.607

FGF2 VCAM1 9606.ENSP00000264498 9606.ENSP00000294728 0 0 0 0 0 0 0 0.615 0.615

FGF2 NOS3 9606.ENSP00000264498 9606.ENSP00000297494 0 0 0 0 0.062 0 0 0.61 0.618

FGF2 SPP1 9606.ENSP00000264498 9606.ENSP00000378517 0 0 0 0 0 0 0 0.64 0.64

FGF2 RET 9606.ENSP00000264498 9606.ENSP00000347942 0 0 0 0 0 0.407 0 0.43 0.647

FGF2 PPARG 9606.ENSP00000264498 9606.ENSP00000287820 0 0 0 0 0 0 0 0.664 0.664

FGF2 INSR 9606.ENSP00000264498 9606.ENSP00000303830 0 0 0 0 0 0 0.6 0.212 0.671

FGF2 TP53 9606.ENSP00000264498 9606.ENSP00000269305 0 0 0 0 0 0 0 0.674 0.674

FGF2 HIF1A 9606.ENSP00000264498 9606.ENSP00000437955 0 0 0 0 0 0 0 0.703 0.703

FGF2 TNF 9606.ENSP00000264498 9606.ENSP00000398698 0 0 0 0 0 0 0 0.741 0.741

FGF2 PF4 9606.ENSP00000264498 9606.ENSP00000296029 0 0 0 0 0 0.213 0 0.723 0.773

FGF2 NGF 9606.ENSP00000264498 9606.ENSP00000358525 0 0 0 0 0.104 0 0 0.846 0.857

FGF2 INS 9606.ENSP00000264498 9606.ENSP00000380432 0 0 0 0 0 0 0 0.874 0.874

FGF2 KIT 9606.ENSP00000264498 9606.ENSP00000288135 0 0 0 0 0 0.183 0.6 0.694 0.891

FGF2 LCN2 9606.ENSP00000264498 9606.ENSP00000362108 0 0 0 0 0 0 0.9 0.283 0.925

FGF2 MMP1 9606.ENSP00000264498 9606.ENSP00000322788 0 0 0 0 0 0 0.9 0.61 0.959

FGF2 MMP3 9606.ENSP00000264498 9606.ENSP00000299855 0 0 0 0 0.072 0 0.9 0.607 0.96

FGF2 IL17A 9606.ENSP00000264498 9606.ENSP00000344192 0 0 0 0 0 0 0.9 0.646 0.963

FGF2 IL10 9606.ENSP00000264498 9606.ENSP00000412237 0 0 0 0 0 0 0.9 0.677 0.966

FGF2 MMP9 9606.ENSP00000264498 9606.ENSP00000361405 0 0 0 0 0 0 0.9 0.754 0.974

FGF2 IL6 9606.ENSP00000264498 9606.ENSP00000385675 0 0 0 0 0.061 0 0.9 0.764 0.975

FGF2 IGF1 9606.ENSP00000264498 9606.ENSP00000302665 0 0 0 0 0 0 0 0.976 0.976

FGF2 VEGFA 9606.ENSP00000264498 9606.ENSP00000478570 0 0 0 0 0 0 0.9 0.923 0.991

FGF2 FGFR2 9606.ENSP00000264498 9606.ENSP00000410294 0 0 0 0 0 0.942 0.8 0.99 0.999

FGF23 KITLG 9606.ENSP00000237837 9606.ENSP00000228280 0 0 0 0 0 0 0 0.404 0.404

FGF23 TNF 9606.ENSP00000237837 9606.ENSP00000398698 0 0 0 0 0 0 0 0.406 0.406

FGF23 NGF 9606.ENSP00000237837 9606.ENSP00000358525 0 0 0 0 0 0 0 0.418 0.418

FGF23 LCN2 9606.ENSP00000237837 9606.ENSP00000362108 0 0 0 0 0 0 0 0.501 0.501

FGF23 LEP 9606.ENSP00000237837 9606.ENSP00000312652 0 0 0 0 0 0 0 0.501 0.501

FGF23 IL6 9606.ENSP00000237837 9606.ENSP00000385675 0 0 0 0 0 0 0 0.504 0.504

FGF23 FURIN 9606.ENSP00000237837 9606.ENSP00000483552 0 0 0 0 0 0 0 0.517 0.517

FGF23 INS 9606.ENSP00000237837 9606.ENSP00000380432 0 0 0 0 0 0 0 0.605 0.605

FGF23 SPP1 9606.ENSP00000237837 9606.ENSP00000378517 0 0 0 0 0 0 0 0.617 0.617

FGF23 INSR 9606.ENSP00000237837 9606.ENSP00000303830 0 0 0 0 0.053 0 0.6 0.166 0.656

FGF23 IGF1 9606.ENSP00000237837 9606.ENSP00000302665 0 0 0 0 0.062 0 0 0.669 0.676

FGF23 KIT 9606.ENSP00000237837 9606.ENSP00000288135 0 0 0 0 0 0.183 0.6 0.145 0.696

FGF23 VDR 9606.ENSP00000237837 9606.ENSP00000447173 0 0 0 0 0 0 0 0.802 0.802

FGF23 REN 9606.ENSP00000237837 9606.ENSP00000272190 0 0 0 0 0.062 0.059 0 0.872 0.878

FGF23 FGFR2 9606.ENSP00000237837 9606.ENSP00000410294 0 0 0 0 0.049 0.659 0.9 0.835 0.993

FGFR2 KITLG 9606.ENSP00000410294 9606.ENSP00000228280 0 0 0 0 0 0.067 0.6 0.244 0.693

FGFR2 NOTCH2 9606.ENSP00000410294 9606.ENSP00000256646 0 0 0 0 0.062 0.076 0 0.414 0.448

FGFR2 MDM2 9606.ENSP00000410294 9606.ENSP00000258149 0 0 0 0 0 0 0.6 0.391 0.745

FGFR2 TP53 9606.ENSP00000410294 9606.ENSP00000269305 0 0 0 0 0 0 0 0.635 0.635

FGFR2 KIT 9606.ENSP00000410294 9606.ENSP00000288135 0 0 0 0.721 0.064 0.39 0 0.606 0.502

FGFR2 IGF1 9606.ENSP00000410294 9606.ENSP00000302665 0 0 0 0 0 0 0.6 0.496 0.789

FGFR2 PLCB1 9606.ENSP00000410294 9606.ENSP00000338185 0 0 0 0 0.062 0.082 0.6 0.08 0.64

FGFR2 STAT5A 9606.ENSP00000410294 9606.ENSP00000341208 0 0 0 0 0.061 0.323 0 0.57 0.703

FGFR2 NKX2-1 9606.ENSP00000410294 9606.ENSP00000346879 0 0 0 0 0.055 0.065 0 0.421 0.443

FGFR2 RET 9606.ENSP00000410294 9606.ENSP00000347942 0 0 0 0.751 0 0.388 0 0.761 0.502

FGFR2 STAT1 9606.ENSP00000410294 9606.ENSP00000354394 0 0 0 0 0 0.111 0.9 0.23 0.925

FGFR2 NGF 9606.ENSP00000410294 9606.ENSP00000358525 0 0 0 0 0 0 0.6 0.239 0.682

FGFR2 JUN 9606.ENSP00000410294 9606.ENSP00000360266 0 0 0 0 0 0.112 0.9 0.34 0.936

FGFR2 PTEN 9606.ENSP00000410294 9606.ENSP00000361021 0 0 0 0 0 0.112 0 0.651 0.676

FGFR2 HDAC1 9606.ENSP00000410294 9606.ENSP00000362649 0 0 0 0 0.062 0 0 0.455 0.467

FGFR2 INS 9606.ENSP00000410294 9606.ENSP00000380432 0 0 0 0 0 0 0.8 0.404 0.875

FGFR2 VEGFA 9606.ENSP00000410294 9606.ENSP00000478570 0 0 0 0 0.063 0.059 0.6 0.556 0.822

FOXP3 TNFRSF1A 9606.ENSP00000365380 9606.ENSP00000162749 0 0 0 0 0 0 0 0.539 0.539

FOXP3 HMOX1 9606.ENSP00000365380 9606.ENSP00000216117 0 0 0 0 0 0 0 0.439 0.439

FOXP3 NFKBIA 9606.ENSP00000365380 9606.ENSP00000216797 0 0 0 0 0 0.078 0 0.394 0.417

FOXP3 TGFB1 9606.ENSP00000365380 9606.ENSP00000221930 0 0 0 0 0.062 0.182 0 0.698 0.748

FOXP3 MPO 9606.ENSP00000365380 9606.ENSP00000225275 0 0 0 0 0 0 0 0.463 0.463

FOXP3 NFKB1 9606.ENSP00000365380 9606.ENSP00000226574 0 0 0 0 0 0.373 0 0.456 0.644

FOXP3 IL2 9606.ENSP00000365380 9606.ENSP00000226730 0 0 0 0 0 0.848 0.9 0.88 0.998

FOXP3 IFNG 9606.ENSP00000365380 9606.ENSP00000229135 0 0 0 0 0.06 0.345 0.9 0.853 0.989

FOXP3 IL4 9606.ENSP00000365380 9606.ENSP00000231449 0 0 0 0 0 0 0 0.874 0.874

FOXP3 IRF1 9606.ENSP00000365380 9606.ENSP00000245414 0 0 0 0 0 0.336 0 0.451 0.62

FOXP3 SMAD7 9606.ENSP00000365380 9606.ENSP00000262158 0 0 0 0 0 0.181 0 0.401 0.488

FOXP3 IL1A 9606.ENSP00000365380 9606.ENSP00000263339 0 0 0 0 0 0 0 0.518 0.518

FOXP3 IL1B 9606.ENSP00000365380 9606.ENSP00000263341 0 0 0 0 0 0 0 0.739 0.739

FOXP3 SELP 9606.ENSP00000365380 9606.ENSP00000263686 0 0 0 0 0 0.056 0 0.426 0.434

FOXP3 ICAM1 9606.ENSP00000365380 9606.ENSP00000264832 0 0 0 0 0 0 0 0.575 0.575

FOXP3 LEF1 9606.ENSP00000365380 9606.ENSP00000265165 0 0 0 0 0 0.162 0 0.474 0.54

FOXP3 TP53 9606.ENSP00000365380 9606.ENSP00000269305 0 0 0 0 0 0.182 0 0.503 0.576

FOXP3 PPARG 9606.ENSP00000365380 9606.ENSP00000287820 0 0 0 0 0 0.182 0 0.953 0.96

FOXP3 KIT 9606.ENSP00000365380 9606.ENSP00000288135 0 0 0 0 0 0.058 0 0.521 0.53

FOXP3 VCAM1 9606.ENSP00000365380 9606.ENSP00000294728 0 0 0 0 0 0 0 0.485 0.485

FOXP3 TLR3 9606.ENSP00000365380 9606.ENSP00000296795 0 0 0 0 0 0.058 0 0.534 0.542

FOXP3 HPRT1 9606.ENSP00000365380 9606.ENSP00000298556 0 0 0 0 0 0 0 0.531 0.531

FOXP3 NOD2 9606.ENSP00000365380 9606.ENSP00000300589 0 0 0 0 0.076 0 0 0.469 0.488

FOXP3 IL13 9606.ENSP00000365380 9606.ENSP00000304915 0 0 0 0 0 0 0 0.778 0.778

FOXP3 IL17RA 9606.ENSP00000365380 9606.ENSP00000320936 0 0 0 0 0 0.182 0 0.477 0.554

FOXP3 HSPA5 9606.ENSP00000365380 9606.ENSP00000324173 0 0 0 0 0 0.056 0 0.453 0.461

FOXP3 NOS2 9606.ENSP00000365380 9606.ENSP00000327251 0 0 0 0 0 0.058 0 0.458 0.467

FOXP3 SOCS1 9606.ENSP00000365380 9606.ENSP00000329418 0 0 0 0 0 0 0 0.635 0.635

FOXP3 STAT5A 9606.ENSP00000365380 9606.ENSP00000341208 0 0 0 0 0 0.151 0 0.87 0.885

FOXP3 USP7 9606.ENSP00000365380 9606.ENSP00000343535 0 0 0 0 0 0.282 0 0.825 0.869

FOXP3 IL17A 9606.ENSP00000365380 9606.ENSP00000344192 0 0 0 0 0 0 0 0.905 0.905

FOXP3 ITGAL 9606.ENSP00000365380 9606.ENSP00000349252 0 0 0 0 0.07 0 0 0.47 0.486

FOXP3 STAT1 9606.ENSP00000365380 9606.ENSP00000354394 0 0 0 0 0 0.377 0 0.659 0.779

FOXP3 MTOR 9606.ENSP00000365380 9606.ENSP00000354558 0 0 0 0 0 0.133 0 0.56 0.603

FOXP3 JUN 9606.ENSP00000365380 9606.ENSP00000360266 0 0 0 0 0 0.078 0.9 0.873 0.987

FOXP3 PTEN 9606.ENSP00000365380 9606.ENSP00000361021 0 0 0 0 0 0.09 0 0.559 0.583

FOXP3 MMP9 9606.ENSP00000365380 9606.ENSP00000361405 0 0 0 0 0.064 0 0 0.466 0.478

FOXP3 HDAC1 9606.ENSP00000365380 9606.ENSP00000362649 0 0 0 0 0 0.592 0 0.77 0.902

FOXP3 TLR4 9606.ENSP00000365380 9606.ENSP00000363089 0 0 0 0 0 0.058 0 0.682 0.687

FOXP3 VDR 9606.ENSP00000365380 9606.ENSP00000447173 0 0 0 0 0 0.062 0 0.403 0.416

FOXP3 TNFRSF1B 9606.ENSP00000365380 9606.ENSP00000365435 0 0 0 0 0 0 0 0.503 0.503

FOXP3 LTA 9606.ENSP00000365380 9606.ENSP00000403495 0 0 0 0 0.072 0 0 0.498 0.514

FOXP3 VEGFA 9606.ENSP00000365380 9606.ENSP00000478570 0 0 0 0 0 0 0 0.601 0.601

FOXP3 INS 9606.ENSP00000365380 9606.ENSP00000380432 0 0 0 0 0 0 0 0.602 0.602

FOXP3 MYD88 9606.ENSP00000365380 9606.ENSP00000401399 0 0 0 0 0 0 0 0.606 0.606

FOXP3 IDO1 9606.ENSP00000365380 9606.ENSP00000430950 0 0 0 0 0 0 0 0.716 0.716

FOXP3 IL6 9606.ENSP00000365380 9606.ENSP00000385675 0 0 0 0 0 0.182 0 0.846 0.868

FOXP3 TNF 9606.ENSP00000365380 9606.ENSP00000398698 0 0 0 0 0 0.27 0 0.839 0.878

FOXP3 IL10 9606.ENSP00000365380 9606.ENSP00000412237 0 0 0 0 0 0 0 0.916 0.916

FOXP3 HIF1A 9606.ENSP00000365380 9606.ENSP00000437955 0 0 0 0 0 0.075 0 0.964 0.965

FURIN MMP2 9606.ENSP00000483552 9606.ENSP00000219070 0 0 0 0 0 0 0 0.478 0.478

FURIN TGFB1 9606.ENSP00000483552 9606.ENSP00000221930 0 0 0 0 0.099 0 0.9 0.403 0.941

FURIN NOTCH2 9606.ENSP00000483552 9606.ENSP00000256646 0 0 0 0 0.086 0 0.9 0.425 0.942

FURIN REN 9606.ENSP00000483552 9606.ENSP00000272190 0 0 0 0 0.093 0.244 0 0.449 0.589

FURIN MMP3 9606.ENSP00000483552 9606.ENSP00000299855 0 0 0 0 0 0 0 0.464 0.464

FURIN IGF1 9606.ENSP00000483552 9606.ENSP00000302665 0 0 0 0 0 0 0 0.54 0.54

FURIN LPL 9606.ENSP00000483552 9606.ENSP00000309757 0 0 0 0 0 0 0.9 0.205 0.917

FURIN HSPA5 9606.ENSP00000483552 9606.ENSP00000324173 0 0 0 0 0.353 0.056 0 0.339 0.561

FURIN P4HB 9606.ENSP00000483552 9606.ENSP00000327801 0 0 0 0 0.263 0.059 0 0.262 0.444

FURIN NGF 9606.ENSP00000483552 9606.ENSP00000358525 0 0 0 0 0 0.057 0.9 0.48 0.946

FURIN MMP9 9606.ENSP00000483552 9606.ENSP00000361405 0 0 0 0 0.072 0 0 0.501 0.517

FURIN INS 9606.ENSP00000483552 9606.ENSP00000380432 0 0 0 0 0 0 0 0.502 0.502

FURIN TNF 9606.ENSP00000483552 9606.ENSP00000398698 0 0 0 0 0 0 0 0.472 0.472

FURIN VEGFA 9606.ENSP00000483552 9606.ENSP00000478570 0 0 0 0 0 0 0 0.401 0.4

GDF5 MMP3 9606.ENSP00000363492 9606.ENSP00000299855 0 0 0 0 0.069 0 0 0.392 0.409

GDF5 IGF1 9606.ENSP00000363492 9606.ENSP00000302665 0 0 0 0 0 0 0 0.524 0.524

GPT HMOX1 9606.ENSP00000378408 9606.ENSP00000216117 0 0 0 0 0 0 0 0.672 0.672

GPT NFKBIA 9606.ENSP00000378408 9606.ENSP00000216797 0 0 0 0 0 0.058 0 0.451 0.46

GPT TGFB1 9606.ENSP00000378408 9606.ENSP00000221930 0 0 0 0 0 0 0 0.504 0.504

GPT PON1 9606.ENSP00000378408 9606.ENSP00000222381 0 0 0 0 0.062 0 0 0.391 0.404

GPT OGDH 9606.ENSP00000378408 9606.ENSP00000222673 0 0 0 0 0.13 0 0.8 0.191 0.847

GPT MPO 9606.ENSP00000378408 9606.ENSP00000225275 0 0 0 0 0.064 0 0 0.651 0.659

GPT IL2 9606.ENSP00000378408 9606.ENSP00000226730 0 0 0 0 0 0 0 0.556 0.556

GPT IFNG 9606.ENSP00000378408 9606.ENSP00000229135 0 0 0 0 0 0 0 0.564 0.564

GPT IL4 9606.ENSP00000378408 9606.ENSP00000231449 0 0 0 0 0 0 0 0.507 0.507

GPT MDM2 9606.ENSP00000378408 9606.ENSP00000258149 0 0 0 0 0 0 0 0.403 0.403

GPT IL1B 9606.ENSP00000378408 9606.ENSP00000263341 0 0 0 0 0 0 0 0.69 0.69

GPT ICAM1 9606.ENSP00000378408 9606.ENSP00000264832 0 0 0 0 0 0 0 0.501 0.501

GPT TP53 9606.ENSP00000378408 9606.ENSP00000269305 0 0 0 0 0 0 0 0.468 0.468

GPT REN 9606.ENSP00000378408 9606.ENSP00000272190 0 0 0 0 0 0 0 0.5 0.499

GPT PPARG 9606.ENSP00000378408 9606.ENSP00000287820 0 0 0 0 0 0.056 0 0.602 0.608

GPT HMGCR 9606.ENSP00000378408 9606.ENSP00000287936 0 0 0 0 0 0 0 0.471 0.471

GPT NOS3 9606.ENSP00000378408 9606.ENSP00000297494 0 0 0 0 0.063 0 0 0.469 0.481

GPT IGF1 9606.ENSP00000378408 9606.ENSP00000302665 0 0 0 0 0 0 0 0.493 0.493

GPT LPL 9606.ENSP00000378408 9606.ENSP00000309757 0 0 0 0 0 0 0 0.466 0.465

GPT GSTM1 9606.ENSP00000378408 9606.ENSP00000311469 0 0 0 0 0.056 0.178 0 0.324 0.429

GPT LEP 9606.ENSP00000378408 9606.ENSP00000312652 0 0 0 0 0 0 0 0.681 0.681

GPT HSPA5 9606.ENSP00000378408 9606.ENSP00000324173 0 0 0 0 0 0.058 0 0.418 0.429

GPT NOS2 9606.ENSP00000378408 9606.ENSP00000327251 0 0 0 0 0.063 0 0 0.394 0.408

GPT IL17A 9606.ENSP00000378408 9606.ENSP00000344192 0 0 0 0 0 0 0 0.466 0.465

GPT SREBF1 9606.ENSP00000378408 9606.ENSP00000348069 0 0 0 0 0.083 0 0 0.654 0.669

GPT MTOR 9606.ENSP00000378408 9606.ENSP00000354558 0 0 0 0 0 0 0 0.413 0.412

GPT JUN 9606.ENSP00000378408 9606.ENSP00000360266 0 0 0 0 0 0 0 0.508 0.508

GPT MMP9 9606.ENSP00000378408 9606.ENSP00000361405 0 0 0 0 0.049 0 0 0.405 0.409

GPT TLR4 9606.ENSP00000378408 9606.ENSP00000363089 0 0 0 0 0 0.057 0 0.601 0.607

GPT SHBG 9606.ENSP00000378408 9606.ENSP00000369816 0 0 0 0 0.09 0 0 0.508 0.533

GPT PC 9606.ENSP00000378408 9606.ENSP00000377532 0.048 0 0 0 0.162 0 0 0.445 0.519

GPT MTTP 9606.ENSP00000378408 9606.ENSP00000427679 0 0 0 0 0.064 0 0 0.486 0.498

GPT VEGFA 9606.ENSP00000378408 9606.ENSP00000478570 0 0 0 0 0 0 0 0.518 0.518

GPT IL10 9606.ENSP00000378408 9606.ENSP00000412237 0 0 0 0 0 0 0 0.669 0.669

GPT TNF 9606.ENSP00000378408 9606.ENSP00000398698 0 0 0 0 0 0 0 0.758 0.758

GPT IL6 9606.ENSP00000378408 9606.ENSP00000385675 0 0 0 0 0 0 0 0.769 0.769

GPT INS 9606.ENSP00000378408 9606.ENSP00000380432 0 0 0 0 0 0 0 0.857 0.857

GRIN2B SLC6A4 9606.ENSP00000477455 9606.ENSP00000261707 0 0 0 0 0 0 0 0.527 0.527

GRIN2B IL1B 9606.ENSP00000477455 9606.ENSP00000263341 0 0 0 0 0 0 0 0.575 0.575

GRIN2B SST 9606.ENSP00000477455 9606.ENSP00000287641 0 0 0 0 0.111 0 0 0.381 0.426

GRIN2B NOS3 9606.ENSP00000477455 9606.ENSP00000297494 0 0 0 0 0.056 0.317 0 0.181 0.425

GRIN2B SCN1A 9606.ENSP00000477455 9606.ENSP00000303540 0 0 0 0 0.174 0.057 0 0.474 0.554

GRIN2B PRKCB 9606.ENSP00000477455 9606.ENSP00000305355 0 0 0 0 0.086 0.436 0 0.133 0.515

GRIN2B ITPR1 9606.ENSP00000477455 9606.ENSP00000306253 0 0 0 0 0.062 0.067 0 0.43 0.458

GRIN2B PPARD 9606.ENSP00000477455 9606.ENSP00000310928 0 0 0 0 0 0 0 0.426 0.426

GRIN2B KCND2 9606.ENSP00000477455 9606.ENSP00000333496 0 0 0 0 0.196 0 0 0.555 0.627

GRIN2B SNCA 9606.ENSP00000477455 9606.ENSP00000338345 0 0 0 0 0.088 0 0 0.392 0.422

GRIN2B MTOR 9606.ENSP00000477455 9606.ENSP00000354558 0 0 0 0 0 0.057 0 0.418 0.427

GRIN2B NGF 9606.ENSP00000477455 9606.ENSP00000358525 0 0 0 0 0 0 0 0.469 0.469

GRIN2B PTEN 9606.ENSP00000477455 9606.ENSP00000361021 0 0 0 0 0 0.065 0 0.513 0.525

GRIN2B TH 9606.ENSP00000477455 9606.ENSP00000370571 0 0 0 0 0.062 0 0 0.447 0.459

GRIN2B KCNC3 9606.ENSP00000477455 9606.ENSP00000434241 0 0 0 0 0.082 0 0 0.51 0.531

GRIN2B HTR2A 9606.ENSP00000477455 9606.ENSP00000437737 0 0 0 0 0.136 0.057 0 0.556 0.606

GRIN2B NOS1 9606.ENSP00000477455 9606.ENSP00000477999 0 0 0 0 0.096 0.393 0.8 0.943 0.993

GSTM1 HMOX1 9606.ENSP00000311469 9606.ENSP00000216117 0 0 0 0 0 0 0 0.552 0.552

GSTM1 PON1 9606.ENSP00000311469 9606.ENSP00000222381 0 0 0 0 0.062 0 0 0.517 0.527

GSTM1 MPO 9606.ENSP00000311469 9606.ENSP00000225275 0 0 0 0 0 0 0 0.467 0.467

GSTM1 TP53 9606.ENSP00000311469 9606.ENSP00000269305 0 0 0 0 0 0 0 0.556 0.556

GSTM1 TNF 9606.ENSP00000311469 9606.ENSP00000398698 0 0 0 0 0 0 0 0.407 0.407

GSTM1 SPP1 9606.ENSP00000311469 9606.ENSP00000378517 0 0 0 0 0 0 0 0.932 0.932

HBA1 HBA2 9606.ENSP00000322421 9606.ENSP00000251595 0 0 0 0.985 0.771 0.8 0.9 0.181 0.995

HBA1 ORM1 9606.ENSP00000322421 9606.ENSP00000259396 0 0 0 0 0.124 0 0 0.392 0.445

HBA1 LYZ 9606.ENSP00000322421 9606.ENSP00000261267 0 0 0 0 0.14 0 0 0.344 0.412

HBA1 INS 9606.ENSP00000322421 9606.ENSP00000380432 0 0 0 0 0 0 0 0.739 0.739

HBA2 LYZ 9606.ENSP00000251595 9606.ENSP00000261267 0 0 0 0 0.139 0 0 0.345 0.412

HBA2 INS 9606.ENSP00000251595 9606.ENSP00000380432 0 0 0 0 0 0 0 0.739 0.739

HDAC1 NFKBIA 9606.ENSP00000362649 9606.ENSP00000216797 0 0 0 0 0.062 0.499 0.9 0.825 0.99

HDAC1 NFKB1 9606.ENSP00000362649 9606.ENSP00000226574 0 0 0 0 0.074 0.894 0 0.971 0.996

HDAC1 IL2 9606.ENSP00000362649 9606.ENSP00000226730 0 0 0 0 0 0.486 0 0.376 0.666

HDAC1 NR3C1 9606.ENSP00000362649 9606.ENSP00000231509 0 0 0 0 0 0.859 0 0.586 0.939

HDAC1 IRF1 9606.ENSP00000362649 9606.ENSP00000245414 0 0 0 0 0 0 0.8 0.697 0.936

HDAC1 MDM2 9606.ENSP00000362649 9606.ENSP00000258149 0 0 0 0 0 0.675 0 0.979 0.993

HDAC1 SMAD7 9606.ENSP00000362649 9606.ENSP00000262158 0 0 0 0 0.054 0.494 0.9 0.832 0.99

HDAC1 LEF1 9606.ENSP00000362649 9606.ENSP00000265165 0 0 0 0 0.064 0.336 0.9 0.953 0.996

HDAC1 TP53 9606.ENSP00000362649 9606.ENSP00000269305 0 0 0 0 0.062 0.927 0.9 0.989 0.999

HDAC1 PPARG 9606.ENSP00000362649 9606.ENSP00000287820 0 0 0 0 0 0.505 0.65 0.622 0.929

HDAC1 PPARD 9606.ENSP00000362649 9606.ENSP00000310928 0 0 0 0 0 0.505 0 0.129 0.551

HDAC1 STAT5A 9606.ENSP00000362649 9606.ENSP00000341208 0 0 0 0 0.048 0.401 0.9 0.666 0.978

HDAC1 USP7 9606.ENSP00000362649 9606.ENSP00000343535 0 0 0 0 0.097 0.486 0 0.575 0.786

HDAC1 IL17A 9606.ENSP00000362649 9606.ENSP00000344192 0 0 0 0 0 0.27 0 0.246 0.426

HDAC1 STAT1 9606.ENSP00000362649 9606.ENSP00000354394 0 0 0 0 0.062 0.316 0 0.938 0.957

HDAC1 TOP1 9606.ENSP00000362649 9606.ENSP00000354522 0 0 0 0 0.106 0.221 0 0.299 0.47

HDAC1 MTOR 9606.ENSP00000362649 9606.ENSP00000354558 0 0 0 0 0.062 0.103 0 0.372 0.425

HDAC1 PARP1 9606.ENSP00000362649 9606.ENSP00000355759 0 0 0 0 0.125 0.682 0 0.572 0.87

HDAC1 JUN 9606.ENSP00000362649 9606.ENSP00000360266 0 0 0 0 0 0.476 0.65 0.791 0.958

HDAC1 PTEN 9606.ENSP00000362649 9606.ENSP00000361021 0 0 0 0 0.062 0.061 0 0.525 0.545

HDAC1 VEGFA 9606.ENSP00000362649 9606.ENSP00000478570 0 0 0 0 0 0 0 0.42 0.42

HDAC1 INS 9606.ENSP00000362649 9606.ENSP00000380432 0 0 0 0 0 0 0 0.45 0.45

HDAC1 VDR 9606.ENSP00000362649 9606.ENSP00000447173 0 0 0 0 0 0.298 0 0.252 0.452

HDAC1 IL6 9606.ENSP00000362649 9606.ENSP00000385675 0 0 0 0 0 0 0 0.474 0.474

HDAC1 TNF 9606.ENSP00000362649 9606.ENSP00000398698 0 0 0 0 0 0.067 0 0.488 0.502

HDAC1 PCNA 9606.ENSP00000362649 9606.ENSP00000368458 0 0 0 0 0.186 0.486 0 0.063 0.574

HDAC1 HELLS 9606.ENSP00000362649 9606.ENSP00000377601 0 0 0 0 0.062 0.551 0 0.276 0.668

HDAC1 HIF1A 9606.ENSP00000362649 9606.ENSP00000437955 0 0 0 0 0.05 0.759 0 0.978 0.994

HELLS TP53 9606.ENSP00000377601 9606.ENSP00000269305 0 0 0 0 0.107 0.281 0 0.254 0.48

HELLS PCNA 9606.ENSP00000377601 9606.ENSP00000368458 0.058 0 0 0 0.357 0.244 0 0.163 0.566

HIF1A MIF 9606.ENSP00000437955 9606.ENSP00000215754 0 0 0 0 0 0.27 0 0.458 0.587

HIF1A HMOX1 9606.ENSP00000437955 9606.ENSP00000216117 0 0 0 0 0 0 0 0.697 0.697

HIF1A NFKBIA 9606.ENSP00000437955 9606.ENSP00000216797 0 0 0 0 0 0 0 0.556 0.556

HIF1A MMP2 9606.ENSP00000437955 9606.ENSP00000219070 0 0 0 0 0 0 0 0.754 0.754

HIF1A TGFB1 9606.ENSP00000437955 9606.ENSP00000221930 0 0 0 0 0 0.209 0 0.581 0.654

HIF1A NFKB1 9606.ENSP00000437955 9606.ENSP00000226574 0 0 0 0 0.062 0 0 0.426 0.439

HIF1A IL2 9606.ENSP00000437955 9606.ENSP00000226730 0 0 0 0 0 0 0 0.505 0.505

HIF1A KITLG 9606.ENSP00000437955 9606.ENSP00000228280 0 0 0 0 0 0 0 0.457 0.457

HIF1A IFNG 9606.ENSP00000437955 9606.ENSP00000229135 0 0 0 0 0 0 0 0.524 0.524

HIF1A IL4 9606.ENSP00000437955 9606.ENSP00000231449 0 0 0 0 0 0 0 0.522 0.522

HIF1A NR3C1 9606.ENSP00000437955 9606.ENSP00000231509 0 0 0 0 0 0.108 0.65 0.656 0.883

HIF1A IRF1 9606.ENSP00000437955 9606.ENSP00000245414 0 0 0 0 0 0 0 0.697 0.697

HIF1A HSPB1 9606.ENSP00000437955 9606.ENSP00000248553 0 0 0 0 0 0 0 0.462 0.462

HIF1A NOTCH2 9606.ENSP00000437955 9606.ENSP00000256646 0 0 0 0 0.065 0 0 0.441 0.455

HIF1A MDM2 9606.ENSP00000437955 9606.ENSP00000258149 0 0 0 0 0.062 0.873 0.9 0.984 0.999

HIF1A SMAD7 9606.ENSP00000437955 9606.ENSP00000262158 0 0 0 0 0 0.056 0 0.528 0.535

HIF1A IL1A 9606.ENSP00000437955 9606.ENSP00000263339 0 0 0 0 0 0 0 0.456 0.456

HIF1A IL1B 9606.ENSP00000437955 9606.ENSP00000263341 0 0 0 0 0 0 0 0.724 0.724

HIF1A ICAM1 9606.ENSP00000437955 9606.ENSP00000264832 0 0 0 0 0 0.273 0 0.544 0.654

HIF1A LEF1 9606.ENSP00000437955 9606.ENSP00000265165 0 0 0 0 0 0.149 0 0.385 0.454

HIF1A TP53 9606.ENSP00000437955 9606.ENSP00000269305 0 0 0 0 0 0.87 0.9 0.989 0.999

HIF1A SOD1 9606.ENSP00000437955 9606.ENSP00000270142 0 0 0 0 0 0 0 0.464 0.464

HIF1A REN 9606.ENSP00000437955 9606.ENSP00000272190 0 0 0 0 0 0 0 0.484 0.484

HIF1A PLOD2 9606.ENSP00000437955 9606.ENSP00000282903 0 0 0 0 0.085 0 0 0.476 0.5

HIF1A PPARG 9606.ENSP00000437955 9606.ENSP00000287820 0 0 0 0 0.062 0.108 0 0.736 0.76

HIF1A KIT 9606.ENSP00000437955 9606.ENSP00000288135 0 0 0 0 0.056 0 0 0.568 0.574

HIF1A VCAM1 9606.ENSP00000437955 9606.ENSP00000294728 0 0 0 0 0.063 0 0 0.511 0.522

HIF1A NOS3 9606.ENSP00000437955 9606.ENSP00000297494 0 0 0 0 0 0 0.9 0.701 0.968

HIF1A HPRT1 9606.ENSP00000437955 9606.ENSP00000298556 0 0 0 0 0 0 0 0.401 0.4

HIF1A MMP3 9606.ENSP00000437955 9606.ENSP00000299855 0 0 0 0 0 0 0 0.478 0.478

HIF1A IGF1 9606.ENSP00000437955 9606.ENSP00000302665 0 0 0 0 0 0 0 0.7 0.7

HIF1A IL13 9606.ENSP00000437955 9606.ENSP00000304915 0 0 0 0 0 0 0 0.451 0.451

HIF1A LEP 9606.ENSP00000437955 9606.ENSP00000312652 0 0 0 0 0 0 0 0.526 0.526

HIF1A MMP1 9606.ENSP00000437955 9606.ENSP00000322788 0 0 0 0 0.065 0 0 0.476 0.489

HIF1A HSPA5 9606.ENSP00000437955 9606.ENSP00000324173 0 0 0 0 0.065 0.056 0 0.522 0.541

HIF1A NOS2 9606.ENSP00000437955 9606.ENSP00000327251 0 0 0 0 0 0 0 0.519 0.519

HIF1A P4HB 9606.ENSP00000437955 9606.ENSP00000327801 0 0 0 0 0 0 0.9 0.3 0.927

HIF1A SELE 9606.ENSP00000437955 9606.ENSP00000331736 0 0 0 0 0 0 0 0.543 0.543

HIF1A STAT5A 9606.ENSP00000437955 9606.ENSP00000341208 0 0 0 0 0 0.056 0 0.482 0.49

HIF1A USP7 9606.ENSP00000437955 9606.ENSP00000343535 0 0 0 0 0.062 0.27 0 0.52 0.643

HIF1A IL17A 9606.ENSP00000437955 9606.ENSP00000344192 0 0 0 0 0 0.182 0 0.518 0.588

HIF1A OCLN 9606.ENSP00000437955 9606.ENSP00000347379 0 0 0 0 0 0 0 0.459 0.459

HIF1A RET 9606.ENSP00000437955 9606.ENSP00000347942 0 0 0 0 0 0 0 0.424 0.424

HIF1A SREBF1 9606.ENSP00000437955 9606.ENSP00000348069 0 0 0 0 0.062 0 0 0.588 0.597

HIF1A VCP 9606.ENSP00000437955 9606.ENSP00000351777 0 0 0 0 0 0.27 0 0.44 0.574

HIF1A STAT1 9606.ENSP00000437955 9606.ENSP00000354394 0 0 0 0 0.062 0.056 0 0.517 0.534

HIF1A TOP1 9606.ENSP00000437955 9606.ENSP00000354522 0 0 0 0 0.063 0 0 0.461 0.473

HIF1A MTOR 9606.ENSP00000437955 9606.ENSP00000354558 0 0 0 0 0 0.096 0 0.807 0.819

HIF1A PARP1 9606.ENSP00000437955 9606.ENSP00000355759 0 0 0 0 0 0.27 0 0.733 0.797

HIF1A NGF 9606.ENSP00000437955 9606.ENSP00000358525 0 0 0 0 0 0 0 0.424 0.424

HIF1A JUN 9606.ENSP00000437955 9606.ENSP00000360266 0 0 0 0 0 0.676 0.9 0.901 0.996

HIF1A PTEN 9606.ENSP00000437955 9606.ENSP00000361021 0 0 0 0 0.062 0 0 0.788 0.793

HIF1A MMP9 9606.ENSP00000437955 9606.ENSP00000361405 0 0 0 0 0 0 0 0.718 0.718

HIF1A TLR4 9606.ENSP00000437955 9606.ENSP00000363089 0 0 0 0 0.062 0.056 0 0.616 0.63

HIF1A SPP1 9606.ENSP00000437955 9606.ENSP00000378517 0 0 0 0 0 0 0 0.556 0.556

HIF1A INS 9606.ENSP00000437955 9606.ENSP00000380432 0 0 0 0 0 0 0 0.667 0.667

HIF1A IL6 9606.ENSP00000437955 9606.ENSP00000385675 0 0 0 0 0.065 0 0 0.717 0.724

HIF1A TNF 9606.ENSP00000437955 9606.ENSP00000398698 0 0 0 0 0 0 0 0.707 0.707

HIF1A MYD88 9606.ENSP00000437955 9606.ENSP00000401399 0 0 0 0 0 0 0 0.403 0.403

HIF1A IL10 9606.ENSP00000437955 9606.ENSP00000412237 0 0 0 0 0 0 0 0.604 0.604

HIF1A NOS1 9606.ENSP00000437955 9606.ENSP00000477999 0 0 0 0 0 0 0 0.422 0.422

HIF1A VEGFA 9606.ENSP00000437955 9606.ENSP00000478570 0 0 0 0 0.062 0.874 0.9 0.919 0.998

HMGCR NR1H2 9606.ENSP00000287936 9606.ENSP00000253727 0 0 0 0 0.062 0 0 0.478 0.489

HMGCR PPARG 9606.ENSP00000287936 9606.ENSP00000287820 0 0 0 0 0.062 0 0 0.568 0.577

HMGCR TNF 9606.ENSP00000287936 9606.ENSP00000398698 0 0 0 0 0 0 0 0.415 0.415

HMGCR LEP 9606.ENSP00000287936 9606.ENSP00000312652 0 0 0 0 0 0 0 0.435 0.435

HMGCR IL6 9606.ENSP00000287936 9606.ENSP00000385675 0 0 0 0 0 0 0 0.455 0.455

HMGCR HSPA5 9606.ENSP00000287936 9606.ENSP00000324173 0 0 0 0 0.062 0.059 0 0.443 0.465

HMGCR NOS3 9606.ENSP00000287936 9606.ENSP00000297494 0 0 0 0 0.049 0 0 0.525 0.529

HMGCR MTTP 9606.ENSP00000287936 9606.ENSP00000427679 0 0 0 0 0 0 0 0.565 0.565

HMGCR LPL 9606.ENSP00000287936 9606.ENSP00000309757 0 0 0 0 0 0 0 0.569 0.569

HMGCR INS 9606.ENSP00000287936 9606.ENSP00000380432 0 0 0 0 0 0 0 0.607 0.607

HMGCR VCP 9606.ENSP00000287936 9606.ENSP00000351777 0 0 0 0 0.062 0.519 0 0.522 0.765

HMGCR SREBF1 9606.ENSP00000287936 9606.ENSP00000348069 0 0 0 0 0.062 0 0.9 0.888 0.988

HMOX1 TNFRSF1A 9606.ENSP00000216117 9606.ENSP00000162749 0 0 0 0 0.094 0 0 0.473 0.502

HMOX1 LEP 9606.ENSP00000216117 9606.ENSP00000312652 0 0 0 0 0 0 0 0.403 0.403

HMOX1 THBD 9606.ENSP00000216117 9606.ENSP00000366307 0 0 0 0 0.088 0 0 0.375 0.405

HMOX1 OCLN 9606.ENSP00000216117 9606.ENSP00000347379 0 0 0 0 0 0 0 0.406 0.406

HMOX1 SREBF1 9606.ENSP00000216117 9606.ENSP00000348069 0 0 0 0 0 0 0 0.413 0.412

HMOX1 MMP3 9606.ENSP00000216117 9606.ENSP00000299855 0 0 0 0 0 0 0 0.413 0.413

HMOX1 IL2 9606.ENSP00000216117 9606.ENSP00000226730 0 0 0 0 0 0 0 0.414 0.414

HMOX1 NGF 9606.ENSP00000216117 9606.ENSP00000358525 0 0 0 0 0 0 0 0.423 0.422

HMOX1 PTEN 9606.ENSP00000216117 9606.ENSP00000361021 0 0 0 0 0.069 0 0 0.405 0.423

HMOX1 IL1A 9606.ENSP00000216117 9606.ENSP00000263339 0 0 0 0 0 0 0 0.426 0.426

HMOX1 MMP1 9606.ENSP00000216117 9606.ENSP00000322788 0 0 0 0 0.068 0 0 0.415 0.431

HMOX1 PRKCD 9606.ENSP00000216117 9606.ENSP00000378217 0 0 0 0 0.069 0.058 0 0.405 0.432

HMOX1 IL17A 9606.ENSP00000216117 9606.ENSP00000344192 0 0 0 0 0 0 0 0.451 0.451

HMOX1 TGFB1 9606.ENSP00000216117 9606.ENSP00000221930 0 0 0 0 0.088 0 0 0.426 0.454

HMOX1 SELP 9606.ENSP00000216117 9606.ENSP00000263686 0 0 0 0 0 0 0 0.455 0.455

HMOX1 STAT1 9606.ENSP00000216117 9606.ENSP00000354394 0 0 0 0 0 0 0 0.455 0.455

HMOX1 IGF1 9606.ENSP00000216117 9606.ENSP00000302665 0 0 0 0 0 0 0 0.46 0.459

HMOX1 SNCA 9606.ENSP00000216117 9606.ENSP00000338345 0 0 0 0 0 0 0 0.46 0.459

HMOX1 MTOR 9606.ENSP00000216117 9606.ENSP00000354558 0 0 0 0 0 0 0 0.46 0.459

HMOX1 REN 9606.ENSP00000216117 9606.ENSP00000272190 0 0 0 0 0.064 0 0 0.46 0.472

HMOX1 IFNG 9606.ENSP00000216117 9606.ENSP00000229135 0 0 0 0 0 0 0 0.475 0.475

HMOX1 TH 9606.ENSP00000216117 9606.ENSP00000370571 0 0 0 0 0.081 0 0 0.458 0.48

HMOX1 MMP2 9606.ENSP00000216117 9606.ENSP00000219070 0 0 0 0 0 0 0 0.486 0.486

HMOX1 MYD88 9606.ENSP00000216117 9606.ENSP00000401399 0 0 0 0 0.083 0 0 0.479 0.502

HMOX1 SPP1 9606.ENSP00000216117 9606.ENSP00000378517 0 0 0 0 0.062 0 0 0.494 0.505

HMOX1 SELE 9606.ENSP00000216117 9606.ENSP00000331736 0 0 0 0 0 0 0 0.509 0.509

HMOX1 NCF1 9606.ENSP00000216117 9606.ENSP00000289473 0 0 0 0 0 0 0 0.517 0.517

HMOX1 LCN2 9606.ENSP00000216117 9606.ENSP00000362108 0 0 0 0 0.062 0 0 0.507 0.517

HMOX1 HSPB1 9606.ENSP00000216117 9606.ENSP00000248553 0 0 0 0 0.062 0 0 0.509 0.519

HMOX1 INS 9606.ENSP00000216117 9606.ENSP00000380432 0 0 0 0 0 0 0 0.619 0.619

HMOX1 TP53 9606.ENSP00000216117 9606.ENSP00000269305 0 0 0 0 0.062 0 0 0.611 0.619

HMOX1 VCAM1 9606.ENSP00000216117 9606.ENSP00000294728 0 0 0 0 0.062 0 0 0.616 0.624

HMOX1 HSPA5 9606.ENSP00000216117 9606.ENSP00000324173 0 0 0 0 0 0 0 0.636 0.637

HMOX1 MMP9 9606.ENSP00000216117 9606.ENSP00000361405 0 0 0 0 0.088 0 0 0.619 0.637

HMOX1 MPO 9606.ENSP00000216117 9606.ENSP00000225275 0 0 0 0 0 0 0 0.652 0.652

HMOX1 PPARG 9606.ENSP00000216117 9606.ENSP00000287820 0 0 0 0 0 0 0 0.654 0.654

HMOX1 NFKBIA 9606.ENSP00000216117 9606.ENSP00000216797 0 0 0 0 0 0 0 0.664 0.664

HMOX1 ICAM1 9606.ENSP00000216117 9606.ENSP00000264832 0 0 0 0 0.077 0 0 0.653 0.666

HMOX1 TLR4 9606.ENSP00000216117 9606.ENSP00000363089 0 0 0 0 0.096 0 0 0.654 0.674

HMOX1 VEGFA 9606.ENSP00000216117 9606.ENSP00000478570 0 0 0 0 0 0 0 0.683 0.683

HMOX1 IL10 9606.ENSP00000216117 9606.ENSP00000412237 0 0 0 0 0 0 0 0.695 0.695

HMOX1 SOD1 9606.ENSP00000216117 9606.ENSP00000270142 0 0 0 0 0 0 0 0.708 0.708

HMOX1 NOS2 9606.ENSP00000216117 9606.ENSP00000327251 0 0 0 0 0 0.177 0 0.667 0.714

HMOX1 IL1B 9606.ENSP00000216117 9606.ENSP00000263341 0 0 0 0 0.07 0 0 0.76 0.767

HMOX1 NOS1 9606.ENSP00000216117 9606.ENSP00000477999 0 0 0 0 0 0.324 0 0.672 0.769

HMOX1 TNF 9606.ENSP00000216117 9606.ENSP00000398698 0 0 0 0 0 0 0 0.775 0.775

HMOX1 NOS3 9606.ENSP00000216117 9606.ENSP00000297494 0 0 0 0 0 0.177 0 0.752 0.787

HMOX1 IL6 9606.ENSP00000216117 9606.ENSP00000385675 0 0 0 0 0.073 0 0 0.78 0.787

HMOX1 IL13 9606.ENSP00000216117 9606.ENSP00000304915 0 0 0 0 0 0 0.9 0.423 0.939

HMOX1 IL4 9606.ENSP00000216117 9606.ENSP00000231449 0 0 0 0 0 0 0.9 0.505 0.948

HMOX1 JUN 9606.ENSP00000216117 9606.ENSP00000360266 0 0 0 0 0 0 0.9 0.692 0.967

HNF1B NOTCH2 9606.ENSP00000480291 9606.ENSP00000256646 0 0 0 0 0 0 0 0.468 0.468

HNF1B TP53 9606.ENSP00000480291 9606.ENSP00000269305 0 0 0 0 0 0 0 0.413 0.412

HNF1B REN 9606.ENSP00000480291 9606.ENSP00000272190 0 0 0 0 0.063 0 0 0.443 0.455

HNF1B PPARG 9606.ENSP00000480291 9606.ENSP00000287820 0 0 0 0 0.052 0.057 0 0.484 0.498

HNF1B SPP1 9606.ENSP00000480291 9606.ENSP00000378517 0 0 0 0 0.055 0 0 0.392 0.4

HNF1B INS 9606.ENSP00000480291 9606.ENSP00000380432 0 0 0 0 0 0 0 0.524 0.524

HPRT1 IL2 9606.ENSP00000298556 9606.ENSP00000226730 0 0 0 0 0 0 0 0.468 0.468

HPRT1 IFNG 9606.ENSP00000298556 9606.ENSP00000229135 0 0 0 0 0 0 0 0.489 0.489

HPRT1 IL4 9606.ENSP00000298556 9606.ENSP00000231449 0 0 0 0 0.062 0 0 0.531 0.542

HPRT1 TFPI 9606.ENSP00000298556 9606.ENSP00000233156 0 0 0 0 0.062 0 0 0.444 0.456

HPRT1 IL1B 9606.ENSP00000298556 9606.ENSP00000263341 0 0 0 0 0 0 0 0.61 0.61

HPRT1 TP53 9606.ENSP00000298556 9606.ENSP00000269305 0 0 0 0 0 0 0 0.523 0.523

HPRT1 PPARG 9606.ENSP00000298556 9606.ENSP00000287820 0 0 0 0 0 0 0 0.4 0.4

HPRT1 KIT 9606.ENSP00000298556 9606.ENSP00000288135 0 0 0 0 0 0 0 0.403 0.403

HPRT1 JUN 9606.ENSP00000298556 9606.ENSP00000360266 0 0 0 0 0 0.094 0 0.369 0.403

HPRT1 MMP9 9606.ENSP00000298556 9606.ENSP00000361405 0 0 0 0 0.062 0 0 0.39 0.403

HPRT1 IL13 9606.ENSP00000298556 9606.ENSP00000304915 0 0 0 0 0 0 0 0.43 0.43

HPRT1 TLR4 9606.ENSP00000298556 9606.ENSP00000363089 0 0 0 0 0 0 0 0.46 0.46

HPRT1 IL17A 9606.ENSP00000298556 9606.ENSP00000344192 0 0 0 0 0.054 0 0 0.528 0.534

HPRT1 INS 9606.ENSP00000298556 9606.ENSP00000380432 0 0 0 0 0.062 0 0 0.527 0.537

HPRT1 IL10 9606.ENSP00000298556 9606.ENSP00000412237 0 0 0 0 0.051 0 0 0.572 0.577

HPRT1 IL6 9606.ENSP00000298556 9606.ENSP00000385675 0 0 0 0 0 0 0 0.621 0.621

HPRT1 TNF 9606.ENSP00000298556 9606.ENSP00000398698 0 0 0 0 0 0 0 0.63 0.63

HPRT1 XDH 9606.ENSP00000298556 9606.ENSP00000368727 0 0 0 0 0.062 0 0.9 0.357 0.934

HSD11B1 NR3C1 9606.ENSP00000355995 9606.ENSP00000231509 0 0 0 0 0.062 0.057 0 0.814 0.822

HSD11B1 REN 9606.ENSP00000355995 9606.ENSP00000272190 0 0 0 0 0.063 0.096 0 0.402 0.449

HSD11B1 SRD5A1 9606.ENSP00000355995 9606.ENSP00000274192 0 0 0 0 0.063 0 0 0.64 0.648

HSD11B1 PPARG 9606.ENSP00000355995 9606.ENSP00000287820 0 0 0 0 0.062 0.057 0.9 0.505 0.95

HSD11B1 IGF1 9606.ENSP00000355995 9606.ENSP00000302665 0 0 0 0 0.084 0 0 0.456 0.48

HSD11B1 LEP 9606.ENSP00000355995 9606.ENSP00000312652 0 0 0 0 0 0 0 0.547 0.547

HSD11B1 NR3C2 9606.ENSP00000355995 9606.ENSP00000350815 0 0 0 0 0.085 0.057 0 0.729 0.746

HSD11B1 TNF 9606.ENSP00000355995 9606.ENSP00000398698 0 0 0 0 0 0 0 0.415 0.415

HSD11B1 IL6 9606.ENSP00000355995 9606.ENSP00000385675 0 0 0 0 0.076 0 0 0.402 0.424

HSD11B1 INS 9606.ENSP00000355995 9606.ENSP00000380432 0 0 0 0 0 0 0 0.601 0.601

HSPA5 NFKBIA 9606.ENSP00000324173 9606.ENSP00000216797 0 0 0 0 0 0.3 0 0.398 0.561

HSPA5 HSPB1 9606.ENSP00000324173 9606.ENSP00000248553 0 0 0 0 0.078 0.095 0 0.713 0.74

HSPA5 IL1B 9606.ENSP00000324173 9606.ENSP00000263341 0 0 0 0 0 0 0 0.506 0.506

HSPA5 TP53 9606.ENSP00000324173 9606.ENSP00000269305 0 0 0 0 0 0.428 0 0.822 0.894

HSPA5 SOD1 9606.ENSP00000324173 9606.ENSP00000270142 0 0 0 0 0 0.149 0.8 0.722 0.948

HSPA5 PPARG 9606.ENSP00000324173 9606.ENSP00000287820 0 0 0 0 0 0.066 0 0.393 0.409

HSPA5 NOS3 9606.ENSP00000324173 9606.ENSP00000297494 0 0 0 0 0.053 0.056 0 0.398 0.415

HSPA5 ITPR1 9606.ENSP00000324173 9606.ENSP00000306253 0 0 0 0 0 0.058 0 0.672 0.678

HSPA5 TLR4 9606.ENSP00000324173 9606.ENSP00000363089 0 0 0 0 0.049 0.102 0 0.39 0.433

HSPA5 PARP1 9606.ENSP00000324173 9606.ENSP00000355759 0 0 0 0 0 0 0 0.442 0.442

HSPA5 KCND2 9606.ENSP00000324173 9606.ENSP00000333496 0 0 0 0 0.064 0.056 0 0.425 0.448

HSPA5 VEGFA 9606.ENSP00000324173 9606.ENSP00000478570 0 0 0 0 0 0 0 0.467 0.467

HSPA5 MTOR 9606.ENSP00000324173 9606.ENSP00000354558 0 0 0 0 0 0.113 0 0.478 0.517

HSPA5 IL6 9606.ENSP00000324173 9606.ENSP00000385675 0 0 0 0 0 0 0 0.527 0.527

HSPA5 SREBF1 9606.ENSP00000324173 9606.ENSP00000348069 0 0 0 0 0 0.061 0 0.538 0.547

HSPA5 PTEN 9606.ENSP00000324173 9606.ENSP00000361021 0 0 0 0 0 0.294 0 0.445 0.592

HSPA5 TNF 9606.ENSP00000324173 9606.ENSP00000398698 0 0 0 0 0 0.123 0 0.585 0.621

HSPA5 INS 9606.ENSP00000324173 9606.ENSP00000380432 0 0 0 0 0 0 0 0.625 0.625

HSPA5 ITPR3 9606.ENSP00000324173 9606.ENSP00000363435 0 0 0 0 0 0.058 0 0.653 0.66

HSPA5 JUN 9606.ENSP00000324173 9606.ENSP00000360266 0 0 0 0 0.062 0.111 0 0.633 0.667

HSPA5 VCP 9606.ENSP00000324173 9606.ENSP00000351777 0.046 0 0 0 0.154 0.211 0 0.869 0.906

HSPA5 SNCA 9606.ENSP00000324173 9606.ENSP00000338345 0 0 0 0 0 0.13 0.8 0.792 0.96

HSPA5 P4HB 9606.ENSP00000324173 9606.ENSP00000327801 0 0 0 0 0.743 0.744 0.36 0.903 0.995

HSPB1 NFKBIA 9606.ENSP00000248553 9606.ENSP00000216797 0 0 0 0 0 0.213 0 0.611 0.68

HSPB1 MMP2 9606.ENSP00000248553 9606.ENSP00000219070 0 0 0 0 0.087 0 0 0.537 0.559

HSPB1 TLR4 9606.ENSP00000248553 9606.ENSP00000363089 0 0 0 0 0 0 0 0.402 0.402

HSPB1 PTEN 9606.ENSP00000248553 9606.ENSP00000361021 0 0 0 0 0.066 0 0 0.392 0.408

HSPB1 NOS3 9606.ENSP00000248553 9606.ENSP00000297494 0 0 0 0 0.062 0.08 0 0.37 0.408

HSPB1 P4HB 9606.ENSP00000248553 9606.ENSP00000327801 0 0 0 0 0.088 0 0 0.392 0.421

HSPB1 VCP 9606.ENSP00000248553 9606.ENSP00000351777 0 0 0 0 0 0.147 0 0.374 0.443

HSPB1 IL1B 9606.ENSP00000248553 9606.ENSP00000263341 0 0 0 0 0 0 0 0.469 0.469

HSPB1 IL6 9606.ENSP00000248553 9606.ENSP00000385675 0 0 0 0 0 0 0 0.517 0.517

HSPB1 INS 9606.ENSP00000248553 9606.ENSP00000380432 0 0 0 0 0 0.27 0 0.416 0.556

HSPB1 JUN 9606.ENSP00000248553 9606.ENSP00000360266 0 0 0 0 0.062 0 0 0.601 0.609

HSPB1 SOD1 9606.ENSP00000248553 9606.ENSP00000270142 0 0 0 0 0.076 0.068 0 0.587 0.613

HSPB1 TNF 9606.ENSP00000248553 9606.ENSP00000398698 0 0 0 0 0 0.27 0 0.553 0.66

HSPB1 VEGFA 9606.ENSP00000248553 9606.ENSP00000478570 0 0 0 0 0.049 0.101 0 0.704 0.725

HSPB1 PRKCD 9606.ENSP00000248553 9606.ENSP00000378217 0 0 0 0 0 0.345 0 0.703 0.797

HSPB1 SNCA 9606.ENSP00000248553 9606.ENSP00000338345 0 0 0 0 0.062 0.629 0 0.589 0.844

HSPB1 TP53 9606.ENSP00000248553 9606.ENSP00000269305 0 0 0 0 0 0.848 0 0.76 0.962

HTR2A NR3C1 9606.ENSP00000437737 9606.ENSP00000231509 0 0 0 0 0 0.056 0 0.459 0.467

HTR2A SLC6A4 9606.ENSP00000437737 9606.ENSP00000261707 0 0 0 0 0 0.057 0 0.912 0.914

HTR2A SST 9606.ENSP00000437737 9606.ENSP00000287641 0 0 0 0 0.098 0 0 0.363 0.401

HTR2A TH 9606.ENSP00000437737 9606.ENSP00000370571 0 0 0 0 0 0 0 0.472 0.472

IARS2 OGDH 9606.ENSP00000355889 9606.ENSP00000222673 0 0 0 0 0.277 0.3 0 0 0.472

ICAM1 TNFRSF1A 9606.ENSP00000264832 9606.ENSP00000162749 0 0 0 0 0.088 0 0 0.635 0.653

ICAM1 MIF 9606.ENSP00000264832 9606.ENSP00000215754 0 0 0 0 0 0 0 0.504 0.504

ICAM1 NFKBIA 9606.ENSP00000264832 9606.ENSP00000216797 0 0 0 0 0.127 0 0 0.719 0.744

ICAM1 MMP2 9606.ENSP00000264832 9606.ENSP00000219070 0 0 0 0 0 0 0 0.611 0.611

ICAM1 TGFB1 9606.ENSP00000264832 9606.ENSP00000221930 0 0 0 0 0.097 0 0 0.51 0.538

ICAM1 MPO 9606.ENSP00000264832 9606.ENSP00000225275 0 0 0 0 0 0 0 0.74 0.74

ICAM1 NFKB1 9606.ENSP00000264832 9606.ENSP00000226574 0 0 0 0 0.096 0 0 0.418 0.451

ICAM1 IL2 9606.ENSP00000264832 9606.ENSP00000226730 0 0 0 0 0 0 0 0.733 0.733

ICAM1 KITLG 9606.ENSP00000264832 9606.ENSP00000228280 0 0 0 0 0 0 0 0.459 0.459

ICAM1 IFNG 9606.ENSP00000264832 9606.ENSP00000229135 0 0 0 0 0 0 0 0.738 0.738

ICAM1 IL4 9606.ENSP00000264832 9606.ENSP00000231449 0 0 0 0 0 0 0 0.733 0.733

ICAM1 IRF1 9606.ENSP00000264832 9606.ENSP00000245414 0 0 0 0 0.157 0 0 0.503 0.563

ICAM1 MDM2 9606.ENSP00000264832 9606.ENSP00000258149 0 0 0 0 0 0 0 0.662 0.662

ICAM1 IL1RN 9606.ENSP00000264832 9606.ENSP00000259206 0 0 0 0 0.139 0 0 0.469 0.523

ICAM1 IL1A 9606.ENSP00000264832 9606.ENSP00000263339 0 0 0 0 0.113 0 0 0.701 0.723

ICAM1 IL1B 9606.ENSP00000264832 9606.ENSP00000263341 0 0 0 0 0.154 0 0 0.858 0.874

ICAM1 SELP 9606.ENSP00000264832 9606.ENSP00000263686 0 0 0 0 0.062 0 0 0.943 0.945

ICAM1 IDO1 9606.ENSP00000264832 9606.ENSP00000430950 0 0 0 0 0.069 0 0 0.391 0.408

ICAM1 SOCS1 9606.ENSP00000264832 9606.ENSP00000329418 0 0 0 0 0.076 0 0 0.391 0.413

ICAM1 STAT5A 9606.ENSP00000264832 9606.ENSP00000341208 0 0 0 0 0.128 0 0 0.361 0.418

ICAM1 NOD2 9606.ENSP00000264832 9606.ENSP00000300589 0 0 0 0 0.088 0 0 0.392 0.421

ICAM1 PF4 9606.ENSP00000264832 9606.ENSP00000296029 0 0 0 0 0.062 0 0 0.463 0.475

ICAM1 LCN2 9606.ENSP00000264832 9606.ENSP00000362108 0 0 0 0 0.062 0 0 0.477 0.488

ICAM1 KIT 9606.ENSP00000264832 9606.ENSP00000288135 0 0 0 0 0.062 0 0 0.503 0.513

ICAM1 IGF1 9606.ENSP00000264832 9606.ENSP00000302665 0 0 0 0 0 0 0 0.516 0.516

ICAM1 LTA 9606.ENSP00000264832 9606.ENSP00000403495 0 0 0 0 0.061 0 0 0.523 0.533

ICAM1 TLR3 9606.ENSP00000264832 9606.ENSP00000296795 0 0 0 0 0 0 0 0.533 0.534

ICAM1 NGF 9606.ENSP00000264832 9606.ENSP00000358525 0 0 0 0 0 0 0 0.534 0.534

ICAM1 TNFAIP3 9606.ENSP00000264832 9606.ENSP00000481570 0 0 0 0 0.238 0 0 0.415 0.535

ICAM1 SPP1 9606.ENSP00000264832 9606.ENSP00000378517 0 0 0 0 0 0 0 0.538 0.538

ICAM1 NOS2 9606.ENSP00000264832 9606.ENSP00000327251 0 0 0 0 0 0.062 0 0.533 0.543

ICAM1 NCF1 9606.ENSP00000264832 9606.ENSP00000289473 0 0 0 0 0.069 0 0 0.541 0.555

ICAM1 LEP 9606.ENSP00000264832 9606.ENSP00000312652 0 0 0 0 0 0 0 0.562 0.562

ICAM1 TP53 9606.ENSP00000264832 9606.ENSP00000269305 0 0 0 0 0.062 0 0 0.559 0.568

ICAM1 REN 9606.ENSP00000264832 9606.ENSP00000272190 0 0 0 0 0 0 0 0.578 0.578

ICAM1 MMP1 9606.ENSP00000264832 9606.ENSP00000322788 0 0 0 0 0.088 0 0 0.568 0.589

ICAM1 MYD88 9606.ENSP00000264832 9606.ENSP00000401399 0 0 0 0 0.09 0 0 0.573 0.595

ICAM1 JUN 9606.ENSP00000264832 9606.ENSP00000360266 0 0 0 0 0 0 0 0.607 0.607

ICAM1 STAT1 9606.ENSP00000264832 9606.ENSP00000354394 0 0 0 0 0.061 0 0 0.6 0.609

ICAM1 PPARG 9606.ENSP00000264832 9606.ENSP00000287820 0 0 0 0 0.062 0 0 0.601 0.609

ICAM1 MMP3 9606.ENSP00000264832 9606.ENSP00000299855 0 0 0 0 0.063 0 0 0.603 0.612

ICAM1 IL13 9606.ENSP00000264832 9606.ENSP00000304915 0 0 0 0 0 0 0 0.637 0.637

ICAM1 OCLN 9606.ENSP00000264832 9606.ENSP00000347379 0 0 0 0 0 0 0 0.666 0.667

ICAM1 IL17A 9606.ENSP00000264832 9606.ENSP00000344192 0 0 0 0 0 0 0 0.686 0.686

ICAM1 THBD 9606.ENSP00000264832 9606.ENSP00000366307 0 0 0 0 0.106 0 0 0.712 0.731

ICAM1 NOS3 9606.ENSP00000264832 9606.ENSP00000297494 0 0 0 0 0 0 0 0.731 0.731

ICAM1 VEGFA 9606.ENSP00000264832 9606.ENSP00000478570 0 0 0 0 0 0 0 0.763 0.763

ICAM1 INS 9606.ENSP00000264832 9606.ENSP00000380432 0 0 0 0 0 0 0 0.766 0.766

ICAM1 MMP9 9606.ENSP00000264832 9606.ENSP00000361405 0 0 0 0 0.093 0 0 0.768 0.781

ICAM1 TLR4 9606.ENSP00000264832 9606.ENSP00000363089 0 0 0 0 0.083 0 0 0.786 0.796

ICAM1 TNF 9606.ENSP00000264832 9606.ENSP00000398698 0 0 0 0 0.119 0 0 0.878 0.888

ICAM1 IL6 9606.ENSP00000264832 9606.ENSP00000385675 0 0 0 0 0.152 0 0 0.876 0.89

ICAM1 SELE 9606.ENSP00000264832 9606.ENSP00000331736 0 0 0 0 0.061 0 0 0.915 0.916

ICAM1 VCAM1 9606.ENSP00000264832 9606.ENSP00000294728 0 0 0 0 0.069 0 0 0.928 0.93

ICAM1 TNFRSF1B 9606.ENSP00000264832 9606.ENSP00000365435 0 0 0 0 0.117 0 0.9 0.564 0.958

ICAM1 IL10 9606.ENSP00000264832 9606.ENSP00000412237 0 0 0 0 0 0 0.9 0.775 0.976

ICAM1 ITGAL 9606.ENSP00000264832 9606.ENSP00000349252 0 0 0 0 0.091 0.972 0.9 0.989 0.999

ICAM1 ITGB2 9606.ENSP00000264832 9606.ENSP00000380948 0 0 0 0 0.113 0.835 0.9 0.989 0.999

IDO1 TGFB1 9606.ENSP00000430950 9606.ENSP00000221930 0 0 0 0 0 0 0 0.4 0.4

IDO1 IL2 9606.ENSP00000430950 9606.ENSP00000226730 0 0 0 0 0 0 0 0.601 0.601

IDO1 IFNG 9606.ENSP00000430950 9606.ENSP00000229135 0 0 0 0 0.089 0 0 0.673 0.69

IDO1 IL4 9606.ENSP00000430950 9606.ENSP00000231449 0 0 0 0 0 0 0 0.569 0.569

IDO1 IRF1 9606.ENSP00000430950 9606.ENSP00000245414 0 0 0 0 0.148 0 0 0.479 0.537

IDO1 IL1B 9606.ENSP00000430950 9606.ENSP00000263341 0 0 0 0 0.09 0 0 0.603 0.623

IDO1 TLR3 9606.ENSP00000430950 9606.ENSP00000296795 0 0 0 0 0 0 0 0.46 0.459

IDO1 IL13 9606.ENSP00000430950 9606.ENSP00000304915 0 0 0 0 0 0 0 0.454 0.454

IDO1 NOS2 9606.ENSP00000430950 9606.ENSP00000327251 0 0 0 0 0 0 0 0.428 0.428

IDO1 SOCS1 9606.ENSP00000430950 9606.ENSP00000329418 0 0 0 0 0.099 0.109 0 0.325 0.411

IDO1 IL17A 9606.ENSP00000430950 9606.ENSP00000344192 0 0 0 0 0 0 0 0.564 0.564

IDO1 STAT1 9606.ENSP00000430950 9606.ENSP00000354394 0 0 0 0 0.158 0 0 0.571 0.623

IDO1 TLR4 9606.ENSP00000430950 9606.ENSP00000363089 0 0 0 0 0 0 0 0.503 0.503

IDO1 IL6 9606.ENSP00000430950 9606.ENSP00000385675 0 0 0 0 0.076 0 0 0.672 0.684

IDO1 TNF 9606.ENSP00000430950 9606.ENSP00000398698 0 0 0 0 0.072 0 0 0.657 0.669

IDO1 IL10 9606.ENSP00000430950 9606.ENSP00000412237 0 0 0 0 0 0 0 0.728 0.728

IDO1 VEGFA 9606.ENSP00000430950 9606.ENSP00000478570 0 0 0 0 0 0 0 0.509 0.509

IFNG TNFRSF1A 9606.ENSP00000229135 9606.ENSP00000162749 0 0 0 0 0 0 0 0.592 0.592

IFNG MIF 9606.ENSP00000229135 9606.ENSP00000215754 0 0 0 0 0 0 0 0.539 0.539

IFNG NFKBIA 9606.ENSP00000229135 9606.ENSP00000216797 0 0 0 0 0 0 0 0.585 0.585

IFNG MMP2 9606.ENSP00000229135 9606.ENSP00000219070 0 0 0 0 0 0 0 0.457 0.457

IFNG TGFB1 9606.ENSP00000229135 9606.ENSP00000221930 0 0 0 0 0 0 0 0.78 0.78

IFNG MPO 9606.ENSP00000229135 9606.ENSP00000225275 0 0 0 0 0 0 0 0.593 0.593

IFNG NFKB1 9606.ENSP00000229135 9606.ENSP00000226574 0 0 0 0 0 0 0 0.457 0.457

IFNG IL2 9606.ENSP00000229135 9606.ENSP00000226730 0 0 0 0 0.087 0 0 0.902 0.907

IFNG KITLG 9606.ENSP00000229135 9606.ENSP00000228280 0 0 0 0 0 0 0 0.499 0.499

IFNG NR3C1 9606.ENSP00000229135 9606.ENSP00000231509 0 0 0 0 0 0 0 0.401 0.401

IFNG PTEN 9606.ENSP00000229135 9606.ENSP00000361021 0 0 0 0 0 0 0 0.405 0.405

IFNG MMP1 9606.ENSP00000229135 9606.ENSP00000322788 0 0 0 0 0.053 0 0 0.425 0.432

IFNG LCN2 9606.ENSP00000229135 9606.ENSP00000362108 0 0 0 0 0 0 0 0.441 0.441

IFNG MMP3 9606.ENSP00000229135 9606.ENSP00000299855 0 0 0 0 0.053 0 0 0.453 0.46

IFNG MTOR 9606.ENSP00000229135 9606.ENSP00000354558 0 0 0 0 0 0 0 0.47 0.47

IFNG KIT 9606.ENSP00000229135 9606.ENSP00000288135 0 0 0 0 0 0 0 0.474 0.474

IFNG VDR 9606.ENSP00000229135 9606.ENSP00000447173 0 0 0 0 0 0 0 0.478 0.478

IFNG SPP1 9606.ENSP00000229135 9606.ENSP00000378517 0 0 0 0 0 0 0 0.478 0.478

IFNG IL17RA 9606.ENSP00000229135 9606.ENSP00000320936 0 0 0 0 0 0 0 0.498 0.498

IFNG NOS3 9606.ENSP00000229135 9606.ENSP00000297494 0 0 0 0 0 0 0 0.502 0.502

IFNG SELP 9606.ENSP00000229135 9606.ENSP00000263686 0 0 0 0 0.065 0 0 0.493 0.505

IFNG OCLN 9606.ENSP00000229135 9606.ENSP00000347379 0 0 0 0 0 0 0 0.505 0.505

IFNG TNFRSF1B 9606.ENSP00000229135 9606.ENSP00000365435 0 0 0 0 0.088 0 0 0.484 0.509

IFNG LEP 9606.ENSP00000229135 9606.ENSP00000312652 0 0 0 0 0 0 0 0.57 0.57

IFNG ITGAL 9606.ENSP00000229135 9606.ENSP00000349252 0 0 0 0 0.096 0 0 0.552 0.578

IFNG PPARG 9606.ENSP00000229135 9606.ENSP00000287820 0 0 0 0 0 0 0 0.579 0.579

IFNG SELE 9606.ENSP00000229135 9606.ENSP00000331736 0 0 0 0 0 0 0 0.588 0.588

IFNG NGF 9606.ENSP00000229135 9606.ENSP00000358525 0 0 0 0 0 0 0 0.591 0.591

IFNG NOD2 9606.ENSP00000229135 9606.ENSP00000300589 0 0 0 0 0.079 0 0 0.579 0.596

IFNG INS 9606.ENSP00000229135 9606.ENSP00000380432 0 0 0 0 0.062 0 0 0.613 0.622

IFNG IGF1 9606.ENSP00000229135 9606.ENSP00000302665 0 0 0 0 0.062 0 0 0.619 0.628

IFNG IL1RN 9606.ENSP00000229135 9606.ENSP00000259206 0 0 0 0 0.076 0 0 0.628 0.642

IFNG VCAM1 9606.ENSP00000229135 9606.ENSP00000294728 0 0 0 0 0 0 0 0.649 0.649

IFNG TP53 9606.ENSP00000229135 9606.ENSP00000269305 0 0 0 0 0 0 0 0.657 0.657

IFNG MMP9 9606.ENSP00000229135 9606.ENSP00000361405 0 0 0 0 0 0 0 0.66 0.661

IFNG VEGFA 9606.ENSP00000229135 9606.ENSP00000478570 0 0 0 0 0 0 0 0.689 0.689

IFNG MYD88 9606.ENSP00000229135 9606.ENSP00000401399 0 0 0 0 0 0 0 0.736 0.736

IFNG NOS2 9606.ENSP00000229135 9606.ENSP00000327251 0 0 0 0 0 0 0 0.764 0.764

IFNG IL1A 9606.ENSP00000229135 9606.ENSP00000263339 0 0 0 0 0 0 0 0.768 0.768

IFNG LTA 9606.ENSP00000229135 9606.ENSP00000403495 0 0 0 0 0.152 0 0 0.75 0.779

IFNG TLR4 9606.ENSP00000229135 9606.ENSP00000363089 0 0 0 0 0 0 0 0.798 0.798

IFNG TLR3 9606.ENSP00000229135 9606.ENSP00000296795 0 0 0 0 0.062 0 0 0.828 0.832

IFNG IL13 9606.ENSP00000229135 9606.ENSP00000304915 0 0 0 0 0.06 0 0 0.883 0.885

IFNG IL17A 9606.ENSP00000229135 9606.ENSP00000344192 0 0 0 0 0.099 0 0 0.895 0.902

IFNG IL1B 9606.ENSP00000229135 9606.ENSP00000263341 0 0 0 0 0.08 0 0 0.903 0.907

IFNG IL4 9606.ENSP00000229135 9606.ENSP00000231449 0 0 0 0 0 0 0 0.91 0.91

IFNG PRKCD 9606.ENSP00000229135 9606.ENSP00000378217 0 0 0 0 0 0 0.9 0.246 0.921

IFNG TNF 9606.ENSP00000229135 9606.ENSP00000398698 0 0 0 0 0.152 0.213 0 0.915 0.938

IFNG IL6 9606.ENSP00000229135 9606.ENSP00000385675 0 0 0 0 0.066 0 0 0.942 0.943

IFNG IL10 9606.ENSP00000229135 9606.ENSP00000412237 0 0 0 0 0.066 0 0 0.95 0.951

IFNG JUN 9606.ENSP00000229135 9606.ENSP00000360266 0 0 0 0 0 0 0.9 0.601 0.958

IFNG SOCS1 9606.ENSP00000229135 9606.ENSP00000329418 0 0 0 0 0.085 0 0.9 0.592 0.959

IFNG STAT5A 9606.ENSP00000229135 9606.ENSP00000341208 0 0 0 0 0.062 0 0.9 0.627 0.962

IFNG IRF1 9606.ENSP00000229135 9606.ENSP00000245414 0 0 0 0 0.088 0 0.9 0.695 0.969

IFNG STAT1 9606.ENSP00000229135 9606.ENSP00000354394 0 0 0 0 0.064 0 0.9 0.832 0.983

IGF1 TNFRSF1A 9606.ENSP00000302665 9606.ENSP00000162749 0 0 0 0 0.062 0 0 0.407 0.419

IGF1 MMP2 9606.ENSP00000302665 9606.ENSP00000219070 0 0 0 0 0.062 0 0.9 0.601 0.959

IGF1 TGFB1 9606.ENSP00000302665 9606.ENSP00000221930 0 0 0 0 0 0 0 0.88 0.88

IGF1 IL2 9606.ENSP00000302665 9606.ENSP00000226730 0 0 0 0 0 0 0 0.504 0.504

IGF1 KITLG 9606.ENSP00000302665 9606.ENSP00000228280 0 0 0 0 0 0 0 0.655 0.655

IGF1 IL4 9606.ENSP00000302665 9606.ENSP00000231449 0 0 0 0 0 0 0 0.575 0.575

IGF1 NR3C1 9606.ENSP00000302665 9606.ENSP00000231509 0 0 0 0 0 0 0 0.521 0.521

IGF1 MDM2 9606.ENSP00000302665 9606.ENSP00000258149 0 0 0 0 0 0 0 0.511 0.511

IGF1 IL1RN 9606.ENSP00000302665 9606.ENSP00000259206 0 0 0 0 0.062 0 0 0.391 0.404

IGF1 IL1A 9606.ENSP00000302665 9606.ENSP00000263339 0 0 0 0 0.062 0 0 0.557 0.566

IGF1 IL1B 9606.ENSP00000302665 9606.ENSP00000263341 0 0 0 0 0.062 0 0 0.761 0.767

IGF1 TP53 9606.ENSP00000302665 9606.ENSP00000269305 0 0 0 0 0 0 0 0.706 0.706

IGF1 SOD1 9606.ENSP00000302665 9606.ENSP00000270142 0 0 0 0 0 0 0 0.504 0.504

IGF1 REN 9606.ENSP00000302665 9606.ENSP00000272190 0 0 0 0 0.062 0 0 0.788 0.792

IGF1 SST 9606.ENSP00000302665 9606.ENSP00000287641 0 0 0 0 0 0 0 0.806 0.806

IGF1 PPARG 9606.ENSP00000302665 9606.ENSP00000287820 0 0 0 0 0.062 0 0 0.768 0.774

IGF1 KIT 9606.ENSP00000302665 9606.ENSP00000288135 0 0 0 0 0 0 0.6 0.611 0.837

IGF1 VCAM1 9606.ENSP00000302665 9606.ENSP00000294728 0 0 0 0 0.062 0 0 0.528 0.538

IGF1 NOS3 9606.ENSP00000302665 9606.ENSP00000297494 0 0 0 0 0 0 0 0.6 0.6

IGF1 MMP3 9606.ENSP00000302665 9606.ENSP00000299855 0 0 0 0 0 0 0 0.556 0.556

IGF1 TH 9606.ENSP00000302665 9606.ENSP00000370571 0 0 0 0 0.073 0 0 0.4 0.42

IGF1 IL17A 9606.ENSP00000302665 9606.ENSP00000344192 0 0 0 0 0 0 0 0.457 0.457

IGF1 LPL 9606.ENSP00000302665 9606.ENSP00000309757 0 0 0 0 0.062 0 0 0.459 0.47

IGF1 SOCS1 9606.ENSP00000302665 9606.ENSP00000329418 0 0 0 0 0 0.162 0 0.395 0.471

IGF1 IL13 9606.ENSP00000302665 9606.ENSP00000304915 0 0 0 0 0 0 0 0.478 0.478

IGF1 STAT1 9606.ENSP00000302665 9606.ENSP00000354394 0 0 0 0 0 0 0 0.48 0.48

IGF1 TLR4 9606.ENSP00000302665 9606.ENSP00000363089 0 0 0 0 0.062 0 0 0.47 0.482

IGF1 SREBF1 9606.ENSP00000302665 9606.ENSP00000348069 0 0 0 0 0 0 0 0.501 0.501

IGF1 VDR 9606.ENSP00000302665 9606.ENSP00000447173 0 0 0 0 0 0 0 0.517 0.517

IGF1 STAT5A 9606.ENSP00000302665 9606.ENSP00000341208 0 0 0 0 0 0 0 0.608 0.609

IGF1 LTA 9606.ENSP00000302665 9606.ENSP00000403495 0 0 0 0 0 0 0 0.611 0.611

IGF1 JUN 9606.ENSP00000302665 9606.ENSP00000360266 0 0 0 0 0 0 0 0.647 0.647

IGF1 MMP9 9606.ENSP00000302665 9606.ENSP00000361405 0 0 0 0 0.062 0 0 0.66 0.668

IGF1 IL10 9606.ENSP00000302665 9606.ENSP00000412237 0 0 0 0 0.062 0 0 0.673 0.68

IGF1 SPP1 9606.ENSP00000302665 9606.ENSP00000378517 0 0 0 0 0 0 0 0.714 0.714

IGF1 PTEN 9606.ENSP00000302665 9606.ENSP00000361021 0 0 0 0 0 0 0 0.726 0.726

IGF1 NGF 9606.ENSP00000302665 9606.ENSP00000358525 0 0 0 0 0 0 0 0.747 0.747

IGF1 MTOR 9606.ENSP00000302665 9606.ENSP00000354558 0 0 0 0 0 0 0 0.782 0.782

IGF1 TNF 9606.ENSP00000302665 9606.ENSP00000398698 0 0 0 0 0 0 0 0.784 0.784

IGF1 SHBG 9606.ENSP00000302665 9606.ENSP00000369816 0 0 0 0 0 0 0 0.803 0.803

IGF1 IL6 9606.ENSP00000302665 9606.ENSP00000385675 0 0 0 0 0 0 0 0.814 0.814

IGF1 LEP 9606.ENSP00000302665 9606.ENSP00000312652 0 0 0 0 0 0 0 0.859 0.859

IGF1 VEGFA 9606.ENSP00000302665 9606.ENSP00000478570 0 0 0 0 0 0 0 0.866 0.866

IGF1 MMP1 9606.ENSP00000302665 9606.ENSP00000322788 0 0 0 0 0 0 0.9 0.483 0.946

IGF1 PRKCD 9606.ENSP00000302665 9606.ENSP00000378217 0 0 0 0 0.049 0 0.9 0.622 0.96

IGF1 INS 9606.ENSP00000302665 9606.ENSP00000380432 0 0 0 0 0 0 0.8 0.991 0.998

IGF1 INSR 9606.ENSP00000302665 9606.ENSP00000303830 0 0 0 0 0 0.945 0.8 0.978 0.999

IL10 TNFRSF1A 9606.ENSP00000412237 9606.ENSP00000162749 0 0 0 0 0 0 0.9 0.671 0.965

IL10 MIF 9606.ENSP00000412237 9606.ENSP00000215754 0 0 0 0 0 0 0 0.624 0.624

IL10 NFKBIA 9606.ENSP00000412237 9606.ENSP00000216797 0 0 0 0 0.062 0 0 0.7 0.707

IL10 MMP2 9606.ENSP00000412237 9606.ENSP00000219070 0 0 0 0 0 0 0.9 0.601 0.958

IL10 TGFB1 9606.ENSP00000412237 9606.ENSP00000221930 0 0 0 0 0 0 0.9 0.788 0.977

IL10 MPO 9606.ENSP00000412237 9606.ENSP00000225275 0 0 0 0 0 0 0 0.76 0.76

IL10 NFKB1 9606.ENSP00000412237 9606.ENSP00000226574 0 0 0 0 0 0 0 0.506 0.506

IL10 IL2 9606.ENSP00000412237 9606.ENSP00000226730 0 0 0 0 0 0 0 0.918 0.918

IL10 KITLG 9606.ENSP00000412237 9606.ENSP00000228280 0 0 0 0 0 0 0 0.556 0.556

IL10 IL4 9606.ENSP00000412237 9606.ENSP00000231449 0 0 0 0 0 0 0 0.937 0.937

IL10 NR3C1 9606.ENSP00000412237 9606.ENSP00000231509 0 0 0 0 0 0.27 0 0.469 0.595

IL10 IRF1 9606.ENSP00000412237 9606.ENSP00000245414 0 0 0 0 0.07 0 0 0.635 0.646

IL10 IL1RN 9606.ENSP00000412237 9606.ENSP00000259206 0 0 0 0 0.08 0 0.9 0.77 0.977

IL10 SMAD7 9606.ENSP00000412237 9606.ENSP00000262158 0 0 0 0 0 0 0 0.401 0.4

IL10 IL1A 9606.ENSP00000412237 9606.ENSP00000263339 0 0 0 0 0.064 0 0.9 0.877 0.987

IL10 IL1B 9606.ENSP00000412237 9606.ENSP00000263341 0 0 0 0 0.086 0 0.9 0.959 0.995

IL10 SELP 9606.ENSP00000412237 9606.ENSP00000263686 0 0 0 0 0.062 0 0 0.606 0.615

IL10 TP53 9606.ENSP00000412237 9606.ENSP00000269305 0 0 0 0 0 0 0 0.58 0.58

IL10 SOD1 9606.ENSP00000412237 9606.ENSP00000270142 0 0 0 0 0 0 0 0.445 0.445

IL10 REN 9606.ENSP00000412237 9606.ENSP00000272190 0 0 0 0 0.062 0 0 0.492 0.503

IL10 PPARG 9606.ENSP00000412237 9606.ENSP00000287820 0 0 0 0 0 0 0 0.688 0.688

IL10 KIT 9606.ENSP00000412237 9606.ENSP00000288135 0 0 0 0 0 0 0 0.533 0.533

IL10 NCF1 9606.ENSP00000412237 9606.ENSP00000289473 0 0 0 0 0.096 0 0 0.393 0.428

IL10 VCAM1 9606.ENSP00000412237 9606.ENSP00000294728 0 0 0 0 0.053 0 0 0.708 0.712

IL10 PF4 9606.ENSP00000412237 9606.ENSP00000296029 0 0 0 0 0.062 0 0 0.496 0.507

IL10 TLR3 9606.ENSP00000412237 9606.ENSP00000296795 0 0 0 0 0 0.064 0 0.784 0.789

IL10 NOS3 9606.ENSP00000412237 9606.ENSP00000297494 0 0 0 0 0 0 0 0.642 0.643

IL10 MMP3 9606.ENSP00000412237 9606.ENSP00000299855 0 0 0 0 0.052 0 0.9 0.611 0.959

IL10 NOD2 9606.ENSP00000412237 9606.ENSP00000300589 0 0 0 0 0.062 0 0 0.72 0.727

IL10 IL13 9606.ENSP00000412237 9606.ENSP00000304915 0 0 0 0 0.062 0 0 0.916 0.917

IL10 LEP 9606.ENSP00000412237 9606.ENSP00000312652 0 0 0 0 0 0 0 0.694 0.694

IL10 IL17RA 9606.ENSP00000412237 9606.ENSP00000320936 0 0 0 0 0 0 0 0.549 0.549

IL10 MMP1 9606.ENSP00000412237 9606.ENSP00000322788 0 0 0 0 0.052 0 0.9 0.565 0.955

IL10 NOS2 9606.ENSP00000412237 9606.ENSP00000327251 0 0 0 0 0 0 0 0.741 0.741

IL10 SOCS1 9606.ENSP00000412237 9606.ENSP00000329418 0 0 0 0 0 0 0 0.67 0.67

IL10 SELE 9606.ENSP00000412237 9606.ENSP00000331736 0 0 0 0 0.049 0 0 0.659 0.662

IL10 STAT5A 9606.ENSP00000412237 9606.ENSP00000341208 0 0 0 0 0 0.068 0 0.671 0.68

IL10 IL17A 9606.ENSP00000412237 9606.ENSP00000344192 0 0 0 0 0.065 0 0.9 0.949 0.994

IL10 OCLN 9606.ENSP00000412237 9606.ENSP00000347379 0 0 0 0 0 0 0 0.583 0.583

IL10 ITGAL 9606.ENSP00000412237 9606.ENSP00000349252 0 0 0 0 0.074 0 0 0.519 0.535

IL10 STAT1 9606.ENSP00000412237 9606.ENSP00000354394 0 0 0 0 0 0.068 0 0.794 0.8

IL10 MTOR 9606.ENSP00000412237 9606.ENSP00000354558 0 0 0 0 0 0 0 0.55 0.55

IL10 PARP1 9606.ENSP00000412237 9606.ENSP00000355759 0 0 0 0 0 0.27 0 0.284 0.455

IL10 S100A8 9606.ENSP00000412237 9606.ENSP00000357722 0 0 0 0 0.062 0 0 0.468 0.479

IL10 S100A9 9606.ENSP00000412237 9606.ENSP00000357727 0 0 0 0 0.062 0 0 0.395 0.408

IL10 NGF 9606.ENSP00000412237 9606.ENSP00000358525 0 0 0 0 0 0 0 0.631 0.631

IL10 JUN 9606.ENSP00000412237 9606.ENSP00000360266 0 0 0 0 0 0 0 0.67 0.67

IL10 PTEN 9606.ENSP00000412237 9606.ENSP00000361021 0 0 0 0 0 0 0 0.481 0.481

IL10 MMP9 9606.ENSP00000412237 9606.ENSP00000361405 0 0 0 0 0 0 0.9 0.758 0.974

IL10 LCN2 9606.ENSP00000412237 9606.ENSP00000362108 0 0 0 0 0 0 0.9 0.561 0.954

IL10 TLR4 9606.ENSP00000412237 9606.ENSP00000363089 0 0 0 0 0 0.121 0 0.889 0.898

IL10 TNFRSF1B 9606.ENSP00000412237 9606.ENSP00000365435 0 0 0 0 0.076 0 0.9 0.603 0.96

IL10 THBD 9606.ENSP00000412237 9606.ENSP00000366307 0 0 0 0 0 0 0 0.519 0.519

IL10 SPP1 9606.ENSP00000412237 9606.ENSP00000378517 0 0 0 0 0 0 0 0.562 0.562

IL10 INS 9606.ENSP00000412237 9606.ENSP00000380432 0 0 0 0 0.062 0 0 0.725 0.731

IL10 ITGB2 9606.ENSP00000412237 9606.ENSP00000380948 0 0 0 0 0.053 0 0 0.426 0.433

IL10 IL6 9606.ENSP00000412237 9606.ENSP00000385675 0 0 0 0 0.073 0 0.3 0.96 0.972

IL10 TNF 9606.ENSP00000412237 9606.ENSP00000398698 0 0 0 0 0.097 0 0.9 0.959 0.996

IL10 MYD88 9606.ENSP00000412237 9606.ENSP00000401399 0 0 0 0 0 0 0 0.849 0.849

IL10 LTA 9606.ENSP00000412237 9606.ENSP00000403495 0 0 0 0 0.081 0 0 0.909 0.913

IL10 TNFAIP3 9606.ENSP00000412237 9606.ENSP00000481570 0 0 0 0 0.062 0 0 0.46 0.471

IL10 VDR 9606.ENSP00000412237 9606.ENSP00000447173 0 0 0 0 0 0 0 0.561 0.561

IL10 VEGFA 9606.ENSP00000412237 9606.ENSP00000478570 0 0 0 0 0 0 0.9 0.845 0.983

IL13 TNFRSF1A 9606.ENSP00000304915 9606.ENSP00000162749 0 0 0 0 0 0 0 0.502 0.502

IL13 MIF 9606.ENSP00000304915 9606.ENSP00000215754 0 0 0 0 0 0 0 0.455 0.455

IL13 NFKBIA 9606.ENSP00000304915 9606.ENSP00000216797 0 0 0 0 0 0 0 0.466 0.466

IL13 MMP2 9606.ENSP00000304915 9606.ENSP00000219070 0 0 0 0 0 0 0 0.415 0.414

IL13 TGFB1 9606.ENSP00000304915 9606.ENSP00000221930 0 0 0 0 0 0 0 0.606 0.606

IL13 MPO 9606.ENSP00000304915 9606.ENSP00000225275 0 0 0 0 0 0 0 0.508 0.508

IL13 IL2 9606.ENSP00000304915 9606.ENSP00000226730 0 0 0 0 0.064 0 0 0.878 0.881

IL13 KITLG 9606.ENSP00000304915 9606.ENSP00000228280 0 0 0 0 0 0 0 0.518 0.518

IL13 IL4 9606.ENSP00000304915 9606.ENSP00000231449 0 0 0 0 0.07 0 0 0.928 0.93

IL13 IRF1 9606.ENSP00000304915 9606.ENSP00000245414 0 0 0 0 0 0 0 0.421 0.42

IL13 IL1RN 9606.ENSP00000304915 9606.ENSP00000259206 0 0 0 0 0 0 0 0.52 0.52

IL13 IL1A 9606.ENSP00000304915 9606.ENSP00000263339 0 0 0 0 0 0 0.9 0.803 0.979

IL13 IL1B 9606.ENSP00000304915 9606.ENSP00000263341 0 0 0 0 0 0 0.9 0.872 0.986

IL13 SELP 9606.ENSP00000304915 9606.ENSP00000263686 0 0 0 0 0 0 0 0.423 0.422

IL13 PPARG 9606.ENSP00000304915 9606.ENSP00000287820 0 0 0 0 0 0 0 0.564 0.564

IL13 KIT 9606.ENSP00000304915 9606.ENSP00000288135 0 0 0 0 0 0 0 0.538 0.538

IL13 VCAM1 9606.ENSP00000304915 9606.ENSP00000294728 0 0 0 0 0 0.213 0.9 0.601 0.965

IL13 TLR3 9606.ENSP00000304915 9606.ENSP00000296795 0 0 0 0 0 0 0 0.601 0.601

IL13 MMP3 9606.ENSP00000304915 9606.ENSP00000299855 0 0 0 0 0 0 0 0.472 0.472

IL13 NOD2 9606.ENSP00000304915 9606.ENSP00000300589 0 0 0 0 0 0 0 0.468 0.468

IL13 MMP1 9606.ENSP00000304915 9606.ENSP00000322788 0 0 0 0 0 0 0 0.404 0.404

IL13 OCLN 9606.ENSP00000304915 9606.ENSP00000347379 0 0 0 0 0 0 0 0.41 0.41

IL13 SPP1 9606.ENSP00000304915 9606.ENSP00000378517 0 0 0 0 0 0 0 0.455 0.455

IL13 SELE 9606.ENSP00000304915 9606.ENSP00000331736 0 0 0 0 0 0 0 0.464 0.464

IL13 NGF 9606.ENSP00000304915 9606.ENSP00000358525 0 0 0 0 0 0 0 0.504 0.504

IL13 SOCS1 9606.ENSP00000304915 9606.ENSP00000329418 0 0 0 0 0 0 0 0.505 0.505

IL13 JUN 9606.ENSP00000304915 9606.ENSP00000360266 0 0 0 0 0 0 0 0.51 0.51

IL13 INS 9606.ENSP00000304915 9606.ENSP00000380432 0 0 0 0 0 0 0 0.517 0.517

IL13 LEP 9606.ENSP00000304915 9606.ENSP00000312652 0 0 0 0 0 0 0 0.532 0.532

IL13 IL17RA 9606.ENSP00000304915 9606.ENSP00000320936 0 0 0 0 0 0 0 0.561 0.561

IL13 STAT5A 9606.ENSP00000304915 9606.ENSP00000341208 0 0 0 0 0 0 0 0.569 0.569

IL13 NOS2 9606.ENSP00000304915 9606.ENSP00000327251 0 0 0 0 0 0 0 0.595 0.595

IL13 MMP9 9606.ENSP00000304915 9606.ENSP00000361405 0 0 0 0 0 0 0 0.61 0.61

IL13 MYD88 9606.ENSP00000304915 9606.ENSP00000401399 0 0 0 0 0 0 0 0.618 0.618

IL13 TLR4 9606.ENSP00000304915 9606.ENSP00000363089 0 0 0 0 0 0 0 0.709 0.709

IL13 VEGFA 9606.ENSP00000304915 9606.ENSP00000478570 0 0 0 0 0 0 0 0.736 0.736

IL13 LTA 9606.ENSP00000304915 9606.ENSP00000403495 0 0 0 0 0.076 0 0 0.763 0.771

IL13 STAT1 9606.ENSP00000304915 9606.ENSP00000354394 0 0 0 0 0 0 0.6 0.658 0.857

IL13 IL17A 9606.ENSP00000304915 9606.ENSP00000344192 0 0 0 0 0.062 0 0 0.902 0.904

IL13 ITGB2 9606.ENSP00000304915 9606.ENSP00000380948 0 0 0 0 0 0 0.9 0.248 0.921

IL13 IL6 9606.ENSP00000304915 9606.ENSP00000385675 0 0 0 0 0 0 0.9 0.881 0.987

IL13 TNF 9606.ENSP00000304915 9606.ENSP00000398698 0 0 0 0 0.061 0 0.9 0.894 0.989

IL17A TNFRSF1A 9606.ENSP00000344192 9606.ENSP00000162749 0 0 0 0 0 0 0 0.588 0.588

IL17A MIF 9606.ENSP00000344192 9606.ENSP00000215754 0 0 0 0 0.062 0 0 0.502 0.512

IL17A NFKBIA 9606.ENSP00000344192 9606.ENSP00000216797 0 0 0 0 0 0 0 0.56 0.56

IL17A MMP2 9606.ENSP00000344192 9606.ENSP00000219070 0 0 0 0 0 0 0.9 0.46 0.943

IL17A TGFB1 9606.ENSP00000344192 9606.ENSP00000221930 0 0 0 0 0 0 0.9 0.625 0.96

IL17A MPO 9606.ENSP00000344192 9606.ENSP00000225275 0 0 0 0 0 0 0 0.667 0.667

IL17A IL2 9606.ENSP00000344192 9606.ENSP00000226730 0 0 0 0 0.077 0.457 0 0.883 0.936

IL17A KITLG 9606.ENSP00000344192 9606.ENSP00000228280 0 0 0 0 0 0 0 0.421 0.42

IL17A IL4 9606.ENSP00000344192 9606.ENSP00000231449 0 0 0 0 0 0 0 0.914 0.914

IL17A IRF1 9606.ENSP00000344192 9606.ENSP00000245414 0 0 0 0 0 0 0 0.46 0.459

IL17A IL1RN 9606.ENSP00000344192 9606.ENSP00000259206 0 0 0 0 0 0 0 0.587 0.587

IL17A IL1A 9606.ENSP00000344192 9606.ENSP00000263339 0 0 0 0 0 0 0 0.807 0.807

IL17A IL1B 9606.ENSP00000344192 9606.ENSP00000263341 0 0 0 0 0 0 0 0.948 0.949

IL17A SELP 9606.ENSP00000344192 9606.ENSP00000263686 0 0 0 0 0 0 0 0.469 0.469

IL17A TP53 9606.ENSP00000344192 9606.ENSP00000269305 0 0 0 0 0.062 0 0 0.455 0.466

IL17A PPARG 9606.ENSP00000344192 9606.ENSP00000287820 0 0 0 0 0 0 0 0.505 0.505

IL17A KIT 9606.ENSP00000344192 9606.ENSP00000288135 0 0 0 0 0 0 0 0.514 0.514

IL17A VCAM1 9606.ENSP00000344192 9606.ENSP00000294728 0 0 0 0 0 0 0 0.603 0.603

IL17A TLR3 9606.ENSP00000344192 9606.ENSP00000296795 0 0 0 0 0 0 0 0.776 0.777

IL17A MMP3 9606.ENSP00000344192 9606.ENSP00000299855 0 0 0 0 0 0 0.9 0.576 0.955

IL17A NOD2 9606.ENSP00000344192 9606.ENSP00000300589 0 0 0 0 0 0 0 0.615 0.615

IL17A LEP 9606.ENSP00000344192 9606.ENSP00000312652 0 0 0 0 0 0 0 0.562 0.562

IL17A IL17RA 9606.ENSP00000344192 9606.ENSP00000320936 0 0 0 0 0 0.924 0.8 0.99 0.999

IL17A MMP1 9606.ENSP00000344192 9606.ENSP00000322788 0 0 0 0 0 0 0.9 0.495 0.947

IL17A NOS2 9606.ENSP00000344192 9606.ENSP00000327251 0 0 0 0 0 0 0 0.526 0.526

IL17A SOCS1 9606.ENSP00000344192 9606.ENSP00000329418 0 0 0 0 0 0 0 0.519 0.519

IL17A SELE 9606.ENSP00000344192 9606.ENSP00000331736 0 0 0 0 0 0 0 0.517 0.517

IL17A STAT5A 9606.ENSP00000344192 9606.ENSP00000341208 0 0 0 0 0 0.189 0 0.705 0.751

IL17A TNFAIP3 9606.ENSP00000344192 9606.ENSP00000481570 0 0 0 0 0 0 0 0.456 0.456

IL17A MTOR 9606.ENSP00000344192 9606.ENSP00000354558 0 0 0 0 0 0 0 0.463 0.463

IL17A NGF 9606.ENSP00000344192 9606.ENSP00000358525 0 0 0 0 0 0 0 0.464 0.463

IL17A ITGAL 9606.ENSP00000344192 9606.ENSP00000349252 0 0 0 0 0 0 0 0.466 0.465

IL17A VDR 9606.ENSP00000344192 9606.ENSP00000447173 0 0 0 0 0 0 0 0.465 0.465

IL17A TNFRSF1B 9606.ENSP00000344192 9606.ENSP00000365435 0 0 0 0 0 0 0 0.493 0.493

IL17A SPP1 9606.ENSP00000344192 9606.ENSP00000378517 0 0 0 0 0 0 0 0.502 0.502

IL17A OCLN 9606.ENSP00000344192 9606.ENSP00000347379 0 0 0 0 0 0 0 0.503 0.503

IL17A JUN 9606.ENSP00000344192 9606.ENSP00000360266 0 0 0 0 0 0 0 0.553 0.553

IL17A INS 9606.ENSP00000344192 9606.ENSP00000380432 0 0 0 0 0.065 0 0 0.558 0.569

IL17A MYD88 9606.ENSP00000344192 9606.ENSP00000401399 0 0 0 0 0 0 0 0.696 0.696

IL17A STAT1 9606.ENSP00000344192 9606.ENSP00000354394 0 0 0 0 0 0.189 0 0.693 0.741

IL17A TLR4 9606.ENSP00000344192 9606.ENSP00000363089 0 0 0 0 0.062 0 0 0.769 0.774

IL17A LTA 9606.ENSP00000344192 9606.ENSP00000403495 0 0 0 0 0 0 0 0.81 0.81

IL17A LCN2 9606.ENSP00000344192 9606.ENSP00000362108 0 0 0 0 0 0 0.9 0.602 0.958

IL17A MMP9 9606.ENSP00000344192 9606.ENSP00000361405 0 0 0 0 0 0 0.9 0.663 0.964

IL17A VEGFA 9606.ENSP00000344192 9606.ENSP00000478570 0 0 0 0 0 0 0.9 0.739 0.972

IL17A TNF 9606.ENSP00000344192 9606.ENSP00000398698 0 0 0 0 0 0 0 0.989 0.989

IL17A IL6 9606.ENSP00000344192 9606.ENSP00000385675 0 0 0 0 0.062 0 0.9 0.949 0.994

IL17RA TNFRSF1A 9606.ENSP00000320936 9606.ENSP00000162749 0 0 0 0 0.097 0 0 0.393 0.428

IL17RA MPO 9606.ENSP00000320936 9606.ENSP00000225275 0 0 0 0 0.065 0 0 0.401 0.416

IL17RA IL2 9606.ENSP00000320936 9606.ENSP00000226730 0 0 0 0 0 0 0 0.456 0.456

IL17RA IL4 9606.ENSP00000320936 9606.ENSP00000231449 0 0 0 0 0 0 0 0.56 0.56

IL17RA IL1A 9606.ENSP00000320936 9606.ENSP00000263339 0 0 0 0 0 0 0 0.401 0.4

IL17RA IL1B 9606.ENSP00000320936 9606.ENSP00000263341 0 0 0 0 0.09 0 0 0.611 0.631

IL17RA TLR4 9606.ENSP00000320936 9606.ENSP00000363089 0 0 0 0 0.069 0 0 0.393 0.41

IL17RA TNFRSF1B 9606.ENSP00000320936 9606.ENSP00000365435 0 0 0 0 0.098 0 0 0.402 0.437

IL17RA LCN2 9606.ENSP00000320936 9606.ENSP00000362108 0 0 0 0 0 0 0 0.457 0.457

IL17RA STAT1 9606.ENSP00000320936 9606.ENSP00000354394 0 0 0 0 0 0 0 0.505 0.505

IL17RA MYD88 9606.ENSP00000320936 9606.ENSP00000401399 0 0 0 0 0.063 0 0 0.582 0.592

IL17RA TNF 9606.ENSP00000320936 9606.ENSP00000398698 0 0 0 0 0.085 0 0 0.631 0.648

IL17RA IL6 9606.ENSP00000320936 9606.ENSP00000385675 0 0 0 0 0 0 0 0.674 0.674

IL1A TNFRSF1A 9606.ENSP00000263339 9606.ENSP00000162749 0 0 0 0 0.062 0 0 0.597 0.606

IL1A MIF 9606.ENSP00000263339 9606.ENSP00000215754 0 0 0 0 0 0 0 0.456 0.456

IL1A NFKBIA 9606.ENSP00000263339 9606.ENSP00000216797 0 0 0 0 0.087 0 0 0.602 0.621

IL1A MMP2 9606.ENSP00000263339 9606.ENSP00000219070 0 0 0 0 0 0 0 0.52 0.52

IL1A TGFB1 9606.ENSP00000263339 9606.ENSP00000221930 0 0 0 0 0 0 0 0.56 0.56

IL1A MPO 9606.ENSP00000263339 9606.ENSP00000225275 0 0 0 0 0.064 0 0 0.556 0.566

IL1A NFKB1 9606.ENSP00000263339 9606.ENSP00000226574 0 0 0 0 0.093 0 0.9 0.46 0.946

IL1A IL2 9606.ENSP00000263339 9606.ENSP00000226730 0 0 0 0 0 0 0 0.845 0.845

IL1A KITLG 9606.ENSP00000263339 9606.ENSP00000228280 0 0 0 0 0 0 0 0.499 0.499

IL1A IL4 9606.ENSP00000263339 9606.ENSP00000231449 0 0 0 0 0 0 0.9 0.845 0.983

IL1A IRF1 9606.ENSP00000263339 9606.ENSP00000245414 0 0 0 0 0.107 0 0 0.458 0.495

IL1A IL1RN 9606.ENSP00000263339 9606.ENSP00000259206 0 0 0 0 0.186 0 0.9 0.848 0.986

IL1A PF4 9606.ENSP00000263339 9606.ENSP00000296029 0 0 0 0 0.066 0 0 0.409 0.425

IL1A PPARG 9606.ENSP00000263339 9606.ENSP00000287820 0 0 0 0 0 0 0 0.457 0.457

IL1A SPP1 9606.ENSP00000263339 9606.ENSP00000378517 0 0 0 0 0.062 0 0 0.455 0.466

IL1A LCN2 9606.ENSP00000263339 9606.ENSP00000362108 0 0 0 0 0.062 0 0 0.457 0.468

IL1A SELP 9606.ENSP00000263339 9606.ENSP00000263686 0 0 0 0 0.062 0 0 0.46 0.471

IL1A NOS2 9606.ENSP00000263339 9606.ENSP00000327251 0 0 0 0 0.063 0 0 0.501 0.512

IL1A NGF 9606.ENSP00000263339 9606.ENSP00000358525 0 0 0 0 0 0 0 0.518 0.518

IL1A STAT1 9606.ENSP00000263339 9606.ENSP00000354394 0 0 0 0 0 0 0 0.519 0.519

IL1A NOD2 9606.ENSP00000263339 9606.ENSP00000300589 0 0 0 0 0.081 0 0 0.517 0.537

IL1A TNFRSF1B 9606.ENSP00000263339 9606.ENSP00000365435 0 0 0 0 0.083 0 0 0.517 0.538

IL1A INS 9606.ENSP00000263339 9606.ENSP00000380432 0 0 0 0 0 0 0 0.556 0.556

IL1A LEP 9606.ENSP00000263339 9606.ENSP00000312652 0 0 0 0 0 0 0 0.569 0.569

IL1A TNFAIP3 9606.ENSP00000263339 9606.ENSP00000481570 0 0 0 0 0.282 0 0 0.467 0.601

IL1A TLR3 9606.ENSP00000263339 9606.ENSP00000296795 0 0 0 0 0.062 0 0 0.602 0.61

IL1A SELE 9606.ENSP00000263339 9606.ENSP00000331736 0 0 0 0 0.062 0 0 0.61 0.618

IL1A MMP3 9606.ENSP00000263339 9606.ENSP00000299855 0 0 0 0 0.069 0 0 0.619 0.63

IL1A MMP1 9606.ENSP00000263339 9606.ENSP00000322788 0 0 0 0 0.111 0 0 0.609 0.637

IL1A VCAM1 9606.ENSP00000263339 9606.ENSP00000294728 0 0 0 0 0.062 0 0 0.636 0.643

IL1A TP53 9606.ENSP00000263339 9606.ENSP00000269305 0 0 0 0 0 0.128 0 0.61 0.645

IL1A MMP9 9606.ENSP00000263339 9606.ENSP00000361405 0 0 0 0 0.069 0 0 0.654 0.664

IL1A VEGFA 9606.ENSP00000263339 9606.ENSP00000478570 0 0 0 0 0 0 0 0.716 0.716

IL1A LTA 9606.ENSP00000263339 9606.ENSP00000403495 0 0 0 0 0.147 0 0 0.786 0.809

IL1A TLR4 9606.ENSP00000263339 9606.ENSP00000363089 0 0 0 0 0.062 0 0 0.841 0.844

IL1A JUN 9606.ENSP00000263339 9606.ENSP00000360266 0 0 0 0 0 0 0.9 0.632 0.961

IL1A TNF 9606.ENSP00000263339 9606.ENSP00000398698 0 0 0 0 0.289 0 0.5 0.917 0.968

IL1A MYD88 9606.ENSP00000263339 9606.ENSP00000401399 0 0 0 0 0.062 0 0.9 0.804 0.98

IL1A IL6 9606.ENSP00000263339 9606.ENSP00000385675 0 0 0 0 0.376 0 0.9 0.91 0.993

IL1A IL1B 9606.ENSP00000263339 9606.ENSP00000263341 0 0 0 0 0.761 0 0.8 0.916 0.995

IL1B TNFRSF1A 9606.ENSP00000263341 9606.ENSP00000162749 0 0 0 0 0.062 0 0 0.833 0.837

IL1B MIF 9606.ENSP00000263341 9606.ENSP00000215754 0 0 0 0 0 0 0 0.659 0.659

IL1B NFKBIA 9606.ENSP00000263341 9606.ENSP00000216797 0 0 0 0 0.155 0 0 0.866 0.882

IL1B MMP2 9606.ENSP00000263341 9606.ENSP00000219070 0 0 0 0 0 0.279 0 0.703 0.776

IL1B TGFB1 9606.ENSP00000263341 9606.ENSP00000221930 0 0 0 0 0.087 0 0 0.775 0.786

IL1B MPO 9606.ENSP00000263341 9606.ENSP00000225275 0 0 0 0 0.098 0 0 0.833 0.843

IL1B NFKB1 9606.ENSP00000263341 9606.ENSP00000226574 0 0 0 0 0.13 0 0.9 0.656 0.967

IL1B IL2 9606.ENSP00000263341 9606.ENSP00000226730 0 0 0 0 0 0 0 0.876 0.876

IL1B KITLG 9606.ENSP00000263341 9606.ENSP00000228280 0 0 0 0 0 0 0 0.616 0.616

IL1B IL4 9606.ENSP00000263341 9606.ENSP00000231449 0 0 0 0 0 0 0.9 0.91 0.99

IL1B NR3C1 9606.ENSP00000263341 9606.ENSP00000231509 0 0 0 0 0 0 0 0.601 0.601

IL1B TFPI 9606.ENSP00000263341 9606.ENSP00000233156 0 0 0 0 0 0 0 0.451 0.451

IL1B IRF1 9606.ENSP00000263341 9606.ENSP00000245414 0 0 0 0 0.203 0 0 0.622 0.686

IL1B IL1RN 9606.ENSP00000263341 9606.ENSP00000259206 0 0 0 0.656 0.396 0 0.9 0.858 0.955

IL1B LYZ 9606.ENSP00000263341 9606.ENSP00000261267 0 0 0 0 0.13 0 0 0.434 0.487

IL1B SLC6A4 9606.ENSP00000263341 9606.ENSP00000261707 0 0 0 0 0 0 0 0.43 0.43

IL1B SMAD7 9606.ENSP00000263341 9606.ENSP00000262158 0 0 0 0 0 0 0 0.464 0.463

IL1B SST 9606.ENSP00000263341 9606.ENSP00000287641 0 0 0 0 0 0 0 0.401 0.4

IL1B XDH 9606.ENSP00000263341 9606.ENSP00000368727 0 0 0 0 0.063 0 0 0.403 0.417

IL1B SCN11A 9606.ENSP00000263341 9606.ENSP00000307599 0 0 0 0 0 0 0 0.442 0.442

IL1B PARP1 9606.ENSP00000263341 9606.ENSP00000355759 0 0 0 0 0 0 0 0.456 0.456

IL1B LPL 9606.ENSP00000263341 9606.ENSP00000309757 0 0 0 0 0.062 0 0 0.459 0.47

IL1B PRKCD 9606.ENSP00000263341 9606.ENSP00000378217 0 0 0 0 0.076 0 0 0.459 0.478

IL1B PTEN 9606.ENSP00000263341 9606.ENSP00000361021 0 0 0 0 0 0 0 0.501 0.501

IL1B KIT 9606.ENSP00000263341 9606.ENSP00000288135 0 0 0 0 0 0 0 0.52 0.52

IL1B TH 9606.ENSP00000263341 9606.ENSP00000370571 0 0 0 0 0 0 0 0.521 0.521

IL1B VDR 9606.ENSP00000263341 9606.ENSP00000447173 0 0 0 0 0.069 0 0 0.508 0.522

IL1B ITGAL 9606.ENSP00000263341 9606.ENSP00000349252 0 0 0 0 0.1 0 0 0.504 0.534

IL1B SREBF1 9606.ENSP00000263341 9606.ENSP00000348069 0 0 0 0 0 0 0 0.535 0.535

IL1B ITGB2 9606.ENSP00000263341 9606.ENSP00000380948 0 0 0 0 0.201 0 0 0.469 0.558

IL1B STAT5A 9606.ENSP00000263341 9606.ENSP00000341208 0 0 0 0 0.089 0 0 0.538 0.561

IL1B SOD1 9606.ENSP00000263341 9606.ENSP00000270142 0 0 0 0 0 0 0 0.562 0.562

IL1B REN 9606.ENSP00000263341 9606.ENSP00000272190 0 0 0 0 0 0 0 0.564 0.564

IL1B SNCA 9606.ENSP00000263341 9606.ENSP00000338345 0 0 0 0 0 0 0 0.568 0.568

IL1B NOS1 9606.ENSP00000263341 9606.ENSP00000477999 0 0 0 0 0.062 0 0 0.567 0.577

IL1B PF4 9606.ENSP00000263341 9606.ENSP00000296029 0 0 0 0 0.108 0 0 0.572 0.602

IL1B MTOR 9606.ENSP00000263341 9606.ENSP00000354558 0 0 0 0 0 0 0 0.602 0.602

IL1B THBD 9606.ENSP00000263341 9606.ENSP00000366307 0 0 0 0 0.153 0 0 0.556 0.607

IL1B S100A9 9606.ENSP00000263341 9606.ENSP00000357727 0 0 0 0 0.301 0 0 0.464 0.609

IL1B NCF1 9606.ENSP00000263341 9606.ENSP00000289473 0 0 0 0 0.145 0 0 0.563 0.611

IL1B SOCS1 9606.ENSP00000263341 9606.ENSP00000329418 0 0 0 0 0.09 0 0 0.603 0.623

IL1B SPP1 9606.ENSP00000263341 9606.ENSP00000378517 0 0 0 0 0.062 0 0 0.617 0.625

IL1B TNFRSF1B 9606.ENSP00000263341 9606.ENSP00000365435 0 0 0 0 0.177 0 0 0.612 0.667

IL1B LCN2 9606.ENSP00000263341 9606.ENSP00000362108 0 0 0 0 0.098 0 0 0.651 0.671

IL1B S100A8 9606.ENSP00000263341 9606.ENSP00000357722 0 0 0 0 0.308 0 0 0.56 0.682

IL1B OCLN 9606.ENSP00000263341 9606.ENSP00000347379 0 0 0 0 0 0 0 0.686 0.686

IL1B TP53 9606.ENSP00000263341 9606.ENSP00000269305 0 0 0 0 0 0 0 0.689 0.689

IL1B NOS3 9606.ENSP00000263341 9606.ENSP00000297494 0 0 0 0 0.062 0 0 0.689 0.696

IL1B NGF 9606.ENSP00000263341 9606.ENSP00000358525 0 0 0 0 0 0 0 0.704 0.704

IL1B TNFAIP3 9606.ENSP00000263341 9606.ENSP00000481570 0 0 0 0 0.298 0.182 0 0.56 0.725

IL1B SELP 9606.ENSP00000263341 9606.ENSP00000263686 0 0 0 0 0.098 0 0 0.711 0.728

IL1B PPARG 9606.ENSP00000263341 9606.ENSP00000287820 0 0 0 0 0.062 0 0 0.739 0.745

IL1B STAT1 9606.ENSP00000263341 9606.ENSP00000354394 0 0 0 0 0.062 0 0 0.74 0.746

IL1B SELE 9606.ENSP00000263341 9606.ENSP00000331736 0 0 0 0 0.062 0 0 0.753 0.758

IL1B MMP1 9606.ENSP00000263341 9606.ENSP00000322788 0 0 0 0 0.128 0.058 0 0.742 0.77

IL1B NOD2 9606.ENSP00000263341 9606.ENSP00000300589 0 0 0 0 0.19 0 0 0.743 0.783

IL1B NOS2 9606.ENSP00000263341 9606.ENSP00000327251 0 0 0 0 0.068 0 0 0.807 0.813

IL1B INS 9606.ENSP00000263341 9606.ENSP00000380432 0 0 0 0 0 0 0 0.816 0.816

IL1B TLR3 9606.ENSP00000263341 9606.ENSP00000296795 0 0 0 0 0.062 0 0 0.817 0.821

IL1B MMP3 9606.ENSP00000263341 9606.ENSP00000299855 0 0 0 0 0.107 0.058 0 0.809 0.825

IL1B VCAM1 9606.ENSP00000263341 9606.ENSP00000294728 0 0 0 0 0.062 0 0 0.826 0.83

IL1B VEGFA 9606.ENSP00000263341 9606.ENSP00000478570 0 0 0 0 0 0 0 0.857 0.857

IL1B MMP9 9606.ENSP00000263341 9606.ENSP00000361405 0 0 0 0 0.2 0.058 0 0.856 0.882

IL1B LEP 9606.ENSP00000263341 9606.ENSP00000312652 0 0 0 0 0 0 0 0.913 0.913

IL1B LTA 9606.ENSP00000263341 9606.ENSP00000403495 0 0 0 0 0.243 0 0 0.905 0.925

IL1B TLR4 9606.ENSP00000263341 9606.ENSP00000363089 0 0 0 0 0.16 0 0 0.917 0.927

IL1B JUN 9606.ENSP00000263341 9606.ENSP00000360266 0 0 0 0 0.052 0 0.8 0.792 0.957

IL1B TNF 9606.ENSP00000263341 9606.ENSP00000398698 0 0 0 0 0.462 0 0.5 0.942 0.983

IL1B MYD88 9606.ENSP00000263341 9606.ENSP00000401399 0 0 0 0 0.097 0 0.9 0.917 0.991

IL1B IL6 9606.ENSP00000263341 9606.ENSP00000385675 0 0 0 0 0.427 0 0.9 0.953 0.997

IL1RN TNFRSF1A 9606.ENSP00000259206 9606.ENSP00000162749 0 0 0 0 0.068 0 0 0.519 0.532

IL1RN NFKBIA 9606.ENSP00000259206 9606.ENSP00000216797 0 0 0 0 0.087 0 0 0.392 0.421

IL1RN TGFB1 9606.ENSP00000259206 9606.ENSP00000221930 0 0 0 0 0.088 0 0 0.504 0.528

IL1RN IL2 9606.ENSP00000259206 9606.ENSP00000226730 0 0 0 0 0 0 0 0.564 0.564

IL1RN IL4 9606.ENSP00000259206 9606.ENSP00000231449 0 0 0 0 0 0 0 0.61 0.61

IL1RN IRF1 9606.ENSP00000259206 9606.ENSP00000245414 0 0 0 0 0.145 0 0 0.33 0.403

IL1RN LEP 9606.ENSP00000259206 9606.ENSP00000312652 0 0 0 0 0 0 0 0.402 0.402

IL1RN LCN2 9606.ENSP00000259206 9606.ENSP00000362108 0 0 0 0 0.158 0 0 0.323 0.406

IL1RN VCAM1 9606.ENSP00000259206 9606.ENSP00000294728 0 0 0 0 0.062 0 0 0.41 0.423

IL1RN MMP1 9606.ENSP00000259206 9606.ENSP00000322788 0 0 0 0 0.086 0.058 0 0.393 0.432

IL1RN INS 9606.ENSP00000259206 9606.ENSP00000380432 0 0 0 0 0 0 0 0.462 0.462

IL1RN S100A9 9606.ENSP00000259206 9606.ENSP00000357727 0 0 0 0 0.351 0 0 0.207 0.464

IL1RN MMP3 9606.ENSP00000259206 9606.ENSP00000299855 0 0 0 0 0.075 0.058 0 0.457 0.485

IL1RN TNFAIP3 9606.ENSP00000259206 9606.ENSP00000481570 0 0 0 0 0.17 0.078 0 0.385 0.488

IL1RN VEGFA 9606.ENSP00000259206 9606.ENSP00000478570 0 0 0 0 0 0 0 0.496 0.495

IL1RN LTA 9606.ENSP00000259206 9606.ENSP00000403495 0 0 0 0 0.085 0 0 0.493 0.517

IL1RN TLR4 9606.ENSP00000259206 9606.ENSP00000363089 0 0 0 0 0.098 0 0 0.53 0.557

IL1RN NOD2 9606.ENSP00000259206 9606.ENSP00000300589 0 0 0 0 0.332 0 0 0.421 0.596

IL1RN MMP9 9606.ENSP00000259206 9606.ENSP00000361405 0 0 0 0 0.282 0.058 0 0.456 0.599

IL1RN S100A8 9606.ENSP00000259206 9606.ENSP00000357722 0 0 0 0 0.37 0 0 0.459 0.645

IL1RN TNF 9606.ENSP00000259206 9606.ENSP00000398698 0 0 0 0 0.201 0 0 0.733 0.778

IL1RN IL6 9606.ENSP00000259206 9606.ENSP00000385675 0 0 0 0 0.112 0 0 0.789 0.805

IL1RN MYD88 9606.ENSP00000259206 9606.ENSP00000401399 0 0 0 0 0.116 0 0.9 0.434 0.945

IL1RN TNFRSF1B 9606.ENSP00000259206 9606.ENSP00000365435 0 0 0 0 0.134 0 0.9 0.501 0.953

IL2 TNFRSF1A 9606.ENSP00000226730 9606.ENSP00000162749 0 0 0 0 0 0 0 0.612 0.612

IL2 MIF 9606.ENSP00000226730 9606.ENSP00000215754 0 0 0 0 0 0 0 0.469 0.469

IL2 NFKBIA 9606.ENSP00000226730 9606.ENSP00000216797 0 0 0 0 0 0 0 0.559 0.559

IL2 MMP2 9606.ENSP00000226730 9606.ENSP00000219070 0 0 0 0 0 0 0 0.424 0.424

IL2 TGFB1 9606.ENSP00000226730 9606.ENSP00000221930 0 0 0 0 0 0 0 0.693 0.694

IL2 MPO 9606.ENSP00000226730 9606.ENSP00000225275 0 0 0 0 0 0 0 0.516 0.516

IL2 NFKB1 9606.ENSP00000226730 9606.ENSP00000226574 0 0 0 0 0 0 0 0.414 0.414

IL2 VDR 9606.ENSP00000226730 9606.ENSP00000447173 0 0 0 0 0 0 0 0.413 0.412

IL2 MMP3 9606.ENSP00000226730 9606.ENSP00000299855 0 0 0 0 0 0 0 0.422 0.422

IL2 NR3C1 9606.ENSP00000226730 9606.ENSP00000231509 0 0 0 0 0 0 0 0.422 0.422

IL2 ITGB2 9606.ENSP00000226730 9606.ENSP00000380948 0 0 0 0 0 0 0 0.426 0.426

IL2 PARP1 9606.ENSP00000226730 9606.ENSP00000355759 0 0 0 0 0 0 0 0.45 0.45

IL2 PPARG 9606.ENSP00000226730 9606.ENSP00000287820 0 0 0 0 0 0 0 0.46 0.459

IL2 NOD2 9606.ENSP00000226730 9606.ENSP00000300589 0 0 0 0 0 0 0 0.46 0.459

IL2 MDM2 9606.ENSP00000226730 9606.ENSP00000258149 0 0 0 0 0 0 0 0.474 0.474

IL2 PTEN 9606.ENSP00000226730 9606.ENSP00000361021 0 0 0 0 0 0 0 0.479 0.479

IL2 NOS2 9606.ENSP00000226730 9606.ENSP00000327251 0 0 0 0 0 0 0 0.487 0.487

IL2 IRF1 9606.ENSP00000226730 9606.ENSP00000245414 0 0 0 0 0 0 0 0.503 0.503

IL2 NGF 9606.ENSP00000226730 9606.ENSP00000358525 0 0 0 0 0 0 0 0.504 0.504

IL2 SELP 9606.ENSP00000226730 9606.ENSP00000263686 0 0 0 0 0 0 0 0.505 0.505

IL2 TNFRSF1B 9606.ENSP00000226730 9606.ENSP00000365435 0 0 0 0 0 0 0 0.519 0.519

IL2 SELE 9606.ENSP00000226730 9606.ENSP00000331736 0 0 0 0 0 0 0 0.533 0.533

IL2 MMP9 9606.ENSP00000226730 9606.ENSP00000361405 0 0 0 0 0 0 0 0.56 0.56

IL2 KIT 9606.ENSP00000226730 9606.ENSP00000288135 0 0 0 0 0 0 0 0.561 0.561

IL2 LEP 9606.ENSP00000226730 9606.ENSP00000312652 0 0 0 0 0 0 0 0.564 0.564

IL2 MYD88 9606.ENSP00000226730 9606.ENSP00000401399 0 0 0 0 0 0 0 0.602 0.602

IL2 VCAM1 9606.ENSP00000226730 9606.ENSP00000294728 0 0 0 0 0 0 0 0.603 0.603

IL2 TLR3 9606.ENSP00000226730 9606.ENSP00000296795 0 0 0 0 0 0 0 0.604 0.604

IL2 ITGAL 9606.ENSP00000226730 9606.ENSP00000349252 0 0 0 0 0 0 0 0.631 0.631

IL2 INS 9606.ENSP00000226730 9606.ENSP00000380432 0 0 0 0 0 0 0 0.656 0.656

IL2 TP53 9606.ENSP00000226730 9606.ENSP00000269305 0 0 0 0 0 0 0 0.667 0.667

IL2 TLR4 9606.ENSP00000226730 9606.ENSP00000363089 0 0 0 0 0 0 0 0.693 0.693

IL2 KITLG 9606.ENSP00000226730 9606.ENSP00000228280 0 0 0 0 0 0 0 0.737 0.737

IL2 VEGFA 9606.ENSP00000226730 9606.ENSP00000478570 0 0 0 0 0 0 0 0.753 0.753

IL2 LTA 9606.ENSP00000226730 9606.ENSP00000403495 0 0 0 0 0.069 0 0 0.812 0.817

IL2 IL6 9606.ENSP00000226730 9606.ENSP00000385675 0 0 0 0 0 0 0 0.914 0.914

IL2 PRKCB 9606.ENSP00000226730 9606.ENSP00000305355 0 0 0 0 0 0 0.9 0.204 0.916

IL2 TNF 9606.ENSP00000226730 9606.ENSP00000398698 0 0 0 0 0 0 0 0.933 0.933

IL2 IL4 9606.ENSP00000226730 9606.ENSP00000231449 0 0 0 0 0.063 0 0 0.936 0.938

IL2 SOCS1 9606.ENSP00000226730 9606.ENSP00000329418 0 0 0 0 0 0 0.9 0.605 0.958

IL2 MTOR 9606.ENSP00000226730 9606.ENSP00000354558 0 0 0 0 0 0 0.9 0.607 0.959

IL2 STAT1 9606.ENSP00000226730 9606.ENSP00000354394 0 0 0 0 0 0 0.9 0.702 0.968

IL2 JUN 9606.ENSP00000226730 9606.ENSP00000360266 0 0 0 0 0 0.27 0.9 0.619 0.969

IL2 STAT5A 9606.ENSP00000226730 9606.ENSP00000341208 0 0 0 0 0 0 0.9 0.837 0.983

IL4 TNFRSF1A 9606.ENSP00000231449 9606.ENSP00000162749 0 0 0 0 0 0 0 0.567 0.567

IL4 MIF 9606.ENSP00000231449 9606.ENSP00000215754 0 0 0 0 0 0 0 0.505 0.505

IL4 NFKBIA 9606.ENSP00000231449 9606.ENSP00000216797 0 0 0 0 0 0 0 0.575 0.575

IL4 MMP2 9606.ENSP00000231449 9606.ENSP00000219070 0 0 0 0 0 0 0 0.468 0.468

IL4 TGFB1 9606.ENSP00000231449 9606.ENSP00000221930 0 0 0 0 0 0 0 0.766 0.766

IL4 MPO 9606.ENSP00000231449 9606.ENSP00000225275 0 0 0 0 0 0 0 0.6 0.6

IL4 NFKB1 9606.ENSP00000231449 9606.ENSP00000226574 0 0 0 0 0 0 0 0.406 0.406

IL4 KITLG 9606.ENSP00000231449 9606.ENSP00000228280 0 0 0 0 0.062 0 0 0.651 0.658

IL4 PAX5 9606.ENSP00000231449 9606.ENSP00000350844 0 0 0 0 0 0 0 0.402 0.403

IL4 NOS3 9606.ENSP00000231449 9606.ENSP00000297494 0 0 0 0 0.062 0 0 0.395 0.408

IL4 OCLN 9606.ENSP00000231449 9606.ENSP00000347379 0 0 0 0 0 0 0 0.408 0.408

IL4 PF4 9606.ENSP00000231449 9606.ENSP00000296029 0 0 0 0 0 0 0 0.422 0.422

IL4 PTEN 9606.ENSP00000231449 9606.ENSP00000361021 0 0 0 0 0.062 0 0 0.427 0.439

IL4 THBD 9606.ENSP00000231449 9606.ENSP00000366307 0 0 0 0 0 0 0 0.456 0.456

IL4 NR3C1 9606.ENSP00000231449 9606.ENSP00000231509 0 0 0 0 0 0 0 0.459 0.459

IL4 MMP1 9606.ENSP00000231449 9606.ENSP00000322788 0 0 0 0 0 0 0 0.46 0.46

IL4 VDR 9606.ENSP00000231449 9606.ENSP00000447173 0 0 0 0 0 0 0 0.464 0.463

IL4 TNFRSF1B 9606.ENSP00000231449 9606.ENSP00000365435 0 0 0 0 0 0 0 0.468 0.468

IL4 NOD2 9606.ENSP00000231449 9606.ENSP00000300589 0 0 0 0 0 0 0 0.509 0.509

IL4 TP53 9606.ENSP00000231449 9606.ENSP00000269305 0 0 0 0 0 0 0 0.509 0.509

IL4 MMP3 9606.ENSP00000231449 9606.ENSP00000299855 0 0 0 0 0 0 0 0.519 0.519

IL4 ITGAL 9606.ENSP00000231449 9606.ENSP00000349252 0 0 0 0 0 0 0 0.519 0.519

IL4 SELP 9606.ENSP00000231449 9606.ENSP00000263686 0 0 0 0 0.062 0 0 0.519 0.529

IL4 SPP1 9606.ENSP00000231449 9606.ENSP00000378517 0 0 0 0 0 0 0 0.535 0.535

IL4 IRF1 9606.ENSP00000231449 9606.ENSP00000245414 0 0 0 0 0 0 0 0.556 0.556

IL4 KIT 9606.ENSP00000231449 9606.ENSP00000288135 0 0 0 0 0 0 0 0.571 0.571

IL4 SELE 9606.ENSP00000231449 9606.ENSP00000331736 0 0 0 0 0.055 0 0 0.565 0.571

IL4 NGF 9606.ENSP00000231449 9606.ENSP00000358525 0 0 0 0 0.062 0 0 0.567 0.576

IL4 LEP 9606.ENSP00000231449 9606.ENSP00000312652 0 0 0 0 0.064 0 0 0.61 0.62

IL4 INS 9606.ENSP00000231449 9606.ENSP00000380432 0 0 0 0 0 0 0 0.637 0.637

IL4 PPARG 9606.ENSP00000231449 9606.ENSP00000287820 0 0 0 0 0.063 0 0 0.654 0.662

IL4 MMP9 9606.ENSP00000231449 9606.ENSP00000361405 0 0 0 0 0.063 0 0 0.659 0.667

IL4 TLR3 9606.ENSP00000231449 9606.ENSP00000296795 0 0 0 0 0 0 0 0.668 0.668

IL4 NOS2 9606.ENSP00000231449 9606.ENSP00000327251 0 0 0 0 0.065 0 0 0.668 0.676

IL4 MYD88 9606.ENSP00000231449 9606.ENSP00000401399 0 0 0 0 0 0 0 0.682 0.682

IL4 MTOR 9606.ENSP00000231449 9606.ENSP00000354558 0 0 0 0 0 0.34 0 0.548 0.689

IL4 VCAM1 9606.ENSP00000231449 9606.ENSP00000294728 0 0 0 0 0 0 0 0.701 0.702

IL4 STAT1 9606.ENSP00000231449 9606.ENSP00000354394 0 0 0 0 0 0 0 0.717 0.717

IL4 VEGFA 9606.ENSP00000231449 9606.ENSP00000478570 0 0 0 0 0 0 0 0.767 0.767

IL4 TLR4 9606.ENSP00000231449 9606.ENSP00000363089 0 0 0 0 0.064 0 0 0.784 0.789

IL4 LTA 9606.ENSP00000231449 9606.ENSP00000403495 0 0 0 0 0 0 0 0.803 0.803

IL4 JUN 9606.ENSP00000231449 9606.ENSP00000360266 0 0 0 0 0 0 0.9 0.603 0.958

IL4 SOCS1 9606.ENSP00000231449 9606.ENSP00000329418 0 0 0 0 0 0 0.9 0.613 0.959

IL4 STAT5A 9606.ENSP00000231449 9606.ENSP00000341208 0 0 0 0 0 0 0.9 0.689 0.967

IL4 IL6 9606.ENSP00000231449 9606.ENSP00000385675 0 0 0 0 0 0 0.9 0.916 0.991

IL4 TNF 9606.ENSP00000231449 9606.ENSP00000398698 0 0 0 0 0 0 0.9 0.933 0.993

IL6 TNFRSF1A 9606.ENSP00000385675 9606.ENSP00000162749 0 0 0 0 0 0 0 0.74 0.74

IL6 MIF 9606.ENSP00000385675 9606.ENSP00000215754 0 0 0 0 0 0 0 0.687 0.687

IL6 NFKBIA 9606.ENSP00000385675 9606.ENSP00000216797 0 0 0 0 0.11 0 0 0.862 0.872

IL6 MMP2 9606.ENSP00000385675 9606.ENSP00000219070 0 0 0 0 0.076 0 0.9 0.715 0.971

IL6 TGFB1 9606.ENSP00000385675 9606.ENSP00000221930 0 0 0 0 0.065 0 0.9 0.901 0.99

IL6 PON1 9606.ENSP00000385675 9606.ENSP00000222381 0 0 0 0 0 0 0 0.505 0.505

IL6 MPO 9606.ENSP00000385675 9606.ENSP00000225275 0 0 0 0 0.062 0 0 0.839 0.843

IL6 NFKB1 9606.ENSP00000385675 9606.ENSP00000226574 0 0 0 0 0.086 0 0.9 0.644 0.964

IL6 KITLG 9606.ENSP00000385675 9606.ENSP00000228280 0 0 0 0 0 0 0 0.861 0.861

IL6 NR3C1 9606.ENSP00000385675 9606.ENSP00000231509 0 0 0 0 0 0 0 0.624 0.624

IL6 IRF1 9606.ENSP00000385675 9606.ENSP00000245414 0 0 0 0 0.082 0 0 0.643 0.658

IL6 MDM2 9606.ENSP00000385675 9606.ENSP00000258149 0 0 0 0 0 0 0 0.429 0.429

IL6 ORM1 9606.ENSP00000385675 9606.ENSP00000259396 0 0 0 0 0 0 0 0.419 0.419

IL6 SLC6A4 9606.ENSP00000385675 9606.ENSP00000261707 0 0 0 0 0 0 0 0.598 0.598

IL6 SMAD7 9606.ENSP00000385675 9606.ENSP00000262158 0 0 0 0 0 0 0 0.504 0.504

IL6 SELP 9606.ENSP00000385675 9606.ENSP00000263686 0 0 0 0 0.063 0 0 0.71 0.716

IL6 TP53 9606.ENSP00000385675 9606.ENSP00000269305 0 0 0 0 0.062 0 0 0.738 0.744

IL6 SOD1 9606.ENSP00000385675 9606.ENSP00000270142 0 0 0 0 0 0 0 0.535 0.535

IL6 REN 9606.ENSP00000385675 9606.ENSP00000272190 0 0 0 0 0 0 0 0.819 0.819

IL6 SST 9606.ENSP00000385675 9606.ENSP00000287641 0 0 0 0 0 0 0 0.425 0.424

IL6 PPARG 9606.ENSP00000385675 9606.ENSP00000287820 0 0 0 0 0 0 0 0.792 0.792

IL6 KIT 9606.ENSP00000385675 9606.ENSP00000288135 0 0 0 0 0 0 0 0.664 0.664

IL6 NCF1 9606.ENSP00000385675 9606.ENSP00000289473 0 0 0 0 0.062 0 0 0.537 0.547

IL6 VCAM1 9606.ENSP00000385675 9606.ENSP00000294728 0 0 0 0 0.083 0 0 0.86 0.866

IL6 PF4 9606.ENSP00000385675 9606.ENSP00000296029 0 0 0 0 0.066 0 0 0.587 0.598

IL6 TLR3 9606.ENSP00000385675 9606.ENSP00000296795 0 0 0 0 0 0 0 0.833 0.833

IL6 NOS3 9606.ENSP00000385675 9606.ENSP00000297494 0 0 0 0 0.062 0 0 0.717 0.724

IL6 MMP3 9606.ENSP00000385675 9606.ENSP00000299855 0 0 0 0 0.112 0 0.9 0.768 0.977

IL6 NOD2 9606.ENSP00000385675 9606.ENSP00000300589 0 0 0 0 0.064 0 0 0.683 0.69

IL6 LPL 9606.ENSP00000385675 9606.ENSP00000309757 0 0 0 0 0 0 0 0.571 0.571

IL6 PPARD 9606.ENSP00000385675 9606.ENSP00000310928 0 0 0 0 0 0 0 0.415 0.415

IL6 LEP 9606.ENSP00000385675 9606.ENSP00000312652 0 0 0 0 0 0 0 0.877 0.877

IL6 MMP1 9606.ENSP00000385675 9606.ENSP00000322788 0 0 0 0 0.154 0 0.9 0.71 0.973

IL6 NOS2 9606.ENSP00000385675 9606.ENSP00000327251 0 0 0 0 0.063 0 0 0.784 0.789

IL6 SOCS1 9606.ENSP00000385675 9606.ENSP00000329418 0 0 0 0 0.091 0 0 0.712 0.727

IL6 SELE 9606.ENSP00000385675 9606.ENSP00000331736 0 0 0 0 0.121 0 0 0.803 0.819

IL6 SNCA 9606.ENSP00000385675 9606.ENSP00000338345 0 0 0 0 0 0 0 0.509 0.509

IL6 STAT5A 9606.ENSP00000385675 9606.ENSP00000341208 0 0 0 0 0.049 0.117 0 0.737 0.76

IL6 OCLN 9606.ENSP00000385675 9606.ENSP00000347379 0 0 0 0 0 0 0 0.669 0.669

IL6 SREBF1 9606.ENSP00000385675 9606.ENSP00000348069 0 0 0 0 0 0 0 0.616 0.616

IL6 ITGAL 9606.ENSP00000385675 9606.ENSP00000349252 0 0 0 0 0.062 0 0 0.519 0.529

IL6 NR3C2 9606.ENSP00000385675 9606.ENSP00000350815 0 0 0 0 0 0 0 0.417 0.417

IL6 STAT1 9606.ENSP00000385675 9606.ENSP00000354394 0 0 0 0 0.062 0.117 0.9 0.846 0.985

IL6 MTOR 9606.ENSP00000385675 9606.ENSP00000354558 0 0 0 0 0 0 0 0.687 0.687

IL6 PARP1 9606.ENSP00000385675 9606.ENSP00000355759 0 0 0 0 0 0 0 0.429 0.429

IL6 S100A8 9606.ENSP00000385675 9606.ENSP00000357722 0 0 0 0 0 0 0 0.506 0.506

IL6 S100A9 9606.ENSP00000385675 9606.ENSP00000357727 0 0 0 0 0 0 0 0.452 0.452

IL6 NGF 9606.ENSP00000385675 9606.ENSP00000358525 0 0 0 0 0.077 0 0 0.727 0.737

IL6 JUN 9606.ENSP00000385675 9606.ENSP00000360266 0 0 0 0 0.052 0 0.9 0.822 0.981

IL6 PTEN 9606.ENSP00000385675 9606.ENSP00000361021 0 0 0 0 0.056 0 0 0.63 0.636

IL6 MMP9 9606.ENSP00000385675 9606.ENSP00000361405 0 0 0 0 0.075 0 0.9 0.861 0.986

IL6 LCN2 9606.ENSP00000385675 9606.ENSP00000362108 0 0 0 0 0.062 0 0.9 0.684 0.967

IL6 TLR4 9606.ENSP00000385675 9606.ENSP00000363089 0 0 0 0 0.081 0 0 0.937 0.94

IL6 TNFRSF1B 9606.ENSP00000385675 9606.ENSP00000365435 0 0 0 0 0.065 0 0 0.683 0.69

IL6 THBD 9606.ENSP00000385675 9606.ENSP00000366307 0 0 0 0 0.098 0 0 0.617 0.639

IL6 XDH 9606.ENSP00000385675 9606.ENSP00000368727 0 0 0 0 0 0 0 0.404 0.404

IL6 SHBG 9606.ENSP00000385675 9606.ENSP00000369816 0 0 0 0 0 0 0 0.504 0.504

IL6 TH 9606.ENSP00000385675 9606.ENSP00000370571 0 0 0 0 0 0 0 0.502 0.502

IL6 PRKCD 9606.ENSP00000385675 9606.ENSP00000378217 0 0 0 0 0 0 0 0.459 0.459

IL6 SPP1 9606.ENSP00000385675 9606.ENSP00000378517 0 0 0 0 0.062 0 0 0.687 0.694

IL6 INS 9606.ENSP00000385675 9606.ENSP00000380432 0 0 0 0 0 0 0 0.88 0.88

IL6 ITGB2 9606.ENSP00000385675 9606.ENSP00000380948 0 0 0 0 0.053 0 0 0.46 0.466

IL6 NOS1 9606.ENSP00000385675 9606.ENSP00000477999 0 0 0 0 0.062 0 0 0.523 0.534

IL6 VDR 9606.ENSP00000385675 9606.ENSP00000447173 0 0 0 0 0 0 0 0.602 0.602

IL6 TNFAIP3 9606.ENSP00000385675 9606.ENSP00000481570 0 0 0 0 0.221 0 0 0.56 0.642

IL6 LTA 9606.ENSP00000385675 9606.ENSP00000403495 0 0 0 0 0.083 0 0 0.832 0.839

IL6 MYD88 9606.ENSP00000385675 9606.ENSP00000401399 0 0 0 0 0.062 0 0 0.861 0.864

IL6 VEGFA 9606.ENSP00000385675 9606.ENSP00000478570 0 0 0 0 0.063 0 0.9 0.879 0.987

IL6 TNF 9606.ENSP00000385675 9606.ENSP00000398698 0 0 0 0 0.125 0 0.9 0.941 0.994

INS TNFRSF1A 9606.ENSP00000380432 9606.ENSP00000162749 0 0 0 0 0 0 0 0.492 0.491

INS MIF 9606.ENSP00000380432 9606.ENSP00000215754 0 0 0 0 0.062 0 0 0.455 0.466

INS NFKBIA 9606.ENSP00000380432 9606.ENSP00000216797 0 0 0 0 0 0 0 0.568 0.568

INS MMP2 9606.ENSP00000380432 9606.ENSP00000219070 0 0 0 0 0 0 0 0.556 0.556

INS TGFB1 9606.ENSP00000380432 9606.ENSP00000221930 0 0 0 0 0 0 0 0.637 0.638

INS PON1 9606.ENSP00000380432 9606.ENSP00000222381 0 0 0 0 0 0 0 0.53 0.53

INS MPO 9606.ENSP00000380432 9606.ENSP00000225275 0 0 0 0 0 0 0 0.507 0.507

INS KITLG 9606.ENSP00000380432 9606.ENSP00000228280 0 0 0 0 0 0 0 0.586 0.586

INS NR3C1 9606.ENSP00000380432 9606.ENSP00000231509 0 0 0 0 0 0 0 0.708 0.708

INS LTF 9606.ENSP00000380432 9606.ENSP00000231751 0 0 0 0 0 0 0.5 0.245 0.606

INS NR1D1 9606.ENSP00000380432 9606.ENSP00000246672 0 0 0 0 0 0 0 0.469 0.469

INS MDM2 9606.ENSP00000380432 9606.ENSP00000258149 0 0 0 0 0 0 0 0.47 0.47

INS LYZ 9606.ENSP00000380432 9606.ENSP00000261267 0 0 0 0 0 0 0.5 0.165 0.564

INS SELP 9606.ENSP00000380432 9606.ENSP00000263686 0 0 0 0 0 0 0 0.505 0.505

INS TP53 9606.ENSP00000380432 9606.ENSP00000269305 0 0 0 0 0 0 0 0.717 0.717

INS SOD1 9606.ENSP00000380432 9606.ENSP00000270142 0 0 0 0 0 0 0 0.501 0.501

INS REN 9606.ENSP00000380432 9606.ENSP00000272190 0 0 0 0 0.062 0 0 0.833 0.837

INS SST 9606.ENSP00000380432 9606.ENSP00000287641 0 0 0 0 0.098 0 0 0.897 0.903

INS PPARG 9606.ENSP00000380432 9606.ENSP00000287820 0 0 0 0 0 0 0 0.887 0.887

INS KIT 9606.ENSP00000380432 9606.ENSP00000288135 0 0 0 0 0 0 0.8 0.594 0.915

INS NCF1 9606.ENSP00000380432 9606.ENSP00000289473 0 0 0 0 0 0 0 0.469 0.469

INS VCAM1 9606.ENSP00000380432 9606.ENSP00000294728 0 0 0 0 0 0 0 0.654 0.654

INS NOS3 9606.ENSP00000380432 9606.ENSP00000297494 0 0 0 0 0 0 0 0.759 0.759

INS MMP3 9606.ENSP00000380432 9606.ENSP00000299855 0 0 0 0 0 0 0 0.504 0.504

INS INSR 9606.ENSP00000380432 9606.ENSP00000303830 0 0 0 0 0 0.972 0.9 0.989 0.999

INS PRKCB 9606.ENSP00000380432 9606.ENSP00000305355 0 0 0 0 0 0 0 0.467 0.467

INS ITPR1 9606.ENSP00000380432 9606.ENSP00000306253 0 0 0 0 0 0 0.65 0.394 0.778

INS LPL 9606.ENSP00000380432 9606.ENSP00000309757 0 0 0 0 0 0 0 0.818 0.818

INS PPARD 9606.ENSP00000380432 9606.ENSP00000310928 0 0 0 0 0 0 0 0.51 0.51

INS LEP 9606.ENSP00000380432 9606.ENSP00000312652 0 0 0 0 0 0 0 0.936 0.936

INS TPO 9606.ENSP00000380432 9606.ENSP00000318820 0 0 0 0 0.062 0 0 0.52 0.53

INS MMP1 9606.ENSP00000380432 9606.ENSP00000322788 0 0 0 0 0 0 0 0.427 0.427

INS NOS2 9606.ENSP00000380432 9606.ENSP00000327251 0 0 0 0 0 0 0 0.467 0.467

INS P4HB 9606.ENSP00000380432 9606.ENSP00000327801 0 0 0 0 0.062 0 0 0.571 0.58

INS SOCS1 9606.ENSP00000380432 9606.ENSP00000329418 0 0 0 0 0 0 0 0.455 0.455

INS SELE 9606.ENSP00000380432 9606.ENSP00000331736 0 0 0 0 0 0 0 0.601 0.601

INS SNCA 9606.ENSP00000380432 9606.ENSP00000338345 0 0 0 0 0 0 0.5 0.556 0.768

INS STAT5A 9606.ENSP00000380432 9606.ENSP00000341208 0 0 0 0 0 0 0 0.554 0.554

INS OCLN 9606.ENSP00000380432 9606.ENSP00000347379 0 0 0 0 0 0 0 0.489 0.489

INS SREBF1 9606.ENSP00000380432 9606.ENSP00000348069 0 0 0 0 0 0 0 0.844 0.844

INS NR3C2 9606.ENSP00000380432 9606.ENSP00000350815 0 0 0 0 0 0 0 0.51 0.51

INS STAT1 9606.ENSP00000380432 9606.ENSP00000354394 0 0 0 0 0 0 0 0.524 0.524

INS MTOR 9606.ENSP00000380432 9606.ENSP00000354558 0 0 0 0 0 0 0 0.867 0.867

INS PARP1 9606.ENSP00000380432 9606.ENSP00000355759 0 0 0 0 0 0 0 0.451 0.451

INS NGF 9606.ENSP00000380432 9606.ENSP00000358525 0 0 0 0 0 0 0 0.781 0.781

INS JUN 9606.ENSP00000380432 9606.ENSP00000360266 0 0 0 0 0 0 0 0.723 0.723

INS PTEN 9606.ENSP00000380432 9606.ENSP00000361021 0 0 0 0 0 0.27 0 0.719 0.786

INS MMP9 9606.ENSP00000380432 9606.ENSP00000361405 0 0 0 0 0 0 0 0.625 0.625

INS LCN2 9606.ENSP00000380432 9606.ENSP00000362108 0 0 0 0 0 0 0 0.582 0.582

INS TLR4 9606.ENSP00000380432 9606.ENSP00000363089 0 0 0 0 0 0 0 0.669 0.669

INS ITPR3 9606.ENSP00000380432 9606.ENSP00000363435 0 0 0 0 0 0 0.65 0.408 0.783

INS SHBG 9606.ENSP00000380432 9606.ENSP00000369816 0 0 0 0 0 0 0 0.87 0.87

INS TH 9606.ENSP00000380432 9606.ENSP00000370571 0 0 0 0 0 0 0 0.561 0.561

INS PC 9606.ENSP00000380432 9606.ENSP00000377532 0 0 0 0 0 0 0 0.61 0.61

INS PRKCD 9606.ENSP00000380432 9606.ENSP00000378217 0 0 0 0 0 0 0 0.574 0.574

INS SPP1 9606.ENSP00000380432 9606.ENSP00000378517 0 0 0 0 0 0 0 0.581 0.581

INS LTA 9606.ENSP00000380432 9606.ENSP00000403495 0 0 0 0 0 0 0 0.403 0.403

INS MYD88 9606.ENSP00000380432 9606.ENSP00000401399 0 0 0 0 0 0 0 0.459 0.459

INS NOS1 9606.ENSP00000380432 9606.ENSP00000477999 0 0 0 0 0 0 0 0.505 0.505

INS MTTP 9606.ENSP00000380432 9606.ENSP00000427679 0 0 0 0 0 0 0 0.594 0.594

INS VDR 9606.ENSP00000380432 9606.ENSP00000447173 0 0 0 0 0 0 0 0.602 0.602

INS VEGFA 9606.ENSP00000380432 9606.ENSP00000478570 0 0 0 0 0 0 0 0.762 0.762

INS TNF 9606.ENSP00000380432 9606.ENSP00000398698 0 0 0 0 0 0 0 0.879 0.879

INSR KITLG 9606.ENSP00000303830 9606.ENSP00000228280 0 0 0 0 0 0 0.8 0.181 0.829

INSR PPARG 9606.ENSP00000303830 9606.ENSP00000287820 0 0 0 0 0 0.077 0 0.507 0.525

INSR STAT5A 9606.ENSP00000303830 9606.ENSP00000341208 0 0 0 0 0 0.345 0 0.181 0.441

INSR VDR 9606.ENSP00000303830 9606.ENSP00000447173 0 0 0 0 0 0.077 0 0.432 0.453

INSR PRKCD 9606.ENSP00000303830 9606.ENSP00000378217 0 0 0 0.553 0.063 0.526 0 0.076 0.545

INSR SREBF1 9606.ENSP00000303830 9606.ENSP00000348069 0 0 0 0 0 0.062 0 0.539 0.55

INSR LEP 9606.ENSP00000303830 9606.ENSP00000312652 0 0 0 0 0 0 0 0.569 0.569

INSR MTOR 9606.ENSP00000303830 9606.ENSP00000354558 0 0 0 0 0.067 0.104 0 0.561 0.6

INSR NGF 9606.ENSP00000303830 9606.ENSP00000358525 0 0 0 0 0 0.056 0.6 0.14 0.647

INSR PLCB1 9606.ENSP00000303830 9606.ENSP00000338185 0 0 0 0 0.062 0.097 0.6 0.144 0.671

INSR VEGFA 9606.ENSP00000303830 9606.ENSP00000478570 0 0 0 0 0.062 0 0.6 0.245 0.691

INSR PTEN 9606.ENSP00000303830 9606.ENSP00000361021 0 0 0 0 0.054 0.364 0 0.638 0.763

INSR PRKCB 9606.ENSP00000303830 9606.ENSP00000305355 0 0 0 0.55 0.048 0.061 0.8 0.269 0.826

INSR SOCS1 9606.ENSP00000303830 9606.ENSP00000329418 0 0 0 0 0 0.642 0.8 0.144 0.933

IRF1 TNFRSF1A 9606.ENSP00000245414 9606.ENSP00000162749 0 0 0 0 0.063 0 0 0.39 0.403

IRF1 NFKBIA 9606.ENSP00000245414 9606.ENSP00000216797 0 0 0 0 0.276 0 0 0.52 0.637

IRF1 NFKB1 9606.ENSP00000245414 9606.ENSP00000226574 0 0 0 0 0.155 0.213 0 0.517 0.651

IRF1 NR3C1 9606.ENSP00000245414 9606.ENSP00000231509 0 0 0 0 0 0 0.9 0.321 0.929

IRF1 NOD2 9606.ENSP00000245414 9606.ENSP00000300589 0 0 0 0 0.135 0 0 0.336 0.401

IRF1 VCAM1 9606.ENSP00000245414 9606.ENSP00000294728 0 0 0 0 0.067 0 0 0.392 0.408

IRF1 NKX2-1 9606.ENSP00000245414 9606.ENSP00000346879 0 0 0 0 0 0 0 0.415 0.415

IRF1 USP7 9606.ENSP00000245414 9606.ENSP00000343535 0 0 0 0 0.063 0 0 0.456 0.468

IRF1 NOS2 9606.ENSP00000245414 9606.ENSP00000327251 0 0 0 0 0 0 0 0.503 0.503

IRF1 PPARG 9606.ENSP00000245414 9606.ENSP00000287820 0 0 0 0 0.062 0 0 0.536 0.546

IRF1 STAT5A 9606.ENSP00000245414 9606.ENSP00000341208 0 0 0 0 0.102 0 0 0.531 0.561

IRF1 TNFAIP3 9606.ENSP00000245414 9606.ENSP00000481570 0 0 0 0 0.327 0 0 0.45 0.614

IRF1 TLR4 9606.ENSP00000245414 9606.ENSP00000363089 0 0 0 0 0.085 0 0 0.604 0.623

IRF1 SOCS1 9606.ENSP00000245414 9606.ENSP00000329418 0 0 0 0 0.122 0 0 0.606 0.639

IRF1 TLR3 9606.ENSP00000245414 9606.ENSP00000296795 0 0 0 0 0.065 0 0 0.669 0.677

IRF1 TNF 9606.ENSP00000245414 9606.ENSP00000398698 0 0 0 0 0.175 0 0 0.675 0.721

IRF1 TP53 9606.ENSP00000245414 9606.ENSP00000269305 0 0 0 0 0.06 0 0 0.768 0.773

IRF1 MYD88 9606.ENSP00000245414 9606.ENSP00000401399 0 0 0 0 0.101 0.344 0 0.822 0.886

IRF1 MDM2 9606.ENSP00000245414 9606.ENSP00000258149 0 0 0 0 0 0.675 0.8 0.249 0.946

IRF1 JUN 9606.ENSP00000245414 9606.ENSP00000360266 0 0 0 0 0.062 0 0.9 0.907 0.99

IRF1 STAT1 9606.ENSP00000245414 9606.ENSP00000354394 0 0 0 0 0.381 0.878 0.9 0.983 0.999

ITGAL MPO 9606.ENSP00000349252 9606.ENSP00000225275 0 0 0 0 0.107 0 0 0.423 0.463

ITGAL SELP 9606.ENSP00000349252 9606.ENSP00000263686 0 0 0 0 0.108 0 0 0.635 0.66

ITGAL VCAM1 9606.ENSP00000349252 9606.ENSP00000294728 0 0 0 0 0.062 0 0 0.949 0.951

ITGAL SELE 9606.ENSP00000349252 9606.ENSP00000331736 0 0 0 0 0.049 0 0 0.631 0.634

ITGAL TLR4 9606.ENSP00000349252 9606.ENSP00000363089 0 0 0 0 0.069 0.059 0 0.39 0.418

ITGAL TNF 9606.ENSP00000349252 9606.ENSP00000398698 0 0 0 0 0.157 0 0 0.649 0.692

ITGAL ITGB2 9606.ENSP00000349252 9606.ENSP00000380948 0 0 0 0 0.418 0.87 0.9 0.989 0.999

ITGB2 MMP2 9606.ENSP00000380948 9606.ENSP00000219070 0 0 0 0 0 0.069 0.9 0.25 0.924

ITGB2 MPO 9606.ENSP00000380948 9606.ENSP00000225275 0 0 0 0 0.171 0.136 0 0.37 0.509

ITGB2 NFKB1 9606.ENSP00000380948 9606.ENSP00000226574 0 0 0 0 0.098 0.056 0.9 0.209 0.923

ITGB2 SELP 9606.ENSP00000380948 9606.ENSP00000263686 0 0 0 0 0.182 0 0.9 0.659 0.969

ITGB2 NCF1 9606.ENSP00000380948 9606.ENSP00000289473 0 0 0 0 0.325 0.161 0 0.288 0.561

ITGB2 VCAM1 9606.ENSP00000380948 9606.ENSP00000294728 0 0 0 0 0.45 0 0.9 0.935 0.996

ITGB2 NOD2 9606.ENSP00000380948 9606.ENSP00000300589 0 0 0 0 0.331 0 0 0.182 0.429

ITGB2 PRKCB 9606.ENSP00000380948 9606.ENSP00000305355 0 0 0 0 0.139 0.282 0 0.141 0.423

ITGB2 P4HB 9606.ENSP00000380948 9606.ENSP00000327801 0 0 0 0 0.073 0 0 0.471 0.489

ITGB2 SELE 9606.ENSP00000380948 9606.ENSP00000331736 0 0 0 0 0.075 0 0 0.651 0.663

ITGB2 SNCA 9606.ENSP00000380948 9606.ENSP00000338345 0 0 0 0 0 0 0 0.453 0.453

ITGB2 S100A8 9606.ENSP00000380948 9606.ENSP00000357722 0 0 0 0 0.347 0 0 0.411 0.599

ITGB2 S100A9 9606.ENSP00000380948 9606.ENSP00000357727 0 0 0 0 0.533 0 0 0.157 0.59

ITGB2 MMP9 9606.ENSP00000380948 9606.ENSP00000361405 0 0 0 0 0.29 0.069 0.9 0.399 0.955

ITGB2 TLR4 9606.ENSP00000380948 9606.ENSP00000363089 0 0 0 0 0.557 0.07 0 0.404 0.733

ITGB2 TNFRSF1B 9606.ENSP00000380948 9606.ENSP00000365435 0 0 0 0 0.287 0 0 0.241 0.436

ITGB2 THBD 9606.ENSP00000380948 9606.ENSP00000366307 0 0 0 0 0.098 0 0 0.376 0.413

ITGB2 PRKCD 9606.ENSP00000380948 9606.ENSP00000378217 0 0 0 0 0.131 0.283 0 0.146 0.422

ITGB2 TNF 9606.ENSP00000380948 9606.ENSP00000398698 0 0 0 0 0.222 0.057 0 0.558 0.648

ITPR1 NOS3 9606.ENSP00000306253 9606.ENSP00000297494 0 0 0 0 0 0 0 0.531 0.531

ITPR1 PRKCB 9606.ENSP00000306253 9606.ENSP00000305355 0 0 0 0 0.141 0 0.8 0.282 0.865

ITPR1 P4HB 9606.ENSP00000306253 9606.ENSP00000327801 0 0 0 0 0.062 0.078 0 0.366 0.403

ITPR1 PRKCD 9606.ENSP00000306253 9606.ENSP00000378217 0 0 0 0 0.065 0.27 0 0.222 0.422

ITPR1 OCLN 9606.ENSP00000306253 9606.ENSP00000347379 0 0 0 0 0 0 0 0.447 0.447

ITPR1 RYR1 9606.ENSP00000306253 9606.ENSP00000352608 0 0 0 0.543 0.064 0.166 0 0.794 0.478

ITPR1 VCP 9606.ENSP00000306253 9606.ENSP00000351777 0 0 0 0 0 0.519 0 0.167 0.582

ITPR1 KCNC3 9606.ENSP00000306253 9606.ENSP00000434241 0 0 0 0 0.087 0 0 0.57 0.591

ITPR1 PTEN 9606.ENSP00000306253 9606.ENSP00000361021 0 0 0 0 0.062 0 0 0.734 0.741

ITPR1 NOS1 9606.ENSP00000306253 9606.ENSP00000477999 0 0 0 0 0 0 0.8 0.244 0.842

ITPR1 ITPR3 9606.ENSP00000306253 9606.ENSP00000363435 0 0 0 0.965 0 0.27 0.8 0.871 0.852

ITPR1 PLCB1 9606.ENSP00000306253 9606.ENSP00000338185 0 0 0 0 0.062 0.127 0.65 0.596 0.868

ITPR1 STIM1 9606.ENSP00000306253 9606.ENSP00000478059 0 0 0 0 0 0.132 0.9 0.914 0.991

ITPR3 NOS3 9606.ENSP00000363435 9606.ENSP00000297494 0 0 0 0 0 0 0 0.402 0.402

ITPR3 PRKCB 9606.ENSP00000363435 9606.ENSP00000305355 0 0 0 0 0.097 0 0.8 0.204 0.843

ITPR3 P4HB 9606.ENSP00000363435 9606.ENSP00000327801 0 0 0 0 0.096 0.078 0 0.371 0.43

ITPR3 PLCB1 9606.ENSP00000363435 9606.ENSP00000338185 0 0 0 0 0.062 0.127 0.65 0.522 0.844

ITPR3 VCP 9606.ENSP00000363435 9606.ENSP00000351777 0 0 0 0 0 0.437 0 0.141 0.496

ITPR3 RYR1 9606.ENSP00000363435 9606.ENSP00000352608 0 0 0 0.543 0.064 0.166 0 0.772 0.469

ITPR3 MTOR 9606.ENSP00000363435 9606.ENSP00000354558 0 0 0 0 0.066 0.066 0 0.376 0.408

ITPR3 PTEN 9606.ENSP00000363435 9606.ENSP00000361021 0 0 0 0 0.062 0.27 0 0.516 0.64

ITPR3 NOS1 9606.ENSP00000363435 9606.ENSP00000477999 0 0 0 0 0 0 0.8 0.234 0.84

ITPR3 STIM1 9606.ENSP00000363435 9606.ENSP00000478059 0 0 0 0 0 0.132 0.9 0.943 0.994

JUN TNFRSF1A 9606.ENSP00000360266 9606.ENSP00000162749 0 0 0 0 0.062 0 0 0.618 0.626

JUN NFKBIA 9606.ENSP00000360266 9606.ENSP00000216797 0 0 0 0 0.125 0 0.9 0.805 0.981

JUN MMP2 9606.ENSP00000360266 9606.ENSP00000219070 0 0 0 0 0 0 0 0.655 0.655

JUN TGFB1 9606.ENSP00000360266 9606.ENSP00000221930 0 0 0 0 0.061 0 0.9 0.593 0.958

JUN MPO 9606.ENSP00000360266 9606.ENSP00000225275 0 0 0 0 0 0 0 0.458 0.458

JUN NFKB1 9606.ENSP00000360266 9606.ENSP00000226574 0 0 0 0 0.063 0 0 0.627 0.636

JUN NR3C1 9606.ENSP00000360266 9606.ENSP00000231509 0 0 0 0 0 0.493 0.9 0.973 0.998

JUN NOTCH2 9606.ENSP00000360266 9606.ENSP00000256646 0 0 0 0 0.052 0 0 0.401 0.408

JUN MDM2 9606.ENSP00000360266 9606.ENSP00000258149 0 0 0 0 0 0.063 0 0.657 0.666

JUN LYZ 9606.ENSP00000360266 9606.ENSP00000261267 0 0 0 0 0 0.129 0 0.43 0.482

JUN SMAD7 9606.ENSP00000360266 9606.ENSP00000262158 0 0 0 0 0.081 0.056 0 0.512 0.54

JUN LEF1 9606.ENSP00000360266 9606.ENSP00000265165 0 0 0 0 0 0 0 0.84 0.84

JUN TP53 9606.ENSP00000360266 9606.ENSP00000269305 0 0 0 0 0 0.149 0.6 0.838 0.94

JUN SOD1 9606.ENSP00000360266 9606.ENSP00000270142 0 0 0 0 0 0 0 0.439 0.439

JUN REN 9606.ENSP00000360266 9606.ENSP00000272190 0 0 0 0 0.062 0 0 0.458 0.469

JUN PPARG 9606.ENSP00000360266 9606.ENSP00000287820 0 0 0 0 0 0.066 0.9 0.842 0.984

JUN KIT 9606.ENSP00000360266 9606.ENSP00000288135 0 0 0 0 0 0.076 0 0.459 0.478

JUN NCF1 9606.ENSP00000360266 9606.ENSP00000289473 0 0 0 0 0 0 0 0.427 0.427

JUN VCAM1 9606.ENSP00000360266 9606.ENSP00000294728 0 0 0 0 0 0 0 0.565 0.565

JUN TLR3 9606.ENSP00000360266 9606.ENSP00000296795 0 0 0 0 0 0 0 0.63 0.631

JUN NOS3 9606.ENSP00000360266 9606.ENSP00000297494 0 0 0 0 0 0 0.9 0.603 0.958

JUN MMP3 9606.ENSP00000360266 9606.ENSP00000299855 0 0 0 0 0 0 0 0.57 0.57

JUN NOD2 9606.ENSP00000360266 9606.ENSP00000300589 0 0 0 0 0 0 0 0.526 0.526

JUN LPL 9606.ENSP00000360266 9606.ENSP00000309757 0 0 0 0 0.063 0 0.9 0.325 0.931

JUN PPARD 9606.ENSP00000360266 9606.ENSP00000310928 0 0 0 0 0 0.066 0 0.394 0.41

JUN LEP 9606.ENSP00000360266 9606.ENSP00000312652 0 0 0 0 0 0 0 0.533 0.533

JUN MMP1 9606.ENSP00000360266 9606.ENSP00000322788 0 0 0 0 0 0.628 0 0.621 0.853

JUN NOS2 9606.ENSP00000360266 9606.ENSP00000327251 0 0 0 0 0 0 0.9 0.581 0.956

JUN P4HB 9606.ENSP00000360266 9606.ENSP00000327801 0 0 0 0 0 0.129 0 0.34 0.4

JUN SOCS1 9606.ENSP00000360266 9606.ENSP00000329418 0 0 0 0 0.062 0 0 0.456 0.467

JUN SELE 9606.ENSP00000360266 9606.ENSP00000331736 0 0 0 0 0 0 0 0.463 0.463

JUN STAT5A 9606.ENSP00000360266 9606.ENSP00000341208 0 0 0 0 0 0.177 0 0.627 0.68

JUN RET 9606.ENSP00000360266 9606.ENSP00000347942 0 0 0 0 0 0.151 0 0.354 0.428

JUN SREBF1 9606.ENSP00000360266 9606.ENSP00000348069 0 0 0 0 0.062 0 0 0.557 0.567

JUN STAT1 9606.ENSP00000360266 9606.ENSP00000354394 0 0 0 0 0 0.343 0 0.764 0.839

JUN TOP1 9606.ENSP00000360266 9606.ENSP00000354522 0 0 0 0 0 0.284 0 0.633 0.726

JUN MTOR 9606.ENSP00000360266 9606.ENSP00000354558 0 0 0 0 0 0.128 0 0.68 0.709

JUN PARP1 9606.ENSP00000360266 9606.ENSP00000355759 0 0 0 0 0.064 0.299 0 0.521 0.658

JUN NGF 9606.ENSP00000360266 9606.ENSP00000358525 0 0 0 0 0 0 0 0.679 0.679

JUN NFIB 9606.ENSP00000360266 9606.ENSP00000370340 0 0 0 0 0.062 0 0 0.388 0.401

JUN TH 9606.ENSP00000360266 9606.ENSP00000370571 0 0 0 0 0 0 0 0.435 0.435

JUN TNFAIP3 9606.ENSP00000360266 9606.ENSP00000481570 0 0 0 0 0.099 0 0 0.403 0.439

JUN STIM1 9606.ENSP00000360266 9606.ENSP00000478059 0 0 0 0 0 0.387 0 0.181 0.477

JUN SPP1 9606.ENSP00000360266 9606.ENSP00000378517 0 0 0 0 0 0 0 0.521 0.521

JUN PRKCD 9606.ENSP00000360266 9606.ENSP00000378217 0 0 0 0 0 0 0 0.557 0.557

JUN LTA 9606.ENSP00000360266 9606.ENSP00000403495 0 0 0 0 0 0 0 0.578 0.578

JUN VDR 9606.ENSP00000360266 9606.ENSP00000447173 0 0 0 0 0 0.289 0 0.534 0.655

JUN TLR4 9606.ENSP00000360266 9606.ENSP00000363089 0 0 0 0 0 0 0 0.716 0.716

JUN MYD88 9606.ENSP00000360266 9606.ENSP00000401399 0 0 0 0 0 0 0 0.73 0.73

JUN PTEN 9606.ENSP00000360266 9606.ENSP00000361021 0 0 0 0 0 0.105 0 0.712 0.731

JUN MMP9 9606.ENSP00000360266 9606.ENSP00000361405 0 0 0 0 0 0.486 0 0.72 0.849

JUN VEGFA 9606.ENSP00000360266 9606.ENSP00000478570 0 0 0 0 0.066 0.182 0.9 0.683 0.972

JUN TNF 9606.ENSP00000360266 9606.ENSP00000398698 0 0 0 0 0 0 0.9 0.847 0.984

KCNC3 SCN1A 9606.ENSP00000434241 9606.ENSP00000303540 0 0 0 0 0.062 0.074 0 0.428 0.46

KCNC3 KCND2 9606.ENSP00000434241 9606.ENSP00000333496 0 0 0 0.757 0.097 0.097 0.5 0.664 0.626

KCNC3 SYT2 9606.ENSP00000434241 9606.ENSP00000356236 0 0 0 0 0.122 0 0 0.373 0.426

KCNC3 KCNJ10 9606.ENSP00000434241 9606.ENSP00000357068 0 0 0 0 0.15 0 0 0.442 0.505

KCND2 SCN1A 9606.ENSP00000333496 9606.ENSP00000303540 0 0 0 0 0.16 0.074 0 0.546 0.616

KCND2 SYT2 9606.ENSP00000333496 9606.ENSP00000356236 0 0 0 0 0.198 0 0 0.347 0.455

KCND2 KCNJ10 9606.ENSP00000333496 9606.ENSP00000357068 0 0 0 0 0.203 0 0 0.381 0.486

KCND2 SCN10A 9606.ENSP00000333496 9606.ENSP00000390600 0 0 0 0 0 0.074 0 0.477 0.495

KCND2 SCN9A 9606.ENSP00000333496 9606.ENSP00000386306 0 0 0 0 0.062 0.074 0 0.532 0.558

KCNJ10 SCN1A 9606.ENSP00000357068 9606.ENSP00000303540 0 0 0 0 0.153 0.102 0 0.391 0.496

KIT TGFB1 9606.ENSP00000288135 9606.ENSP00000221930 0 0 0 0 0.062 0.07 0 0.395 0.426

KIT MPO 9606.ENSP00000288135 9606.ENSP00000225275 0 0 0 0 0.065 0 0 0.681 0.688

KIT KITLG 9606.ENSP00000288135 9606.ENSP00000228280 0 0 0 0 0.062 0.977 0.9 0.99 0.999

KIT NOTCH2 9606.ENSP00000288135 9606.ENSP00000256646 0 0 0 0 0 0.056 0 0.421 0.43

KIT MDM2 9606.ENSP00000288135 9606.ENSP00000258149 0 0 0 0 0 0 0 0.556 0.556

KIT LEF1 9606.ENSP00000288135 9606.ENSP00000265165 0 0 0 0 0.053 0.073 0 0.381 0.409

KIT TP53 9606.ENSP00000288135 9606.ENSP00000269305 0 0 0 0 0 0.217 0 0.745 0.792

KIT SELE 9606.ENSP00000288135 9606.ENSP00000331736 0 0 0 0 0 0.076 0 0.387 0.409

KIT RET 9606.ENSP00000288135 9606.ENSP00000347942 0 0 0 0.66 0 0.213 0 0.803 0.425

KIT TLR4 9606.ENSP00000288135 9606.ENSP00000363089 0 0 0 0 0 0.09 0 0.396 0.427

KIT SPP1 9606.ENSP00000288135 9606.ENSP00000378517 0 0 0 0 0 0 0 0.455 0.455

KIT NOS3 9606.ENSP00000288135 9606.ENSP00000297494 0 0 0 0 0 0 0 0.459 0.459

KIT NKX2-1 9606.ENSP00000288135 9606.ENSP00000346879 0 0 0 0 0 0.065 0 0.455 0.468

KIT MMP9 9606.ENSP00000288135 9606.ENSP00000361405 0 0 0 0 0 0 0 0.546 0.546

KIT PAX5 9606.ENSP00000288135 9606.ENSP00000350844 0 0 0 0 0 0.056 0 0.556 0.562

KIT VCAM1 9606.ENSP00000288135 9606.ENSP00000294728 0 0 0 0 0.064 0 0 0.574 0.584

KIT MTOR 9606.ENSP00000288135 9606.ENSP00000354558 0 0 0 0 0 0.097 0 0.564 0.589

KIT PLCB1 9606.ENSP00000288135 9606.ENSP00000338185 0 0 0 0 0 0.082 0.6 0 0.617

KIT TNF 9606.ENSP00000288135 9606.ENSP00000398698 0 0 0 0 0 0 0 0.626 0.627

KIT PTEN 9606.ENSP00000288135 9606.ENSP00000361021 0 0 0 0 0 0.07 0 0.689 0.699

KIT NGF 9606.ENSP00000288135 9606.ENSP00000358525 0 0 0 0 0 0 0.6 0.419 0.757

KIT VEGFA 9606.ENSP00000288135 9606.ENSP00000478570 0 0 0 0 0 0.059 0.6 0.761 0.902

KIT STAT1 9606.ENSP00000288135 9606.ENSP00000354394 0 0 0 0 0 0.658 0.6 0.392 0.909

KIT STAT5A 9606.ENSP00000288135 9606.ENSP00000341208 0 0 0 0 0 0.27 0.8 0.621 0.939

KIT SOCS1 9606.ENSP00000288135 9606.ENSP00000329418 0 0 0 0 0 0.49 0.9 0.456 0.969

KITLG TNFRSF1A 9606.ENSP00000228280 9606.ENSP00000162749 0 0 0 0 0 0 0 0.404 0.404

KITLG TGFB1 9606.ENSP00000228280 9606.ENSP00000221930 0 0 0 0 0 0 0 0.424 0.424

KITLG PTEN 9606.ENSP00000228280 9606.ENSP00000361021 0 0 0 0 0 0 0 0.403 0.403

KITLG LEF1 9606.ENSP00000228280 9606.ENSP00000265165 0 0 0 0 0 0 0 0.451 0.451

KITLG SPP1 9606.ENSP00000228280 9606.ENSP00000378517 0 0 0 0 0 0 0 0.467 0.467

KITLG TP53 9606.ENSP00000228280 9606.ENSP00000269305 0 0 0 0 0 0 0 0.476 0.476

KITLG VCAM1 9606.ENSP00000228280 9606.ENSP00000294728 0 0 0 0 0 0 0 0.561 0.561

KITLG TNF 9606.ENSP00000228280 9606.ENSP00000398698 0 0 0 0 0 0 0 0.612 0.612

KITLG VEGFA 9606.ENSP00000228280 9606.ENSP00000478570 0 0 0 0 0 0 0 0.685 0.685

KITLG STAT1 9606.ENSP00000228280 9606.ENSP00000354394 0 0 0 0 0 0 0.6 0.302 0.708

KITLG NGF 9606.ENSP00000228280 9606.ENSP00000358525 0 0 0 0 0.076 0 0 0.708 0.719

KITLG STAT5A 9606.ENSP00000228280 9606.ENSP00000341208 0 0 0 0 0 0 0.6 0.557 0.815

KITLG SOCS1 9606.ENSP00000228280 9606.ENSP00000329418 0 0 0 0 0 0 0.9 0.165 0.912

KITLG MMP9 9606.ENSP00000228280 9606.ENSP00000361405 0 0 0 0 0 0 0.9 0.535 0.951

LCN2 MMP2 9606.ENSP00000362108 9606.ENSP00000219070 0 0 0 0 0 0.27 0.9 0.671 0.973

LCN2 TGFB1 9606.ENSP00000362108 9606.ENSP00000221930 0 0 0 0 0 0 0.9 0.358 0.933

LCN2 MPO 9606.ENSP00000362108 9606.ENSP00000225275 0 0 0 0 0.088 0 0 0.625 0.643

LCN2 LTF 9606.ENSP00000362108 9606.ENSP00000231751 0 0 0 0 0.187 0.154 0 0.556 0.667

LCN2 ORM1 9606.ENSP00000362108 9606.ENSP00000259396 0 0 0 0 0.107 0 0 0.423 0.463

LCN2 REN 9606.ENSP00000362108 9606.ENSP00000272190 0 0 0 0 0 0 0 0.558 0.558

LCN2 PPARG 9606.ENSP00000362108 9606.ENSP00000287820 0 0 0 0 0 0 0 0.414 0.414

LCN2 VCAM1 9606.ENSP00000362108 9606.ENSP00000294728 0 0 0 0 0.062 0 0 0.426 0.438

LCN2 MMP3 9606.ENSP00000362108 9606.ENSP00000299855 0 0 0 0 0.062 0 0.9 0.361 0.934

LCN2 LEP 9606.ENSP00000362108 9606.ENSP00000312652 0 0 0 0 0 0 0 0.506 0.506

LCN2 MMP1 9606.ENSP00000362108 9606.ENSP00000322788 0 0 0 0 0.052 0 0.9 0.508 0.949

LCN2 S100A8 9606.ENSP00000362108 9606.ENSP00000357722 0 0 0 0 0.205 0 0 0.345 0.457

LCN2 S100A9 9606.ENSP00000362108 9606.ENSP00000357727 0 0 0 0 0.227 0 0 0.37 0.492

LCN2 MMP9 9606.ENSP00000362108 9606.ENSP00000361405 0 0 0 0 0.096 0.67 0.9 0.989 0.999

LCN2 TLR4 9606.ENSP00000362108 9606.ENSP00000363089 0 0 0 0 0 0 0 0.566 0.566

LCN2 SPP1 9606.ENSP00000362108 9606.ENSP00000378517 0 0 0 0 0.062 0 0 0.606 0.614

LCN2 TNF 9606.ENSP00000362108 9606.ENSP00000398698 0 0 0 0 0.062 0 0 0.673 0.68

LCN2 VEGFA 9606.ENSP00000362108 9606.ENSP00000478570 0 0 0 0 0 0.101 0.9 0.469 0.948

LEF1 NFKB1 9606.ENSP00000265165 9606.ENSP00000226574 0 0 0 0 0.055 0.102 0 0.373 0.422

LEF1 SMAD7 9606.ENSP00000265165 9606.ENSP00000262158 0 0 0 0 0 0.555 0 0.335 0.691

LEF1 PTEN 9606.ENSP00000265165 9606.ENSP00000361021 0 0 0 0 0.062 0.129 0 0.417 0.482

LEF1 STAT5A 9606.ENSP00000265165 9606.ENSP00000341208 0 0 0 0 0.079 0.27 0 0.324 0.506

LEF1 TP53 9606.ENSP00000265165 9606.ENSP00000269305 0 0 0 0 0 0 0 0.523 0.523

LEF1 VDR 9606.ENSP00000265165 9606.ENSP00000447173 0 0 0 0 0 0.34 0 0.475 0.639

LEF1 PAX5 9606.ENSP00000265165 9606.ENSP00000350844 0 0 0 0 0 0.182 0.9 0.515 0.956

LEP TNFRSF1A 9606.ENSP00000312652 9606.ENSP00000162749 0 0 0 0 0 0 0 0.413 0.412

LEP MIF 9606.ENSP00000312652 9606.ENSP00000215754 0 0 0 0 0 0 0 0.408 0.408

LEP MMP2 9606.ENSP00000312652 9606.ENSP00000219070 0 0 0 0 0 0 0 0.46 0.459

LEP TGFB1 9606.ENSP00000312652 9606.ENSP00000221930 0 0 0 0 0 0 0 0.442 0.442

LEP PON1 9606.ENSP00000312652 9606.ENSP00000222381 0 0 0 0 0 0 0 0.568 0.568

LEP MPO 9606.ENSP00000312652 9606.ENSP00000225275 0 0 0 0 0.062 0 0 0.452 0.463

LEP NR3C1 9606.ENSP00000312652 9606.ENSP00000231509 0 0 0 0 0 0 0 0.558 0.558

LEP NR1D1 9606.ENSP00000312652 9606.ENSP00000246672 0 0 0 0 0 0 0 0.428 0.428

LEP TP53 9606.ENSP00000312652 9606.ENSP00000269305 0 0 0 0 0 0 0 0.467 0.467

LEP REN 9606.ENSP00000312652 9606.ENSP00000272190 0 0 0 0 0 0 0 0.787 0.787

LEP SST 9606.ENSP00000312652 9606.ENSP00000287641 0 0 0 0 0 0 0 0.624 0.624

LEP PPARG 9606.ENSP00000312652 9606.ENSP00000287820 0 0 0 0 0.062 0 0.9 0.852 0.984

LEP VCAM1 9606.ENSP00000312652 9606.ENSP00000294728 0 0 0 0 0 0 0 0.561 0.561

LEP NOS3 9606.ENSP00000312652 9606.ENSP00000297494 0 0 0 0 0.062 0 0 0.601 0.609

LEP MMP3 9606.ENSP00000312652 9606.ENSP00000299855 0 0 0 0 0.062 0 0 0.439 0.451

LEP LPL 9606.ENSP00000312652 9606.ENSP00000309757 0 0 0 0 0.062 0 0 0.757 0.762

LEP PPARD 9606.ENSP00000312652 9606.ENSP00000310928 0 0 0 0 0 0 0 0.452 0.452

LEP NR3C2 9606.ENSP00000312652 9606.ENSP00000350815 0 0 0 0 0 0 0 0.403 0.403

LEP NOS1 9606.ENSP00000312652 9606.ENSP00000477999 0 0 0 0 0.062 0 0 0.392 0.405

LEP VDR 9606.ENSP00000312652 9606.ENSP00000447173 0 0 0 0 0 0 0 0.455 0.455

LEP PTEN 9606.ENSP00000312652 9606.ENSP00000361021 0 0 0 0 0 0 0 0.463 0.463

LEP NGF 9606.ENSP00000312652 9606.ENSP00000358525 0 0 0 0 0 0 0 0.493 0.493

LEP SELE 9606.ENSP00000312652 9606.ENSP00000331736 0 0 0 0 0 0 0 0.517 0.517

LEP TH 9606.ENSP00000312652 9606.ENSP00000370571 0 0 0 0 0 0 0 0.52 0.52

LEP STAT1 9606.ENSP00000312652 9606.ENSP00000354394 0 0 0 0 0 0 0 0.529 0.529

LEP MMP9 9606.ENSP00000312652 9606.ENSP00000361405 0 0 0 0 0 0 0 0.556 0.556

LEP TLR4 9606.ENSP00000312652 9606.ENSP00000363089 0 0 0 0 0 0 0 0.616 0.616

LEP SPP1 9606.ENSP00000312652 9606.ENSP00000378517 0 0 0 0 0 0 0 0.656 0.656

LEP MTOR 9606.ENSP00000312652 9606.ENSP00000354558 0 0 0 0 0 0 0 0.684 0.685

LEP SHBG 9606.ENSP00000312652 9606.ENSP00000369816 0 0 0 0 0.064 0 0 0.747 0.753

LEP SREBF1 9606.ENSP00000312652 9606.ENSP00000348069 0 0 0 0 0 0 0 0.755 0.755

LEP STAT5A 9606.ENSP00000312652 9606.ENSP00000341208 0 0 0 0 0 0 0.6 0.502 0.792

LEP VEGFA 9606.ENSP00000312652 9606.ENSP00000478570 0 0 0 0 0 0 0 0.864 0.864

LEP TNF 9606.ENSP00000312652 9606.ENSP00000398698 0 0 0 0 0 0 0 0.871 0.871

LPL PON1 9606.ENSP00000309757 9606.ENSP00000222381 0 0 0 0 0 0 0.54 0.455 0.738

LPL NR3C1 9606.ENSP00000309757 9606.ENSP00000231509 0 0 0 0 0 0 0.9 0.341 0.931

LPL PPARG 9606.ENSP00000309757 9606.ENSP00000287820 0 0 0 0 0.062 0 0.9 0.851 0.984

LPL VCAM1 9606.ENSP00000309757 9606.ENSP00000294728 0 0 0 0 0.062 0 0 0.391 0.404

LPL NOS3 9606.ENSP00000309757 9606.ENSP00000297494 0 0 0 0 0 0 0 0.402 0.402

LPL TLR4 9606.ENSP00000309757 9606.ENSP00000363089 0 0 0 0 0.077 0 0 0.398 0.421

LPL PPARD 9606.ENSP00000309757 9606.ENSP00000310928 0 0 0 0 0 0 0 0.497 0.497

LPL SPP1 9606.ENSP00000309757 9606.ENSP00000378517 0 0 0 0 0.088 0 0 0.513 0.537

LPL TNF 9606.ENSP00000309757 9606.ENSP00000398698 0 0 0 0 0.062 0 0 0.577 0.587

LPL MTTP 9606.ENSP00000309757 9606.ENSP00000427679 0 0 0 0 0 0 0 0.666 0.666

LPL SREBF1 9606.ENSP00000309757 9606.ENSP00000348069 0 0 0 0 0 0 0 0.754 0.754

LTA TNFRSF1A 9606.ENSP00000403495 9606.ENSP00000162749 0 0 0 0 0.062 0.994 0.8 0.994 0.999

LTA MIF 9606.ENSP00000403495 9606.ENSP00000215754 0 0 0 0 0 0 0 0.429 0.429

LTA NFKBIA 9606.ENSP00000403495 9606.ENSP00000216797 0 0 0 0 0.085 0 0 0.414 0.442

LTA MMP2 9606.ENSP00000403495 9606.ENSP00000219070 0 0 0 0 0 0 0 0.492 0.492

LTA TGFB1 9606.ENSP00000403495 9606.ENSP00000221930 0 0 0 0 0.062 0 0 0.503 0.514

LTA MPO 9606.ENSP00000403495 9606.ENSP00000225275 0 0 0 0 0 0 0 0.539 0.539

LTA TP53 9606.ENSP00000403495 9606.ENSP00000269305 0 0 0 0 0 0 0 0.594 0.594

LTA VCAM1 9606.ENSP00000403495 9606.ENSP00000294728 0 0 0 0 0 0 0 0.736 0.736

LTA TLR3 9606.ENSP00000403495 9606.ENSP00000296795 0 0 0 0 0 0 0 0.671 0.671

LTA MMP3 9606.ENSP00000403495 9606.ENSP00000299855 0 0 0 0 0.062 0 0 0.405 0.418

LTA NOD2 9606.ENSP00000403495 9606.ENSP00000300589 0 0 0 0 0.102 0 0 0.464 0.498

LTA MMP1 9606.ENSP00000403495 9606.ENSP00000322788 0 0 0 0 0.062 0 0 0.405 0.418

LTA SELE 9606.ENSP00000403495 9606.ENSP00000331736 0 0 0 0 0 0 0 0.427 0.427

LTA STAT1 9606.ENSP00000403495 9606.ENSP00000354394 0 0 0 0 0.059 0 0 0.478 0.487

LTA NGF 9606.ENSP00000403495 9606.ENSP00000358525 0 0 0 0 0 0 0 0.47 0.47

LTA MMP9 9606.ENSP00000403495 9606.ENSP00000361405 0 0 0 0 0.087 0 0 0.71 0.724

LTA TLR4 9606.ENSP00000403495 9606.ENSP00000363089 0 0 0 0 0.056 0 0 0.557 0.564

LTA TNFRSF1B 9606.ENSP00000403495 9606.ENSP00000365435 0 0 0 0 0.111 0.852 0.9 0.811 0.997

LTA TNF 9606.ENSP00000403495 9606.ENSP00000398698 0 0 0 0.759 0.282 0.27 0.8 0.83 0.908

LTA MYD88 9606.ENSP00000403495 9606.ENSP00000401399 0 0 0 0 0.062 0 0 0.742 0.748

LTA VEGFA 9606.ENSP00000403495 9606.ENSP00000478570 0 0 0 0 0 0 0 0.632 0.632

LTF MPO 9606.ENSP00000231751 9606.ENSP00000225275 0 0 0 0 0.119 0 0 0.574 0.609

LTF ORM1 9606.ENSP00000231751 9606.ENSP00000259396 0 0 0 0 0.142 0 0 0.337 0.407

LTF MMP9 9606.ENSP00000231751 9606.ENSP00000361405 0 0 0 0 0.153 0 0 0.337 0.415

LTF S100A8 9606.ENSP00000231751 9606.ENSP00000357722 0 0 0 0 0.212 0.08 0 0.421 0.544

LTF S100A9 9606.ENSP00000231751 9606.ENSP00000357727 0 0 0 0 0.202 0.08 0 0.446 0.558

LTF LYZ 9606.ENSP00000231751 9606.ENSP00000261267 0 0 0 0 0.127 0.27 0.5 0.652 0.874

LYZ MPO 9606.ENSP00000261267 9606.ENSP00000225275 0 0 0 0 0.138 0 0 0.474 0.527

LYZ TLR4 9606.ENSP00000261267 9606.ENSP00000363089 0 0 0 0 0.14 0 0 0.369 0.435

LYZ TNF 9606.ENSP00000261267 9606.ENSP00000398698 0 0 0 0 0.107 0 0 0.433 0.473

LYZ SNCA 9606.ENSP00000261267 9606.ENSP00000338345 0 0 0 0 0 0 0.5 0.064 0.511

LYZ S100A8 9606.ENSP00000261267 9606.ENSP00000357722 0 0 0 0 0.265 0 0 0.371 0.518

LYZ S100A9 9606.ENSP00000261267 9606.ENSP00000357727 0 0 0 0 0.303 0 0 0.427 0.583

MDM2 NFKBIA 9606.ENSP00000258149 9606.ENSP00000216797 0 0 0 0 0 0 0 0.459 0.459

MDM2 NR3C1 9606.ENSP00000258149 9606.ENSP00000231509 0 0 0 0 0.063 0.334 0 0.31 0.532

MDM2 VCP 9606.ENSP00000258149 9606.ENSP00000351777 0 0 0 0 0.062 0.298 0 0.167 0.403

MDM2 MMP9 9606.ENSP00000258149 9606.ENSP00000361405 0 0 0 0 0 0 0 0.403 0.403

MDM2 PPARD 9606.ENSP00000258149 9606.ENSP00000310928 0 0 0 0 0 0.427 0 0.092 0.457

MDM2 PARP1 9606.ENSP00000258149 9606.ENSP00000355759 0 0 0 0 0 0 0 0.46 0.46

MDM2 TNF 9606.ENSP00000258149 9606.ENSP00000398698 0 0 0 0 0 0 0 0.468 0.468

MDM2 PCNA 9606.ENSP00000258149 9606.ENSP00000368458 0 0 0 0 0 0.486 0 0.107 0.521

MDM2 PPARG 9606.ENSP00000258149 9606.ENSP00000287820 0 0 0 0 0 0.282 0 0.367 0.526

MDM2 MTOR 9606.ENSP00000258149 9606.ENSP00000354558 0 0 0 0 0 0 0 0.564 0.564

MDM2 TOP1 9606.ENSP00000258149 9606.ENSP00000354522 0 0 0 0 0 0.27 0 0.457 0.586

MDM2 VEGFA 9606.ENSP00000258149 9606.ENSP00000478570 0 0 0 0 0 0.27 0 0.561 0.665

MDM2 PTEN 9606.ENSP00000258149 9606.ENSP00000361021 0 0 0 0 0.062 0 0.9 0.775 0.977

MDM2 USP7 9606.ENSP00000258149 9606.ENSP00000343535 0 0 0 0 0.062 0.92 0.9 0.989 0.999

MDM2 TP53 9606.ENSP00000258149 9606.ENSP00000269305 0 0 0 0 0.24 0.998 0.9 0.995 0.999

MIF SPP1 9606.ENSP00000215754 9606.ENSP00000378517 0 0 0 0 0.062 0 0 0.391 0.404

MIF VCAM1 9606.ENSP00000215754 9606.ENSP00000294728 0 0 0 0 0 0.064 0 0.416 0.43

MIF SOD1 9606.ENSP00000215754 9606.ENSP00000270142 0 0 0 0 0.351 0.043 0 0.242 0.488

MIF MMP9 9606.ENSP00000215754 9606.ENSP00000361405 0 0 0 0 0 0 0 0.503 0.503

MIF TLR4 9606.ENSP00000215754 9606.ENSP00000363089 0 0 0 0 0 0 0 0.523 0.523

MIF VEGFA 9606.ENSP00000215754 9606.ENSP00000478570 0 0 0 0 0 0 0 0.595 0.595

MIF TNF 9606.ENSP00000215754 9606.ENSP00000398698 0 0 0 0 0 0 0 0.684 0.684

MIF TP53 9606.ENSP00000215754 9606.ENSP00000269305 0 0 0 0 0.062 0.494 0 0.504 0.744

MMP1 TNFRSF1A 9606.ENSP00000322788 9606.ENSP00000162749 0 0 0 0 0.061 0 0 0.391 0.403

MMP1 NFKBIA 9606.ENSP00000322788 9606.ENSP00000216797 0 0 0 0 0.062 0 0 0.459 0.47

MMP1 MMP2 9606.ENSP00000322788 9606.ENSP00000219070 0 0 0 0.869 0.154 0 0.9 0.883 0.921

MMP1 TGFB1 9606.ENSP00000322788 9606.ENSP00000221930 0 0 0 0 0.076 0 0.9 0.609 0.96

MMP1 MPO 9606.ENSP00000322788 9606.ENSP00000225275 0 0 0 0 0 0 0 0.405 0.405

MMP1 TP53 9606.ENSP00000322788 9606.ENSP00000269305 0 0 0 0 0 0 0 0.524 0.524

MMP1 VCAM1 9606.ENSP00000322788 9606.ENSP00000294728 0 0 0 0 0.065 0 0 0.526 0.537

MMP1 MMP3 9606.ENSP00000322788 9606.ENSP00000299855 0 0 0.298 0.953 0.529 0 0.9 0.912 0.953

MMP1 TLR4 9606.ENSP00000322788 9606.ENSP00000363089 0 0 0 0 0 0 0 0.428 0.428

MMP1 SELE 9606.ENSP00000322788 9606.ENSP00000331736 0 0 0 0 0.052 0 0 0.46 0.466

MMP1 SPP1 9606.ENSP00000322788 9606.ENSP00000378517 0 0 0 0 0.049 0 0 0.517 0.521

MMP1 TNF 9606.ENSP00000322788 9606.ENSP00000398698 0 0 0 0 0.064 0 0 0.7 0.707

MMP1 MMP9 9606.ENSP00000322788 9606.ENSP00000361405 0 0 0 0.75 0.518 0 0.9 0.901 0.961

MMP1 VEGFA 9606.ENSP00000322788 9606.ENSP00000478570 0 0 0 0 0.069 0 0.9 0.683 0.967

MMP2 TNFRSF1A 9606.ENSP00000219070 9606.ENSP00000162749 0 0 0 0 0.108 0 0 0.376 0.42

MMP2 NFKBIA 9606.ENSP00000219070 9606.ENSP00000216797 0 0 0 0 0 0 0 0.517 0.517

MMP2 NGF 9606.ENSP00000219070 9606.ENSP00000358525 0 0 0 0 0.098 0 0 0.396 0.431

MMP2 SMAD7 9606.ENSP00000219070 9606.ENSP00000262158 0 0 0 0 0.051 0.057 0 0.435 0.45

MMP2 PARP1 9606.ENSP00000219070 9606.ENSP00000355759 0 0 0 0 0 0 0 0.451 0.451

MMP2 SELP 9606.ENSP00000219070 9606.ENSP00000263686 0 0 0 0 0 0 0 0.46 0.459

MMP2 MYD88 9606.ENSP00000219070 9606.ENSP00000401399 0 0 0 0 0 0 0 0.462 0.462

MMP2 MMP3 9606.ENSP00000219070 9606.ENSP00000299855 0 0 0 0.88 0.18 0 0.3 0.878 0.464

MMP2 MTOR 9606.ENSP00000219070 9606.ENSP00000354558 0 0 0 0 0 0 0 0.469 0.469

MMP2 REN 9606.ENSP00000219070 9606.ENSP00000272190 0 0 0 0 0.052 0 0 0.486 0.492

MMP2 SELE 9606.ENSP00000219070 9606.ENSP00000331736 0 0 0 0 0 0 0 0.504 0.504

MMP2 MPO 9606.ENSP00000219070 9606.ENSP00000225275 0 0 0 0 0 0 0 0.505 0.505

MMP2 TLR4 9606.ENSP00000219070 9606.ENSP00000363089 0 0 0 0 0.062 0 0 0.501 0.511

MMP2 PPARG 9606.ENSP00000219070 9606.ENSP00000287820 0 0 0 0 0.062 0 0 0.504 0.514

MMP2 OCLN 9606.ENSP00000219070 9606.ENSP00000347379 0 0 0 0 0 0 0 0.559 0.559

MMP2 PTEN 9606.ENSP00000219070 9606.ENSP00000361021 0 0 0 0 0 0 0 0.566 0.566

MMP2 NOS3 9606.ENSP00000219070 9606.ENSP00000297494 0 0 0 0 0 0 0 0.602 0.602

MMP2 VCAM1 9606.ENSP00000219070 9606.ENSP00000294728 0 0 0 0 0.109 0 0 0.602 0.63

MMP2 TP53 9606.ENSP00000219070 9606.ENSP00000269305 0 0 0 0 0.062 0 0 0.669 0.676

MMP2 SPP1 9606.ENSP00000219070 9606.ENSP00000378517 0 0 0 0 0.076 0 0 0.691 0.702

MMP2 TNF 9606.ENSP00000219070 9606.ENSP00000398698 0 0 0 0 0 0 0 0.734 0.734

MMP2 NCF1 9606.ENSP00000219070 9606.ENSP00000289473 0 0 0 0 0 0 0.65 0.319 0.751

MMP2 MMP9 9606.ENSP00000219070 9606.ENSP00000361405 0 0 0 0.929 0.062 0 0.9 0.923 0.908

MMP2 VEGFA 9606.ENSP00000219070 9606.ENSP00000478570 0 0 0 0 0.065 0 0.9 0.848 0.984

MMP2 TGFB1 9606.ENSP00000219070 9606.ENSP00000221930 0 0 0 0 0.062 0.634 0.9 0.693 0.988

MMP3 TNFRSF1A 9606.ENSP00000299855 9606.ENSP00000162749 0 0 0 0 0.062 0 0 0.419 0.431

MMP3 NFKBIA 9606.ENSP00000299855 9606.ENSP00000216797 0 0 0 0 0.062 0 0 0.525 0.535

MMP3 TGFB1 9606.ENSP00000299855 9606.ENSP00000221930 0 0 0 0 0 0 0.9 0.544 0.952

MMP3 MPO 9606.ENSP00000299855 9606.ENSP00000225275 0 0 0 0 0 0 0 0.455 0.455

MMP3 TP53 9606.ENSP00000299855 9606.ENSP00000269305 0 0 0 0 0 0 0 0.523 0.523

MMP3 REN 9606.ENSP00000299855 9606.ENSP00000272190 0 0 0 0 0.052 0 0 0.396 0.403

MMP3 VCAM1 9606.ENSP00000299855 9606.ENSP00000294728 0 0 0 0 0.062 0 0 0.543 0.553

MMP3 OCLN 9606.ENSP00000299855 9606.ENSP00000347379 0 0 0 0 0 0 0 0.456 0.456

MMP3 NGF 9606.ENSP00000299855 9606.ENSP00000358525 0 0 0 0 0.088 0 0 0.453 0.48

MMP3 SELE 9606.ENSP00000299855 9606.ENSP00000331736 0 0 0 0 0 0 0 0.481 0.481

MMP3 TLR4 9606.ENSP00000299855 9606.ENSP00000363089 0 0 0 0 0.062 0 0 0.497 0.508

MMP3 TNF 9606.ENSP00000299855 9606.ENSP00000398698 0 0 0 0 0.064 0 0 0.762 0.767

MMP3 MMP9 9606.ENSP00000299855 9606.ENSP00000361405 0 0 0 0.791 0.518 0 0.8 0.903 0.918

MMP3 VEGFA 9606.ENSP00000299855 9606.ENSP00000478570 0 0 0 0 0 0 0.9 0.671 0.965

MMP3 SPP1 9606.ENSP00000299855 9606.ENSP00000378517 0 0 0 0 0.062 0.213 0.9 0.811 0.984

MMP9 TNFRSF1A 9606.ENSP00000361405 9606.ENSP00000162749 0 0 0 0 0.07 0 0 0.519 0.533

MMP9 NFKBIA 9606.ENSP00000361405 9606.ENSP00000216797 0 0 0 0 0.092 0 0 0.683 0.7

MMP9 TGFB1 9606.ENSP00000361405 9606.ENSP00000221930 0 0 0 0 0.085 0.634 0.9 0.762 0.991

MMP9 MPO 9606.ENSP00000361405 9606.ENSP00000225275 0 0 0 0 0.076 0 0 0.716 0.727

MMP9 NFKB1 9606.ENSP00000361405 9606.ENSP00000226574 0 0 0 0 0.085 0 0 0.412 0.439

MMP9 SMAD7 9606.ENSP00000361405 9606.ENSP00000262158 0 0 0 0 0 0.057 0 0.501 0.509

MMP9 SELP 9606.ENSP00000361405 9606.ENSP00000263686 0 0 0 0 0.07 0 0 0.566 0.579

MMP9 TP53 9606.ENSP00000361405 9606.ENSP00000269305 0 0 0 0 0 0 0 0.704 0.704

MMP9 REN 9606.ENSP00000361405 9606.ENSP00000272190 0 0 0 0 0.052 0 0 0.54 0.545

MMP9 PPARG 9606.ENSP00000361405 9606.ENSP00000287820 0 0 0 0 0 0 0 0.652 0.652

MMP9 NCF1 9606.ENSP00000361405 9606.ENSP00000289473 0 0 0 0 0.152 0 0.65 0.435 0.818

MMP9 VCAM1 9606.ENSP00000361405 9606.ENSP00000294728 0 0 0 0 0.062 0 0 0.722 0.728

MMP9 PF4 9606.ENSP00000361405 9606.ENSP00000296029 0 0 0 0 0.096 0 0 0.463 0.494

MMP9 NOS3 9606.ENSP00000361405 9606.ENSP00000297494 0 0 0 0 0.062 0 0 0.658 0.665

MMP9 NOS2 9606.ENSP00000361405 9606.ENSP00000327251 0 0 0 0 0.065 0 0 0.535 0.547

MMP9 SELE 9606.ENSP00000361405 9606.ENSP00000331736 0 0 0 0 0 0 0 0.634 0.634

MMP9 OCLN 9606.ENSP00000361405 9606.ENSP00000347379 0 0 0 0 0 0 0 0.694 0.694

MMP9 STAT1 9606.ENSP00000361405 9606.ENSP00000354394 0 0 0 0 0 0 0 0.537 0.537

MMP9 MTOR 9606.ENSP00000361405 9606.ENSP00000354558 0 0 0 0 0 0 0 0.528 0.528

MMP9 S100A8 9606.ENSP00000361405 9606.ENSP00000357722 0 0 0 0 0.23 0 0 0.341 0.471

MMP9 S100A9 9606.ENSP00000361405 9606.ENSP00000357727 0 0 0 0 0.314 0 0 0.34 0.528

MMP9 NGF 9606.ENSP00000361405 9606.ENSP00000358525 0 0 0 0 0 0 0 0.525 0.525

MMP9 PTEN 9606.ENSP00000361405 9606.ENSP00000361021 0 0 0 0 0 0 0 0.62 0.62

MMP9 PRKCD 9606.ENSP00000361405 9606.ENSP00000378217 0 0 0 0 0.077 0 0 0.392 0.414

MMP9 THBD 9606.ENSP00000361405 9606.ENSP00000366307 0 0 0 0 0.133 0 0 0.376 0.436

MMP9 TNFRSF1B 9606.ENSP00000361405 9606.ENSP00000365435 0 0 0 0 0.098 0 0 0.413 0.447

MMP9 MYD88 9606.ENSP00000361405 9606.ENSP00000401399 0 0 0 0 0.096 0 0 0.518 0.546

MMP9 SPP1 9606.ENSP00000361405 9606.ENSP00000378517 0 0 0 0 0.11 0 0 0.694 0.717

MMP9 TLR4 9606.ENSP00000361405 9606.ENSP00000363089 0 0 0 0 0.116 0 0 0.833 0.847

MMP9 TNF 9606.ENSP00000361405 9606.ENSP00000398698 0 0 0 0 0.176 0 0 0.872 0.89

MMP9 VEGFA 9606.ENSP00000361405 9606.ENSP00000478570 0 0 0 0 0 0 0.9 0.879 0.987

MPO TNFRSF1A 9606.ENSP00000225275 9606.ENSP00000162749 0 0 0 0 0 0 0 0.413 0.412

MPO NFKBIA 9606.ENSP00000225275 9606.ENSP00000216797 0 0 0 0 0 0 0 0.568 0.568

MPO PON1 9606.ENSP00000225275 9606.ENSP00000222381 0 0 0 0 0 0 0 0.923 0.923

MPO REN 9606.ENSP00000225275 9606.ENSP00000272190 0 0 0 0 0.089 0 0 0.38 0.411

MPO TP53 9606.ENSP00000225275 9606.ENSP00000269305 0 0 0 0 0 0 0 0.424 0.424

MPO TNFRSF1B 9606.ENSP00000225275 9606.ENSP00000365435 0 0 0 0 0.096 0 0 0.403 0.437

MPO PF4 9606.ENSP00000225275 9606.ENSP00000296029 0 0 0 0 0.098 0 0 0.413 0.447

MPO XDH 9606.ENSP00000225275 9606.ENSP00000368727 0 0 0 0 0 0 0 0.448 0.448

MPO PPARG 9606.ENSP00000225275 9606.ENSP00000287820 0 0 0 0 0.062 0.056 0 0.467 0.486

MPO PAX5 9606.ENSP00000225275 9606.ENSP00000350844 0 0 0 0 0.062 0 0 0.477 0.488

MPO OCLN 9606.ENSP00000225275 9606.ENSP00000347379 0 0 0 0 0 0 0 0.494 0.494

MPO S100A9 9606.ENSP00000225275 9606.ENSP00000357727 0 0 0 0 0.231 0 0 0.394 0.514

MPO S100A8 9606.ENSP00000225275 9606.ENSP00000357722 0 0 0 0 0.232 0.128 0 0.341 0.52

MPO MYD88 9606.ENSP00000225275 9606.ENSP00000401399 0 0 0 0 0 0 0 0.529 0.529

MPO NOS2 9606.ENSP00000225275 9606.ENSP00000327251 0 0 0 0 0 0.09 0 0.535 0.559

MPO VEGFA 9606.ENSP00000225275 9606.ENSP00000478570 0 0 0 0 0.062 0 0 0.562 0.571

MPO NOS3 9606.ENSP00000225275 9606.ENSP00000297494 0 0 0 0 0 0.09 0 0.565 0.587

MPO NCF1 9606.ENSP00000225275 9606.ENSP00000289473 0 0 0 0 0.096 0 0 0.593 0.616

MPO VCAM1 9606.ENSP00000225275 9606.ENSP00000294728 0 0 0 0 0 0 0 0.633 0.633

MPO SELE 9606.ENSP00000225275 9606.ENSP00000331736 0 0 0 0 0 0.056 0 0.636 0.641

MPO SELP 9606.ENSP00000225275 9606.ENSP00000263686 0 0 0 0 0.096 0.056 0 0.674 0.698

MPO TLR4 9606.ENSP00000225275 9606.ENSP00000363089 0 0 0 0 0.088 0 0 0.702 0.716

MPO TNF 9606.ENSP00000225275 9606.ENSP00000398698 0 0 0 0 0.074 0 0 0.861 0.866

MTOR NFKBIA 9606.ENSP00000354558 9606.ENSP00000216797 0 0 0 0 0.054 0.359 0 0.467 0.649

MTOR TGFB1 9606.ENSP00000354558 9606.ENSP00000221930 0 0 0 0 0 0 0 0.408 0.408

MTOR NFKB1 9606.ENSP00000354558 9606.ENSP00000226574 0 0 0 0 0.054 0.159 0.9 0.233 0.93
[truncated: 40,681 more chars]
