# Supplementary material for: Decoding the Mechanism of CheReCunJin Formula in Treating Sjögren's Syndrome Based on Network Pharmacology and Molecular Docking
Source: Evid Based Complement Alternat Med. 2022 Sep 20;2022:1193846. doi: 10.1155/2022/1193846 (PMC9553462; doi:10.1155/2022/1193846)
Supplement: Supplementary Materials — Table S1: the active ingredients of CRCJ. Table S2: the nodes and edges of the network. Table S3: summary table of drug targets. Table S4: summary table of disease targets. Table S5: network cluster results. [file 1193846.f1.zip › 1193846.f1/Supplementary Table 3.docx]

Supplementary Table 3

ABAT CRCJF

ABCA2 CRCJF

ABCC1 CRCJF

ABCC2 CRCJF

ABCC4  CRCJF

ABCG1 CRCJF

ACACA  CRCJF

ACHE CRCJF

ACP3 CRCJF

ADCY2 CRCJF

ADH1C  CRCJF

ADIPOQ CRCJF

ADRA1A CRCJF

ADRA1B CRCJF

ADRA1D CRCJF

ADRA2A CRCJF

ADRB1 CRCJF

ADRB2  CRCJF

AHR CRCJF

AHSA1 CRCJF

AKR1B1 CRCJF

AKR1C1 CRCJF

AKR1C3 CRCJF

AKT1 CRCJF

ALDH2 CRCJF

ALDH3A1  CRCJF

ALOX5 CRCJF

APOB CRCJF

APOD CRCJF

APP CRCJF

AR CRCJF

ATP5F1B CRCJF

BACE CRCJF

BAD CRCJF

BATF3 CRCJF

BAX CRCJF

BBC3 CRCJF

BCL2 CRCJF

BCL2L1 CRCJF

BIRC4 CRCJF

BIRC5  CRCJF

C1R CRCJF

CA2 CRCJF

CACNA2D1 CRCJF

CALM CRCJF

CASP3 CRCJF

CASP7 CRCJF

CASP8 CRCJF

CASP9 CRCJF

CAT CRCJF

CAV1 CRCJF

CCL2 CRCJF

CCNA2  CRCJF

CCNB1 CRCJF

CCND1 CRCJF

CD36 CRCJF

CD40LG CRCJF

CDK1 CRCJF

CDK2 CRCJF

CDK4 CRCJF

CDK7  CRCJF

CDKN1A CRCJF

CDKN2A CRCJF

CES1 CRCJF

CETP CRCJF

CHEK1 CRCJF

CHEK2 CRCJF

CHRM1 CRCJF

CHRM2 CRCJF

CHRM3 CRCJF

CHRM4 CRCJF

CHRM5 CRCJF

CHRNA7  CRCJF

CHUK CRCJF

CLDN4 CRCJF

COL19A1 CRCJF

COL3A1 CRCJF

CRP CRCJF

CTNNB1 CRCJF

CTRB1 CRCJF

CTSD CRCJF

CXCL10 CRCJF

CXCL11 CRCJF

CXCL2  CRCJF

CXCL8 CRCJF

CYCS CRCJF

CYP19A1 CRCJF

CYP1A1 CRCJF

CYP1A2 CRCJF

CYP1B1 CRCJF

Cyp2b1 CRCJF

CYP2C9 CRCJF

CYP3A4 CRCJF

DCAF5 CRCJF

DIO1 CRCJF

DPEP1 CRCJF

DPP4 CRCJF

DRD5 CRCJF

DUOX2 CRCJF

E2F1 CRCJF

E2F2 CRCJF

EDN1 CRCJF

EGF CRCJF

EGFR CRCJF

EGLN1 CRCJF

EIF6 CRCJF

ELK1 CRCJF

ERBB2 CRCJF

ERBB3 CRCJF

ESR1  CRCJF

ESR2  CRCJF

F10 CRCJF

F3 CRCJF

F7 CRCJF

FABP5 CRCJF

FASLG CRCJF

FASN CRCJF

FOS CRCJF

FOSL1 CRCJF

FOSL2 CRCJF

G6PD CRCJF

GABRA1 CRCJF

GABRA2 CRCJF

GABRA3 CRCJF

GABRA5 CRCJF

GJA1  CRCJF

GLB1 CRCJF

GOT1 CRCJF

GRIA2  CRCJF

GSK3B CRCJF

GSR CRCJF

GSTM1 CRCJF

GSTM2 CRCJF

GSTP1 CRCJF

GYRB CRCJF

HAS2 CRCJF

HEL-S-89n CRCJF

HIF1AN CRCJF

HK2 CRCJF

HMGCR CRCJF

HMOX1 CRCJF

HSD3B1 CRCJF

HSD3B2 CRCJF

HSF1 CRCJF

HSP90AB1 CRCJF

HSPB1 CRCJF

HTR CRCJF

HTR2A CRCJF

ICAM1 CRCJF

IFNG CRCJF

IGF2  CRCJF

IGFBP3 CRCJF

Ighg1 CRCJF

IKBKB CRCJF

IL10 CRCJF

IL1A CRCJF

IL1B CRCJF

IL2 CRCJF

IL4 CRCJF

IL6 CRCJF

INSR CRCJF

IRF1 CRCJF

JUN CRCJF

JVH1 CRCJF

KCNH2 CRCJF

KCNJ10 CRCJF

KCNK2  CRCJF

KCNMA1 CRCJF

KDR CRCJF

LDLR CRCJF

LOX12  CRCJF

LTA4H CRCJF

MAOA CRCJF

MAOB CRCJF

MAP2 CRCJF

MAPK14 CRCJF

MAPK3 CRCJF

MAPK8  CRCJF

MCL1 CRCJF

MDM2 CRCJF

MDR1 CRCJF

MET CRCJF

MGAM  CRCJF

MMP1 CRCJF

MMP10  CRCJF

MMP2  CRCJF

MMP3 CRCJF

MMP9 CRCJF

MPO CRCJF

MT-ND6 CRCJF

MTOR CRCJF

MTTP CRCJF

MYC CRCJF

N1 CRCJF

NCF1 CRCJF

NCOA1 CRCJF

NCOA2 CRCJF

NFATC1 CRCJF

NFE2L2 CRCJF

NFKBIA CRCJF

NKX3-1 CRCJF

NOS2 CRCJF

NOS3 CRCJF

NOX5 CRCJF

NPEPPS CRCJF

NQO1 CRCJF

NR1I2 CRCJF

NR1I2  CRCJF

NR1I3 CRCJF

NR3C1 CRCJF

NR3C2 CRCJF

NUF2  CRCJF

ODC1 CRCJF

OL1A2 CRCJF

OLR1 CRCJF

OPRD1 CRCJF

OPRM1 CRCJF

PARP1 CRCJF

PCNA CRCJF

PCOLCE CRCJF

PDE10A CRCJF

PDE3A CRCJF

PECAM1 CRCJF

PGR CRCJF

PIK3CG CRCJF

PIM1 CRCJF

PIP4K2A CRCJF

PKIA  CRCJF

PLA2G4A CRCJF

PLAT CRCJF

PLAU CRCJF

PLB1 CRCJF

PON1 CRCJF

POR CRCJF

PPARA CRCJF

PPARD CRCJF

PPARG CRCJF

PPP3CA CRCJF

PRKACA CRCJF

PRKCA CRCJF

PRKCB  CRCJF

PRKCD CRCJF

PRSS1 CRCJF

PRXC1A CRCJF

PSMD3 CRCJF

PTEN  CRCJF

PTGER3 CRCJF

PTGES CRCJF

PTGES2 CRCJF

PTGS1 CRCJF

PTGS2 CRCJF

PTPN1 CRCJF

PYGM CRCJF

RAF1 CRCJF

RASA1 CRCJF

RASSF1 CRCJF

RB1 CRCJF

RELA  CRCJF

RUNX1T1 CRCJF

RUNX2  CRCJF

RXRA CRCJF

RXRB  CRCJF

RXRG  CRCJF

SCN5A CRCJF

SELE CRCJF

SELP CRCJF

SERPINE1 CRCJF

SIRT1 CRCJF

SLC2A4  CRCJF

SLC6A2  CRCJF

SLC6A3 CRCJF

SLC6A4 CRCJF

SLPI CRCJF

SOAT1 CRCJF

SOAT2 CRCJF

SOD1 CRCJF

SPP1 CRCJF

SREBF1 CRCJF

STAT1 CRCJF

Ste CRCJF

TDRD7  CRCJF

TEP1 CRCJF

TGFB1 CRCJF

THBD CRCJF

TNF CRCJF

TNFRSF1A CRCJF

TNFRSF1B CRCJF

TOP1 CRCJF

TOP2A CRCJF

TP53 CRCJF

TRPV1 CRCJF

TYR CRCJF

UCP2 CRCJF

UGT1A1 CRCJF

VCAM1 CRCJF

VEGFA CRCJF

XDH CRCJF

44263 CRCJF

AARS CRCJF

AARS2 CRCJF

ABCA1 CRCJF

ABCB1 CRCJF

ABCB4 CRCJF

ABCC4 CRCJF

ABCF1 CRCJF

ABCG2 CRCJF

ABHD5 CRCJF

ABHD6 CRCJF

ACAD8 CRCJF

ACADM CRCJF

ACADS CRCJF

ACADSB CRCJF

ACAT1 CRCJF

ACE CRCJF

ACE2 CRCJF

ACIN1 CRCJF

ACMSD CRCJF

ACO2 CRCJF

ACOT4 CRCJF

ACOX1 CRCJF

ACP5 CRCJF

ACPP CRCJF

ACSL1 CRCJF

ACSL3 CRCJF

ACSL4 CRCJF

ACSS1 CRCJF

ACSS2 CRCJF

ACTN2 CRCJF

ACTN3 CRCJF

ACVR2A CRCJF

ACY1 CRCJF

ACY3 CRCJF

ADA CRCJF

ADAM17 CRCJF

ADAM8 CRCJF

ADAMTS20 CRCJF

ADAMTS9 CRCJF

ADAP1 CRCJF

ADAP2 CRCJF

ADCY1 CRCJF

ADCY3 CRCJF

ADCY6 CRCJF

ADH1A CRCJF

ADH1B CRCJF

ADH1C CRCJF

ADH4 CRCJF

ADH7 CRCJF

ADIRF CRCJF

ADK CRCJF

ADORA1 CRCJF

ADORA2A CRCJF

ADORA2B CRCJF

ADORA3 CRCJF

ADRA2B CRCJF

ADRA2C CRCJF

ADRB2 CRCJF

ADRB3 CRCJF

ADRBK1 CRCJF

ADSL CRCJF

ADSS CRCJF

ADSSL1 CRCJF

AFM CRCJF

AGER CRCJF

AGMAT CRCJF

AGRN CRCJF

AGT CRCJF

AGTR1 CRCJF

AGTR2 CRCJF

AGTRAP CRCJF

AGXT CRCJF

AGXT2 CRCJF

AHCY CRCJF

AHCYL1 CRCJF

AIF1 CRCJF

AIFM1 CRCJF

AK5 CRCJF

AK9 CRCJF

AKR1C2 CRCJF

AKR1D1 CRCJF

ALAD CRCJF

ALAS1 CRCJF

ALAS2 CRCJF

ALB CRCJF

ALDH1A1 CRCJF

ALDH1A2 CRCJF

ALDH1A3 CRCJF

ALDH1B1 CRCJF

ALDH3A1 CRCJF

ALDH3B1 CRCJF

ALDH3B2 CRCJF

ALDH5A1 CRCJF

ALDH7A1 CRCJF

ALDH8A1 CRCJF

ALDH9A1 CRCJF

ALDOA CRCJF

ALDOB CRCJF

ALDOC CRCJF

ALOX15 CRCJF

ALOX15B CRCJF

ALOX5AP CRCJF

AMD1 CRCJF

AMELX CRCJF

AMICA1 CRCJF

AMN CRCJF

AMPD3 CRCJF

ANAPC2 CRCJF

ANG CRCJF

ANGPT1 CRCJF

ANK2 CRCJF

ANK3 CRCJF

ANKH CRCJF

ANO9 CRCJF

ANXA1 CRCJF

ANXA13 CRCJF

AP3D1 CRCJF

APLN CRCJF

APLP1 CRCJF

APOA1 CRCJF

APOA2 CRCJF

APOC2 CRCJF

APOE CRCJF

APOH CRCJF

APRT CRCJF

AQP1 CRCJF

AQP2 CRCJF

AQP8 CRCJF

ARCN1 CRCJF

AREG CRCJF

ARFGEF2 CRCJF

ARG1 CRCJF

ARG2 CRCJF

ARHGEF2 CRCJF

ARID1A CRCJF

ARPIN CRCJF

ARRB1 CRCJF

ARRB2 CRCJF

ARRDC3 CRCJF

ARV1 CRCJF

ARX CRCJF

ASCL1 CRCJF

ASL CRCJF

ASNS CRCJF

ASPA CRCJF

ASPDH CRCJF

ASPH CRCJF

ASRGL1 CRCJF

ASS1 CRCJF

ATIC CRCJF

ATM CRCJF

ATOH1 CRCJF

ATP11C CRCJF

ATP13A2 CRCJF

ATP1A1 CRCJF

ATP1A2 CRCJF

ATP2A1 CRCJF

ATP2B4 CRCJF

ATP4A CRCJF

ATP8B1 CRCJF

AURKA CRCJF

AVP CRCJF

AVPR1A CRCJF

AVPR1B CRCJF

AVPR2 CRCJF

AZIN1 CRCJF

AZIN2 CRCJF

B4GALT1 CRCJF

BAAT CRCJF

BAK1 CRCJF

BBOX1 CRCJF

BCAT1 CRCJF

BCAT2 CRCJF

BCHE CRCJF

BCKDHA CRCJF

BCKDHB CRCJF

BCKDK CRCJF

BCL11B CRCJF

BCL2L11 CRCJF

BDH1 CRCJF

BDKRB2 CRCJF

BDNF CRCJF

BGLAP CRCJF

BICD1 CRCJF

BIN3 CRCJF

BLVRA CRCJF

BLVRB CRCJF

BMP2 CRCJF

BMP4 CRCJF

BMP5 CRCJF

BMP6 CRCJF

BNIP3 CRCJF

BRCA1 CRCJF

C1QTNF1 CRCJF

C1QTNF3 CRCJF

C2CD5 CRCJF

C2orf83 CRCJF

C3 CRCJF

C5 CRCJF

CA7 CRCJF

CACNA1A CRCJF

CACNA1B CRCJF

CACNA1C CRCJF

CACNA1D CRCJF

CACNA1F CRCJF

CACNA1G CRCJF

CACNA1H CRCJF

CACNA1I CRCJF

CACNA1S CRCJF

CACNA2D2 CRCJF

CACNB2 CRCJF

CACNG1 CRCJF

CAD CRCJF

CALB1 CRCJF

CALCA CRCJF

CALHM1 CRCJF

CALY CRCJF

CAMK2D CRCJF

CAMK2G CRCJF

CAPN3 CRCJF

CARTPT CRCJF

CASQ1 CRCJF

CASQ2 CRCJF

CAV3 CRCJF

CBFA2T3 CRCJF

CBR1 CRCJF

CBR3 CRCJF

CBR4 CRCJF

CBS CRCJF

CBSL CRCJF

CCL3 CRCJF

CCL5 CRCJF

CCM2L CRCJF

CCR7 CRCJF

CCS CRCJF

CD28 CRCJF

CD300A CRCJF

CD34 CRCJF

CD40 CRCJF

CD47 CRCJF

CD63 CRCJF

CD74 CRCJF

CDC20 CRCJF

CDC42 CRCJF

CDH11 CRCJF

CDH3 CRCJF

CDH5 CRCJF

CDH8 CRCJF

CDIPT CRCJF

CDK5R1 CRCJF

CDK5R2 CRCJF

CEND1 CRCJF

CERS1 CRCJF

CERS2 CRCJF

CETN1 CRCJF

CETN2 CRCJF

CFTR CRCJF

CHAT CRCJF

CHDH CRCJF

CHGA CRCJF

CHKA CRCJF

CHRFAM7A CRCJF

CHRNA10 CRCJF

CHRNA2 CRCJF

CHRNA3 CRCJF

CHRNA4 CRCJF

CHRNA5 CRCJF

CHRNA6 CRCJF

CHRNA7 CRCJF

CHRNA9 CRCJF

CHRNB2 CRCJF

CHRNB3 CRCJF

CHRNB4 CRCJF

CKB CRCJF

CKM CRCJF

CKMT1A CRCJF

CKMT1B CRCJF

CKMT2 CRCJF

CLDN5 CRCJF

CLN3 CRCJF

CMPK1 CRCJF

CMPK2 CRCJF

CNR1 CRCJF

CNR2 CRCJF

CNST CRCJF

CNTF CRCJF

CNTNAP4 CRCJF

COL1A1 CRCJF

COL27A1 CRCJF

COLGALT1 CRCJF

COLGALT2 CRCJF

COLQ CRCJF

COMT CRCJF

COX1 CRCJF

COX2 CRCJF

COX3 CRCJF

COX4I1 CRCJF

COX5A CRCJF

COX5B CRCJF

COX6A2 CRCJF

COX6B1 CRCJF

COX6C CRCJF

COX7A1 CRCJF

COX7B CRCJF

COX7C CRCJF

COX8A CRCJF

CPLX2 CRCJF

CPS1 CRCJF

CPT1A CRCJF

CPT2 CRCJF

CRACR2A CRCJF

CRAT CRCJF

CREB1 CRCJF

CRH CRCJF

CRHBP CRCJF

CRHR2 CRCJF

CRLF1 CRCJF

CROT CRCJF

CRTAP CRCJF

CRYM CRCJF

CRYZ CRCJF

CRYZL1 CRCJF

CSF1 CRCJF

CSF2 CRCJF

CTCF CRCJF

CTNNBIP1 CRCJF

CTPS1 CRCJF

CTR9 CRCJF

CTSG CRCJF

CTSH CRCJF

CUBN CRCJF

CX3CR1 CRCJF

CXCL13 CRCJF

CXCR4 CRCJF

CYB5R1 CRCJF

CYB5R3 CRCJF

CYBA CRCJF

CYBB CRCJF

CYGB CRCJF

CYP11A1 CRCJF

CYP17A1 CRCJF

CYP24A1 CRCJF

CYP27A1 CRCJF

CYP27B1 CRCJF

CYP2E1 CRCJF

CYP2R1 CRCJF

CYP39A1 CRCJF

CYP4B1 CRCJF

CYP4F11 CRCJF

CYP4F2 CRCJF

CYP51A1 CRCJF

CYR61 CRCJF

DAB2 CRCJF

DAB2IP CRCJF

DAGLA CRCJF

DAO CRCJF

DARS CRCJF

DARS2 CRCJF

DBH CRCJF

DBT CRCJF

DCK CRCJF

DCSTAMP CRCJF

DCT CRCJF

DDC CRCJF

DGAT2 CRCJF

DGKA CRCJF

DGKI CRCJF

DGUOK CRCJF

DHFR CRCJF

DHFRL1 CRCJF

DHODH CRCJF

DHRS2 CRCJF

DHRS3 CRCJF

DHRS4 CRCJF

DHRS9 CRCJF

DHTKD1 CRCJF

DKK3 CRCJF

DLD CRCJF

DLG4 CRCJF

DLL1 CRCJF

DLST CRCJF

DLX5 CRCJF

DMGDH CRCJF

DMTN CRCJF

DNAJA3 CRCJF

DNAJB9 CRCJF

DNAJC3 CRCJF

DNM3 CRCJF

DNMT1 CRCJF

DNMT3A CRCJF

DNMT3B CRCJF

DOCK4 CRCJF

DOCK5 CRCJF

DPPA3 CRCJF

DPYD CRCJF

DPYS CRCJF

DRD1 CRCJF

DRD2 CRCJF

DRD3 CRCJF

DRD4 CRCJF

DTYMK CRCJF

DUT CRCJF

ECE1 CRCJF

EDA CRCJF

EDN2 CRCJF

EDN3 CRCJF

EDNRA CRCJF

EGLN2 CRCJF

EGLN3 CRCJF

EGR1 CRCJF

EIF2AK1 CRCJF

ELANE CRCJF

ELOVL1 CRCJF

ELOVL3 CRCJF

ELOVL4 CRCJF

ELOVL6 CRCJF

ELOVL7 CRCJF

ENPP1 CRCJF

ENPP3 CRCJF

ENPP6 CRCJF

EOMES CRCJF

EPCAM CRCJF

EPHA4 CRCJF

EPHB1 CRCJF

EPHX2 CRCJF

EPO CRCJF

EPRS CRCJF

ERO1B CRCJF

ESR1 CRCJF

ESR2 CRCJF

ESRRG CRCJF

ETFDH CRCJF

ETHE1 CRCJF

F11 CRCJF

F12 CRCJF

F2 CRCJF

F2RL1 CRCJF

F9 CRCJF

FABP2 CRCJF

FABP3 CRCJF

FABP6 CRCJF

FADD CRCJF

FADS1 CRCJF

FADS2 CRCJF

FAS CRCJF

FBLN1 CRCJF

FBP1 CRCJF

FBXO45 CRCJF

FCER1A CRCJF

FCER1G CRCJF

FCER2 CRCJF

FDXR CRCJF

FECH CRCJF

FER CRCJF

FFAR1 CRCJF

FGA CRCJF

FGF1 CRCJF

FGF10 CRCJF

FGF2 CRCJF

FGF23 CRCJF

FGF4 CRCJF

FGFR2 CRCJF

FKBP1A CRCJF

FKBP1B CRCJF

FLAD1 CRCJF

FLOT1 CRCJF

FMR1 CRCJF

FNDC5 CRCJF

FNTA CRCJF

FOLR1 CRCJF

FOLR2 CRCJF

FOLR3 CRCJF

FOXA1 CRCJF

FOXP3 CRCJF

FSCN1 CRCJF

FURIN CRCJF

FUT7 CRCJF

FXYD1 CRCJF

GABBR1 CRCJF

GABRA4 CRCJF

GABRA6 CRCJF

GABRB1 CRCJF

GABRB2 CRCJF

GABRB3 CRCJF

GABRD CRCJF

GABRE CRCJF

GABRG1 CRCJF

GABRG2 CRCJF

GABRG3 CRCJF

GABRP CRCJF

GABRQ CRCJF

GAL3ST1 CRCJF

GALC CRCJF

GAMT CRCJF

GARS CRCJF

GAS6 CRCJF

GATA3 CRCJF

GATM CRCJF

GBA CRCJF

GC CRCJF

GCAT CRCJF

GCDH CRCJF

GCSH CRCJF

GDF5 CRCJF

GFER CRCJF

GFI1 CRCJF

GGCX CRCJF

GHRH CRCJF

GHRL CRCJF

GJA5 CRCJF

GJD4 CRCJF

GLDC CRCJF

GLI3 CRCJF

GLRA1 CRCJF

GLRA2 CRCJF

GLRA3 CRCJF

GLRB CRCJF

GLUL CRCJF

GLYAT CRCJF

GLYATL1 CRCJF

GLYATL2 CRCJF

GLYR1 CRCJF

GNA15 CRCJF

GNAS CRCJF

GNAT1 CRCJF

GNAT2 CRCJF

GNAT3 CRCJF

GNB2L1 CRCJF

GNMT CRCJF

GNPAT CRCJF

GNRH1 CRCJF

GOT2 CRCJF

GPBAR1 CRCJF

GPD1L CRCJF

GPER1 CRCJF

GPLD1 CRCJF

GPR143 CRCJF

GPR18 CRCJF

GPR27 CRCJF

GPR55 CRCJF

GPRC5A CRCJF

GPT CRCJF

GPT2 CRCJF

GPX7 CRCJF

GRIA1 CRCJF

GRIA2 CRCJF

GRIA3 CRCJF

GRIA4 CRCJF

GRIK1 CRCJF

GRIK2 CRCJF

GRIK3 CRCJF

GRIK4 CRCJF

GRIK5 CRCJF

GRIN1 CRCJF

GRIN2A CRCJF

GRIN2B CRCJF

GRIN2C CRCJF

GRIN2D CRCJF

GRIN3A CRCJF

GRIN3B CRCJF

GRM7 CRCJF

GSK3A CRCJF

GSS CRCJF

GUCY1B3 CRCJF

GUK1 CRCJF

HAAO CRCJF

HACD1 CRCJF

HACL1 CRCJF

HAP1 CRCJF

HBA1 CRCJF

HBA2 CRCJF

HCAR2 CRCJF

HCAR3 CRCJF

HCN2 CRCJF

HCN4 CRCJF

HCRT CRCJF

HDAC1 CRCJF

HDAC2 CRCJF

HDAC9 CRCJF

HEG1 CRCJF

HELB CRCJF

HELLS CRCJF

HIBADH CRCJF

HIF1A CRCJF

HINT1 CRCJF

HIPK2 CRCJF

HMBS CRCJF

HMGA2 CRCJF

HNF1B CRCJF

HOMER1 CRCJF

HOPX CRCJF

HOXA5 CRCJF

HPD CRCJF

HPN CRCJF

HPRT1 CRCJF

HPS4 CRCJF

HPX CRCJF

HRC CRCJF

HRH1 CRCJF

HRH2 CRCJF

HRH3 CRCJF

HRH4 CRCJF

HSD11B1 CRCJF

HSD17B1 CRCJF

HSD17B11 CRCJF

HSD17B2 CRCJF

HSD17B6 CRCJF

HSD17B7 CRCJF

HSD17B8 CRCJF

HSH2D CRCJF

HSP90AA1 CRCJF

HSPA5 CRCJF

HTN1 CRCJF

HTR1A CRCJF

HTR1B CRCJF

HTR1D CRCJF

HTR1E CRCJF

HTR1F CRCJF

HTR2B CRCJF

HTR2C CRCJF

HTR3A CRCJF

HTR3B CRCJF

HTR3C CRCJF

HTR3D CRCJF

HTR3E CRCJF

HTR4 CRCJF

HTR5A CRCJF

HTR6 CRCJF

HTR7 CRCJF

HTT CRCJF

HYAL2 CRCJF

IARS CRCJF

IARS2 CRCJF

IDNK CRCJF

IDO1 CRCJF

IFI6 CRCJF

IGF1 CRCJF

IGF2 CRCJF

IHH CRCJF

IL13 CRCJF

IL17A CRCJF

IL17RA CRCJF

IL18R1 CRCJF

IL1RN CRCJF

IL34 CRCJF

IL4I1 CRCJF

ILVBL CRCJF

IMPA1 CRCJF

IMPA2 CRCJF

IMPDH1 CRCJF

IMPDH2 CRCJF

INS CRCJF

IRX5 CRCJF

ITFG2 CRCJF

ITGAL CRCJF

ITGAV CRCJF

ITGB2 CRCJF

ITGB3 CRCJF

ITPR1 CRCJF

ITPR2 CRCJF

ITPR3 CRCJF

IVD CRCJF

IYD CRCJF

JAK3 CRCJF

JMJD6 CRCJF

JUNB CRCJF

JUP CRCJF

KANK2 CRCJF

KARS CRCJF

KCNA1 CRCJF

KCNA10 CRCJF

KCNA2 CRCJF

KCNA3 CRCJF

KCNA4 CRCJF

KCNA5 CRCJF

KCNA6 CRCJF

KCNA7 CRCJF

KCNB1 CRCJF

KCNB2 CRCJF

KCNC1 CRCJF

KCNC2 CRCJF

KCNC3 CRCJF

KCND1 CRCJF

KCND2 CRCJF

KCND3 CRCJF

KCNE2 CRCJF

KCNE5 CRCJF

KCNIP2 CRCJF

KCNJ1 CRCJF

KCNJ11 CRCJF

KCNJ12 CRCJF

KCNJ14 CRCJF

KCNJ15 CRCJF

KCNJ8 CRCJF

KCNK4 CRCJF

KCNQ1 CRCJF

KDM1A CRCJF

KDM3A CRCJF

KDM6B CRCJF

KHDRBS1 CRCJF

KIF14 CRCJF

KISS1 CRCJF

KIT CRCJF

KITLG CRCJF

KL CRCJF

KLF4 CRCJF

KLF5 CRCJF

KLKB1 CRCJF

KMO CRCJF

KMT2A CRCJF

KYNU CRCJF

L3HYPDH CRCJF

LAMP2 CRCJF

LANCL2 CRCJF

LARP4B CRCJF

LARS CRCJF

LARS2 CRCJF

LCMT1 CRCJF

LCMT2 CRCJF

LCN2 CRCJF

LEF1 CRCJF

LEP CRCJF

LGALS3 CRCJF

LGALS9 CRCJF

LHCGR CRCJF

LIAS CRCJF

LIG4 CRCJF

LILRB1 CRCJF

LIPT1 CRCJF

LONP1 CRCJF

LPCAT1 CRCJF

LPCAT2 CRCJF

LPCAT4 CRCJF

LPL CRCJF

LRAT CRCJF

LRRC4B CRCJF

LRRC8A CRCJF

LRRK2 CRCJF

LTA CRCJF

LTF CRCJF

LYZ CRCJF

MAD2L2 CRCJF

MAGI2 CRCJF

MAP2K1 CRCJF

MAP2K5 CRCJF

MAP4K4 CRCJF

MAPK9 CRCJF

MAS1 CRCJF

MAT1A CRCJF

MAT2A CRCJF

MATN1 CRCJF

MC1R CRCJF

MC2R CRCJF

MC3R CRCJF

MC4R CRCJF

MC5R CRCJF

MCM3 CRCJF

MECOM CRCJF

MED1 CRCJF

MEF2C CRCJF

METRNL CRCJF

MEX3C CRCJF

MGLL CRCJF

MGMT CRCJF

MGP CRCJF

MIF CRCJF

MIP CRCJF

MLNR CRCJF

MMAA CRCJF

MMAB CRCJF

MMACHC CRCJF

MMP2 CRCJF

MMP28 CRCJF

MRPS36 CRCJF

MT3 CRCJF

MTAP CRCJF

MTNR1A CRCJF

MTNR1B CRCJF

MTR CRCJF

MTRR CRCJF

MUT CRCJF

MYD88 CRCJF

MYF5 CRCJF

MYF6 CRCJF

MYL2 CRCJF

MYO5A CRCJF

MYOD1 CRCJF

MYOG CRCJF

NAGS CRCJF

NAMPT CRCJF

NAPEPLD CRCJF

NAPRT CRCJF

NARS CRCJF

NARS2 CRCJF

NAV2 CRCJF

NCBP2 CRCJF

NCF2 CRCJF

NCF4 CRCJF

NCK1 CRCJF

NCK2 CRCJF

NCKAP1L CRCJF

NCOA3 CRCJF

NCOR1 CRCJF

NDOR1 CRCJF

NDRG2 CRCJF

NEDD4 CRCJF

NEDD4L CRCJF

NEFH CRCJF

NEFL CRCJF

NEUROD2 CRCJF

NF2 CRCJF

NFIB CRCJF

NFKB1 CRCJF

NFKB2 CRCJF

NFS1 CRCJF

NFX1 CRCJF

NGF CRCJF

NGFR CRCJF

NKX2-1 CRCJF

NLGN1 CRCJF

NLGN3 CRCJF

NMUR2 CRCJF

NNMT CRCJF

NOD2 CRCJF

NODAL CRCJF

NOS1 CRCJF

NOS1AP CRCJF

NOTCH1 CRCJF

NOTCH2 CRCJF

NOX1 CRCJF

NPPA CRCJF

NPPC CRCJF

NPY2R CRCJF

NPY5R CRCJF

NQO2 CRCJF

NR0B1 CRCJF

NR1D1 CRCJF

NR1H2 CRCJF

NR1H3 CRCJF

NR1H4 CRCJF

NRG1 CRCJF

NRP1 CRCJF

NRXN1 CRCJF

NRXN2 CRCJF

NRXN3 CRCJF

NT5C1A CRCJF

NT5C2 CRCJF

NT5E CRCJF

NT5M CRCJF

NTSR1 CRCJF

NUDT12 CRCJF

NUDT9 CRCJF

NUFIP2 CRCJF

OAT CRCJF

OAZ1 CRCJF

OAZ2 CRCJF

OAZ3 CRCJF

OCA2 CRCJF

OCLN CRCJF

OGDH CRCJF

OGDHL CRCJF

OGFOD1 CRCJF

OPN3 CRCJF

OPN4 CRCJF

OPN5 CRCJF

OPRK1 CRCJF

ORM1 CRCJF

OSBPL8 CRCJF

OTC CRCJF

OXCT1 CRCJF

OXCT2 CRCJF

OXER1 CRCJF

OXT CRCJF

OXTR CRCJF

P2RX1 CRCJF

P2RX2 CRCJF

P2RX3 CRCJF

P2RY1 CRCJF

P3H1 CRCJF

P3H2 CRCJF

P3H3 CRCJF

P4HA1 CRCJF

P4HA2 CRCJF

P4HA3 CRCJF

P4HB CRCJF

PAH CRCJF

PAICS CRCJF

PAM CRCJF

PAOX CRCJF

PARK2 CRCJF

PARP10 CRCJF

PARS2 CRCJF

PAWR CRCJF

PAX2 CRCJF

PAX5 CRCJF

PAX7 CRCJF

PAXBP1 CRCJF

PC CRCJF

PCCB CRCJF

PCK1 CRCJF

PCSK6 CRCJF

PCSK9 CRCJF

PCYT1A CRCJF

PCYT1B CRCJF

PDE11A CRCJF

PDE1A CRCJF

PDE1B CRCJF

PDE1C CRCJF

PDE2A CRCJF

PDE3B CRCJF

PDE4A CRCJF

PDE4B CRCJF

PDE4C CRCJF

PDE4D CRCJF

PDE5A CRCJF

PDE6A CRCJF

PDE6B CRCJF

PDE6C CRCJF

PDE7A CRCJF

PDE7B CRCJF

PDE8A CRCJF

PDE8B CRCJF

PDE9A CRCJF

PDGFA CRCJF

PDGFB CRCJF

PDHB CRCJF

PDXP CRCJF

PF4 CRCJF

PFKL CRCJF

PGD CRCJF

PGLS CRCJF

PGRMC1 CRCJF

PHB CRCJF

PHGDH CRCJF

PHKG2 CRCJF

PHOSPHO1 CRCJF

PHOX2B CRCJF

PHYKPL CRCJF

PIK3CA CRCJF

PIK3CB CRCJF

PIK3CD CRCJF

PIK3R1 CRCJF

PIK3R5 CRCJF

PIK3R6 CRCJF

PINK1 CRCJF

PIPOX CRCJF

PKD2 CRCJF

PKLR CRCJF

PKM CRCJF

PKP2 CRCJF

PKP3 CRCJF

PLA2G1B CRCJF

PLA2G2D CRCJF

PLA2G2E CRCJF

PLA2R1 CRCJF

PLCB1 CRCJF

PLCG2 CRCJF

PLD1 CRCJF

PLD2 CRCJF

PLD3 CRCJF

PLD4 CRCJF

PLG CRCJF

PLIN5 CRCJF

PLN CRCJF

PLOD1 CRCJF

PLOD2 CRCJF

PLOD3 CRCJF

PML CRCJF

PNLIPRP2 CRCJF

PNP CRCJF

PNPLA2 CRCJF

PODXL CRCJF

POLA1 CRCJF

POLA2 CRCJF

POLB CRCJF

POLD1 CRCJF

POLE CRCJF

POLE2 CRCJF

POLE3 CRCJF

POLE4 CRCJF

POLG CRCJF

POU1F1 CRCJF

POU4F3 CRCJF

PPARGC1B CRCJF

PPAT CRCJF

PPIA CRCJF

PPIB CRCJF

PPIC CRCJF

PPIF CRCJF

PPIG CRCJF

PPIH CRCJF

PPP1R15A CRCJF

PPP1R1B CRCJF

PPP1R9B CRCJF

PPP2CA CRCJF

PPP2CB CRCJF

PPP2R1A CRCJF

PPP3CB CRCJF

PPP3R1 CRCJF

PQLC2 CRCJF

PRDM16 CRCJF

PRDM8 CRCJF

PRKAA1 CRCJF

PRKAA2 CRCJF

PRKAB1 CRCJF

PRKAB2 CRCJF

PRKAG1 CRCJF

PRKAG2 CRCJF

PRKAG3 CRCJF

PRKCB CRCJF

PRKCG CRCJF

PRKDC CRCJF

PRLR CRCJF

PROC CRCJF

PRODH CRCJF

PROS1 CRCJF

PROSC CRCJF

PROZ CRCJF

PRPH CRCJF

PRPS1 CRCJF

PRSS12 CRCJF

PTEN CRCJF

PTGER1 CRCJF

PTGER2 CRCJF

PTGER4 CRCJF

PTGFR CRCJF

PTGIR CRCJF

PTGIS CRCJF

PTK2B CRCJF

PTPN2 CRCJF

PYCR1 CRCJF

PYCR2 CRCJF

PYCRL CRCJF

PYGL CRCJF

QDPR CRCJF

QPRT CRCJF

RAB11FIP1 CRCJF

RAB11FIP3 CRCJF

RAB11FIP5 CRCJF

RAB1A CRCJF

RAB3A CRCJF

RAB3B CRCJF

RAB8B CRCJF

RAC1 CRCJF

RAC2 CRCJF

RAC3 CRCJF

RAG1 CRCJF

RAG2 CRCJF

RAP1GAP CRCJF

RAPGEF2 CRCJF

RARA CRCJF

RARB CRCJF

RARG CRCJF

RARRES1 CRCJF

RARS CRCJF

RBP1 CRCJF

RBP3 CRCJF

RDH10 CRCJF

RDH11 CRCJF

RDH12 CRCJF

RDH13 CRCJF

RDH14 CRCJF

RDH5 CRCJF

RDH8 CRCJF

REN CRCJF

RERE CRCJF

REST CRCJF

RET CRCJF

RETSAT CRCJF

RFK CRCJF

RGCC CRCJF

RGS2 CRCJF

RHO CRCJF

RINT1 CRCJF

RIPK1 CRCJF

RIPK2 CRCJF

RLBP1 CRCJF

RNASE1 CRCJF

RNASE2 CRCJF

RNASE4 CRCJF

RNASE8 CRCJF

RNF207 CRCJF

ROCK1 CRCJF

ROCK2 CRCJF

RPL3 CRCJF

RPL31 CRCJF

RPL7A CRCJF

RPS6KA2 CRCJF

RPS6KA3 CRCJF

RRM1 CRCJF

RRM2 CRCJF

RRM2B CRCJF

RS1 CRCJF

RTN2 CRCJF

RUNX1 CRCJF

RXFP4 CRCJF

RXRB CRCJF

RXRG CRCJF

RYR1 CRCJF

RYR2 CRCJF

RYR3 CRCJF

S100A8 CRCJF

S100A9 CRCJF

S100G CRCJF

S1PR2 CRCJF

SALL1 CRCJF

SC5D CRCJF

SCD CRCJF

SCD5 CRCJF

SCGB1A1 CRCJF

SCN10A CRCJF

SCN11A CRCJF

SCN1A CRCJF

SCN1B CRCJF

SCN2A CRCJF

SCN2B CRCJF

SCN3A CRCJF

SCN3B CRCJF

SCN4A CRCJF

SCN4B CRCJF

SCN7A CRCJF

SCN8A CRCJF

SCN9A CRCJF

SCRIB CRCJF

SDC4 CRCJF

SDHA CRCJF

SDHAF2 CRCJF

SDHB CRCJF

SDHC CRCJF

SDHD CRCJF

SEC14L2 CRCJF

SEC14L3 CRCJF

SEC14L4 CRCJF

SEC14L6 CRCJF

SEC24B CRCJF

SEC61B CRCJF

SEMA4D CRCJF

SERPINB3 CRCJF

SERPINB7 CRCJF

SERPINE2 CRCJF

SERPINF2 CRCJF

SERPINH1 CRCJF

SFXN5 CRCJF

SHANK3 CRCJF

SHBG CRCJF

SHH CRCJF

SHMT1 CRCJF

SHMT2 CRCJF

SHOX2 CRCJF

SI CRCJF

SIGMAR1 CRCJF

SIPA1 CRCJF

SIRT2 CRCJF

SIX1 CRCJF

SIX3 CRCJF

SIX4 CRCJF

SLC11A1 CRCJF

SLC11A2 CRCJF

SLC13A1 CRCJF

SLC13A2 CRCJF

SLC13A3 CRCJF

SLC13A4 CRCJF

SLC13A5 CRCJF

SLC15A1 CRCJF

SLC15A2 CRCJF

SLC16A1 CRCJF

SLC16A12 CRCJF

SLC16A2 CRCJF

SLC16A3 CRCJF

SLC16A4 CRCJF

SLC16A5 CRCJF

SLC16A6 CRCJF

SLC16A7 CRCJF

SLC16A8 CRCJF

SLC17A7 CRCJF

SLC18A1 CRCJF

SLC18A2 CRCJF

SLC18A3 CRCJF

SLC19A1 CRCJF

SLC19A2 CRCJF

SLC19A3 CRCJF

SLC1A1 CRCJF

SLC1A3 CRCJF

SLC1A4 CRCJF

SLC1A5 CRCJF

SLC1A6 CRCJF

SLC22A1 CRCJF

SLC22A11 CRCJF

SLC22A4 CRCJF

SLC22A5 CRCJF

SLC22A6 CRCJF

SLC22A7 CRCJF

SLC22A8 CRCJF

SLC23A1 CRCJF

SLC23A2 CRCJF

SLC24A2 CRCJF

SLC25A1 CRCJF

SLC25A10 CRCJF

SLC25A12 CRCJF

SLC25A13 CRCJF

SLC25A15 CRCJF

SLC25A2 CRCJF

SLC25A20 CRCJF

SLC25A29 CRCJF

SLC25A32 CRCJF

SLC26A3 CRCJF

SLC26A6 CRCJF

SLC2A1 CRCJF

SLC2A2 CRCJF

SLC32A1 CRCJF

SLC34A1 CRCJF

SLC35F3 CRCJF

SLC35G1 CRCJF

SLC36A1 CRCJF

SLC38A3 CRCJF

SLC38A7 CRCJF

SLC3A1 CRCJF

SLC3A2 CRCJF

SLC44A4 CRCJF

SLC46A1 CRCJF

SLC47A1 CRCJF

SLC4A7 CRCJF

SLC52A2 CRCJF

SLC52A3 CRCJF

SLC5A6 CRCJF

SLC5A7 CRCJF

SLC6A14 CRCJF

SLC6A2 CRCJF

SLC6A5 CRCJF

SLC6A7 CRCJF

SLC6A8 CRCJF

SLC6A9 CRCJF

SLC7A1 CRCJF

SLC7A11 CRCJF

SLC7A2 CRCJF

SLC7A3 CRCJF

SLC7A4 CRCJF

SLC7A7 CRCJF

SLC7A8 CRCJF

SLC8A1 CRCJF

SLC8A2 CRCJF

SLC8A3 CRCJF

SLC8B1 CRCJF

SLC9A1 CRCJF

SLC9A3R1 CRCJF

SLCO1B1 CRCJF

SLCO1B3 CRCJF

SLIT2 CRCJF

SLITRK6 CRCJF

SMAD7 CRCJF

SMO CRCJF

SNAI1 CRCJF

SNAI2 CRCJF

SNCA CRCJF

SNTG2 CRCJF

SNW1 CRCJF

SOCS1 CRCJF

SOD2 CRCJF

SOD3 CRCJF

SORCS3 CRCJF

SOX15 CRCJF

SOX9 CRCJF

SP1 CRCJF

SP3 CRCJF

SPARC CRCJF

SPI1 CRCJF

SPR CRCJF

SPRY1 CRCJF

SPRY2 CRCJF

SPX CRCJF

SQRDL CRCJF

SRC CRCJF

SRD5A1 CRCJF

SRD5A2 CRCJF

SRD5A3 CRCJF

SST CRCJF

STAP1 CRCJF

STAR CRCJF

STAT5A CRCJF

STATH CRCJF

STIM1 CRCJF

STIM2 CRCJF

STUB1 CRCJF

STX1A CRCJF

STX3 CRCJF

SUCLA2 CRCJF

SUCLG1 CRCJF

SUCLG2 CRCJF

SUCNR1 CRCJF

SUGCT CRCJF

SULT1A1 CRCJF

SULT1A2 CRCJF

SULT1E1 CRCJF

SULT2A1 CRCJF

SULT2B1 CRCJF

SUMO1 CRCJF

SUOX CRCJF

SYT2 CRCJF

TAC1 CRCJF

TAC4 CRCJF

TACR2 CRCJF

TAF7 CRCJF

TAL1 CRCJF

TALDO1 CRCJF

TARS CRCJF

TARS2 CRCJF

TAS1R1 CRCJF

TAS1R2 CRCJF

TAS1R3 CRCJF

TBC1D32 CRCJF

TBPL1 CRCJF

TBR1 CRCJF

TBX21 CRCJF

TBXAS1 CRCJF

TCAF1 CRCJF

TCAF2 CRCJF

TCF3 CRCJF

TCN1 CRCJF

TERT CRCJF

TET1 CRCJF

TFAP2C CRCJF

TFPI CRCJF

TGFB2 CRCJF

TH CRCJF

THNSL1 CRCJF

THRA CRCJF

THRB CRCJF

THTPA CRCJF

TICAM2 CRCJF

TIGAR CRCJF

TIRAP CRCJF

TKT CRCJF

TKTL1 CRCJF

TLR3 CRCJF

TLR4 CRCJF

TMBIM6 CRCJF

TMEM110 CRCJF

TMLHE CRCJF

TNFAIP3 CRCJF

TNFSF11 CRCJF

TNNC1 CRCJF

TNR CRCJF

TOP2B CRCJF

TOX3 CRCJF

TP53I3 CRCJF

TPH1 CRCJF

TPH2 CRCJF

TPK1 CRCJF

TPM1 CRCJF

TPMT CRCJF

TPO CRCJF

TRDMT1 CRCJF

TREM1 CRCJF

TRIM24 CRCJF

TRIM28 CRCJF

TRPA1 CRCJF

TRPM8 CRCJF

TRPV3 CRCJF

TSPO CRCJF

TST CRCJF

TTPA CRCJF

TUBA1A CRCJF

TUBA1B CRCJF

TUBA1C CRCJF

TUBA3C CRCJF

TUBA3D CRCJF

TUBA4A CRCJF

TUBB CRCJF

TUBB1 CRCJF

TUBB2A CRCJF

TUBB2B CRCJF

TUBB4A CRCJF

TUBB4B CRCJF

TXNRD1 CRCJF

TXNRD3 CRCJF

TYMS CRCJF

TYRP1 CRCJF

UBE2B CRCJF

UBIAD1 CRCJF

UBR5 CRCJF

UCHL1 CRCJF

UCN2 CRCJF

UGCG CRCJF

UGT3A2 CRCJF

UGT8 CRCJF

UHRF1 CRCJF

UHRF2 CRCJF

UMPS CRCJF

UROD CRCJF

UROS CRCJF

USP7 CRCJF

UTS2 CRCJF

UTS2R CRCJF

VARS CRCJF

VCP CRCJF

VDAC1 CRCJF

VDAC2 CRCJF

VDAC3 CRCJF

VDR CRCJF

VKORC1 CRCJF

VKORC1L1 CRCJF

VTI1A CRCJF

WDR77 CRCJF

WLS CRCJF

WNT10B CRCJF

WNT11 CRCJF

WNT2 CRCJF

WNT2B CRCJF

WNT4 CRCJF

WNT5A CRCJF

WT1 CRCJF

XCL1 CRCJF

XRCC4 CRCJF

XRCC6BP1 CRCJF

YBX3 CRCJF

YWHAE CRCJF

ZEB2 CRCJF

ZFP42 CRCJF

ZFPM1 CRCJF

ZNF219 CRCJF

ZNF536 CRCJF

ZP3 CRCJF

ZPR1 CRCJF

ZRANB3 CRCJF

AASS CRCJF

ABAT CRCJF

ABCB1 CRCJF

ABCB11 CRCJF

ABCC1 CRCJF

ABCC2 CRCJF

ABCC4 CRCJF

ABCC6 CRCJF

ABCC8 CRCJF

ABCC9 CRCJF

ABCG1 CRCJF

ABL1 CRCJF

ABL2 CRCJF

ACADSB CRCJF

ACOX1 CRCJF

ACP1 CRCJF

ACSL1 CRCJF

ACSL3 CRCJF

ACSL4 CRCJF

ACSS1 CRCJF

ACSS2 CRCJF

ACTA1 CRCJF

ACVR1 CRCJF

ACVR1B CRCJF

ACVRL1 CRCJF

ADA CRCJF

ADCY1 CRCJF

ADCY5 CRCJF

ADH1C CRCJF

ADH5 CRCJF

ADK CRCJF

ADORA1 CRCJF

ADORA2A CRCJF

ADORA2B CRCJF

ADORA3 CRCJF

ADRBK1 CRCJF

ADRBK2 CRCJF

AFG3L2 CRCJF

AHCY CRCJF

AHR CRCJF

AHSP CRCJF

AK1 CRCJF

AK2 CRCJF

AK8 CRCJF

AKR1C1 CRCJF

AKR1C2 CRCJF

AKR1C3 CRCJF

AKR1D1 CRCJF

AKT1 CRCJF

ALDH18A1 CRCJF

ALDH2 CRCJF

ALDH5A1 CRCJF

ALK CRCJF

ALKBH2 CRCJF

ALKBH3 CRCJF

ALOX15 CRCJF

ALOX5 CRCJF

AMHR2 CRCJF

AMT CRCJF

AMY1A CRCJF

AMY2A CRCJF

AMY2B CRCJF

ANXA1 CRCJF

ANXA3 CRCJF

APAF1 CRCJF

APP CRCJF

APRT CRCJF

AQP1 CRCJF

AR CRCJF

ARAF CRCJF

ARF1 CRCJF

ARF6 CRCJF

ARG1 CRCJF

ARG2 CRCJF

ASNA1 CRCJF

ASNS CRCJF

ASRGL1 CRCJF

ASS1 CRCJF

ATIC CRCJF

ATOX1 CRCJF

ATP1A1 CRCJF

ATP1A2 CRCJF

ATP1A3 CRCJF

ATP5A1 CRCJF

ATP5B CRCJF

ATP5C1 CRCJF

AURKB CRCJF

B3GAT1 CRCJF

B3GAT3 CRCJF

B4GALT1 CRCJF

BAG1 CRCJF

BALF5 CRCJF

BAMF_RS28815 CRCJF

BBOX1 CRCJF

BC1747 CRCJF

BC1844 CRCJF

BC2969 CRCJF

BCAT1 CRCJF

BCAT2 CRCJF

BCHE CRCJF

BLVRB CRCJF

BPI CRCJF

BST1 CRCJF

BXE_RS00860 CRCJF

C8G CRCJF

CA1 CRCJF

CA12 CRCJF

CA14 CRCJF

CA2 CRCJF

CA3 CRCJF

CA4 CRCJF

CA5A CRCJF

CA5B CRCJF

CA6 CRCJF

CA7 CRCJF

CA9 CRCJF

CACNA1C CRCJF

CACNA1D CRCJF

CACNA1F CRCJF

CACNA1S CRCJF

CACNB1 CRCJF

CACNB2 CRCJF

CACNB3 CRCJF

CACNB4 CRCJF

CALM1 CRCJF

CASK CRCJF

CASP3 CRCJF

CBR1 CRCJF

CCBL2 CRCJF

CCT3 CRCJF

CDA CRCJF

CDK15 CRCJF

CDK6 CRCJF

CEBPB CRCJF

CES1 CRCJF

CFTR CRCJF

CHRM3 CRCJF

CLEC14A CRCJF

CLEC4E CRCJF

CLPS CRCJF

CMPK1 CRCJF

CNR1 CRCJF

CNR2 CRCJF

COMT CRCJF

COMTD1 CRCJF

COX4I1 CRCJF

COX5A CRCJF

COX5B CRCJF

COX6A2 CRCJF

COX6B1 CRCJF

COX6C CRCJF

COX7A1 CRCJF

COX7B CRCJF

COX7C CRCJF

COX8A CRCJF

CP CRCJF

CPQ CRCJF

CPS1 CRCJF

CPT1A CRCJF

CPT2 CRCJF

CRAT CRCJF

CREB1 CRCJF

CROT CRCJF

CRYZ CRCJF

CSNK1G2 CRCJF

CSNK2A1 CRCJF

CSNK2B CRCJF

CTBP1 CRCJF

CTPS1 CRCJF

CTRB1 CRCJF

CYB5A CRCJF

CYC1 CRCJF

CYP17A1 CRCJF

CYP19A1 CRCJF

CYP1A2 CRCJF

CYP1B1 CRCJF

CYP27B1 CRCJF

CYP2B6 CRCJF

CYP2C8 CRCJF

CYP2C9 CRCJF

CYP2D6 CRCJF

CYP2E1 CRCJF

CYP3A4 CRCJF

CYP3A43 CRCJF

CYP3A5 CRCJF

CYP3A7 CRCJF

CYTH2 CRCJF

DAM CRCJF

DAPK1 CRCJF

DBH CRCJF

DCK CRCJF

DGKA CRCJF

DGKD CRCJF

DGKG CRCJF

DHFR CRCJF

DHFRL1 CRCJF

DHODH CRCJF

DHRS4L1 CRCJF

DNMT1 CRCJF

DNPEP CRCJF

DPH5 CRCJF

DPYD CRCJF

DR_A0214 CRCJF

DTYMK CRCJF

DVU2680 CRCJF

E CRCJF

EARS2 CRCJF

ECI2 CRCJF

EDNRA CRCJF

EEF2 CRCJF

EFTUD1 CRCJF

EGLN1 CRCJF

EGLN2 CRCJF

EGLN3 CRCJF

EIF3F CRCJF

ELOVL4 CRCJF

ENPEP CRCJF

ENPP1 CRCJF

EPHA2 CRCJF

EPHB2 CRCJF

EPRS CRCJF

ERG11 CRCJF

ESR1 CRCJF

ESR2 CRCJF

ESRRA CRCJF

ESRRB CRCJF

ESRRG CRCJF

ETFDH CRCJF

EXTL2 CRCJF

F2 CRCJF

FABP6 CRCJF

FADS1 CRCJF

FADS2 CRCJF

FBP1 CRCJF

FECH CRCJF

FEN1 CRCJF

FFAR1 CRCJF

FGF1 CRCJF

FHIT CRCJF

FKBP1A CRCJF

FOLH1 CRCJF

FOLR1 CRCJF

FOLR2 CRCJF

FOLR3 CRCJF

FPGS CRCJF

FTCD CRCJF

FTH1 CRCJF

FTL CRCJF

FURIN CRCJF

FXN CRCJF

GABBR1 CRCJF

GABBR2 CRCJF

GABRA1 CRCJF

GABRA2 CRCJF

GABRA3 CRCJF

GABRA4 CRCJF

GABRA5 CRCJF

GABRA6 CRCJF

GABRB1 CRCJF

GABRB2 CRCJF

GABRB3 CRCJF

GABRD CRCJF

GABRE CRCJF

GABRG1 CRCJF

GABRG2 CRCJF

GABRG3 CRCJF

GABRP CRCJF

GABRQ CRCJF

GAD1 CRCJF

GAD2 CRCJF

GALE CRCJF

GALK1 CRCJF

GAMT CRCJF

GAPDH CRCJF

GAPDHS CRCJF

GART CRCJF

GATB CRCJF

GATM CRCJF

GBA CRCJF

GCK CRCJF

GCLC CRCJF

GCLM CRCJF

GGACT CRCJF

GGCX CRCJF

GIG18 CRCJF

GLO1 CRCJF

GLRA3 CRCJF

GLS CRCJF

GLS2 CRCJF

GLT6D1 CRCJF

GLTP CRCJF

GLUD1 CRCJF

GLUD2 CRCJF

GLUL CRCJF

GM2A CRCJF

GMPS CRCJF

GNAS CRCJF

GNMT CRCJF

GNPDA1 CRCJF

GOT1 CRCJF

GOT2 CRCJF

GPBAR1 CRCJF

GPER1 CRCJF

GPHN CRCJF

GPT CRCJF

GPT2 CRCJF

GRIA1 CRCJF

GRIA2 CRCJF

GRIA3 CRCJF

GRIA4 CRCJF

GRID1 CRCJF

GRID2 CRCJF

GRIK1 CRCJF

GRIK2 CRCJF

GRIK3 CRCJF

GRIK4 CRCJF

GRIK5 CRCJF

GRIN1 CRCJF

GRIN2A CRCJF

GRIN2B CRCJF

GRIN2C CRCJF

GRIN2D CRCJF

GRIN3A CRCJF

GRIN3B CRCJF

GRM1 CRCJF

GRM4 CRCJF

GRM7 CRCJF

GRM8 CRCJF

GSG2 CRCJF

GSK3B CRCJF

GSS CRCJF

GST CRCJF

GSTA1 CRCJF

GSTP1 CRCJF

GUCA1A CRCJF

GYG1 CRCJF

HAO1 CRCJF

HAO2 CRCJF

HBA1 CRCJF

HBB CRCJF

HCAR3 CRCJF

HCK CRCJF

HCN2 CRCJF

HDAC2 CRCJF

HDAC8 CRCJF

HDAC9 CRCJF

HIBCH CRCJF

HINT1 CRCJF

HK1 CRCJF

HMGCR CRCJF

HMOX1 CRCJF

HNF4A CRCJF

HNF4G CRCJF

HNMT CRCJF

HOXA10 CRCJF

HPGDS CRCJF

HSD11B1 CRCJF

HSD11B2 CRCJF

HSD17B1 CRCJF

HSD17B11 CRCJF

HSD3B1 CRCJF

HSP90AA1 CRCJF

HSP90B1 CRCJF

HSPA2 CRCJF

HSPA5 CRCJF

HSPA8 CRCJF

IFNB1 CRCJF

IGF1R CRCJF

IGHG1 CRCJF

IGHG2 CRCJF

IKBKB CRCJF

IL1B CRCJF

IL6 CRCJF

IMPDH1 CRCJF

IMPDH2 CRCJF

INS CRCJF

ISG20 CRCJF

ISYNA1 CRCJF

ITGA5 CRCJF

ITGAL CRCJF

ITGB2 CRCJF

ITGB3 CRCJF

ITPKA CRCJF

ITPR1 CRCJF

JAK1 CRCJF

KANSL3 CRCJF

KCNJ3 CRCJF

KCNMA1 CRCJF

KDM5D CRCJF

KHSRP CRCJF

KIF1A CRCJF

KIF2C CRCJF

KRT12 CRCJF

KRT2 CRCJF

KRTAP5-2 CRCJF

KRTAP5-3 CRCJF

L CRCJF

L3HYPDH CRCJF

LALBA CRCJF

LCK CRCJF

LCN2 CRCJF

LCT CRCJF

LCTL CRCJF

LDHA CRCJF

LGALS1 CRCJF

LGALS2 CRCJF

LGALS3 CRCJF

LGALS7 CRCJF

LGSN CRCJF

LIP3 CRCJF

LPA CRCJF

LPL CRCJF

LSM6 CRCJF

LSS CRCJF

LTB4R CRCJF

LTB4R2 CRCJF

LTF CRCJF

LY96 CRCJF

LYZ CRCJF

MAFF_RS13750 CRCJF

MAN1B1 CRCJF

MAN2A1 CRCJF

MAOB CRCJF

MAPK10 CRCJF

MAPK12 CRCJF

MAPK3 CRCJF

MAPK8IP1 CRCJF

MB CRCJF

MBL2 CRCJF

ME2 CRCJF

MED1 CRCJF

MGAT1 CRCJF

MIF CRCJF

MIP CRCJF

MPN348 CRCJF

MPO CRCJF

MTAP CRCJF

MT-CO1 CRCJF

MT-CO2 CRCJF

MT-CO3 CRCJF

MT-CYB CRCJF

MTFMT CRCJF

MTHFD1 CRCJF

MTHFD2 CRCJF

MTHFR CRCJF

MTNR1A CRCJF

MTNR1B CRCJF

MTR CRCJF

MTTP CRCJF

NADSYN1 CRCJF

NAE1 CRCJF

NAGA CRCJF

NAGS CRCJF

NARS CRCJF

NARS2 CRCJF

NCAN CRCJF

NCOA1 CRCJF

NCOA2 CRCJF

NEIL1 CRCJF

NEIL2 CRCJF

NFKB1 CRCJF

NFKB2 CRCJF

NFKBIA CRCJF

NME1 CRCJF

NNMT CRCJF

NOS1 CRCJF

NOS2 CRCJF

NPPB CRCJF

NQO1 CRCJF

NQO2 CRCJF

NR0B1 CRCJF

NR1H2 CRCJF

NR1H3 CRCJF

NR1H4 CRCJF

NR1I2 CRCJF

NR1I3 CRCJF

NR3C1 CRCJF

NR3C2 CRCJF

NT5C2 CRCJF

NUDT9 CRCJF

OAS1 CRCJF

OAT CRCJF

OAZ1 CRCJF

OAZ2 CRCJF

OAZ3 CRCJF

OGDH CRCJF

OGFOD1 CRCJF

OGFOD2 CRCJF

OPLAH CRCJF

OPRK1 CRCJF

ORM1 CRCJF

OTC CRCJF

P CRCJF

P3H1 CRCJF

P3H2 CRCJF

P3H3 CRCJF

P4HA1 CRCJF

P4HA2 CRCJF

P4HTM CRCJF

P5CR2 CRCJF

PAEP CRCJF

PAM CRCJF

PAPS CRCJF

PAPSS1 CRCJF

PARP1 CRCJF

PARS2 CRCJF

PB1 CRCJF

PCMT1 CRCJF

PCTP CRCJF

PCYT1A CRCJF

PCYT1B CRCJF

PDE4A CRCJF

PDE4B CRCJF

PDE4C CRCJF

PDE4D CRCJF

PDE7A CRCJF

PDE7B CRCJF

PECR CRCJF

PFAS CRCJF

PFKFB4 CRCJF

PGR CRCJF

PHOSPHO1 CRCJF

PHYH CRCJF

PI4K2B CRCJF

PIK3CA CRCJF

PIK3CG CRCJF

PIK3R1 CRCJF

PIM1 CRCJF

PISD CRCJF

PITPNA CRCJF

PKIA CRCJF

PLA2G1B CRCJF

PLA2G2A CRCJF

PLA2G2D CRCJF

PLA2G2E CRCJF

PLAT CRCJF

PLD1 CRCJF

PLD2 CRCJF

PLG CRCJF

PLK1 CRCJF

PLOD1 CRCJF

PLOD2 CRCJF

PLOD3 CRCJF

PMP2 CRCJF

PNLIP CRCJF

PNMT CRCJF

PNP CRCJF

PNPO CRCJF

POLA1 CRCJF

POLB CRCJF

POLE CRCJF

POLE2 CRCJF

POLE3 CRCJF

POLE4 CRCJF

POR CRCJF

PPA_RS05235 CRCJF

PPARA CRCJF

PPARD CRCJF

PPARG CRCJF

PPAT CRCJF

PPCDC CRCJF

PPIA CRCJF

PPIB CRCJF

PPIC CRCJF

PPIF CRCJF

PPIG CRCJF

PPIH CRCJF

PPP1CC CRCJF

PPP2CA CRCJF

PPP2CB CRCJF

PPP3CA CRCJF

PPP3R1 CRCJF

PPP5C CRCJF

PPT1 CRCJF

PREP CRCJF

PRKAA1 CRCJF

PRKAA2 CRCJF

PRKAB1 CRCJF

PRKAB2 CRCJF

PRKACA CRCJF

PRKAG1 CRCJF

PRKAG2 CRCJF

PRKAG3 CRCJF

PRKAR1A CRCJF

PRKAR2B CRCJF

PRKCA CRCJF

PRKCB CRCJF

PRLR CRCJF

PRMT1 CRCJF

PRMT3 CRCJF

PROCR CRCJF

PRODH CRCJF

PROSC CRCJF

PSAP CRCJF

PSAT1 CRCJF

PTDSS1 CRCJF

PTDSS2 CRCJF

PTEN CRCJF

PTGR1 CRCJF

PTGS1 CRCJF

PTGS2 CRCJF

PTK2B CRCJF

PVALB CRCJF

PVR CRCJF

PYCR1 CRCJF

PYCR2 CRCJF

PYCRL CRCJF

PYGL CRCJF

PYGM CRCJF

QPRT CRCJF

RAD51 CRCJF

RARG CRCJF

RCVRN CRCJF

RFK CRCJF

RHO CRCJF

RNASE1 CRCJF

RORA CRCJF

RPL10L CRCJF

RPL11 CRCJF

RPL13A CRCJF

RPL15 CRCJF

RPL19 CRCJF

RPL23 CRCJF

RPL23A CRCJF

RPL26L1 CRCJF

RPL3 CRCJF

RPL37 CRCJF

RPL8 CRCJF

RPS6KA3 CRCJF

RPS6KA4 CRCJF

RRM1 CRCJF

RRM2 CRCJF

RRM2B CRCJF

RSL24D1 CRCJF

RTCB CRCJF

RUVBL2 CRCJF

RXRA CRCJF

RXRB CRCJF

RXRG CRCJF

S100B CRCJF

S100P CRCJF

SCARB1 CRCJF

SCN10A CRCJF

SCN11A CRCJF

SCN1A CRCJF

SCN1B CRCJF

SCN2A CRCJF

SCN2B CRCJF

SCN3A CRCJF

SCN3B CRCJF

SCN4A CRCJF

SCN4B CRCJF

SCN5A CRCJF

SCN7A CRCJF

SCN8A CRCJF

SCN9A CRCJF

SDHA CRCJF

SDHB CRCJF

SDHC CRCJF

SDHD CRCJF

SEC14L2 CRCJF

SEC14L3 CRCJF

SEC14L4 CRCJF

SERPINA1 CRCJF

SETD7 CRCJF

SF3B3 CRCJF

SFTPD CRCJF

SGK1 CRCJF

SHBG CRCJF

SHMT1 CRCJF

SHMT2 CRCJF

SIGLEC1 CRCJF

SIGMAR1 CRCJF

SIRT3 CRCJF

SIRT5 CRCJF

SLC18A2 CRCJF

SLC1A1 CRCJF

SLC1A2 CRCJF

SLC1A3 CRCJF

SLC1A5 CRCJF

SLC1A6 CRCJF

SLC1A7 CRCJF

SLC22A4 CRCJF

SLC22A5 CRCJF

SLC23A1 CRCJF

SLC25A15 CRCJF

SLC25A18 CRCJF

SLC25A2 CRCJF

SLC25A20 CRCJF

SLC25A22 CRCJF

SLC25A29 CRCJF

SLC25A4 CRCJF

SLC2A1 CRCJF

SLC38A3 CRCJF

SLC5A2 CRCJF

SLC6A14 CRCJF

SLC6A2 CRCJF

SLC6A3 CRCJF

SLC6A4 CRCJF

SLC6A7 CRCJF

SLC7A1 CRCJF

SLC7A11 CRCJF

SLC7A2 CRCJF

SLC7A3 CRCJF

SLC7A4 CRCJF

SLC8A1 CRCJF

SLCO1B3 CRCJF

SMARCA5 CRCJF

SMPD3 CRCJF

SMPD4 CRCJF

SMU_260 CRCJF

SOAT1 CRCJF

SOAT2 CRCJF

SOD1 CRCJF

SQLE CRCJF

SRD5A1 CRCJF

SREBF1 CRCJF

SRPK2 CRCJF

STK17B CRCJF

STM4066 CRCJF

SULT2A1 CRCJF

SULT2B1 CRCJF

SYK CRCJF

TAS1R2 CRCJF

TAT CRCJF

TF CRCJF

TFRC CRCJF

TGFBR2 CRCJF

TGM3 CRCJF

THYA CRCJF

TK CRCJF

TK2 CRCJF

TLR4 CRCJF

TLR7 CRCJF

TM0024 CRCJF

TM0065 CRCJF

TM0096 CRCJF

TM0306 CRCJF

TM0588 CRCJF

TM0857 CRCJF

TM1070 CRCJF

TM1131 CRCJF

TM1223 CRCJF

TM1468 CRCJF

TMLHE CRCJF

TNF CRCJF

TNK2 CRCJF

TOP2A CRCJF

TOP2B CRCJF

TP53 CRCJF

TPK1 CRCJF

TPR CRCJF

TRAPPC3 CRCJF

TRDMT1 CRCJF

TRPA1 CRCJF

TRPM7 CRCJF

TRPM8 CRCJF

TRPV1 CRCJF

TRPV3 CRCJF

TTHA0667 CRCJF

TTHA1133 CRCJF

TTHA1134 CRCJF

TTHA1135 CRCJF

TTHA1435 CRCJF

TTHA1586 CRCJF

TUBA4A CRCJF

TUBB CRCJF

TYMS CRCJF

TYR CRCJF

UAP1 CRCJF

UBA1 CRCJF

UCK2 CRCJF

UCKL1 CRCJF

UGT3A1 CRCJF

UL30 CRCJF

UMPS CRCJF

UQCR10 CRCJF

UQCRB CRCJF

UQCRC1 CRCJF

UQCRC2 CRCJF

UQCRFS1 CRCJF

UQCRH CRCJF

UQCRQ CRCJF

VCP CRCJF

VDR CRCJF

VKORC1 CRCJF

VLDLR CRCJF

XDH CRCJF

YARS CRCJF

YWHAE CRCJF

AADAT CRCJF

ABAT CRCJF

ABCA1 CRCJF

ABCB1 CRCJF

ABL1 CRCJF

ABO CRCJF

ACACB CRCJF

ACHE CRCJF

ACTA1 CRCJF

ACTB CRCJF

ACYP2 CRCJF

ADCY2 CRCJF

ADH1B CRCJF

ADH1C CRCJF

ADORA1 CRCJF

AHR CRCJF

AHSP CRCJF

AKR1C1 CRCJF

AKR1C3 CRCJF

AKR1D1 CRCJF

AKT1 CRCJF

ALAD CRCJF

ALB CRCJF

ALDH1L1 CRCJF

ALOX5 CRCJF

AMY1A CRCJF

ANXA1 CRCJF

AR CRCJF

aroQ CRCJF

ART1 CRCJF

ASNS CRCJF

ATP1A1 CRCJF

ATP5A1 CRCJF

ATP8A1 CRCJF

BCHE CRCJF

BCL2 CRCJF

CDIPT CRCJF

COMT CRCJF

CTRB1 CRCJF

cumD CRCJF

CYP2A6 CRCJF

def CRCJF

EPRS CRCJF

ERG11 CRCJF

FAH CRCJF

GABRA1 CRCJF

GAPDHS CRCJF

GLO1 CRCJF

HCAR2 CRCJF

IGHG1 CRCJF

IGKV2-30 CRCJF

KYNU CRCJF

MAOA CRCJF

MBL2 CRCJF

MIF CRCJF

NA CRCJF

NR1I2 CRCJF

NR3C1 CRCJF

PTGR1 CRCJF

RORA CRCJF

SDHA CRCJF

SLC19A2 CRCJF

TOP2A CRCJF

TRPA1 CRCJF

TYR CRCJF

VDR CRCJF
