# Supplementary material for: Decoding the Mechanism of CheReCunJin Formula in Treating Sjögren's Syndrome Based on Network Pharmacology and Molecular Docking
Source: Evid Based Complement Alternat Med. 2022 Sep 20;2022:1193846. doi: 10.1155/2022/1193846 (PMC9553462; doi:10.1155/2022/1193846)
Supplement: Supplementary Materials — Table S1: the active ingredients of CRCJ. Table S2: the nodes and edges of the network. Table S3: summary table of drug targets. Table S4: summary table of disease targets. Table S5: network cluster results. [file 1193846.f1.zip › 1193846.f1/Supplementary Table 4.docx]

Supplementary Table 4

CASP3 CTD

IL1B CTD

VEGFA CTD

AGT CTD

CASP9 CTD

MPO CTD

NPPB CTD

TNF CTD

CYP3A4 CTD

ALB CTD

CAT CTD

CCL2 CTD

CDKN1A CTD

CXCL8 CTD

CYP1A2 CTD

IL6 CTD

LITAF CTD

NR1D1 CTD

PER1 CTD

PPARD CTD

PRELP CTD

PRKCB CTD

RELA CTD

SMAD7 CTD

SOD1 CTD

TGFBI CTD

TNFAIP2 CTD

TP53 CTD

IRAK1BP1 DisGeNET

STAT4 DisGeNET

DGKQ DisGeNET

GTF2I DisGeNET

TNFAIP3 DisGeNET

PRDM1 DisGeNET

PHIP DisGeNET

FCGR2A DisGeNET

IL12A DisGeNET

NCF1 DisGeNET

ITSN2 DisGeNET

TNIP1 DisGeNET

PTTG1 DisGeNET

TNF DisGeNET

TNFSF13B DisGeNET

TGFB1 DisGeNET

ID3 DisGeNET

THBS1 DisGeNET

FAS DisGeNET

HMOX1 DisGeNET

IL2 DisGeNET

IL2RA DisGeNET

AIRE DisGeNET

IL4 DisGeNET

ST14 DisGeNET

TRAF3IP2 DisGeNET

MAP3K14 DisGeNET

NFKBIA DisGeNET

E2F1 DisGeNET

RORC DisGeNET

CCR7 DisGeNET

CYP19A1 DisGeNET

CTSS DisGeNET

LYZ DisGeNET

TP53 DisGeNET

BCL2 DisGeNET

PRKAR1A DisGeNET

CXADRP1 DisGeNET

ARR3 DisGeNET

IL17A DisGeNET

IL10 DisGeNET

ESR1 DisGeNET

SPG7 DisGeNET

STAT3 DisGeNET

IFNG DisGeNET

CASR DisGeNET

CXADR DisGeNET

TRIM13 DisGeNET

HBG1 DisGeNET

HBA2 DisGeNET

VCAM1 DisGeNET

NR1I3 DisGeNET

F5 DisGeNET

BCR DisGeNET

CFB DisGeNET

CCL25 DisGeNET

IL21 DisGeNET

TNFSF13 DisGeNET

RAD23B DisGeNET

PTN DisGeNET

MIR495 DisGeNET

B2M DisGeNET

ADIPOQ DisGeNET

ATF7IP DisGeNET

CX3CL1 DisGeNET

FAM167A DisGeNET

TACR1 DisGeNET

TFPI DisGeNET

ACE DisGeNET

BMP6 DisGeNET

TLR3 DisGeNET

VTCN1 DisGeNET

SLC6A4 DisGeNET

CDR3 DisGeNET

TSPYL2 DisGeNET

SELE DisGeNET

ERVK-6 DisGeNET

BLK DisGeNET

THBD DisGeNET

FCRL4 DisGeNET

CXCL12 DisGeNET

RBP4 DisGeNET

DYM DisGeNET

ERVK-20 DisGeNET

FLVCR1 DisGeNET

HBA1 DisGeNET

HBB DisGeNET

HBG2 DisGeNET

ERVW-1 DisGeNET

ICAM1 DisGeNET

AQP8 DisGeNET

IL1B DisGeNET

FASLG DisGeNET

IL5 DisGeNET

GLO1 DisGeNET

PUF60 DisGeNET

ERVW-4 DisGeNET

CXCL13 DisGeNET

CCR9 DisGeNET

HPSE DisGeNET

CTLA4 DisGeNET

DECR1 DisGeNET

ERG DisGeNET

ALB DisGeNET

F2 DisGeNET

F2RL1 DisGeNET

IL7 DisGeNET

CXCL8 DisGeNET

IL12RB1 DisGeNET

MMP3 DisGeNET

MMP9 DisGeNET

MTHFR DisGeNET

MUC1 DisGeNET

ACR DisGeNET

FURIN DisGeNET

IL21R DisGeNET

IL22 DisGeNET

PC DisGeNET

PLS3 DisGeNET

MMP2 DisGeNET

CXCL9 DisGeNET

AQP5 DisGeNET

IDO1 DisGeNET

AQP9 DisGeNET

ITGB2 DisGeNET

GDF6 DisGeNET

LBR DisGeNET

MIR10B DisGeNET

MIR155 DisGeNET

MDK DisGeNET

MIF DisGeNET

PMCH DisGeNET

CHRM1 DrugBank

CHRM3 DrugBank

CYP3A4 DrugBank

CYP2D6 DrugBank

FMO1 DrugBank

DNA DrugBank

TLR7 DrugBank

TLR9 DrugBank

CYP2D6 DrugBank

ABCB1 DrugBank

ALB DrugBank

ORM1 DrugBank

ORM2 DrugBank

CYP3A4 DrugBank

SLCO1A2 DrugBank

ACE2 DrugBank

CYP2C8 DrugBank

CHRM1 DrugBank

CHRM2 DrugBank

CYP2A6 DrugBank

CHRM3 DrugBank

CYP3A4 DrugBank

CHRM4 DrugBank

SERPINA6 DrugBank

CYP3A4 DrugBank

NR3C1 DrugBank

SLCO1A2 DrugBank

ABCB1 DrugBank

ALB DrugBank

NR3C1 DrugBank

CYP3A4 DrugBank

ABCB1 DrugBank

SLCO1A2 DrugBank

CYP2C19 DrugBank

ALB DrugBank

CYP3A5 DrugBank

SERPINA6 DrugBank

CYP3A4 DrugBank

CYP3A43 DrugBank

CYP3A43 DrugBank

CYP3A7 DrugBank

CYP2A6 DrugBank

CYP1B1 DrugBank

CYP2B6 DrugBank

CYP2C8 DrugBank

CYP2C9 DrugBank

HSD11B1 DrugBank

FBN1 GeneCards

BRCA1 GeneCards

TP53 GeneCards

PTEN GeneCards

SMARCA4 GeneCards

FGFR2 GeneCards

ERCC6 GeneCards

FLNA GeneCards

TSC2 GeneCards

NOD2 GeneCards

COL3A1 GeneCards

NLRP3 GeneCards

GJB2 GeneCards

RET GeneCards

COL5A1 GeneCards

FGFR3 GeneCards

FGFR1 GeneCards

ERCC8 GeneCards

HSPG2 GeneCards

HRAS GeneCards

LMNA GeneCards

FAS GeneCards

SCN1A GeneCards

KCNJ2 GeneCards

IFIH1 GeneCards

SCN10A GeneCards

CHD7 GeneCards

IL10 GeneCards

COL1A1 GeneCards

AKT1 GeneCards

COL5A2 GeneCards

PLOD1 GeneCards

EP300 GeneCards

TNFRSF1A GeneCards

TP63 GeneCards

ALB GeneCards

TNF GeneCards

LRRC56 GeneCards

MUSK GeneCards

ERCC4 GeneCards

AIRE GeneCards

PAX6 GeneCards

FOXG1 GeneCards

FOXP3 GeneCards

ERCC2 GeneCards

WDPCP GeneCards

SLC12A3 GeneCards

CD40LG GeneCards

POLD1 GeneCards

CACNB2 GeneCards

HNF1B GeneCards

ARID1B GeneCards

PSTPIP1 GeneCards

HLA-B GeneCards

DSP GeneCards

LRP2 GeneCards

F5 GeneCards

THBD GeneCards

GJA1 GeneCards

GJB6 GeneCards

SCN11A GeneCards

SMARCA2 GeneCards

SPTAN1 GeneCards

HLA-DRB1 GeneCards

SBDS GeneCards

FGF10 GeneCards

MEFV GeneCards

HARS1 GeneCards

ACTB GeneCards

ADA2 GeneCards

IL6 GeneCards

ERCC1 GeneCards

HPRT1 GeneCards

BDNF GeneCards

KCNJ10 GeneCards

ADAMTS13 GeneCards

ERCC5 GeneCards

FASLG GeneCards

NDUFAF2 GeneCards

MTOR GeneCards

PGBD3 GeneCards

SNRPN GeneCards

SIL1 GeneCards

TTR GeneCards

LOC101448202 GeneCards

POLR1C GeneCards

KIT GeneCards

FKBP14 GeneCards

CTLA4 GeneCards

TLR4 GeneCards

TH GeneCards

AKT3 GeneCards

JAK2 GeneCards

LEP GeneCards

SOX2 GeneCards

CRLF1 GeneCards

TBX4 GeneCards

MTHFR GeneCards

SREBF1 GeneCards

EDAR GeneCards

LOC113939944 GeneCards

IL1B GeneCards

CRP GeneCards

ABCC9 GeneCards

ERCC3 GeneCards

NECTIN1 GeneCards

GDF5 GeneCards

MSX1 GeneCards

IFNG GeneCards

CP GeneCards

GRIN2B GeneCards

RYR1 GeneCards

ERBB3 GeneCards

EGFR GeneCards

ZMPSTE24 GeneCards

GPC3 GeneCards

C4A GeneCards

PAFAH1B1 GeneCards

GSN GeneCards

TNXB GeneCards

CCNH GeneCards

IARS2 GeneCards

MPLKIP GeneCards

WNT10A GeneCards

MVK GeneCards

GNAQ GeneCards

AEBP1 GeneCards

HELLS GeneCards

HLA-A GeneCards

TRIM21 GeneCards

STAT4 GeneCards

CTNNA1 GeneCards

TGFB1 GeneCards

IL4 GeneCards

FGF9 GeneCards

FGF8 GeneCards

CFHR1 GeneCards

APOE GeneCards

SAMD9 GeneCards

TTN GeneCards

ACE GeneCards

VEGFA GeneCards

CCR6 GeneCards

SSB GeneCards

SCN9A GeneCards

LBR GeneCards

HLA-DQB1 GeneCards

CD27 GeneCards

CD40 GeneCards

CXCL8 GeneCards

SYT2 GeneCards

FKBP14-AS1 GeneCards

MMP2 GeneCards

APOH GeneCards

CD4 GeneCards

SLC39A13 GeneCards

GDNF GeneCards

TNFAIP3 GeneCards

IL1A GeneCards

CUL4B GeneCards

GTF2H5 GeneCards

B2M GeneCards

NGF GeneCards

RNF125 GeneCards

CCL2 GeneCards

CD79A GeneCards

U2AF1 GeneCards

IL23R GeneCards

CACNA1A GeneCards

IKBKG GeneCards

PCDH19 GeneCards

EFL1 GeneCards

ERCC8-AS1 GeneCards

F2 GeneCards

APOA1 GeneCards

ADA GeneCards

LOC102724058 GeneCards

H2AC18 GeneCards

HARS2 GeneCards

EFEMP1 GeneCards

ERBB2 GeneCards

RAD51 GeneCards

ERAP1 GeneCards

IL2RA GeneCards

AP1B1 GeneCards

CCR1 GeneCards

IL2 GeneCards

COL17A1 GeneCards

CALR GeneCards

IL1RN GeneCards

USP7 GeneCards

AFF2 GeneCards

IL12A GeneCards

UVSSA GeneCards

SYNGAP1 GeneCards

TNFSF13B GeneCards

MTTP GeneCards

RTEL1 GeneCards

BIVM-ERCC5 GeneCards

DDX11 GeneCards

TMPO GeneCards

RO60 GeneCards

CHD4 GeneCards

ABL1 GeneCards

KARS1 GeneCards

REN GeneCards

LTF GeneCards

SST GeneCards

DNAH8 GeneCards

IKZF1 GeneCards

KLRC4 GeneCards

MMP9 GeneCards

ESR1 GeneCards

FGF3 GeneCards

IL17A GeneCards

IL12A-AS1 GeneCards

CDKN1A GeneCards

VWF GeneCards

IL7R GeneCards

STAT1 GeneCards

MAB21L1 GeneCards

PTPN22 GeneCards

ICAM1 GeneCards

LPL GeneCards

HLA-C GeneCards

HADHA GeneCards

MMP14 GeneCards

UBAC2 GeneCards

TARS1 GeneCards

IL18 GeneCards

NFKBIA GeneCards

FGF2 GeneCards

FN1 GeneCards

CHRM3 GeneCards

APOB GeneCards

PSORS1C1 GeneCards

MIR155 GeneCards

PIK3C2A GeneCards

EGF GeneCards

SERPINC1 GeneCards

EDA GeneCards

TGM1 GeneCards

VCP GeneCards

HLA-DQA1 GeneCards

MPO GeneCards

VAMP1 GeneCards

ZNRD2 GeneCards

NKX2-1 GeneCards

CFTR GeneCards

TPO GeneCards

CD28 GeneCards

GPT GeneCards

P4HB GeneCards

CCL5 GeneCards

FGF17 GeneCards

IL13 GeneCards

AMPH GeneCards

DENND11 GeneCards

PRTN3 GeneCards

CSF2 GeneCards

PSMB9 GeneCards

CHUK GeneCards

TNFRSF1B GeneCards

AIMP1 GeneCards

PLCB1 GeneCards

RNF113A GeneCards

AQP5 GeneCards

IFNA1 GeneCards

CD8A GeneCards

SSNA1 GeneCards

PRF1 GeneCards

MUC5AC GeneCards

CASP3 GeneCards

IRF5 GeneCards

DDB1 GeneCards

POLR1A GeneCards

NBEA GeneCards

OTULIN GeneCards

SPP1 GeneCards

NLRP1 GeneCards

ICOSLG GeneCards

GATAD2B GeneCards

FGF23 GeneCards

DCX GeneCards

DPYSL5 GeneCards

SOX3 GeneCards

BTNL2 GeneCards

XPA GeneCards

RHOA GeneCards

HMOX1 GeneCards

PON1 GeneCards

ETS1 GeneCards

HADHB GeneCards

CCL3 GeneCards

SLC17A5 GeneCards

IL7 GeneCards

BGLAP GeneCards

ACVRL1 GeneCards

LMNB1 GeneCards

GTF2E2 GeneCards

HSPD1 GeneCards

FCGR3B GeneCards

IL1R1 GeneCards

CA2 GeneCards

CXCL10 GeneCards

SYP GeneCards

NEU1 GeneCards

GAPDH GeneCards

CCR5 GeneCards

MBL2 GeneCards

AKT2 GeneCards

CERS3 GeneCards

GJA8 GeneCards

SAG GeneCards

VCAM1 GeneCards

SELE GeneCards

NRTN GeneCards

TLR2 GeneCards

FEN1 GeneCards

LMNB2 GeneCards

FCGR2A GeneCards

BHLHA9 GeneCards

ITGAM GeneCards

SERPINA3 GeneCards

GSTM1 GeneCards

IL5 GeneCards

CXCR2 GeneCards

EDARADD GeneCards

MUC1 GeneCards

PDPN GeneCards

HGF GeneCards

DDB2 GeneCards

JUN GeneCards

MIF GeneCards

SYK GeneCards

CXCL13 GeneCards

FGF7 GeneCards

GGT1 GeneCards

ITGB2 GeneCards

LTA GeneCards

IL33 GeneCards

CYP3A4 GeneCards

RPA1 GeneCards

CETP GeneCards

VIP GeneCards

NOS1 GeneCards

CYP2D6 GeneCards

MMP1 GeneCards

ENO2 GeneCards

CYP2C19 GeneCards

PTPRC GeneCards

IL15 GeneCards

IL3 GeneCards

XPC GeneCards

CCL11 GeneCards

MIR125A GeneCards

AP4S1 GeneCards

KDM4C GeneCards

SELL GeneCards

CASP1 GeneCards

IL17RD GeneCards

TACR1 GeneCards

KRT1 GeneCards

HDAC1 GeneCards

NFKB1 GeneCards

RCAN2 GeneCards

IFNA2 GeneCards

CEP290 GeneCards

MECP2 GeneCards

ALDH3A2 GeneCards

CDH1 GeneCards

COL4A3 GeneCards

POLG GeneCards

COL1A2 GeneCards

TGFBR2 GeneCards

CFH GeneCards

INSR GeneCards

STAT3 GeneCards

CAV3 GeneCards

C3 GeneCards

PITX2 GeneCards

RELN GeneCards

NOTCH2 GeneCards

PDGFRA GeneCards

CD46 GeneCards

COL11A2 GeneCards

INS GeneCards

FOXC1 GeneCards

TREX1 GeneCards

CYLD GeneCards

COL18A1 GeneCards

SMAD3 GeneCards

EDNRB GeneCards

ATP13A2 GeneCards

CFB GeneCards

PRKCD GeneCards

SETBP1 GeneCards

ELN GeneCards

FERMT1 GeneCards

CTSC GeneCards

XIAP GeneCards

INPP5K GeneCards

STIM1 GeneCards

FLNB GeneCards

PSMB8 GeneCards

NAGLU GeneCards

MAPK1 GeneCards

TGM5 GeneCards

SCO2 GeneCards

MPZ GeneCards

TAP2 GeneCards

FRAXA GeneCards

H19 GeneCards

PPARG GeneCards

TAP1 GeneCards

ADIPOQ GeneCards

GTF2IRD1 GeneCards

CASP8 GeneCards

GTF2I GeneCards

MDM2 GeneCards

ITPR1 GeneCards

IGF1 GeneCards

LAMA3 GeneCards

RNU4ATAC GeneCards

HOXD13 GeneCards

NDN GeneCards

COLQ GeneCards

AICDA GeneCards

EDN1 GeneCards

SGSH GeneCards

PLOD2 GeneCards

POMC GeneCards

C1S GeneCards

KITLG GeneCards

ATP6V1B2 GeneCards

HFE GeneCards

CLN3 GeneCards

CRYAA GeneCards

GMPPB GeneCards

CYP19A1 GeneCards

GATA4 GeneCards

BSCL2 GeneCards

NOS3 GeneCards

PRL GeneCards

SLC6A4 GeneCards

COL7A1 GeneCards

OPA3 GeneCards

HCCS GeneCards

AMHR2 GeneCards

NR3C1 GeneCards

CXCR4 GeneCards

DHODH GeneCards

KRT14 GeneCards

SHBG GeneCards

CYP21A2 GeneCards

SERPINA1 GeneCards

CA8 GeneCards

APTX GeneCards

SIAE GeneCards

RETN GeneCards

C2 GeneCards

ISCA1 GeneCards

OCLN GeneCards

FLT3 GeneCards

ETV6 GeneCards

PLA2G6 GeneCards

CSF3 GeneCards

SLC12A2 GeneCards

PUF60 GeneCards

HESX1 GeneCards

ELANE GeneCards

ABHD5 GeneCards

MIR146A GeneCards

IL6R GeneCards

HTR2A GeneCards

SNCA GeneCards

CRH GeneCards

AGTR1 GeneCards

ACSL4 GeneCards

DNMT1 GeneCards

HP GeneCards

GUSB GeneCards

CLDN4 GeneCards

CTNS GeneCards

EDNRA GeneCards

CALCA GeneCards

CXCL12 GeneCards

PLAG1 GeneCards

MSTO1 GeneCards

CTDP1 GeneCards

CD55 GeneCards

COQ8A GeneCards

SOD1 GeneCards

ENSG00000259505 GeneCards

ENSG00000234586 GeneCards

ENSG00000244429 GeneCards

TFRC GeneCards

NCF1 GeneCards

CCN2 GeneCards

TPP1 GeneCards

ABCA1 GeneCards

SELP GeneCards

NTRK1 GeneCards

PDE8B GeneCards

ALDH3A1 GeneCards

EYS GeneCards

C4B GeneCards

LIPC GeneCards

FOXP2 GeneCards

PERP GeneCards

SATB1 GeneCards

SMPD1 GeneCards

HIF1A GeneCards

BAX GeneCards

HLA-DRA GeneCards

PHIP GeneCards

CDKN3 GeneCards

CLN8 GeneCards

LGALS4 GeneCards

TNFSF4 GeneCards

CYCS GeneCards

AGPAT2 GeneCards

SOCS1 GeneCards

TG GeneCards

GAST GeneCards

RAX GeneCards

PLS1 GeneCards

NPY GeneCards

VIM GeneCards

GAD2 GeneCards

CLDN3 GeneCards

NR3C2 GeneCards

TLR3 GeneCards

ATP1A2 GeneCards

CLU GeneCards

CLN5 GeneCards

AQP4 GeneCards

FADD GeneCards

FXN GeneCards

HSPA5 GeneCards

SPTBN2 GeneCards

CXCR5 GeneCards

IRF1 GeneCards

BMP6 GeneCards

TNFRSF13B GeneCards

VDR GeneCards

LOC110806262 GeneCards

OTOA GeneCards

KCND2 GeneCards

ANXA5 GeneCards

NOS2 GeneCards

DNASE1L3 GeneCards

KIR3DL1 GeneCards

ITGA4 GeneCards

ACP5 GeneCards

CST3 GeneCards

MCAM GeneCards

MAPK10 GeneCards

TOR1A GeneCards

MMP3 GeneCards

ACE2 GeneCards

ATXN2 GeneCards

MUC19 GeneCards

ACP1 GeneCards

IRAK1 GeneCards

BCL6 GeneCards

XDH GeneCards

NFIB GeneCards

BLK GeneCards

FLG GeneCards

TOP1 GeneCards

CXCR3 GeneCards

STS GeneCards

MYD88 GeneCards

FAM167A GeneCards

LAMP1 GeneCards

IFNG-AS1 GeneCards

S100A9 GeneCards

CAVIN1 GeneCards

BCL2 GeneCards

CXCR2P1 GeneCards

CCK GeneCards

ELOVL4 GeneCards

SUMF1 GeneCards

ATP6AP1 GeneCards

MERTK GeneCards

MME GeneCards

BCL10 GeneCards

LINC00426 GeneCards

LINC02384 GeneCards

SAA1 GeneCards

TLR9 GeneCards

HLA-DPB1 GeneCards

DNAJC3 GeneCards

NPC1 GeneCards

IL2RB GeneCards

AGMO GeneCards

IFNB1 GeneCards

PHYH GeneCards

SFTPD GeneCards

CX3CR1 GeneCards

HMGB1 GeneCards

ACADVL GeneCards

ITGAL GeneCards

IL17F GeneCards

PROP1 GeneCards

PARP1 GeneCards

TAF8 GeneCards

PCNA GeneCards

SAR1B GeneCards

TBK1 GeneCards

GBA2 GeneCards

XRCC5 GeneCards

ALPP GeneCards

MUC16 GeneCards

CR2 GeneCards

GSTT1 GeneCards

MAG GeneCards

CCR7 GeneCards

CD44 GeneCards

SCT GeneCards

S100A8 GeneCards

SIGLEC5 GeneCards

CENPB GeneCards

CD70 GeneCards

DAB1 GeneCards

IRAK1BP1 GeneCards

GZMB GeneCards

CTSD GeneCards

CENPC GeneCards

MIR200B GeneCards

M6PR GeneCards

XRCC6 GeneCards

AQP2 GeneCards

ARSA GeneCards

KRT7 GeneCards

HSPB1 GeneCards

FURIN GeneCards

PDCD1 GeneCards

HMGCR GeneCards

LCN2 GeneCards

IRF3 GeneCards

ITGA2 GeneCards

LAMB1 GeneCards

MLN GeneCards

ATP7B GeneCards

TGFA GeneCards

CCL4 GeneCards

LTBP1 GeneCards

PNPLA1 GeneCards

ATF6 GeneCards

TNFRSF13C GeneCards

KIR2DS4 GeneCards

CAT GeneCards

MIA2 GeneCards

CFLAR GeneCards

SRD5A1 GeneCards

DIABLO GeneCards

MICB GeneCards

TNPO3 GeneCards

ADCYAP1 GeneCards

NR1H2 GeneCards

CR1 GeneCards

FBL GeneCards

FLNC GeneCards

IRF7 GeneCards

NCAM1 GeneCards

CD274 GeneCards

ATP6V1B1 GeneCards

FCGR3A GeneCards

C5AR1 GeneCards

MALT1 GeneCards

MX1 GeneCards

EXOSC10 GeneCards

LEF1 GeneCards

KRT19 GeneCards

ALOX5 GeneCards

GOLGA1 GeneCards

LYZ GeneCards

RNASE3 GeneCards

C3AR1 GeneCards

CA4 GeneCards

IL23A GeneCards

PF4 GeneCards

CLN6 GeneCards

IRF4 GeneCards

SPTBN1 GeneCards

TFEB GeneCards

CD59 GeneCards

HLA-DRB5 GeneCards

EEF1A1 GeneCards

KCNC3 GeneCards

DNASE1 GeneCards

STAT5A GeneCards

MCOLN1 GeneCards

ADAM17 GeneCards

FES GeneCards

TNFRSF10A GeneCards

TNFSF10 GeneCards

MUC5B GeneCards

TNIP1 GeneCards

GRP GeneCards

IL1RL1 GeneCards

KIR2DL1 GeneCards

OBSCN GeneCards

CD80 GeneCards

CXCL9 GeneCards

TLR7 GeneCards

OGDH GeneCards

DEGS2 GeneCards

CHI3L1 GeneCards

BCL2L1 GeneCards

ID3 GeneCards

ITPR3 GeneCards

KRT16 GeneCards

IL17RA GeneCards

ARMS2 GeneCards

MAPK8 GeneCards

ADIPOR1 GeneCards

IFNAR1 GeneCards

CTSG GeneCards

PLIN1 GeneCards

LCN1 GeneCards

MIR142 GeneCards

PAX5 GeneCards

BANK1 GeneCards

VTI1B GeneCards

IL12B GeneCards

CHRM3 TTD

CD19 TTD

CTSS TTD

CD40 TTD

CCR9 TTD

IL7 TTD

CD40LG TTD

TNFRSF13C TTD

FCGR2B TTD

ICOSLG TTD

CASP3 SS

IL1B SS

VEGFA SS

AGT SS

CASP9 SS

MPO SS

NPPB SS

TNF SS

CYP3A4 SS

ALB SS

CAT SS

CCL2 SS

CDKN1A SS

CXCL8 SS

CYP1A2 SS

IL6 SS

LITAF SS

NR1D1 SS

PER1 SS

PPARD SS

PRELP SS

PRKCB SS

RELA SS

SMAD7 SS

SOD1 SS

TGFBI SS

TNFAIP2 SS

TP53 SS

IRAK1BP1 SS

STAT4 SS

DGKQ SS

GTF2I SS

TNFAIP3 SS

PRDM1 SS

PHIP SS

FCGR2A SS

IL12A SS

NCF1 SS

ITSN2 SS

TNIP1 SS

PTTG1 SS

TNFSF13B SS

TGFB1 SS

ID3 SS

THBS1 SS

FAS SS

HMOX1 SS

IL2 SS

IL2RA SS

AIRE SS

IL4 SS

ST14 SS

TRAF3IP2 SS

MAP3K14 SS

NFKBIA SS

E2F1 SS

RORC SS

CCR7 SS

CYP19A1 SS

CTSS SS

LYZ SS

BCL2 SS

PRKAR1A SS

CXADRP1 SS

ARR3 SS

IL17A SS

IL10 SS

ESR1 SS

SPG7 SS

STAT3 SS

IFNG SS

CASR SS

CXADR SS

TRIM13 SS

HBG1 SS

HBA2 SS

VCAM1 SS

NR1I3 SS

F5 SS

BCR SS

CFB SS

CCL25 SS

IL21 SS

TNFSF13 SS

RAD23B SS

PTN SS

MIR495 SS

B2M SS

ADIPOQ SS

ATF7IP SS

CX3CL1 SS

FAM167A SS

TACR1 SS

TFPI SS

ACE SS

BMP6 SS

TLR3 SS

VTCN1 SS

SLC6A4 SS

CDR3 SS

TSPYL2 SS

SELE SS

ERVK-6 SS

BLK SS

THBD SS

FCRL4 SS

CXCL12 SS

RBP4 SS

DYM SS

ERVK-20 SS

FLVCR1 SS

HBA1 SS

HBB SS

HBG2 SS

ERVW-1 SS

ICAM1 SS

AQP8 SS

FASLG SS

IL5 SS

GLO1 SS

PUF60 SS

ERVW-4 SS

CXCL13 SS

CCR9 SS

HPSE SS

CTLA4 SS

DECR1 SS

ERG SS

F2 SS

F2RL1 SS

IL7 SS

IL12RB1 SS

MMP3 SS

MMP9 SS

MTHFR SS

MUC1 SS

ACR SS

FURIN SS

IL21R SS

IL22 SS

PC SS

PLS3 SS

MMP2 SS

CXCL9 SS

AQP5 SS

IDO1 SS

AQP9 SS

ITGB2 SS

GDF6 SS

LBR SS

MIR10B SS

MIR155 SS

MDK SS

MIF SS

PMCH SS

CHRM1 SS

CHRM3 SS

CYP2D6 SS

FMO1 SS

DNA SS

TLR7 SS

TLR9 SS

ABCB1 SS

ORM1 SS

ORM2 SS

SLCO1A2 SS

ACE2 SS

CYP2C8 SS

CHRM2 SS

CYP2A6 SS

CHRM4 SS

SERPINA6 SS

NR3C1 SS

CYP2C19 SS

CYP3A5 SS

CYP3A43 SS

CYP3A7 SS

CYP1B1 SS

CYP2B6 SS

CYP2C9 SS

HSD11B1 SS

FBN1 SS

BRCA1 SS

PTEN SS

SMARCA4 SS

FGFR2 SS

ERCC6 SS

FLNA SS

TSC2 SS

NOD2 SS

COL3A1 SS

NLRP3 SS

GJB2 SS

RET SS

COL5A1 SS

FGFR3 SS

FGFR1 SS

ERCC8 SS

HSPG2 SS

HRAS SS

LMNA SS

SCN1A SS

KCNJ2 SS

IFIH1 SS

SCN10A SS

CHD7 SS

COL1A1 SS

AKT1 SS

COL5A2 SS

PLOD1 SS

EP300 SS

TNFRSF1A SS

TP63 SS

LRRC56 SS

MUSK SS

ERCC4 SS

PAX6 SS

FOXG1 SS

FOXP3 SS

ERCC2 SS

WDPCP SS

SLC12A3 SS

CD40LG SS

POLD1 SS

CACNB2 SS

HNF1B SS

ARID1B SS

PSTPIP1 SS

HLA-B SS

DSP SS

LRP2 SS

GJA1 SS

GJB6 SS

SCN11A SS

SMARCA2 SS

SPTAN1 SS

HLA-DRB1 SS

SBDS SS

FGF10 SS

MEFV SS

HARS1 SS

ACTB SS

ADA2 SS

ERCC1 SS

HPRT1 SS

BDNF SS

KCNJ10 SS

ADAMTS13 SS

ERCC5 SS

NDUFAF2 SS

MTOR SS

PGBD3 SS

SNRPN SS

SIL1 SS

TTR SS

LOC101448202 SS

POLR1C SS

KIT SS

FKBP14 SS

TLR4 SS

TH SS

AKT3 SS

JAK2 SS

LEP SS

SOX2 SS

CRLF1 SS

TBX4 SS

SREBF1 SS

EDAR SS

LOC113939944 SS

CRP SS

ABCC9 SS

ERCC3 SS

NECTIN1 SS

GDF5 SS

MSX1 SS

CP SS

GRIN2B SS

RYR1 SS

ERBB3 SS

EGFR SS

ZMPSTE24 SS

GPC3 SS

C4A SS

PAFAH1B1 SS

GSN SS

TNXB SS

CCNH SS

IARS2 SS

MPLKIP SS

WNT10A SS

MVK SS

GNAQ SS

AEBP1 SS

HELLS SS

HLA-A SS

TRIM21 SS

CTNNA1 SS

FGF9 SS

FGF8 SS

CFHR1 SS

APOE SS

SAMD9 SS

TTN SS

CCR6 SS

SSB SS

SCN9A SS

HLA-DQB1 SS

CD27 SS

CD40 SS

SYT2 SS

FKBP14-AS1 SS

APOH SS

CD4 SS

SLC39A13 SS

GDNF SS

IL1A SS

CUL4B SS

GTF2H5 SS

NGF SS

RNF125 SS

CD79A SS

U2AF1 SS

IL23R SS

CACNA1A SS

IKBKG SS

PCDH19 SS

EFL1 SS

ERCC8-AS1 SS

APOA1 SS

ADA SS

LOC102724058 SS

H2AC18 SS

HARS2 SS

EFEMP1 SS

ERBB2 SS

RAD51 SS

ERAP1 SS

AP1B1 SS

CCR1 SS

COL17A1 SS

CALR SS

IL1RN SS

USP7 SS

AFF2 SS

UVSSA SS

SYNGAP1 SS

MTTP SS

RTEL1 SS

BIVM-ERCC5 SS

DDX11 SS

TMPO SS

RO60 SS

CHD4 SS

ABL1 SS

KARS1 SS

REN SS

LTF SS

SST SS

DNAH8 SS

IKZF1 SS

KLRC4 SS

FGF3 SS

IL12A-AS1 SS

VWF SS

IL7R SS

STAT1 SS

MAB21L1 SS

PTPN22 SS

LPL SS

HLA-C SS

HADHA SS

MMP14 SS

UBAC2 SS

TARS1 SS

IL18 SS

FGF2 SS

FN1 SS

APOB SS

PSORS1C1 SS

PIK3C2A SS

EGF SS

SERPINC1 SS

EDA SS

TGM1 SS

VCP SS

HLA-DQA1 SS

VAMP1 SS

ZNRD2 SS

NKX2-1 SS

CFTR SS

TPO SS

CD28 SS

GPT SS

P4HB SS

CCL5 SS

FGF17 SS

IL13 SS

AMPH SS

DENND11 SS

PRTN3 SS

CSF2 SS

PSMB9 SS

CHUK SS

TNFRSF1B SS

AIMP1 SS

PLCB1 SS

RNF113A SS

IFNA1 SS

CD8A SS

SSNA1 SS

PRF1 SS

MUC5AC SS

IRF5 SS

DDB1 SS

POLR1A SS

NBEA SS

OTULIN SS

SPP1 SS

NLRP1 SS

ICOSLG SS

GATAD2B SS

FGF23 SS

DCX SS

DPYSL5 SS

SOX3 SS

BTNL2 SS

XPA SS

RHOA SS

PON1 SS

ETS1 SS

HADHB SS

CCL3 SS

SLC17A5 SS

BGLAP SS

ACVRL1 SS

LMNB1 SS

GTF2E2 SS

HSPD1 SS

FCGR3B SS

IL1R1 SS

CA2 SS

CXCL10 SS

SYP SS

NEU1 SS

GAPDH SS

CCR5 SS

MBL2 SS

AKT2 SS

CERS3 SS

GJA8 SS

SAG SS

NRTN SS

TLR2 SS

FEN1 SS

LMNB2 SS

BHLHA9 SS

ITGAM SS

SERPINA3 SS

GSTM1 SS

CXCR2 SS

EDARADD SS

PDPN SS

HGF SS

DDB2 SS

JUN SS

SYK SS

FGF7 SS

GGT1 SS

LTA SS

IL33 SS

RPA1 SS

CETP SS

VIP SS

NOS1 SS

MMP1 SS

ENO2 SS

PTPRC SS

IL15 SS

IL3 SS

XPC SS

CCL11 SS

MIR125A SS

AP4S1 SS

KDM4C SS

SELL SS

CASP1 SS

IL17RD SS

KRT1 SS

HDAC1 SS

NFKB1 SS

RCAN2 SS

IFNA2 SS

CEP290 SS

MECP2 SS

ALDH3A2 SS

CDH1 SS

COL4A3 SS

POLG SS

COL1A2 SS

TGFBR2 SS

CFH SS

INSR SS

CAV3 SS

C3 SS

PITX2 SS

RELN SS

NOTCH2 SS

PDGFRA SS

CD46 SS

COL11A2 SS

INS SS

FOXC1 SS

TREX1 SS

CYLD SS

COL18A1 SS

SMAD3 SS

EDNRB SS

ATP13A2 SS

PRKCD SS

SETBP1 SS

ELN SS

FERMT1 SS

CTSC SS

XIAP SS

INPP5K SS

STIM1 SS

FLNB SS

PSMB8 SS

NAGLU SS

MAPK1 SS

TGM5 SS

SCO2 SS

MPZ SS

TAP2 SS

FRAXA SS

H19 SS

PPARG SS

TAP1 SS

GTF2IRD1 SS

CASP8 SS

MDM2 SS

ITPR1 SS

IGF1 SS

LAMA3 SS

RNU4ATAC SS

HOXD13 SS

NDN SS

COLQ SS

AICDA SS

EDN1 SS

SGSH SS

PLOD2 SS

POMC SS

C1S SS

KITLG SS

ATP6V1B2 SS

HFE SS

CLN3 SS

CRYAA SS

GMPPB SS

GATA4 SS

BSCL2 SS

NOS3 SS

PRL SS

COL7A1 SS

OPA3 SS

HCCS SS

AMHR2 SS

CXCR4 SS

DHODH SS

KRT14 SS

SHBG SS

CYP21A2 SS

SERPINA1 SS

CA8 SS

APTX SS

SIAE SS

RETN SS

C2 SS

ISCA1 SS

OCLN SS

FLT3 SS

ETV6 SS

PLA2G6 SS

CSF3 SS

SLC12A2 SS

HESX1 SS

ELANE SS

ABHD5 SS

MIR146A SS

IL6R SS

HTR2A SS

SNCA SS

CRH SS

AGTR1 SS

ACSL4 SS

DNMT1 SS

HP SS

GUSB SS

CLDN4 SS

CTNS SS

EDNRA SS

CALCA SS

PLAG1 SS

MSTO1 SS

CTDP1 SS

CD55 SS

COQ8A SS

ENSG00000259505 SS

ENSG00000234586 SS

ENSG00000244429 SS

TFRC SS

CCN2 SS

TPP1 SS

ABCA1 SS

SELP SS

NTRK1 SS

PDE8B SS

ALDH3A1 SS

EYS SS

C4B SS

LIPC SS

FOXP2 SS

PERP SS

SATB1 SS

SMPD1 SS

HIF1A SS

BAX SS

HLA-DRA SS

CDKN3 SS

CLN8 SS

LGALS4 SS

TNFSF4 SS

CYCS SS

AGPAT2 SS

SOCS1 SS

TG SS

GAST SS

RAX SS

PLS1 SS

NPY SS

VIM SS

GAD2 SS

CLDN3 SS

NR3C2 SS

ATP1A2 SS

CLU SS

CLN5 SS

AQP4 SS

FADD SS

FXN SS

HSPA5 SS

SPTBN2 SS

CXCR5 SS

IRF1 SS

TNFRSF13B SS

VDR SS

LOC110806262 SS

OTOA SS

KCND2 SS

ANXA5 SS

NOS2 SS

DNASE1L3 SS

KIR3DL1 SS

ITGA4 SS

ACP5 SS

CST3 SS

MCAM SS

MAPK10 SS

TOR1A SS

ATXN2 SS

MUC19 SS

ACP1 SS

IRAK1 SS

BCL6 SS

XDH SS

NFIB SS

FLG SS

TOP1 SS

CXCR3 SS

STS SS

MYD88 SS

LAMP1 SS

IFNG-AS1 SS

S100A9 SS

CAVIN1 SS

CXCR2P1 SS

CCK SS

ELOVL4 SS

SUMF1 SS

ATP6AP1 SS

MERTK SS

MME SS

BCL10 SS

LINC00426 SS

LINC02384 SS

SAA1 SS

HLA-DPB1 SS

DNAJC3 SS

NPC1 SS

IL2RB SS

AGMO SS

IFNB1 SS

PHYH SS

SFTPD SS

CX3CR1 SS

HMGB1 SS

ACADVL SS

ITGAL SS

IL17F SS

PROP1 SS

PARP1 SS

TAF8 SS

PCNA SS

SAR1B SS

TBK1 SS

GBA2 SS

XRCC5 SS

ALPP SS

MUC16 SS

CR2 SS

GSTT1 SS

MAG SS

CD44 SS

SCT SS

S100A8 SS

SIGLEC5 SS

CENPB SS

CD70 SS

DAB1 SS

GZMB SS

CTSD SS

CENPC SS

MIR200B SS

M6PR SS

XRCC6 SS

AQP2 SS

ARSA SS

KRT7 SS

HSPB1 SS

PDCD1 SS

HMGCR SS

LCN2 SS

IRF3 SS

ITGA2 SS

LAMB1 SS

MLN SS

ATP7B SS

TGFA SS

CCL4 SS

LTBP1 SS

PNPLA1 SS

ATF6 SS

TNFRSF13C SS

KIR2DS4 SS

MIA2 SS

CFLAR SS

SRD5A1 SS

DIABLO SS

MICB SS

TNPO3 SS

ADCYAP1 SS

NR1H2 SS

CR1 SS

FBL SS

FLNC SS

IRF7 SS

NCAM1 SS

CD274 SS

ATP6V1B1 SS

FCGR3A SS

C5AR1 SS

MALT1 SS

MX1 SS

EXOSC10 SS

LEF1 SS

KRT19 SS

ALOX5 SS

GOLGA1 SS

RNASE3 SS

C3AR1 SS

CA4 SS

IL23A SS

PF4 SS

CLN6 SS

IRF4 SS

SPTBN1 SS

TFEB SS

CD59 SS

HLA-DRB5 SS

EEF1A1 SS

KCNC3 SS

DNASE1 SS

STAT5A SS

MCOLN1 SS

ADAM17 SS

FES SS

TNFRSF10A SS

TNFSF10 SS

MUC5B SS

GRP SS

IL1RL1 SS

KIR2DL1 SS

OBSCN SS

CD80 SS

OGDH SS

DEGS2 SS

CHI3L1 SS

BCL2L1 SS

ITPR3 SS

KRT16 SS

IL17RA SS

ARMS2 SS

MAPK8 SS

ADIPOR1 SS

IFNAR1 SS

CTSG SS

PLIN1 SS

LCN1 SS

MIR142 SS

PAX5 SS

BANK1 SS

VTI1B SS

IL12B SS

CD19 SS

FCGR2B SS
