# Supplementary material for: Decoding the Mechanism of CheReCunJin Formula in Treating Sjögren's Syndrome Based on Network Pharmacology and Molecular Docking
Source: Evid Based Complement Alternat Med. 2022 Sep 20;2022:1193846. doi: 10.1155/2022/1193846 (PMC9553462; doi:10.1155/2022/1193846)
Supplement: Supplementary Materials — Table S1: the active ingredients of CRCJ. Table S2: the nodes and edges of the network. Table S3: summary table of drug targets. Table S4: summary table of disease targets. Table S5: network cluster results. [file 1193846.f1.zip › 1193846.f1/Supplementary Table 5.docx]

Supplementary Table 5

Cluster Score (Density*#Nodes) Nodes Edges Node IDs

1 58.417 73 2103 VEGFA, ACE, HMOX1, MMP2, TGFB1, IFNG, CAT, FGF2, REN, MMP1, CXCL8, SPP1, MMP3, SELE, IGF1, EDN1, NFKBIA, IL1A, IL17A, IL2, TNFRSF1A, BDNF, IL4, IL13, CD40LG, LTA, CCL5, MPO, CCL2, CXCR4, APOE, CRP, IL1B, ICAM1, PPARG, VCAM1, ALB, NOS3, LEP, TLR3, CSF2, MMP9, TLR4, CXCL10, IL10, ADIPOQ, CYCS, AKT1, CD28, IL6, NOS2, INS, TNF, STAT5A, CASP8, EGF, TP53, ERBB2, STAT1, EGFR, KIT, FASLG, BCL2L1, NGF, CASP3, CD40, FOXP3, MTOR, JUN, PTEN, MYD88, CCL3, HIF1A

2 5.273 12 29 GSTM1, SCN9A, SCN1A, CACNA1A, CYP1A2, SCN10A, CYP2C9, SLC6A4, HTR2A, CACNB2, CAV3, KCND2

3 4.583 25 55 IL1RN, CXCL13, APOB, C3, APOA1, SELP, NFKB1, IRF1, NCF1, CCR7, HSPB1, LCN2, GPT, AGTR1, NOD2, AGT, NR3C1, FAS, CHUK, CASP9, CDKN1A, CX3CR1, ESR1, ELANE, TNFRSF1B

4 3.5 13 21 RET, ABCB1, ABCA1, PON1, HMGCR, TH, CALCA, LPL, BAX, NOS1, SNCA, SREBF1, FADD

5 3.2 6 8 F2, HDAC1, COL1A1, BRCA1, VDR, BGLAP

6 3 3 3 PRKCB, ITPR3, ITPR1

7 3 3 3 HBA1, HBA2, CA2
